# Author Correction: Expanding chemical space by *para*-C−H arylation of arenes

**DOI:** 10.1038/s41467-022-32720-3

**Published:** 2022-08-25

**Authors:** Sudip Maiti, Yingzi Li, Sheuli Sasmal, Srimanta Guin, Trisha Bhattacharya, Goutam Kumar Lahiri, Robert S. Paton, Debabrata Maiti

**Affiliations:** 1 Department of Chemistry, Indian Institute of Technology Bombay, Mumbai, 400076 India; 2 Department of Chemistry, Colorado State University, Fort Collins, CO 80523 USA; 3 IDP in Climate Studies, Indian Institute of Technology Bombay, 400076 Mumbai, India

**Keywords:** Homogeneous catalysis, Catalytic mechanisms

Correction to: *Nature Communications* 10.1038/s41467-022-31506-x, published online 08 July 2022

The original version of this Article contained an error in Fig. 1 and Fig. 3. In the original version of Figs. 1 and 3, the sugars were drawn with the L form instead of the D form. The sugars in this work were obtained from the naturally occurring D enantiomers.

The correct version of Fig. 1 is:
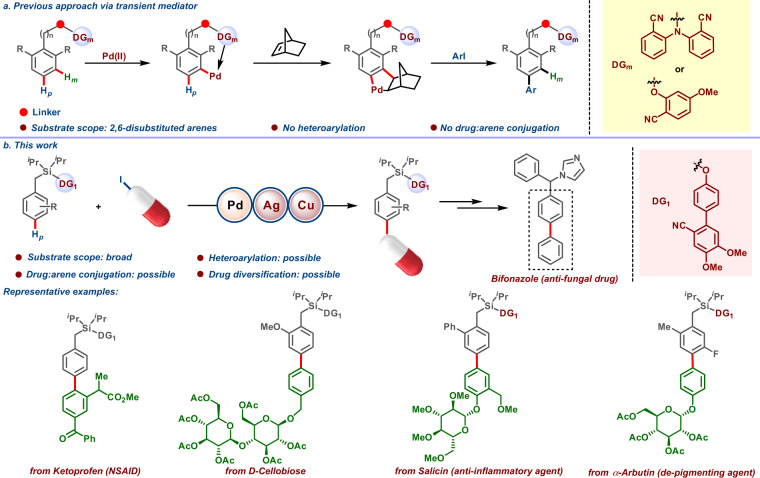


which replaces the previous incorrect version:
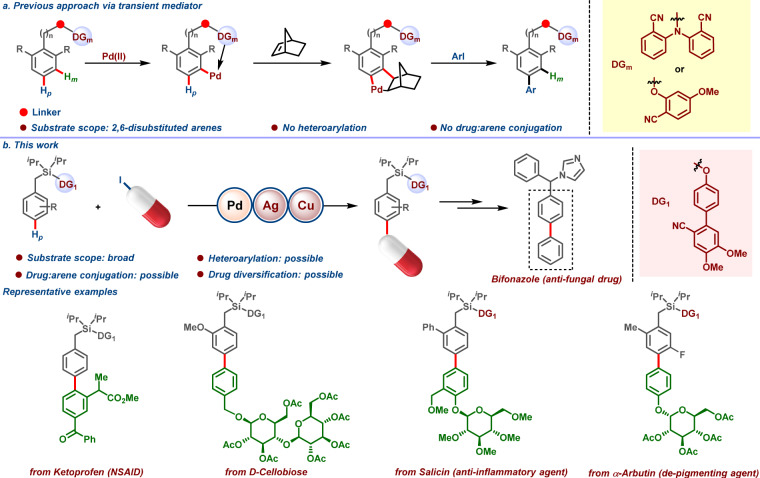


The correct version of Fig. 3 is:
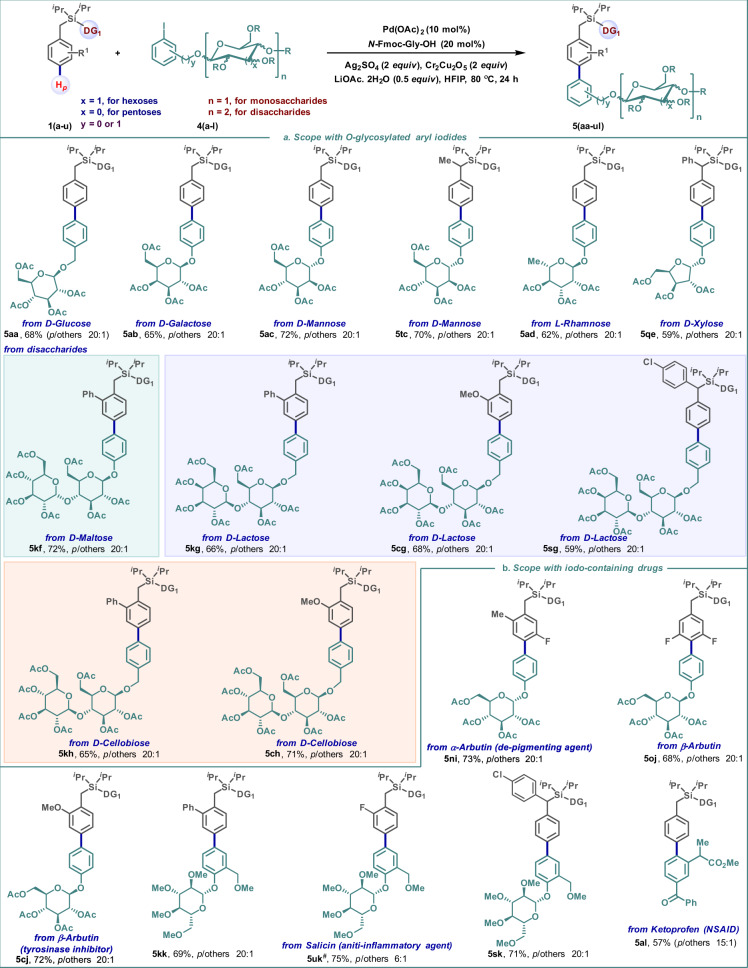


which replaces the previous incorrect version:
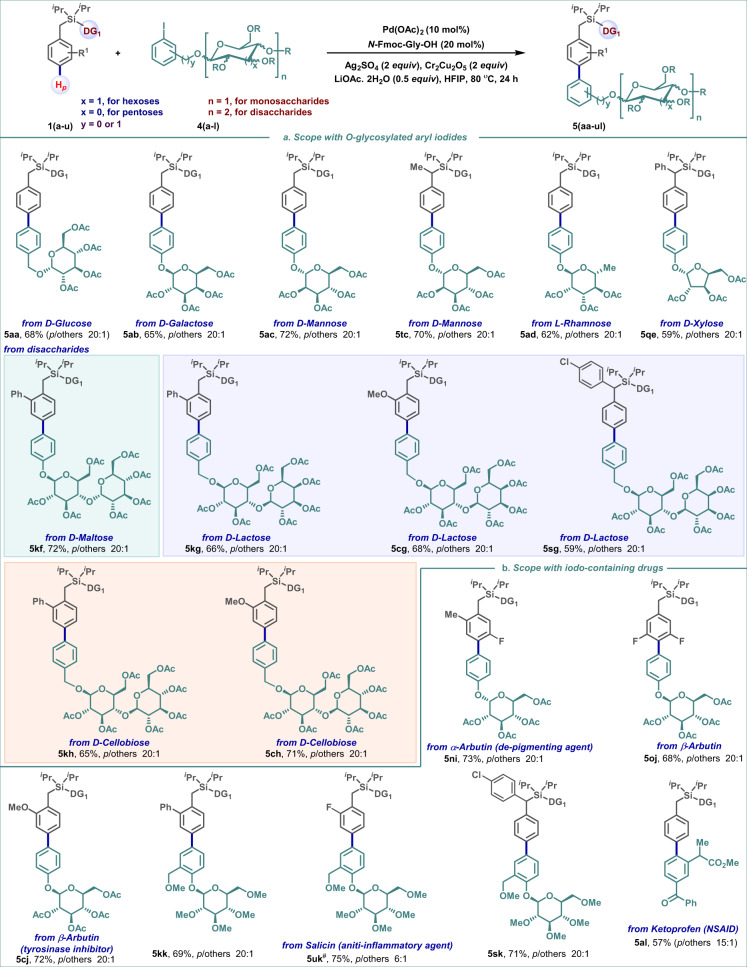


This has been corrected in both the PDF and HTML versions of the Article.

