# Supplementary material for: Expanding chemical space by para-C−H arylation of arenes
Source: Nat Commun. 2022 Jul 8;13:3963. doi: 10.1038/s41467-022-31506-x (PMC9270437; doi:10.1038/s41467-022-31506-x)
Supplement: Supplementary file 1 — Supplementary Information [file 41467_2022_31506_MOESM1_ESM.pdf]

Supplementary Information

Expanding chemical space by *para*-C-H arylation of arenes

Sudip Maiti, Yingzi Li, Sheuli Sasmal, Srimanta Guin, Trisha Bhattacharya, Goutam Kumar Lahiri, Robert S. Paton & Debabrata Maiti

Department of Chemistry, Indian Institute of Technology Bombay, Mumbai, India. dmaiti@iitb.ac.in

Department of Chemistry, Colorado State University, Fort Collins, Colorado 80523, USA. robert.paton@colostate.edu

Department of Chemistry, Indian Institute of Technology Bombay, Mumbai, India. lahiri@chem.iitb.ac.in

Table of Contents

Supplementary Methods

1. General Considerations……………………………………………………S3
2. Experimental Section
   2.1. Optimization details for *para*-C−H arylation………………………..S4-S10
   2.2. Screening of Template……………………………………………….S11
   2.3. Preparation of Starting materials…………………………………….S11-S20
   2.4. General procedure for palladium catalyzed *para*-selective C−H arylation of arene……………………………………………………………S20-S22
   2.5. Determination of *para*-selectivity…………………………………...S22-S30
   2.6. Characterization data of *para*-arylated products…………………….S30-S81
   2.7. Mechanistic studies…………………………………………………..S81-S87
   2.8. Computational methods……………………………………………...S88-S93

Supplementary Discussion

3. X-Ray Crystallographic Data…………………………………………S94-S95
4. NMR Spectra………………………………………………………..S95-S173

Supplementary References……………………………………………………S174-S175

1. General Considerations

1.1. Reagent Information

Unless otherwise stated, all reactions were carried out under atmospheric condition in screw cap reaction tubes. All the commercial materials and solvents were used as received unless otherwise noted. DCM was dried by distillation over CaH_2. THF was dried by distillation over sodium/benzophenone. For column chromatography, silica gel (100–200 mesh) obtained from SRL Co. and neutral activated alumina from Merck was used. A gradient elution using petroleum ether and ethyl acetate was performed, based on Merck aluminum TLC sheets (silica gel 60F254). All the benzyl chlorides and bromides were bought from Sigma Aldrich/Alfa Aesar (India)/TCI (India)/Spectrochem. $Pd(OAc)_2$ (XX%, Alfa Aesar), LiOAc. $2H_2O$ (Spectrochem), Fmoc-Gly-OH (Spectrochem), Ag_2SO_4 (Sigma Aldrich), $Cu_2Cr_2O_5$ (Sigma Aldrich) were used in the Pd-catalyzed arylation reactions.

1.2. Analytical Information

All isolated compounds were characterized by ^{1}H NMR, ^{13}C NMR spectroscopy, high resolution mass spectrometry (HRMS) and infrared spectroscopy (IR). All Nuclear Magnetic Resonance spectra were recorded on Bruker 400 MHz and 500 MHz instruments. NMR spectra were reported in units, parts per million (ppm), using residual solvent peaks (7.26 ppm for ^{1}H NMR and 77.23 ppm for ^{13}C NMR in CDCl3) as internal reference. All ^{13}C NMR spectra were obtained with ^{1}H decoupling. Multiplicities are recorded as: s = singlet, d = doublet, t = triplet, dd = doublet of doublets, td = triplet of doublets, br s = broad singlet, m = multiplet. High-resolution mass spectra (HRMS) were recorded on a Q-TOF micromass (YA-105) mass spectrometer and a Bruker Maxis Impact (282001.00081) in ESI mode.

1.3. Description of Reaction Tube:

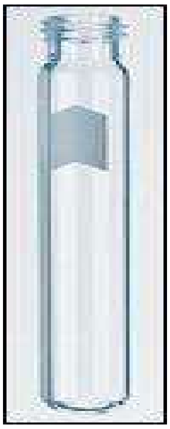

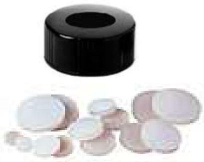

Pictorial description of reaction tube for *para*-olefination: Fisher brand Disposable Borosilicate Glass Tubes (16*125mm) with Threaded End (Fisher Scientific Order No. 1495935A) [left]; Kimble Black Phenolic Screw Thread Closures with Open Tops (Fisher Scientific Order No. 033407E); Thermo Scientific National PTFE/Silicone Septa for Sample Screw Thread Caps (Fisher Scientific Order No. 03394A).

2. Experimental Section

2.1. Optimization details for *para*-C−H arylation

[a]Yield are based on HPLC of the crude reaction mixture using acetophenone as internal standard. [b]Selectivity are based on ^{1}H NMR of the crude reaction mixture. Singlet of benzylic proton in ^{1}H NMR was used to measure the selectivity. nd, not detected

Supplementary Table 1. Palladium catalyst Optimization

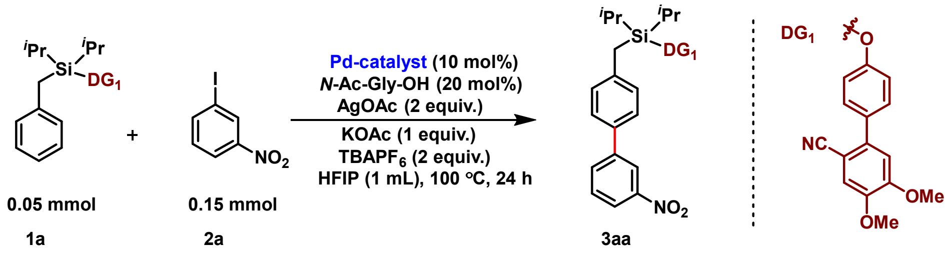

Entry	Pd-catalyst	Yield[a] % (*p*:others)[b]
**1**	**$Pd(OAc)_2$**	**25 (4:1)**
2	$Pd(OPiv)_2$	15 (4:1)
3	$Pd(TFA)_2$	Trace
4	$Pd(COD)Cl_2$	Trace
5	$Pd(PhCN)_2Cl_2$	10 (4:1)
6	$Pd(acac)_2$	Nd
7	$Pd(PPh_3)_2Cl_2$	7
8	$Pd(PPh_3)_4$	10 (4:1)
9	$Pd_2(dba)_3$	6
10	$Pd(CH_3CN)_2Cl_2$	Nd
11	$Pd(dppf)Cl_2$	Nd

Supplementary Table 2. Silver salt Optimization

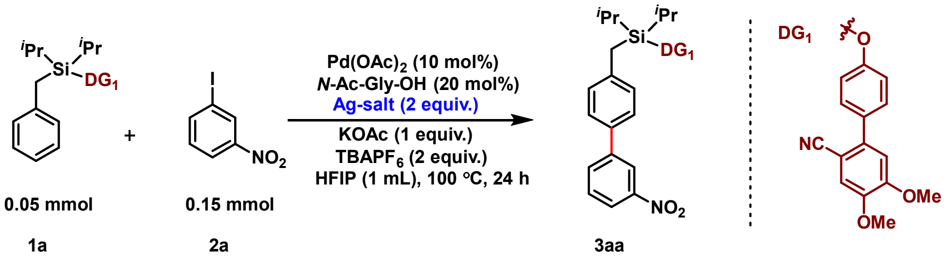

Entry	Ag-Salt	Yield[a] % (*p*:others)[b]
**1**	**AgOAc**	**25 (4:1)**
2	Ag_2CO_3	7
3	AgTFA	Nd
4	$AgNO_2$	Nd
5	$AgNO_3$	Nd
6	AgI	Nd
**7**	**Ag_2SO_4**	**24 (4:1)**
8	Ag_2O	Trace
9	AgCN	Nd
10	$AgBF_4$	Nd
11	$AgSbPF_6$	Nd
12	$AgPF_6$	nd
13	AgF	nd

Supplementary Table 3. Control experiment

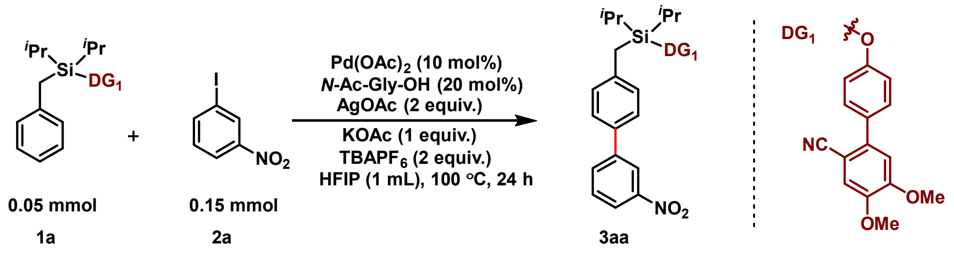

Entry	$Pd(OAc)_2$	*N*-Ac-Gly-OH	AgOAc	KOAc	$TBAPF_6$	Yield[a] %
1	-	✓	✓	✓	✓	nd
2	✓	-	✓	✓	✓	<5
3	✓	✓	-	✓	✓	<5
4	✓	✓	✓	-	✓	<5
**5**	✓	✓	✓	✓	-	**50**

Supplementary Table 4. Base Optimization

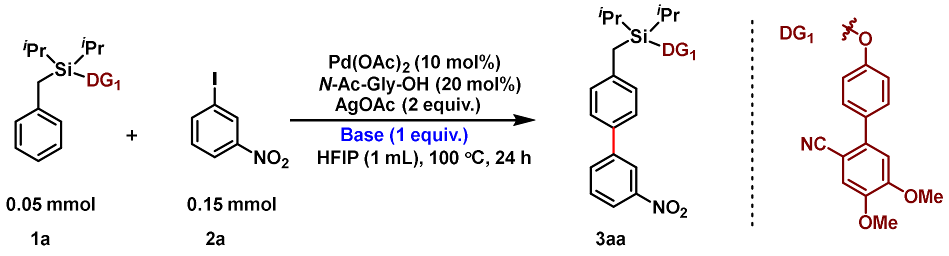

Entry	Base	Yield[a] % (*p*:others)[b]
1	KOAc	50 (5:1)
2	NaOAc	40 (5:1)
**3**	**LiOAc.2H_2O**	**55 (5:1)**
4	HCO_2Na	24
5	CF_3CO_2Na	10
6	Na_2CO_3	20
7	$NaHCO_3$	10
8	K_2CO_3	12
9	$KHCO_3$	7
10	Li_2CO_3	15
11	NaO*t*Bu	10
12	KO*t*Bu	12
13	LiO*t*Bu	9
14	NaOH	8
15	KOH	5
16	LiOH	20
17	Cs_2CO_3	trace
18	K_3PO_4	nd
19	NaOPiv	15
20	NaH_2PO_4	nd

Supplementary Table 5. Copper Salt Optimization

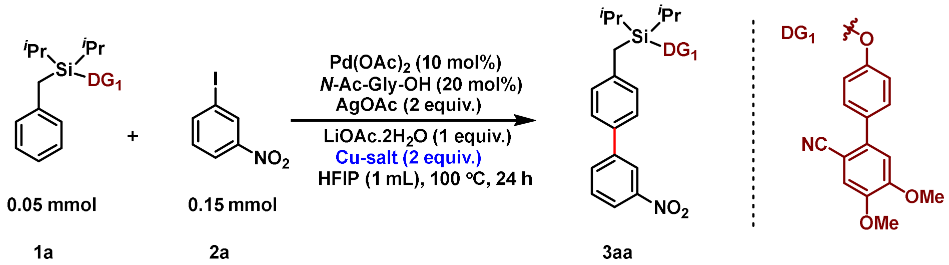

Entry	Cu-Salt	Yield[a] % (*p*:others)[b]
1	CuCl	35
2	$CuCl_2$	15
3	$Cu(OAc)_2$	47
4	CuOAc	18
5	CuO	30
**6**	**$Cu_2Cr_2O_5$**	**57 (5:1)**
7	CuF2	12
8	$Cu(NO_3)_2.3H_2O$	10
9	$CuBr_2$	trace
10	CuCN	nd
11	CuSCN	nd
12	$Cu(OTf)_2$	trace

Supplementary Table 6. Copper, silver salt and base combination Optimization

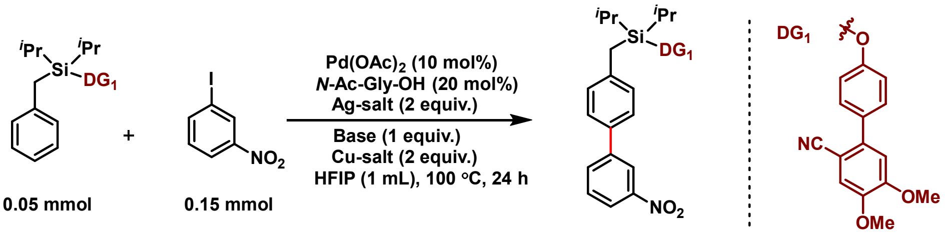

0.05 mmol 0.15 mmol

1a 2a 3aa

Entry	Ag-salt	Cu-salt	Base	Yield[a] % (*p*:others)[b]
1	AgOAc	$Cu_2Cr_2O_5$	KOAc	50
2	Ag_2SO_4	$Cu_2Cr_2O_5$	KOAc	58
**3**	**Ag_2SO_4**	**$Cu_2Cr_2O_5$**	**$LiOAc.2H_2O$**	**70 (6:1)**
4	Ag_2SO_4	$Cu(OAc)_2$	$LiOAc.2H_2O$	45
5	Ag_2SO_4	$Cu(OAc)_2$	KOAc	42

Supplementary Table 7. Optimization of amount of Ag_2SO_4 and $Cu_2Cr_2O_5$

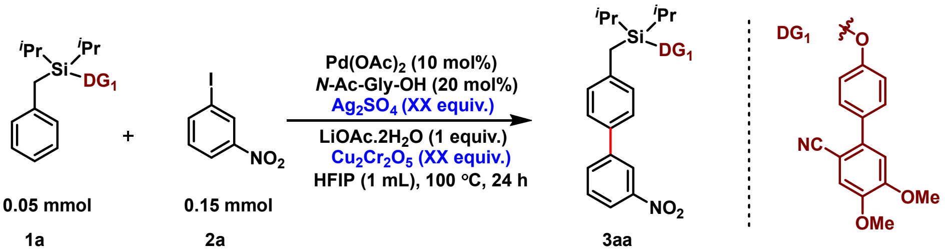

0.05 mmol 0.15 mmol

1a 2a 3aa

Entry	Ag_2SO_4 (equiv.)	$Cu_2Cr_2O_5$ (equiv.)	Yield[a] % (*p*:others)[b]
1	1	1	45
2	1	2	55
3	1	3	53
4	2	1	47
**5**	**2**	**2**	**70 (6:1)**
6	2	3	65

Supplementary Table 8. Optimization of amount of LiOAc.2H2O

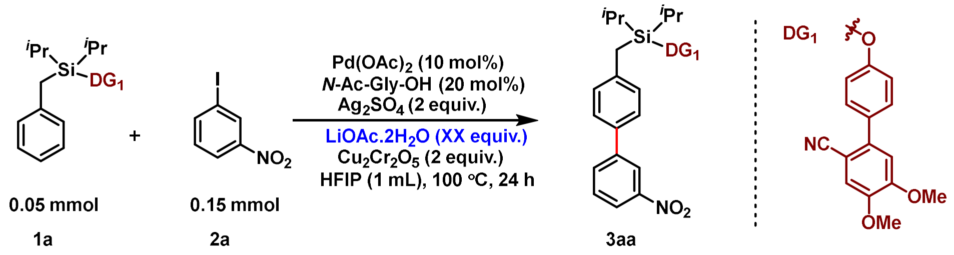

Entry	LiOAc.2H2O (equiv.)	Yield[a] % (*p*:others)[b]
1	0.1	55
2	0.2	57
3	0.3	60
4	0.4	69
**5**	**0.5**	**75 (6:1)**
6	0.6	72
7	0.8	71
8	1	70

Supplementary Table 9. Ligand Optimization

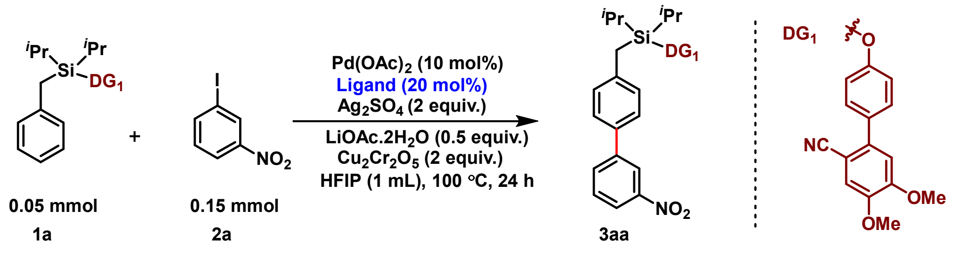

Entry	Ligand	Yield[a] % (*p*:others)[b]
1	*N*-Ac-Gly-OH	75 (6:1)
2	*N*-Ac-L-Leucine	55 (5:1)
3	N-Ac-L-Valine	65 (6:1)

4	*N*-Ac-D-Leu-OH	62 (4:1)
5	*N*-Ac-DL-Valine	68 (7:1)
6	*N*-Ac-DL-Trp-OH	Trace
7	*N*-Ac-DL-Norleucine	60 (7:1)
8	*N*-Ac-DL-2-Phenylglycine	52 (3:1)
9	*N*-Ac-4-hydroxy-L-Proline	45 (4:1)
10	*N*-Boc-Leu-OH	55 (4:1)
11	*N*-Boc-L-Isoleucine	50 (5:1)
12	*N*-Boc-L-Phenylalanine	59 (2:1)
13	*N*-Boc-L-Phenylglycine	48 (3:1)
14	*N*-Boc-D-Valine	52 (6:1)
15	*N*-Boc-L-Aspartic acid	trace
16	*N*-Boc-Glycine	62 (5:1)
17	*N*-Boc-L-*tert*-Leucine	54 (3:1)
18	*N*-Form-Glycine	45 (3:1)
19	*N*-Boc-D-Serine	44
20	*N*-Boc-L-Tyrosine	49
21	*N*-Boc-L-Alanine	43
22	*N*-Boc-4-Nitro-L-Phenylalanine	trace
23	*N*-Boc-Glu-OH	nd
24	Fmoc-L-Glutamine	nd
25	Fmoc-L-Leucine	60 (8:1)
**26**	**Fmoc-Glycine**	**75 (12:1)**
27	Fmoc-Alanine	65 (4:1)
28	Fmoc-L-Methionine	40 (3:1)
29	Fmoc-L-Threonine	35 (2:1)
30	N-Carbobenzoxy-DL-valine	nd

Supplementary Table 10. Optimization of amount of aryl iodide

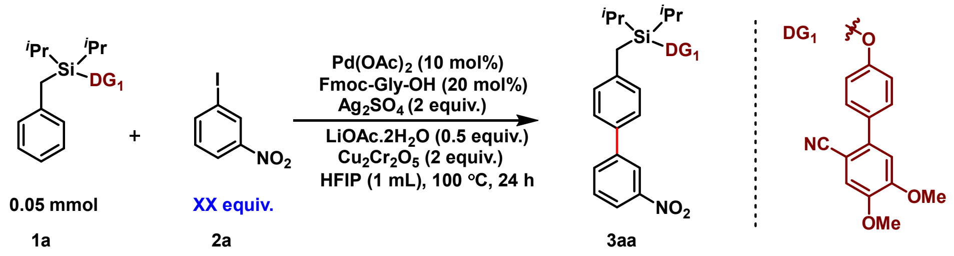

Entry	Amount (equiv.)	Yield[a] % (*p*:others)[b]
**1**	**1**	**85 (15:1)**
2	1.5	83 (14:1)
3	2	80
4	2.5	76

5	3	75 (12:1)

Supplementary Table 11. Temperature Optimization

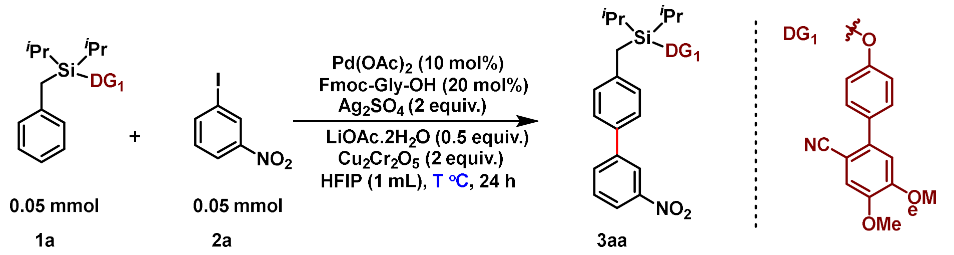

Entry	Temperature °C	Yield[a] % (*p*:others)[b]
1	40	45
2	50	50
3	60	58
4	70	75
**5**	**80**	**89 (15:1)**
6	90	85
7	100	85

Supplementary Table 12. Solvent Optimization

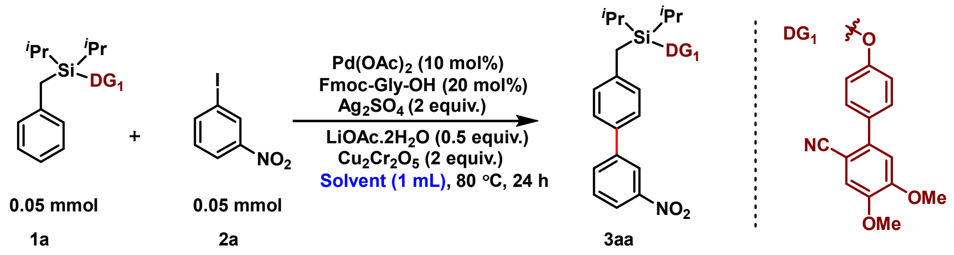

Entry	Solvent	Yield[a] % (*p*:others)[b]
**1**	**HFIP**	**89 (15:1)**
2	TFT	20
3	TFE	15
4	DCE	25
5	1,4-Dioxane	18
6	TBME	9
7	2-Propanol	6
8	MeOH	nd
9	$CHCl_3$	trace
10	CH_2Cl_2	5
11	THF	7

2.2. Screening of Template

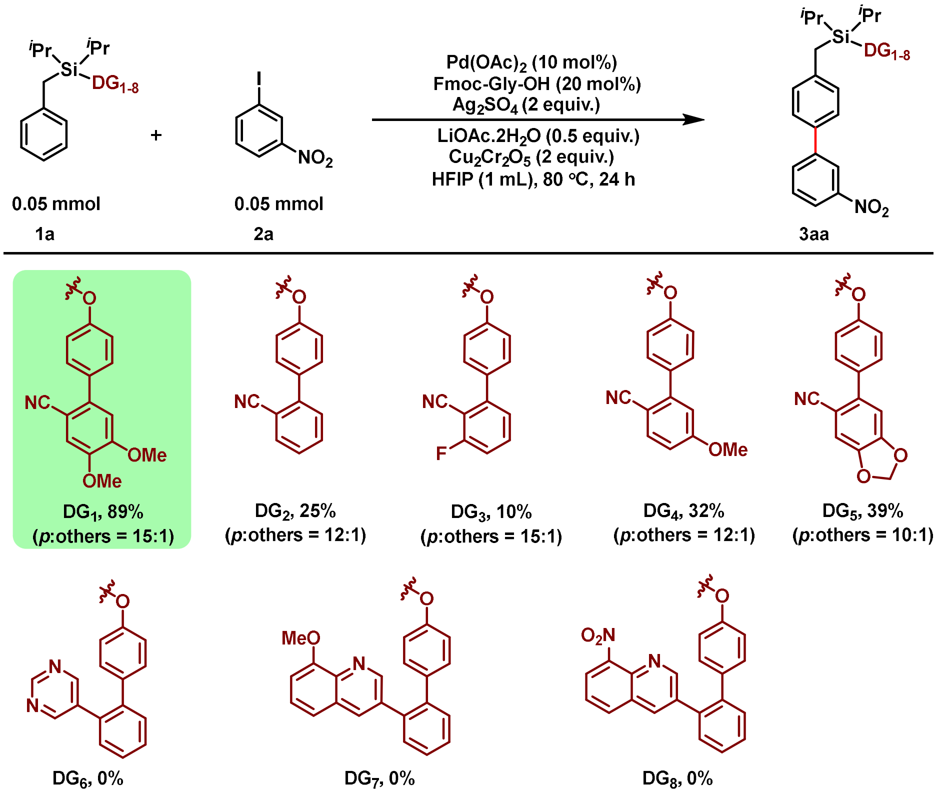

2.3. Preparation of Starting materials

2.3.1. Synthesis of benzylsilane ether derivatives:

Step 1:

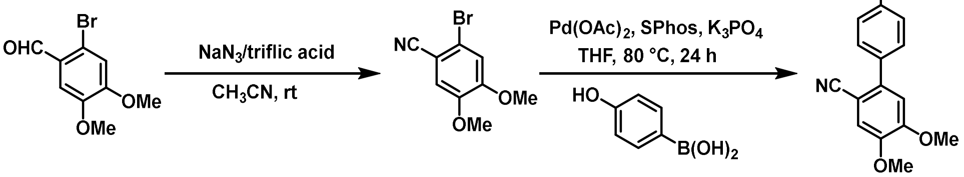

Step 2 and Step 3:

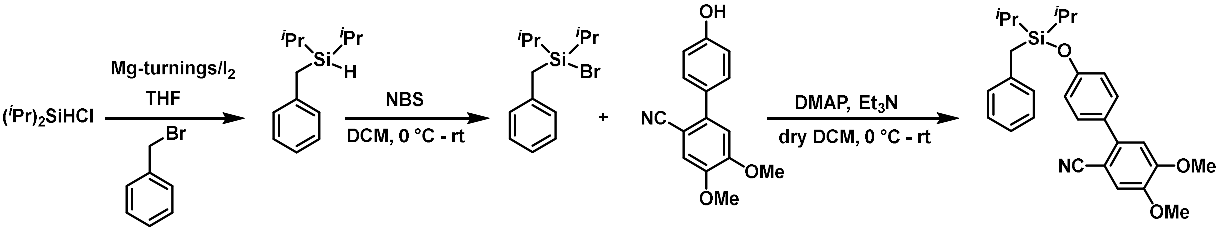

Step 1: Preparation of 4'-hydroxy-4,5-dimethoxy-[1,1'-biphenyl]-2-carbonitrile:

(a) In an oven dried round bottom flask (250 mL), charged with stir-bar, aldehyde substrate (A) (20 mmol) and NaN_3 (3 equiv.) were taken. MeCN (60 mL) was added to it and stirred at room temperature for 15 mins. 3.5 equiv. of triflic acid was added to the mixture in portion with a plastic dropper. After the addition the reaction was allowed to stir at room temperature for 6 h. Upon completion the reaction was diluted with ethyl acetate and the organic solvent was evaporated under reduced pressure. The solid residue was dissolved in ethyl acetate and washed with saturated $NaHCO_3$ solution (3 times). The organic fraction was then dried over anhydrous Na_2SO_4 and purified through column chromatography.[1] Quantitative conversion; white solid.

(b) In an oven dried reaction tube, charged with stir-bar, Pd $(OAc)_2$ (3 mol%), S-phos (6 mol%), B (3 mmol), 4-hydroxyphenyl boronic acid (3.5 mmol) and K_3PO_4 (3 equiv.) were added. The reaction tubes were capped with Teflon cap and purged with N_2 using schlenk line set up. THF was added to the reaction mixture (5 mL) and submerged in a preheated 100 °C oil bath and allowed for vigorous stirring for 24 hours. After 24 hour, reaction mixture was allowed to cool and diluted with EtOAc and extracted with brine solution. The organic layer was dried over $Na_2SO_4$and concentrated by evaporation. Concentrated organic part was purified by column chromatography. Pale yellow crystalline compound was isolated in 75% yields using ethyl acetate and pet ether mixture (20:80) as an eluent.

Step 2: Preparation of Benzyldiisopropylsilane

In a clean, oven-dried screw cap reaction tube, charged with magnetic stir–bar, activated magnesium turnings (15 mmol, 3 equiv.) and I_2 (one bead) were taken. The reaction tube was evacuated and back filled with nitrogen three times. Dry THF (15 mL) was added to it followed by di-isopropylchlorosilane (6 mmol, 1.2 equiv) in drop wise fashion and stirred at room temperature for 15 mins. A solution of benzyl chloride/bromide (5 mmol) in dry THF (10 mL) was added to the solution drop wise over a period of 15 minutes under ice cold condition. The mixture was vigorously stirred for 3 hours. Upon completion, the reaction mixture was quenched and washed with brine solution (3X10 mL). Aqueous part was washed thrice with ethyl acetate (3X20 mL). The combined organic layer was then dried over anhydrous Na_2SO_4. The crude mixture was purified by flash column chromatography using silica gel (60-120/100-200 mesh size) and petroleum-ether as the eluent. Benzyldiisopropylsilane was collected and used for next step.

Step 3: To an ice cold suspension of *N*-bromosuccinimide (5.0 mmol, 1.0 equiv) in 10 mL dry DCM, benzyldiisopropylsilane (**F**) (5.0 mmol, 1.0 equiv) was added drop wise under N_2 atmosphere. The reaction was kept on stirring for 3 hours at room temperature. In another clean round bottomed flask, charged with magnetic stir–bar, 4'-hydroxy-4, 5-dimethoxybiphenyl-2-carbonitrile (**D**) (5 mmol, 1.0 equiv) and 4-dimethylaminopyridine (10 mol%) were taken. The set up was evacuated and refilled with N_2. 5 mL dry DCM was added to the mixture followed by triethylamine (15 mmol, 3.0 equiv) in a drop wise fashion. The entire solution was kept for stirring at room temperature until 4'-hydroxy-4,5-dimethoxybiphenyl-2-carbonitrile gets dissolved completely. The aforementioned solution of benzylbromodiisopropylsilane was added drop wise under the ice-cold condition. The reaction mixture was then stirred overnight at room temperature. Upon completion, the mixture was quenched with water (20 mL) and extracted with ethyl acetate thrice (3X30 mL). The organic layer was combined and dried over anhydrous Na_2SO_4. The final substrate (**H**) was purified through column chromatography using silica gel (60-120/100-200 mesh size) and petroleum-ether/ethyl acetate (90/10, v/v) as the eluent. Isolated compound turned white solid upon drying. Yield: 73%

All the benzylsilane ethers were synthesized following the above procedures and characterized by ^{1}H and ^{13}C NMR spectroscopy, matched with our previous reports.[2-4]

2.3.2. General Procedure for Synthesis of Sugar derived aryl iodide:

To a stirred solution of peracetylated sugar (6 mmol, 1 equiv.) in CH_2Cl_2 (15 mL), iodophenol (7.2 mmol, 1.2 equiv.) and $BF_3{\cdot}OEt_2$ (12 mmol, 2 equiv.) were added at 0 ºC. The reaction mixture was stirred at RT for 10 h. After completion of reaction, the reaction mixture was quenched with aq. $NaHCO_3$ and dissolved in CH_2Cl_2. Separated organic layer was dried over Na_2SO_4, concentrated under reduced pressure and purified by column-chromatography to obtain the desired aryl iodide containing sugar compound.

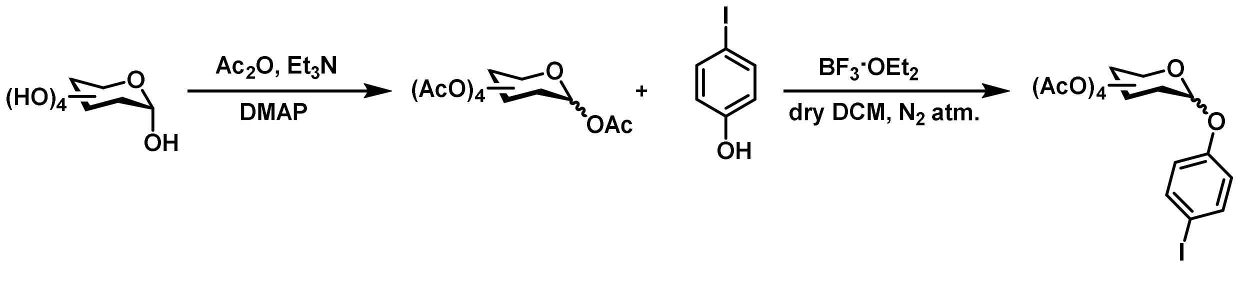

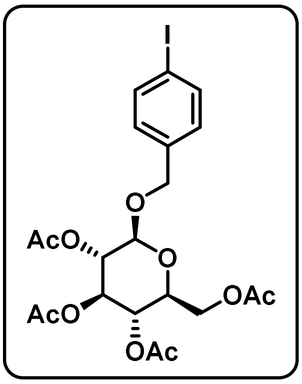

(2S,3S,4R,5S,6S)-2-(acetoxymethyl)-6-((4-iodobenzyl)oxy)tetrahydro-2H-pyran-3,4,5-triyl triacetate:

Eluent: petroleum ether/ethyl acetate (90:10, v/v).

Physical State: White solid

Isolated Yield: 80%

^{1}H NMR (400 MHz, $CDCl_3$) δ 7.67 (d, J = 8.4 Hz, 2H), 7.03 (d, J = 8.4 Hz, 2H), 5.22 – 5.02 (m, 3H), 4.83 (d, J = 12.5 Hz, 1H), 4.59 – 4.50 (m, J = 10.2, 6.1 Hz, 2H), 4.30 – 4.24 (m, J = 12.3, 4.7 Hz, 1H), 4.19 – 4.12 (m, J = 12.3, 2.4 Hz, 1H), 3.71 – 3.64 (m, J = 9.8, 4.7, 2.5 Hz, 1H), 2.10 (s, 3H), 2.02 (s, 3H), 2.01 (s, 3H), 2.00 (s, 3H).

^{13}C NMR (101 MHz, $CDCl_3$) δ 170.87, 170.47, 169.60, 169.48, 137.77, 136.59, 129.68, 99.66, 93.73, 72.95, 72.10, 71.43, 70.27, 68.54, 62.08, 20.96, 20.86, 20.80.

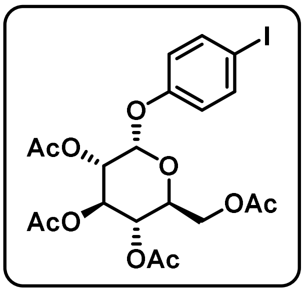

(2S,3S,4R,5S,6S)-2-(acetoxymethyl)-6-(4-iodophenoxy)tetrahydro-2H-pyran-3,4,5-triyl triacetate:

Eluent: petroleum ether/ethyl acetate (90:10, v/v).

Physical State: White solid

Isolated Yield: 80%

^{1}H NMR (400 MHz, $CDCl_3$) δ 7.56 (d, J = 8.7 Hz, 2H), 6.84 (d, J = 8.7 Hz, 2H), 5.71 – 5.58 (m, J = 17.3, 6.8 Hz, 2H), 5.11 (t, J = 9.9 Hz, 1H), 5.00 (dd, J = 10.2, 3.6 Hz, 1H),

4.20 (dd, J = 12.5, 4.7 Hz, 1H), 4.10 – 3.96 (m, 2H), 2.03 (s, 3H), 2.02 (s, 3H), 2.00 (s, 6H).
^{13}C NMR (101 MHz, $CDCl_3$) δ 170.58, 170.24, 170.21, 169.66, 155.98, 138.64, 118.95, 94.28, 85.93, 70.41, 70.00, 68.27, 61.62, 20.80, 20.76, 20.72, 20.69.

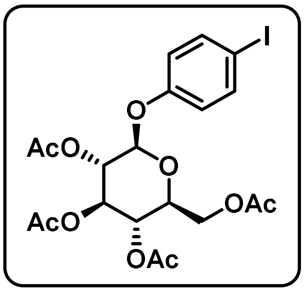

(2S,3S,4R,5S,6R)-2-(acetoxymethyl)-6-(4-iodophenoxy)tetrahydro-2H-pyran-3,4,5-triyl triacetate:
Eluent: petroleum ether/ethyl acetate (90:10, v/v).
Physical State: White solid
Isolated Yield: 80%
^{1}H NMR (400 MHz, $CDCl_3$) δ 7.58 (d, J = 9.0 Hz, 2H), 6.76 (d, J = 9.0 Hz, 2H), 5.34 – 5.21 (m, 2H), 5.15 (t, 1H), 5.03 (d, J = 7.5 Hz, 1H), 4.27 (dd, J = 12.3, 5.4 Hz, 1H), 4.16 (dd, J = 12.3, 2.4 Hz, 1H), 3.88 – 3.79 (m, J = 10.0, 5.4, 2.5 Hz, 1H), 2.07 (s, 3H), 2.05 (s, 3H), 2.04 (s, 3H), 2.03 (s, 3H).
^{13}C NMR (101 MHz, $CDCl_3$) δ 170.74, 170.43, 169.59, 169.46, 156.86, 138.70, 119.47, 99.16, 86.43, 72.82, 72.34, 71.30, 68.39, 62.09, 20.90, 20.82.

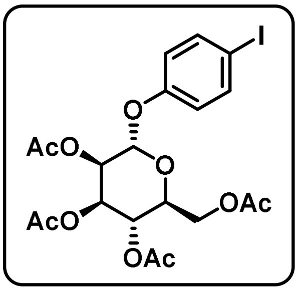

(2S,3S,4R,5R,6R)-2-(acetoxymethyl)-6-(4-iodophenoxy) tetrahydro-2H-pyran-3,4,5-triyl triacetate:
Eluent: petroleum ether/ethyl acetate (90:10, v/v).
Physical State: White solid
Isolated Yield: 80%
^{1}H NMR (400 MHz, $CDCl_3$) δ 7.58 (d, *J* = 8.3 Hz, 2H), 6.86 (d, *J* = 8.2 Hz, 2H), 5.51 (dd, *J* = 10.6, 4.0 Hz, 1H), 5.47 (s, 1H), 5.42 – 5.40 (m, 1H), 5.34 (t, *J* = 10.1 Hz, 1H), 4.25 (dd, *J* = 12.2, 6.1 Hz, 1H), 4.08 – 4.00 (m, 2H), 2.18 (s, 3H), 2.04 (s, 3H), 2.02 (s, 6H).

^{13}C NMR (101 MHz, $CDCl_3$) δ 170.64, 170.11, 170.09, 169.87, 155.58, 138.68, 118.96, 95.92, 86.00, 69.49, 69.40, 68.92, 66.01, 62.24, 21.02, 20.85, 20.83.

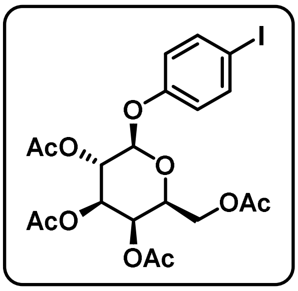

(2S,3R,4R,5S,6R)-2-(acetoxymethyl)-6-(4-iodophenoxy) tetrahydro-2H-pyran-3,4,5-triyl triacetate:
Eluent: petroleum ether/ethyl acetate (90:10, v/v).
Physical State: White solid
Isolated Yield: 85%
^{1}H NMR (400 MHz, $CDCl_3$) δ 7.59 (d, J = 8.9 Hz, 2H), 6.84 (d, J = 9.1 Hz, 2H), 5.73 (d, J = 3.7 Hz, 1H), 5.59 – 5.46 (m, 2H), 5.26 (dd, J = 10.6, 3.6 Hz, 1H), 4.27 (t, J = 6.1 Hz, 1H), 4.13 – 4.07 (m, 1H), 4.06 – 4.01 (m, 1H), 2.16 (s, 3H), 2.06 (s, 3H), 2.02 (s, 3H), 1.94 (s, 3H).
^{13}C NMR (101 MHz, $CDCl_3$) δ 170.56, 170.49, 170.34, 170.22, 156.30, 138.71, 119.22, 95.05, 85.94, 67.98, 67.86, 67.60, 67.52, 61.64, 20.91, 20.85, 20.81, 20.77.

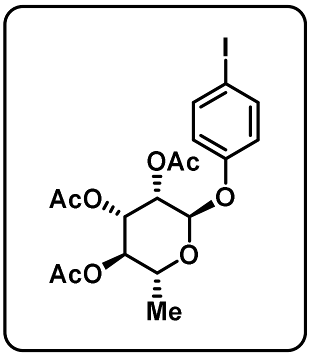

(2R,3S,4S,5R,6R)-2-(4-iodophenoxy)-6-methyltetrahydro-2H-pyran-3,4,5-triyl triacetate:
Eluent: petroleum ether/ethyl acetate (90:10, v/v).
Physical State: Crystalline white
Isolated Yield: 75%
^{1}H NMR (400 MHz, $CDCl_3$) δ 7.58 (d, J = 8.8 Hz, 2H), 6.84 (d, J = 8.8 Hz, 2H), 5.47 (dd, J = 10.1, 3.1 Hz, 1H), 5.40 (d, J = 3.4 Hz, 2H), 5.13 (t, J = 10.0 Hz, 1H), 3.99 – 3.86 (m, J = 12.5, 6.2 Hz, 1H), 2.18 (s, 3H), 2.05 (s, 3H), 2.02 (s, 3H), 1.18 (d, J = 6.2 Hz, 3H).

^{13}C NMR (101 MHz, $CDCl_3$) δ 170.22, 170.14, 155.81, 138.66, 118.78, 95.73, 85.60, 70.96, 69.69, 68.94, 67.46, 21.08, 20.97, 20.93, 17.60.

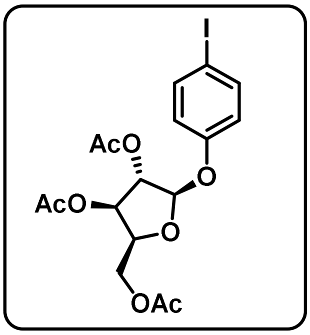

(2S,3R,4S,5R)-2-(acetoxymethyl)-5-(4-iodophenoxy)tetrahydrofuran-3,4-diyl diacetate (major isomer):

Eluent: petroleum ether/ethyl acetate (90:10, v/v).

Physical State: Sticky yellow liquid

Isolated Yield: 75%

^{1}H NMR (400 MHz, $CDCl_3$) δ 7.57 (d, J = 8.8 Hz, 2H), 6.84 (d, J = 8.9 Hz, 2H), 5.70 – 5.61 (m, 2H), 5.08 – 4.99 (m, 1H), 4.95 (dd, J = 10.2, 3.6 Hz, 1H), 3.84 (dd, J = 11.0, 6.0 Hz, 1H), 3.65 (t, J = 11.0 Hz, 1H), 2.06 (s, 3H), 2.04 (s, 3H), 2.03 (s, 3H).

^{13}C NMR (101 MHz, $CDCl_3$) δ 170.38, 170.27, 170.08, 156.16, 138.66, 118.98, 94.40, 85.75, 70.71, 69.49, 69.18, 59.22, 20.95, 20.86, 20.82.

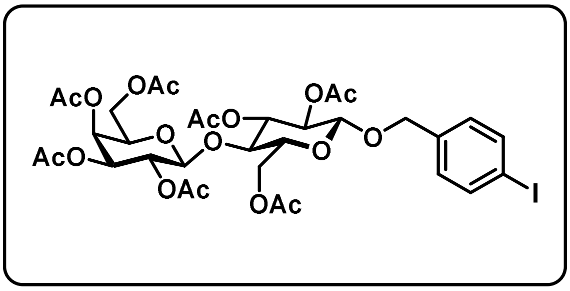

(2R,3S,4S,5R,6S)-2-(acetoxymethyl)-6-(((2R,3R,4S,5R,6R)-4,5-diacetoxy-2-(acetoxymethyl)-6-((4-iodobenzyl)oxy)tetrahydro-2H-pyran-3-yl)oxy)tetrahydro-2H-pyran-3,4,5-triyl triacetate:

Eluent: petroleum ether/ethyl acetate (70:30, v/v).

Physical State: White solid

Isolated Yield: 80%

^{1}H NMR (400 MHz, $CDCl_3$) δ 7.66 (d, J = 8.4 Hz, 2H), 7.01 (d, J = 8.3 Hz, 2H), 5.34 (d, J = 4.7 Hz, 1H), 5.16 (t, J = 9.3 Hz, 1H), 5.12 – 5.07 (m, 1H), 4.98 – 4.93 (m, 2H), 4.79 (d, J = 12.2 Hz, 1H), 4.54 – 4.52 (m, 1H), 4.51 (d, J = 2.4 Hz, 1H), 4.49 (d, J = 2.8 Hz, 1H), 4.48 (t, 1H), 4.13 – 4.06 (m, 3H), 3.86 (t, J = 6.8 Hz, 1H), 3.81 (t, 1H), 3.60 – 3.55 (m, 1H), 2.14 (s, 3H), 2.13 (s, 3H), 2.05 (s, 3H), 2.04 (s, 6H), 2.01 (s, 3H), 1.96 (s, 3H).

^{13}C NMR (101 MHz, $CDCl_3$) δ 170.48, 170.27, 170.18, 169.91, 169.69, 169.20, 137.68, 137.63, 136.51, 129.74, 129.58, 101.10, 99.33, 93.61, 76.24, 72.81, 71.68, 71.05, 70.76, 70.18, 69.20, 66.72, 62.01, 60.92, 20.98, 20.89, 20.79, 20.73, 20.61.

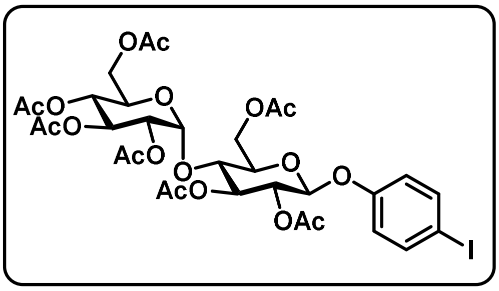

(2R,3R,4S,5R,6S)-2-(acetoxymethyl)-6-(((1R,2S,3S,4R,6R)-2,3-diacetoxy-6-(acetoxymethyl)-4-(4-iodophenoxy)cyclohexyl)oxy)tetrahydro-2H-pyran-3,4,5-triyl triacetate:

Eluent: petroleum ether/ethyl acetate (70:30, v/v).

Physical State: White solid

Isolated Yield: 82%

^{1}H NMR (400 MHz, $CDCl_3$) δ 7.57 (d, J = 8.9 Hz, 2H), 6.74 (d, J = 8.9 Hz, 2H), 5.42 (d, J = 4.0 Hz, 1H), 5.36 (d, J = 9.3 Hz, 1H), 5.33 – 5.26 (m, 1H), 5.08 – 5.05 (m, 2H), 5.02 (d, J = 9.6 Hz, 1H), 4.85 (dd, J = 10.6, 4.0 Hz, 1H), 4.46 (dd, J = 12.1, 2.8 Hz, 1H), 4.23 (dd, J = 12.3, 4.7 Hz, 2H), 4.07 (d, J = 3.7 Hz, 1H), 4.04 (dd, J = 10.0, 2.5 Hz, 1H), 3.98 – 3.93 (m, 1H), 3.87 – 3.82 (m, 1H), 2.09 (s, 3H), 2.07 (s, 3H), 2.03 (s, 3H), 2.03 (s, 3H), 2.02 (s, 3H), 2.02 (s, 3H), 1.99 (s, 3H).

^{13}C NMR (101 MHz, $CDCl_3$) δ 170.71, 170.53, 170.37, 170.14, 169.75, 169.61, 156.73, 138.67, 119.44, 98.49, 95.85, 86.34, 75.40, 72.82, 72.56, 72.10, 70.21, 69.46, 68.81, 68.21, 62.93, 61.76, 21.08, 20.90, 20.85, 20.79, 20.76, 20.74.

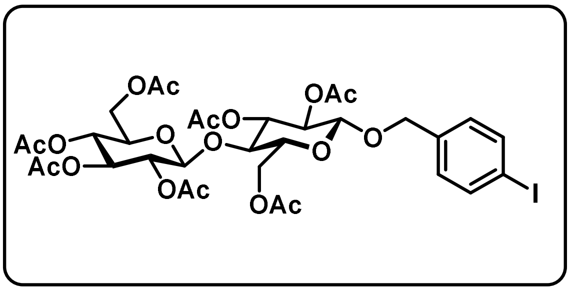

(2R,3R,4S,5R,6S)-2-(acetoxymethyl)-6-(((2R,3R,4S,5R,6R)-4,5-diacetoxy-2-(acetoxymethyl)-6-((4-iodobenzyl)oxy)tetrahydro-2H-pyran-3-yl)oxy)tetrahydro-2H-pyran-3,4,5-triyl triacetate:

Eluent: petroleum ether/ethyl acetate (70:30, v/v).

Physical State: White solid

Isolated Yield: 76%

^{1}H NMR (400 MHz, $CDCl_3$) δ 7.65 (d, J = 8.3 Hz, 2H), 7.00 (d, J = 8.3 Hz, 2H), 5.12 (t, J = 9.3 Hz, 2H), 5.04 (t, J = 9.6 Hz, 1H), 4.98 – 4.86 (m, 2H), 4.77 (d, J = 12.5 Hz, 1H), 4.55 – 4.46 (m, 4H), 4.35 (dd, J = 12.5, 4.4 Hz, 1H), 4.08 (dd, J = 12.0, 4.9 Hz, 1H), 4.02 (d, J = 12.4 Hz, 1H), 3.78 (t, J = 9.5 Hz, 1H), 3.67 – 3.61 (m, 1H), 3.59 – 3.52 (m, 1H), 2.12 (s, 3H), 2.06 (s, 3H), 2.01 (s, 3H), 1.99 (s, 9H), 1.96 (s, 3H).
^{13}C NMR (101 MHz, $CDCl_3$) δ 170.69, 170.49, 170.42, 169.99, 169.73, 169.50, 169.22, 137.72, 136.56, 129.64, 100.93, 99.49, 93.70, 76.54, 73.08, 72.94, 72.58, 72.13, 71.77, 71.65, 70.27, 67.93, 61.92, 61.70, 21.06, 20.85, 20.83, 20.72.

2.3.3. Synthesis of (2R,3R,4S,5R,6R)-4-(4-Iodo-2-(methoxymethyl)phenoxy)-2,3,5-trimethoxy-6-(methoxymethyl)tetrahydro-2H-pyran:

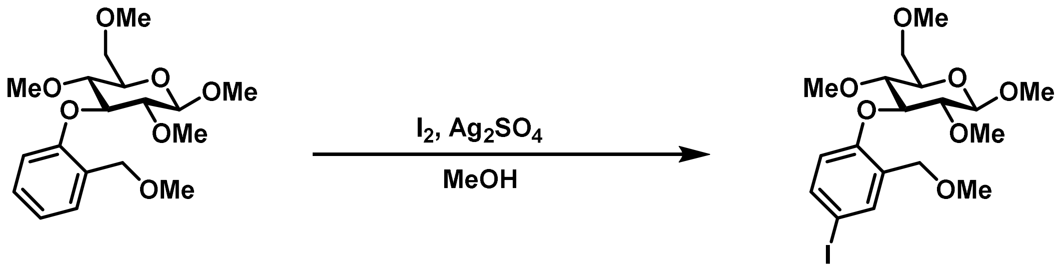

According to a literature procedure,[5] a suspension of (2R,3R,4S,5R,6R)-2,3,5- trimethoxy-6-(methoxymethyl)-4-(2-(methoxymethyl)phenoxy)tetrahydro-2H-pyran (356 mg, 1.0 mmol), iodine (330 mg, 1.05 mmol) and silver sulfate (328 mg, 1.05 mmol) in methanol was stirred at rt for 1 h and then the solid filtrated off. The filtrate was treated with saturated aqueous sodium sulfite solution until the violet color disappeared and then concentrated under reduced pressure. The resulting residue was extracted with dichloromethane (20 mL) and the organic phase washed with water (2 × 10 mL) and brine (10 mL), and dried over sodium sulfate. Upon removal of the solvent under reduced pressure, compound S25 was obtained as a colorless solid (429 mg, 89%).

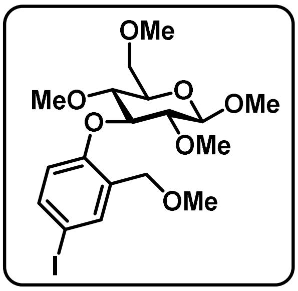

(2R,3R,4S,5R,6R)-4-(4-iodo-2-(methoxymethyl)phenoxy)-2,3,5-trimethoxy-6-(methoxymethyl)tetrahydro-2H-pyran:

Eluent: petroleum ether/ethyl acetate (80:20, v/v).
Physical State: Colourless solid
Isolated Yield: 89%
^{1}H NMR (400 MHz, $CDCl_3$) δ 7.68 (s, 1H), 7.50 (d, J = 8.7 Hz, 1H), 6.77 (d, J = 8.7 Hz, 1H), 4.75 (d, J = 6.8 Hz, 1H), 4.48 (q, J = 13.1 Hz, 2H), 3.65 (s, 3H), 3.63 (s, 3H), 3.61 (d, J = 2.1 Hz, 1H), 3.56 (d, J = 4.6 Hz, 1H), 3.54 (s, 3H), 3.40 (s, 3H), 3.37 (s, 3H), 3.30 – 3.19 (m, 3H).
^{13}C NMR (101 MHz, $CDCl_3$) δ 154.61, 137.39, 137.05, 130.70, 117.33, 101.27, 86.72, 85.79, 83.63, 79.23, 75.03, 71.20, 68.70, 61.17, 60.98, 60.66, 59.58, 58.73.

2.4. General procedure for palladium catalyzed *para*-selective C−H arylation of arene:

In an oven-dried screw cap reaction tube charged with a magnetic stir-bar was added $Pd(OAc)_2$ (10 mol%), Fmoc-Gly-OH (20 mol%), Ag_2SO_4 (2 equiv.), $Cu_2Cr_2O_5$ (2 equiv.) and $LiOAc.2H_2O$ (0.5 equiv.). After that benzylsilyl ether substrate (0.1 mmol, 1 equiv.) and aryl iodide (1 equiv.) was added. Subsequently, 1 mL of 1,1,1,3,3,3-Hexafluoro-2-propanol (HFIP) was added with a disposable laboratory syringe under aerobic condition.

The tube was placed in a preheated oil bath at 80 ºC and the reaction mixture was stirred for 24 h. The reaction mixture was then cooled to room temperature and filtered through a celite pad with ethyl acetate. The filtrate was concentrated *in vacuo* and the resulting residue was purified by silica gel (100-200 mesh size) column chromatography to give the desired product.

2.4.1. General procedure for directing group removal of *para*-arylated protocol

Method 1: In a clean, oven-dried screw cap reaction tube with previously placed magnetic stir–bar, *para*-arylated compound (0.1 mmol) was dissolved in 3 mL of THF, a solution of 1M TBAF (1.0 mL, 2.0 eq.) in THF was added drop wise at RT. The solution was stirred for 3 hours at room temperature. After completion of reaction, solvent was evaporated to dryness, and the residue was purified by chromatography using silica gel.

Method 2: In a clean, oven-dried screw cap reaction tube with previously placed magnetic stir–bar, *para*-arylated compound (0.1 mmol) and *p*-toluenesulfonic acid (10 mol%) were dissolved in 3 mL of EtOH and 1 mL H_2O (EtOH/H_2O: 3/1). The solution was stirred at 110 °C for 16 hours. After being stirred, reaction mixture was removed from oil-bath and kept at room temperature. Ethanol was removed under reduced pressure and aqueous part was extracted by EtOAc. Organic part was evaporated to dryness and the residue was

purified by column chromatography silica gel.

2.4.2. General procedure for different application of *para*-arylated protocol

2.4.2.a. Procedure for the *ortho*-olefination of silanol derivative

According to a literature procedure,[6] a 20 mL oven-dried screw cap reaction tube was charged with *para*-arylated silanol derivative **6** (0.1 mmol), olefin (0.2 mmol), KH_2PO_4 (0.2 mmol), $Pd(OAc)_2$ (0.02 mmol, 20 mol%), AgOAc (0.2 mmol) and $CHCl_3$ (1.5 mL). The reaction tube was then sealed, heated to 100 °C, and stirred for 16 hours. The reaction mixture was cooled to room temperature, diluted with ethyl acetate (5 mL), filtered through a pad of Celite, and the filtrate was then concentrated under vacuo. The crude product was purified by flash chromatography on silica gel (gradient eluent of EtOAc in Hexanes) to yield the products 7 and 8.

2.4.2.b. Preparation of *para*-arylated benzyl alcohol

A screw cap reaction tube containing stirring bar was charged with *para*-arylated compound **3qz'** (0.1 mmol), KF (2 equiv.) and $KHCO_3$ (10 equiv.). THF (0.25 mL), MeOH (0.25 mL) and 30% H_2O_2 (150 μL) were added via syringes and the reaction mixture was heated at 60 °C for 24 h. After aqueous work up combined organic layer was concentrated and purified by column chromatography.

2.4.2.c. Preparation of *para*-arylated benzaldehyde

A screw cap reaction tube containing stirring bar was charged with *para*-arylated compound **3al** (0.1 mmol), CsF (2 equiv.) and PhNO (3 equiv.). Commercial DMF (1mL) was added via syringe and the reaction mixture was heated at 65 °C for 4 h. After aqueous work up combined organic layer was concentrated and purified by column chromatography

2.4.2.d. Nucleophilic addition of silyl motif to aldehyde

In a closed cap reaction tube, *para*-arylated product **3hm** (0.1 mmol, 1.0 equiv.) and corresponding aryl aldehyde **13** (0.12 mmol, 1.2 equiv.) were dissolved in THF (1 mL). To the reaction mixture TBAF (1 M solution in THF, 0.1 mmol, 1 equiv) was added. The reaction mixture was stirred at room temperature for 12 h. The reaction mixture was then extracted with ethyl acetate (20 mL, then 2 x 10 mL) and dried over Na_2SO_4 and concentrated by rotary evaporation. The crude benzyl alcohol derivative **14** was further purified by column chromatography.

2.4.2.e. Synthesis of antifungal drug Bifonazole

As stated above the *para*-arylated product **3qz'** was treated with condition **2.4.2.b** to produce the corresponding benzyl alcohol. The benzhydrol (0.1 mmol) was then stirred with an excess of thionyl chloride (1 mL) at r.t. for about 6 h. The mixture was then evaporated under reduced pressure. The crude product was used without further purification.

Benzhydryl chloride derivative was dissolved in dioxane (1 mL) and added dropwise to a solution of imidazole (3 equiv.) in the same solvent (2 mL). The mixture was refluxed for 24 h, and the progress of the reaction was monitored by TLC. Then the mixture was evaporated to give a yellow oil which was dissolved in ethyl acetate (10 mL), washed with water (3×50 mL), dried over Na_2SO_4 and evaporated. The crude product was purified by column chromatography on silica gel using PE/EA as eluents.

2.5. Determination of *para*-selectivity

The selectivity of *para*-C–H arylation product was determined by ^{1}H NMR of crude reaction mixture. Singlet of benzylic proton in ^{1}H NMR was used to measure the selectivity.

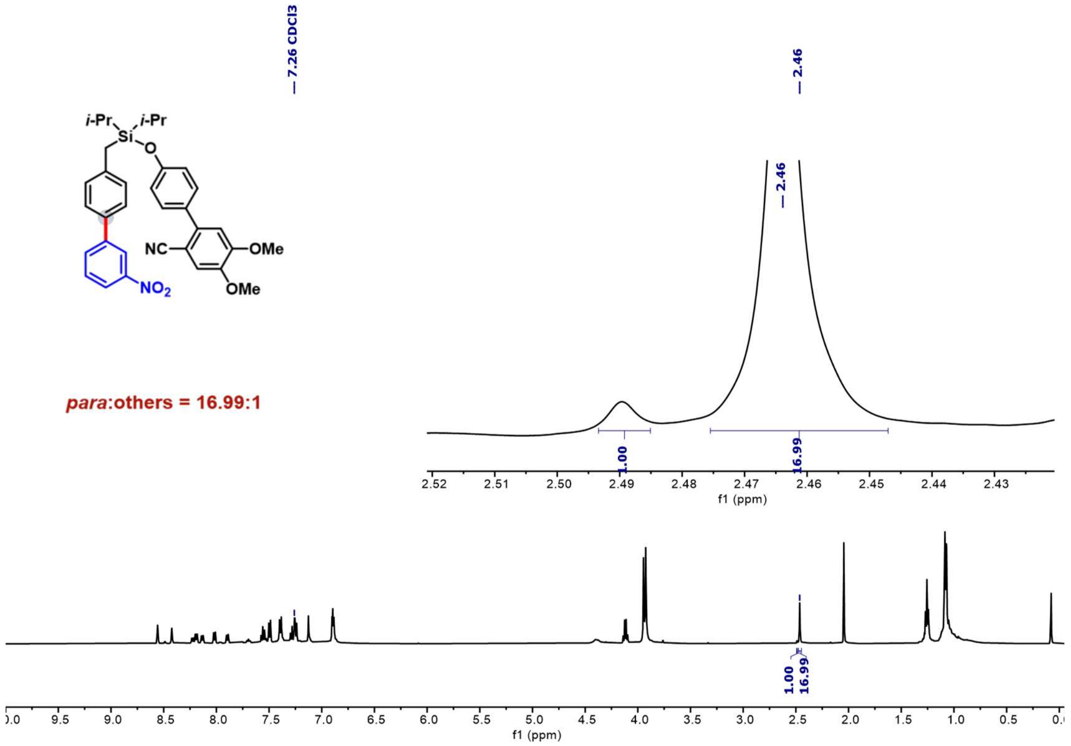

Supplementary Figure 1. ^{1}H NMR of crude reaction mixture of **3aa** in $CDCl_3$

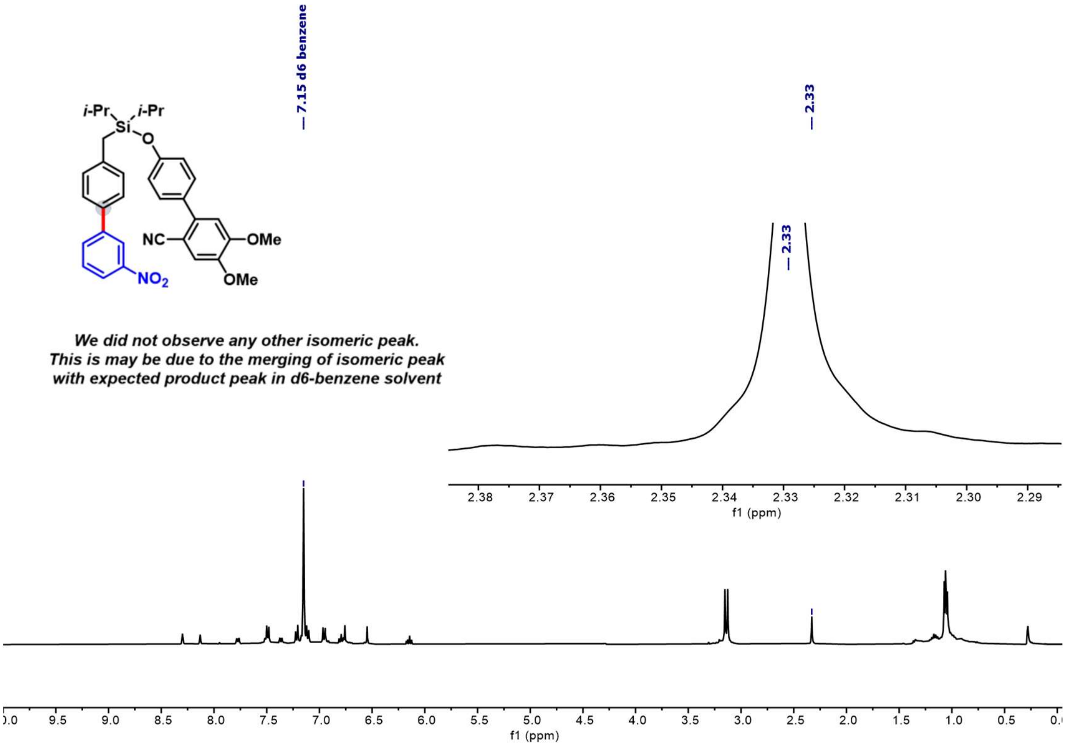

Supplementary Figure 2. ^{1}H NMR of crude reaction mixture of **3aa** in C_6D_6

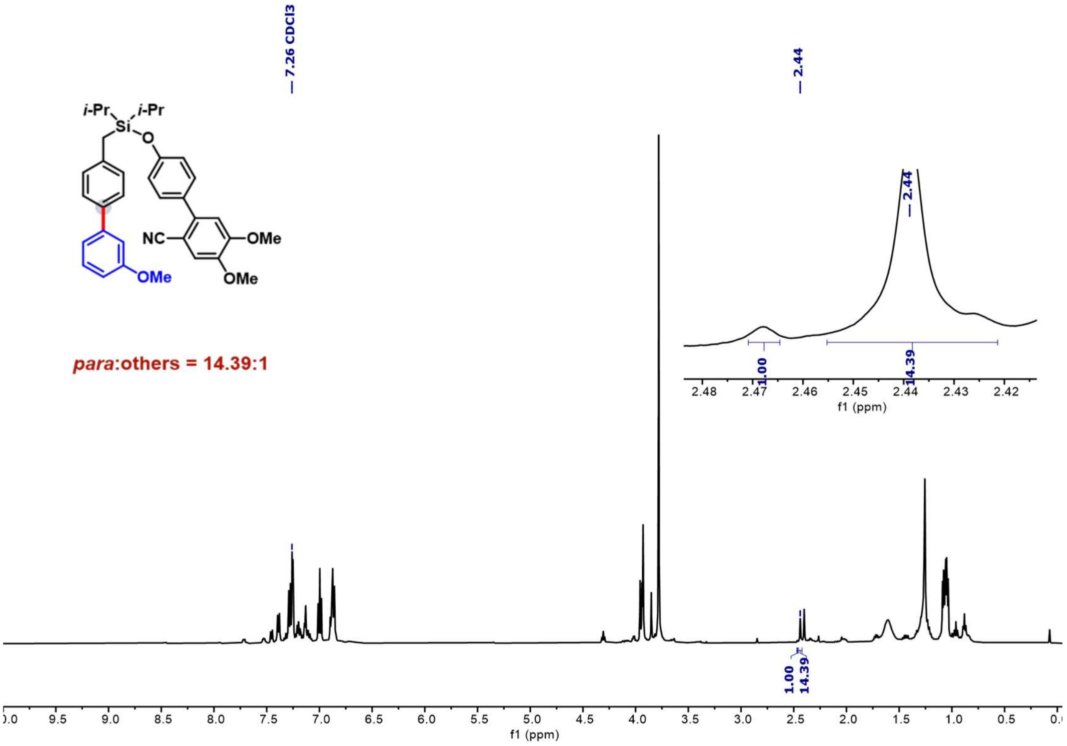

Supplementary Figure 3. ^{1}H NMR of crude reaction mixture of **3ac** in $CDCl_3$

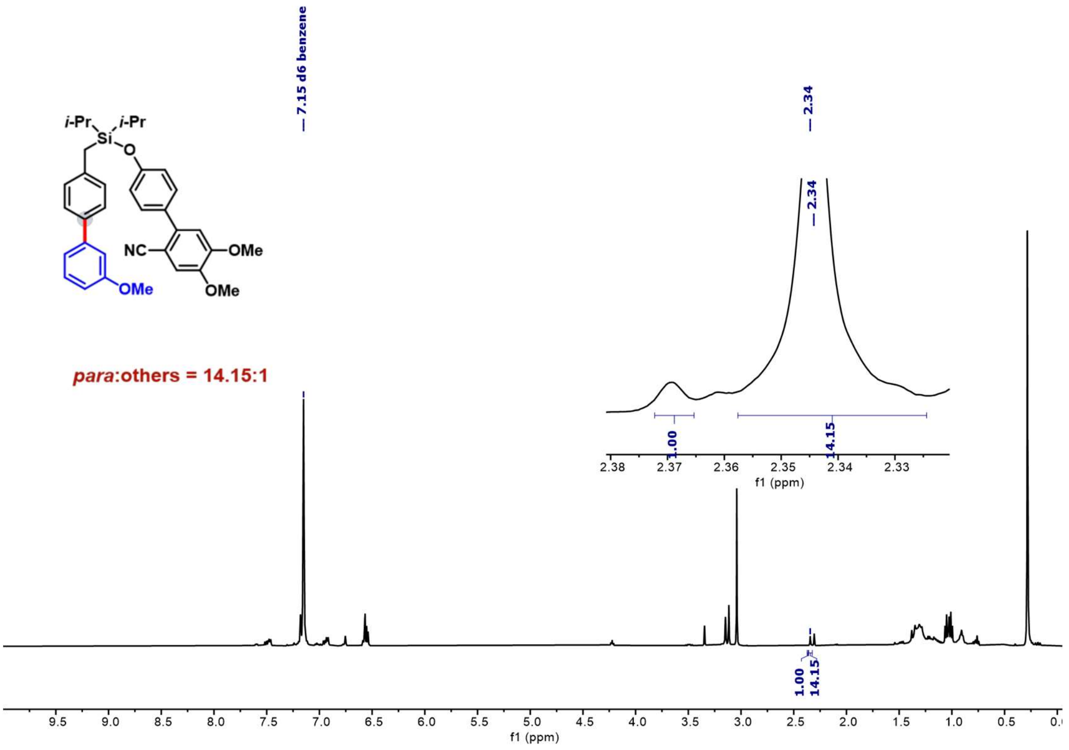

Supplementary Figure 4. ^{1}H NMR of crude reaction mixture of **3ac** in C_6D_6

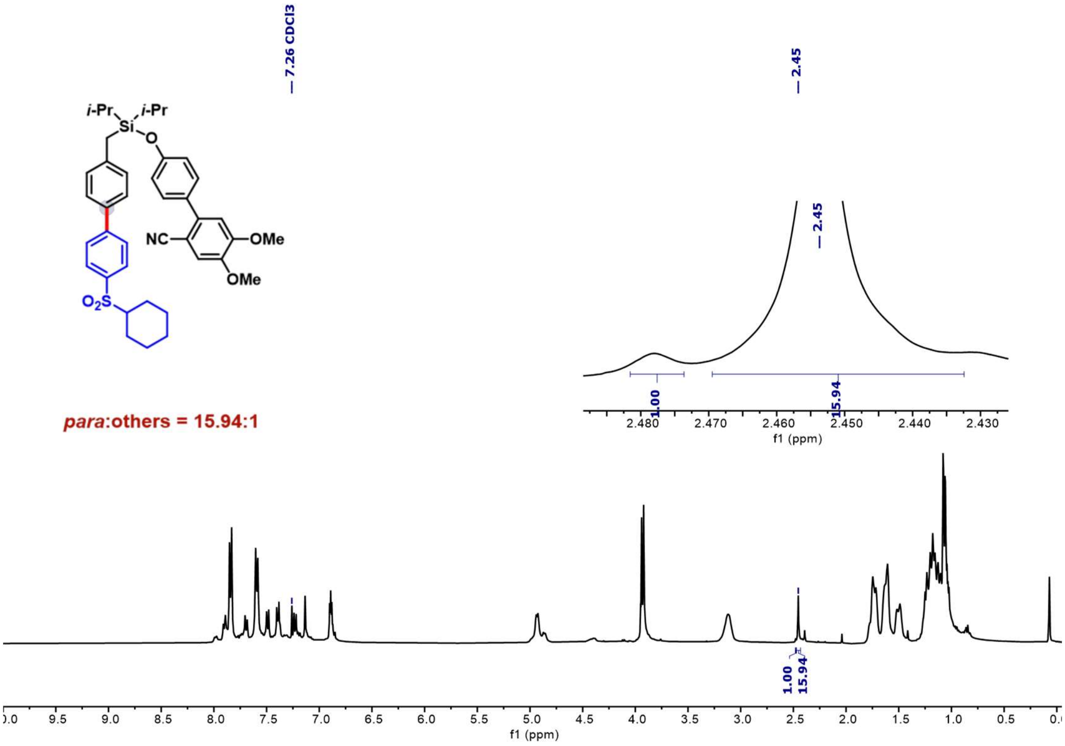

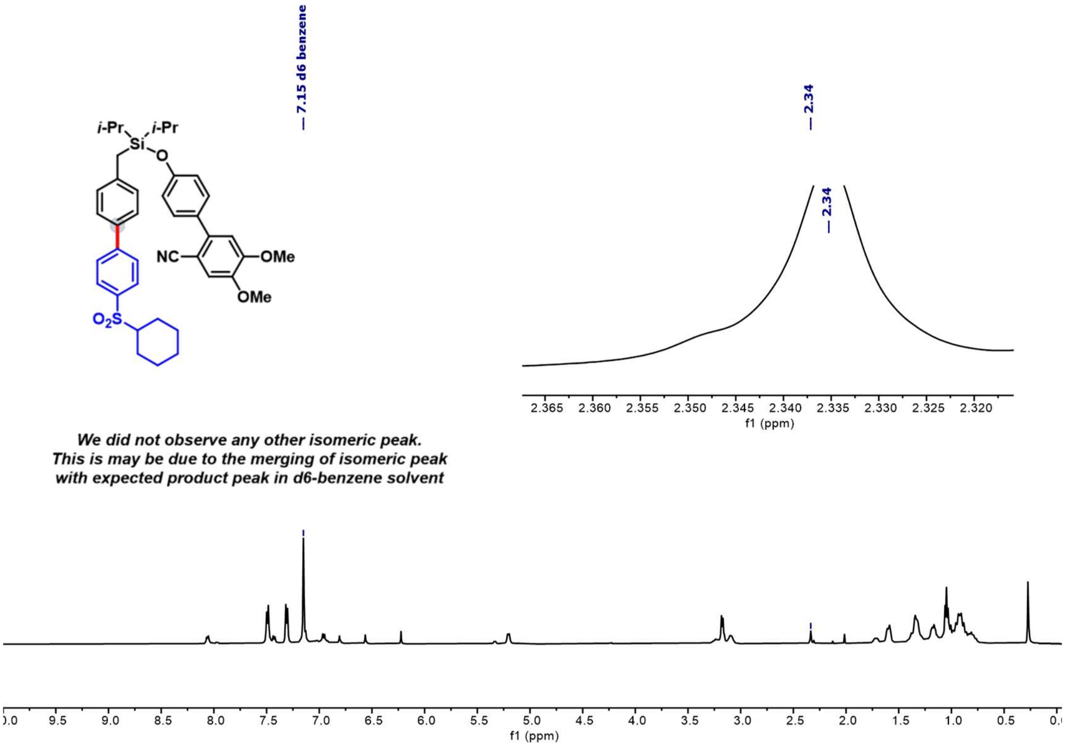

Supplementary Figure 5. ^{1}H NMR of crude reaction mixture of **3aj** in $CDCl_3$ (top) and C_6D_6 (bottom)

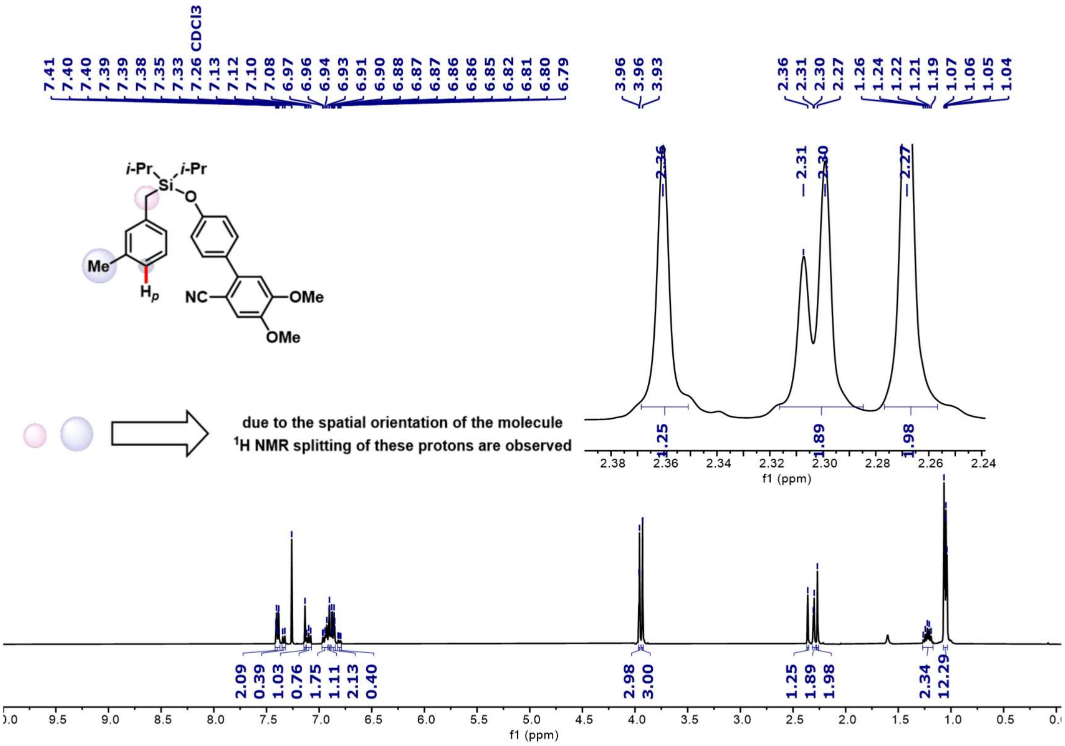

Supplementary Figure 6. ^{1}H NMR of substrate **3g**

^{1}H NMR of isolated compound (3gv):

Peaks at 2.47 ppm, 2.41 ppm, and 2.32 ppm are from the expected compound **3gv**. Due to the spatial orientation of the molecule benzyl peaks are splitting further. These peaks are not from the any other isomers.

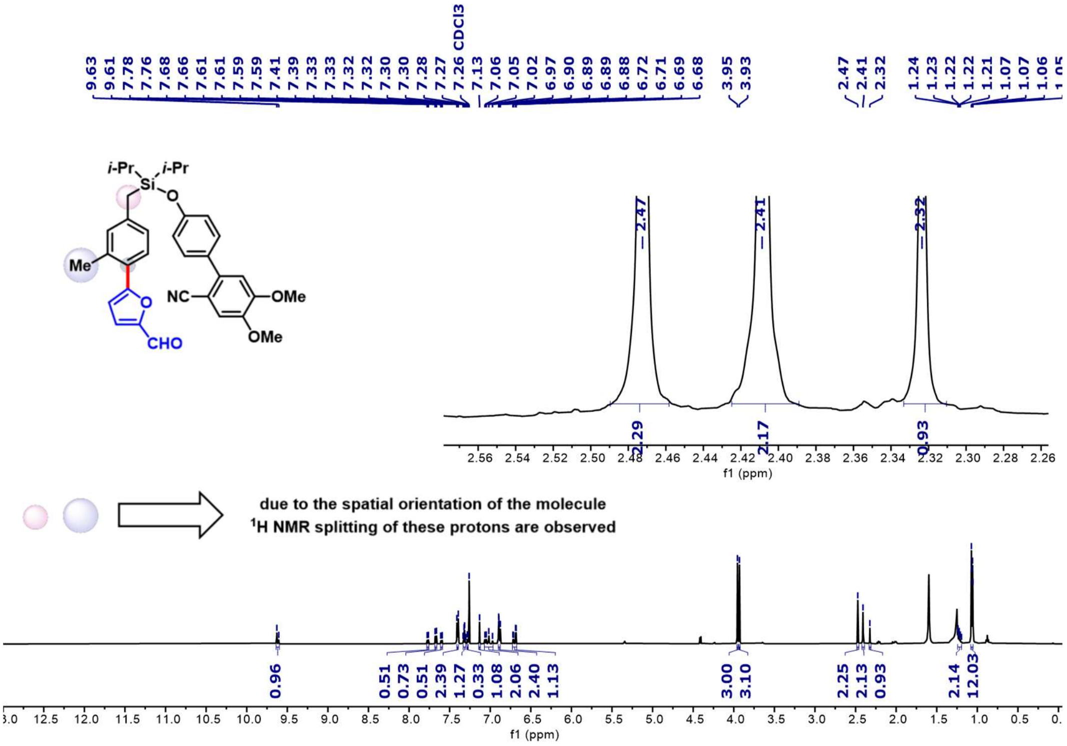

Supplementary Figure 7. ^{1}H NMR of isolated compound **3gv**

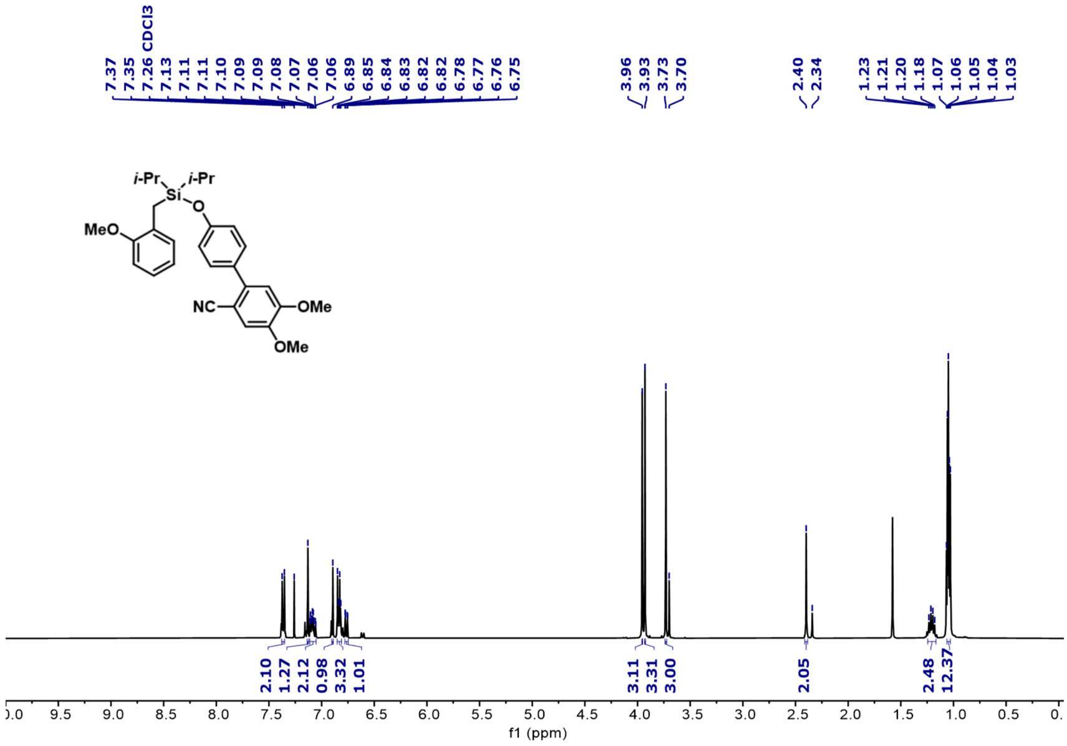

Supplementary Figure 8. ^{1}H NMR of substrate **1c**

^{1}H NMR of isolated compound (3cx):

Peaks at 2.43 ppm, 2.40 ppm, and 2.34 ppm are from the expected compound **3cx**. Due to the spatial orientation of the molecule benzyl peaks are splitting further. These peaks are not from any other isomers. In this case the polarity of the expected product **3cx** and starting material **1c** is similar. That's why there are some extra peaks in the ^{1}H NMR of isolated product.

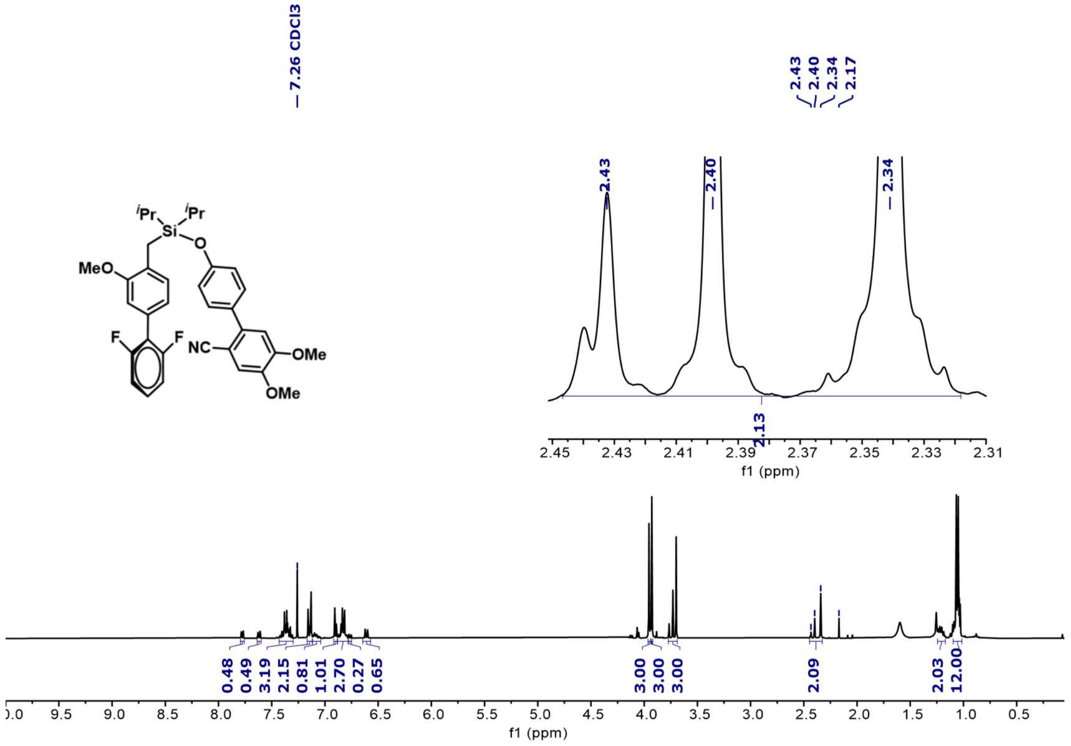

Supplementary Figure 9. ^{1}H NMR of isolated compound **3cx**

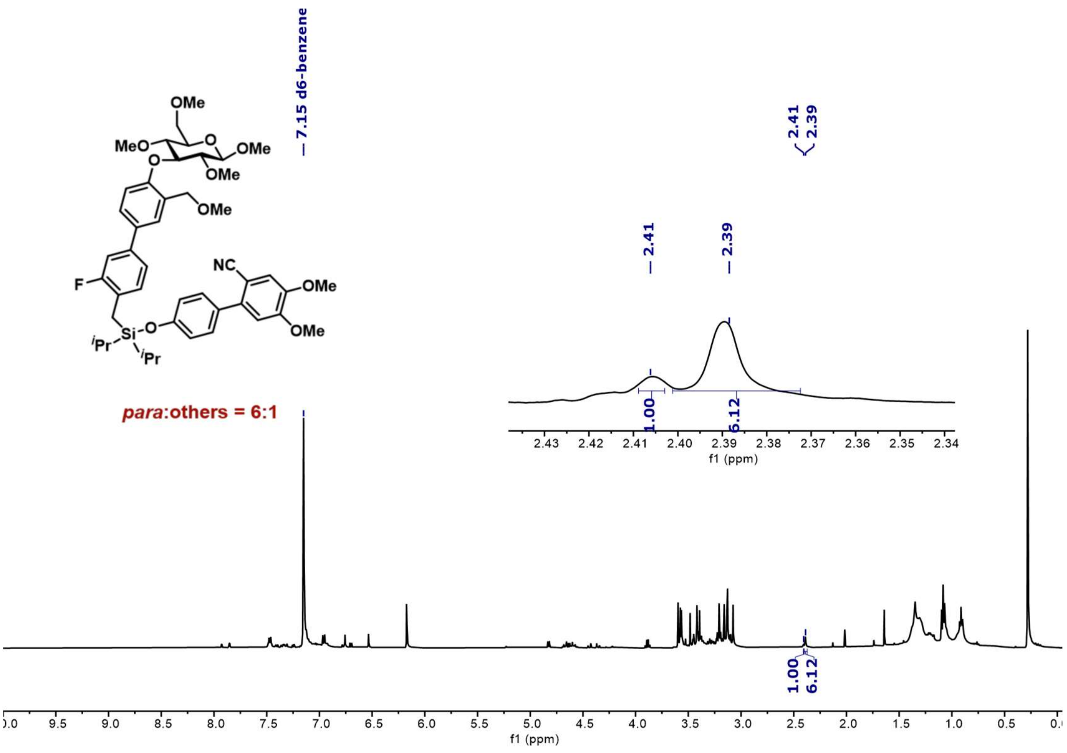

Supplementary Figure 10. ^{1}H NMR of crude reaction mixture of **5uk**

2.6. Characterization data of *para*-arylated products

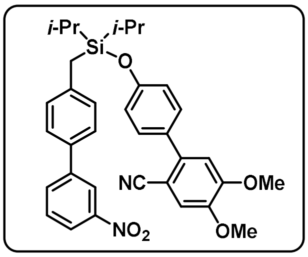

4'-((diisopropyl((3'-nitro-[1,1'-biphenyl]-4-yl)methyl)silyl)oxy)-4,5-dimethoxy-[1,1'-biphenyl]-2-carbonitrile (3aa): Compound 3aa was prepared under the General Procedure 2.4.

Eluent: petroleum ether/ethyl acetate (90:10, v/v).

Physical State: Sticky yellow liquid

Isolated Yield: 84% (48.7 mg); (*p*:others = 15:1).

R_f Value: 0.5 (20% ethyl acetate in petroleum ether).

^{1}H NMR (500 MHz, $CDCl_3$) δ 8.43 (t, J = 2.0 Hz, 1H), 8.13 (d, J = 10.8 Hz, 1H), 7.90 (d, J = 7.8 Hz, 1H), 7.56 (t, J = 7.9 Hz, 1H), 7.50 (d, J = 8.2 Hz, 2H), 7.40 (d, J = 8.5 Hz, 2H),

7.25 (d, J = 8.2 Hz, 2H), 7.13 (s, 1H), 6.91 – 6.85 (m, 3H), 3.95 (s, 3H), 3.92 (s, 3H), 2.46 (s, 2H), 1.30 – 1.23 (m, 2H), 1.08 (dd, J = 7.5, 2.4 Hz, 12H).
^{13}C NMR (126 MHz, $CDCl_3$) δ 156.08, 152.69, 148.87, 148.16, 142.88, 140.10, 139.64, 134.90, 132.85, 131.49, 130.07, 129.80, 129.77, 127.12, 121.73, 121.69, 120.15, 119.49, 115.15, 112.45, 102.28, 56.44, 56.31, 20.99, 17.65, 17.60, 13.13.
IR (thin film, cm^{-1}): 684.366, 754.894, 839.516, 915.704, 1029.429, 1173.504, 1215.557, 1266.285, 1349.978, 1462.717, 1502.955, 1603.933, 2220.334, 2867.192, 2929.604.
HRMS (ESI): Calculated for $C_{34}H_{37}N_2O_5Si$ [M+H$^+$]: 581.2466; found: 581.2463.

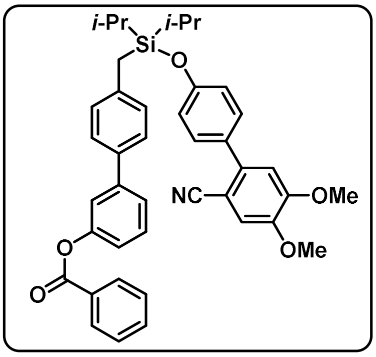

4'-((((2'-cyano-4',5'-dimethoxy-[1,1'-biphenyl]-4-yl)oxy)diisopropylsilyl)methyl)-[1,1'-biphenyl]-3-yl benzoate (3ab): Compound 3ab was prepared under the General Procedure 2.4.
Eluent: petroleum ether/ethyl acetate (90:10, v/v).
Physical State: yellowish liquid
Isolated Yield: 80% (52.46 mg); (*p*:others = 15:1).
R*f* Value: 0.5 (20% ethyl acetate in petroleum ether).
^{1}H NMR (500 MHz, $CDCl_3$) δ 8.24 – 8.21 (m, 2H), 7.65 (t, J = 7.4 Hz, 1H), 7.52 (t, J = 7.8 Hz, 2H), 7.49 – 7.45 (m, 4H), 7.44 (d, J = 2.1 Hz, 1H), 7.39 (d, J = 8.6 Hz, 2H), 7.20 (d, J = 8.2 Hz, 2H), 7.17 – 7.15 (m, 1H), 7.12 (s, 1H), 6.88 (d, J = 7.7 Hz, 3H), 3.93 (d, J = 8.0 Hz, 6H), 2.44 (s, 2H), 1.30 – 1.27 (m, 1H), 1.07 (dd, J = 7.4, 4.3 Hz, 12H).
^{13}C NMR (126 MHz, $CDCl_3$) δ 165.45, 156.20, 152.71, 151.56, 148.16, 142.99, 140.24, 138.50, 136.50, 133.81, 131.44, 130.40, 130.07, 129.89, 129.81, 129.53, 128.79, 127.21, 124.51, 120.29, 120.23, 119.53, 115.21, 112.54, 102.33, 56.48, 56.35, 20.91, 17.68, 17.64, 13.13.
IR (thin film, cm^{-1}): 708.281, 757.113, 839.636, 913.980, 1026.075, 1081.867, 1174.989, 1262.821, 1352.935, 1463.310, 1502.984, 1603.736, 1737.141, 2219.857, 2867.544, 2929.449.
HRMS (ESI): Calculated for $C_{41}H_{41}NO_5Si$ [M+H$^+$]: 656.2866; found: 656.2856.

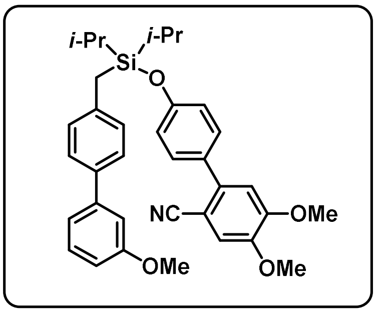

4'-((diisopropyl((3'-methoxy-[1,1'-biphenyl]-4-yl)methyl)silyl)oxy)-4,5-dimethoxy-[1,1'-biphenyl]-2-carbonitrile (3ac): Compound 3ac was prepared under the General Procedure 2.4.

Eluent: petroleum ether/ethyl acetate (95:5, v/v).

Physical State: Yellow Sticky liquid

Isolated Yield: 69% (39 mg); (*p*:others = 14:1).

R_f Value: 0.5 (20% ethyl acetate in petroleum ether).

^{1}H NMR (500 MHz, $CDCl_3$) δ 7.45 (d, J = 8.0 Hz, 2H), 7.39 (d, J = 8.4 Hz, 2H), 7.32 (t, J = 7.9 Hz, 1H), 7.21 – 7.16 (m, 3H), 7.13 (s, 1H), 7.11 (s, 1H), 6.89 – 6.84 (m, 4H), 3.94 (s, 3H), 3.93 (s, 3H), 3.85 (s, 3H), 2.44 (s, 2H), 1.30 – 1.26 (m, J = 7.4 Hz, 2H), 1.08 (d, J = 5.0 Hz, 6H), 1.07 (d, J = 5.1 Hz, 6H).

^{13}C NMR (126 MHz, $CDCl_3$) δ 160.13, 156.24, 152.73, 148.18, 142.80, 140.25, 138.13, 137.37, 131.43, 130.06, 129.87, 129.45, 127.19, 120.25, 119.56, 115.22, 112.68, 112.55, 102.35, 56.49, 56.34, 55.50, 20.88, 17.69, 17.65, 13.13.

IR (thin film, cm^{-1}): 758.952, 840.879, 914.516, 1038.716, 1175.877, 1175.877, 1267.433, 1353.329, 1462.805, 1502.374, 1604.559, 1723.678, 2219.833, 2867.370, 2943.897.

HRMS (ESI): Calculated for $C_{35}H_{39}NNaO_4Si$ [M+Na$^+$]: 588.2541; found: 585.2544.

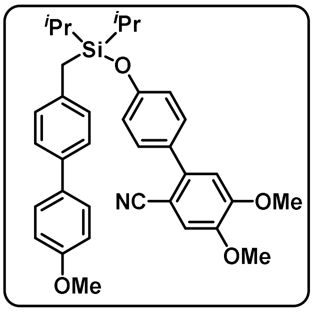

4'-((diisopropyl((4'-methoxy-[1,1'-biphenyl]-4-yl)methyl)silyl)oxy)-4,5-dimethoxy-[1,1'-biphenyl]-2-carbonitrile (3ad): Compound 3ad was prepared under the **General Procedure 2.4.**

Eluent: petroleum ether/ethyl acetate (95:5, v/v).

Physical State: yellow sticky liquid

Isolated Yield: 70% (39.6 mg); (*p*:others = 15:1).

R_f Value: 0.5 (20% ethyl acetate in petroleum ether).

^{1}H NMR (500 MHz, $CDCl_3$) δ 7.51 (d, J = 8.8 Hz, 2H), 7.41 (d, J = 8.2 Hz, 2H), 7.39 (d, J = 8.6 Hz, 2H), 7.17 (d, J = 8.2 Hz, 2H), 7.13 (s, 1H), 6.95 (d, J = 8.8 Hz, 2H), 6.89 (d, J = 3.1 Hz, 2H), 6.87 (s, 1H), 3.94 (s, 3H), 3.93 (s, 3H), 3.84 (s, 3H), 2.43 (s, 2H), 1.28 – 1.24 (m, J = 7.4 Hz, 2H), 1.08 (d, J = 5.3 Hz, 6H), 1.07 (d, J = 5.3 Hz, 6H).
^{13}C NMR (126 MHz, $CDCl_3$) δ 159.04, 156.26, 152.71, 148.16, 140.26, 137.17, 133.87, 131.39, 130.04, 129.44, 127.99, 126.74, 120.25, 119.53, 115.21, 114.33, 112.53, 102.34, 56.48, 56.34, 55.55, 20.76, 17.69, 17.65, 13.11.
IR (thin film, cm^{-1}): 758.952, 840.879, 914.516, 1038.716, 1175.877, 1175.877, 1267.433, 1353.329, 1462.805, 1502.374, 1604.559, 1723.678, 2219.833, 2867.370, 2943.897.
HRMS (ESI): Calculated for $C_{35}H_{40}NO_4Si$ [M+H$^+$]: 566.2721; found: 566.2722.

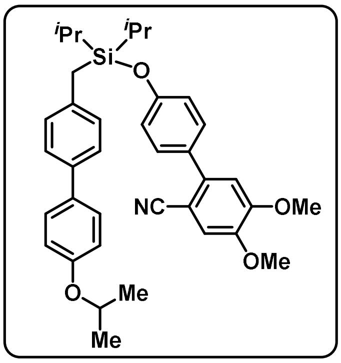

4'-((((4'-isopropoxy-[1,1'-biphenyl]-4-yl)methyl)diisopropylsilyl)oxy)-4,5-dimethoxy-[1,1'-biphenyl]-2-carbonitrile (3ae): Compound 3ae was prepared under the **General Procedure 2.4.**
Eluent: petroleum ether/ethyl acetate (95:5, v/v).
Physical State: yellow sticky liquid
Isolated Yield: 85% (50.5 mg); (*p*:others = 30:1).
R_f Value: 0.5 (15% ethyl acetate in petroleum ether).
^{1}H NMR (400 MHz, $CDCl_3$) δ 7.49 (d, J = 8.7 Hz, 2H), 7.41 (d, J = 8.2 Hz, 2H), 7.38 (d, J = 8.6 Hz, 2H), 7.17 (d, J = 8.1 Hz, 2H), 7.13 (s, 1H), 6.92 (d, J = 8.7 Hz, 2H), 6.89 – 6.87 (m, J = 4.2 Hz, 3H), 4.61 – 4.53 (m, J = 12.1, 6.1 Hz, 1H), 3.94 (s, 3H), 3.93 (s, 3H), 2.42 (s, 2H), 1.35 (d, J = 6.0 Hz, 6H), 1.28 – 1.23 (m, J = 7.5 Hz, 5H), 1.08 (d, J = 4.3 Hz, 6H), 1.06 (d, J = 4.3 Hz, 6H).
^{13}C NMR (101 MHz, $CDCl_3$) δ 157.34, 156.26, 152.70, 148.15, 140.26, 137.18, 137.08, 133.62, 131.38, 130.04, 129.42, 127.97, 126.70, 120.25, 119.54, 116.29, 115.20, 112.53, 102.32, 70.16, 56.48, 56.34, 22.30, 20.75, 17.68, 17.65, 13.10.
IR (thin film, cm^{-1}): 678.377, 758.595, 839.165, 914.964, 1029.756, 1121.157, 1266.512, 1372.106, 1463.233, 1502.179, 1604.670, 2220.093, 2867.649, 2932.671.
HRMS (ESI): Calculated for $C_{37}H_{43}NNaO_4Si$ [M+Na$^+$]: 616.2854; found: 616.2851.

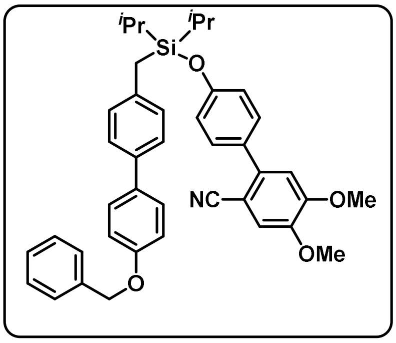

4'-((((4'-(benzyloxy)-[1,1'-biphenyl]-4-yl)methyl)diisopropylsilyl)oxy)-4,5-dimethoxy-[1,1'-biphenyl]-2-carbonitrile (3af): Compound 3af was prepared under the **General Procedure 2.4.**

Eluent: petroleum ether/ethyl acetate (90:10, v/v).

Physical State: Yellow sticky liquid

Isolated Yield: 82% (52.6 mg); (*p*:others = 20:1).

R*f* Value: 0.5 (20% ethyl acetate in petroleum ether).

^{1}H NMR (400 MHz, $CDCl_3$) δ 7.51 (d, J = 8.7 Hz, 2H), 7.45 (d, J = 7.2 Hz, 2H), 7.42 – 7.37 (m, 6H), 7.34 (d, J = 7.2 Hz, 1H), 7.17 (d, J = 8.2 Hz, 2H), 7.13 (s, 1H), 7.02 (d, J = 8.7 Hz, 2H), 6.90 – 6.87 (m, 3H), 5.10 (s, 2H), 3.94 (s, 3H), 3.93 (s, 3H), 2.42 (s, 2H), 1.30 – 1.21 (m, 5H), 1.08 (d, J = 4.2 Hz, 6H), 1.06 (d, J = 4.2 Hz, 6H).

^{13}C NMR (101 MHz, $CDCl_3$) δ 158.26, 156.26, 152.71, 148.16, 140.26, 137.22, 137.08, 134.11, 131.39, 130.05, 129.44, 128.81, 128.18, 128.01, 127.69, 126.74, 120.25, 119.54, 115.29, 115.20, 112.53, 102.33, 70.29, 56.48, 56.34, 20.76, 17.69, 17.65, 13.11.

IR (thin film, cm^{-1}): 755.512, 840.672, 913.593, 1029.500, 1174.936, 1174.936, 1241.862, 1266.064, 1352.823, 1461.758, 1501.214, 1603.923, 1738.526, 2219.266, 2866.967, 2928.469.

HRMS (ESI): Calculated for $C_{41}H_{43}NKO_4Si$ [M+K$^+$]: 680.2504; found: 680.2505.

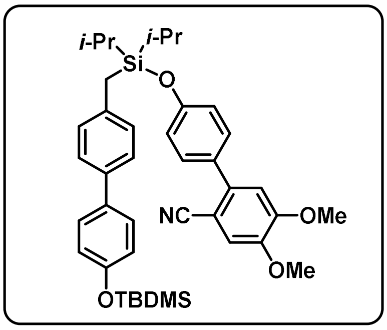

4'-((((4'-((tert-butyldimethylsilyl)oxy)-[1,1'-biphenyl]-4-yl)methyl)diisopropylsilyl)oxy)-4,5-dimethoxy-[1,1'-biphenyl]-2-carbonitrile (3ag): Compound 3ag was prepared under the **General Procedure 2.4.**

Eluent: petroleum ether/ethyl acetate (95:5, v/v).

Physical State: Yellow sticky liquid
Isolated Yield: 74% (49 mg); (*p*:others = 16:1).
R$_f$ Value: 0.6 (15% ethyl acetate in petroleum ether).
^{1}H NMR (500 MHz, $CDCl_3$) δ 7.44 (d, J = 8.5 Hz, 2H), 7.41 (d, J = 8.1 Hz, 2H), 7.39 (d, J = 8.6 Hz, 2H), 7.17 (d, J = 8.0 Hz, 2H), 7.13 (s, 1H), 6.89 (d, J = 2.9 Hz, 2H), 6.88 – 6.84 (m, J = 8.8 Hz, 3H), 3.94 (s, 3H), 3.93 (s, 3H), 2.42 (s, 2H), 1.29 – 1.22 (m, 2H), 1.07 (dd, J = 7.3, 4.6 Hz, 12H), 1.00 (s, 9H), 0.21 (s, 6H).
^{13}C NMR (126 MHz, $CDCl_3$) δ 156.28, 155.16, 152.75, 148.21, 140.28, 137.15, 134.37, 131.40, 130.06, 129.42, 127.90, 126.74, 120.49, 120.27, 119.55, 115.28, 115.21, 112.58, 112.54, 102.35, 56.49, 56.36, 25.92, 20.77, 18.45, 17.69, 17.66, 13.13.
IR (thin film, cm^{-1}): 757.773, 838.557, 914.565, 1029.687, 1171.868, 1265.827, 1353.464, 1502.163, 1604.494, 2220.244, 2864.630, 2930.759.
HRMS (ESI): Calculated for $C_{40}H_{52}NO_4Si_2$ [M+H$^+$]: 666.3429; found: 666.3426.

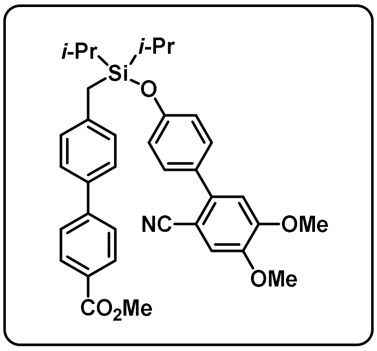

Methyl 4'-((((2'-cyano-4',5'-dimethoxy-[1,1'-biphenyl]-4-yl)oxy)diisopropylsilyl)methyl)-[1,1'-biphenyl]-4-carboxylate (3ah): Compound 3ah was prepared under the **General Procedure 2.4.**
Eluent: petroleum ether/ethyl acetate (90:10, v/v).
Physical State: Yellow sticky liquid
Isolated Yield: 77% (45.7 mg); (*p*:others = 17:1).
R$_f$ Value: 0.4 (20% ethyl acetate in petroleum ether).
^{1}H NMR (500 MHz, $CDCl_3$) δ 8.07 (d, J = 8.2 Hz, 2H), 7.65 (d, J = 8.3 Hz, 2H), 7.50 (d, J = 8.0 Hz, 2H), 7.39 (d, J = 8.4 Hz, 2H), 7.22 (d, J = 8.0 Hz, 2H), 7.13 (s, 1H), 6.90 – 6.87 (m, 3H), 3.94 (s, 3H), 3.93 (s, 6H), 2.45 (s, 2H), 1.29 – 1.26 (m, 2H), 1.08 (d, J = 3.8 Hz, 6H), 1.07 (d, J = 3.8 Hz, 6H).
^{13}C NMR (126 MHz, $CDCl_3$) δ 167.29, 156.16, 152.71, 148.18, 145.68, 140.18, 139.18, 136.22, 131.47, 130.25, 130.07, 129.63, 128.61, 127.29, 126.81, 120.20, 119.52, 115.19, 112.49, 102.33, 56.47, 56.33, 52.28, 21.01, 17.68, 17.63, 13.13.
IR (thin film, cm^{-1}): 754.269, 836.937, 914.513, 1028.638, 1215.631, 1274.655, 1352.813, 1439.595, 1502.625, 1604.551, 1720.768, 2220.149, 2868.327, 2948.117, 3021.740.
HRMS (ESI): Calculated for $C_{36}H_{39}NNaO_5Si$ [M+Na$^+$]: 616.2490; found: 616.2490.

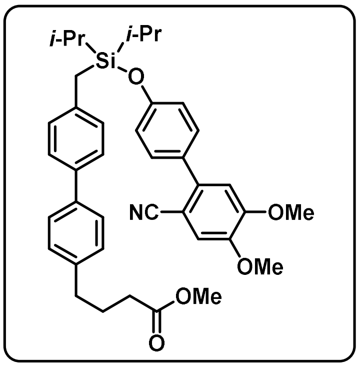

methyl 4-(4'-((((2'-cyano-4',5'-dimethoxy-[1,1'-biphenyl]-4-yl)oxy)diisopropylsilyl)methyl)-[1,1'-biphenyl]-4-yl)butanoate (3ai): Compound 3ai was prepared under the **General Procedure 2.4.**

Eluent: petroleum ether/ethyl acetate (90:10, v/v).

Physical State: Yellow sticky liquid

Isolated Yield: 75% (47.6 mg); (*p*:others = 18:1).

R*f* Value: 0.5 (20% ethyl acetate in petroleum ether).

^{1}H NMR (500 MHz, $CDCl_3$) δ 7.50 (d, J = 8.1 Hz, 2H), 7.45 (d, J = 8.1 Hz, 2H), 7.39 (d, J = 8.6 Hz, 2H), 7.22 (d, J = 8.1 Hz, 2H), 7.19 (d, J = 8.1 Hz, 2H), 7.13 (s, 1H), 6.89 – 6.87 (m, 3H), 3.94 (s, 3H), 3.93 (s, 3H), 3.67 (s, 3H), 2.67 (t, 2H), 2.43 (s, 2H), 2.36 (t, 2H), 2.01 – 1.97 (m, J = 7.5 Hz, 2H), 1.29 – 1.26 (m, J = 7.1 Hz, 2H), 1.08 (d, J = 5.1 Hz, 6H), 1.06 (d, J = 5.1 Hz, 6H).

^{13}C NMR (126 MHz, $CDCl_3$) δ 174.19, 156.25, 152.70, 148.16, 140.25, 139.00, 137.68, 130.05, 129.45, 129.05, 126.98, 120.25, 119.54, 115.20, 112.53, 102.34, 56.48, 56.35, 51.75, 34.93, 33.61, 29.92, 26.69, 20.82, 17.69, 17.65, 13.11.

IR (thin film, cm^{-1}): 759.395, 840.351, 914.449, 1029.861, 1138.131, 1243.147, 1267.008, 1353.145, 1461.785, 1502.975, 1603.632, 1737.188, 2219.620, 2867.247, 2946.091.

HRMS (ESI): Calculated for $C_{39}H_{46}NO_5Si$ [M+H$^+$]: 636.3140; found: 636.3133.

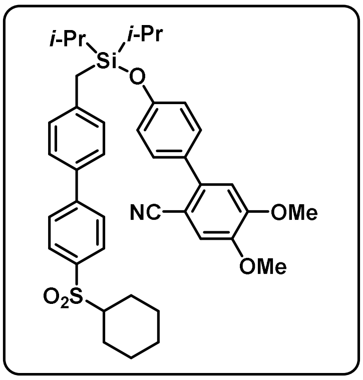

4'-((((4'-(cyclohexylsulfonyl)-[1,1'-biphenyl]-4-yl)methyl)diisopropylsilyl)oxy)-4,5-dimethoxy-[1,1'-biphenyl]-2-carbonitrile (3aj): Compound 3aj was prepared under the **General Procedure 2.4.**

Eluent: petroleum ether/ethyl acetate (95:5, v/v).

Physical State: Yellow sticky liquid

Isolated Yield: 72% (49 mg); (*p*:others = 15:1).

R*f* Value: 0.5 (20% ethyl acetate in petroleum ether).

^{1}H NMR (500 MHz, $CDCl_3$) δ 7.90 (d, J = 8.2 Hz, 2H), 7.70 (d, J = 8.1 Hz, 2H), 7.50 (d, J = 7.9 Hz, 2H), 7.40 (d, J = 8.1 Hz, 2H), 7.23 (d, J = 8.0 Hz, 2H), 7.14 (s, 1H), 6.92 – 6.87 (m, 3H), 3.95 (s, 3H), 3.93 (s, 3H), 3.22 – 3.13 (m, 1H), 2.46 (s, 2H), 1.81 – 1.77 (m, 2H), 1.66 – 1.60 (m, 4H), 1.54 – 1.50 (m, 2H), 1.21 – 1.12 (m, 4H), 1.08 (dd, J = 7.5, 2.4 Hz, 12H).

^{13}C NMR (101 MHz, $CDCl_3$) δ 156.26, 156.14, 152.76, 148.24, 145.33, 140.18, 139.68, 139.58, 137.40, 135.57, 131.54, 130.10, 130.05, 129.74, 129.41, 127.57, 127.39, 127.32, 126.78, 120.26, 120.19, 119.51, 115.24, 112.54, 102.36, 56.49, 56.35, 52.86, 34.22, 25.36, 24.84, 21.06, 17.68, 17.63, 13.16.

IR (thin film, cm^{-1}): 755.512, 840.672, 913.593, 1029.500, 1174.936, 1174.936, 1241.862, 1266.064, 1352.823, 1461.758, 1501.214, 1603.923, 1738.526, 2219.266, 2866.967, 2928.469.

HRMS (ESI): Calculated for $C_{40}H_{47}KNO_5SSi$ [M+K$^+$]: 720.2576; found: 720.2584.

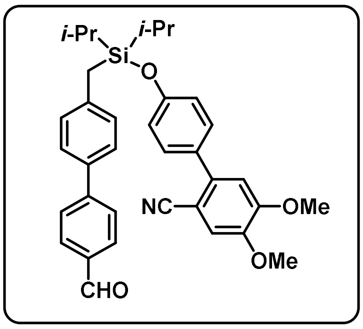

4'-((((4'-formyl-[1,1'-biphenyl]-4-yl)methyl)diisopropylsilyl)oxy)-4,5-dimethoxy-[1,1'-biphenyl]-2-carbonitrile (3ak): Compound 3ak was prepared under the **General Procedure 2.4.**

Eluent: petroleum ether/ethyl acetate (90:10, v/v).

Physical State: yellowish liquid

Isolated Yield: 85% (48 mg); (*p*:others = 16:1).

R*f* Value: 0.4 (20% ethyl acetate in petroleum ether).

^{1}H NMR (500 MHz, $CDCl_3$) δ 10.03 (s, 1H), 7.92 (d, J = 8.0 Hz, 2H), 7.74 (d, J = 8.0 Hz, 2H), 7.52 (d, J = 7.9 Hz, 2H), 7.39 (d, J = 8.3 Hz, 2H), 7.24 (d, J = 7.9 Hz, 2H), 7.13 (s, 1H), 6.90 – 6.87 (m, 3H), 3.94 (d, J = 6.9 Hz, 6H), 2.46 (s, 2H), 1.29 – 1.26 (m, 2H), 1.08 (dd, J = 7.1, 2.9 Hz, 12H).

^{13}C NMR (126 MHz, $CDCl_3$) δ 192.17, 156.15, 152.73, 148.21, 147.29, 140.18, 139.69, 135.94, 135.06, 131.52, 130.48, 130.09, 129.73, 127.45, 127.41, 120.20, 119.52, 115.21, 112.51, 102.36, 56.49, 56.35, 21.09, 17.69, 17.64, 13.16.
IR (thin film, cm^{-1}): 754.059, 818.229, 912.704, 1025.803, 1090.038, 1170.588, 1241.498, 1261.682, 1352.845, 1462.051, 1501.812, 1602.065, 1699.679, 2219.054, 2866.471, 2929.712.
HRMS (ESI): Calculated for $C_{35}H_{38}NO_4Si$ [M+H$^+$]: 564.2565; found: 564.2564.

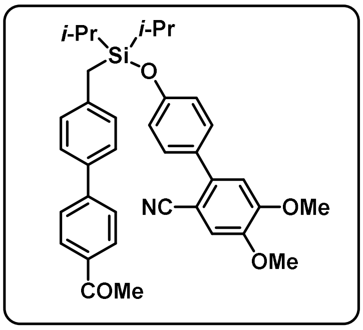

4'-((((4'-acetyl-[1,1'-biphenyl]-4-yl)methyl)diisopropylsilyl)oxy)-4,5-dimethoxy-[1,1'-biphenyl]-2-carbonitrile (3al): Compound 3al was prepared under the **General Procedure 2.4.**
Eluent: petroleum ether/ethyl acetate (90:10, v/v).
Physical State: yellowish liquid
Isolated Yield: 68% (39.3 mg); (*p*:others = 20:1).
R_f Value: 0.4 (20% ethyl acetate in petroleum ether).
^{1}H NMR (500 MHz, $CDCl_3$) δ 8.00 (d, J = 8.4 Hz, 2H), 7.67 (d, J = 8.4 Hz, 2H), 7.51 (d, J = 8.1 Hz, 2H), 7.39 (d, J = 8.5 Hz, 2H), 7.23 (d, J = 8.1 Hz, 2H), 7.13 (s, 1H), 6.90 – 6.87 (m, 3H), 3.94 (d, J = 6.6 Hz, 6H), 2.62 (s, 3H), 2.46 (s, 2H), 1.29 – 1.25 (m, 2H), 1.08 (dd, J = 7.4, 3.7 Hz, 12H).
^{13}C NMR (126 MHz, $CDCl_3$) δ 198.01, 156.17, 152.73, 148.21, 145.86, 140.19, 139.33, 136.10, 135.68, 131.50, 130.08, 129.68, 129.10, 127.30, 126.98, 120.21, 120.14, 119.52, 115.22, 112.52, 102.36, 56.49, 56.34, 26.84, 21.05, 17.69, 17.64, 13.15.
IR (thin film, cm^{-1}): 755.412, 840.358, 914.657, 1028.686, 1174.192, 1267.009, 1355.161, 1462.643, 1502.832, 1603.170, 1680.821, 2219.589, 2867.760, 2944.903.
HRMS (ESI): Calculated for $C_{36}H_{40}NO_4Si$ [M+H$^+$]: 578.2721; found: 578.2725.

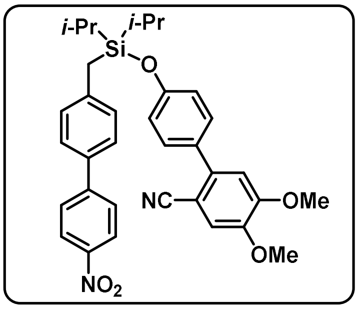

4'-((diisopropyl((4'-nitro-[1,1'-biphenyl]-4-yl)methyl)silyl)oxy)-4,5-dimethoxy-[1,1'-biphenyl]-2-carbonitrile (3am): Compound 3am was prepared under the **General Procedure 2.4.**

Eluent: petroleum ether/ethyl acetate (90:10, v/v).

Physical State: Yellow sticky liquid

Isolated Yield: 79% (46 mg); (*p*:others = 16:1).

R_f Value: 0.5 (20% ethyl acetate in petroleum ether).

^{1}H NMR (400 MHz, $CDCl_3$) δ 8.26 (d, J = 8.8 Hz, 2H), 7.72 (d, J = 8.8 Hz, 2H), 7.50 (d, J = 8.2 Hz, 2H), 7.39 (d, 2H), 7.24 (d, J = 8.2 Hz, 2H), 7.13 (s, 1H), 6.91 – 6.86 (m, 3H), 3.95 (s, 3H), 3.93 (s, 3H), 2.47 (s, 2H), 1.31 – 1.26 (m, 3H), 1.08 (dd, J = 7.4, 2.1 Hz, 12H).

^{13}C NMR (101 MHz, $CDCl_3$) δ 156.09, 152.73, 148.22, 147.69, 146.90, 140.29, 140.13, 134.98, 131.55, 130.10, 129.84, 127.50, 127.43, 124.28, 120.16, 119.51, 115.18, 112.49, 102.34, 56.48, 56.35, 21.15, 17.68, 17.63, 13.16.

IR (thin film, cm^{-1}): 754.870, 835.774, 916.490, 1029.091, 1215.419, 1267.749, 1345.033, 1503.614, 1602.093, 2936.459, 3021.230.

HRMS (ESI): Calculated for $C_{34}H_{37}N_2O_5Si$ [M+H$^+$]: 581.2466; found: 581.2463.

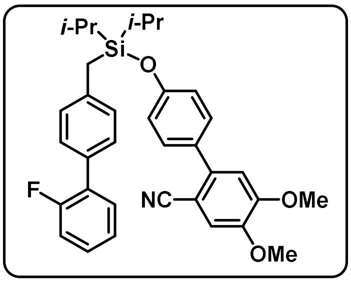

4'-((((2'-fluoro-[1,1'-biphenyl]-4-yl)methyl)diisopropylsilyl)oxy)-4,5-dimethoxy-[1,1'-biphenyl]-2-carbonitrile (3an): Compound 3an was prepared under the **General Procedure 2.4.**

Eluent: petroleum ether/ethyl acetate (95:5, v/v).

Physical State: Yellow sticky liquid

Isolated Yield: 92% (51 mg); (*p*:others = 13:1).

R_f Value: 0.6 (20% ethyl acetate in petroleum ether).

^{1}H NMR (500 MHz, $CDCl_3$) δ 7.45 – 7.37 (m, 5H), 7.29 – 7.26 (m, 1H), 7.21 (d, J = 8.2 Hz, 2H), 7.19 – 7.16 (m, 1H), 7.12 (dt, J = 2.6, 1.9 Hz, 2H), 6.89 (s, 1H), 6.87 (d, J = 8.6

Hz, 2H), 3.94 (d, J = 7.7 Hz, 6H), 2.45 (s, 2H), 1.30 – 1.27 (m, 2H), 1.08 (dd, J = 7.2, 6.6 Hz, 12H).

^{13}C NMR (126 MHz, $CDCl_3$) δ 160.97, 156.21, 152.70, 148.15, 140.25, 138.40, 131.41, 130.84, 130.81, 130.05, 129.13, 129.10, 128.74, 128.68, 124.48, 124.45, 120.23, 119.53, 116.31, 116.13, 115.19, 112.52, 102.33, 56.48, 56.34, 20.99, 17.69, 17.64, 13.10.

^{19}F NMR (471 MHz, $CDCl_3$) δ -117.95.

IR (thin film, cm^{-1}): 755.306, 908.906, 1029.432, 1215.403, 1267.670, 1503.444, 1604.709, 2220.236, 2947.531, 3020.678.

HRMS (ESI): Calculated for $C_{34}H_{37}FNO_3Si$ [M+H$^+$]: 554.2520; found: 554.2521.

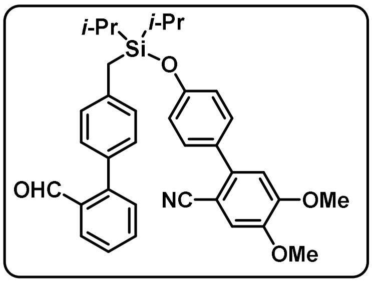

4'-((((2'-formyl-[1,1'-biphenyl]-4-yl)methyl)diisopropylsilyl)oxy)-4,5-dimethoxy-[1,1'-biphenyl]-2-carbonitrile (3ao): Compound 3ao was prepared under the **General Procedure 2.4.**

Eluent: petroleum ether/ethyl acetate (90:10, v/v).

Physical State: Yellow sticky liquid

Isolated Yield: 77% (43 mg); (*p*:others = 15:1).

R*f* Value: 0.4 (20% ethyl acetate in petroleum ether).

^{1}H NMR (500 MHz, $CDCl_3$) δ 10.00 (s, 1H), 8.02 (d, J = 7.9 Hz, 1H), 7.63 (t, J = 7.7 Hz, 1H), 7.51 – 7.44 (m, 2H), 7.42 (d, J = 8.3 Hz, 2H), 7.28 (d, 4H), 7.15 (s, 1H), 6.93 (s, 1H), 6.89 (d, J = 8.4 Hz, 2H), 3.98 (s, 3H), 3.95 (s, 3H), 2.50 (s, 2H), 1.34 – 1.29 (m, 2H), 1.10 (dd, J = 7.5, 4.7 Hz, 12H).

^{13}C NMR (126 MHz, $CDCl_3$) δ 192.92, 156.13, 152.75, 148.19, 146.30, 140.18, 139.21, 134.03, 133.87, 133.75, 131.54, 130.99, 130.34, 130.09, 129.13, 127.68, 127.66, 120.16, 115.20, 112.55, 102.33, 77.48, 76.98, 56.49, 56.38, 21.05, 17.67, 17.62, 13.14.

IR (thin film, cm^{-1}): 754.059, 818.229, 912.704, 1025.803, 1090.038, 1170.588, 1241.498, 1261.682, 1352.845, 1462.051, 1501.812, 1602.065, 1699.679, 2219.054, 2866.471, 2929.712

HRMS (ESI): Calculated for $C_{35}H_{37}NNaO_4Si$ [M+Na$^+$]: 586.2384; found: 586.2381.

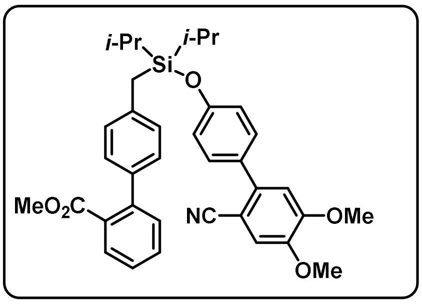

methyl 4'-((((2'-cyano-4',5'-dimethoxy-[1,1'-biphenyl]-4-yl)oxy)diisopropylsilyl)methyl)-[1,1'-biphenyl]-2-carboxylate (3ap): Compound 3ap was prepared under the **General Procedure 2.4.**

Eluent: petroleum ether/ethyl acetate (90:10, v/v).

Physical State: Yellow sticky liquid

Isolated Yield: 70% (42 mg); (*p*:others = 25:1).

R_f Value: 0.4 (20% ethyl acetate in petroleum ether).

^{1}H NMR (400 MHz, $CDCl_3$) δ 7.76 (dd, J = 8.0, 1.5 Hz, 1H), 7.49 (td, J = 7.5, 1.5 Hz, 1H), 7.41 (d, J = 8.6 Hz, 2H), 7.37 (d, J = 7.3 Hz, 2H), 7.18 (s, 4H), 7.13 (s, 1H), 6.94 – 6.86 (m, 3H), 3.94 (s, 3H), 3.92 (s, 3H), 3.59 (s, 3H), 2.45 (s, 2H), 1.31 – 1.20 (m, 2H), 1.08 (dd, J = 7.4, 1.7 Hz, 12H).

^{13}C NMR (101 MHz, $CDCl_3$) δ 169.87, 156.15, 152.66, 148.12, 142.20, 140.15, 137.82, 137.63, 131.40, 131.25, 130.75, 130.04, 129.78, 128.80, 128.41, 126.99, 120.17, 119.44, 115.15, 112.48, 102.27, 56.42, 56.28, 52.04, 20.87, 17.62, 17.58, 13.10.

IR (thin film, cm^{-1}): 754.269, 836.937, 914.513, 1028.638, 1215.631, 1274.655, 1352.813, 1439.595, 1502.625, 1604.551, 1720.768, 2220.149, 2868.327, 2948.117, 3021.740.

HRMS (ESI): Calculated for $C_{36}H_{39}NNaO_5Si$ [M+Na$^+$]: 616.2490; found: 616.2490.

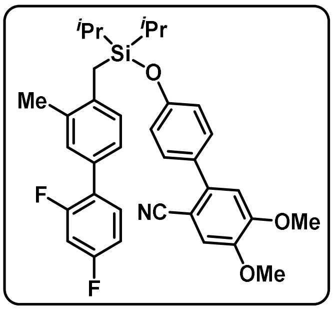

4'-((((2',4'-difluoro-3-methyl-[1,1'-biphenyl]-4-yl)methyl)diisopropylsilyl)oxy)-4,5-dimethoxy-[1,1'-biphenyl]-2-carbonitrile (3bq): Compound 3bq was prepared under the **General Procedure 2.4.**

Eluent: petroleum ether/ethyl acetate (95:5, v/v).

Physical State: Yellow sticky liquid

Isolated Yield: 73% (42.7 mg); (*p*:others = 12:1).

R_f Value: 0.6 (15% ethyl acetate in petroleum ether).

^{1}H NMR (400 MHz, $CDCl_3$) δ 7.41 – 7.34 (m, 1H), 7.31 (d, J = 8.7 Hz, 2H), 7.25 – 7.20 (m, 3H), 7.11 (s, 1H), 6.92 – 6.87 (m, 1H), 6.86 – 6.82 (m, 2H), 6.69 (d, J = 8.5 Hz, 2H), 3.93 (d, J = 3.5 Hz, 6H), 2.41 (s, 2H), 2.34 (s, 3H), 1.36 – 1.27 (m, 2H), 1.12 (d, J = 7.4 Hz, 6H), 1.07 (d, J = 7.4 Hz, 6H).
^{13}C NMR (101 MHz, $CDCl_3$) δ 155.99, 152.72, 148.18, 140.25, 137.36, 136.01, 131.61, 131.56, 131.47, 131.29, 130.96, 130.93, 129.92, 129.53, 127.30, 126.53, 126.50, 120.28, 119.96, 119.47, 115.26, 112.55, 111.65, 111.61, 111.44, 111.40, 104.63, 104.36, 104.11, 102.36, 56.48, 56.31, 20.76, 18.08, 17.81, 17.60, 13.44.
^{19}F NMR (471 MHz, $CDCl_3$) δ -112.45, -113.46.
IR (thin film, cm^{-1}): 753.706, 848.278, 916.770, 1030.313, 1138.776, 1266.956, 1353.535, 1503.258, 1603.523, 2220.236, 2868.554, 2941.937.
HRMS (ESI): Calculated for $C_{35}H_{38}F_2NO_3Si$ [M+H$^+$]: 586.2584; found: 586.2585.

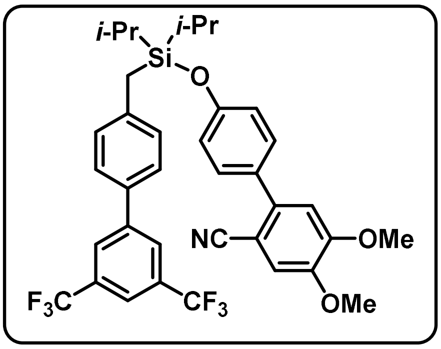

4'-((((3',5'-bis(trifluoromethyl)-[1,1'-biphenyl]-4-yl)methyl)diisopropylsilyl)oxy)-4,5-dimethoxy-[1,1'-biphenyl]-2-carbonitrile (3ar): Compound 3ar was prepared under the **General Procedure 2.4.**
Eluent: petroleum ether/ethyl acetate (95:5, v/v).
Physical State: Yellow sticky liquid
Isolated Yield: 62% (41.6 mg); (*p*:others = 12:1).
R*f* Value: 0.6 (20% ethyl acetate in petroleum ether).
^{1}H NMR (500 MHz, $CDCl_3$) δ 7.99 (s, 2H), 7.79 (s, 1H), 7.48 (d, J = 8.1 Hz, 2H), 7.40 (d, J = 8.5 Hz, 2H), 7.26 (s, 2H), 7.13 (s, 1H), 6.90 (d, J = 8.3 Hz, 3H), 3.94 (d, J = 7.7 Hz, 6H), 2.47 (s, 2H), 1.29 – 1.27 (m, 2H), 1.08 (d, J = 7.2 Hz, 12H).
^{13}C NMR (126 MHz, $CDCl_3$) δ 156.12, 152.76, 148.24, 143.33, 140.16, 134.48, 132.32, 132.06, 131.58, 130.13, 129.95, 127.23, 127.02, 124.74, 122.58, 120.60, 120.18, 119.51, 115.23, 112.49, 102.39, 56.50, 56.34, 21.10, 17.69, 17.64, 13.20.
^{19}F NMR (471 MHz, $CDCl_3$) δ -62.81.
IR (thin film, cm^{-1}): 754.324, 908.520, 1038.122, 1137.610, 1278.538, 1382.375, 1503.647, 1605.333, 2220.236, 2948.703.
HRMS (ESI): Calculated for $C_{36}H_{36}F_6NO_3Si$ [M+H$^+$]: 672.2456; found: 672.2454.

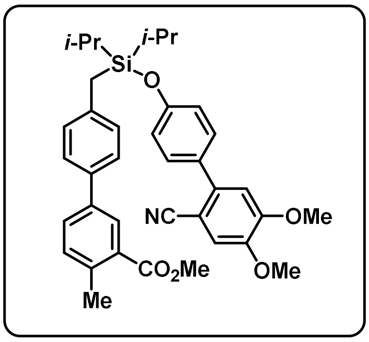

methyl 4'-((((2'-cyano-4',5'-dimethoxy-[1,1'-biphenyl]-4-yl)oxy)diisopropylsilyl)methyl)-4-methyl-[1,1'-biphenyl]-3-carboxylate (3as): Compound 3as was prepared under the **General Procedure 2.4.**

Eluent: petroleum ether/ethyl acetate (90:10, v/v).

Physical State: Yellow sticky liquid

Isolated Yield: 80% (48.6 mg); (*p*:others = 14:1).

R_f Value: 0.4 (20% ethyl acetate in petroleum ether).

^{1}H NMR (500 MHz, $CDCl_3$) δ 8.13 (d, J = 2.0 Hz, 1H), 7.61 (dd, J = 7.9, 2.1 Hz, 1H), 7.47 (d, J = 8.2 Hz, 2H), 7.39 (d, J = 8.6 Hz, 2H), 7.28 (d, J = 8.1 Hz, 1H), 7.20 (d, J = 8.2 Hz, 2H), 7.13 (s, 1H), 6.90 – 6.88 (m, J = 8.3 Hz, 3H), 3.94 (s, 3H), 3.93 (s, 3H), 3.91 (s, 3H), 2.61 (s, 3H), 2.44 (s, 2H), 1.27 – 1.23 (m, J = 10.0, 4.7 Hz, 3H), 1.08 (d, J = 3.8 Hz, 6H), 1.07 (d, J = 3.8 Hz, 6H).

^{13}C NMR (101 MHz, $CDCl_3$) δ 168.35, 156.23, 152.74, 148.20, 138.82, 138.21, 136.37, 132.35, 131.45, 130.31, 130.07, 129.56, 129.01, 126.95, 120.24, 115.24, 112.55, 102.36, 56.49, 56.34, 52.07, 21.55, 20.90, 17.69, 17.65, 13.15.

IR (thin film, cm^{-1}): 754.269, 836.937, 914.513, 1028.638, 1215.631, 1274.655, 1352.813, 1439.595, 1502.625, 1604.551, 1720.768, 2220.149, 2868.327, 2948.117, 3021.740.

HRMS (ESI): Calculated for $C_{37}H_{42}NO_5Si$ [M+H$^+$]: 608.2827; found: 608.2825.

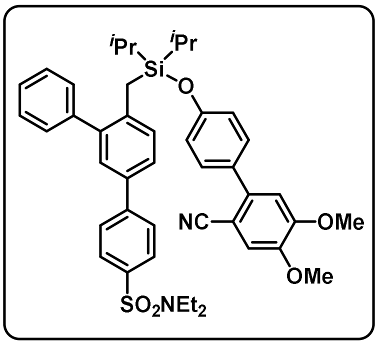

4'-((((2'-cyano-4',5'-dimethoxy-[1,1'-biphenyl]-4-yl)oxy)diisopropylsilyl)methyl)-N,N-diethyl-[1,1':3',1''-terphenyl]-4-sulfonamide (3kt): Compound 3kt was prepared under the **General Procedure 2.4.**

Eluent: petroleum ether/ethyl acetate (90:10, v/v).

Physical State: Yellow solid

Isolated Yield: 76% (56 mg); (*p*:others = 13:1).

^{1}H NMR (400 MHz, CDCl3) δ 7.90 (d, 2H), 7.82 (d, J = 8.6 Hz, 2H), 7.71 (dd, J = 8.6, 1.7 Hz, 4H), 7.53 – 7.48 (m, 1H), 7.46 (d, J = 3.0 Hz, 1H), 7.43 (s, 1H), 7.41 (d, J = 4.2 Hz, 1H), 7.37 (d, J = 7.6 Hz, 2H), 7.32 (d, J = 8.7 Hz, 2H), 7.13 (s, 1H), 6.86 (s, 1H), 6.67 (d, J = 8.7 Hz, 2H), 3.94 (s, 3H), 3.93 (s, 3H), 3.30 – 3.21 (m, 8H), 2.54 (s, 2H), 1.25 (s, 2H), 1.17 (t, 6H), 1.14 (t, 6H), 0.89 (d, J = 7.4 Hz, 6H), 0.87 (d, J = 7.4 Hz, 6H).

^{13}C NMR (101 MHz, $CDCl_3$) δ 156.02, 152.74, 148.21, 144.93, 143.42, 142.24, 141.91, 140.49, 140.19, 138.77, 137.21, 135.79, 131.32, 131.26, 130.85, 130.45, 129.98, 129.94, 129.82, 129.41, 128.59, 128.08, 127.93, 127.69, 127.44, 127.30, 126.09, 120.00, 119.51, 115.24, 112.51, 102.34, 56.50, 56.35, 42.32, 42.28, 41.83, 17.87, 17.56, 17.51, 17.39, 17.37, 14.41, 13.28.

IR (thin film, cm^{-1}): 755.306, 908.906, 1029.432, 1215.403, 1267.670, 1503.444, 1604.709, 2220.236, 2947.531, 3020.678.

HRMS (ESI): Calculated for $C_{44}H_{50}N_2NaO_5SSi$ [M+Na$^+$]: 769.3102; found: 769.3099.

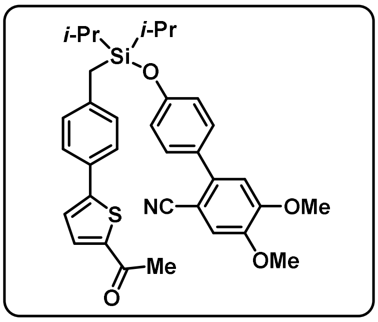

4'-(((4-(5-acetylthiophen-2-yl)benzyl)diisopropylsilyl)oxy)-4,5-dimethoxy-[1,1'-biphenyl]-2-carbonitrile (3au): Compound 3au was prepared under the **General Procedure 2.4.**

Eluent: petroleum ether/ethyl acetate (90:10, v/v).

Physical State: Yellow sticky liquid

Isolated Yield: 58% (34 mg); (*p*:others = 17:1).

R*f* Value: 0.4 (20% ethyl acetate in petroleum ether).

^{1}H NMR (500 MHz, $CDCl_3$) δ 7.63 (d, J = 3.9 Hz, 1H), 7.51 (d, J = 8.1 Hz, 2H), 7.39 (d, J = 8.4 Hz, 2H), 7.27 (d, 1H), 7.16 (d, J = 8.0 Hz, 2H), 7.13 (s, 1H), 6.90 – 6.86 (m, J = 8.7 Hz, 3H), 3.95 (s, 3H), 3.93 (s, 3H), 2.55 (s, 3H), 2.43 (s, 2H), 1.26 – 1.22 (m, J = 14.9, 7.2 Hz, 3H), 1.07 (d, 6H), 1.06 (d, J = 2.2 Hz, 6H).

^{13}**C NMR** (126 MHz, $CDCl_3$) δ 190.80, 156.09, 153.46, 152.72, 148.20, 142.49, 140.49, 140.17, 133.81, 131.55, 130.11, 129.85, 129.72, 126.39, 123.41, 120.18, 119.55, 115.18, 112.50, 102.34, 56.49, 56.37, 26.75, 21.32, 17.68, 17.63, 13.15.

IR (thin film, cm^{-1}): 683.202, 755.042, 918.007, 1034.686, 1173.754, 1215.807, 1272.561, 1355.465, 1443.889, 1503.601, 1604.259, 1657.229, 2220.529, 2868.000, 2944.749, 3020.650.

HRMS (ESI): Calculated for $C_{34}H_{38}NO_4SSi$ [M+H$^+$]: 584.2285; found: 584.2289.

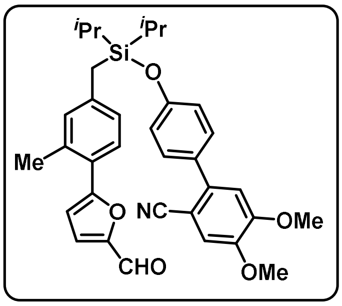

4'-(((4-(5-formylfuran-2-yl)-3-methylbenzyl)diisopropylsilyl)oxy)-4,5-dimethoxy-[1,1'-biphenyl]-2-carbonitrile (3gv): Compound 3gv was prepared under the **General Procedure 2.4.**

Eluent: petroleum ether/ethyl acetate (95:5, v/v).

Physical State: Yellow solid

Isolated Yield: 65% (37 mg); (*p*:others = 15:1).

R*f* Value: 0.6 (15% ethyl acetate in petroleum ether).

^{1}**H NMR** (500 MHz, $CDCl_3$) δ 9.62 (d, J = 12.1 Hz, 1H), 7.77 (d, J = 7.5 Hz, 0.51H), 7.67 (d, J = 8.0 Hz, 0.73H), 7.60 (dd, J = 7.4, 1.0 Hz, 0.51H), 7.40 (d, J = 8.6 Hz, 2H), 7.34 – 7.30 (m, 1H), 7.28 (d, J = 3.7 Hz, 0.33H), 7.13 (s, 1H), 7.07 – 6.97 (m, 2H), 6.90 – 6.87 (m, 2H), 6.72 – 6.67 (m, 1H), 3.95 (s, 3H), 3.93 (s, 3H), 2.47 (s, 2H), 2.41 (s, 2H), 2.32 (s, 1H), 1.24 – 1.19 (m, 2H), 1.06 (dd, J = 7.4, 1.8 Hz, 12H).

^{13}**C NMR** (101 MHz, $CDCl_3$) δ 177.58, 160.16, 156.35, 156.13, 153.01, 152.77, 152.73, 151.65, 149.38, 148.24, 148.18, 140.97, 140.28, 140.18, 138.09, 137.13, 136.04, 135.97, 132.26, 131.54, 131.36, 130.51, 130.30, 130.11, 130.02, 129.71, 128.42, 127.95, 127.28, 127.06, 126.18, 125.23, 125.06, 123.15, 122.82, 122.55, 120.30, 120.20, 120.14, 119.52, 115.86, 115.26, 112.56, 111.21, 110.58, 107.78, 102.35, 56.50, 56.37, 56.35, 31.80, 31.13, 29.91, 29.28, 22.87, 22.55, 22.10, 21.57, 21.30, 20.99, 20.73, 20.01, 18.98, 17.71, 17.68, 17.65, 17.63, 17.61, 14.33, 13.22, 13.14, 11.64.

IR (thin film, cm^{-1}): 756.227, 839.555, 913.906, 1030.781, 1174.222, 1264.866, 1441.807, 1503.380, 1603.767, 1663.104, 1731.032, 2219.338, 2867.828, 2925.216

HRMS (ESI): Calculated for $C_{34}H_{38}NO_5Si$ [M+H$^+$]: 568.2518; found: 568.2519.

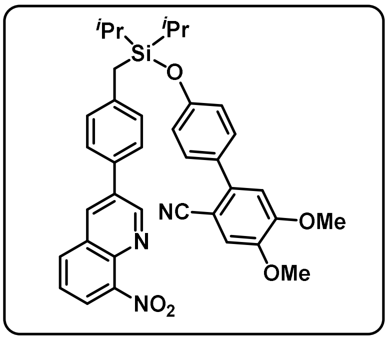

4'-((diisopropyl(4-(8-nitroquinolin-3-yl)benzyl)silyl)oxy)-4,5-dimethoxy-[1,1'-biphenyl]-2-carbonitrile (3aw): Compound 3aw was prepared under the **General Procedure 2.4.**

Eluent: petroleum ether/ethyl acetate (95:5, v/v).

Physical State: Yellow solid

Isolated Yield: 63% (39.7 mg); (*p*:others = 20:1).

R*f* Value: 0.5 (20% ethyl acetate in petroleum ether).

^{1}H NMR (500 MHz, $CDCl_3$) δ 9.33 (d, J = 2.3 Hz, 0.43H), 8.37 (d, J = 2.3 Hz, 0.41H), 8.09 – 8.00 (m, 1H), 7.77 (d, J = 7.6 Hz, 0.54H), 7.63 – 7.60 (m, 0.63H), 7.60 – 7.57 (m, 1H), 7.45 (d, J = 8.1 Hz, 1H), 7.39 (d, J = 8.1 Hz, 2H), 7.33 – 7.28 (m, 2H), 7.17 (d, J = 8.2 Hz, 1H), 7.13 (s, 1H), 6.91 – 6.87 (m, 4H), 3.94 (s, 3H), 3.92 (s, 3H), 2.49 (s, 0.90H), 2.42 (s, 1.33H), 1.31 – 1.24 (m, 2H), 1.10 (dd, J = 7.4, 4.7 Hz, 5H), 1.07 (dd, J = 7.4, 3.8 Hz, 7H).

^{13}C NMR (126 MHz, $CDCl_3$) δ 156.24, 156.09, 152.74, 152.69, 152.44, 148.21, 148.14, 144.00, 141.51, 140.23, 140.13, 138.48, 137.39, 137.31, 135.65, 132.91, 132.53, 132.41, 131.53, 131.38, 130.11, 130.08, 130.05, 129.39, 127.92, 127.46, 127.25, 126.77, 125.77, 125.21, 123.58, 120.25, 120.19, 120.16, 119.52, 115.18, 112.52, 112.47, 102.30, 67.10, 56.46, 56.33, 47.29, 21.15, 20.78, 17.70, 17.67, 17.64, 13.15, 13.10.

IR (thin film, cm^{-1}): 754.870, 835.774, 916.490, 1029.091, 1215.419, 1267.749, 1345.033, 1503.614, 1602.093, 2936.459, 3021.230.

HRMS (ESI): Calculated for $C_{37}H_{37}KN_3O_5Si$ [M+K$^+$]: 670.2134; found: 670.2144.

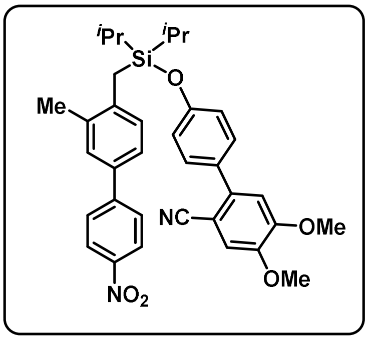

4'-((diisopropyl((3-methyl-4'-nitro-[1,1'-biphenyl]-4-yl)methyl)silyl)oxy)-4,5-dimethoxy-[1,1'-biphenyl]-2-carbonitrile (3bm): Compound 3bm was prepared under the **General Procedure 2.4.**

Eluent: petroleum ether/ethyl acetate (95:5, v/v).

Physical State: Yellow sticky liquid

Isolated Yield: 78% (46.4 mg); (*p*:others = 18:1).

R*f* Value: 0.5 (15% ethyl acetate in petroleum ether).

^{1}H NMR (500 MHz, $CDCl_3$) δ 8.25 (d, J = 8.7 Hz, 2H), 7.73 (d, J = 8.7 Hz, 2H), 7.39 (d, J = 6.0 Hz, 2H), 7.32 (d, J = 8.5 Hz, 2H), 7.28 (s, 1H), 7.13 (s, 1H), 6.86 (s, 1H), 6.72 (d, J = 8.5 Hz, 2H), 3.94 (s, 3H), 3.94 (s, 3H), 2.45 (s, 2H), 2.39 (s, 3H), 1.36 – 1.30 (m, J = 14.8, 7.4 Hz, 3H), 1.14 (d, J = 7.4 Hz, 6H), 1.08 (d, J = 7.4 Hz, 6H).

^{13}C NMR (101 MHz, $CDCl_3$) δ 155.89, 152.72, 148.22, 147.86, 146.83, 140.12, 139.12, 136.70, 135.19, 131.37, 130.20, 129.94, 129.39, 127.49, 124.88, 124.21, 119.89, 119.47, 115.18, 112.47, 102.32, 56.47, 56.32, 20.83, 18.29, 17.78, 17.58, 13.49.

IR (thin film, cm^{-1}): 760.377, 839.268, 917.841, 1029.594, 1215.130, 1344.078, 1502.738, 1601.202, 2220.236, 2946.171, 3020.977.

HRMS (ESI): Calculated for $C_{35}H_{38}N_2NaO_5Si$ [M+Na$^+$]: 617.2442; found: 617.2438.

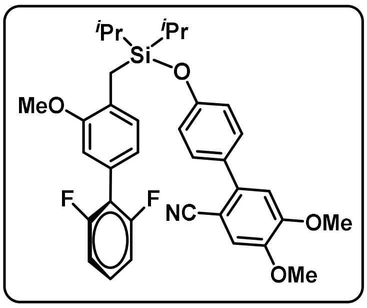

4'-((((2',6'-difluoro-3-methoxy-[1,1'-biphenyl]-4-yl)methyl)diisopropylsilyl)oxy)-4,5-dimethoxy-[1,1'-biphenyl]-2-carbonitrile (3cx): Compound 3cx was prepared under the **General Procedure 2.4.**

Eluent: petroleum ether/ethyl acetate (95:5, v/v).

Physical State: Light yellow sticky liquid

Isolated Yield: 70% (42.11 mg); (*p*:others = 15:1).

R*f* Value: 0.6 (15% ethyl acetate in petroleum ether).

^{1}H NMR (400 MHz, $CDCl_3$) δ 7.78 (d, J = 7.5 Hz, 0.48H), 7.62 (d, J = 7.4 Hz, 0.49H), 7.43 – 7.30 (m, 3H), 7.17 – 7.12 (m, 2H), 7.11 – 7.04 (m, 1H), 6.90 (d, 1H), 6.88 – 6.78 (m, 3H), 6.76 (d, J = 8.0 Hz, 0.27H), 6.61 (d, J = 8.9 Hz, 0.65H), 3.96 (s, 3H), 3.93 (s, 3H), 3.77 – 3.69 (m, 3H), 2.44 – 2.34 (m, 2H), 1.24 – 1.18 (m, 2H), 1.11 – 1.02 (m, 12H).

^{13}C NMR (101 MHz, $CDCl_3$) δ 156.83, 156.49, 156.22, 155.95, 152.73, 148.16, 148.13, 144.54, 141.75, 140.40, 140.35, 132.80, 131.22, 131.03, 130.35, 130.19, 129.95, 129.88, 129.85, 128.37, 127.81, 127.45, 127.30, 125.86, 124.91, 120.53, 120.28, 120.20, 120.10, 120.07, 119.56, 115.23, 112.62, 112.57, 111.68, 110.11, 102.32, 65.40, 56.49, 56.36, 56.35, 55.29, 55.19, 55.00, 50.58, 17.55, 17.53, 17.51, 14.86, 14.82, 13.57, 13.50.

^{19}F NMR (471 MHz, $CDCl_3$) δ -112.73, -112.83, -113.17, -113.56.
IR (thin film, cm^{-1}): 754.854, 910.831, 1030.443, 1137.715, 1268.273, 1353.117, 1463.278, 1503.139, 1603.990, 2220.783, 2868.498, 2944.246.
HRMS (ESI): Calculated for [M+Na$^+$]: 617.2442; found: 617.2438.

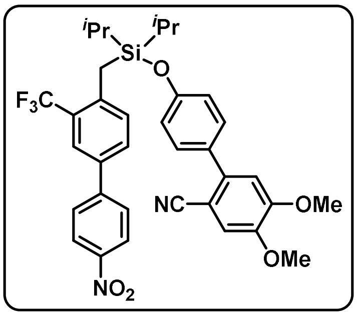

4'-((diisopropyl((4'-nitro-3-(trifluoromethyl)-[1,1'-biphenyl]-4-yl)methyl)silyl)oxy)-4,5-dimethoxy-[1,1'-biphenyl]-2-carbonitrile (3dm): Compound 3dm was prepared under the **General Procedure 2.4.**
Eluent: petroleum ether/ethyl acetate (95:5, v/v).
Physical State: Yellow sticky liquid
Isolated Yield: 78% (50.59 mg); (*p*:others = 18:1).
R$_f$ Value: 0.5 (15% ethyl acetate in petroleum ether).
^{1}H NMR (500 MHz, $CDCl_3$) δ 8.28 (d, J = 8.8 Hz, 2H), 7.83 (d, J = 1.5 Hz, 1H), 7.73 (d, J = 8.8 Hz, 2H), 7.66 (dd, J = 8.1, 1.6 Hz, 1H), 7.48 (d, J = 8.1 Hz, 1H), 7.38 (d, J = 8.6 Hz, 2H), 7.11 (s, 1H), 6.87 (d, J = 3.8 Hz, 2H), 6.86 (s, 1H), 3.94 (s, 3H), 3.93 (s, 3H), 2.65 (s, 2H), 1.38 – 1.28 (m, J = 14.8, 7.4 Hz, 2H), 1.09 (d, J = 7.5 Hz, 6H), 1.01 (d, J = 7.4 Hz, 6H).
^{13}C NMR (101 MHz, $CDCl_3$) δ 155.88, 152.75, 148.26, 147.40, 146.19, 140.02, 139.58, 135.38, 132.68, 131.61, 130.24, 130.14, 129.02, 128.73, 127.78, 126.01, 125.26, 125.20, 124.42, 123.28, 120.02, 119.48, 115.14, 112.45, 102.32, 56.49, 56.35, 18.02, 17.64, 17.40, 13.50.
^{19}F NMR (471 MHz, $CDCl_3$) δ -59.73.
IR (thin film, cm^{-1}): 681.834, 840.362, 915.783, 1140.178, 1263.288, 1346.501, 1463.337, 1502.900, 1602.286, 2219.613, 2869.355, 2947.071.
HRMS (ESI): Calculated for $C_{35}H_{35}F_3N_2NaO_5Si$ [M+Na$^+$]: 671.2160; found: 671.2167.

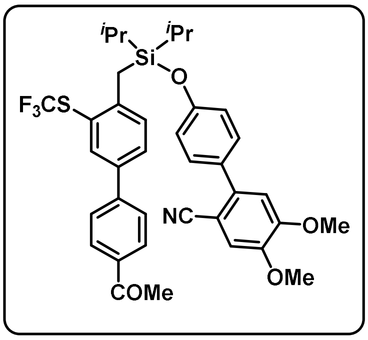

4'-((((4'-acetyl-3-((trifluoromethyl)thio)-[1,1'-biphenyl]-4-yl)methyl)diisopropylsilyl)oxy)-4,5-dimethoxy-[1,1'-biphenyl]-2-carbonitrile (3el): Compound 3el was prepared under the **General Procedure 2.4.**

Eluent: petroleum ether/ethyl acetate (95:5, v/v).

Physical State: Yellow solid

Isolated Yield: 62% (42 mg); (*p*:others = 17:1).

R_f Value: 0.5 (15% ethyl acetate in petroleum ether).

^{1}H NMR (400 MHz, $CDCl_3$) δ 8.01 (d, J = 8.4 Hz, 2H), 7.90 (s, 1H), 7.66 (d, J = 8.4 Hz, 2H), 7.63 (dd, J = 8.1, 2.2 Hz, 1H), 7.43 (d, J = 8.2 Hz, 1H), 7.34 (d, J = 8.5 Hz, 2H), 7.11 (s, 1H), 6.85 (s, 1H), 6.77 (d, J = 8.5 Hz, 2H), 3.93 (s, 6H), 2.82 (s, 2H), 2.63 (s, 3H), 1.35 – 1.28 (m, 1H), 1.11 (d, J = 7.4 Hz, 6H), 1.08 (d, J = 7.3 Hz, 6H).

^{13}C NMR (101 MHz, $CDCl_3$) δ 197.89, 155.81, 152.74, 148.23, 145.54, 144.17, 140.07, 137.64, 136.94, 136.24, 131.57, 131.23, 130.09, 129.93, 129.23, 127.95, 127.28, 127.14, 125.23, 120.22, 119.87, 119.49, 115.21, 112.49, 102.33, 56.50, 56.34, 26.88, 19.91, 17.79, 17.59, 13.59.

^{19}F NMR (471 MHz, $CDCl_3$) δ -42.44.

IR (thin film, cm^{-1}): 755.969, 908.993, 1031.263, 1116.169, 1215.691, 1266.879, 1355.965, 1503.737, 1605.085, 1682.199, 2220.236, 2929.565.

HRMS (ESI): Calculated for $C_{37}H_{39}F_3NO_4SSi$ [M+H$^+$]: 678.2316; found: 678.2312.

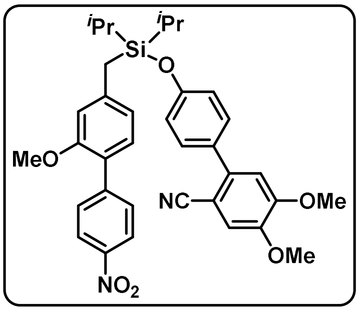

4'-((diisopropyl((2-methoxy-4'-nitro-[1,1'-biphenyl]-4-yl)methyl)silyl)oxy)-4,5-dimethoxy-[1,1'-biphenyl]-2-carbonitrile (3fm): Compound 3fm was prepared under the **General Procedure 2.4.**

Eluent: petroleum ether/ethyl acetate (95:5, v/v).

Physical State: Yellow sticky liquid

Isolated Yield: 69% (42.13 mg); (*p*:others = 12:1).

R_f Value: 0.5 (15% ethyl acetate in petroleum ether).

^{1}H NMR (500 MHz, $CDCl_3$) δ 8.21 (d, J = 8.9 Hz, 2H), 7.68 (d, J = 8.8 Hz, 2H), 7.40 (d, J = 8.5 Hz, 2H), 7.19 (d, J = 7.8 Hz, 1H), 7.13 (s, 1H), 6.90 (d, J = 8.6 Hz, 2H), 6.87 (s, 1H), 6.83 (dd, J = 7.8, 1.6 Hz, 1H), 6.76 (s, 1H), 3.93 (s, 6H), 3.69 (s, 3H), 2.47 (s, 2H), 1.33 – 1.26 (m, 2H), 1.11 (d, J = 3.7 Hz, 6H), 1.10 (d, J = 3.6 Hz, 6H).

^{13}C NMR (101 MHz, $CDCl_3$) δ 156.50, 156.13, 152.76, 148.26, 146.50, 145.79, 141.70, 140.07, 131.51, 130.68, 130.34, 130.12, 124.64, 123.38, 121.84, 120.07, 119.50, 115.85, 115.22, 112.48, 112.37, 102.31, 56.50, 56.34, 55.67, 21.63, 17.74, 17.67, 13.23.

IR (thin film, cm⁻¹): 681.834, 840.362, 915.783, 1140.178, 1263.288, 1346.501, 1463.337, 1502.900, 1602.286, 2219.613, 2869.355, 2947.071.
HRMS (ESI): Calculated for $C_{35}H_{39}N_2O_6Si$ [M+H⁺]: 611.2524; found: 6111.2525.

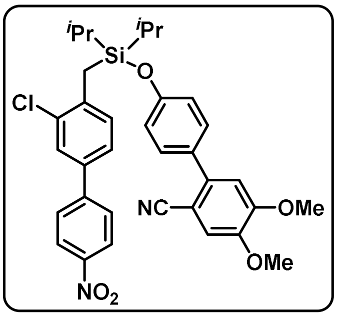

4'-((((3-chloro-4'-nitro-[1,1'-biphenyl]-4-yl)methyl)diisopropylsilyl)oxy)-4,5-dimethoxy-[1,1'-biphenyl]-2-carbonitrile (3hm): Compound 3hm was prepared under the **General Procedure 2.4.**
Eluent: petroleum ether/ethyl acetate (95:5, v/v).
Physical State: Yellow sticky liquid
Isolated Yield: 76% (46.7 mg); (*p*:others = 11:1).
R_f Value: 0.5 (15% ethyl acetate in petroleum ether).
^{1}H NMR (400 MHz, $CDCl_3$) δ 8.23 (d, J = 8.7 Hz, 2H), 7.68 (d, J = 8.7 Hz, 2H), 7.58 (s, 1H), 7.39 – 7.35 (m, J = 8.5 Hz, 3H), 7.30 (d, J = 8.0 Hz, 1H), 7.11 (s, 1H), 6.88 – 6.84 (m, J = 8.3 Hz, 3H), 3.93 (s, 3H), 3.91 (s, 3H), 2.61 (s, 2H), 1.37 – 1.30 (m, J = 14.9, 7.5 Hz, 2H), 1.11 – 1.07 (m, J = 7.5 Hz, 12H).
^{13}C NMR (101 MHz, $CDCl_3$) δ 155.87, 152.67, 148.15, 147.17, 146.10, 140.01, 138.43, 136.61, 134.08, 131.41, 130.00, 128.21, 127.55, 125.56, 124.25, 119.96, 119.45, 115.08, 112.39, 102.20, 56.40, 56.27, 18.64, 17.60, 17.46, 13.60.
IR (thin film, cm⁻¹): 684.366, 754.894, 839.516, 915.704, 1029.429, 1173.504, 1215.557, 1266.285, 1349.978, 1462.717, 1502.955, 1603.933, 2220.334, 2867.192, 2929.604.
HRMS (ESI): Calculated for $C_{34}H_{35}ClN_2O_5Si$ [M+H⁺]: 615.2077; found: 615.2069.

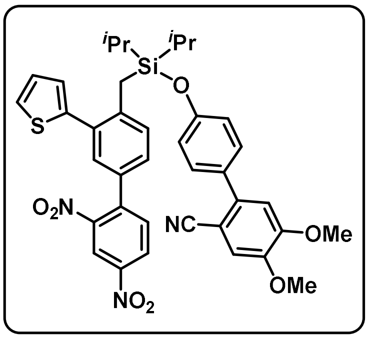

4'-((((2',4'-dinitro-3-(thiophen-2-yl)-[1,1'-biphenyl]-4 yl)methyl)diisopropylsilyl)oxy)-4,5-dimethoxy-[1,1'-biphenyl]-2-carbonitrile (3iy): Compound 3iy was prepared under the **General Procedure 2.4.**
Eluent: petroleum ether/ethyl acetate (95:5, v/v).

Physical State: Yellow sticky liquid
Isolated Yield: 66% (46.7 mg); (*p*:others = 20:1).
R*f* Value: 0.5 (15% ethyl acetate in petroleum ether).
^{1}H NMR (400 MHz, $CDCl_3$) δ 9.07 (d, J = 2.8 Hz, 0.44H), 8.57 (d, J = 2.4 Hz, 1H), 8.46 (dd, J = 9.3, 2.8 Hz, 0.50H), 8.37 (dd, J = 8.7, 2.4 Hz, 1H), 7.66 (d, J = 8.5 Hz, 1H), 7.32 (dd, J = 14.1, 8.8 Hz, 4H), 7.23 – 7.17 (m, 2H), 7.12 (s, 1H), 7.00 (s, 1H), 6.86 (s, 1H), 6.64 (d, J = 8.5 Hz, 2H), 3.95 (s, 3H), 3.93 (s, 3H), 2.42 (s, 2H), 1.19 – 1.11 (m, 1H), 0.94 (dd, J = 10.7, 7.3 Hz, 12H).
^{13}C NMR (126 MHz, $CDCl_3$) δ 155.98, 152.76, 148.23, 146.95, 140.95, 140.09, 138.00, 133.41, 132.91, 132.17, 131.82, 131.59, 131.49, 131.34, 130.94, 130.74, 130.37, 129.97, 128.90, 126.63, 125.05, 122.09, 121.44, 120.01, 119.48, 115.22, 112.50, 102.26, 56.48, 56.36, 18.53, 17.51, 17.39, 13.17.
IR (thin film, cm^{-1}): 764.257, 839.250, 911.825, 1029.155, 1138.323, 1265.590, 1346.490, 1462.912, 1531.281, 1531.281, 1601.326, 2219.491, 2867.534, 2943.945, 3096.758.
HRMS (ESI): Calculated for $C_{38}H_{38}N_3O_7SSi$ [M+H$^+$]: 708.2124; found: 708.2123.

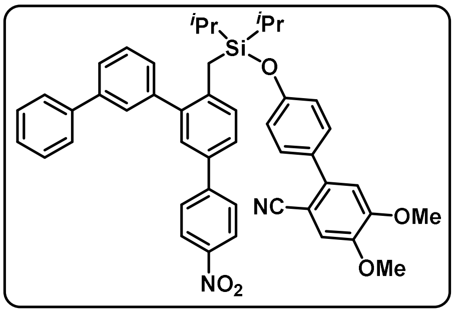

4'-((diisopropyl((4-nitro-[1,1':3',1'':3'',1'''-quaterphenyl]-4'-yl)methyl)silyl)oxy)-4,5-dimethoxy-[1,1'-biphenyl]-2-carbonitrile (3jm): Compound 3jm was prepared under the **General Procedure 2.4.**
Eluent: petroleum ether/ethyl acetate (95:5, v/v).
Physical State: Yellow sticky liquid
Isolated Yield: 72% (52 mg); (*p*:others = 12:1).
R*f* Value: 0.5 (15% ethyl acetate in petroleum ether).
^{1}H NMR (500 MHz, $CDCl_3$) δ 8.25 (d, J = 8.9 Hz, 2H), 7.77 (d, J = 8.9 Hz, 2H), 7.60 – 7.57 (m, 4H), 7.54 (s, 1H), 7.51 (d, J = 7.6 Hz, 1H), 7.45 – 7.40 (m, 3H), 7.37 (d, J = 9.3 Hz, 2H), 7.34 (d, 1H), 7.29 (d, J = 8.5 Hz, 2H), 7.12 (s, 1H), 6.83 (s, 1H), 6.65 (d, J = 8.5 Hz, 2H), 3.93 (d, J = 3.2 Hz, 6H), 2.60 (s, 2H), 1.17 – 1.08 (m, 2H), 0.90 (d, J = 7.5 Hz, 6H), 0.89 (d, J = 3.4 Hz, 6H).
^{13}C NMR (101 MHz, $CDCl_3$) δ 155.92, 152.70, 148.19, 147.41, 147.00, 142.21, 141.67, 140.12, 138.08, 135.23, 131.02, 129.98, 129.50, 129.01, 128.73, 127.61, 127.39, 126.33, 126.17, 124.30, 119.95, 119.50, 115.17, 112.46, 102.30, 56.49, 56.33, 18.14, 17.51, 17.39, 13.29.

IR (thin film, cm⁻¹): 667.970, 754.768, 854.059, 917.122, 1029.420, 1241.881, 1264.903, 1347.035, 1462.394, 1502.360, 1600.905, 2219.863, 2868.439, 2946.171.
HRMS (ESI): Calculated for $C_{46}H_{44}N_2NaO_5Si$ [M+Na$^+$]: 755.2912; found: 755.2911.

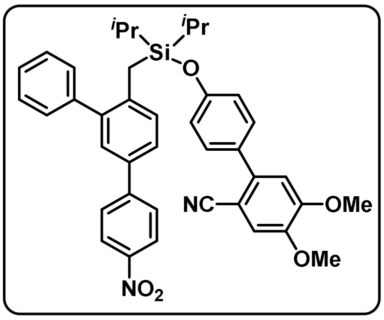

4'-((diisopropyl((4-nitro-[1,1':3',1''-terphenyl]-4'-yl)methyl)silyl)oxy)-4,5-dimethoxy-[1,1'-biphenyl]-2-carbonitrile (3km): Compound 3km was prepared under the **General Procedure 2.4.**
Eluent: petroleum ether/ethyl acetate (95:5, v/v).
Physical State: Yellow sticky liquid
Isolated Yield: 71% (46.6 mg); (*p*:others = 26:1).
R_f Value: 0.5 (15% ethyl acetate in petroleum ether).
^{1}H NMR (400 MHz, $CDCl_3$) δ 8.25 (d, J = 8.8 Hz, 2H), 7.75 (d, J = 8.8 Hz, 2H), 7.53 – 7.36 (m, 8H), 7.32 (d, J = 8.5 Hz, 2H), 7.13 (s, 1H), 6.86 (s, 1H), 6.67 (d, J = 8.5 Hz, 2H), 3.94 (s, 3H), 3.93 (s, 3H), 2.55 (s, 2H), 1.15 – 1.04 (m, J = 14.7, 7.5 Hz, 2H), 0.88 (t, J = 7.5 Hz, 12H).
^{13}C NMR (126 MHz, $CDCl_3$) δ 155.97, 152.71, 148.20, 147.45, 146.98, 142.36, 141.71, 140.14, 138.00, 135.14, 131.32, 130.99, 129.97, 129.78, 129.48, 128.63, 127.57, 127.40, 126.20, 124.28, 119.96, 119.50, 115.19, 112.48, 102.31, 56.48, 56.33, 17.99, 17.49, 17.38, 13.29.
IR (thin film, cm⁻¹): 684.366, 754.894, 839.516, 915.704, 1029.429, 1173.504, 1215.557, 1266.285, 1349.978, 1462.717, 1502.955, 1603.933, 2220.334, 2867.192, 2929.604.
HRMS (ESI): Calculated for $C_{40}H_{41}N_2O_5Si$ [M+H$^+$]: 657.2735; found: 657.2731.

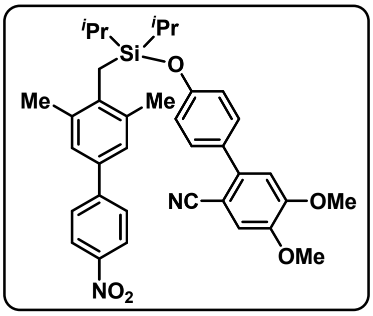

4'-((((3,5-dimethyl-4'-nitro-[1,1'-biphenyl]-4-yl)methyl)diisopropylsilyl)oxy)-4,5-dimethoxy-[1,1'-biphenyl]-2-carbonitrile (3lm): Compound 3lm was prepared under the **General Procedure 2.4.**

Eluent: petroleum ether/ethyl acetate (95:5, v/v).

Physical State: Yellow sticky liquid

Isolated Yield: 74% (45 mg); (*p*:others = 23:1).

R*f* Value: 0.5 (15% ethyl acetate in petroleum ether).

1H NMR (400 MHz, $CDCl_3$) δ 8.21 (d, J = 8.8 Hz, 2H), 7.69 (d, J = 8.8 Hz, 2H), 7.29 (d, J = 8.5 Hz, 2H), 7.24 (s, 2H), 7.10 (s, 1H), 6.83 (s, 1H), 6.71 (d, J = 8.5 Hz, 2H), 3.92 (s, 6H), 2.46 (s, 2H), 2.41 (s, 6H), 1.33 – 1.24 (m, 3H), 1.13 (d, J = 7.4 Hz, 6H), 1.01 (d, J = 7.3 Hz, 6H).

^{13}C NMR (101 MHz, $CDCl_3$) δ 155.84, 152.71, 148.21, 147.95, 146.74, 140.07, 138.33, 136.60, 134.68, 131.24, 129.89, 127.45, 127.08, 124.11, 119.69, 119.46, 115.17, 112.44, 102.27, 56.46, 56.30, 21.74, 17.71, 17.47, 16.14, 14.31.

IR (thin film, cm^{-1}): 703.443, 759.493, 841.305, 915.568, 1030.130, 1346.474, 1462.776, 1503.123, 1601.638, 2219.501, 2867.339, 2943.837.

HRMS (ESI): Calculated for $C_{36}H_{40}N_2NaO_5Si$ [M+Na$^+$]: 631.2598; found: 631.2597.

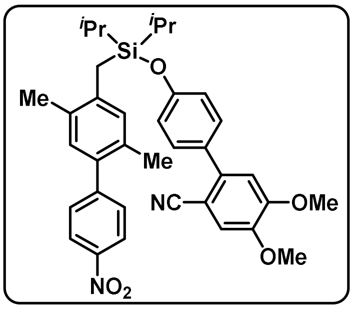

4'-((((2,5-dimethyl-4'-nitro-[1,1'-biphenyl]-4-yl)methyl)diisopropylsilyl)oxy)-4,5-dimethoxy-[1,1'-biphenyl]-2-carbonitrile (3mm): Compound 3mm was prepared under the **General Procedure 2.4.**

Eluent: petroleum ether/ethyl acetate (95:5, v/v).

Physical State: Yellow sticky liquid

Isolated Yield: 72% (43.8 mg); (*p*:others = 30:1).

R*f* Value: 0.5 (15% ethyl acetate in petroleum ether).

^{1}H NMR (400 MHz, $CDCl_3$) δ 8.22 (d, J = 8.8 Hz, 2H), 7.46 (d, J = 8.8 Hz, 2H), 7.33 (d, J = 8.6 Hz, 2H), 7.12 (s, 1H), 7.05 (s, 1H), 6.95 (s, 1H), 6.86 (s, 1H), 6.72 (d, J = 8.6 Hz, 2H), 3.93 (s, 3H), 3.93 (s, 3H), 2.38 (s, 2H), 2.30 (s, 3H), 2.20 (s, 3H), 1.35 – 1.27 (m, J = 14.6, 7.4 Hz, 2H), 1.12 (d, J = 7.4 Hz, 6H), 1.07 (d, J = 7.4 Hz, 6H).

^{13}C NMR (101 MHz, $CDCl_3$) δ 157.51, 156.00, 152.73, 149.24, 146.71, 140.13, 138.09, 136.19, 133.59, 132.23, 131.87, 131.59, 130.37, 129.92, 123.48, 119.95, 119.47, 115.20, 112.48, 102.32, 56.49, 56.33, 20.10, 17.92, 17.81, 17.61, 13.50.

IR (thin film, cm^{-1}): 703.443, 759.493, 841.305, 915.568, 1030.130, 1346.474, 1462.776, 1503.123, 1601.638, 2219.501, 2867.339, 2943.837.

HRMS (ESI): Calculated for $C_{36}H_{41}N_2O_5Si$ [M+H$^+$]: 609.2779; found: 609.2777.

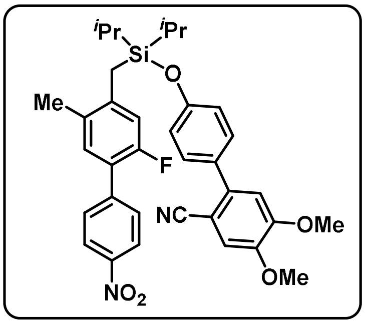

4'-((((2-fluoro-5-methyl-4'-nitro-[1,1'-biphenyl]-4-yl)methyl)diisopropylsilyl)oxy)-4,5-dimethoxy-[1,1'-biphenyl]-2-carbonitrile (3nm): Compound 3nm was prepared under the **General Procedure 2.4.**

Eluent: petroleum ether/ethyl acetate (95:5, v/v).

Physical State: Yellow sticky liquid

Isolated Yield: 67% (41 mg); (*p*:others = 30:1).

R*f* Value: 0.5 (15% ethyl acetate in petroleum ether).

^{1}H NMR (500 MHz, $CDCl_3$) δ 8.23 (d, J = 8.8 Hz, 2H), 7.69 (d, J = 7.7 Hz, 2H), 7.32 (d, J = 8.6 Hz, 2H), 7.17 (d, J = 8.2 Hz, 1H), 7.11 (s, 1H), 6.98 (d, J = 12.0 Hz, 1H), 6.85 (s, 1H), 6.74 (d, J = 8.6 Hz, 2H), 3.93 (s, 3H), 3.92 (s, 3H), 2.40 (s, 2H), 2.31 (s, 3H), 1.37 – 1.29 (m, J = 14.8, 7.5 Hz, 2H), 1.13 (d, J = 7.5 Hz, 6H), 1.08 (d, J = 7.4 Hz, 6H).

^{13}C NMR (126 MHz, $CDCl_3$) δ 159.06, 157.09, 155.74, 152.74, 148.25, 146.95, 142.94, 141.36, 141.30, 140.04, 132.35, 131.93, 131.91, 131.51, 130.00, 129.75, 129.72, 123.78, 122.79, 122.69, 119.81, 119.45, 116.86, 116.68, 115.16, 112.48, 102.30, 77.23, 56.48, 56.32, 19.92, 18.67, 17.76, 17.55, 13.50.

^{19}F NMR (471 MHz, $CDCl_3$) δ -122.52.

IR (thin film, cm^{-1}): 667.970, 754.768, 854.059, 917.122, 1029.420, 1241.881, 1264.903, 1347.035, 1462.394, 1502.360, 1600.905, 2219.863, 2868.439, 2946.171.

HRMS (ESI): Calculated for $C_{35}H_{38}FN_2O_5Si$ [M+H$^+$]: 613.2529; found: 613.2535.

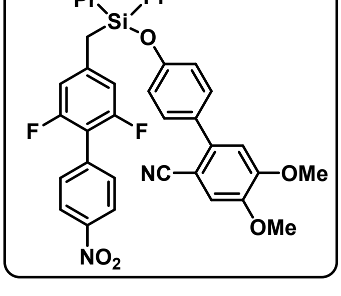

4'-((((2,6-difluoro-4'-nitro-[1,1'-biphenyl]-4-yl)methyl)diisopropylsilyl)oxy)-4,5-dimethoxy-[1,1'-biphenyl]-2-carbonitrile (3om): Compound 3om was prepared under the **General Procedure 2.4.**

Eluent: petroleum ether/ethyl acetate (95:5, v/v).

Physical State: Yellow sticky liquid

Isolated Yield: 70% (43.16 mg); (*p*:others = 30:1).
R_f Value: 0.5 (15% ethyl acetate in petroleum ether).
^{1}H NMR (500 MHz, $CDCl_3$) δ 8.27 (d, J = 8.4 Hz, 2H), 7.63 (d, J = 8.1 Hz, 2H), 7.42 (d, J = 8.2 Hz, 2H), 7.13 (s, 1H), 6.91 (d, J = 8.2 Hz, 2H), 6.88 (s, 1H), 6.80 (d, J = 8.9 Hz, 2H), 3.93 (s, 6H), 2.44 (s, 2H), 1.33 – 1.24 (m, 2H), 1.10 (d, J = 7.0 Hz, 12H).
^{13}C NMR (101 MHz, $CDCl_3$) δ 160.96, 158.40, 155.79, 152.77, 148.30, 147.42, 143.50, 142.23, 139.98, 136.61, 131.88, 131.52, 131.49, 130.22, 123.53, 120.04, 119.45, 115.23, 112.58, 112.54, 112.33, 102.36, 56.49, 56.32, 21.73, 17.66, 17.58, 13.24.
IR (thin film, cm^{-1}): 755.519, 854.273, 919.270, 1029.627, 1265.742, 1344.040, 1462.690, 1516.756, 1599.882, 1732.572, 2219.466, 2867.065, 2927.088.
HRMS (ESI): Calculated for $C_{34}H_{34}F_2N_2NaO_5Si$ [M+Na$^+$]: 639.2097; found: 639.2095.

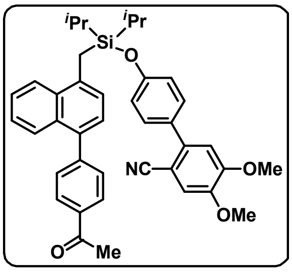

4'-((((4-(4-acetylphenyl)naphthalen-1-yl)methyl)diisopropylsilyl)oxy)-4,5-dimethoxy-[1,1'-biphenyl]-2-carbonitrile (3pl): Compound 3pl was prepared under the **General Procedure 2.4.**
Eluent: petroleum ether/ethyl acetate (93:7, v/v).
Physical State: Yellow sticky liquid
Isolated Yield: 73% (45.8 mg); (*p*:others = 13:1).
R_f Value: 0.4 (20% ethyl acetate in petroleum ether).
^{1}H NMR (400 MHz, $CDCl_3$) δ 8.17 (d, J = 8.4 Hz, 1H), 8.05 (d, J = 8.0 Hz, 2H), 7.84 (d, J = 8.3 Hz, 1H), 7.58 (d, J = 7.8 Hz, 2H), 7.50 (t, 1H), 7.40 (dd, J = 12.9, 7.3 Hz, 2H), 7.32 – 7.27 (m, J = 6.9 Hz, 3H), 7.12 (s, 1H), 6.83 (s, 1H), 6.71 (d, J = 8.3 Hz, 2H), 3.93 (s, 6H), 2.89 (s, 2H), 2.67 (s, 3H), 1.36 – 1.27 (m, J = 14.7, 7.3 Hz, 2H), 1.07 (t, J = 8.1 Hz, 12H).
^{13}C NMR (101 MHz, $CDCl_3$) δ 198.11, 156.00, 152.71, 148.18, 146.47, 140.16, 136.46, 136.20, 132.49, 131.99, 131.74, 131.24, 130.69, 130.64, 129.88, 128.49, 128.42, 126.81, 126.33, 126.04, 125.62, 125.41, 124.87, 123.20, 119.92, 115.24, 112.52, 102.30, 56.48, 56.31, 26.89, 18.19, 17.78, 17.66, 13.63.
IR (thin film, cm^{-1}): 755.969, 908.993, 1031.263, 1116.169, 1215.691, 1266.879, 1355.965, 1503.737, 1605.085, 1682.199, 2220.236, 2929.565.
HRMS (ESI): Calculated for $C_{40}H_{42}NO_4Si$ [M+H$^+$]: 627.2831; found: 627.2831.

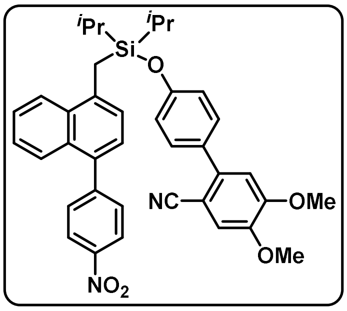

4'-((diisopropyl((4-(4-nitrophenyl)naphthalen-1-yl)methyl)silyl)oxy)-4,5-dimethoxy-[1,1'-biphenyl]-2-carbonitrile (3pm): Compound 3pm was prepared under the **General Procedure 2.4.**

Eluent: petroleum ether/ethyl acetate (93:7, v/v).

Physical State: Yellow sticky liquid

Isolated Yield: 70% (44.15 mg); (*p*:others = 12:1).

R*f* Value: 0.4 (20% ethyl acetate in petroleum ether).

^{1}H NMR (400 MHz, $CDCl_3$) δ 8.31 (d, J = 8.7 Hz, 2H), 8.20 (d, J = 8.5 Hz, 1H), 7.79 (d, J = 8.4 Hz, 1H), 7.64 (d, J = 8.7 Hz, 2H), 7.53 (t, J = 6.9 Hz, 1H), 7.46 – 7.41 (m, 1H), 7.41 – 7.33 (m, 2H), 7.29 (d, J = 8.2 Hz, 2H), 7.12 (s, 1H), 6.84 (s, 1H), 6.70 (d, J = 8.7 Hz, 2H), 3.93 (s, 6H), 2.90 (s, 2H), 1.35 – 1.26 (m, 2H), 1.10 – 1.04 (m, 12H).

^{13}C NMR (101 MHz, $CDCl_3$) δ 155.93, 152.70, 148.33, 148.18, 147.10, 140.07, 137.03, 136.39, 135.18, 132.47, 131.43, 131.25, 131.22, 129.88, 129.86, 127.02, 126.83, 126.44, 126.01, 125.91, 125.82, 125.55, 124.83, 123.67, 123.61, 122.76, 119.86, 119.46, 115.19, 112.46, 102.24, 56.46, 56.30, 18.28, 17.76, 17.63, 13.62, 13.56.

IR (thin film, cm^{-1}): 684.366, 754.894, 839.516, 915.704, 1029.429, 1173.504, 1215.557, 1266.285, 1349.978, 1462.717, 1502.955, 1603.933, 2220.334, 2867.192, 2929.604.

HRMS (ESI): Calculated for $C_{38}H_{39}N_2O_5Si$ [M+H$^+$]: 631.2623; found: 631.2623.

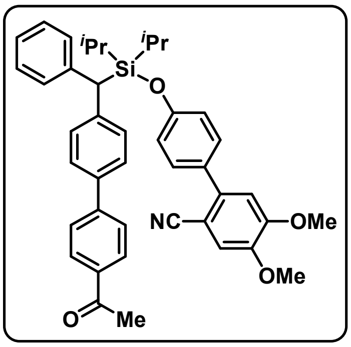

4'-((((4'-acetyl-[1,1'-biphenyl]-4-yl)(phenyl)methyl)diisopropylsilyl)oxy)-4,5-dimethoxy-[1,1'-biphenyl]-2-carbonitrile (3ql): Compound 3ql was prepared under the **General Procedure 2.4.**

Eluent: petroleum ether/ethyl acetate (80:20, v/v).

Physical State: Colorless sticky liquid

Isolated Yield: 61% (39.8 mg); (*p*:others = 30:1).
R*f* Value: 0.5 (25% ethyl acetate in petroleum ether).
^{1}H NMR (400 MHz, $CDCl_3$) δ 8.00 (d, J = 8.5 Hz, 2H), 7.67 (d, J = 8.5 Hz, 2H), 7.62 – 7.54 (m, 4H), 7.52 (d, J = 8.2 Hz, 2H), 7.39 (d, J = 8.7 Hz, 2H), 7.30 (t, J = 7.7 Hz, 2H), 7.19 (t, J = 7.3 Hz, 1H), 7.13 (s, 1H), 6.91 – 6.84 (m, 3H), 3.95 (s, 3H), 3.93 (s, 3H), 3.85 (s, 1H), 2.62 (s, 3H), 1.35 – 1.27 (m, 2H), 0.97 – 0.88 (m, 12H).
^{13}C NMR (101 MHz, $CDCl_3$) δ 198.03, 156.03, 152.73, 148.21, 145.70, 142.71, 141.84, 140.15, 137.18, 135.78, 131.57, 130.16, 130.10, 129.68, 129.10, 128.81, 127.46, 127.10, 126.05, 120.05, 119.51, 115.20, 112.51, 102.35, 56.49, 56.35, 42.61, 26.85, 18.19, 18.11, 17.86, 13.88, 13.86.
IR (thin film, cm^{-1}): 702.201, 751.239, 842.602, 920.306, 1030.573, 1137.818, 1267.356, 1355.441, 1463.179, 1503.159, 1603.469, 1681.190, 2219.231, 2868.170, 2947.052.
HRMS (ESI): Calculated for $C_{42}H_{43}NNaO_4Si$ [M+Na$^+$]: 676.2864; found: 676.2865.

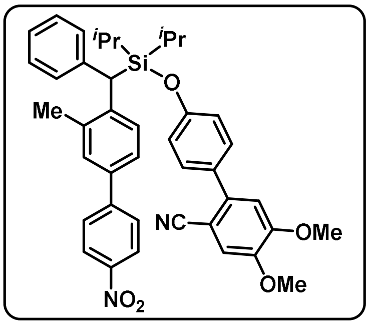

4'-((diisopropyl((3-methyl-4'-nitro-[1,1'-biphenyl]-4-yl)(phenyl)methyl)silyl)oxy)-4,5-dimethoxy-[1,1'-biphenyl]-2-carbonitrile (3rm): Compound 3rm was prepared under the **General Procedure 2.4.**
Eluent: petroleum ether/ethyl acetate (95:5, v/v).
Physical State: Yellow sticky liquid
Isolated Yield: 64% (42.9 mg); (*p*:others = 20:1).
R*f* Value: 0.5 (15% ethyl acetate in petroleum ether).
^{1}H NMR (400 MHz, $CDCl_3$) δ 8.30 – 8.24 (m, 3H), 8.05 (d, J = 8.0 Hz, 1H), 7.73 (dd, J = 12.0, 8.8 Hz, 4H), 7.54 (s, 3H), 7.45 (s, 1H), 7.39 (d, J = 8.7 Hz, 2H), 7.14 (s, 1H), 6.90 – 6.87 (m, 3H), 4.12 (s, 1H), 3.94 (d, 6H), 2.48 (s, 3H), 1.42 – 1.33 (m, 2H), 1.04 (d, J = 7.5 Hz, 6H), 0.96 (dd, J = 7.4, 3.4 Hz, 6H).
^{13}C NMR (101 MHz, $CDCl_3$) δ 155.85, 152.83, 148.37, 147.50, 147.41, 147.14, 142.48, 141.24, 140.05, 137.64, 136.72, 136.11, 131.84, 131.51, 130.19, 129.98, 127.70, 127.65, 125.30, 124.34, 124.30, 120.01, 119.49, 115.27, 112.53, 102.45, 56.54, 56.37, 37.49, 32.15, 22.92, 20.76, 18.33, 18.22, 18.10, 17.70, 14.33, 14.22, 13.77.
IR (thin film, cm^{-1}): 755.519, 854.273, 919.270, 1029.627, 1265.742, 1344.040, 1462.690, 1516.756, 1599.882, 1732.572, 2219.466, 2867.065, 2927.088.
HRMS (ESI): Calculated for $C_{41}H_{43}N_2O_5Si$ [M+H$^+$]: 671.2934; found: 671.2933.

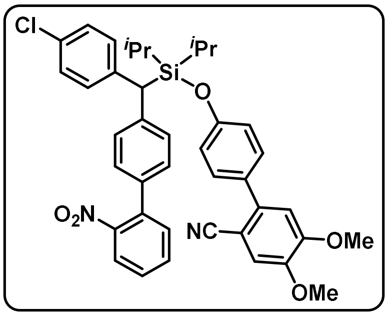

4'-((((4-chlorophenyl)(2'-nitro-[1,1'-biphenyl]-4-yl)methyl)diisopropylsilyl)oxy)-4,5-dimethoxy-[1,1'-biphenyl]-2-carbonitrile (3sz): Compound 3sz was prepared under the **General Procedure 2.4.**

Eluent: petroleum ether/ethyl acetate (95:5, v/v).

Physical State: Yellow sticky liquid

Isolated Yield: 66% (45.6 mg); (*p*:others = 20:1).

R_f Value: 0.5 (15% ethyl acetate in petroleum ether).

^{1}H NMR (400 MHz, $CDCl_3$) δ 7.85 – 7.81 (m, 1H), 7.59 (t, J = 7.5 Hz, 1H), 7.52 – 7.47 (m, 2H), 7.46 (d, J = 6.0 Hz, 2H), 7.41 (t, 3H), 7.39 (d, J = 5.9 Hz, 1H), 7.37 – 7.33 (m, 1H), 7.28 (d, J = 8.0 Hz, 2H), 7.24 (d, J = 8.4 Hz, 1H), 7.13 (s, 1H), 6.90 (d, J = 8.8 Hz, 1H), 6.83 (dd, J = 8.7, 5.4 Hz, 2H), 3.95 (s, 3H), 3.93 (s, 3H), 3.81 (d, J = 10.7 Hz, 1H), 1.35 – 1.26 (m, 2H), 0.95 (m, 12H).

^{13}C NMR (126 MHz, $CDCl_3$) δ 155.86, 155.82, 152.77, 149.55, 148.23, 142.30, 141.97, 140.45, 140.36, 140.18, 140.14, 137.95, 136.58, 136.31, 135.09, 132.46, 132.43, 132.16, 131.95, 131.86, 131.73, 131.69, 131.17, 130.97, 130.21, 130.14, 129.59, 129.32, 129.15, 129.00, 128.89, 128.87, 128.35, 128.33, 128.20, 125.69, 124.27, 124.23, 120.01, 119.51, 115.24, 112.62, 112.59, 102.37, 56.51, 56.38, 42.15, 41.98, 18.14, 18.09, 17.89, 17.85, 17.78, 17.73, 13.92, 13.86, 13.76, 13.60.

IR (thin film, cm^{-1}): 755.519, 854.273, 919.270, 1029.627, 1265.742, 1344.040, 1462.690, 1516.756, 1599.882, 1732.572, 2219.466, 2867.065, 2927.088.

HRMS (ESI): Calculated for $C_{40}H_{40}ClN_2O_5Si$ [M+H$^+$]: 691.2398; found: 691.2399.

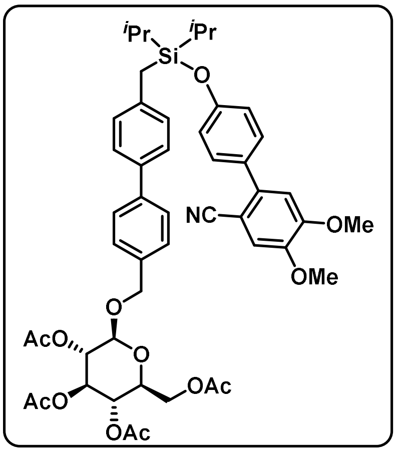

(2S,3S,4R,5S,6S)-2-(acetoxymethyl)-6-((4'-((((2'-cyano-4',5'-dimethoxy-[1,1'-biphenyl]-4-yl)oxy)diisopropylsilyl)methyl)-[1,1'-biphenyl]-4-yl)methoxy)tetrahydro-2H-pyran-3,4,5-triyl triacetate (5aa): Compound 5aa was prepared under the **General Procedure 2.4.**

Eluent: petroleum ether/ethyl acetate (80:20, v/v).

Physical State: Yellow sticky liquid

Isolated Yield: 68% (60.9 mg); (*p*:others = 20:1).

R_f Value: 0.3 (30% ethyl acetate in petroleum ether).

^{1}H NMR (500 MHz, $CDCl_3$) δ 7.56 (d, J = 8.1 Hz, 2H), 7.46 (d, J = 8.2 Hz, 2H), 7.39 (d, J = 8.5 Hz, 2H), 7.32 (d, J = 8.1 Hz, 2H), 7.21 (d, J = 8.1 Hz, 2H), 7.13 (s, 1H), 6.90 – 6.88 (m, 3H), 5.20 – 5.05 (m, J = 9.3, 8.7 Hz, 3H), 4.92 (d, J = 12.3 Hz, 1H), 4.65 (d, J = 12.3 Hz, 1H), 4.57 (d, J = 7.9 Hz, 1H), 4.28 (dd, J = 12.3, 4.7 Hz, 1H), 4.18 (dd, J = 12.3, 2.3 Hz, 1H), 3.94 (s, 3H), 3.93 (s, 3H), 3.71 – 3.66 (m, J = 9.8, 4.6, 2.4 Hz, 1H), 2.44 (s, 2H), 2.11 (s, 3H), 2.02 (s, 5H), 2.00 (s, 3H), 1.29 – 1.22 (m, 3H), 1.07 (dd, J = 7.4, 3.8 Hz, 11H).

^{13}C NMR (126 MHz, $CDCl_3$) δ 170.92, 170.49, 169.63, 169.56, 156.21, 152.73, 148.19, 141.09, 140.22, 138.18, 136.95, 135.32, 131.45, 130.90, 130.76, 130.06, 129.81, 129.53, 128.45, 127.56, 127.35, 127.10, 127.07, 120.23, 119.51, 115.23, 112.54, 102.35, 99.42, 73.07, 72.05, 71.53, 70.71, 68.65, 62.17, 56.48, 56.34, 20.96, 20.88, 20.81, 20.79, 17.68, 17.64, 13.13.

IR (thin film, cm^{-1}): 760.377, 912.952, 1039.383, 1215.354, 1367.571, 1502.551, 1604.208, 1755.575, 2220.236, 2946.285, 3022.332.

HRMS (ESI): Calculated for $C_{49}H_{57}NNaO_{13}Si$ [M+Na$^+$]: 918.3491; found: 918.3493.

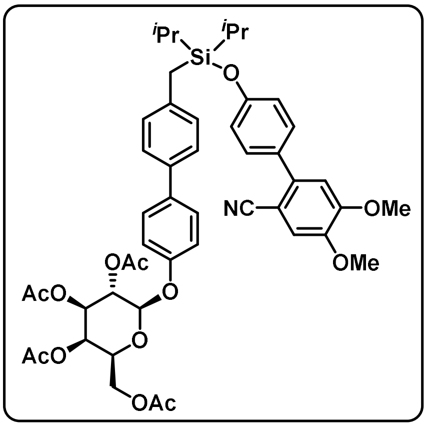

(2S,3R,4R,5S,6R)-2-(acetoxymethyl)-6-((4'-((((2'-cyano-4',5'-dimethoxy-[1,1'-biphenyl]-4-yl)oxy)diisopropylsilyl)methyl)-[1,1'-biphenyl]-4-yl)oxy)tetrahydro-2H-pyran-3,4,5-triyl triacetate (5ab): Compound 5ab was prepared under the **General Procedure 2.4.**

Eluent: petroleum ether/ethyl acetate (80:20, v/v).

Physical State: Yellow sticky liquid

Isolated Yield: 65% (57.3 mg); (*p*:others = 20:1).

R_f Value: 0.3 (30% ethyl acetate in petroleum ether).

^{1}H NMR (400 MHz, $CDCl_3$) δ 7.51 (d, J = 8.7 Hz, 2H), 7.41 (d, J = 5.5 Hz, 2H), 7.39 (d, J = 6.0 Hz, 2H), 7.19 (d, J = 8.1 Hz, 2H), 7.13 (s, 1H), 7.10 (d, J = 8.8 Hz, 2H), 6.90 – 6.87 (m, 3H), 5.80 (d, J = 3.6 Hz, 1H), 5.59 (dd, J = 10.8, 3.4 Hz, 1H), 5.56 – 5.52 (m, 1H), 5.30 (dd, J = 10.8, 3.6 Hz, 1H), 4.38 (t, J = 6.6 Hz, 1H), 4.17 – 4.11 (m, 1H), 4.10 – 4.04 (m, 1H), 3.95 (s, 3H), 3.93 (s, 3H), 2.43 (s, 2H), 2.17 (s, 3H), 2.09 (s, 3H), 2.04 (s, 3H), 1.94 (s, 3H), 1.31 – 1.21 (m, 2H), 1.07 (dd, J = 7.4, 3.3 Hz, 12H).
^{13}C NMR (101 MHz, $CDCl_3$) δ 170.65, 170.57, 170.43, 170.29, 156.23, 155.68, 152.73, 148.20, 140.23, 137.73, 136.75, 136.29, 131.45, 130.07, 129.52, 128.16, 126.87, 120.25, 119.52, 117.22, 115.23, 112.54, 102.36, 95.17, 68.13, 68.03, 67.78, 67.40, 61.70, 56.49, 56.35, 20.97, 20.90, 20.86, 20.81, 17.68, 17.64, 13.12.
IR (thin film, cm^{-1}): 758.498, 824.086, 917.029, 1070.620, 1136.953, 1218.917, 1371.710, 1462.925, 1502.590, 1604.344, 1750.002, 2219.977, 2868.345, 2944.205.
HRMS (ESI): Calculated for $C_{48}H_{55}KNO_{12}Si$ [M+K$^+$]: 904.3125; found: 904.3123.

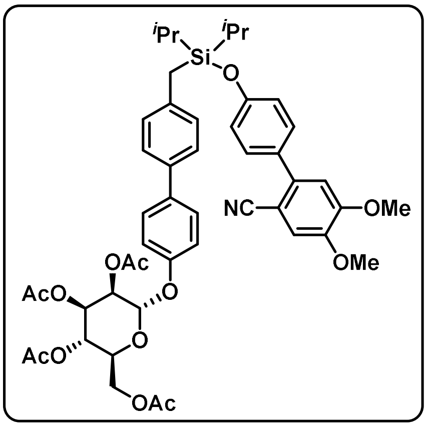

(2S,3S,4R,5R,6S)-2-(acetoxymethyl)-6-((4'-((((2'-cyano-4',5'-dimethoxy-[1,1'-biphenyl]-4-yl)oxy)diisopropylsilyl)methyl)-[1,1'-biphenyl]-4-yl)oxy)tetrahydro-2H-pyran-3,4,5-triyl triacetate (5ac): Compound 5ac was prepared under the **General Procedure 2.4.**
Eluent: petroleum ether/ethyl acetate (80:20, v/v).
Physical State: Yellow sticky liquid
Isolated Yield: 72% (63.5 mg); (*p*:others = 20:1).
R*f* Value: 0.3 (30% ethyl acetate in petroleum ether).
^{1}H NMR (400 MHz, $CDCl_3$) δ 7.51 (d, J = 8.7 Hz, 2H), 7.42 – 7.37 (m, J = 8.3, 7.0 Hz, 4H), 7.18 (d, J = 8.2 Hz, 2H), 7.14 – 7.11 (m, J = 7.5 Hz, 3H), 6.91 – 6.87 (m, 3H), 5.58 (dd, J = 10.0, 3.5 Hz, 1H), 5.55 (d, J = 1.6 Hz, 1H), 5.47 – 5.45 (m, J = 3.4, 1.8 Hz, 1H), 5.37 (t, J = 10.0 Hz, 1H), 4.29 (dd, J = 11.9, 5.0 Hz, 1H), 4.15 – 4.06 (m, 2H), 3.94 (s, 3H), 3.93 (s, 3H), 2.43 (s, 2H), 2.21 (s, 3H), 2.06 (s, 3H), 2.04 (s, 3H), 2.03 (s, 3H), 1.30 – 1.21 (m, 2H), 1.08 (d, J = 3.1 Hz, 6H), 1.06 (d, J = 3.1 Hz, 6H).
^{13}C NMR (101 MHz, $CDCl_3$) δ 170.80, 170.19, 169.99, 156.23, 154.97, 152.73, 148.19, 140.24, 137.74, 136.69, 136.27, 131.45, 130.07, 129.52, 128.14, 126.86, 120.25, 119.52,

116.96, 115.22, 112.54, 102.36, 96.09, 69.65, 69.38, 69.13, 66.20, 65.56, 62.35, 56.49, 56.35, 42.20, 30.34, 29.91, 23.56, 21.10, 20.91, 20.82, 17.68, 17.65, 14.30, 13.13, 11.32.
IR (thin film, cm^{-1}): 668.041, 755.599, 824.413, 915.690, 1039.423, 1135.990, 1216.410, 1369.757, 1462.864, 1502.281, 1604.619, 1752.003, 2220.720, 2947.516, 3022.824.
HRMS (ESI): Calculated for $C_{48}H_{55}NNaO_{13}Si$ [M+Na$^+$]: 904.3335; found: 904.3351.

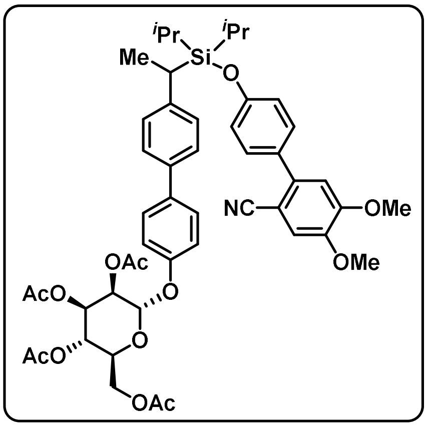

(2S,3S,4R,5R,6S)-2-(acetoxymethyl)-6-((4'-(1-(((2'-cyano-4',5'-dimethoxy-[1,1'-biphenyl]-4-yl)oxy)diisopropylsilyl)ethyl)-[1,1'-biphenyl]-4-yl)oxy)tetrahydro-2H-pyran-3,4,5-triyl triacetate (5tc): Compound 5tc was prepared under the **General Procedure 2.4.**
Eluent: petroleum ether/ethyl acetate (80:20, v/v).
Physical State: Yellow sticky liquid
Isolated Yield: 70% (62.7 mg); (*p*:others = 20:1).
R*f* Value: 0.3 (30% ethyl acetate in petroleum ether).
^{1}H NMR (400 MHz, $CDCl_3$) δ 7.53 (d, J = 8.7 Hz, 2H), 7.45 (d, J = 8.1 Hz, 2H), 7.38 (d, J = 8.5 Hz, 2H), 7.28 (d, J = 8.2 Hz, 2H), 7.15 – 7.11 (m, J = 4.3 Hz, 3H), 6.91 – 6.87 (m, 3H), 5.58 (dd, J = 10.1, 3.5 Hz, 1H), 5.55 (d, J = 1.4 Hz, 1H), 5.46 (dd, J = 3.3, 1.8 Hz, 1H), 5.37 (t, J = 10.0 Hz, 1H), 4.29 (dd, J = 12.0, 5.1 Hz, 1H), 4.15 – 4.06 (m, 2H), 3.94 (s, 3H), 3.93 (s, 3H), 2.66 (q, J = 7.5 Hz, 1H), 2.21 (s, 3H), 2.06 (s, 3H), 2.04 (s, 3H), 2.03 (s, 3H), 1.56 (d, J = 7.6 Hz, 3H), 1.41 – 1.31 (m, J = 14.9, 7.5 Hz, 2H), 1.09 (dd, J = 7.4, 4.0 Hz, 6H), 1.02 (d, J = 7.4 Hz, 3H), 0.97 (d, J = 7.4 Hz, 3H).
^{13}C NMR (101 MHz, $CDCl_3$) δ 170.79, 170.22, 170.18, 170.00, 156.41, 155.00, 152.73, 148.18, 144.01, 140.27, 137.08, 136.27, 131.29, 130.03, 128.71, 128.18, 126.75, 120.14, 119.52, 116.98, 115.24, 112.54, 102.35, 96.10, 69.66, 69.39, 69.14, 66.22, 62.37, 56.49, 56.34, 27.34, 22.89, 21.09, 20.91, 20.89, 18.20, 18.12, 17.94, 17.87, 16.41, 13.24, 13.11.
IR (thin film, cm^{-1}): 685.393, 752.861, 840.628, 914.962, 1039.126, 1135.661, 1216.429, 1369.522, 1462.622, 1502.065, 1604.080, 1751.349, 2220.614, 2869.799, 2950.577.
HRMS (ESI): Calculated for $C_{49}H_{57}NKO_{13}Si$ [M+K$^+$]: 934.3231; found: 934.3244.

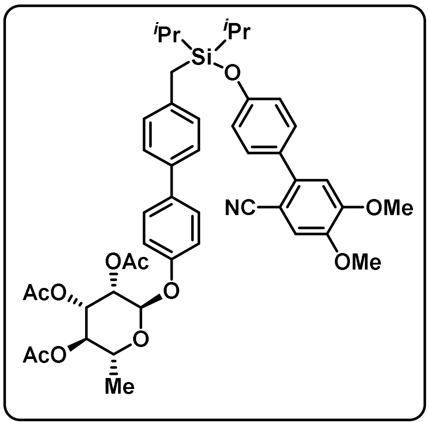

(2R,3S,4S,5R,6R)-2-((4'-((((2'-cyano-4',5'-dimethoxy-[1,1'-biphenyl]-4-yl)oxy)diisopropylsilyl)methyl)-[1,1'-biphenyl]-4-yl)oxy)-6-methyltetrahydro-2H-pyran-3,4,5-triyl triacetate (5ad): Compound 5ad was prepared under the **General Procedure 2.4.**

Eluent: petroleum ether/ethyl acetate (80:20, v/v).

Physical State: Yellow sticky liquid

Isolated Yield: 62% (51.08 mg); (*p*:others = 20:1).

R_f Value: 0.3 (30% ethyl acetate in petroleum ether).

^{1}H NMR (500 MHz, $CDCl_3$) δ 7.51 (d, J = 8.7 Hz, 2H), 7.41 (d, J = 8.2 Hz, 2H), 7.39 (d, J = 8.5 Hz, 2H), 7.18 (d, J = 8.1 Hz, 2H), 7.13 (d, J = 6.2 Hz, 2H), 7.10 (s, 1H), 6.89 (d, J = 2.5 Hz, 2H), 6.88 (s, 1H), 5.53 (dd, J = 10.1, 3.5 Hz, 1H), 5.48 (s, 1H), 5.46 – 5.43 (m, J = 3.4, 1.8 Hz, 1H), 5.17 (t, J = 10.0 Hz, 1H), 4.05 – 3.99 (m, 1H), 3.94 (s, 3H), 3.93 (s, 3H), 2.43 (s, 2H), 2.20 (s, 3H), 2.06 (s, 3H), 2.04 (s, 3H), 1.29 – 1.23 (m, 2H), 1.21 (d, J = 6.3 Hz, 3H), 1.07 (d, J = 4.5 Hz, 6H), 1.06 (d, J = 4.5 Hz, 6H).

^{13}C NMR (126 MHz, $CDCl_3$) δ 170.32, 170.29, 170.25, 156.22, 155.21, 152.70, 148.16, 140.23, 137.60, 136.76, 135.88, 131.41, 130.05, 129.48, 128.11, 126.84, 120.24, 119.53, 116.77, 115.19, 112.51, 102.32, 95.88, 71.19, 69.91, 69.13, 67.34, 56.47, 56.34, 21.13, 21.02, 20.97, 20.79, 17.68, 17.66, 17.64, 13.10.

IR (thin film, cm^{-1}): 756.650, 824.069, 914.094, 1038.678, 1135.538, 1215.704, 1370.614, 1501.721, 1604.426, 1749.671, 2219.977, 2932.874.

HRMS (ESI): Calculated for $C_{46}H_{53}NNaO_{11}Si$ [M+Na$^+$]: 846.3280; found: 846.3281.

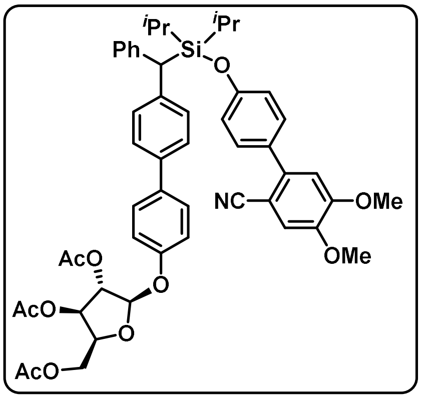

(2S,3R,4S,5R)-2-(acetoxymethyl)-5-((4'-((((2'-cyano-4',5'-dimethoxy-[1,1'-biphenyl]-4-yl)oxy)diisopropylsilyl)(phenyl)methyl)-[1,1'-biphenyl]-4-yl)oxy)tetrahydrofuran-3,4-diyl diacetate (5qe): Compound 5qe was prepared under the **General Procedure 2.4.**
Eluent: petroleum ether/ethyl acetate (80:20, v/v).
Physical State: Yellow sticky liquid
Isolated Yield: 59% (52.2 mg); (*p*:others = 20:1).
R_f Value: 0.3 (30% ethyl acetate in petroleum ether).
^{1}H NMR (400 MHz, $CDCl_3$) δ 7.55 – 7.50 (m, 6H), 7.46 (d, J = 8.3 Hz, 2H), 7.38 (d, J = 8.6 Hz, 2H), 7.30 (d, J = 7.4 Hz, 2H), 7.18 (d, J = 7.4 Hz, 1H), 7.13 (s, 1H), 7.10 (d, J = 8.8 Hz, 2H), 6.89 – 6.86 (m, 3H), 5.71 (dd, J = 11.2, 8.5 Hz, 2H), 5.10 – 5.03 (m, 1H), 4.99 (dd, J = 10.2, 3.6 Hz, 1H), 3.95 (s, 3H), 3.93 (s, 3H), 3.87 (dd, J = 11.0, 5.9 Hz, 1H), 3.82 (s, 1H), 3.74 (t, J = 11.0 Hz, 1H), 2.08 (s, 3H), 2.07 (s, 3H), 2.04 (s, 3H), 1.34 – 1.28 (m, J = 9.1, 5.2 Hz, 2H), 0.93 (dd, J = 10.8, 7.5 Hz, 12H).
^{13}C NMR (101 MHz, $CDCl_3$) δ 170.51, 170.37, 170.21, 156.10, 155.66, 152.72, 148.19, 142.09, 141.14, 140.20, 137.84, 135.97, 131.50, 130.08, 130.00, 129.68, 128.75, 128.26, 127.02, 125.94, 120.09, 119.53, 116.99, 115.20, 112.53, 102.35, 94.50, 70.90, 69.74, 69.41, 59.22, 56.49, 56.36, 42.47, 21.02, 20.93, 20.90, 18.18, 18.12, 17.86, 13.86.
IR (thin film, cm^{-1}): 752.611, 839.811, 916.491, 1047.489, 1136.822, 1220.201, 1369.111, 1463.841, 1503.050, 1603.631, 1753.291, 2219.935, 2868.902, 2947.938.
HRMS (ESI): Calculated for $C_{51}H_{55}NNaO_{11}Si$ [M+Na$^+$]: 908.3437; found: 908.3430.

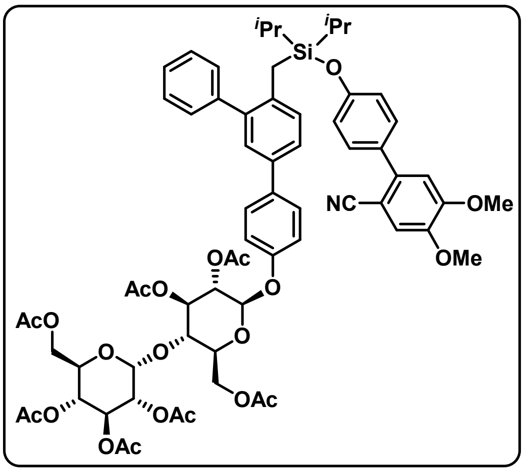

(2R,3R,4S,5R,6R)-2-(acetoxymethyl)-6-(((2S,3S,4R,5S,6R)-4,5-diacetoxy-2-(acetoxymethyl)-6-((4'-((((2'-cyano-4',5'-dimethoxy-[1,1'-biphenyl]-4-yl)oxy)diisopropylsilyl)methyl)-[1,1':3',1''-terphenyl]-4-yl)oxy)tetrahydro-2H-pyran-3-yl)oxy)tetrahydro-2H-pyran-3,4,5-triyl triacetate (5kf): Compound 5kf was prepared under the **General Procedure 2.4.**

Eluent: petroleum ether/ethyl acetate (60:40, v/v).

Physical State: Yellow sticky liquid

Isolated Yield: 72% (89.7 mg); (*p*:others = 20:1).

R_f Value: 0.3 (40% ethyl acetate in petroleum ether).

^{1}H NMR (500 MHz, $CDCl_3$) δ 7.54 (d, J = 8.6 Hz, 2H), 7.43 – 7.41 (m, 2H), 7.39 – 7.34 (m, J = 15.3, 8.2 Hz, 6H), 7.31 (d, J = 8.4 Hz, 2H), 7.13 (s, 1H), 7.01 (d, J = 8.6 Hz, 2H), 6.86 (s, 1H), 6.66 (d, J = 8.4 Hz, 2H), 5.44 (d, J = 3.7 Hz, 1H), 5.38 (t, J = 10.0 Hz, 1H), 5.33 (t, J = 8.6 Hz, 1H), 5.16 – 5.09 (m, 2H), 5.08 – 5.02 (m, J = 11.3, 5.4 Hz, 2H), 4.87 (dd, J = 10.5, 3.9 Hz, 1H), 4.48 (dd, 1H), 4.29 – 4.23 (m, 3H), 4.10 (t, 1H), 4.05 (dd, J = 11.0 Hz, 1H), 3.93 (d, J = 2.0 Hz, 6H), 2.51 (s, 2H), 2.10 (s, 3H), 2.09 (s, 3H), 2.05 (d, J = 2.2 Hz, 6H), 2.04 (s, 3H), 2.03 (s, 3H), 2.01 (s, 3H), 1.12 – 1.05 (m, J = 14.7, 7.3 Hz, 2H), 0.89 (d, J = 10.0 Hz, 6H), 0.86 (d, J = 7.7 Hz, 6H).

^{13}C NMR (101 MHz, $CDCl_3$) δ 170.77, 170.65, 170.44, 170.16, 169.87, 169.66, 156.22, 156.08, 152.69, 148.14, 142.24, 141.96, 140.24, 136.83, 136.30, 135.45, 131.21, 130.59, 129.94, 129.87, 128.98, 128.50, 128.17, 127.11, 125.73, 120.02, 119.51, 117.40, 115.21, 112.51, 102.29, 98.74, 95.85, 77.23, 75.56, 72.92, 72.50, 70.22, 69.52, 68.81, 68.23, 63.05, 61.79, 56.48, 56.32, 32.12, 22.89, 21.13, 20.97, 20.89, 20.85, 20.81, 20.79, 17.51, 17.40, 14.32, 13.23.

IR (thin film, cm^{-1}): 755.494, 839.576, 912.812, 1035.509, 1137.418, 1220.453, 1368.692, 1462.283, 1503.119, 1603.792, 1751.131, 2220.474, 2945.041.

HRMS (ESI): Calculated for $C_{66}H_{75}NNaO_{21}Si$ [M+K$^+$]: 1268.4493; found: 1268.4493.

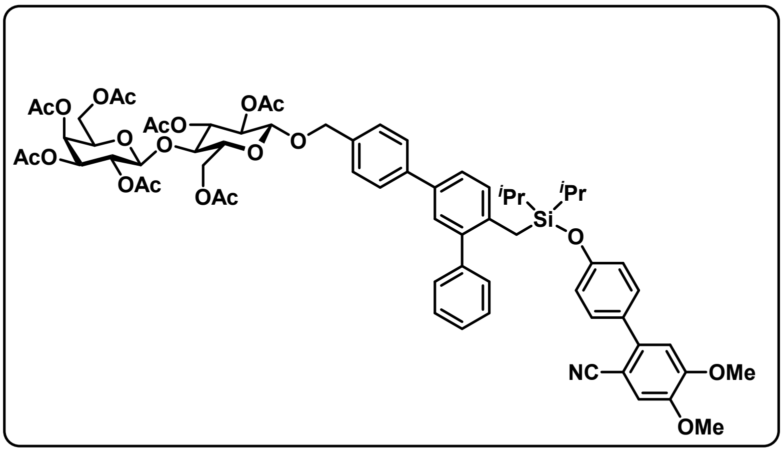

(2R,3S,4S,5R,6S)-2-(acetoxymethyl)-6-(((2R,3R,4S,5R,6R)-4,5-diacetoxy-2-(acetoxymethyl)-6-((4'-((((2'-cyano-4',5'-dimethoxy-[1,1'-biphenyl]-4-yl)oxy)diisopropylsilyl)methyl)-[1,1':3',1''-terphenyl]-4-yl)methoxy)tetrahydro-2H-pyran-3-yl)oxy)tetrahydro-2H-pyran-3,4,5-triyl triacetate (5kg): Compound 5kg was prepared under the **General Procedure 2.4.**

Eluent: petroleum ether/ethyl acetate (60:40, v/v).

Physical State: Yellow sticky liquid

Isolated Yield: 66% (83.2 mg); (*p*:others = 20:1).

R_f Value: 0.3 (40% ethyl acetate in petroleum ether).

^{1}H NMR (400 MHz, $CDCl_3$) δ 7.58 (d, J = 8.3 Hz, 1H), 7.47 (d, J = 8.0 Hz, 1H), 7.43 (d, J = 7.4 Hz, 2H), 7.41 – 7.36 (m, 3H), 7.35 (d, J = 6.4 Hz, 2H), 7.33 – 7.29 (m, 5H), 7.12 (s, 1H), 6.85 (s, 1H), 6.67 (d, J = 8.7 Hz, 2H), 5.34 (d, J = 3.6 Hz, 1H), 5.15 (t, J = 9.2 Hz, 1H), 5.12 – 5.06 (m, 1H), 4.99 – 4.92 (m, 2H), 4.87 (d, J = 12.3 Hz, 1H), 4.61 (d, J = 12.4 Hz, 1H), 4.53 (d, J = 7.9 Hz, 1H), 4.51 – 4.46 (m, 2H), 4.12 – 4.06 (m, 3H), 3.92 (s, 6H), 3.87 (t, J = 7.0 Hz, 1H), 3.82 (t, J = 9.4 Hz, 1H), 3.62 – 3.55 (m, 1H), 2.52 (s, 2H), 2.14 (s, 6H), 2.06 – 2.00 (m, 9H), 2.01 (s, 3H), 1.96 (s, 3H), 1.14 – 1.03 (m, 2H), 0.87 (t, J = 7.8 Hz, 12H).

^{13}C NMR (101 MHz, $CDCl_3$) δ 170.63, 170.57, 170.37, 170.29, 170.02, 169.85, 169.29, 156.06, 152.67, 148.12, 142.21, 141.96, 140.81, 140.23, 137.08, 135.85, 135.45, 131.20, 130.59, 129.93, 129.87, 129.20, 128.66, 128.49, 128.42, 128.23, 128.10, 127.95, 127.10, 125.92, 120.01, 119.50, 115.84, 115.20, 112.50, 102.27, 101.23, 99.14, 76.44, 72.98, 72.85, 71.84, 71.28, 71.18, 70.87, 70.64, 69.30, 66.81, 62.19, 61.01, 56.46, 56.31, 36.82, 32.11, 29.88, 29.54, 28.61, 24.86, 22.88, 21.09, 20.99, 20.91, 20.82, 20.70, 17.65, 17.55, 17.50, 17.38, 14.31, 13.23, 13.15.

IR (thin film, cm^{-1}): 753.995, 840.779, 912.040, 1051.926, 1137.059, 1216.472, 1368.559, 1461.787, 1502.766, 1603.389, 1749.531, 2219.960, 2943.826**.**

HRMS (ESI): Calculated for $C_{67}H_{78}NO_{21}Si$ [M+H$^+$]: 1260.4800; found: 1260.4800.

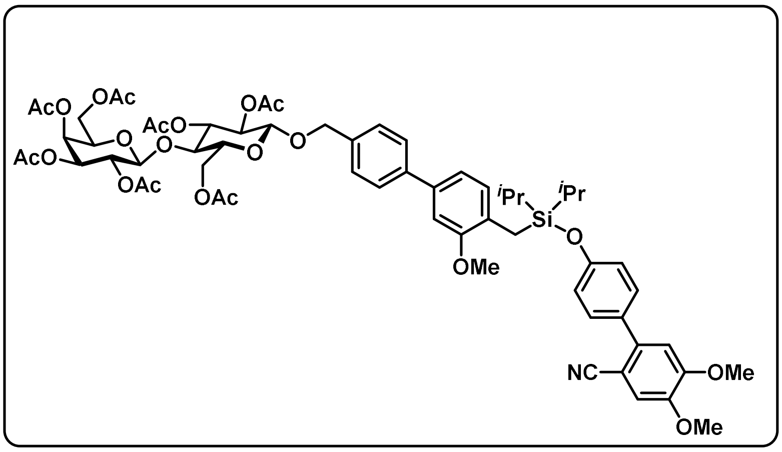

(2R,3S,4S,5R,6S)-2-(acetoxymethyl)-6-(((2R,3R,4S,5R,6R)-4,5-diacetoxy-2-(acetoxymethyl)-6-((4'-((((2'-cyano-4',5'-dimethoxy-[1,1'-biphenyl]-4-yl)oxy)diisopropylsilyl)methyl)-3'-methoxy-[1,1'-biphenyl]-4-yl)methoxy)tetrahydro-2H-pyran-3-yl)oxy)tetrahydro-2H-pyran-3,4,5-triyl triacetate (5cg): Compound 5cg was prepared under the **General Procedure 2.4.**

Eluent: petroleum ether/ethyl acetate (60:40, v/v).

Physical State: Yellow sticky liquid

Isolated Yield: 68% (82.5 mg); (*p*:others = 20:1).

R_f Value: 0.3 (40% ethyl acetate in petroleum ether).

^{1}H NMR (400 MHz, $CDCl_3$) δ 7.54 (d, J = 8.3 Hz, 1H), 7.45 (d, J = 8.3 Hz, 1H), 7.40 – 7.32 (m, 3H), 7.31 – 7.27 (m, 2H), 7.17 – 7.12 (m, 2H), 6.88 – 6.86 (m, 1H), 6.86 – 6.83 (m, 2H), 6.82 (t, J = 2.3 Hz, 1H), 5.34 (d, J = 3.4 Hz, 1H), 5.16 (t, J = 9.3 Hz, 1H), 5.09 (dd, 1H), 5.01 – 4.92 (m, 2H), 4.86 (t, 1H), 4.60 (t, 1H), 4.54 (dd, J = 8.0, 1.5 Hz, 1H), 4.49 (t, J = 7.8 Hz, 2H), 4.13 – 4.07 (m, 3H), 3.93 (s, 6H), 3.88 (t, 1H), 3.83 (d, J = 8.9 Hz, 1H), 3.79 (d, J = 16.0 Hz, 3H), 3.63 – 3.57 (m, 1H), 2.44 (d, J = 11.2 Hz, 2H), 2.14 – 2.12 (m, 6H), 2.05 – 2.02 (m, 9H), 2.01 (d, J = 9.5 Hz, 3H), 1.96 (s, 3H), 1.28 – 1.21 (m, 2H), 1.09 – 1.04 (m, 12H).

^{13}C NMR (101 MHz, CDCl3) δ 170.63, 170.57, 170.37, 170.28, 170.02, 169.85, 169.29, 157.12, 156.62, 156.43, 156.40, 152.70, 148.13, 141.48, 141.14, 140.35, 140.31, 138.55, 137.76, 135.40, 134.90, 132.85, 131.07, 131.04, 130.62, 129.88, 129.02, 128.41, 128.38, 127.90, 127.21, 126.99, 126.90, 124.48, 120.19, 120.12, 119.55, 119.25, 115.25, 115.22, 112.56, 112.54, 110.42, 108.90, 102.30, 102.27, 101.24, 99.23, 99.15, 76.45, 73.01, 72.88, 72.85, 71.88, 71.20, 70.89, 70.76, 70.67, 69.32, 66.83, 62.21, 61.02, 60.61, 56.48, 56.33, 55.21, 55.13, 36.83, 32.12, 29.85, 29.56, 28.63, 24.88, 24.03, 23.54, 22.89, 21.09, 21.00, 20.93, 20.90, 20.87, 20.83, 20.71, 17.60, 17.57, 15.12, 14.71, 14.39, 14.31, 13.55.

IR (thin film, cm^{-1}): 754.980, 911.906, 1050.080, 1137.294, 1216.519, 1368.691, 1463.068, 1502.398, 1603.829, 1749.360, 2219.960, 2943.116.

HRMS (ESI): Calculated for $C_{62}H_{76}NO_{22}Si$ [M+H$^+$]: 1214.4529; found: 1214.4528.

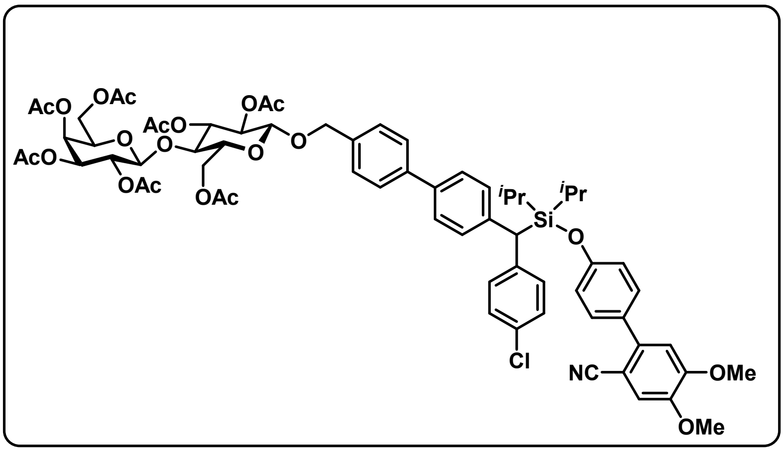

(2R,3S,4S,5R,6S)-2-(acetoxymethyl)-6-(((2R,3R,4S,5R,6R)-4,5-diacetoxy-2-(acetoxymethyl)-6-((4'-((4-chlorophenyl)(((2'-cyano-4',5'-dimethoxy-[1,1'-biphenyl]-4-yl)oxy)diisopropylsilyl)methyl)-[1,1'-biphenyl]-4-yl)methoxy)tetrahydro-2H-pyran-3-yl)oxy)tetrahydro-2H-pyran-3,4,5-triyl triacetate (5sg): Compound 5sg was prepared under the **General Procedure 2.4.**

Eluent: petroleum ether/ethyl acetate (60:40, v/v).

Physical State: Yellow sticky liquid

Isolated Yield: 59% (76.4 mg); (*p*:others = 20:1).

R_f Value: 0.3 (40% ethyl acetate in petroleum ether).

^{1}H NMR (400 MHz, $CDCl_3$) δ 7.55 (d, J = 8.3 Hz, 2H), 7.52 (s, 3H), 7.45 (d, J = 8.6 Hz, 2H), 7.39 (d, J = 8.6 Hz, 2H), 7.31 (m, 3H), 7.26 (d, J = 8.4 Hz, 2H), 7.13 (s, 1H), 6.90 – 6.86 (m, 3H), 5.34 (d, J = 4.7 Hz, 1H), 5.15 (t, J = 9.2 Hz, 1H), 5.09 (dd, J = 10.5, 7.8 Hz, 1H), 5.00 – 4.92 (m, 2H), 4.88 (d, J = 12.4 Hz, 1H), 4.62 (d, J = 12.5 Hz, 1H), 4.56 – 4.51 (m, 2H), 4.48 (d, J = 7.9 Hz, 1H), 4.15 – 4.05 (m, 3H), 3.95 (s, 3H), 3.93 (s, 3H), 3.87 (t, 1H), 3.85 – 3.81 (m, 1H), 3.80 (s, 1H), 3.64 – 3.55 (m, 1H), 2.14 (d, J = 1.7 Hz, 6H), 2.04 (t, J = 2.6 Hz, 9H), 2.01 (s, 3H), 1.96 (s, 3H), 1.29 – 1.23 (m, 2H), 0.97 – 0.92 (m, 12H).

^{13}C NMR (101 MHz, CDCl3) δ 170.62, 170.57, 170.37, 170.28, 170.01, 169.84, 169.29, 155.90, 152.76, 148.25, 141.11, 140.77, 140.10, 138.31, 135.64, 131.77, 131.72, 130.91, 130.15, 129.95, 128.83, 128.41, 127.39, 127.17, 120.01, 119.95, 119.48, 115.23, 112.53, 102.38, 101.24, 99.24, 77.43, 76.45, 73.01, 72.89, 71.89, 71.20, 70.90, 70.68, 69.33, 66.84, 62.21, 61.03, 60.60, 56.49, 56.36, 41.80, 36.83, 32.12, 29.85, 29.55, 28.64, 24.88, 24.03, 23.54, 22.89, 21.09, 21.00, 20.91, 20.82, 20.70, 18.15, 17.86, 17.84, 14.39, 14.31, 13.88, 13.81.

IR (thin film, cm^{-1}): 756.650, 916.699, 1056.150, 1215.366, 1369.257, 1503.066, 1604.232, 1750.763, 2219.960, 2945.484

HRMS (ESI): Calculated for $C_{67}H_{76}ClNNaO_{21}Si$ [M+Na$^+$]: 1316.4260; found: 1316.4266.

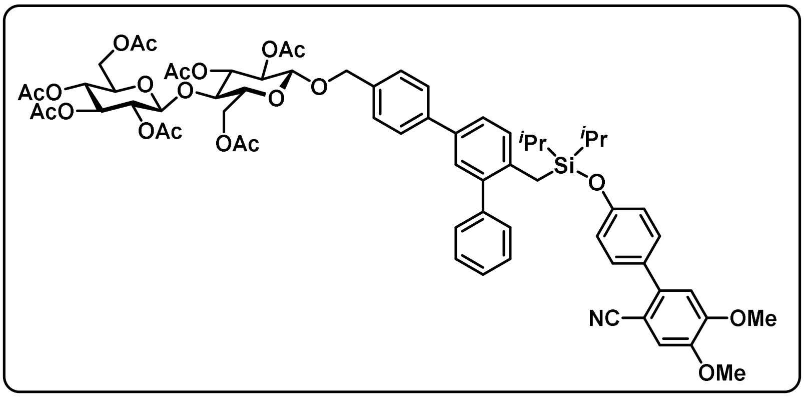

(2R,3R,4S,5R,6S)-2-(acetoxymethyl)-6-(((2R,3R,4S,5R,6R)-4,5-diacetoxy-2-(acetoxymethyl)-6-((4'-((((2'-cyano-4',5'-dimethoxy-[1,1'-biphenyl]-4-yl)oxy)diisopropylsilyl)methyl)-[1,1':3',1''-terphenyl]-4-yl)methoxy)tetrahydro-2H-pyran-3-yl)oxy)tetrahydro-2H-pyran-3,4,5-triyl triacetate (5kh): Compound 5kh was prepared under the **General Procedure 2.4.**

Eluent: petroleum ether/ethyl acetate (60:40, v/v).

Physical State: Yellow sticky liquid

Isolated Yield: 65% (81.9 mg); (*p*:others = 20:1).

R*f* Value: 0.3 (40% ethyl acetate in petroleum ether).

^{1}H NMR (400 MHz, $CDCl_3$) δ 7.57 (d, J = 8.0 Hz, 2H), 7.47 (dd, J = 7.9, 2.1 Hz, 1H), 7.43 (dd, J = 6.0, 1.6 Hz, 2H), 7.41 – 7.38 (m, 3H), 7.37 – 7.34 (m, 2H), 7.31 (d, J = 6.4 Hz, 2H), 7.29 (d, J = 6.1 Hz, 2H), 7.13 (s, 1H), 6.86 (s, 1H), 6.67 (d, J = 8.7 Hz, 2H), 5.14 (t, J = 9.3 Hz, 2H), 5.05 (t, J = 9.7 Hz, 1H), 5.00 – 4.91 (m, 2H), 4.87 (d, J = 11.6 Hz, 1H), 4.63 – 4.56 (m, 2H), 4.51 (dd, J = 7.9, 5.5 Hz, 2H), 4.36 (dd, J = 12.4, 4.4 Hz, 1H), 4.14 – 4.08 (m, 1H), 4.06 – 4.01 (m, 1H), 3.93 (s, 3H), 3.92 (s, 3H), 3.79 (t, J = 9.5 Hz, 1H), 3.68 – 3.62 (m, 1H), 3.60 – 3.53 (m, 1H), 2.52 (s, 2H), 2.14 (s, 3H), 2.07 (s, 3H), 2.02 (s, 3H), 2.00 (s, 9H), 1.98 (s, 3H), 1.12 – 1.05 (m, 2H), 0.88 (d, J = 6.9 Hz, 6H), 0.86 (d, J = 7.6 Hz, 6H).

^{13}C NMR 13C NMR (101 MHz, CDCl3) δ 170.73, 170.58, 170.45, 170.05, 169.83, 169.54, 169.26, 156.08, 152.68, 148.13, 142.23, 141.98, 140.83, 140.24, 137.09, 135.86, 135.44, 131.21, 130.61, 129.94, 129.88, 129.21, 128.50, 128.44, 127.11, 125.94, 120.08, 119.97, 119.51, 115.23, 115.20, 112.56, 112.48, 102.29, 100.96, 99.22, 73.15, 72.92, 72.70, 72.17, 72.14, 71.81, 71.75, 70.67, 67.99, 56.55, 56.40, 56.26, 21.12, 21.07, 20.89, 20.86, 20.83, 20.76, 20.73, 17.66, 17.51, 17.40, 13.25.

IR (thin film, cm^{-1}): 754.167, 841.166, 909.266, 1040.051, 1231.471, 1368.167, 1462.066, 1503.628, 1603.750, 1754.355, 2220.048, 2868.576, 2940.016.

HRMS (ESI): Calculated for $C_{67}H_{78}NO_{21}Si$ [M+H$^+$]: 1260.4829; found: 1260.4830.

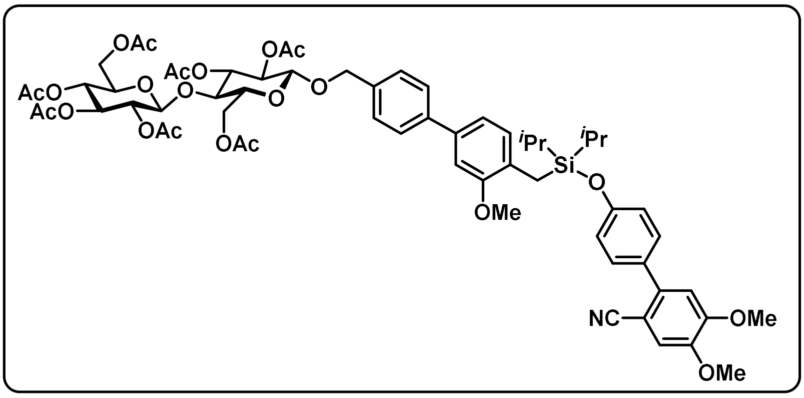

(2R,3R,4S,5R,6S)-2-(acetoxymethyl)-6-(((2R,3R,4S,5R,6R)-4,5-diacetoxy-2-(acetoxymethyl)-6-((4'-((((2'-cyano-4',5'-dimethoxy-[1,1'-biphenyl]-4-yl)oxy)diisopropylsilyl)methyl)-3'-methoxy-[1,1'-biphenyl]-4-yl)methoxy)tetrahydro-2H-pyran-3-yl)oxy)tetrahydro-2H-pyran-3,4,5-triyl triacetate (5ch): Compound 5ch was prepared under the **General Procedure 2.4.**

Eluent: petroleum ether/ethyl acetate (60:40, v/v).

Physical State: Yellow sticky liquid

Isolated Yield: 71% (86.2 mg); (*p*:others = 20:1).

R*f* Value: 0.3 (40% ethyl acetate in petroleum ether).

^{1}H NMR (400 MHz, $CDCl_3$) δ 7.54 (d, J = 8.3 Hz, 1H), 7.45 (d, J = 8.3 Hz, 1H), 7.37 – 7.32 (m, 3H), 7.31 – 7.26 (m, 2H), 7.17 – 7.12 (m, 2H), 6.86 (d, J = 2.1 Hz, 1H), 6.85 – 6.83 (m, 2H), 6.82 (t, J = 2.2 Hz, 1H), 5.13 (t, J = 9.3 Hz, 2H), 5.05 (t, J = 9.7 Hz, 1H), 5.00 – 4.93 (m, 1H), 4.91 (t, 1H), 4.85 (d, J = 12.5 Hz, 1H), 4.65 – 4.56 (m, 1H), 4.56 – 4.49 (m, 3H), 4.36 (dd, J = 12.5, 4.5 Hz, 1H), 4.13 – 4.09 (m, 2H), 4.03 (dd, J = 12.4, 2.3 Hz, 1H), 3.92 (s, 6H), 3.78 (d, J = 15.5 Hz, 3H), 3.68 – 3.61 (m, 1H), 3.61 – 3.54 (m, 1H), 2.44 (d, J = 11.2 Hz, 2H), 2.14 (d, J = 2.6 Hz, 3H), 2.07 (s, 3H), 2.03 (s, 3H), 2.02 (s, 3H), 2.00 (d, J = 1.8 Hz, 6H), 1.97 (s, 3H), 1.27 – 1.23 (m, 1H), 1.08 – 1.05 (m, 12H).

^{13}C NMR (101 MHz, $CDCl_3$) δ 170.71, 170.57, 170.44, 170.04, 169.81, 169.52, 169.25, 157.10, 156.60, 156.41, 156.39, 152.68, 148.10, 141.47, 141.12, 140.33, 140.29, 138.53, 137.74, 135.36, 134.87, 132.83, 131.05, 131.02, 130.61, 129.87, 129.01, 128.39, 127.88, 127.20, 126.97, 126.88, 124.46, 120.14, 119.54, 119.23, 115.20, 112.53, 110.40, 108.90, 102.27, 102.24, 100.95, 99.28, 99.20, 76.64, 73.13, 72.90, 72.69, 71.80, 70.76, 70.66, 67.98, 62.06, 61.74, 60.59, 56.47, 56.31, 21.23, 21.08, 20.90, 20.86, 20.83, 20.73, 17.57, 14.40, 14.36, 13.53.

IR (thin film, cm^{-1}): 754.167, 841.166, 909.266, 1040.051, 1231.471, 1368.167, 1462.066, 1503.628, 1603.750, 1754.355, 2220.048, 2868.576, 2940.016.

HRMS (ESI): Calculated for $C_{62}H_{75}NNaO_{22}Si$ [M+Na$^+$]: 1236.4415; found: 1236.4412.

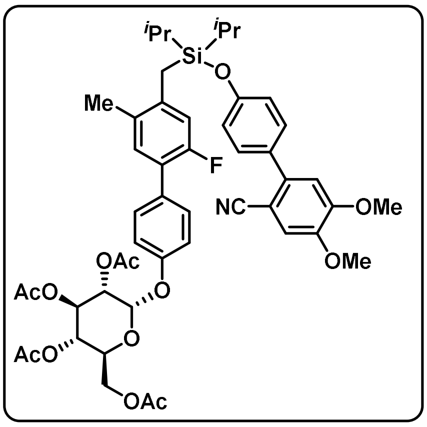

(2S,3S,4R,5S,6S)-2-(acetoxymethyl)-6-((4'-((((2'-cyano-4',5'-dimethoxy-[1,1'-biphenyl]-4-yl)oxy)diisopropylsilyl)methyl)-2'-fluoro-5'-methyl-[1,1'-biphenyl]-4-yl)oxy)tetrahydro-2H-pyran-3,4,5-triyl triacetate (5ni): Compound 5ni was prepared under the **General Procedure 2.4.**

Eluent: petroleum ether/ethyl acetate (80:20, v/v).

Physical State: Yellow sticky liquid

Isolated Yield: 73% (66.7 mg); (*p*:others = 20:1).

R_f Value: 0.3 (30% ethyl acetate in petroleum ether).

^{1}H NMR (500 MHz, $CDCl_3$) δ 7.47 (d, J = 10.2 Hz, 2H), 7.33 (d, J = 8.7 Hz, 2H), 7.15 – 7.08 (m, 4H), 6.92 (d, J = 11.9 Hz, 1H), 6.85 (s, 1H), 6.74 (d, J = 8.7 Hz, 2H), 5.77 (d, J = 3.6 Hz, 1H), 5.72 (t, 1H), 5.17 (t, J = 9.8 Hz, 1H), 5.06 (dd, J = 10.3, 3.7 Hz, 1H), 4.27 (dd, J = 12.5, 4.5 Hz, 1H), 4.17 – 4.12 (m, 1H), 4.08 – 4.03 (m, 1H), 3.92 (s, 6H), 2.36 (s, 2H), 2.29 (s, 3H), 2.07 (s, 3H), 2.06 (s, 3H), 2.05 (s, 6H), 1.34 – 1.28 (m, 2H), 1.11 (d, J = 7.5 Hz, 6H), 1.06 (d, J = 7.4 Hz, 6H).

^{13}C NMR (126 MHz, $CDCl_3$) δ 170.84, 170.43, 169.87, 155.86, 155.44, 152.68, 148.16, 140.15, 138.92, 131.91, 131.76, 131.44, 131.11, 130.35, 130.32, 130.00, 124.18, 119.93, 119.50, 116.61, 116.58, 116.40, 115.15, 112.49, 102.28, 94.37, 70.63, 70.29, 68.51, 68.24, 61.79, 56.48, 56.32, 32.14, 29.91, 29.58, 22.91, 20.95, 20.89, 20.87, 20.84, 19.93, 18.27, 17.78, 17.57, 14.34, 13.44.

^{19}F NMR (471 MHz, $CDCl_3$) δ -123.64.

IR (thin film, cm^{-1}): 681.463, 758.742, 839.491, 916.876, 1040.644, 1220.855, 1368.820, 1463.180, 1503.641, 1604.659, 1751.135, 2220.230, 2868.886, 2942.140.

HRMS (ESI): Calculated for $C_{49}H_{57}FNO_{13}Si$ [M+H$^+$]: 914.3578; found: 914.3578.

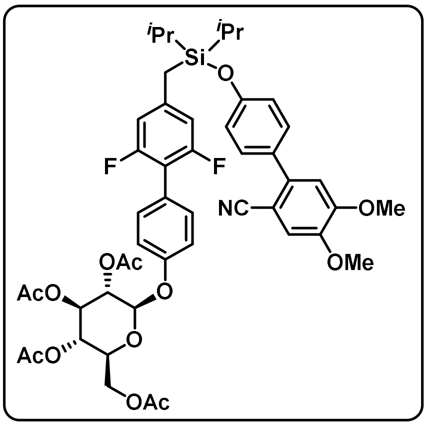

(2S,3S,4R,5S,6R)-2-(acetoxymethyl)-6-((4'-((((2'-cyano-4',5'-dimethoxy-[1,1'-biphenyl]-4-yl)oxy)diisopropylsilyl)methyl)-2',6'-difluoro-[1,1'-biphenyl]-4-yl)oxy)tetrahydro-2H-pyran-3,4,5-triyl triacetate (5oj): Compound 5oj was prepared under the **General Procedure 2.4.**

Eluent: petroleum ether/ethyl acetate (80:20, v/v).

Physical State: Yellow sticky liquid

Isolated Yield: 68% (62.4 mg); (*p*:others = 20:1).

R_f Value: 0.3 (30% ethyl acetate in petroleum ether).

^{1}H NMR (400 MHz, $CDCl_3$) δ 7.42 – 7.36 (m, 4H), 7.13 (s, 1H), 7.03 (d, J = 8.4 Hz, 2H), 6.91 – 6.87 (m, 3H), 6.73 (d, J = 8.3 Hz, 2H), 5.57 – 5.46 (m, J = 24.6, 14.8 Hz, 1H), 5.30 – 5.28 (m, J = 3.2 Hz, 1H), 5.20 – 5.12 (m, 2H), 5.09 (t, 1H), 4.32 – 4.25 (m, 2H), 3.93 (d, J = 3.0 Hz, 6H), 2.40 (s, 2H), 2.09 (d, J = 4.2 Hz, 3H), 2.07 (s, 3H), 2.05 (s, 3H), 2.04 (s, 3H), 1.30 – 1.25 (m, J = 12.4 Hz, 2H), 1.09 (d, J = 7.5 Hz, 12H).

^{13}C NMR (126 MHz, $CDCl_3$) δ 170.83, 170.47, 169.64, 156.62, 155.91, 152.79, 148.29, 140.11, 131.87, 131.81, 130.18, 128.27, 124.64, 120.12, 116.88, 115.28, 112.62, 112.27, 102.40, 99.16, 90.47, 73.00, 72.32, 71.43, 71.26, 70.06, 68.70, 68.58, 67.59, 62.18, 56.52, 56.35, 56.32, 20.96, 20.92, 20.89, 20.86, 20.83, 20.80, 17.67, 17.60, 13.21.

^{19}F NMR (471 MHz, $CDCl_3$) δ -115.97.

IR (thin film, cm^{-1}): 753.936, 1039.649, 1215.671, 1369.590, 1503.757, 1605.497, 1754.450, 2941.197, 3023.289.

HRMS (ESI): Calculated for $C_{48}H_{53}F_2NNaO_{13}Si$ [M+Na$^+$]: 940.3146; found: 940.3149.

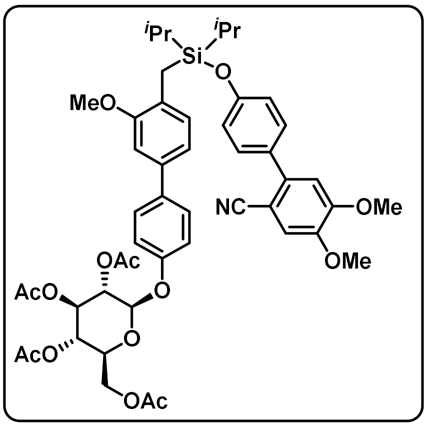

(2S,3S,4R,5S,6R)-2-(acetoxymethyl)-6-((4'-((((2'-cyano-4',5'-dimethoxy-[1,1'-biphenyl]-4-yl)oxy)diisopropylsilyl)methyl)-3'-methoxy-[1,1'-biphenyl]-4-yl)oxy)tetrahydro-2H-pyran-3,4,5-triyl triacetate (5cj): Compound 5cj was prepared under the **General Procedure 2.4.**

Eluent: petroleum ether/ethyl acetate (80:20, v/v).

Physical State: Yellow sticky liquid

Isolated Yield: 72% (65.6 mg); (*p*:others = 20:1).

R_f Value: 0.3 (30% ethyl acetate in petroleum ether).

^{1}H NMR (400 MHz, $CDCl_3$) δ 7.50 (d, J = 8.6 Hz, 1H), 7.38 – 7.33 (m, 3H), 7.13 (d, J = 6.7 Hz, 2H), 7.00 (dd, J = 12.4, 8.7 Hz, 3H), 6.86 (d, J = 7.6 Hz, 2H), 6.84 – 6.80 (m, 2H), 5.34 – 5.25 (m, 2H), 5.21 – 5.15 (m, 1H), 5.09 (t, J = 6.7 Hz, 1H), 4.33 – 4.27 (m, J = 12.1, 4.5 Hz, 1H), 4.21 – 4.14 (m, 1H), 3.93 (s, 6H), 3.89 – 3.85 (m, 1H), 3.78 (d, J = 6.5 Hz, 3H), 2.43 (d, J = 12.8 Hz, 2H), 2.09 – 2.04 (m, 12H), 1.28 – 1.20 (m, J = 19.0, 7.5 Hz, 2H), 1.09 – 1.05 (m, 12H).

^{13}C NMR (126 MHz, $CDCl_3$) δ 170.85, 170.50, 169.64, 169.57, 157.10, 156.40, 156.32, 155.93, 152.69, 148.11, 140.28, 138.28, 136.67, 132.65, 131.05, 130.60, 129.89, 129.88, 128.94, 128.26, 127.93, 127.81, 126.60, 124.21, 120.17, 120.09, 119.58, 119.03, 117.39, 117.33, 115.18, 112.50, 110.40, 108.72, 102.22, 99.45, 99.34, 72.98, 72.94, 72.25, 72.16, 71.39, 68.48, 62.11, 56.49, 56.35, 56.33, 55.22, 55.11, 20.94, 20.91, 20.85, 20.82, 17.61, 17.57, 15.00, 14.64, 13.53.

IR (thin film, cm^{-1}): 753.936, 1039.649, 1215.671, 1369.590, 1503.757, 1605.497, 1754.450, 2220.190, 2941.197, 3023.289.

HRMS (ESI): Calculated for $C_{49}H_{57}NNaO_{14}Si$ [M+Na$^+$]: 934.3441; found: 934.3439.

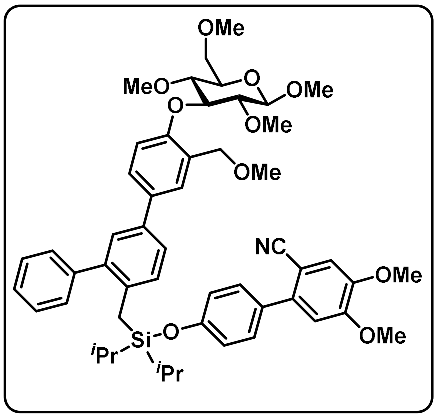

4'-((diisopropyl((3-(methoxymethyl)-4-(((2R,3R,4S,5R,6R)-2,3,5-trimethoxy-6-(methoxymethyl)tetrahydro-2H-pyran-4-yl)oxy)-[1,1':3',1''-terphenyl]-4'-yl)methyl)silyl)oxy)-4,5-dimethoxy-[1,1'-biphenyl]-2-carbonitrile (5kk): Compound 5kk was prepared under the **General Procedure 2.4.**

Eluent: petroleum ether/ethyl acetate (70:30, v/v).

Physical State: Yellow sticky liquid

Isolated Yield: 69% (61.4 mg); (*p*:others = 20:1).

R_f Value: 0.3 (30% ethyl acetate in petroleum ether).

^{1}H NMR (400 MHz, $CDCl_3$) δ 7.64 (s, 1H), 7.50 – 7.43 (m, 2H), 7.43 – 7.39 (m, 3H), 7.38 – 7.34 (m, 3H), 7.33 – 7.29 (m, 3H), 7.12 (s, 1H), 7.06 (d, J = 8.4 Hz, 1H), 6.86 (s, 1H), 6.66 (d, J = 8.3 Hz, 2H), 4.84 (d, J = 7.3 Hz, 1H), 4.65 – 4.51 (m, 2H), 3.96 – 3.90 (m, 9H), 3.67 (s, 4H), 3.64 (d, J = 3.5 Hz, 1H), 3.59 (d, J = 4.7 Hz, 1H), 3.56 (s, 3H), 3.41 (s, 3H), 3.38 (s, 3H), 3.33 – 3.23 (m, 3H), 2.50 (s, 2H), 1.05 - 1.11 (m, 2H), 0.87 (t, J = 7.8 Hz, 12H).

^{13}C NMR (101 MHz, $CDCl_3$) δ 156.12, 154.29, 152.69, 148.12, 142.38, 141.86, 140.29, 137.16, 135.38, 135.12, 131.17, 130.48, 129.94, 128.98, 128.47, 128.26, 127.15, 127.05, 125.78, 120.05, 119.54, 115.60, 115.22, 112.54, 102.29, 101.52, 86.81, 83.78, 79.35, 75.04, 71.31, 69.47, 61.18, 60.98, 60.67, 59.61, 58.61, 56.48, 56.34, 36.84, 24.89, 17.53, 17.42, 13.25.

IR (thin film, cm^{-1}): 756.241, 839.844, 915.368, 1095.002, 1266.850, 1387.805, 1503.144, 1603.620, 1732.899, 2219.991, 2931.587.

HRMS (ESI): Calculated for $C_{52}H_{64}NO_{10}Si$ [M+H$^+$]: 890.4294; found: 890.4292.

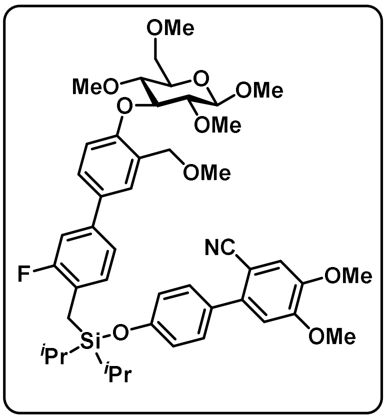

4'-((((3-fluoro-3'-(methoxymethyl)-4'-(((2R,3R,4S,5R,6R)-2,3,5-trimethoxy-6-(methoxymethyl)tetrahydro-2H-pyran-4-yl)oxy)-[1,1'-biphenyl]-4-yl)methyl)diisopropylsilyl)oxy)-4,5-dimethoxy-[1,1'-biphenyl]-2-carbonitrile (5uk): Compound 5uk was prepared under the **General Procedure 2.4.**

Eluent: petroleum ether/ethyl acetate (70:30, v/v).

Physical State: Yellow sticky liquid

Isolated Yield: 75% (62.3 mg); (*p*:others = 6:1).

R_f Value: 0.3 (30% ethyl acetate in petroleum ether).

^{1}H NMR (500 MHz, $CDCl_3$) δ 7.60 (d, J = 2.6 Hz, 1H), 7.43 (dd, J = 8.5, 2.4 Hz, 1H), 7.39 (d, J = 8.5 Hz, 2H), 7.21 (m, 1H), 7.17 (t, J = 8.2 Hz, 1H), 7.13 (s, 1H), 7.06 (d, J = 8.5 Hz, 1H), 7.02 (d, J = 11.1 Hz, 1H), 6.90 – 6.87 (m, 3H), 4.85 (d, J = 7.3 Hz, 1H), 4.62 (t, 1H), 4.57 – 4.54 (m, 1H), 3.94 – 3.92 (m, 6H), 3.67 (s, 6H), 3.65 (d, J = 6.7 Hz, 2H), 3.56 (s, 3H), 3.43 (s, 3H), 3.41 – 3.40 (m, 1H), 3.39 (s, 3H), 3.38 (d, J = 4.1 Hz, 1H), 3.28 – 3.25 (m, 2H), 2.39 (s, 2H), 1.31 – 1.26 (m, 2H), 1.09 – 1.04 (m, 12H).

^{13}C NMR (126 MHz, $CDCl_3$) δ 161.84, 159.91, 156.13, 154.48, 152.69, 148.13, 140.24, 139.25, 139.19, 135.00, 134.44, 131.40, 131.36, 128.34, 127.01, 126.97, 122.32, 121.21, 120.17, 120.14, 120.11, 119.54, 115.49, 115.19, 113.61, 113.42, 112.52, 102.30, 101.89, 101.41, 101.34, 86.79, 83.74, 79.32, 75.04, 71.29, 69.41, 61.19, 61.00, 60.69, 59.61, 58.67, 56.47, 56.45, 56.34, 17.55, 17.51, 13.35.

IR (thin film, cm^{-1}): 756.241, 839.844, 915.368, 1095.002, 1266.850, 1387.805, 1503.144, 1603.620, 1732.899, 2219.991, 2931.587.

HRMS (ESI): Calculated for $C_{46}H_{59}FNO_{10}Si$ [M+H$^+$]: 832.3834; found: 832.3833.

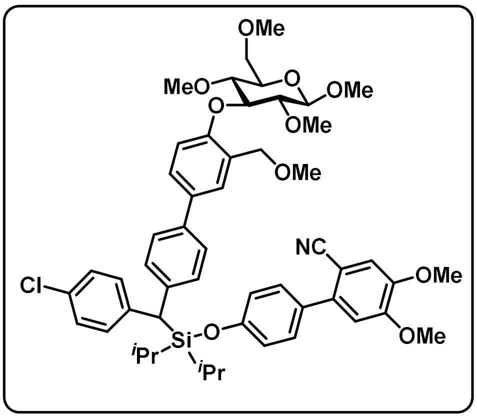

4'-((((4-chlorophenyl)(3'-(methoxymethyl)-4'-(((2R,3R,4S,5R,6R)-2,3,5-trimethoxy-6-(methoxymethyl)tetrahydro-2H-pyran-4-yl)oxy)-[1,1'-biphenyl]-4-yl)methyl)diisopropylsilyl)oxy)-4,5-dimethoxy-[1,1'-biphenyl]-2-carbonitrile (5sk): Compound 5sk was prepared under the **General Procedure 2.4.**

Eluent: petroleum ether/ethyl acetate (70:30, v/v).

Physical State: Yellow sticky liquid

Isolated Yield: 71% (65 mg); (*p*:others = 20:1).

R_f Value: 0.3 (30% ethyl acetate in petroleum ether).

^{1}H NMR (500 MHz, $CDCl_3$) δ 7.61 (d, J = 2.4 Hz, 1H), 7.49 (s, 2H), 7.46 – 7.43 (m, 4H), 7.41 – 7.38 (m, 3H), 7.24 (d, 2H), 7.13 (s, 1H), 7.06 (d, J = 8.6 Hz, 1H), 6.88 (m, 3H), 4.84 (d, J = 7.5 Hz, 1H), 4.64 – 4.54 (m, 2H), 3.95 (s, 3H), 3.93 (s, 3H), 3.78 (s, 1H), 3.69 (d, J = 7.6 Hz, 1H), 3.67 (s, 6H), 3.65 – 3.64 (m, 1H), 3.59 (d, J = 4.6 Hz, 1H), 3.56 (s, 3H), 3.42 (s, 3H), 3.38 (s, 3H), 3.30 (d, J = 7.6 Hz, 1H), 3.28 – 3.25 (m, 2H), 1.31 – 1.26 (m, 2H), 0.97 – 0.90 (m, 12H).

^{13}C NMR (126 MHz, $CDCl_3$) δ 155.93, 154.34, 152.72, 148.20, 140.89, 140.40, 140.14, 138.40, 135.34, 131.68, 130.89, 130.15, 129.85, 128.80, 128.23, 127.19, 127.08, 120.03, 119.99, 119.52, 115.49, 115.18, 112.52, 102.35, 101.46, 86.80, 83.76, 79.33, 75.04, 71.30, 69.47, 61.19, 61.00, 60.69, 59.63, 58.64, 56.49, 56.37, 41.73, 18.17, 17.88, 17.85, 13.89, 13.80.

IR (thin film, cm^{-1}): 753.053, 839.896, 918.804, 1094.263, 1265.397, 1387.281, 1463.494, 1502.750, 1603.562, 1732.937, 2219.576, 2929.343.

HRMS (ESI): Calculated for $C_{52}H_{63}ClNO_{10}Si$ [M+H$^+$]: 924.3904; found: 924.3895.

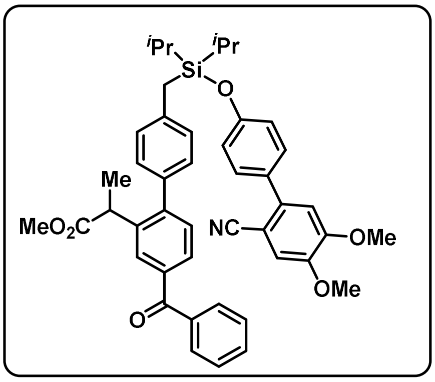

methyl 2-(4-benzoyl-4'-((((2'-cyano-4',5'-dimethoxy-[1,1'-biphenyl]-4-yl)oxy)diisopropylsilyl)methyl)-[1,1'-biphenyl]-2-yl)propanoate (5al): Compound 5al was prepared under the **General Procedure 2.4.**

Eluent: petroleum ether/ethyl acetate (95:5, v/v).

Physical State: Yellow solid

Isolated Yield: 57% (41.4 mg); (*p*:others = 15:1).

R_f Value: 0.6 (15% ethyl acetate in petroleum ether).

^{1}H NMR (400 MHz, $CDCl_3$) δ 7.84 – 7.80 (m, 3H), 7.69 (dd, J = 7.9, 1.8 Hz, 1H), 7.62 – 7.57 (m, 1H), 7.50 (d, J = 7.6 Hz, 2H), 7.48 – 7.44 (m, 1H), 7.39 (d, J = 8.7 Hz, 2H), 7.37 – 7.33 (m, 1H), 7.24 – 7.21 (m, 3H), 7.13 (s, 1H), 6.90 – 6.87 (m, 3H), 3.99 (q, J = 7.1 Hz, 1H), 3.95 (s, 3H), 3.93 (s, 3H), 3.62 (s, 3H), 2.47 (s, 2H), 1.37 (d, J = 7.1 Hz, 3H), 1.31 – 1.26 (m, 2H), 1.08 (dd, J = 7.4, 3.4 Hz, 12H).

^{13}C NMR (101 MHz, $CDCl_3$) δ 196.44, 175.15, 156.20, 152.78, 148.26, 146.15, 140.20, 139.15, 138.53, 137.83, 136.95, 136.60, 132.81, 132.70, 131.54, 130.64, 130.33, 130.18, 130.09, 130.05, 129.25, 129.10, 128.63, 128.58, 128.56, 128.51, 120.23, 119.50, 115.27, 112.56, 102.40, 56.51, 56.36, 52.30, 41.43, 21.05, 19.37, 17.67, 17.61, 13.16.

IR (thin film, cm^{-1}): 755.969, 908.993, 1031.263, 1116.169, 1215.691, 1266.879, 1355.965, 1503.737, 1605.085, 1682.199, 1750.346, 2220.236, 2929.565.

HRMS (ESI): Calculated for $C_{45}H_{48}NO_6Si$ [$M+H^+$]: 726.3258; found: 726.3257.

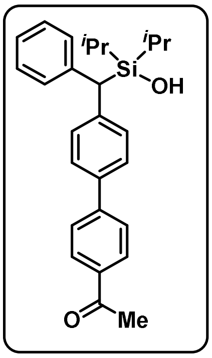

1-(4'-((hydroxydiisopropylsilyl)(phenyl)methyl)-[1,1'-biphenyl]-4-yl)ethan-1-one (6): Compound 6 was prepared under the **General Procedure 2.4.1.**

Eluent: petroleum ether/ethyl acetate (85:15, v/v).

Physical State: Light yellow liquid
Isolated Yield: 93% (38.7 mg).
R*f* Value: 0.4 (20% ethyl acetate in petroleum ether).
^{1}H NMR (400 MHz, $CDCl_3$) δ 8.00 (d, J = 8.5 Hz, 2H), 7.65 (d, J = 8.4 Hz, 2H), 7.55 (s, 4H), 7.48 (d, J = 8.2 Hz, 2H), 7.29 (t, J = 7.7 Hz, 2H), 7.17 (t, J = 7.3 Hz, 1H), 3.74 (s, 1H), 2.62 (s, 3H), 2.31 (brs, 1H), 1.07 – 0.99 (m, 2H), 0.98 – 0.89 (m, 12H).
^{13}C NMR (101 MHz, $CDCl_3$) δ 198.12, 145.70, 143.28, 142.39, 136.82, 135.64, 129.75, 129.27, 129.08, 128.75, 127.41, 126.99, 125.74, 42.81, 26.77, 17.85, 17.79, 17.70, 13.39, 13.36.
IR (thin film, cm^{-1}): 702.019, 843.978, 960.808, 1004.622, 1110.577, 1182.414, 1270.777, 1360.129, 1491.605, 1602.633, 1672.698, 1908.652, 2866.681, 2943.776, 3024.665, 3485.329.
HRMS (ESI): Calculated for $C_{27}H_{33}O_2Si$ [M+H$^+$]: 417.2243; found: 417.2244.

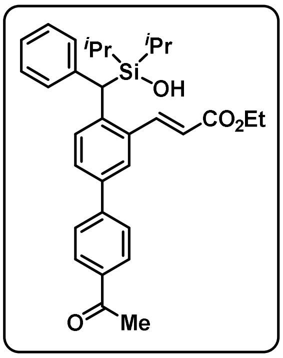

ethyl (E)-3-(4'-acetyl-4-((hydroxydiisopropylsilyl)(phenyl)methyl)-[1,1'-biphenyl]-3-yl)acrylate (7) (NMR spectrum shows both the regioisomer **7** and **8** are formed in 1:0.8 ratio**):** Compound 7 was prepared under the **General Procedure 2.4.2.a.**
Eluent: petroleum ether/ethyl acetate (85:15, v/v).
Physical State: Yellow sticky liquid
Isolated Yield: 72% (37 mg).
R*f* Value: 0.4 (20% ethyl acetate in petroleum ether).
^{1}H NMR (400 MHz, $CDCl_3$) δ 8.25 (d, J = 13.7 Hz, 1H), 8.21 (d, J = 13.7 Hz, 1H), 8.02 (d, J = 8.5 Hz, 1H), 7.98 (t, J = 8.1 Hz, 3H), 7.88 (d, J = 7.8 Hz, 1H), 7.73 (d, J = 2.1 Hz, 1H), 7.67 (d, J = 8.5 Hz, 1H), 7.65 – 7.60 (m, 3H), 7.53 (d, J = 8.5 Hz, 2H), 7.50 – 7.45 (m, 2H), 7.42 – 7.39 (m, 2H), 7.37 (dd, J = 7.7, 1.5 Hz, 1H), 7.27 (t, J = 7.7 Hz, 3H), 7.22 (t, 1H), 7.14 (t, J = 7.3 Hz, 1H), 6.36 (d, J = 15.7 Hz, 1H), 6.29 (d, J = 15.7 Hz, 1H), 4.28 (qd, J = 7.1, 5.7 Hz, 4H), 4.05 (s, 1H), 4.01 (s, 1H), 2.64 (s, 2H), 2.62 (s, 3H), 1.35 (td, J = 7.2, 3.8 Hz, 6H), 1.08 – 0.98 (m, 4H), 0.97 – 0.85 (m, 24H).
^{13}C NMR (101 MHz, $CDCl_3$) δ 198.04, 197.98, 167.15, 166.98, 145.59, 145.01, 143.12, 143.06, 142.32, 142.25, 141.65, 141.60, 137.75, 136.97, 136.12, 135.79, 134.88, 134.24, 131.52, 130.94, 130.10, 129.60, 129.18, 129.11, 128.94, 128.63, 127.79, 127.61, 127.16, 127.05, 126.59, 126.35, 125.81, 121.17, 120.63, 60.82, 60.74, 37.69, 37.65, 26.88, 26.84, 17.90, 17.88, 17.85, 17.81, 17.77, 17.63, 14.52, 13.56, 13.53, 13.41.

IR (thin film, cm^{-1}): 755.316, 843.896, 909.607, 1033.192, 1180.222, 1269.738, 1313.600, 1366.715, 1464.461, 1604.308, 1680.502, 2943.555

HRMS (ESI): Calculated for $C_{32}H_{39}O_4Si$ [M+H$^+$]: 515.2529; found: 515.2528.

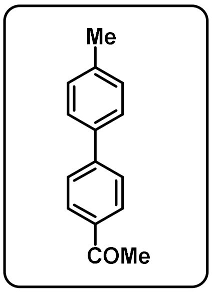

1-(4'-methyl-[1,1'-biphenyl]-4-yl)ethan-1-one (9): Compound 9 was prepared under the **General Procedure 2.4.1.**

Eluent: petroleum ether/ethyl acetate (95:5, v/v).

Physical State: Yellow solid

Isolated Yield: 95% (20 mg)

^{1}H NMR (400 MHz, $CDCl_3$) δ 8.02 (d, J = 8.5 Hz, 2H), 7.67 (d, J = 8.4 Hz, 2H), 7.54 (d, J = 8.2 Hz, 2H), 7.28 (d, J = 8.0 Hz, 2H), 2.63 (s, 3H), 2.42 (s, 3H).

^{13}C NMR (126 MHz, $CDCl_3$) δ 197.93, 145.86, 138.39, 137.08, 135.74, 129.85, 129.07, 127.25, 127.09, 26.78, 21.32.

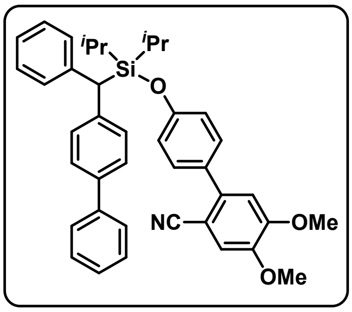

4'-((([1,1'-biphenyl]-4-yl(phenyl)methyl)diisopropylsilyl)oxy)-4,5-dimethoxy-[1,1'-biphenyl]-2-carbonitrile (3qz'): Compound 3qz' was prepared under the **General Procedure 2.4.**

Eluent: petroleum ether/ethyl acetate (90:10, v/v).

Physical State: White solid

Isolated Yield: 70% (42.8 mg); (*p*:others = 15:1).

R_f Value: 0.5 (20% ethyl acetate in petroleum ether).

^{1}H NMR (500 MHz, CDCl3) δ 7.62 – 7.59 (m, 4H), 7.57 (t, 4H), 7.45 – 7.41 (m, 4H), 7.33 (t, J = 7.8 Hz, 3H), 7.21 (t, J = 7.4 Hz, 1H), 7.15 (s, 1H), 6.92 (d, J = 8.5 Hz, 2H), 6.91 (s, 1H), 3.95 (s, 3H), 3.93 (s, 3H), 3.88 (s, 1H), 1.39 – 1.30 (m, 2H), 1.01 – 0.94 (m, 12H).

^{13}C NMR (126 MHz, CDCl3) δ 156.03, 152.62, 148.09, 142.00, 141.31, 141.00, 140.09, 138.48, 131.43, 130.02, 129.91, 129.62, 128.84, 128.69, 127.22, 127.13, 127.01, 125.88,

120.02, 119.48, 115.08, 112.42, 102.23, 56.38, 56.25, 42.41, 18.13, 18.07, 17.81, 13.80, 13.78.

HRMS (ESI): Calculated for $C_{40}H_{41}NNaO_3Si$ [M+Na$^+$]: 634.2748; found: 634.2744.

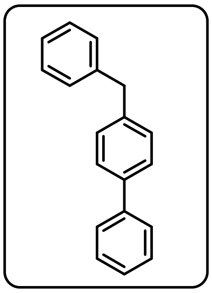

4-benzyl-1,1'-biphenyl (10): Compound 10 was prepared under the **General Procedure 2.4.1.**

Eluent: petroleum ether/ethyl acetate (96:4, v/v).

Physical State: White solid

Isolated Yield: 94% (22.9 mg).

^{1}H NMR (500 MHz, $CDCl_3$) δ 7.63 (d, J = 8.1 Hz, 2H), 7.58 (d, J = 8.2 Hz, 2H), 7.48 (t, J = 7.7 Hz, 2H), 7.41 – 7.34 (m, 3H), 7.32 (d, J = 8.2 Hz, 2H), 7.30 – 7.27 (m, 3H), 4.08 (s, 2H).

^{13}C NMR (126 MHz, $CDCl_3$) δ 141.21, 141.19, 140.45, 139.23, 129.52, 129.17, 128.92, 128.72, 127.41, 127.28, 127.21, 126.35, 41.78.

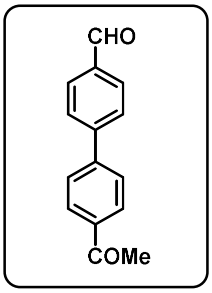

4'-acetyl-[1,1'-biphenyl]-4-carbaldehyde (11): Compound 11 was prepared under the **General Procedure 2.4.2.c.**

Eluent: petroleum ether/ethyl acetate (90:10, v/v).

Physical State: Yellow liquid

Isolated Yield: 79% (18 mg).

^{1}H NMR (400 MHz, $CDCl_3$) δ 10.07 (s, 1H), 8.06 (d, J = 8.1 Hz, 2H), 7.98 (d, J = 7.9 Hz, 2H), 7.78 (d, J = 8.0 Hz, 2H), 7.72 (d, J = 8.2 Hz, 2H), 2.65 (s, 3H).

^{13}C NMR (101 MHz, $CDCl_3$) δ 197.76, 191.95, 145.91, 144.33, 136.93, 136.02, 130.52, 129.23, 128.10, 127.75, 26.90.

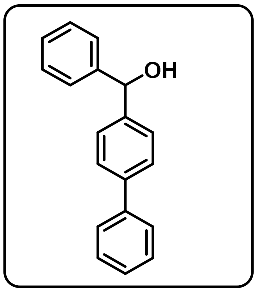

[1,1'-biphenyl]-4-yl(phenyl)methanol (12): Compound 12 was prepared under the **General Procedure 2.4.2.b.**
Eluent: petroleum ether/ethyl acetate (90:10, v/v).
Physical State: Colorless liquid
Isolated Yield: 89% (23.17 mg).
^{1}H NMR (400 MHz, $CDCl_3$) δ 7.59 – 7.54 (m, 4H), 7.48 – 7.43 (m, 3H), 7.43 – 7.41 (m, 3H), 7.39 – 7.35 (m, 2H), 7.34 (t, J = 2.1 Hz, 1H), 7.33 – 7.26 (m, 2H), 5.90 (s, 1H).
^{13}C NMR (126 MHz, $CDCl_3$) δ 143.97, 143.04, 141.00, 140.73, 128.97, 128.78, 127.88, 127.51, 127.48, 127.30, 127.19, 126.76, 76.28.

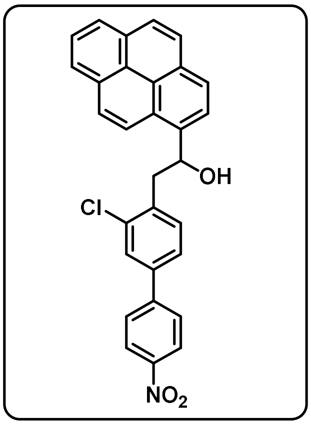

2-(3-chloro-4'-nitro-[1,1'-biphenyl]-4-yl)-1-(pyren-1-yl)ethan-1-ol (14): Compound 14 was prepared under the **General Procedure 2.4.2.d.**
Eluent: petroleum ether/ethyl acetate (70:30, v/v).
Physical State: Yellow solid
Isolated Yield: 82% (39.2 mg).
^{1}H NMR (400 MHz, $CDCl_3$) δ 8.47 (d, J = 9.3 Hz, 1H), 8.33 – 8.24 (m, 4H), 8.23 – 8.18 (m, 2H), 8.12 (d, J = 9.2 Hz, 1H), 8.08 (s, 2H), 8.02 (t, J = 7.6 Hz, 1H), 7.69 (s, 1H), 7.66 (d, J = 8.8 Hz, 2H), 7.37 (s, 2H), 6.16 (dd, J = 9.1, 4.3 Hz, 1H), 3.57 (dd, J = 13.8, 4.5 Hz, 1H), 3.40 (dd, J = 13.8, 9.1 Hz, 1H).
^{13}C NMR (101 MHz, $CDCl_3$) δ 147.58, 146.04, 139.11, 137.40, 137.07, 135.42, 133.29, 131.62, 131.13, 130.86, 130.78, 128.56, 128.04, 127.91, 127.86, 127.70, 127.67, 126.26, 125.81, 125.62, 125.39, 125.36, 125.12, 125.04, 124.42, 123.56, 122.54, 70.48, 43.49.

IR (thin film, cm^{-1}): 667.976, 753.826, 1046.388, 1216.093, 1345.612, 1519.036, 1733.434, 2931.637, 3020.990.
HRMS (ESI): Calculated for $C_{30}H_{21}ClNO_3$ [M+H$^+$]: 478.1125; found: 478.1129.

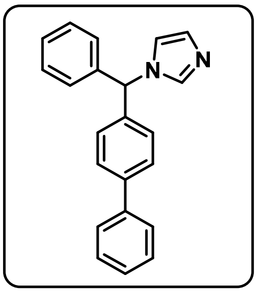

1-([1,1'-biphenyl]-4-yl(phenyl)methyl)-1H-imidazole (15): Compound 15 was prepared under the **General Procedure 2.4.2.e.**
Eluent: petroleum ether/ethyl acetate (80:20, v/v).
Physical State: White solid
Isolated Yield: 75% (23.3 mg).
^{1}H NMR (400 MHz, $CDCl_3$) δ 7.60 – 7.56 (m, 4H), 7.48 – 7.42 (m, 3H), 7.39 – 7.33 (m, 4H), 7.20 – 7.13 (m, 4H), 7.12 (t, J = 1.2 Hz, 1H), 6.89 (t, J = 1.3 Hz, 1H), 6.56 (s, 1H).
^{13}C NMR (101 MHz, $CDCl_3$) δ 141.56, 140.46, 139.31, 138.30, 137.64, 129.65, 129.15, 129.08, 128.72, 128.67, 128.30, 127.86, 127.77, 127.31, 119.59, 65.04.

2.7. Mechanistic studies

2.7.1. Kinetic experiments:

Order determination studies were performed with respect to substrate **1a**, and aryl iodide **2m**. Amount of product in each reaction was measured by HPLC using acetophenone as the internal standard and concentration of the product was plotted against time (in minutes). As *para*-scaffold and aryl iodide were involved in this reaction, we can assume the rate of the reaction is only dependent on the concentration of scaffold and aryl iodide.

So, Rate = k. [scaffold]^x [aryl iodide]^y ……….(1)

Order determination with respect to substrate **1a**

Procedure: Several oven-dried screw cap reaction tubes were charged with a magnetic stir-bars, in every reaction tubes: $Pd(OAc)_2$ (10 mol%), Fmoc-Gly-OH (20 mol%), Ag_2SO_4 (2.0 equiv), $Cu_2Cr_2O_5$ (2.0 equiv), LiOAc.2H_2O (0.5 equiv), substrate **1a** [0.05 mmol] and aryl iodide **2m** (0.05 mmol). Then 1 mL of 1,1,1,3,3,3-hexafluoro-2-propanol (HFIP) was added. The reaction mixture was stirred vigorously on a preheated oil bath at 80 ˚C along. The reactions were monitored and removed the reactions for yield analysis one by one after

the interval of 15 min, 30 min, 45 min, 60 min, 90 min and the processes have been monitored up to 720 min.

Same set of experiments were repeated with the 0.025 mmol of substrate **1a** while other parameters remain same and the formation of product was monitored.

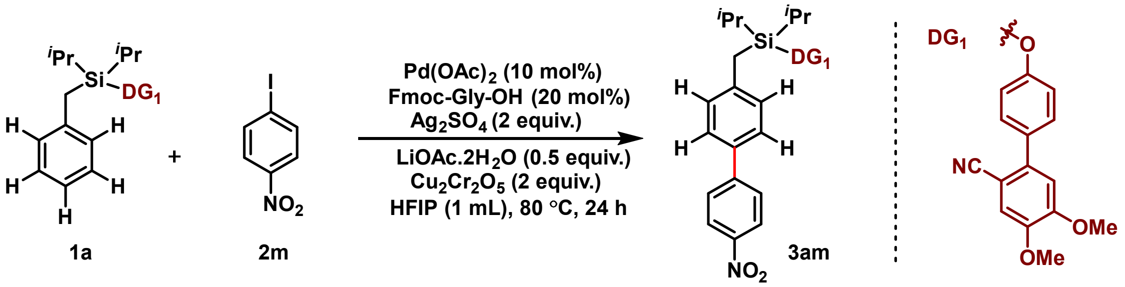

Run	**1a** substrate (mmol)	aryl iodide (**2m**) (mmol)	$Pd(OAc)_2$ (mmol)	Fmoc-Gly-OH (mmol)	Ag_2SO_4 (mmol)	$Cu_2Cr_2O_5$ (mmol)	$LiOAc.2H_2O$ (mmol)	HFIP (mL)
1	0.05	0.05	0.005	0.01	0.1	0.1	0.025	1
2	0.025	0.05	0.005	0.01	0.1	0.1	0.025	1

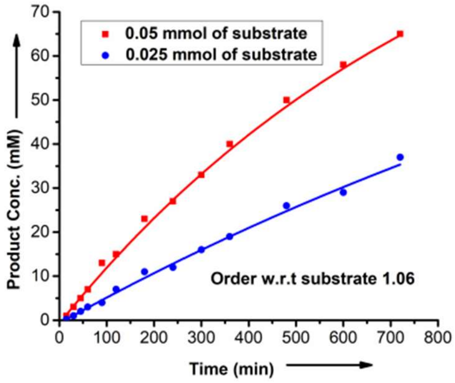

Supplementary Figure 11. Product formation plot in run 1 and run 2

From the equation (1) we got, Rate = k. [substrate]^x [aryl iodide]^y

For run 1, initial rate = Rate 1

So, Rate 1 = k. [substrate]^x [aryl iodide]^y

or, (15.371 – 12.524) / (129.761 – 105.083) = k . [0.05]^x [0.05]^y

or, 0.115 = k . [0.05]^x [0.05]^y ....(2)

For run 2, initial rate = Rate 2

So, Rate 2 = k. [substrate]^x [aryl iodide]^y

or, (6.844 – 5.496) / (129.761 – 105.083) = k . [0.025]^x [0.05]^y

or, 0.055 = k . [0.025]^x [0.05]^y ....(3)

Hence from equation (2) and (3)

We get, [Rate 1/ Rate 2] = [0.05/ 0.025]^x

or, x = [log (Rate 1) – log (Rate 2)] / [log (0.05) – log (0.025)]

or, x = [log (0.115) – log (0.055)] / [log (0.05) – log (0.025)]

or, x = 1.06

So, order with respect to scaffold (**1a**) is ~ 1

Order determination with respect to aryl iodide **2m**

Procedure: Several oven-dried screw cap reaction tubes were charged with a magnetic stir-bars, in every reaction tubes: $Pd(OAc)_2$ (10 mol%), Fmoc-Gly-OH (20 mol%), Ag_2SO_4 (2.0 equiv), $Cu_2Cr_2O_5$ (2.0 equiv), $LiOAc.2H_2O$ (0.5 equiv), substrate **1a** [0.05 mmol] and aryl iodide **2m** (0.05 mmol). Then 1 mL of 1,1,1,3,3,3-hexafluoro-2-propanol (HFIP) was added. The reaction mixture was stirred vigorously on a preheated oil bath at 80 ˚C along. The reactions were monitored and removed the reactions for yield analysis one by one after the interval of 15 min, 30 min, 45 min, 60 min, 90 min and the processes have been monitored up to 720 min.

Same set of experiments were repeated with the 0.025 mmol of substrate **2m** while other parameters remain same and the formation of product was monitored.

Run	**1a** substrate (mmol)	aryl iodide (**2m**) (mmol)	$Pd(OAc)_2$ (mmol)	Fmoc-Gly-OH (mmol)	Ag_2SO_4 (mmol)	$Cu_2Cr_2O_5$ (mmol)	$LiOAc.2H_2O$ (mmol)	HFIP (mL)
1	0.05	0.05	0.005	0.01	0.1	0.1	0.025	1
3	0.05	0.025	0.005	0.01	0.1	0.1	0.025	1

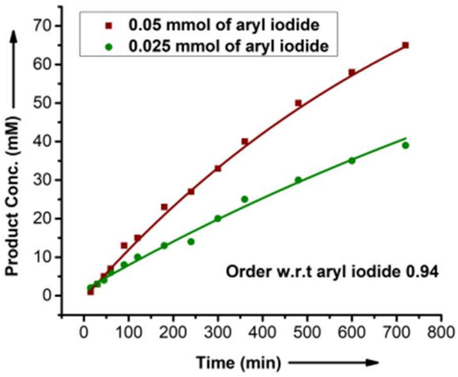

Supplementary Figure 12. Product formation plot in run 1 and run 3

From the equation (1) we got, Rate = k. $[\text{substrate}]^x$ $[\text{aryl iodide}]^y$

For run 1, initial rate = Rate 1

So, Rate 1 = k. $[\text{substrate}]^x$ $[\text{aryl iodide}]^y$

or, (15.411 – 12.496) / (129.879 – 105.115) = k . $[0.05]^x$ $[0.05]^y$

or, 0.116 = k . $[0.05]^x$ $[0.05]^y$ ……………(4)

For run 3, initial rate = Rate 3

So, Rate 3 = k. $[\text{substrate}]^x$ $[\text{aryl iodide}]^y$

or, (9.783 – 8.261) / (129.879 – 105.115) = k . $[0.05]^x$ $[0.025]^y$

or, 0.061 = k . $[0.05]^x$ $[0.025]^y$ ……………(5)

Hence from equation (4) and (5)

We get, [Rate 1/ Rate 3] = $[0.05/ 0.025]^y$

or, y = [log (Rate 1) – log (Rate 3)] / [log (0.05) – log (0.025)]

or, y = [log (0.116) – log (0.061)] / [log (0.05) – log (0.025)]

or, y = 0.94

So, order with respect to aryl iodide (**2m**) is ~ 1

***k_H/k_D* determination:**

Kinetic studies were conducted to obtain the value of kinetic isotope effect (KIE) of the *para*-arylation reaction. In this mechanistic study the ratio of k_H/k_D was determined from initial slope method. The arylation reaction with the substrate **1a** and aryl iodide **2m** was monitored by measuring the amount of the product formation (yield %) through HPLC analysis using acetophenone as internal standard. Then concentration of the product was plotted against time (in minutes). The same experimentation in same scale was done using **1a-d7** as substrate. The initial rate of both the experiments was compared to calculate the value of k_H/k_D.

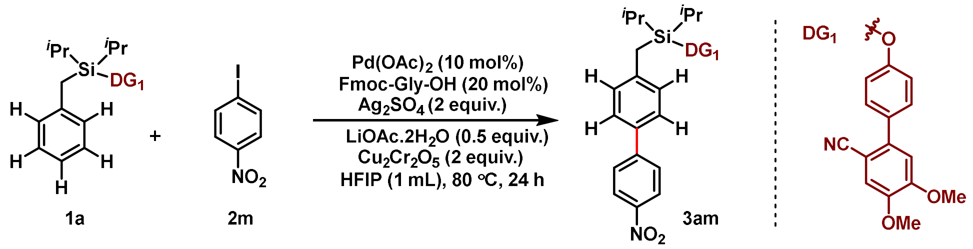

Several oven-dried screw cap reaction tubes were charged with a magnetic stir-bars, in every reaction tubes: $Pd(OAc)_2$ (10 mol%), Fmoc-Gly-OH (20 mol%), Ag_2SO_4 (2.0 equiv), $Cu_2Cr_2O_5$ (2.0 equiv), LiOAc.2H_2O (0.5 equiv), substrate **1a** [0.05 mmol] and aryl iodide **2m** (0.05 mmol). Then 1 mL of 1,1,1,3,3,3-hexafluoro-2-propanol (HFIP) was added. The reaction mixture was stirred vigorously on a preheated oil bath at 80 ˚C along. The reactions were monitored and removed the reactions for yield analysis one by one after the interval of 15 min, 30 min, 45 min, 60 min, 90 min and the processes have been monitored up to 720 min.

Same set of experiments were repeated with the deuterated substrates **1a-d7** and the formation of product was monitored.

Run	**1a** substrate (mmol)	aryl iodide (**2m**) (mmol)	$Pd(OAc)_2$ (mmol)	Fmoc-Gly-OH (mmol)	Ag_2SO_4 (mmol)	$Cu_2Cr_2O_5$ (mmol)	$LiOAc.2H_2O$ (mmol)
1	0.05	0.05	0.005	0.01	0.1	0.1	0.025

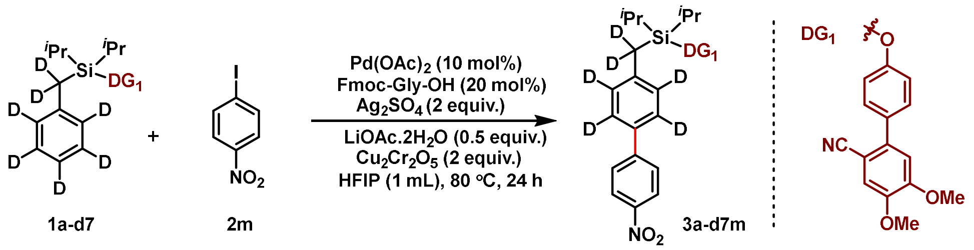

Run	Deuterated substrate **1a-d7** (mmol)	aryl iodide (**2m**) (mmol)	$Pd(OAc)_2$ (mmol)	Fmoc-Gly-OH (mmol)	Ag_2SO_4 (mmol)	$Cu_2Cr_2O_5$ (mmol)	$LiOAc.2H_2O$ (mmol)
4	0.05	0.05	0.005	0.01	0.1	0.1	0.025

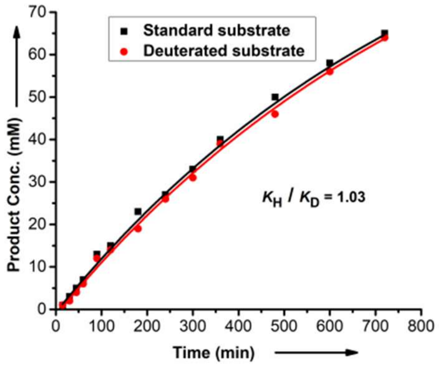

Supplementary Figure 13. Determination of kinetic isotope effect

From the equation (1) we got, Rate = k. [substrate]^x [aryl iodide]^y

For run 1, initial rate = Rate 1

So, Rate 1 = k_H. [substrate]^x [aryl iodide]^y

or, $(16.344 - 12.529) / (138.319 - 105.686) = k_H \, . \, [0.05]^x \, [0.05]^y$

or, $0.116 = k_H \, . \, [0.05]^x \, [0.05]^y$ ……………(6)

For run 4, initial rate = Rate 4

So, Rate 4 = k_D. [deuterated substrate]^x [aryl iodide]^y

or, $(15.422 - 11.709) / (138.319 - 105.686) = k_D \, . \, [0.05]^x \, [0.05]^y$

or, $0.113 = k_D \, . \, [0.05]^x \, [0.05]^y$ ……………(7)

So, k_H / k_D = Rate 1/ Rate 4

or, $k_H / k_D = 0.116 / 0.113$

or, $\mathbf{k_H / k_D = 1.03}$

2.8. Computational methods

Density functional theory (DFT) calculations were performed with Gaussian 16 Revision B.01.[7] The geometries of all intermediates and transition states were optimized with the B3LYP[8,9] functional and the 6-31G(d)[10-12] basis set for all atoms, except Pd, I, and Ag, which were described by the LANL08(f)[13] Effective Core Potential and valence basis set.[14] Single-point energies were evaluated with the wB97XD functional[15] and a def2-TZVP[16] basis set in solvent. Solvation effects were modeled using the SMD[17] implicit solvation model. Since SMD parameters for HFIP are not available by default in Gaussian 16, parameters for isopropanol were used with the dielectric constant modified to that of HFIP ($\varepsilon = 16.7$).

2.8.1. Mechanism of Pd-Catalyzed catalyzed *para*-C-H arylation

The dissociation energy of the trimeric Pd-catalyst is 14.7 kcal/mol. In our reaction system, due to greater dissociation energy of the trimeric catalyst (14.7 kcal/mol), the lowest activation energy of the oxidative addition transition state ts-6a is 39.7 kcal/mol. This ruled out trimeric Pd as the active catalyst in this reaction.

$$1/3{*}Pd_3(OAc)_6 \xrightleftharpoons{\text{14.7 kcal/mol}} Pd(OAc)_2$$

Also, the occurrence of hydrolysis is consistent with the known reactivity of $Pd_3(OAc)_6$ toward ligands such as amines, phosphines and arsines leading to mononuclear complexes in which the acetate groups are monodentate[18,19]. Therefore, the Pd monomeric as the active catalyst mechanism has been presented in our reaction.

To understand the origin of *para*-selectivity, we investigated and compared several possible *ortho*-, *meta*-, and *para*- concerted metalation-deprotonation (CMD) transition state structures. The main text (Fig. 5) compares barrier heights of the *para*-TS with an acetate ligand and a mono-protected amino acid (MPAA) ligand. All *ortho*-, *meta*-, and *para*- TSs are shown in Supplementary Fig. 14 and Supplementary Fig. 15. As shown in Supplementary Fig. 15, with the MPAA Ac-Gly-OH, TS structures have a [5,6]-palladacycle conducive for C–H activation. The *para*-TS is favored over the corresponding *meta* and *ortho* structures by 3.5 and 3.8 kcal/mol, respectively. With the N-Ac-Gly-OH ligand, the *para*-TS1 is 4.3 and 1.9 kcal/mol more stable than the *meta*- and *ortho*-TS1 in Supplementary Fig. 14, respectively. With an acetate ligand (Supplementary Fig. 15) we obtain the same trend in regioselectivity, although the barriers are uniformly higher than for the MPAA-assisted pathway.

Experimentally, HFIP gave superior results to other solvents. To investigate whether specific hydrogen-bonding interactions between HFIP and substrate may play a significant

role in our computational model, CMD transition structures were located with an explicit molecule of HFIP (Supplementary Fig. 16). Quantitatively, the relative stabilities were affected by less than 1 kcal/mol, and the same qualitative trend emerged as in our implicitly solvated calculations. Therefore, explicit HFIP molecules were not used in remaining computational studies. We also investigated alternative computational models varying the amino acid N-protecting group. Experimentally, Fmoc was found to be useful in selectivity enhancement. Fmoc-Glycine ligand-assisted CMD transition states were computed (Supplementary Fig. 17), however, the use of a simpler N-Ac-Gly-OH ligand in our computations led to very similar computed energetics, and so this smaller system was used throughout.

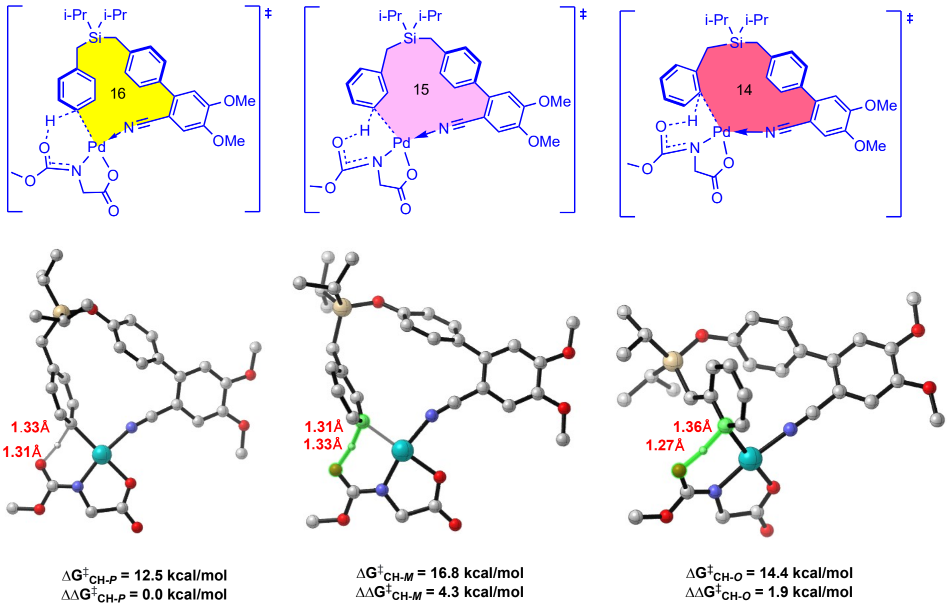

Supplementary Figure 14. Optimized geometries and corresponding relative Gibbs free energies (in kcal/mol) for N-Ac-Gly-OH ligand assisted *para*-, *meta*-, and *ortho*- transition states.

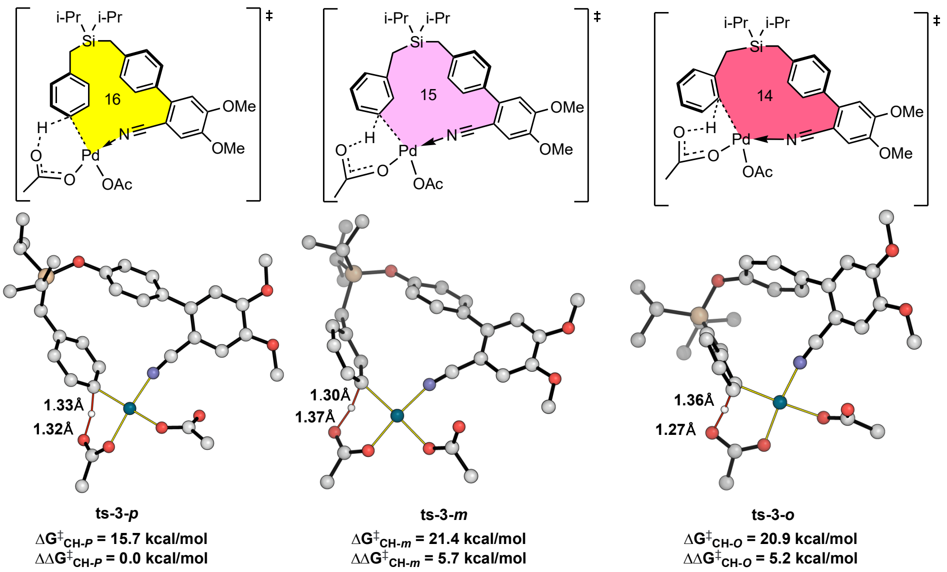

Supplementary Figure 15. Optimized geometries and corresponding relative Gibbs free energies (in kcal/mol) for acetate assisted *para*-, *meta*-, and *ortho*- C−H activation transition states.

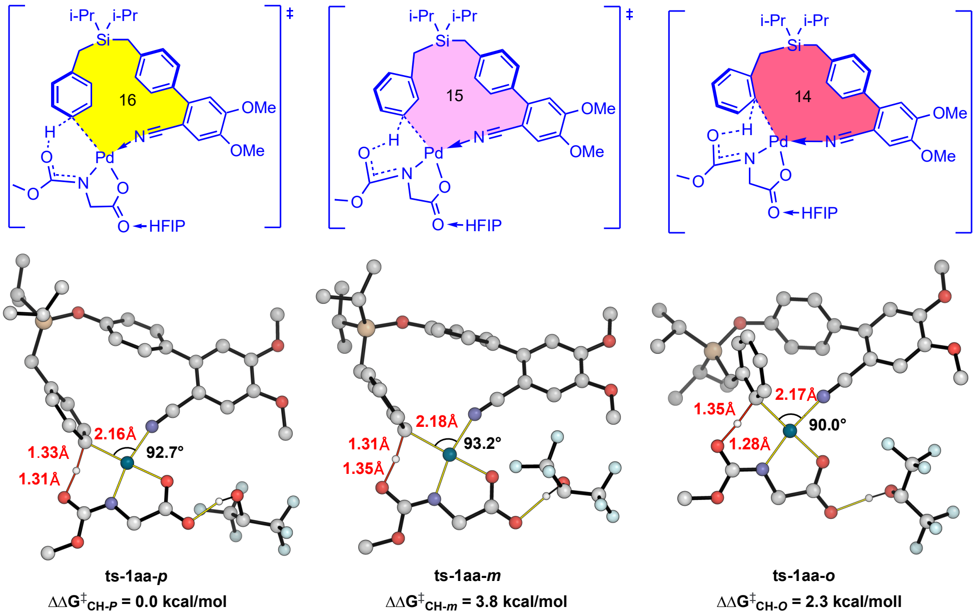

Supplementary Figure 16. Optimized geometries and corresponding relative Gibbs free energies (in kcal/mol) for the N-Ac-Gly-OH ligand assisted *para*-, *meta*-, and *ortho*-transition states with one molecule HFIP.

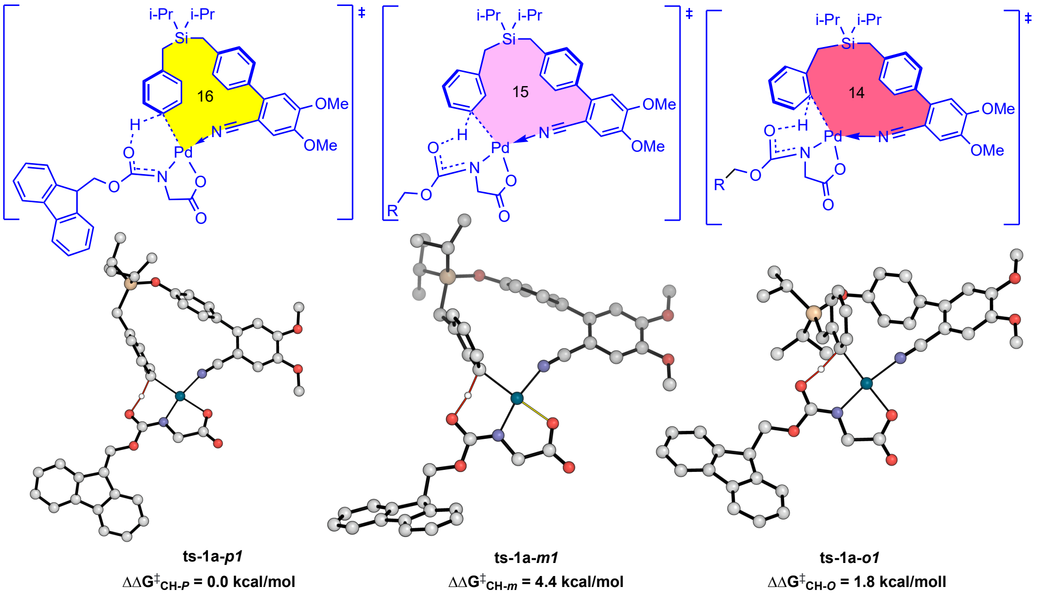

Supplementary Figure 17. Optimized geometries and corresponding relative Gibbs free energies (in kcal/mol) for the Fmoc-Glycine ligand assisted *para*-, *meta*-, and *ortho*-transition states.

Various computational approaches were pursued to quantify and interrogate the basis for site-selectivity. Firstly, we defined an isodesmic reaction scheme allowing comparison of cyclic and acyclic versions of regioisomeric structures (Supplementary Fig. 18). Based on our overall Gibbs energy surface and experimental kinetic studies, we focus on the turnover-determining oxidative addition TS. The corresponding macrocyclic ring strain Gibbs energies for the 16-membered *para*-TS, the 15-membered *meta*- and 14-membered *ortho*-TSs in the turnover-determining step is 0.0, 4.3 and 3.2 kcal/mol, respectively. The difference between the computed energies of the macrocyclic strain/unstrained systems in the transition states indicate the 16-membered para-TS2a has the least strain.

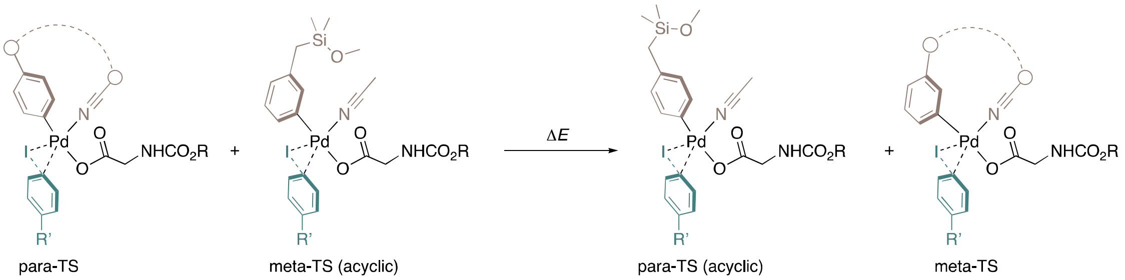

Supplementary Figure 18. Isodesmic reaction scheme used to the compare ring-strain in regioisomeric transition structures.

Since both mono- and multi-metallic mechanisms have been proposed in the study of Pd-catalyzed C–H activation with MPAAs, we considered several possible pathways. The computed Gibbs energy profile of a Pd–Ag heterodimeric catalyzed *para*-C–H arylation reaction is shown in Supplementary Fig. 19. We compared several possible *para*-selective CMD transition-state structures, involving $PdAg(OAc)_3$ (**ts-4**), $PdAgL(OAc)_2$ (**ts-4a**), PdAgL(OAc)(**ts-4b**), and PdAgL(OAc) with one molecule of HFIP (**ts-4c**). In these studies, the CMD **ts-4** with $PdAg(OAc)_3$ has the lowest energetic barrier of 14.2 kcal/mol. The oxidative addition of aryl iodide onto Pd(II) species **int9** occur *via* transition state **ts-5** with an energetic span of 29.6 kcal/mol, which is 2.2 kcal/mol higher than that of our PdL monomer models. Subsequently, reductive elimination of C–C bond via **ts-6** with an activation free energy of 9.9 kcal/mol gives arylation-Pd(II) complex **int11**. *Ortho*-, *meta*-, and *para*-selective $PdAg(OAc)_3$ CMD transition states are shown in Supplementary Fig. 20. We found that the *para*- transition state **TS4-p** is only 0.1 kcal/mol more stable than the *meta*- transition state **TS4-m** in terms of Gibbs free energies. To conclude, the overall barrier for the heterodimeric pathway is slightly higher than that obtained with a single Pd center, and the computed regioselectivity agrees less favorably with experiment. On this basis, we focus our attention in the manuscript on monomeric pathways only.

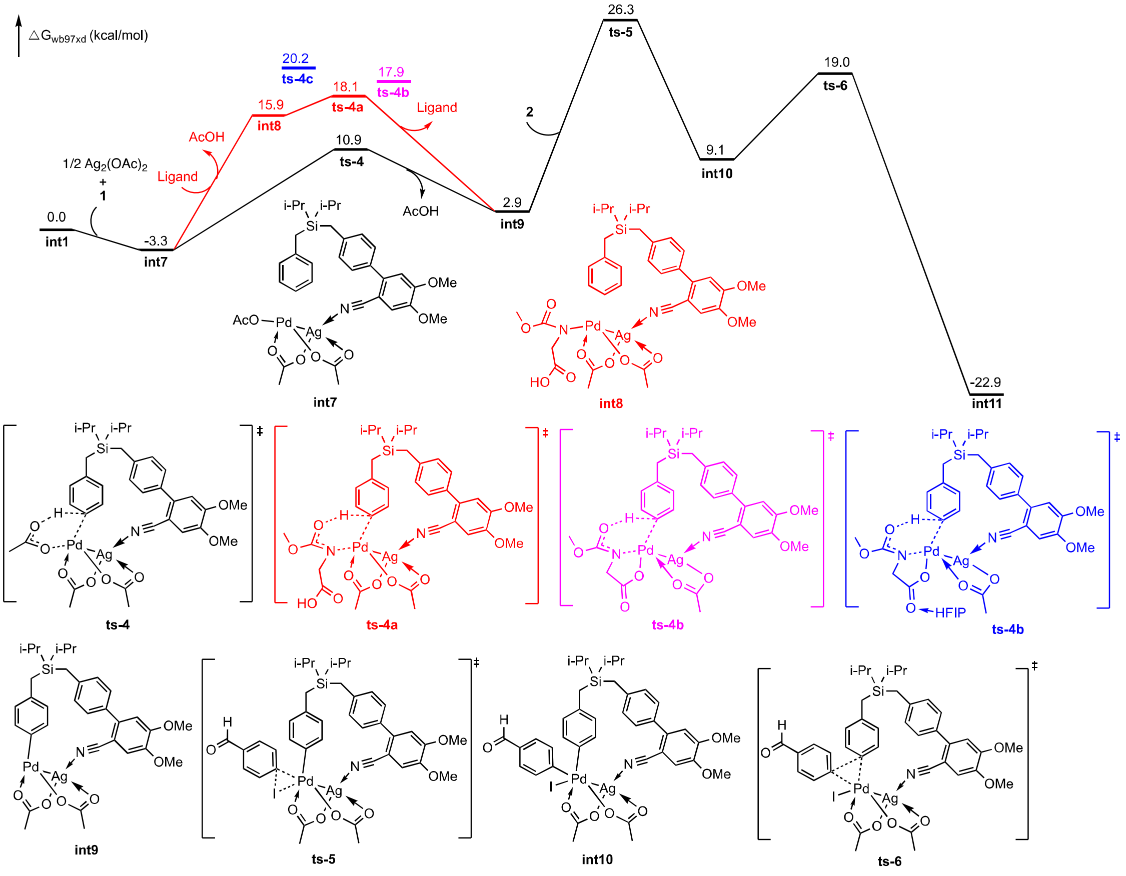

Supplementary Figure 19. Computed Gibbs energy profile of Pd–Ag heterodimeric catalyzed *para*-C–H arylation reaction at the wB97XD/def2-TZVP//B3LYP/6-31G(d)+LANL08(f) level of theory.

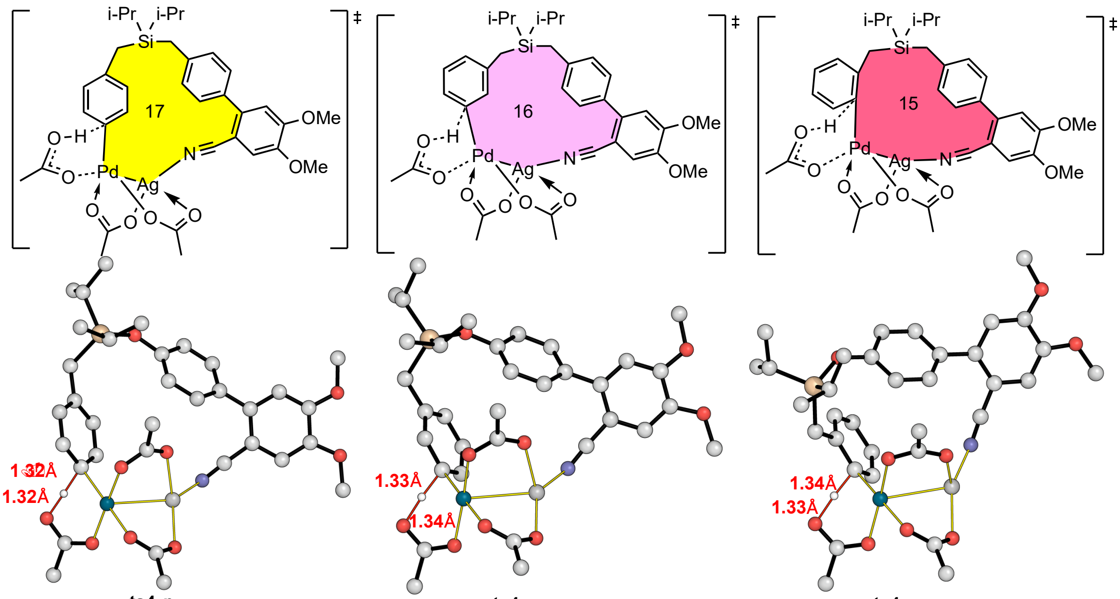

ts4-*p* $\Delta\Delta G_{CH\text{-}P}$ = 0.0 kcal/mol

ts4-*m* $\Delta\Delta G_{CH\text{-}m}$ = 0.1 kcal/mol

ts4-*o* $\Delta\Delta G_{CH\text{-}O}$ = 4.1 kcal/moll

Supplementary Figure 20. Optimized geometries and corresponding relative Gibbs free energies (in kcal/mol) for the *para*-, *meta*-, and *ortho*- C−H activation transition states for Pd–Ag heterodimeric model.

3. X-Ray Crystallographic Data

4'-((((4'-formyl-[1,1'-biphenyl]-4-yl)methyl)diisopropylsilyl)oxy)-4,5-dimethoxy-[1,1'-biphenyl]-2-carbonitrile (3ak): CCDC 2163139

Bond precision:	C-C = 0.0183 A	Wavelength=0.71073	
Cell:	a=14.8394(10)	b=15.3036(9)	c=26.486(2)
	alpha=90	beta=90	gamma=90
Temperature:	150 K		

	Calculated	Reported
Volume	6014.9(7)	6014.8(7)
Space group	P n a 21	P n a 21
Hall group	P 2c -2n	P 2c -2n
Moiety formula	C35 H37 N O4 Si	2(C35 H37 N O4 Si)
Sum formula	C35 H37 N O4 Si	C70 H74 N2 O8 Si2
Mr	563.75	1127.49
Dx,g cm-3	1.245	1.245
Z	8	4
Mu (mm-1)	0.118	0.118
F000	2400.0	2400.0
F000’	2401.65	
h,k,lmax	17,18,31	17,18,31
Nref	10628[5440]	10283
Tmin,Tmax	0.929,0.945	0.352,1.000
Tmin’	0.929	

Correction method= # Reported T Limits: Tmin=0.352 Tmax=1.000AbsCorr = MULTI-SCAN

Data completeness= 1.89/0.97 Theta(max)= 24.994

R(reflections)= 0.0986(5080) wR2(reflections)= 0.3002(10283)

S = 1.032 Npar= 751

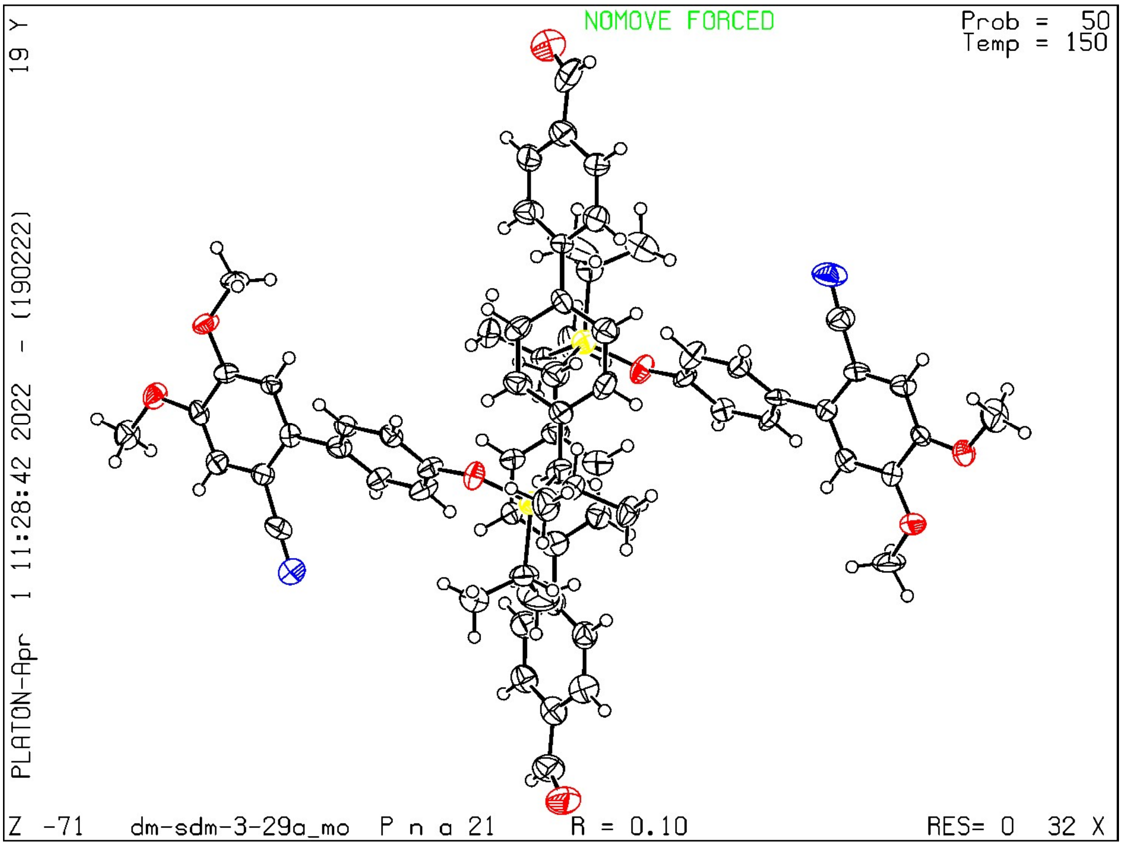

4. NMR Spectra

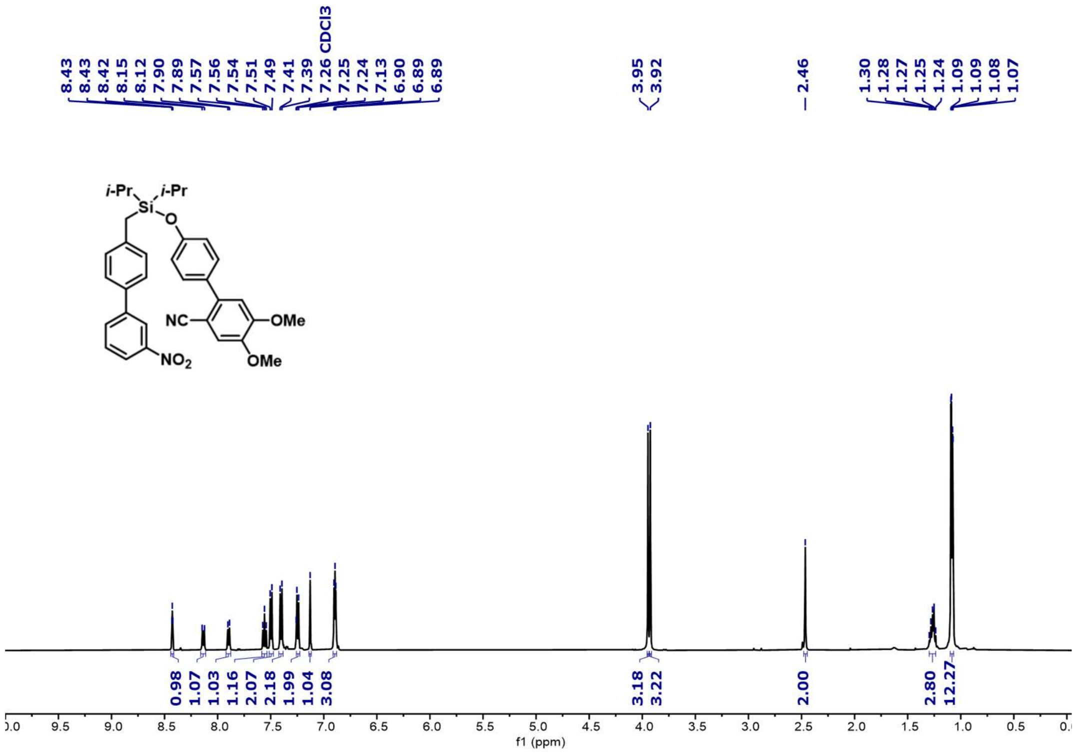

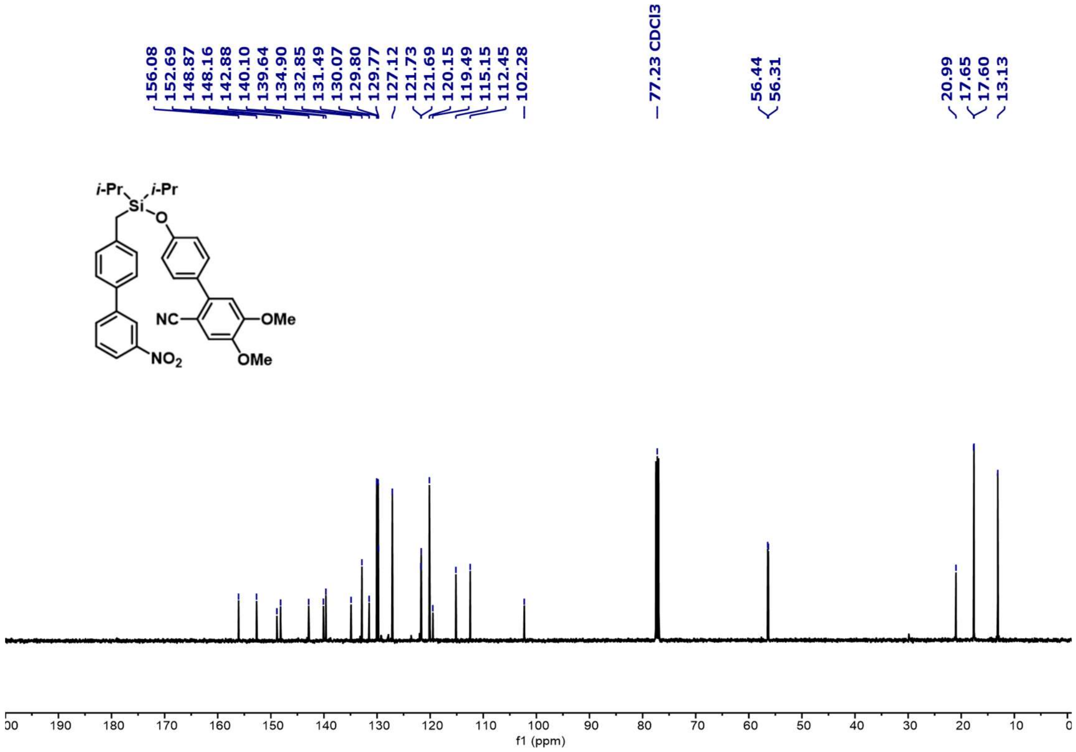

Supplementary Figure 21. ^{1}H (top) and ^{13}C (bottom) NMR of **3aa**

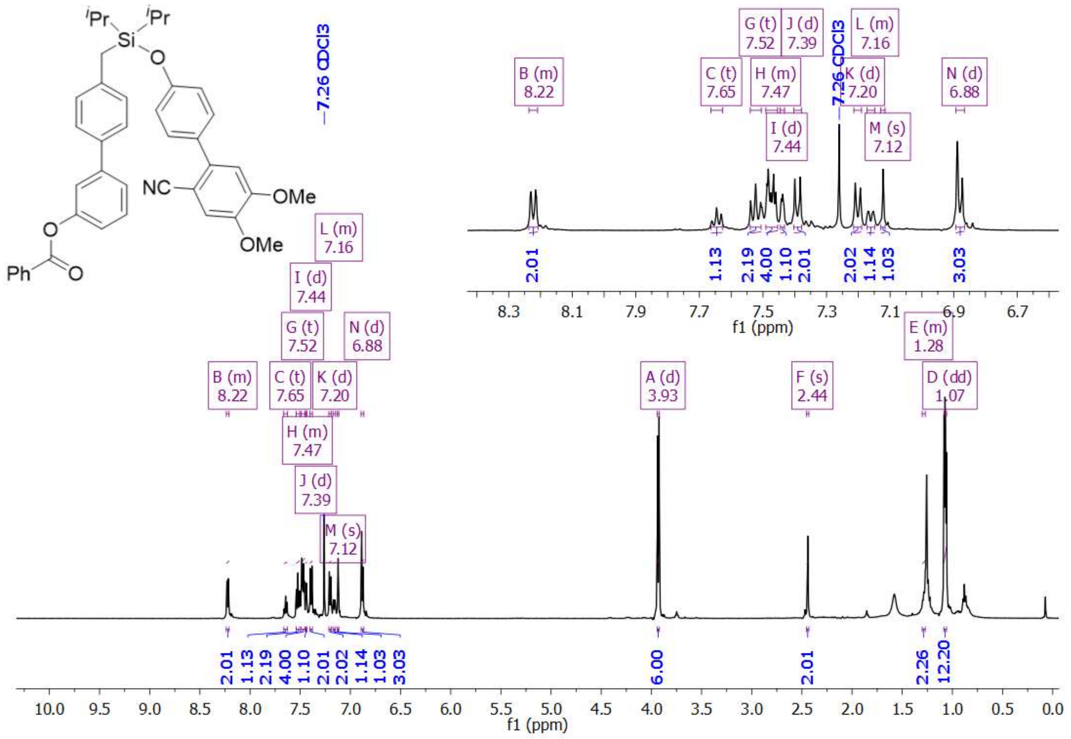

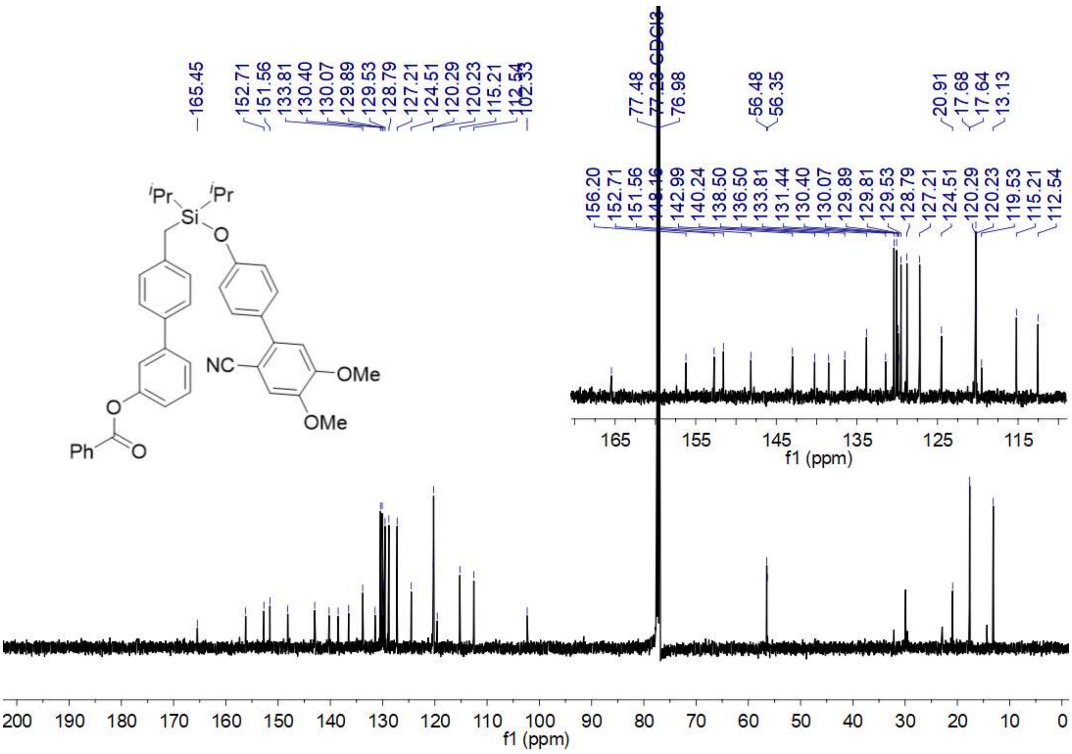

Supplementary Figure 22. ^{1}H (top) and ^{13}C (bottom) NMR of **3ab**

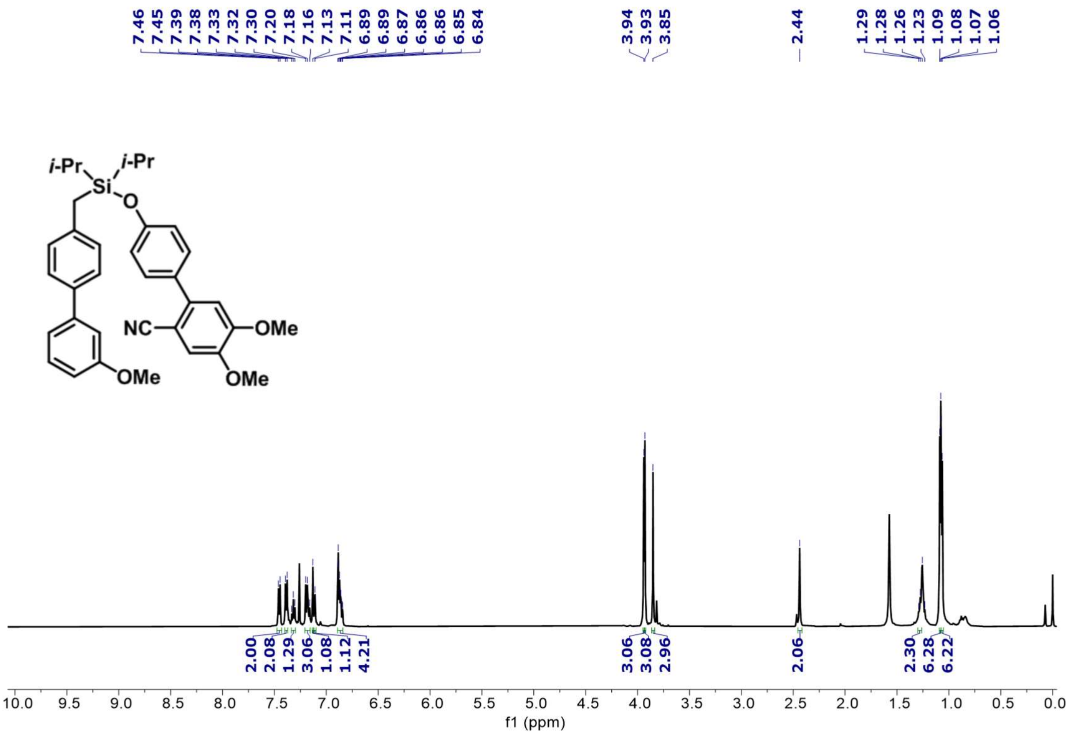

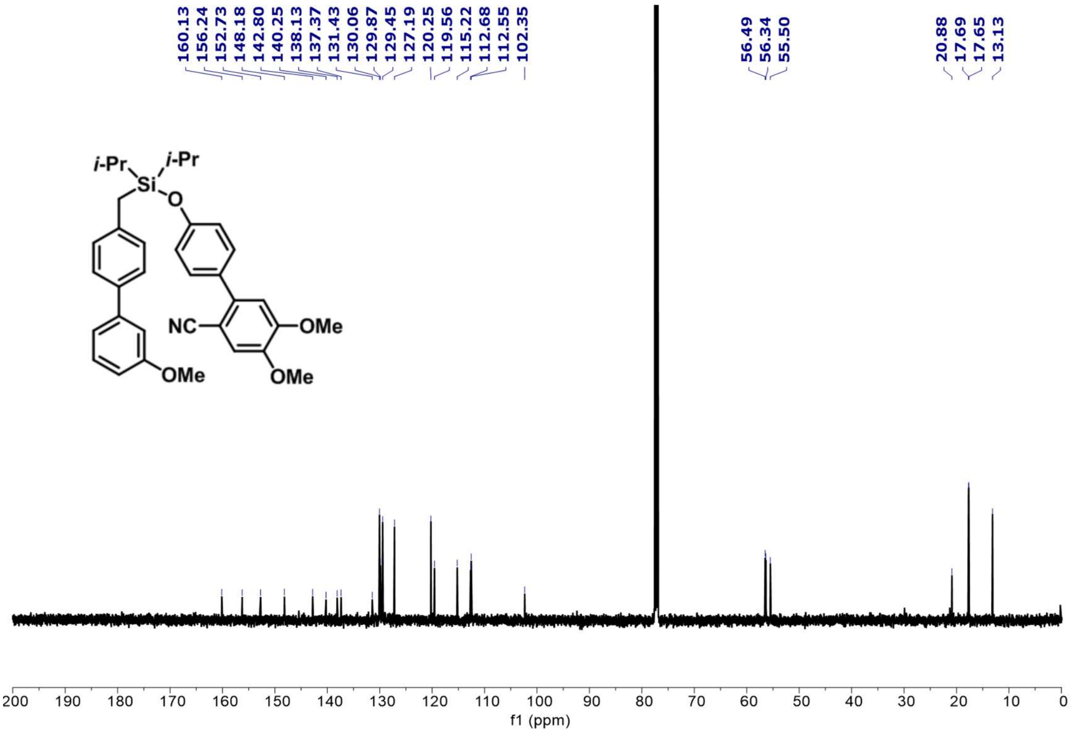

Supplementary Figure 23. ^{1}H (top) and ^{13}C (bottom) NMR of **3ac**

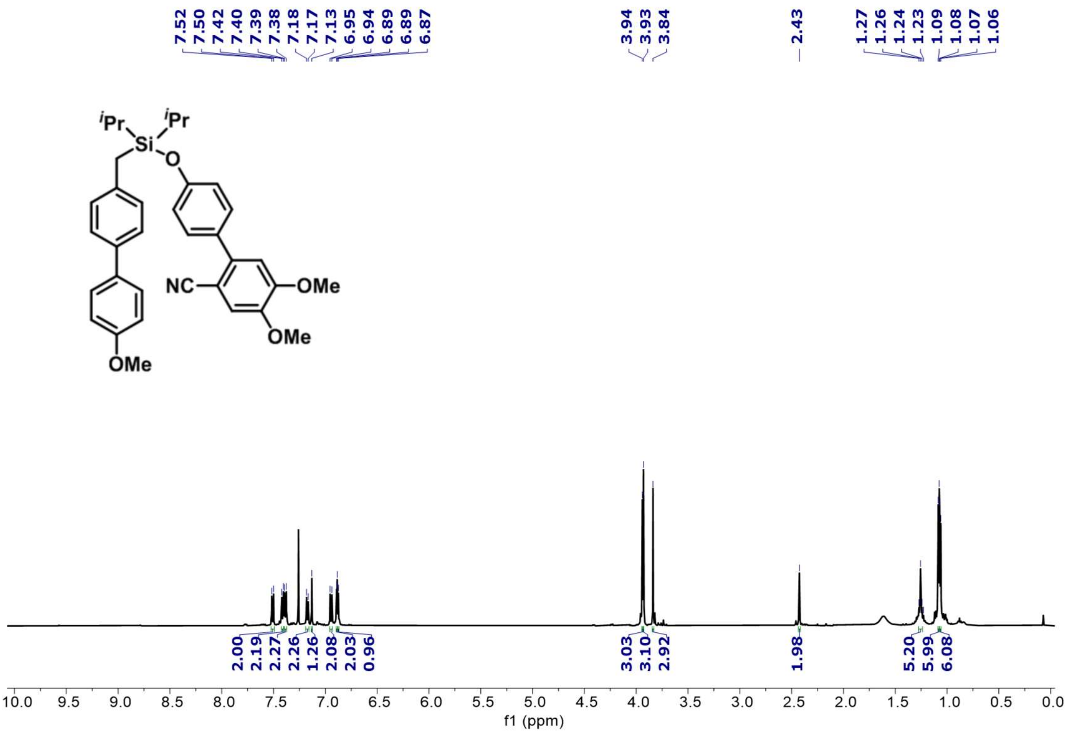

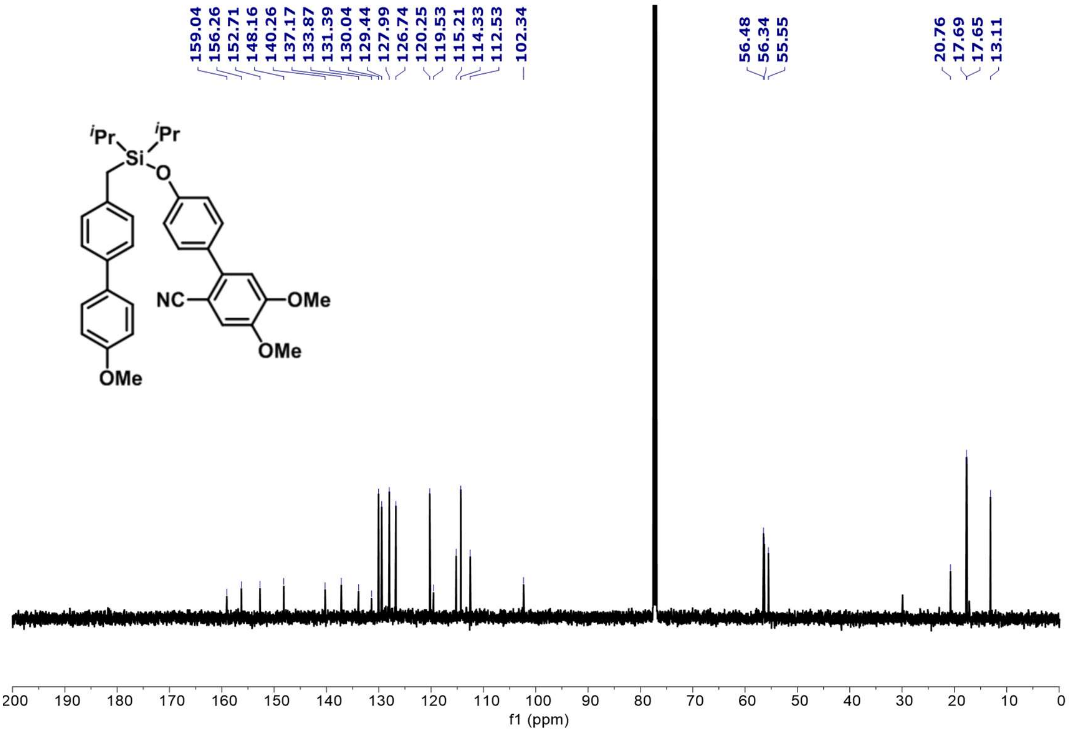

Supplementary Figure 24. ^{1}H (top) and ^{13}C (bottom) NMR of **3ad**

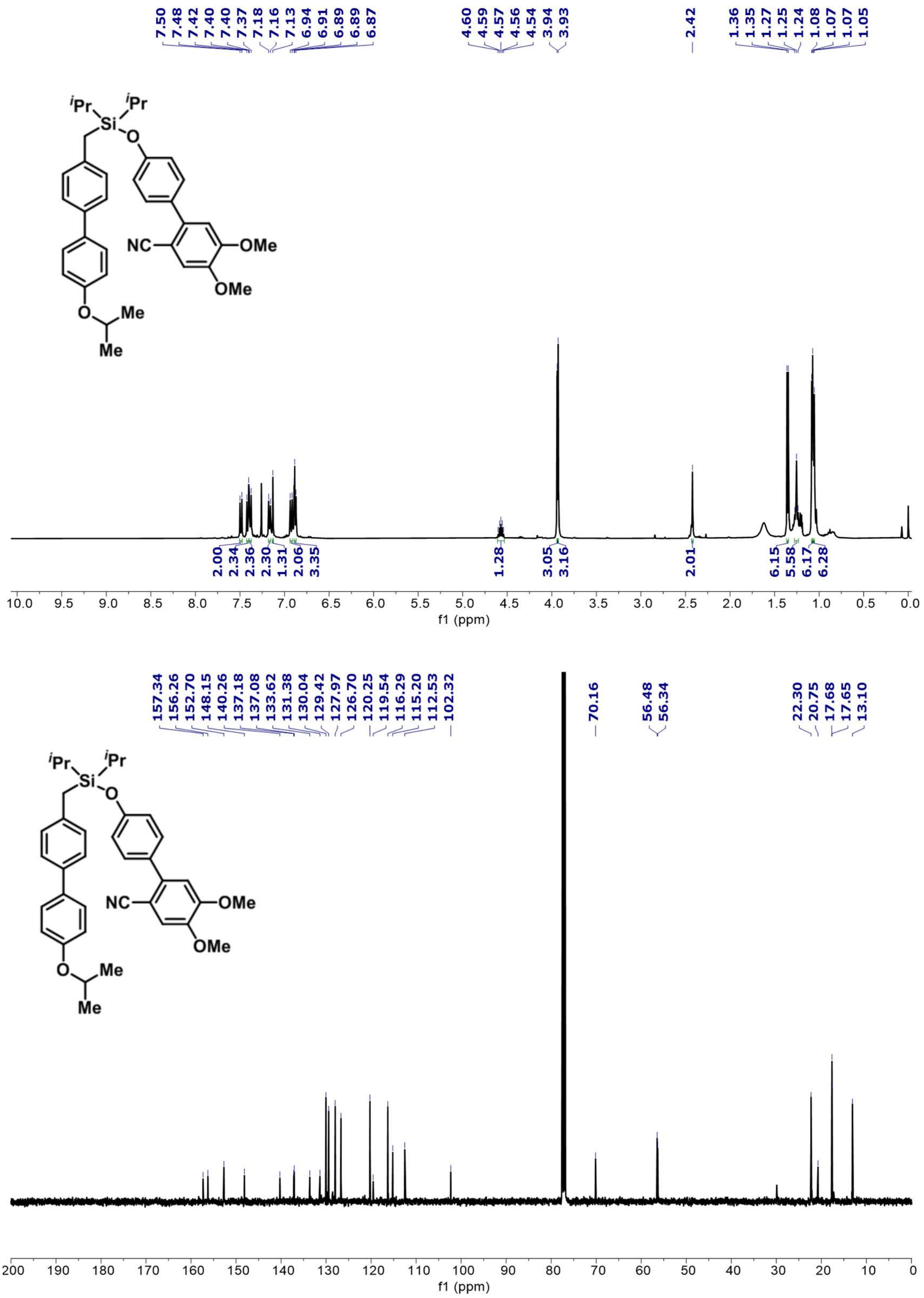

Supplementary Figure 25. ^{1}H (top) and ^{13}C (bottom) NMR of **3ae**

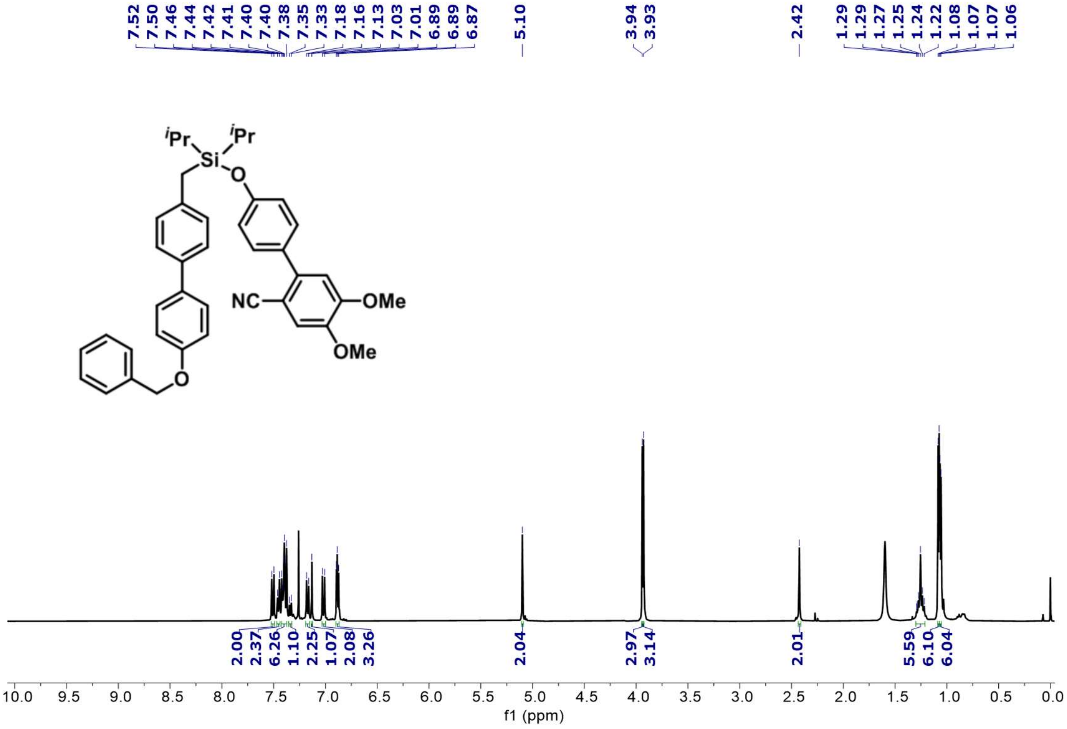

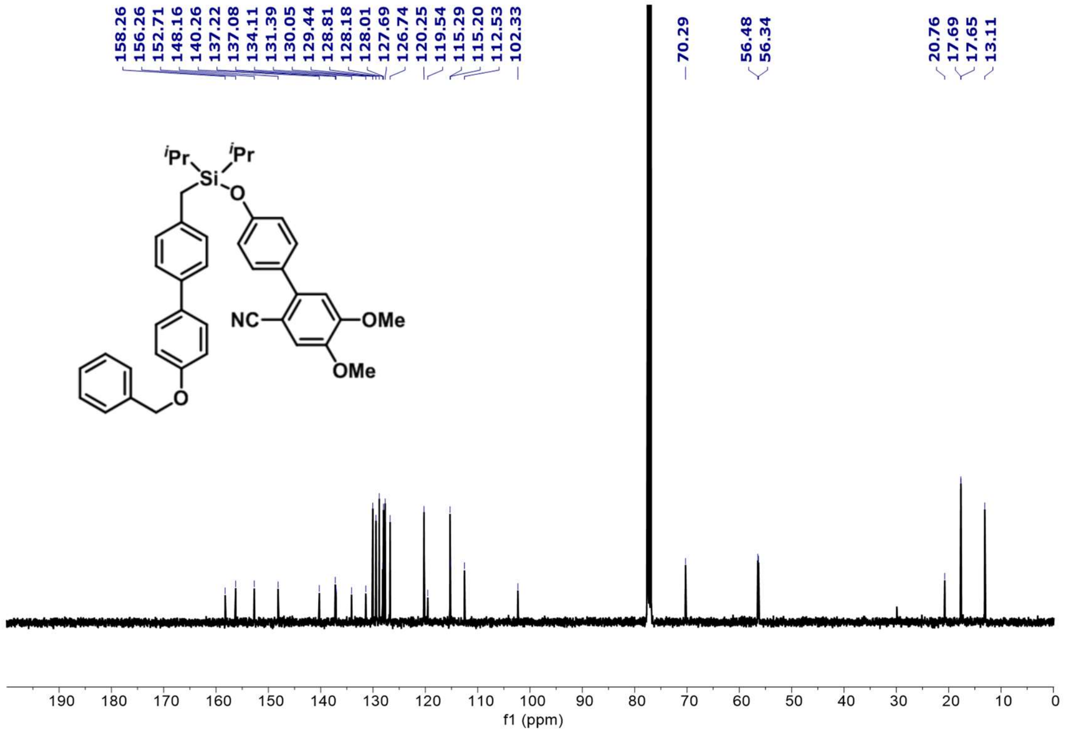

Supplementary Figure 26. ^{1}H (top) and ^{13}C (bottom) NMR of **3af**

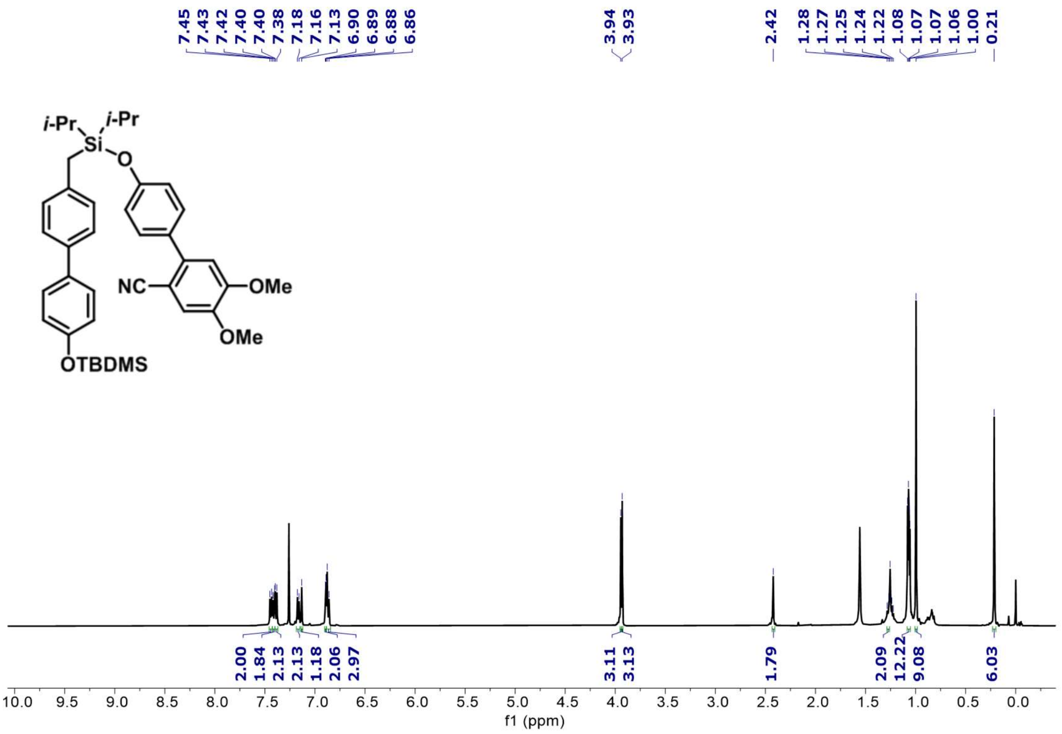

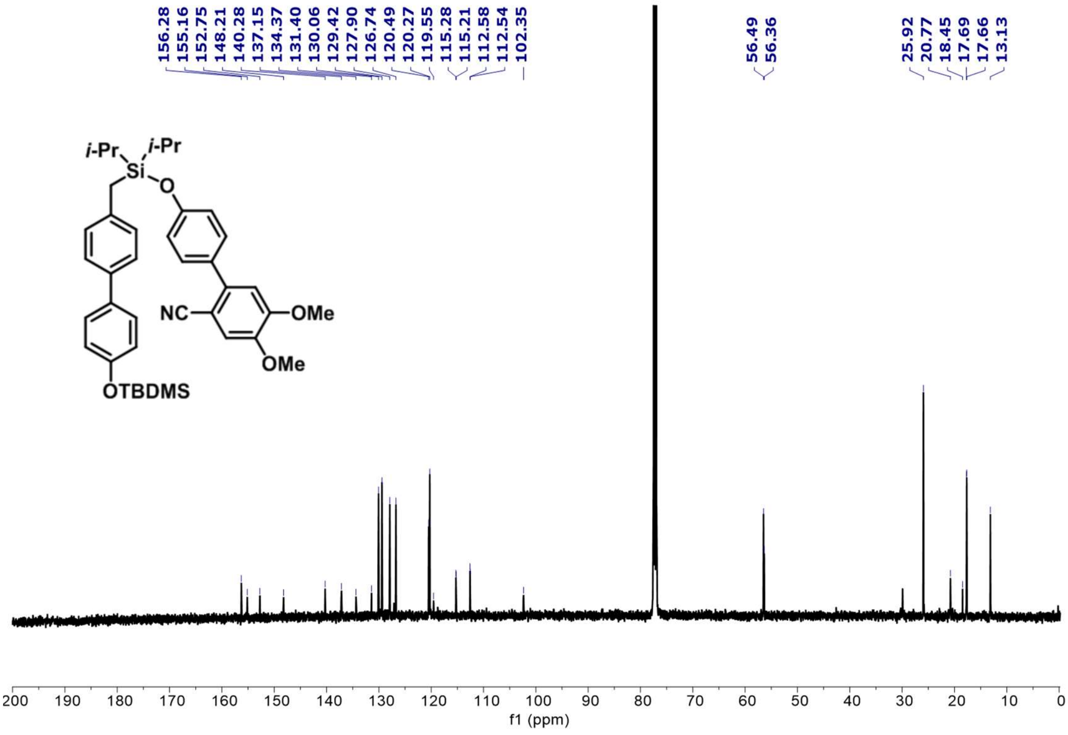

Supplementary Figure 27. ^{1}H (top) and ^{13}C (bottom) NMR of **3ag**

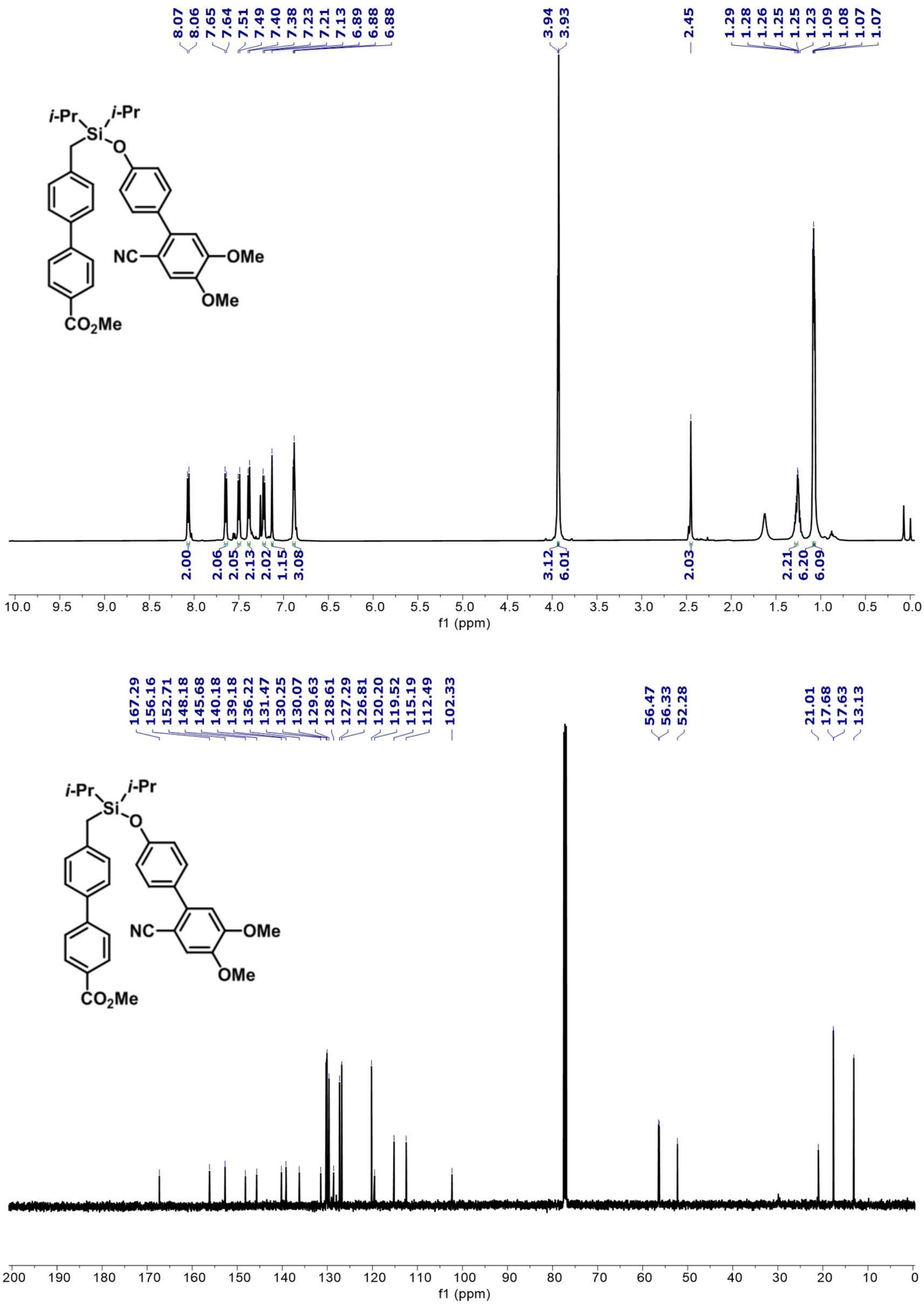

Supplementary Figure 28. ^{1}H (top) and ^{13}C (bottom) NMR of **3ah**

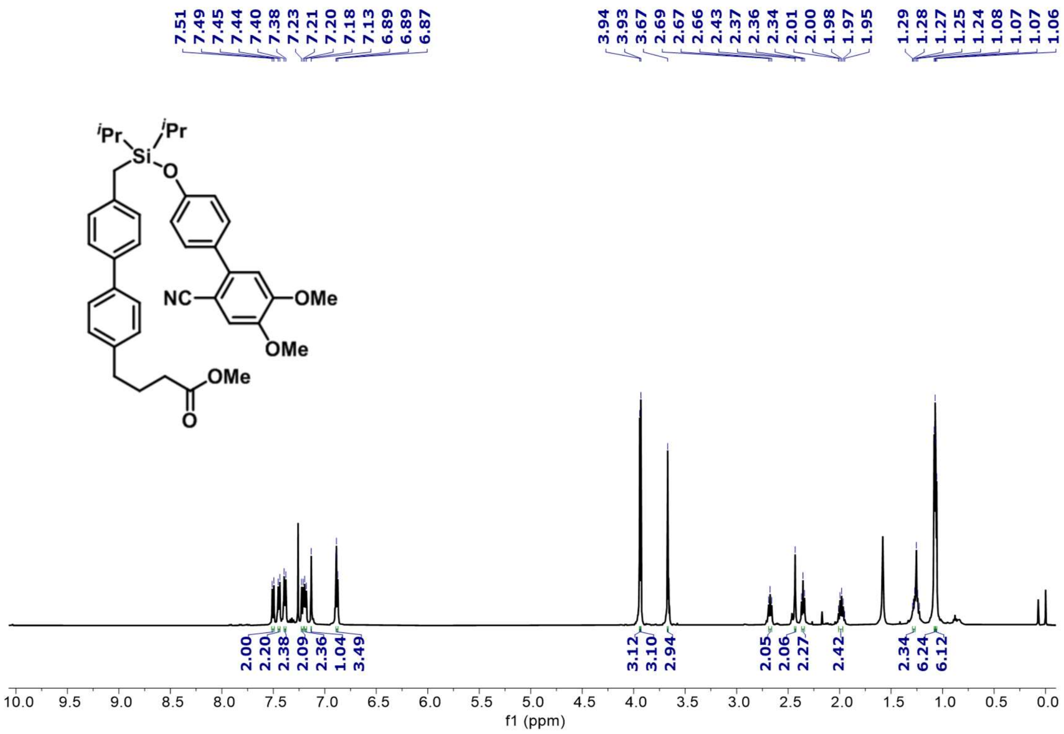

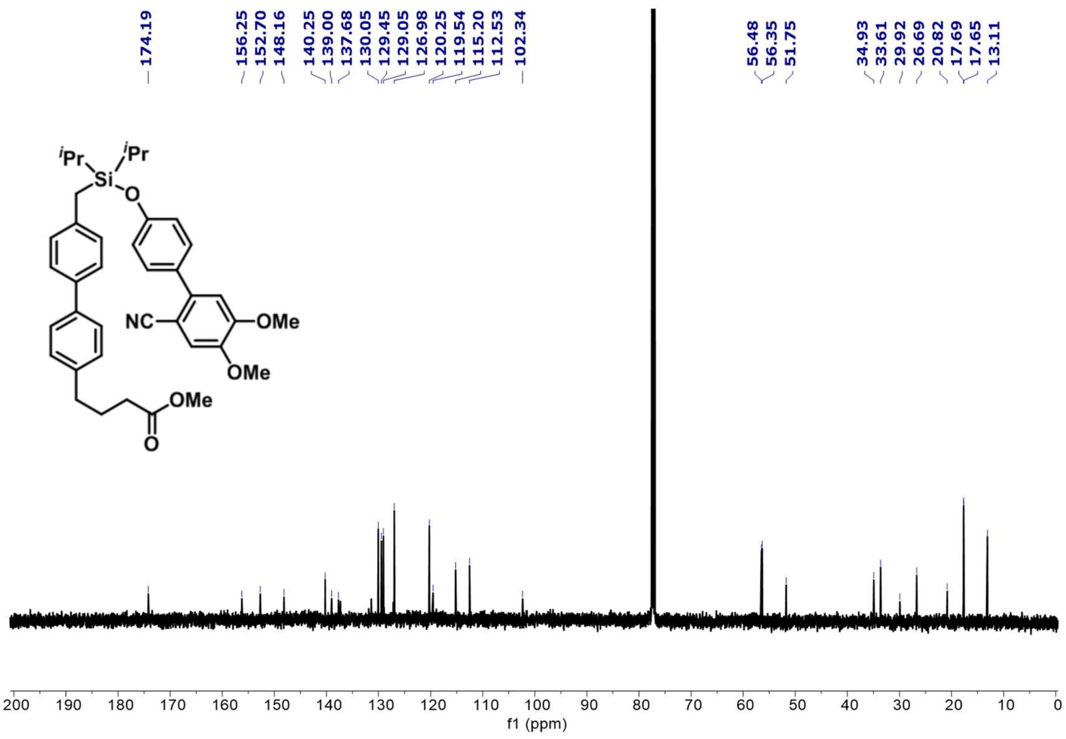

Supplementary Figure 29. ^{1}H (top) and ^{13}C (bottom) NMR of **3ai**

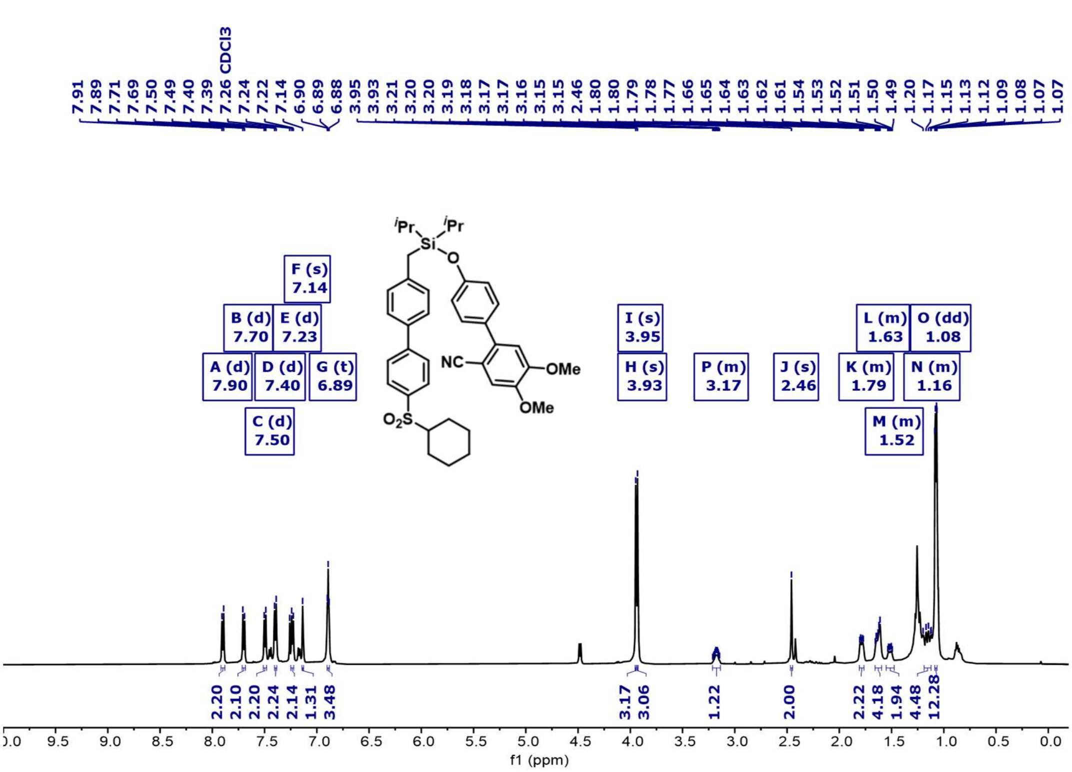

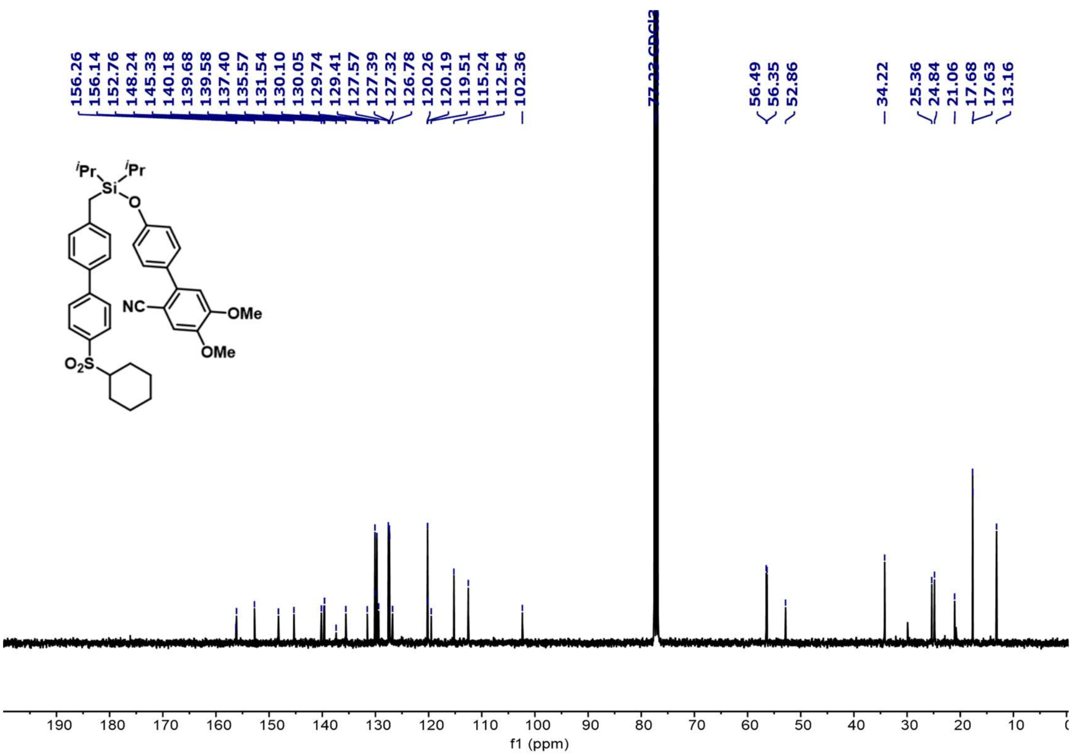

Supplementary Figure 30. ^{1}H (top) and ^{13}C (bottom) NMR of **3aj**

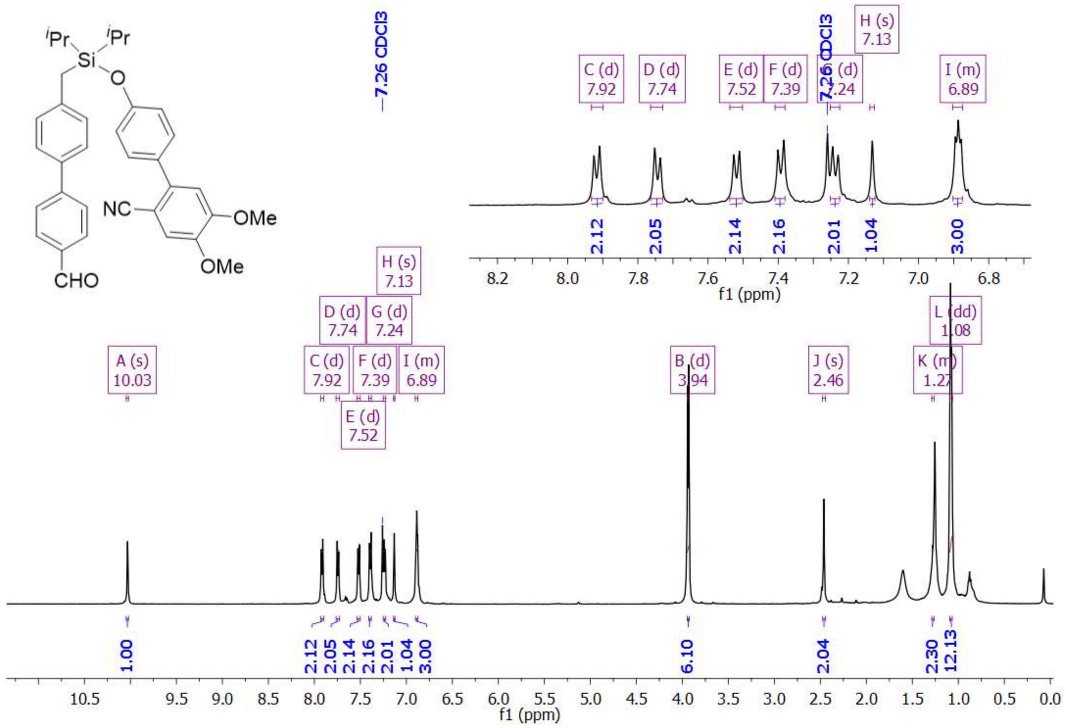

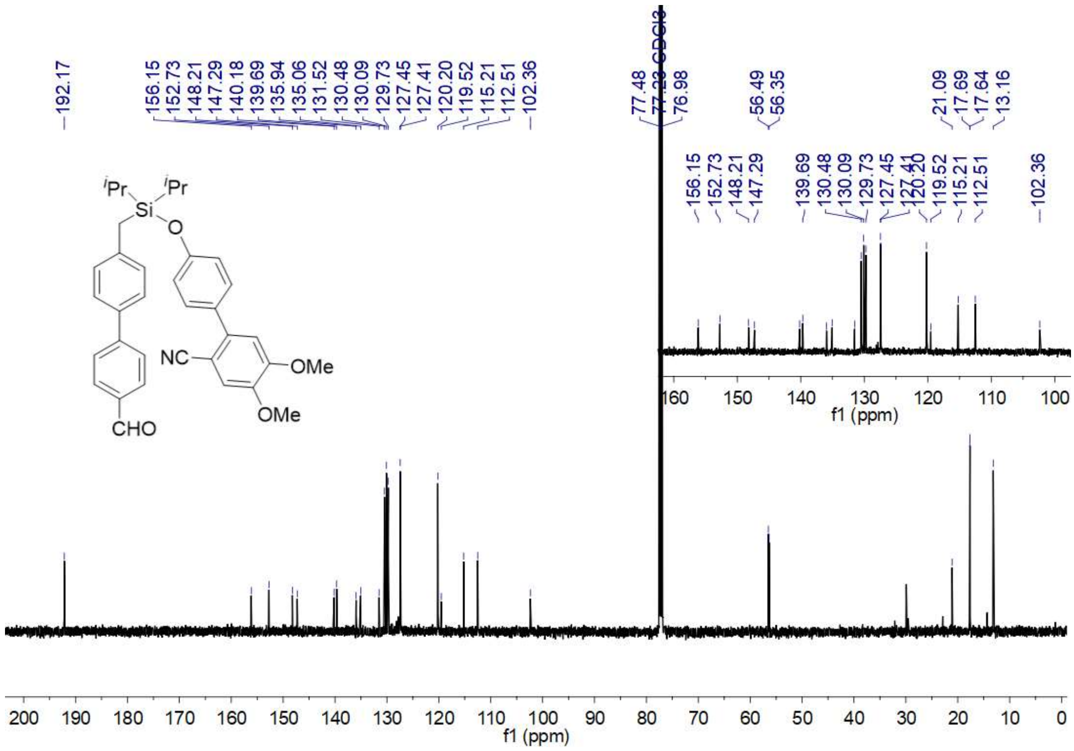

Supplementary Figure 31. ^{1}H (top) and ^{13}C (bottom) NMR of **3ak**

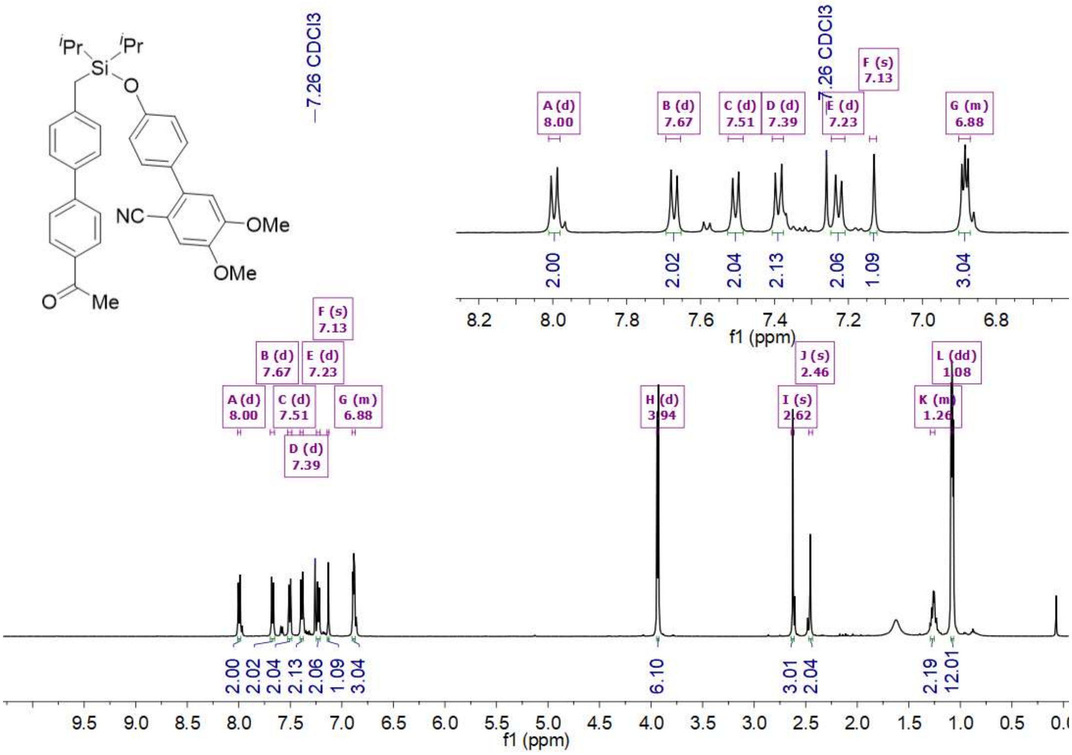

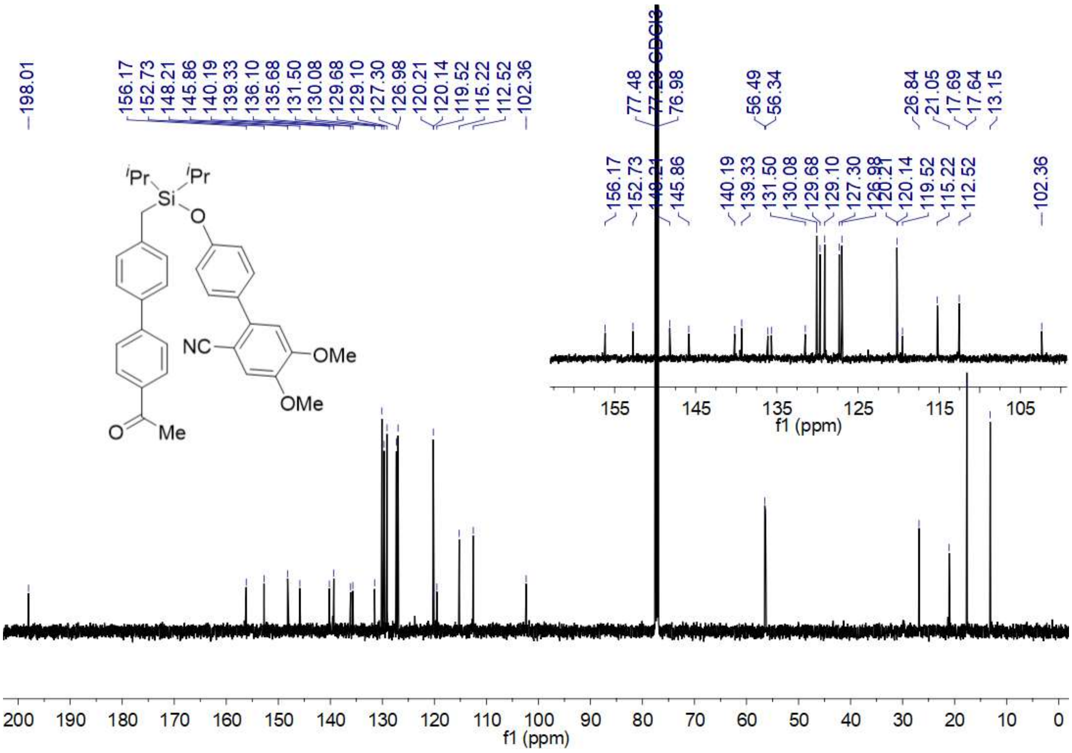

Supplementary Figure 32. ^{1}H (top) and ^{13}C (bottom) NMR of **3al**

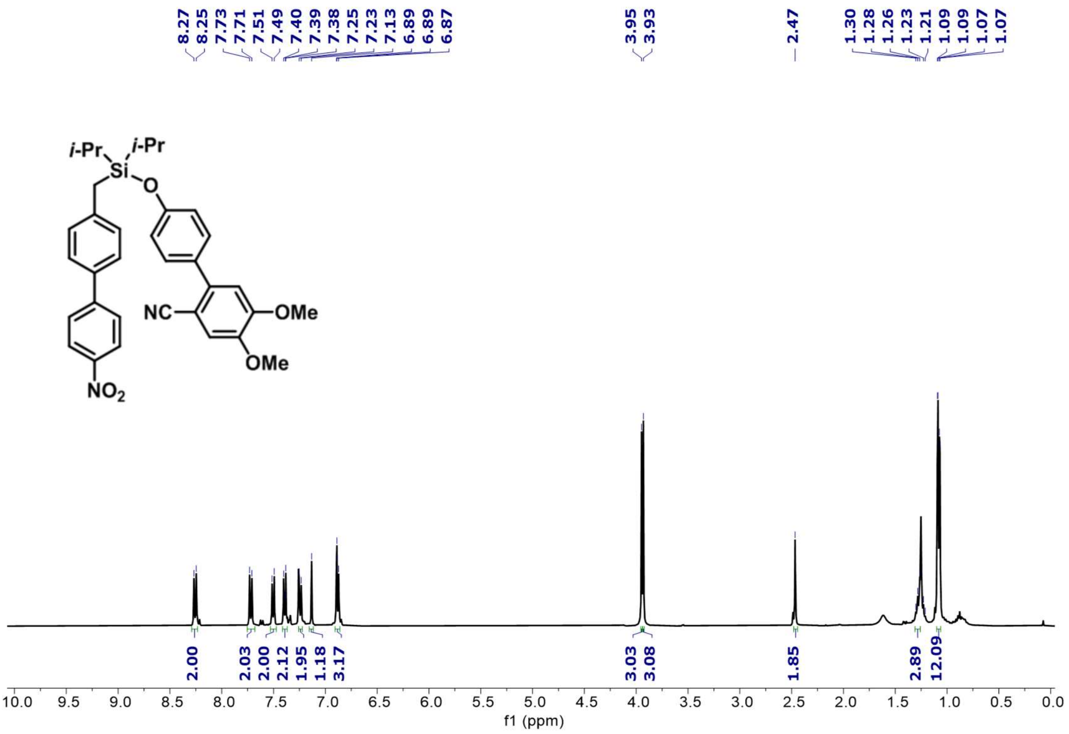

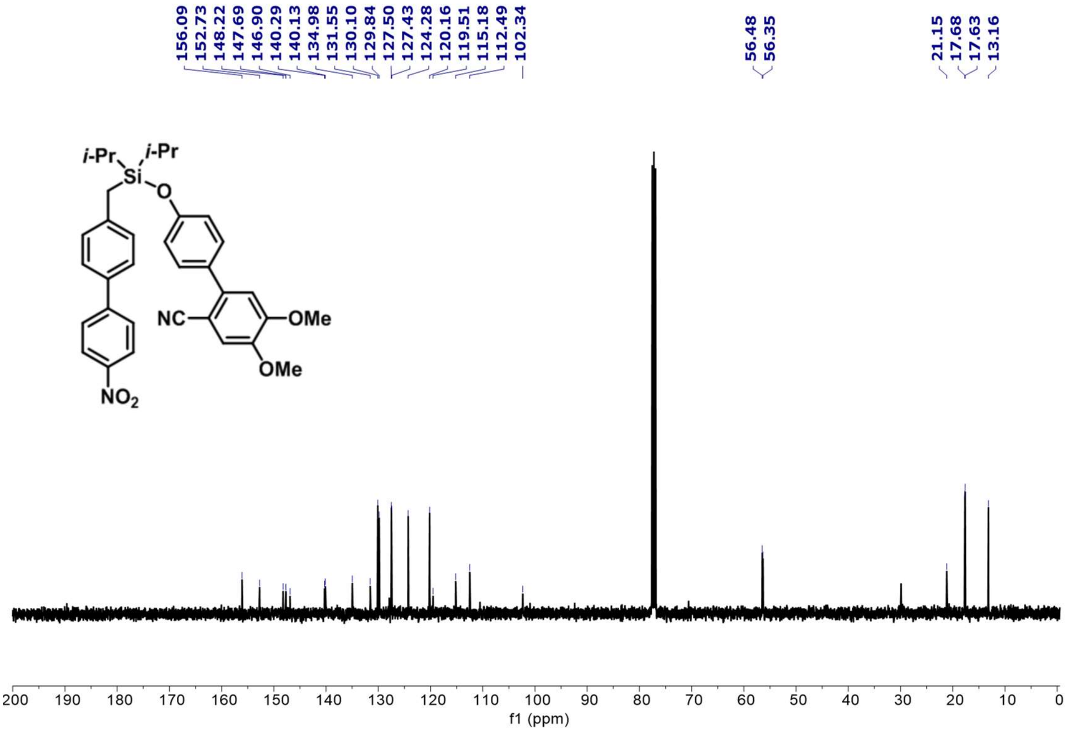

Supplementary Figure 33. ^{1}H (top) and ^{13}C (bottom) NMR of **3am**

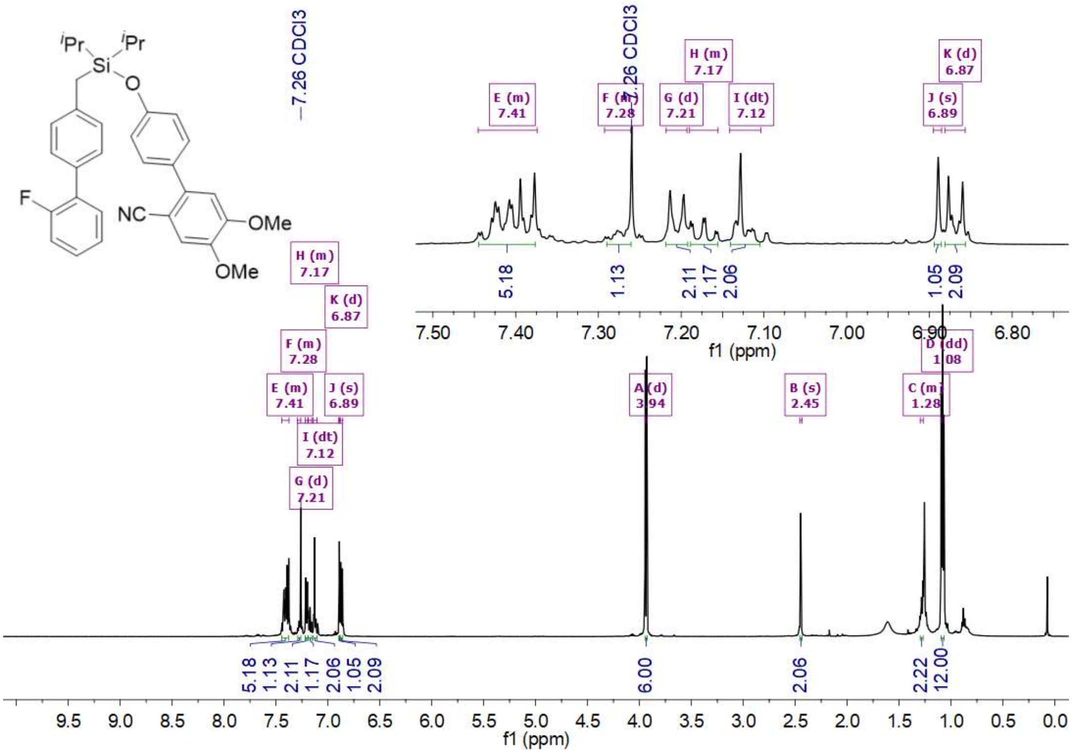

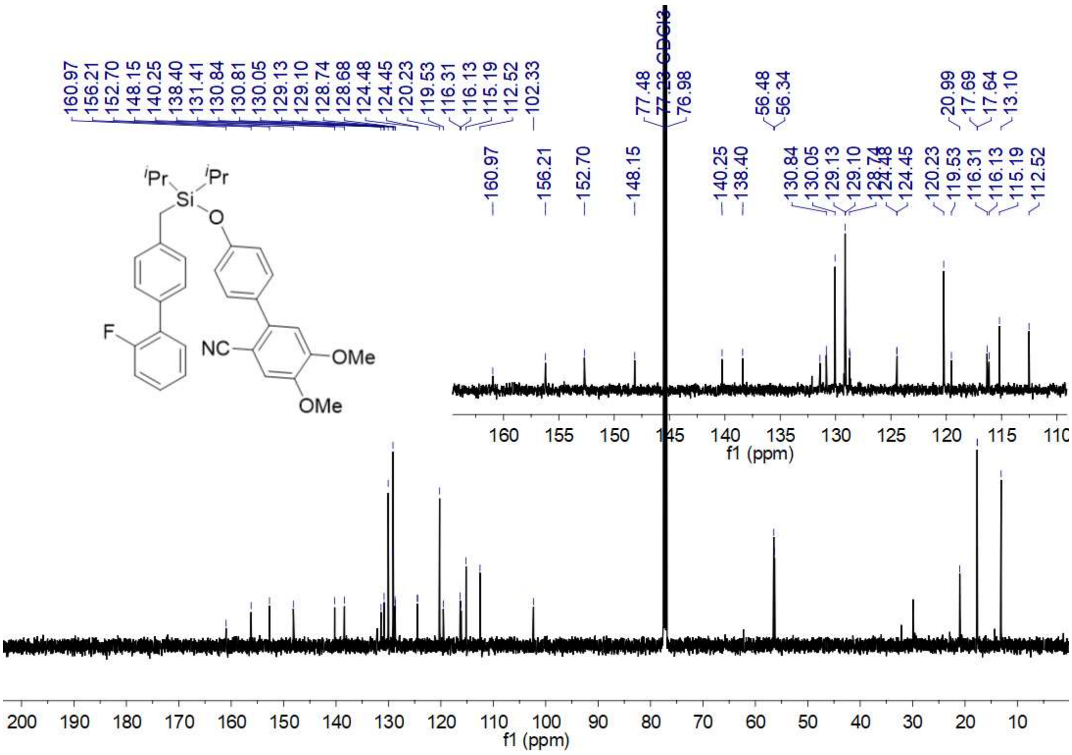

Supplementary Figure 34. ^{1}H (top) and ^{13}C (bottom) NMR of **3an**

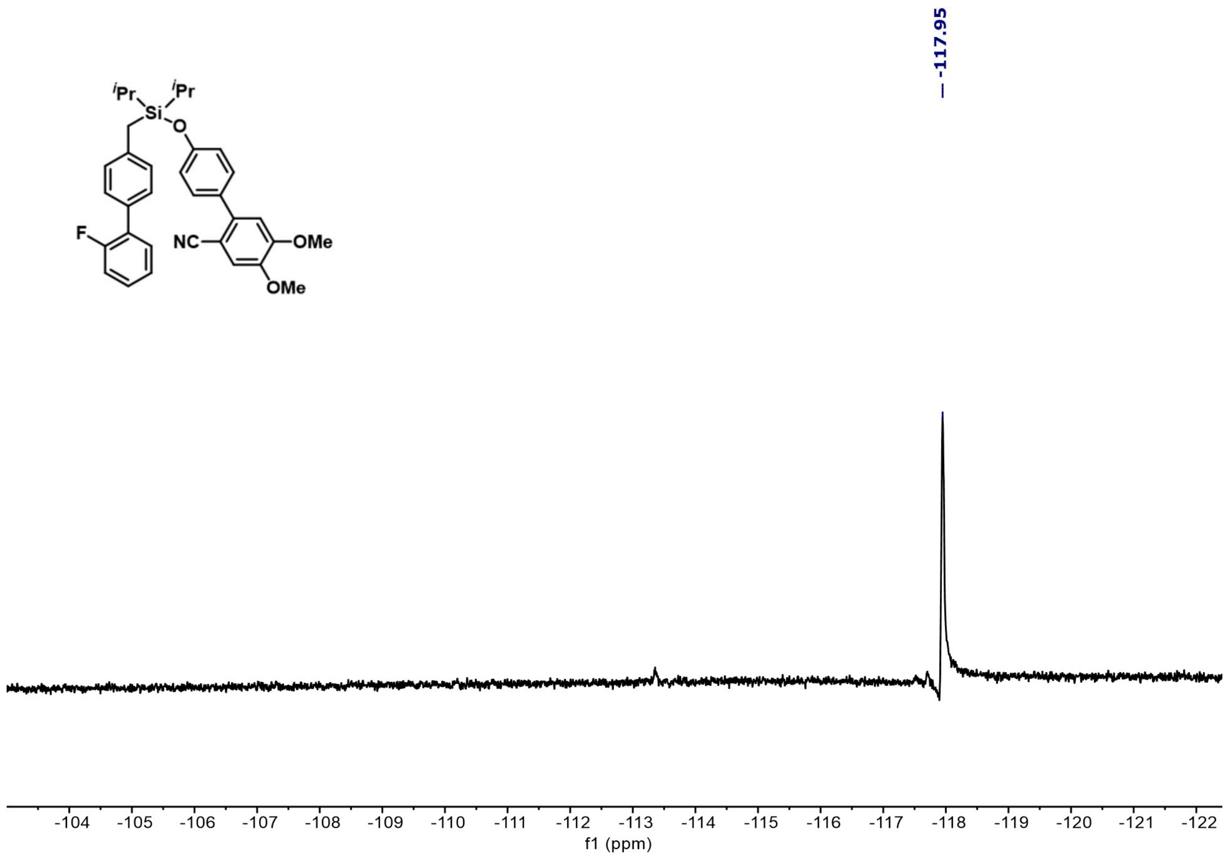

Supplementary Figure 35. ^{19}F NMR of **3an**

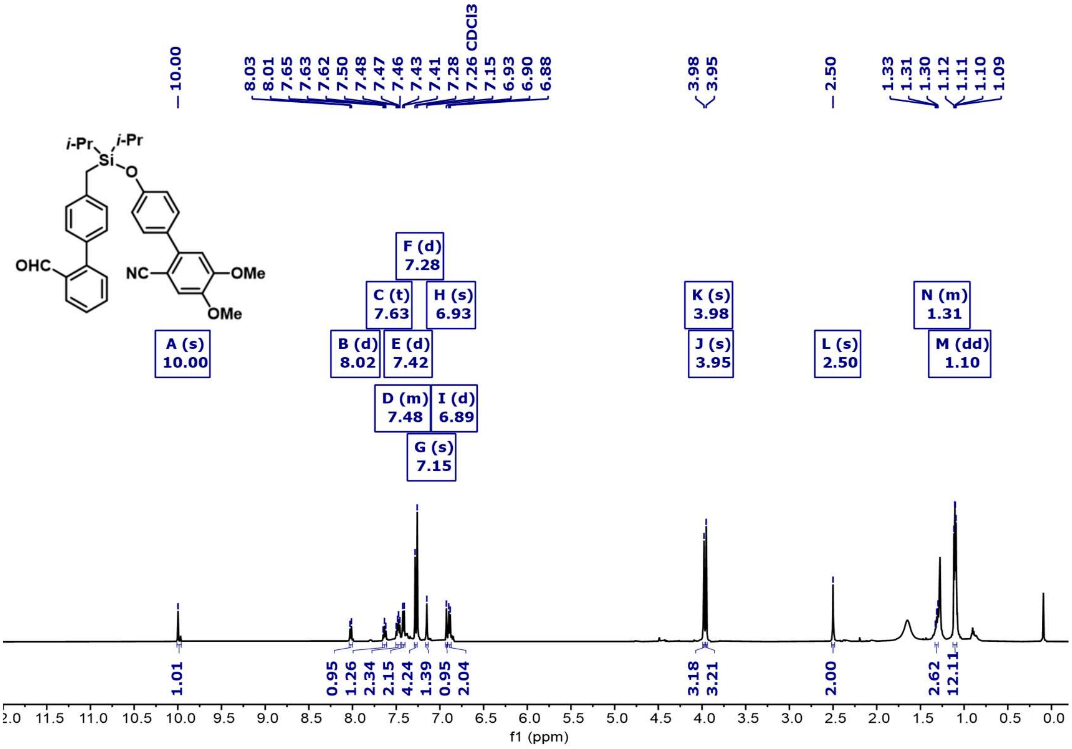

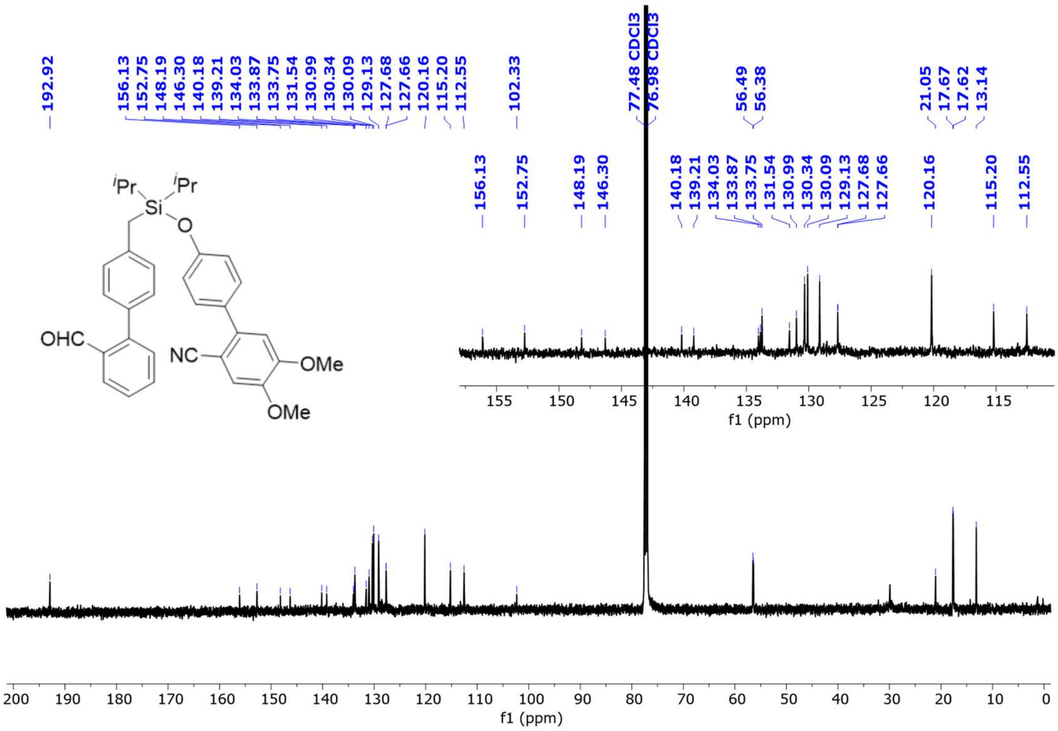

Supplementary Figure 36. ^{1}H (top) and ^{13}C (bottom) NMR of **3ao**

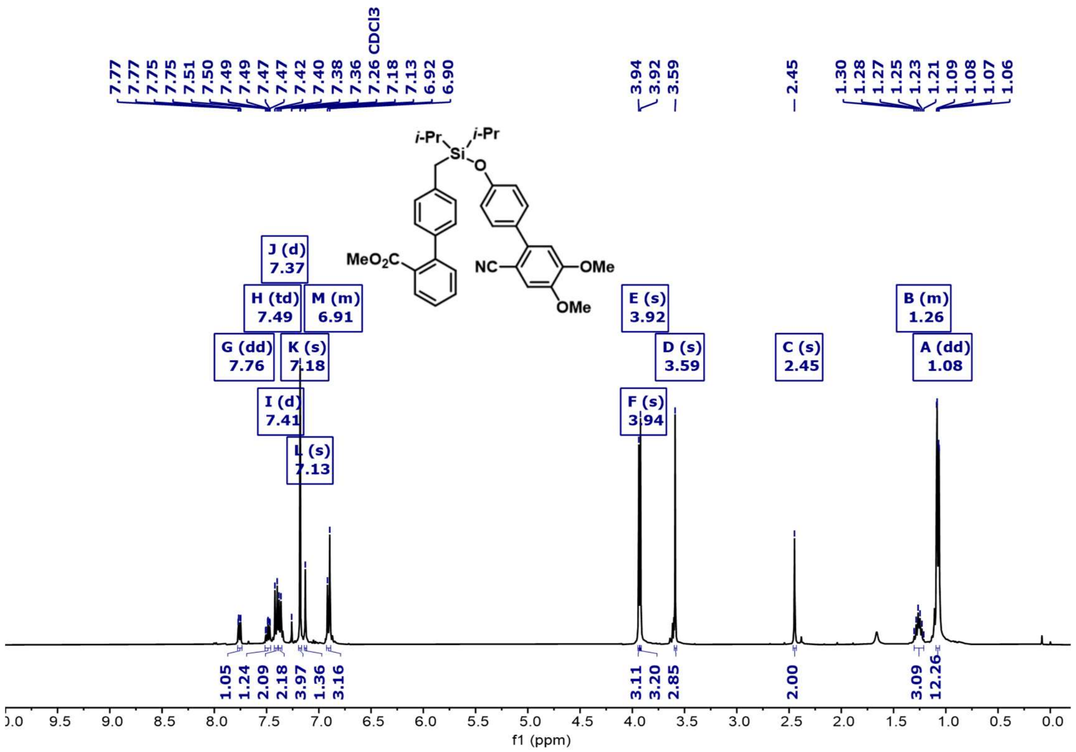

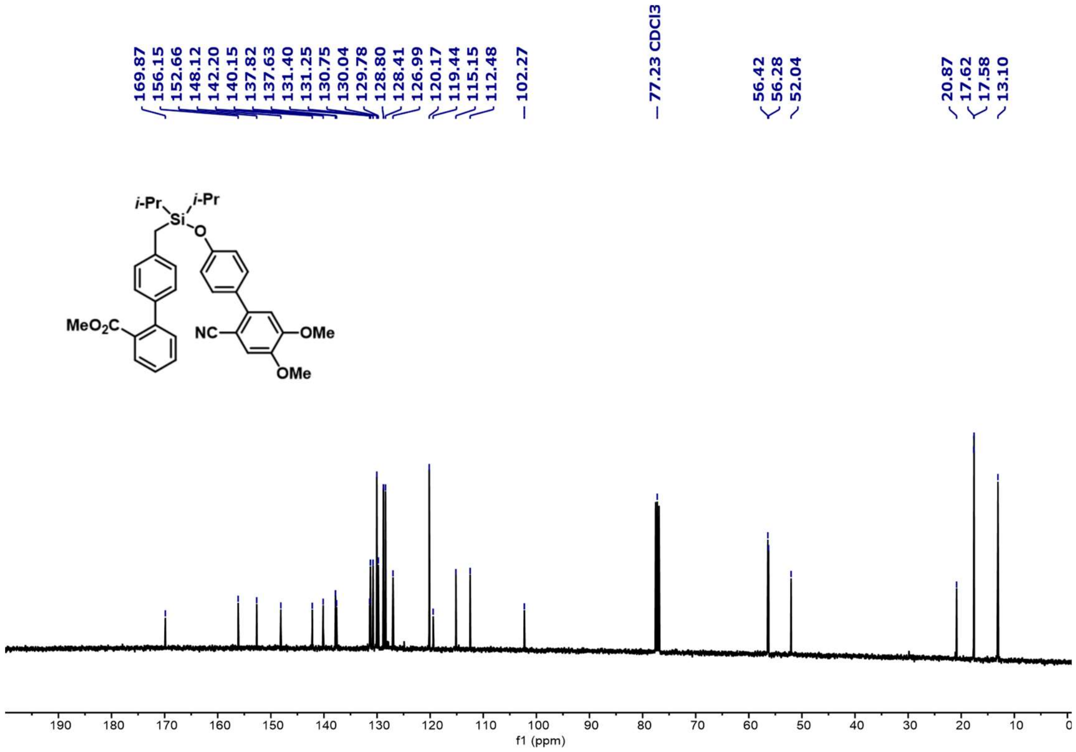

Supplementary Figure 37. ^{1}H (top) and ^{13}C (bottom) NMR of **3ap**

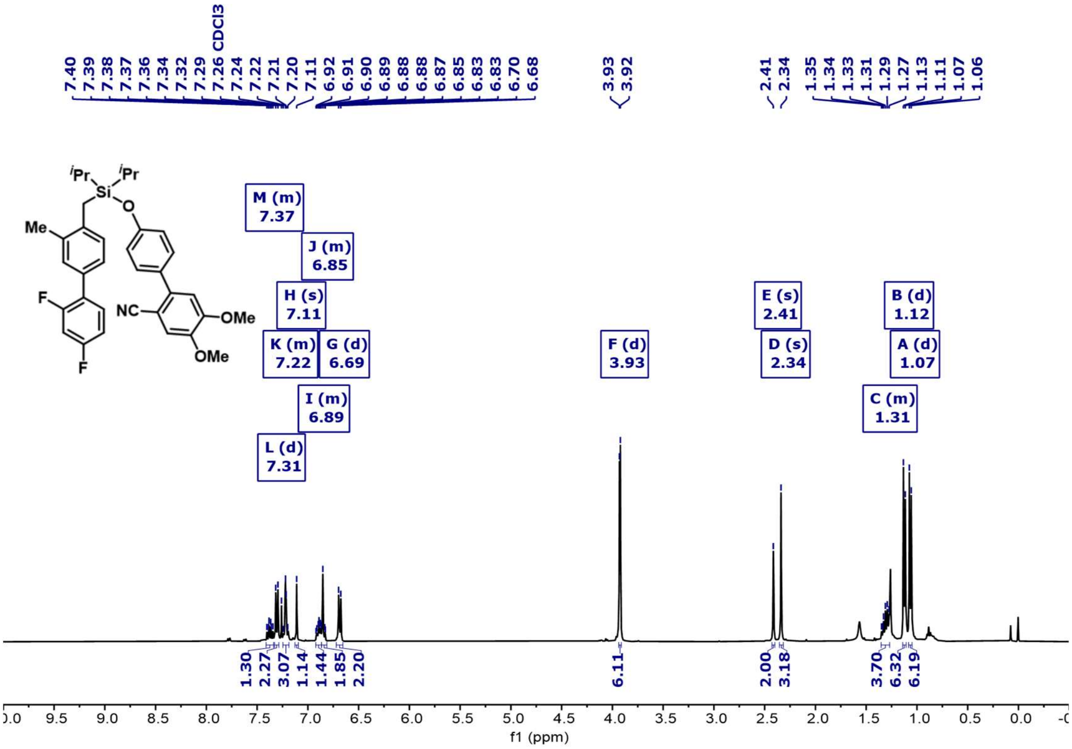

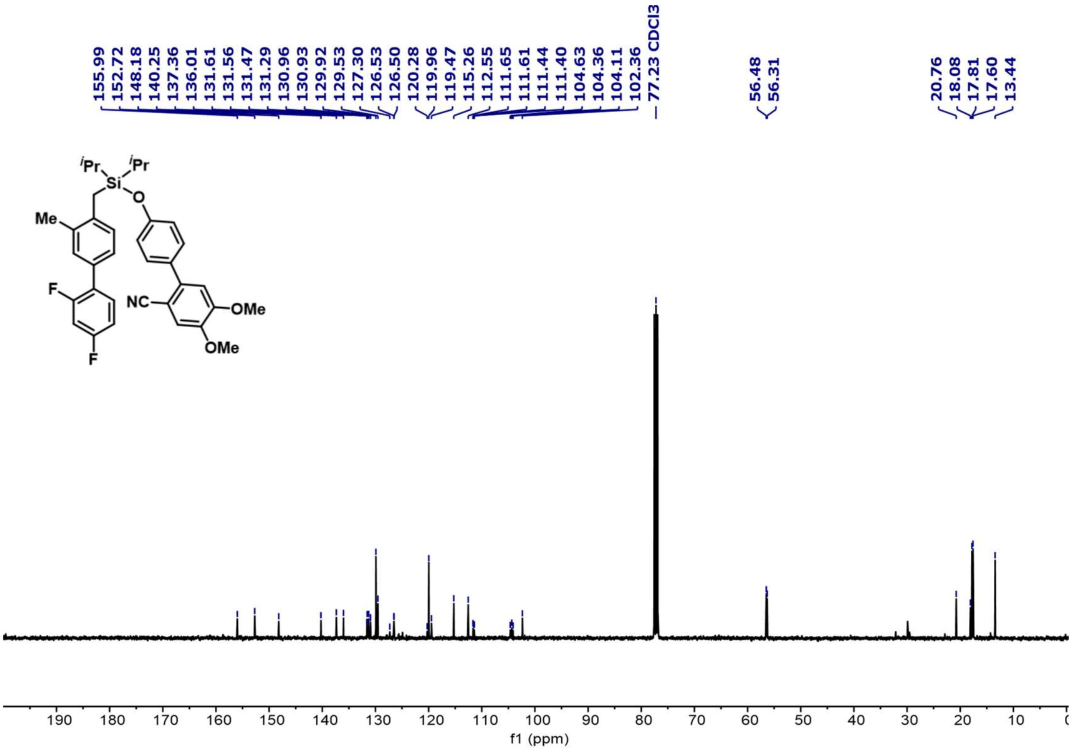

Supplementary Figure 38. ^{1}H (top) and ^{13}C (bottom) NMR of **3bq**

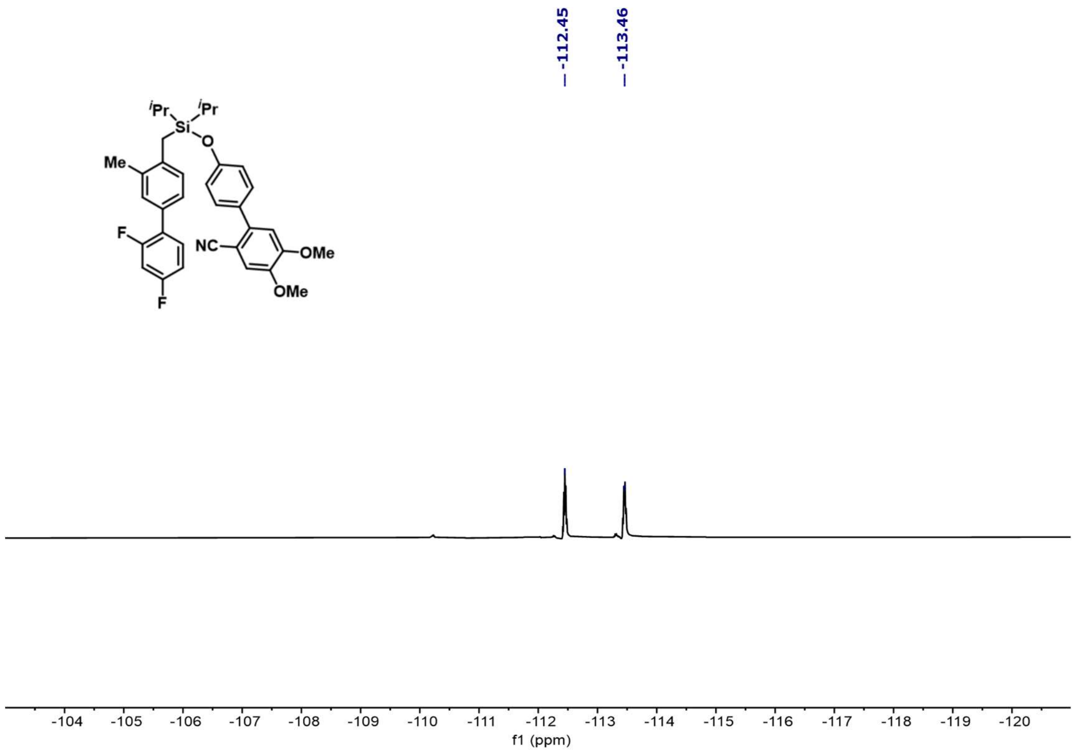

Supplementary Figure 39. ^{19}F NMR of **3bq**

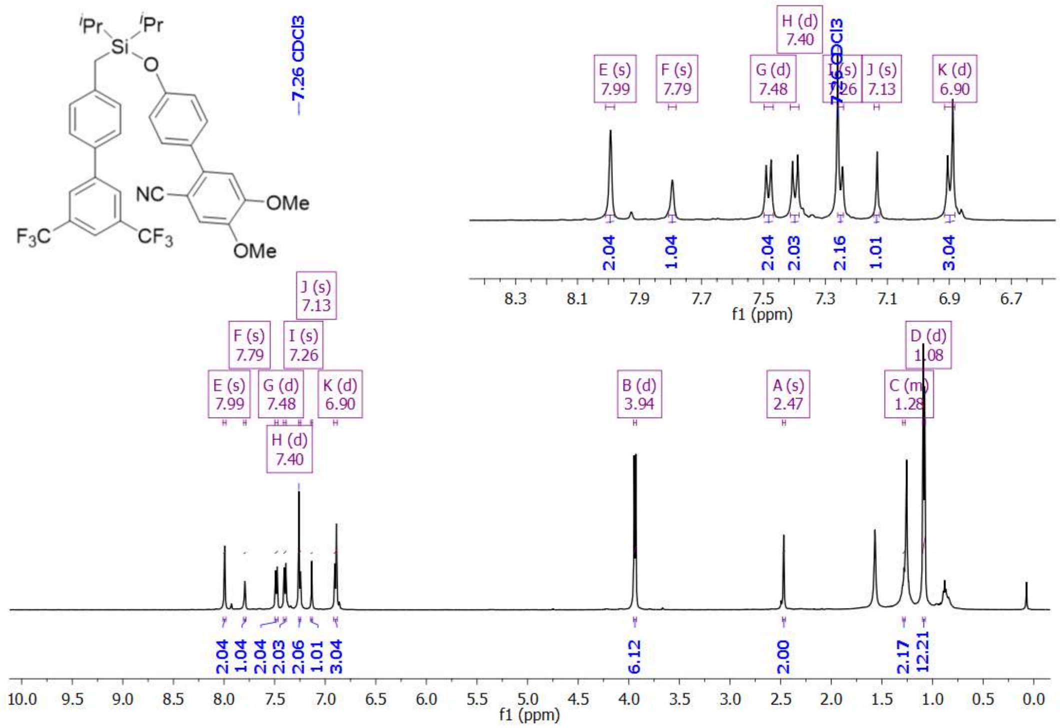

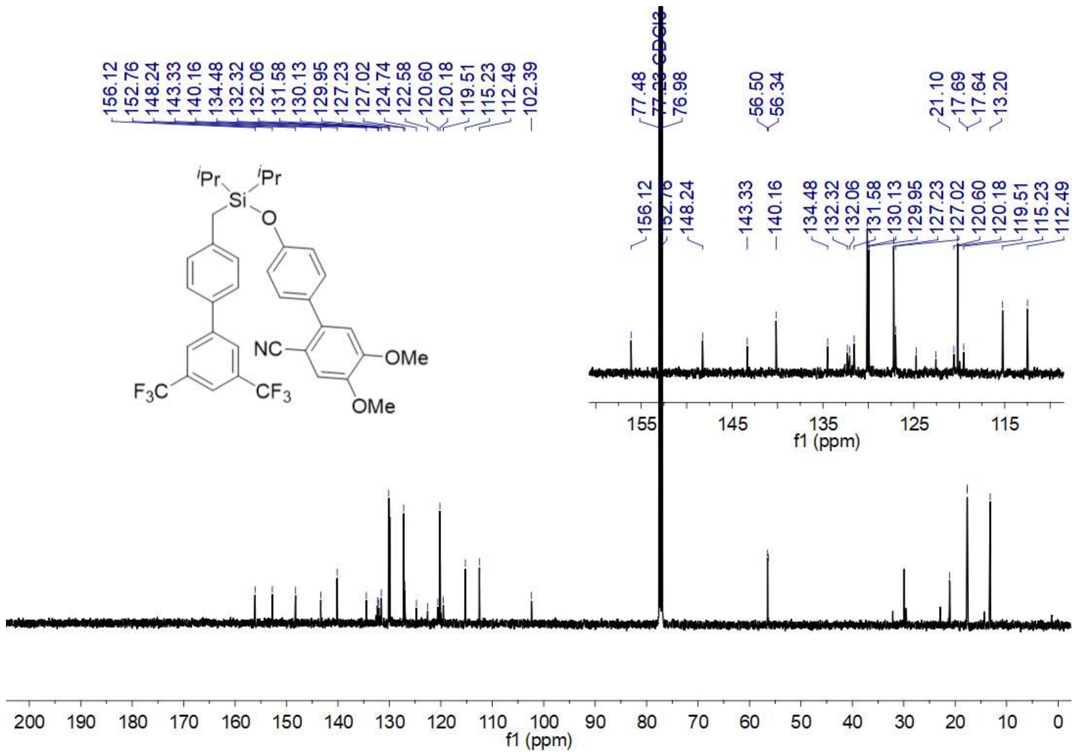

Supplementary Figure 40. ^{1}H (top) and ^{13}C (bottom) NMR of **3ar**

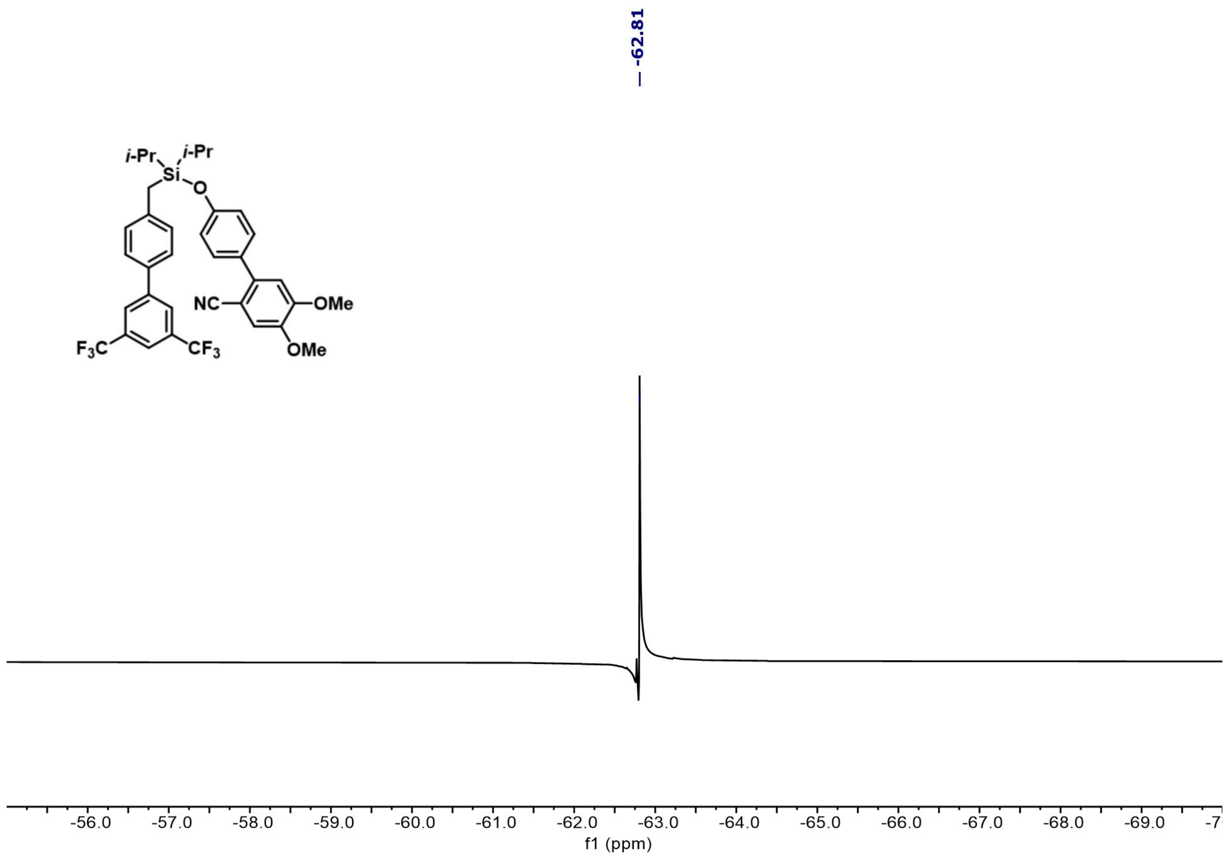

Supplementary Figure 41. ^{19}F NMR of **3ar**

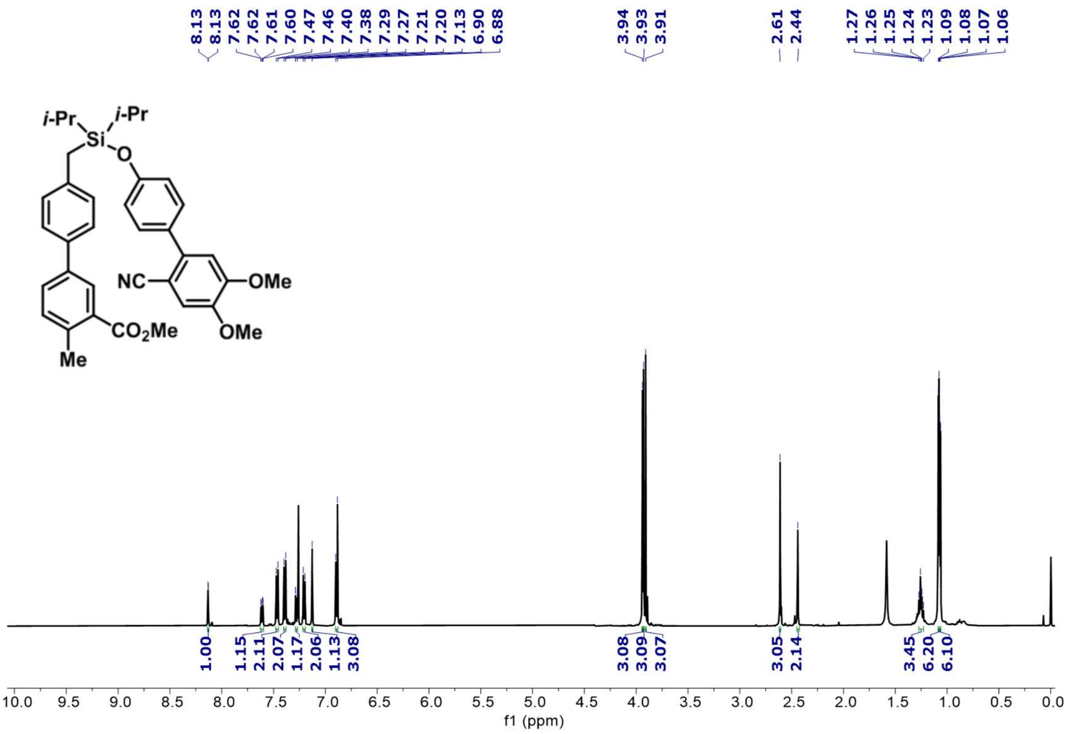

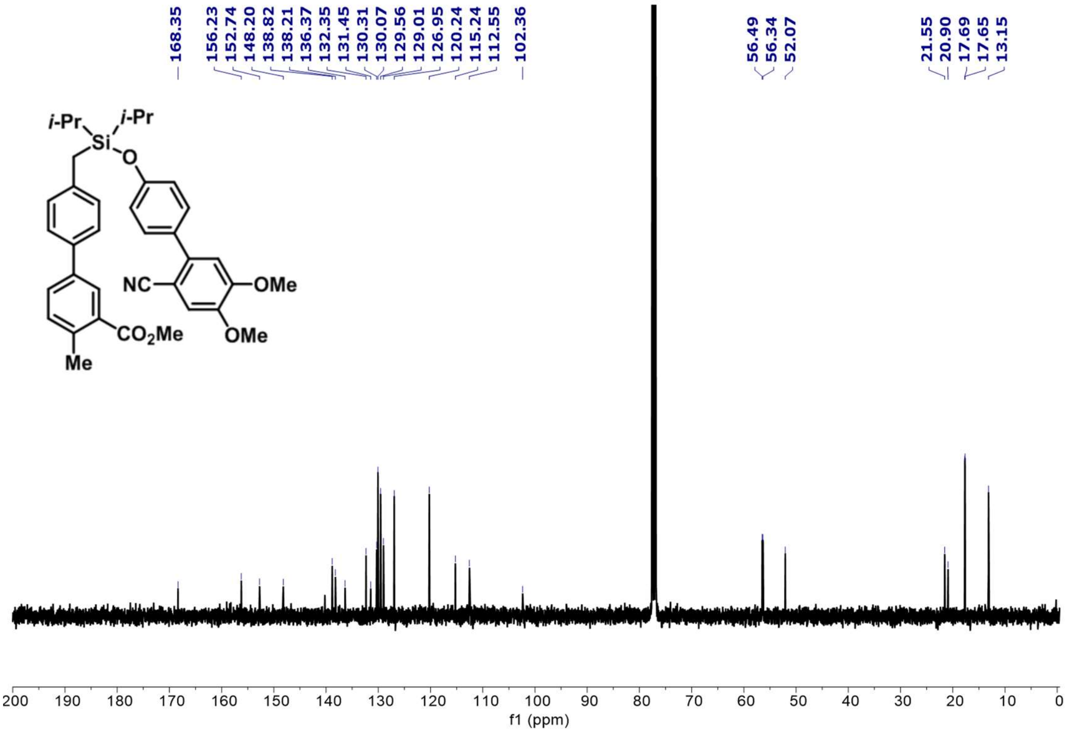

Supplementary Figure 42. ^{1}H (top) and ^{13}C (bottom) NMR of **3as**

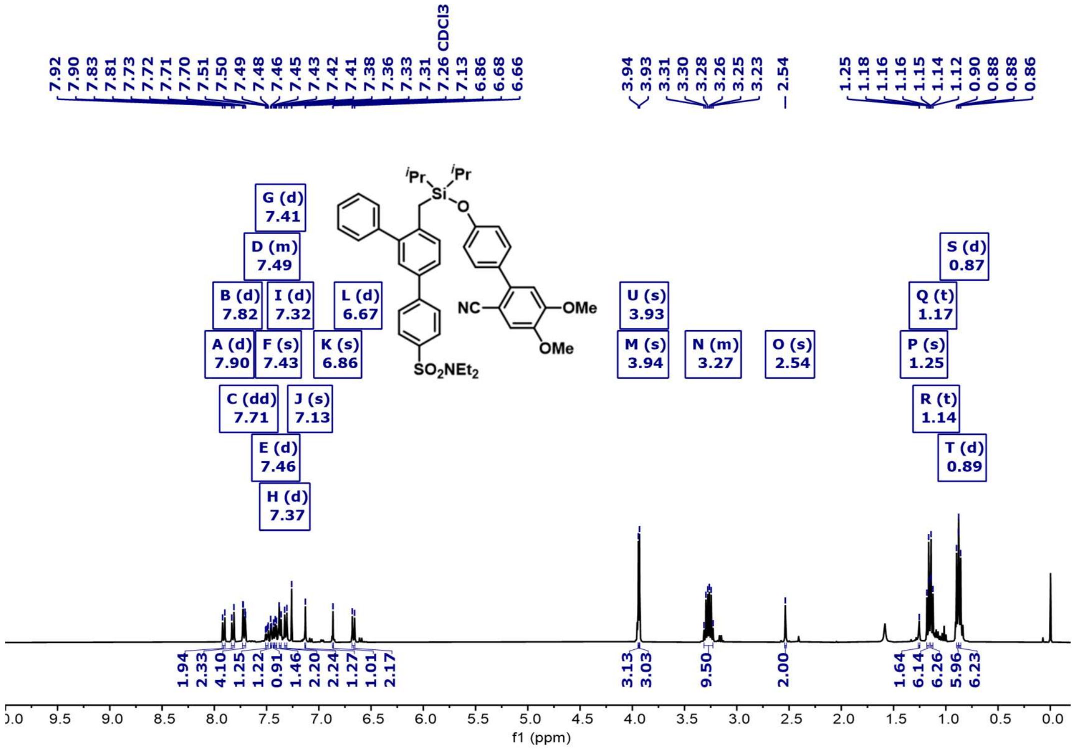

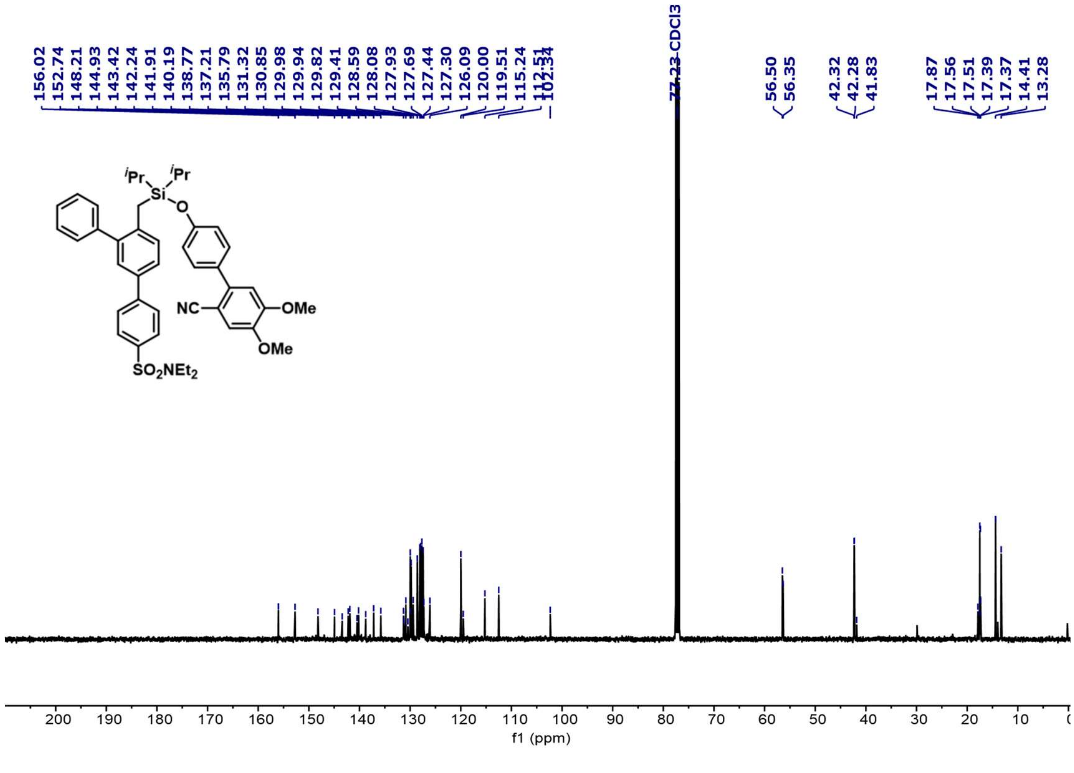

Supplementary Figure 43. ^{1}H (top) and ^{13}C (bottom) NMR of **3kt**

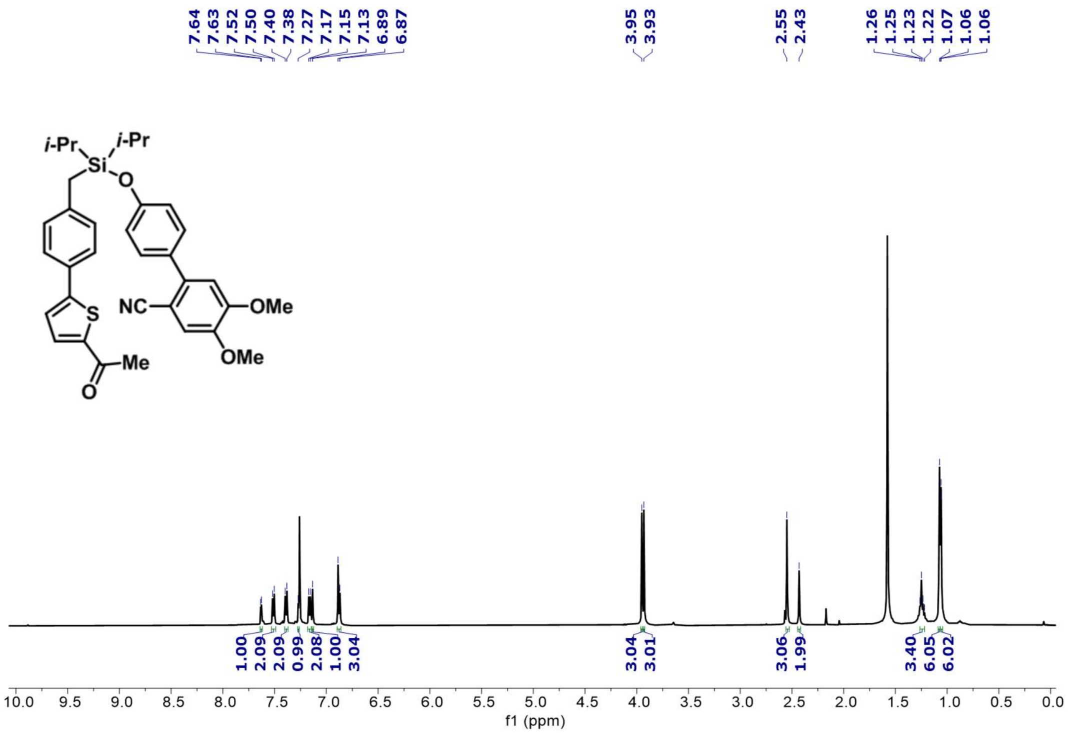

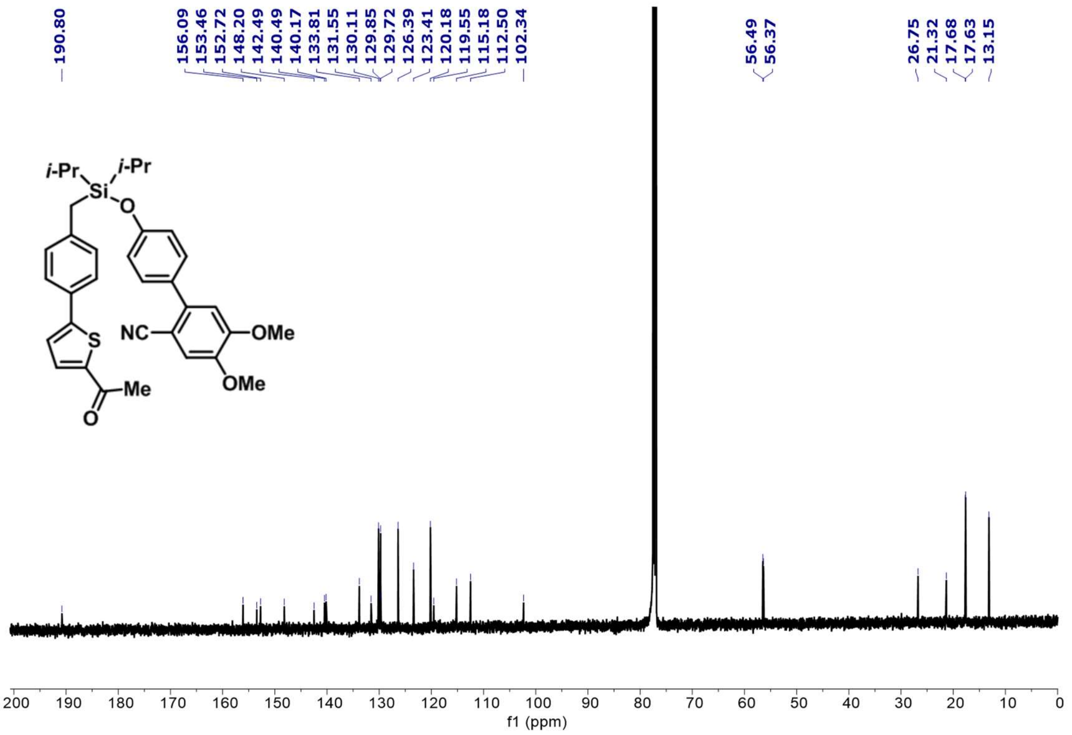

Supplementary Figure 44. ^{1}H (top) and ^{13}C (bottom) NMR of **3au**

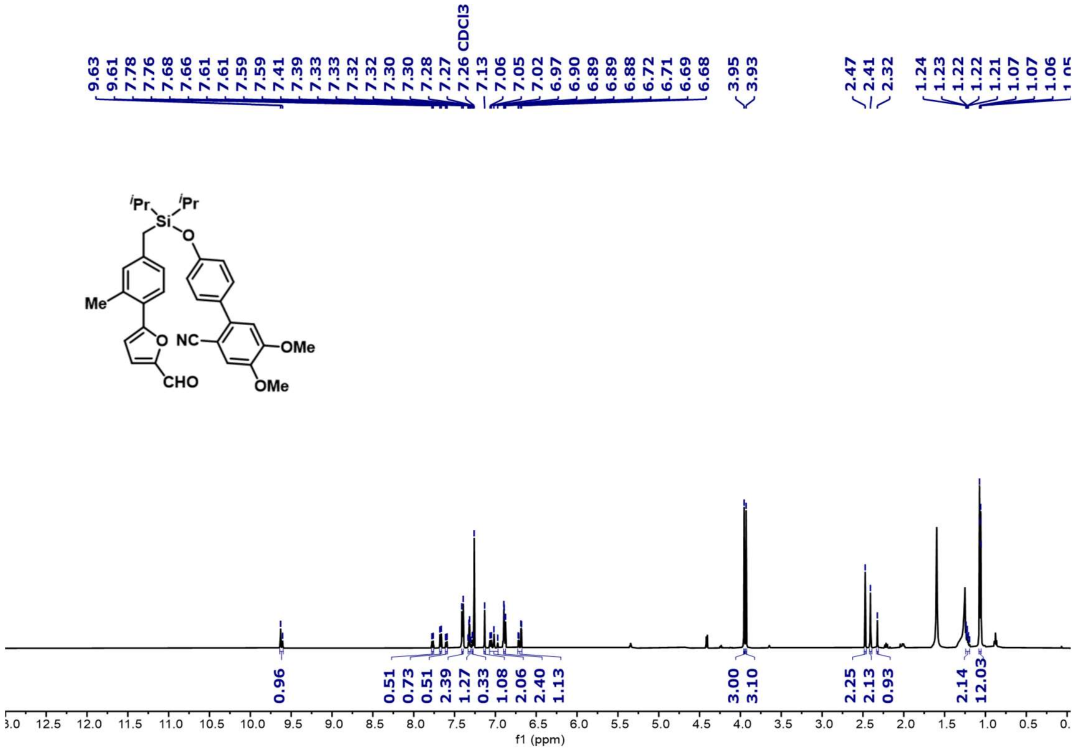

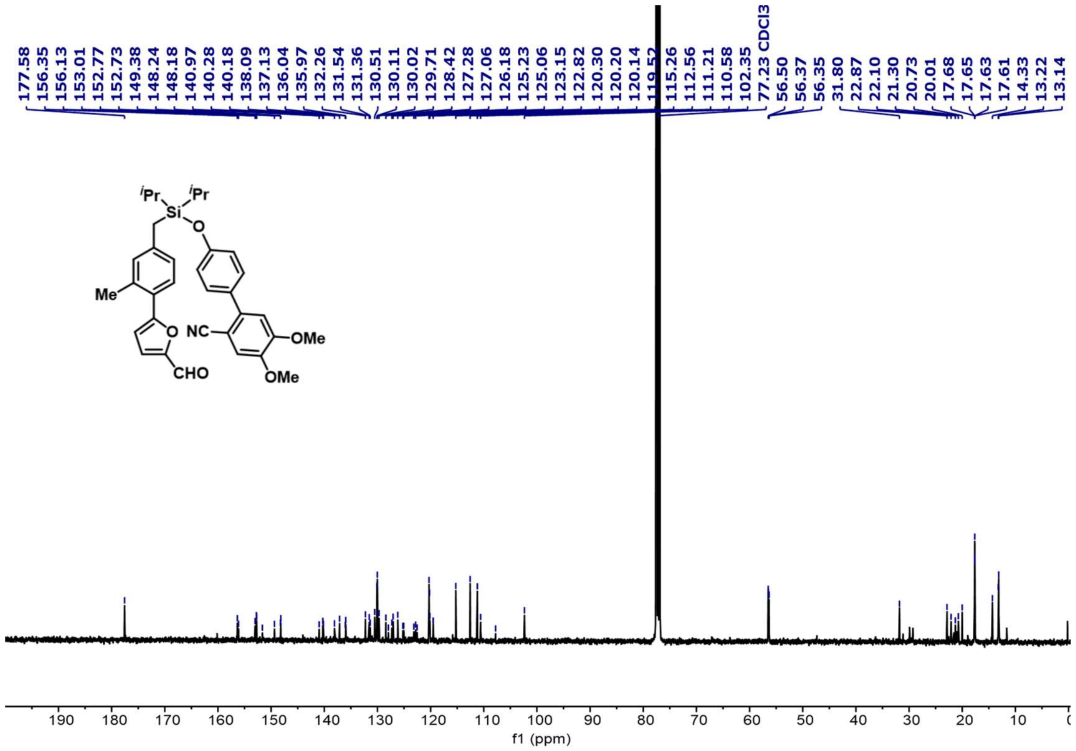

Supplementary Figure 45. ^{1}H (top) and ^{13}C (bottom) NMR of **3gv**

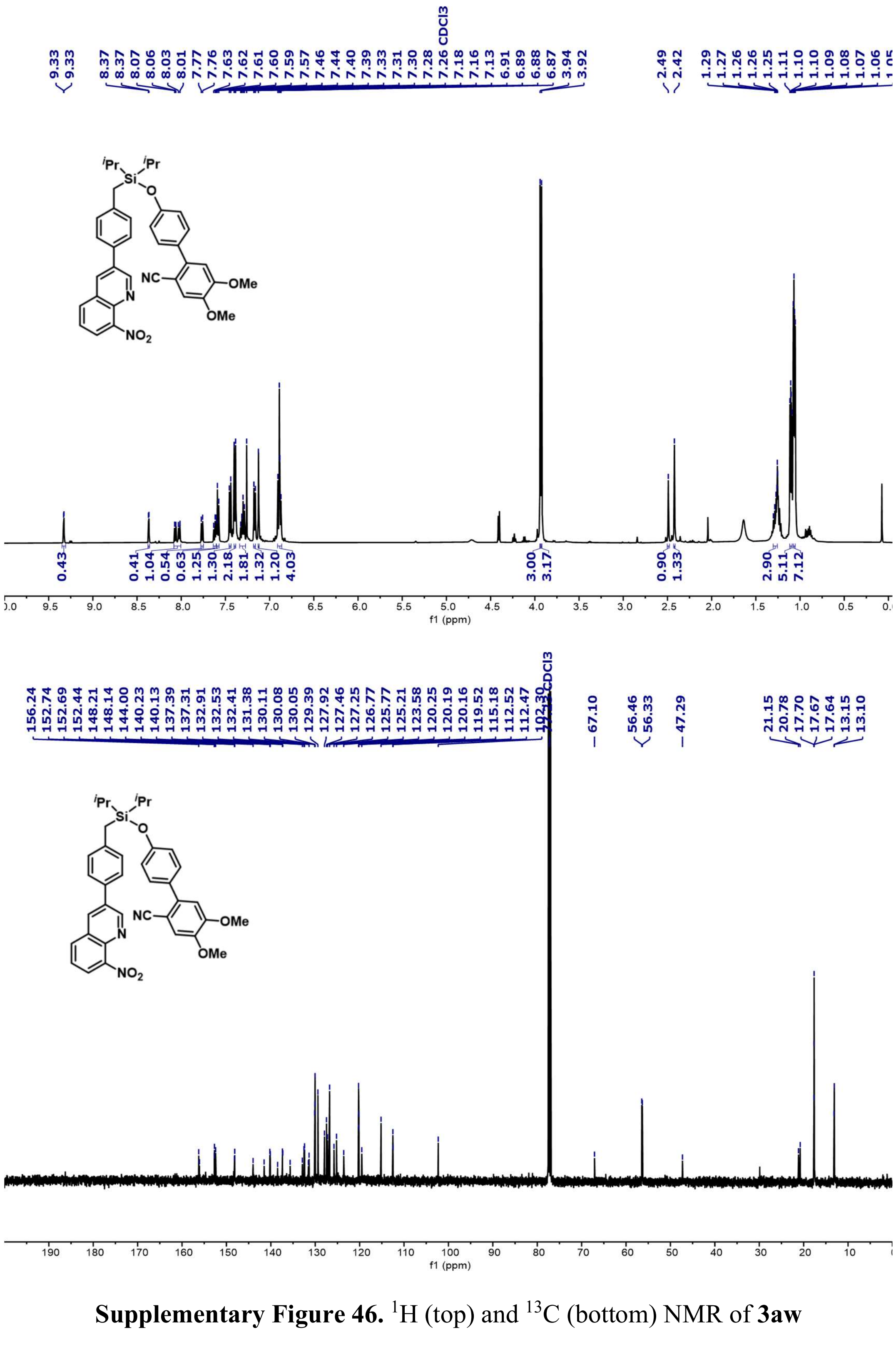

Supplementary Figure 46. ^{1}H (top) and ^{13}C (bottom) NMR of **3aw**

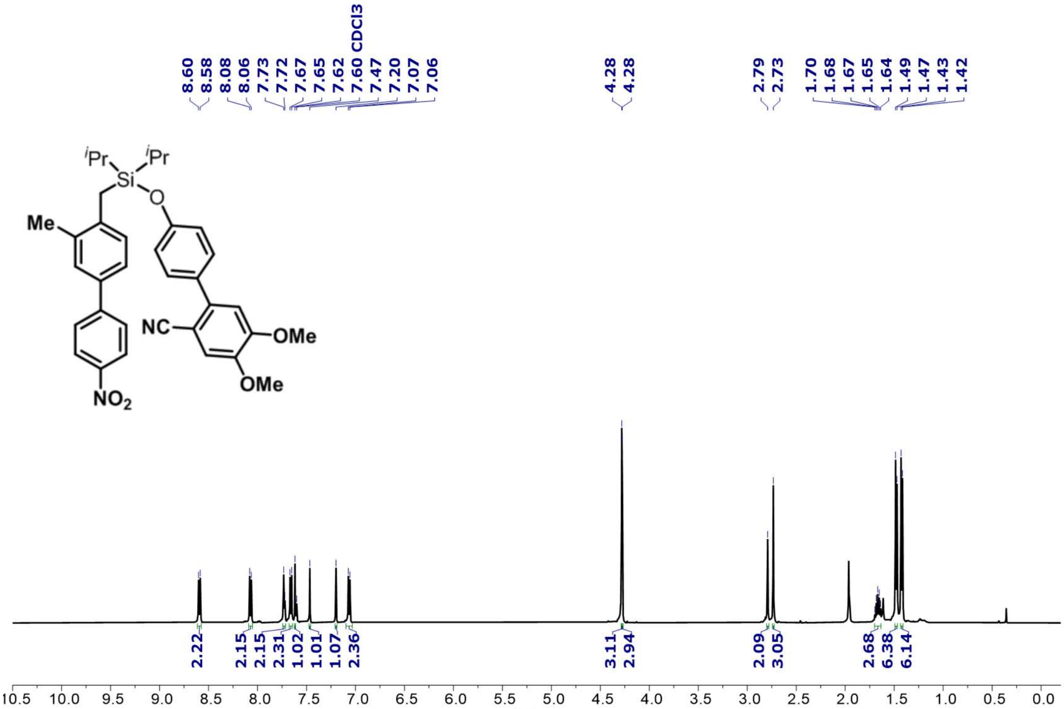

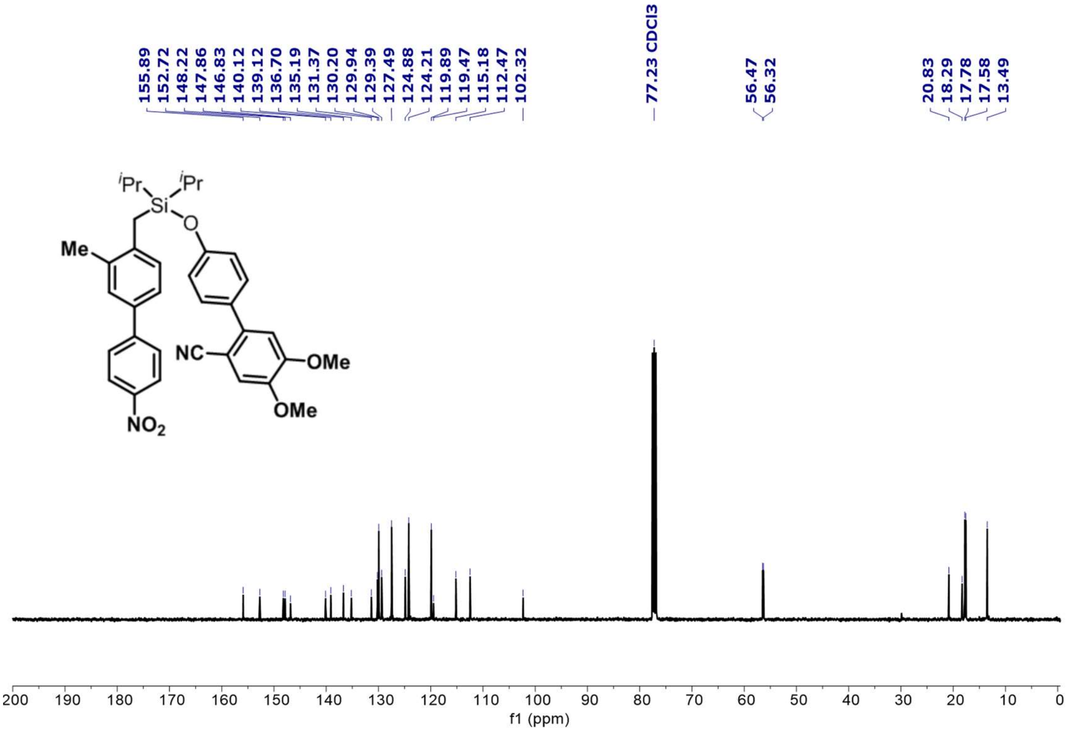

Supplementary Figure 47. ^{1}H (top) and ^{13}C (bottom) NMR of **3bm**

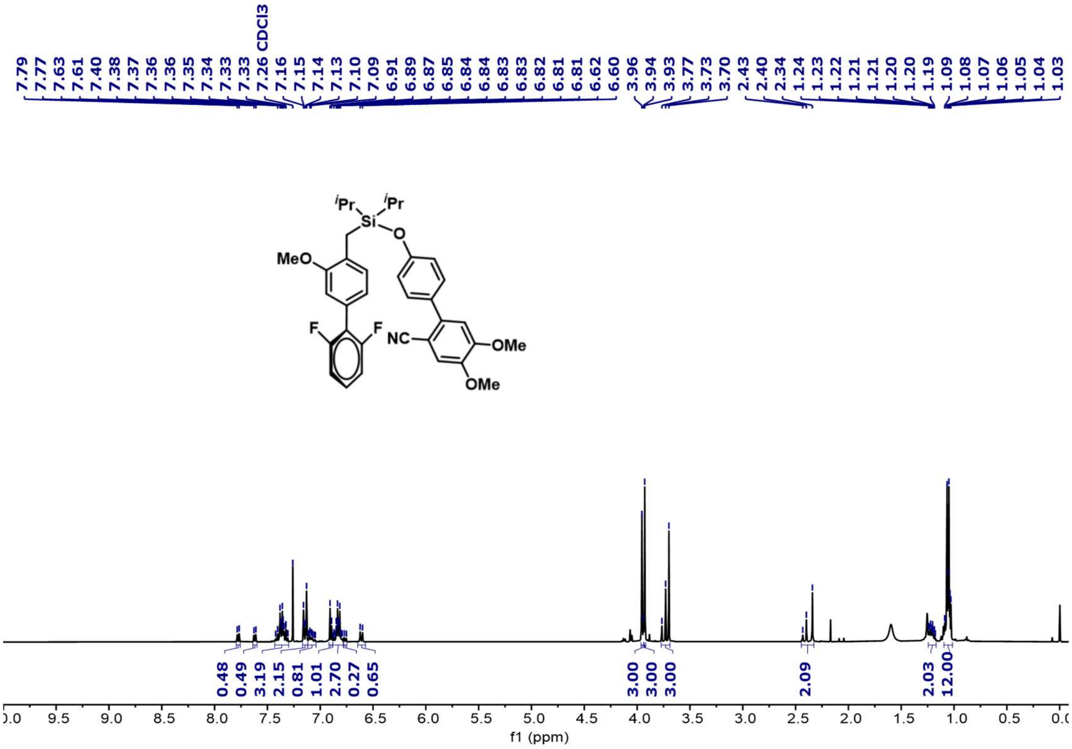

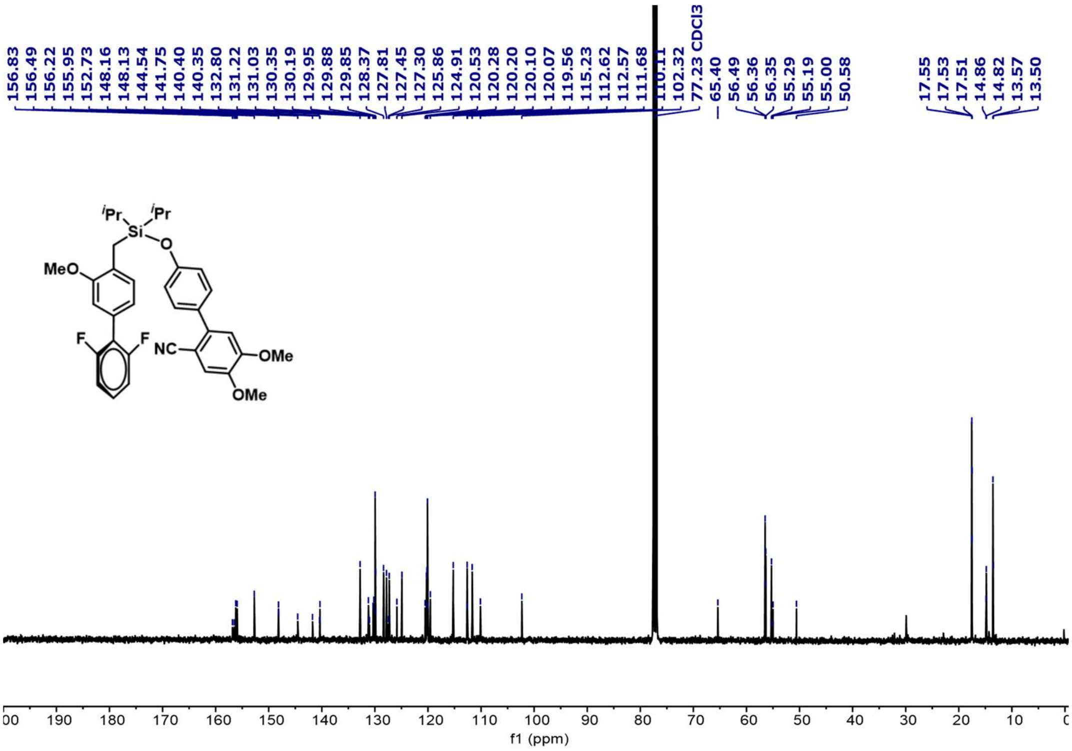

Supplementary Figure 48. ^{1}H (top) and ^{13}C (bottom) NMR of **3cx**

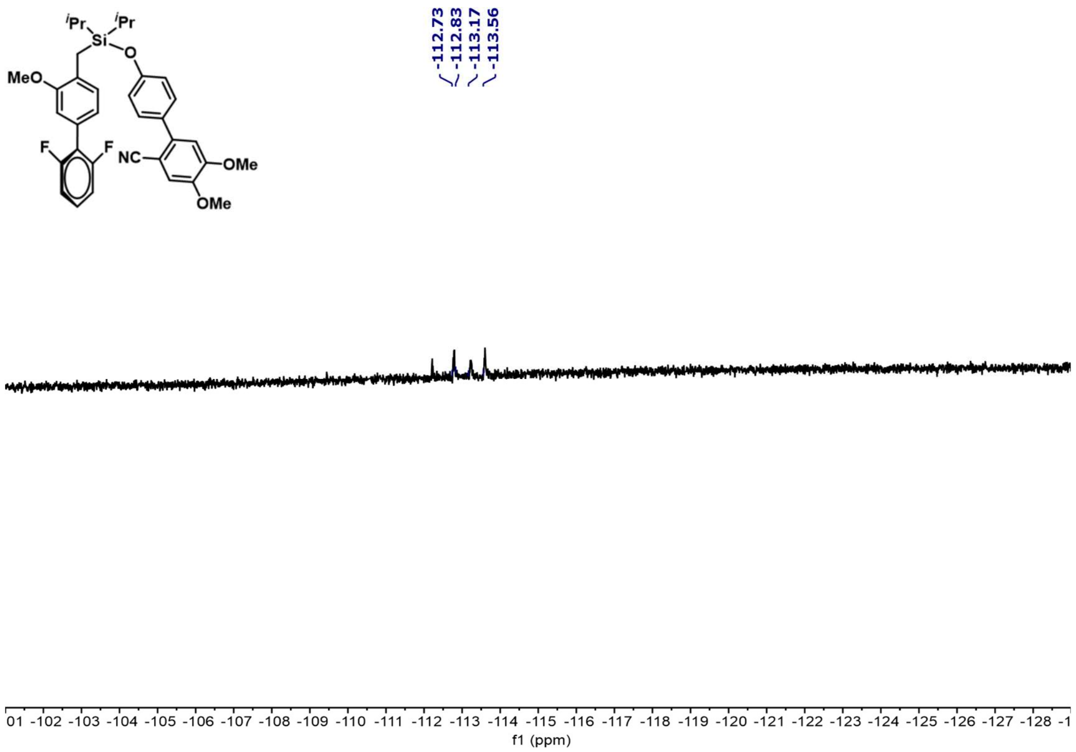

Supplementary Figure 49. ^{19}F NMR of **3cx**

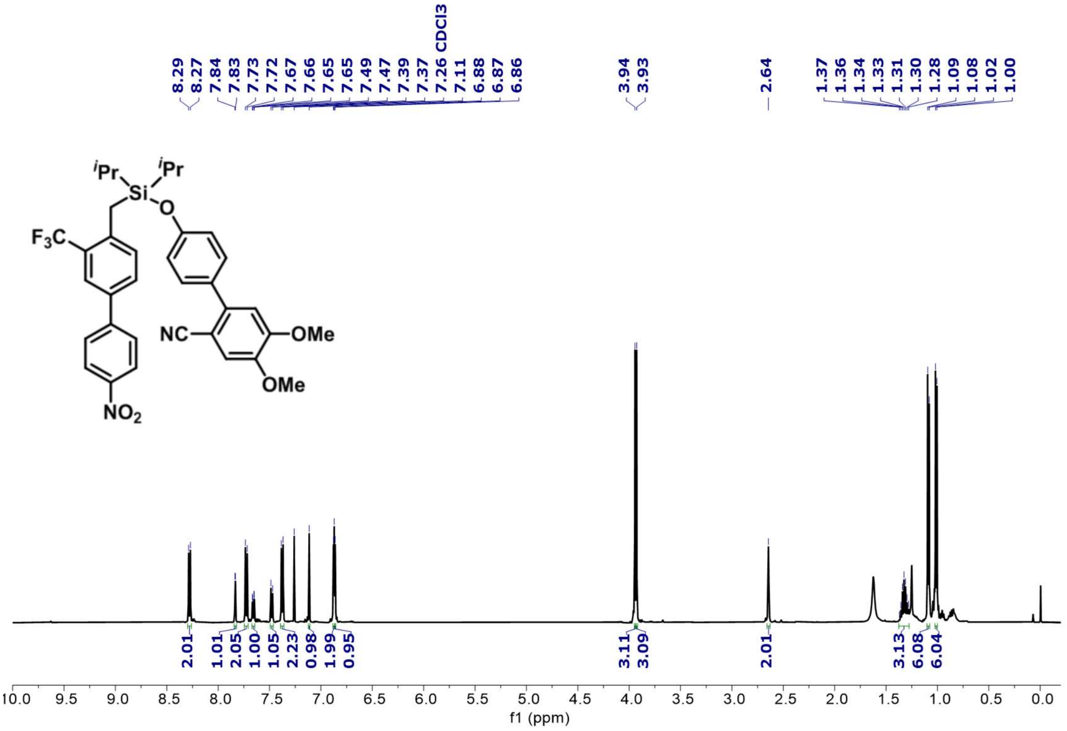

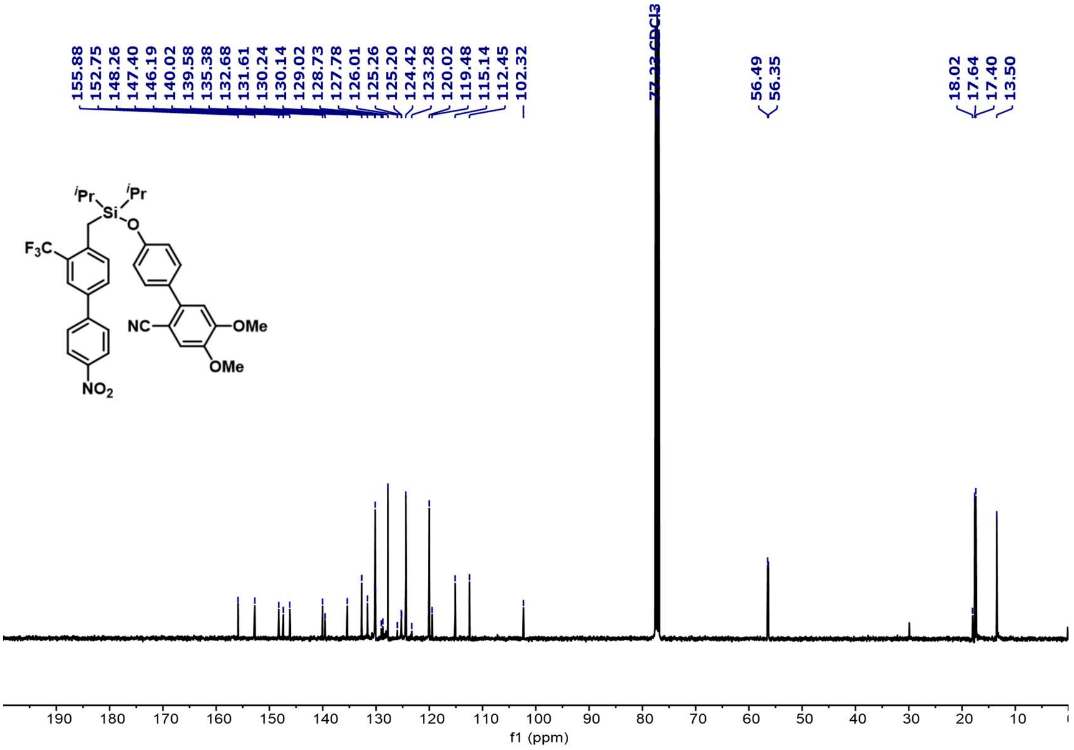

Supplementary Figure 50. ^{1}H (top) and ^{13}C (bottom) NMR of **3dm**

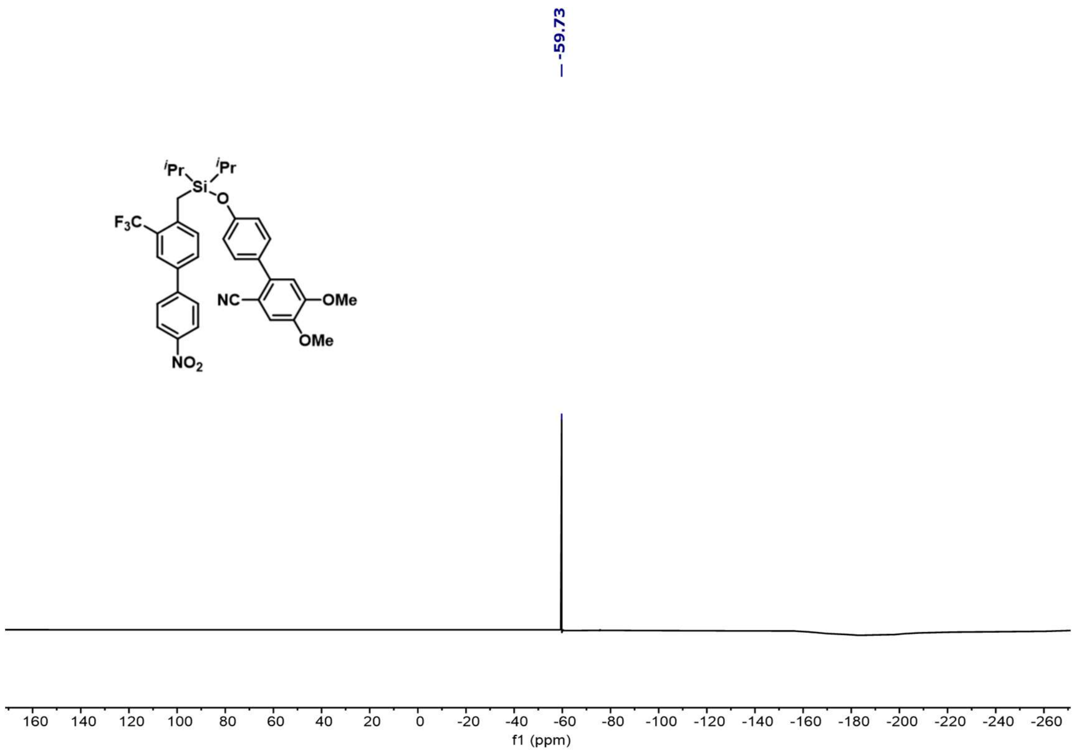

Supplementary Figure 51. ^{19}F NMR of **3dm**

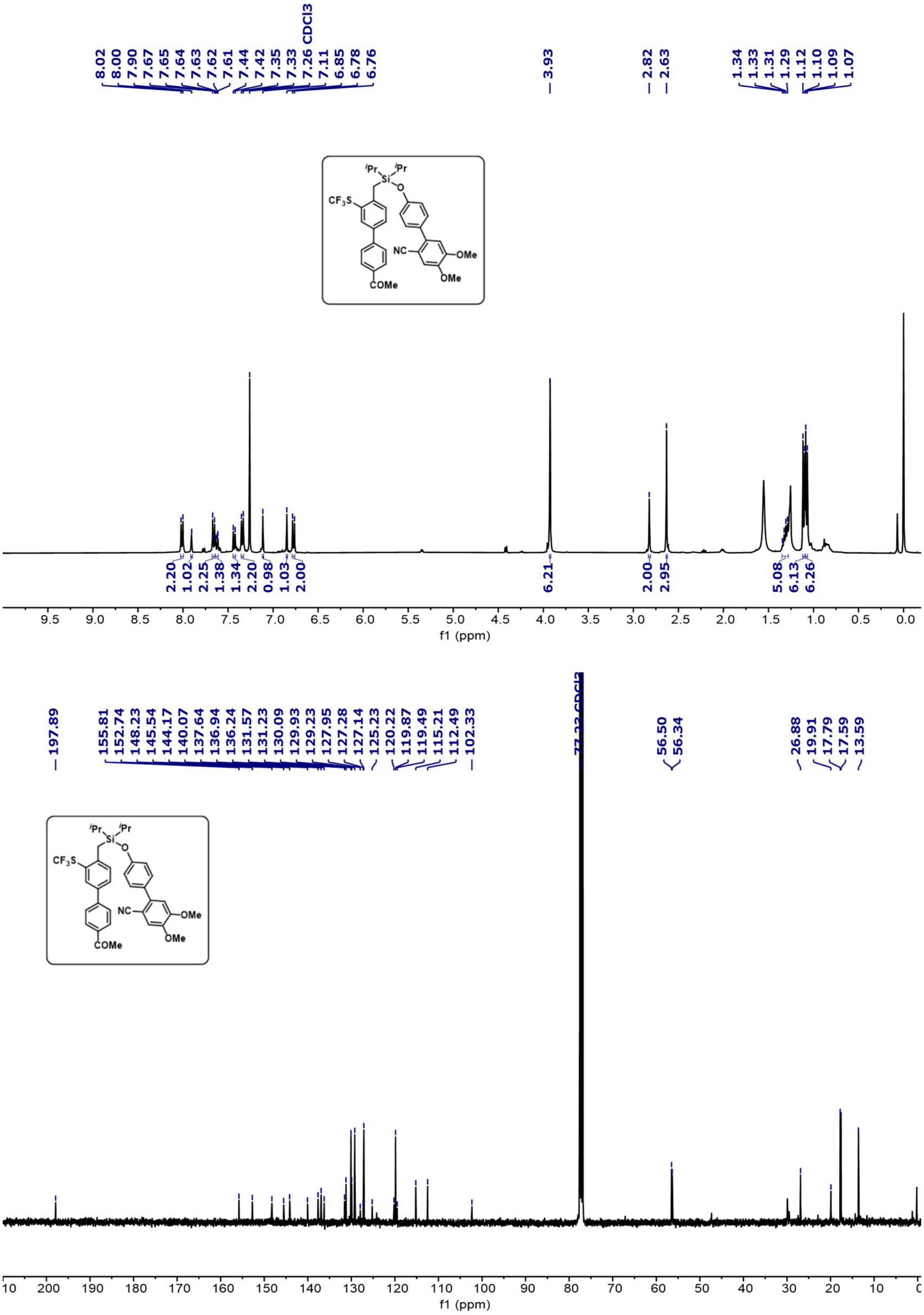

Supplementary Figure 52. ^{1}H (top) and ^{13}C (bottom) NMR of **3el**

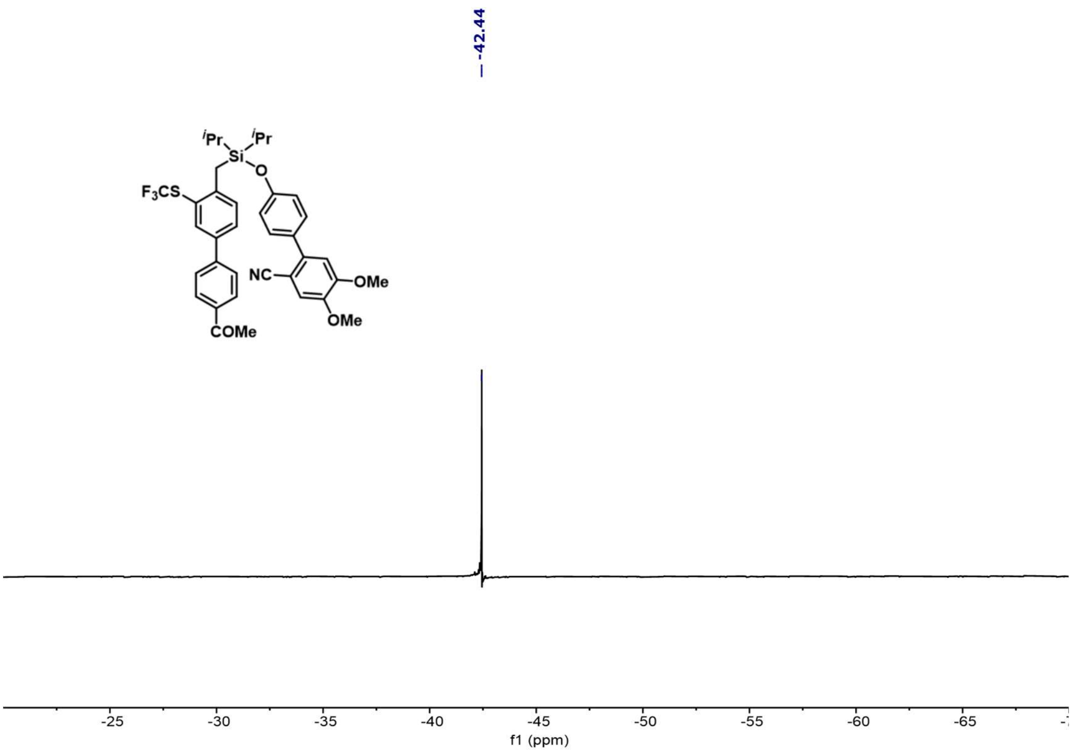

Supplementary Figure 53. ^{19}F NMR of **3em**

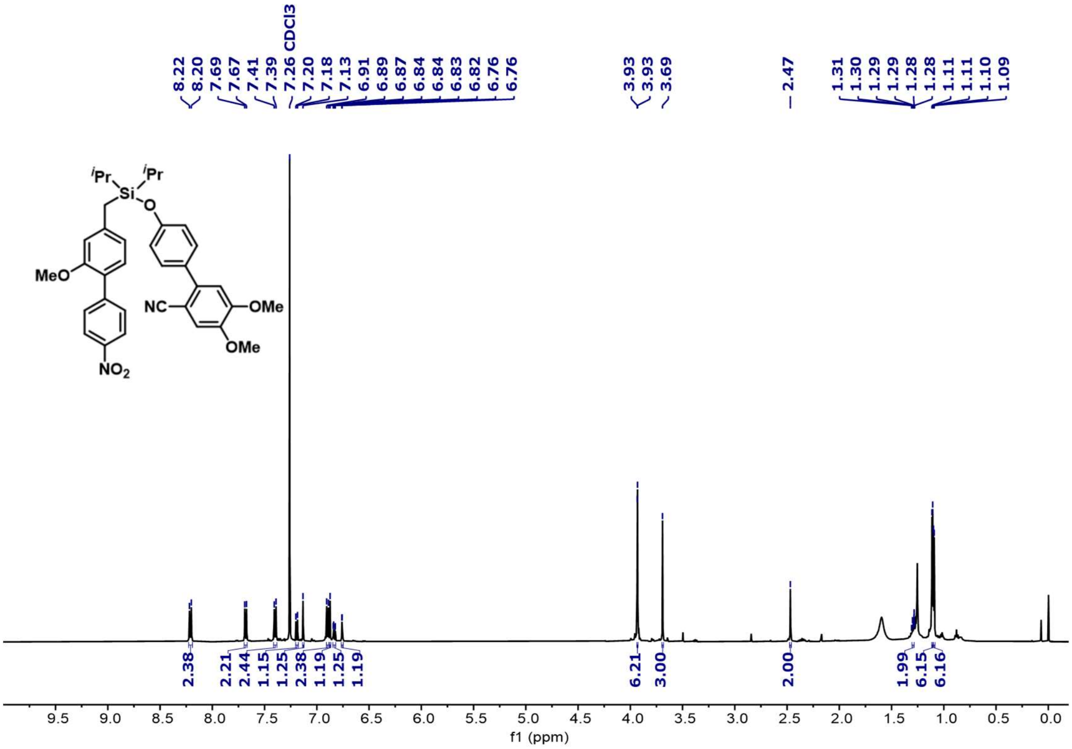

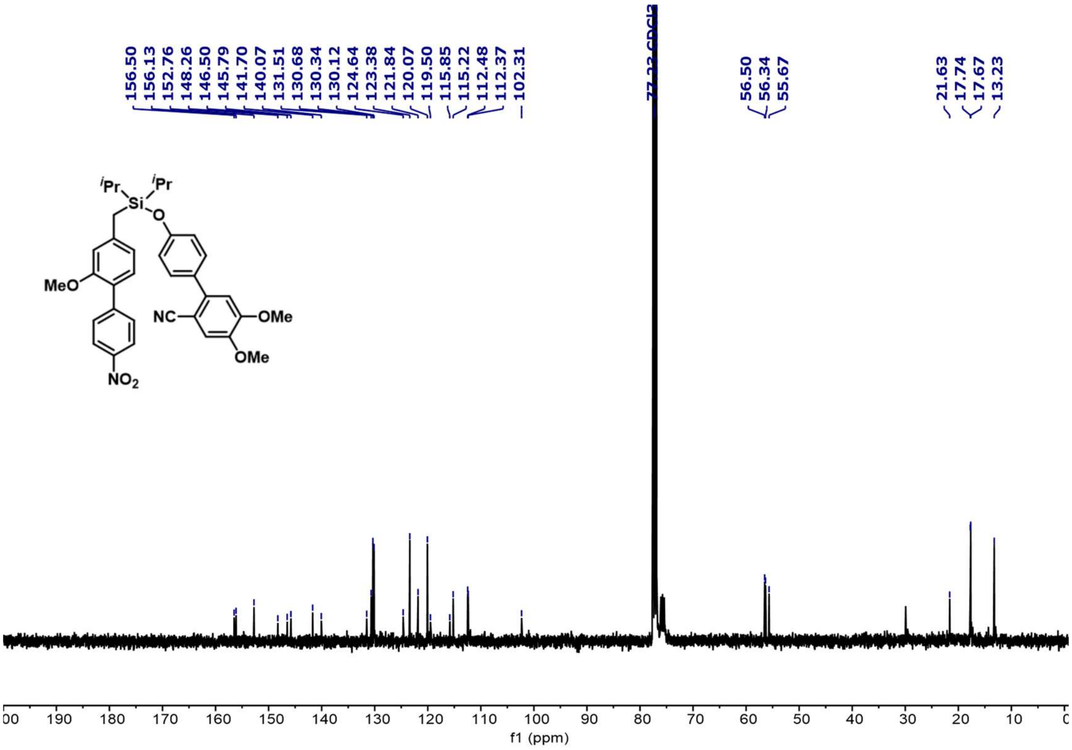

Supplementary Figure 54. ^{1}H (top) and ^{13}C (bottom) NMR of **3fm**

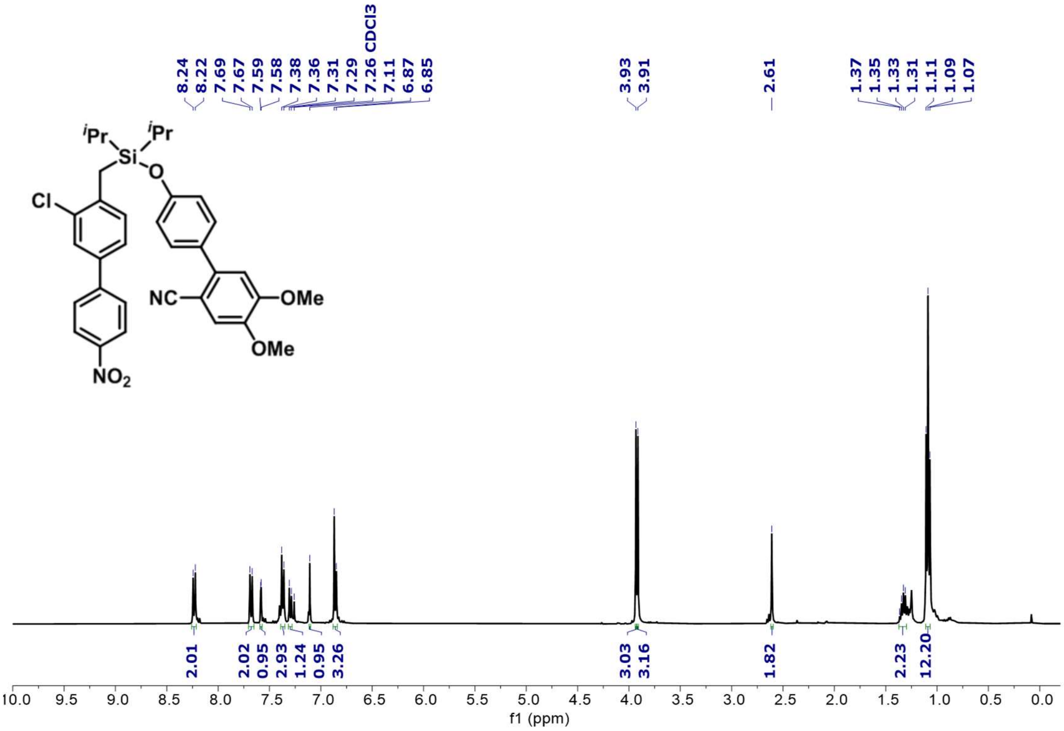

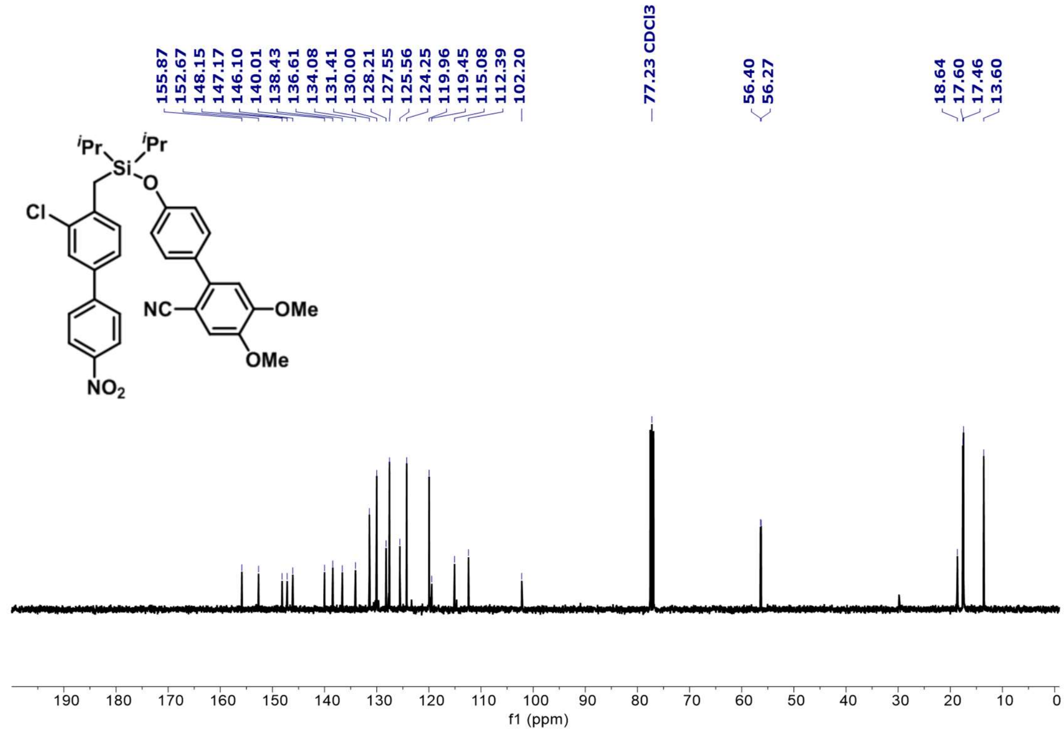

Supplementary Figure 55. ^{1}H (top) and ^{13}C (bottom) NMR of **3hm**

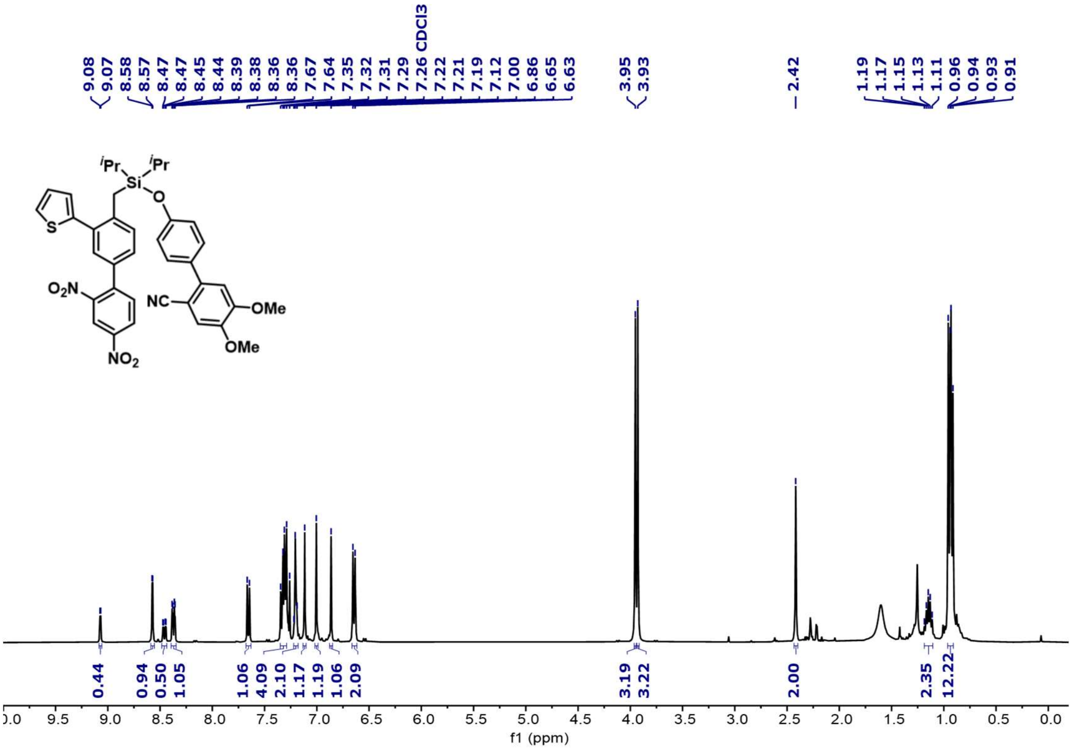

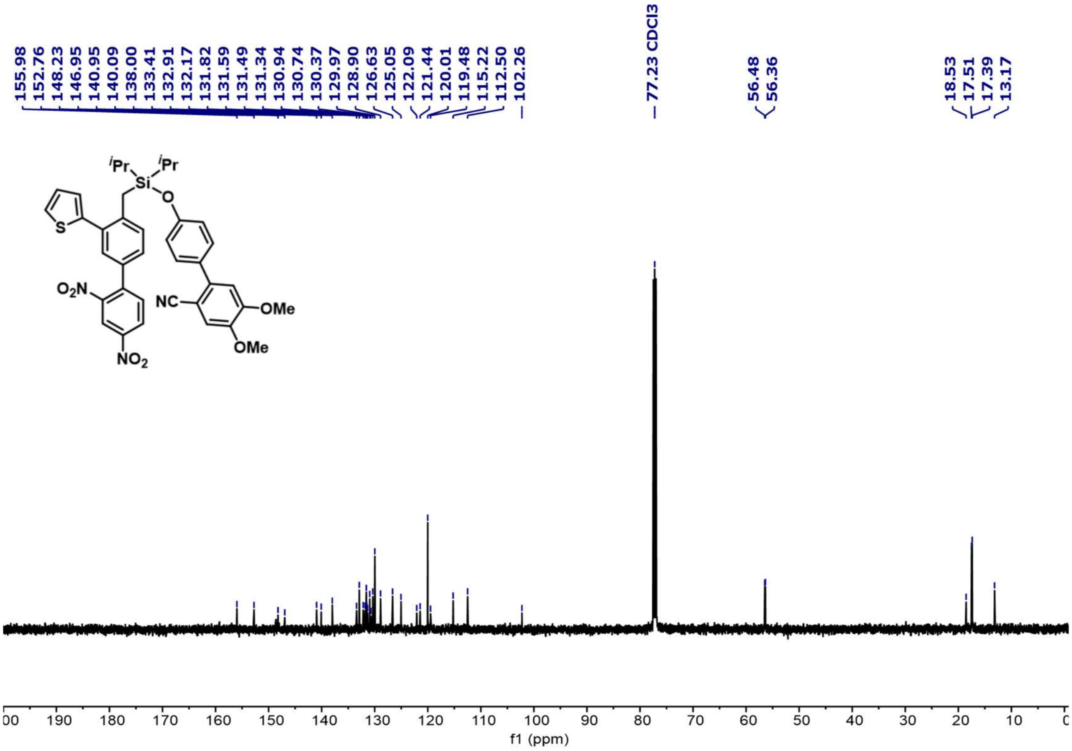

Supplementary Figure 56. ^{1}H (top) and ^{13}C (bottom) NMR of **3iy**

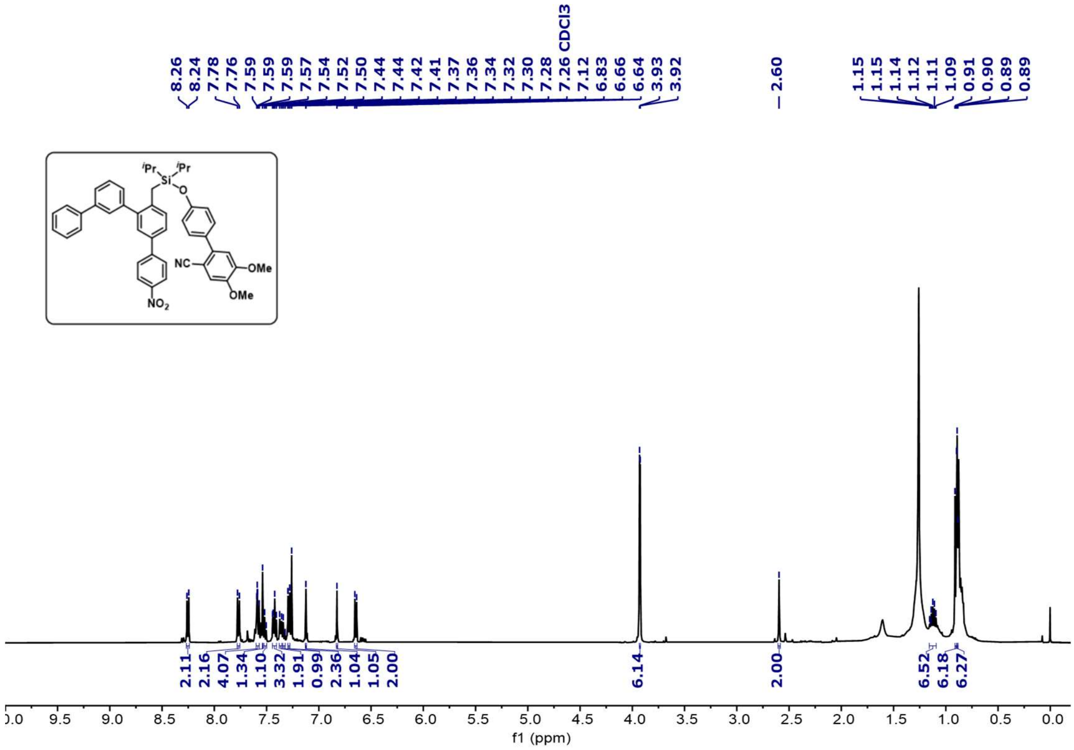

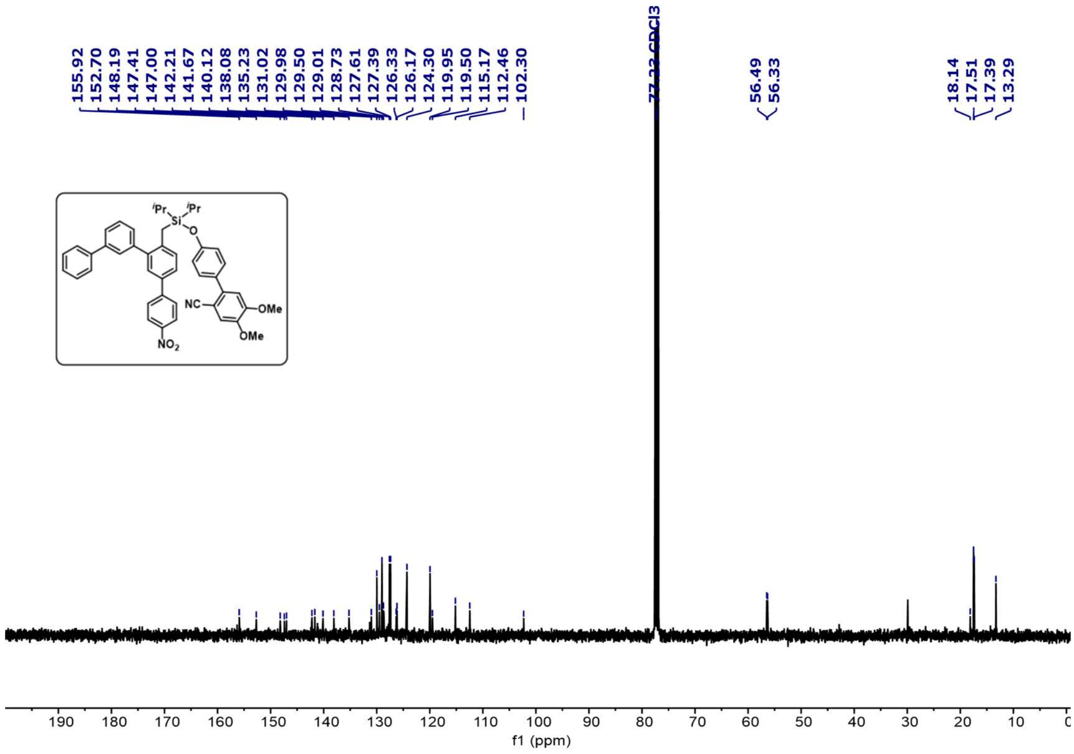

Supplementary Figure 57. ^{1}H (top) and ^{13}C (bottom) NMR of **3jm**

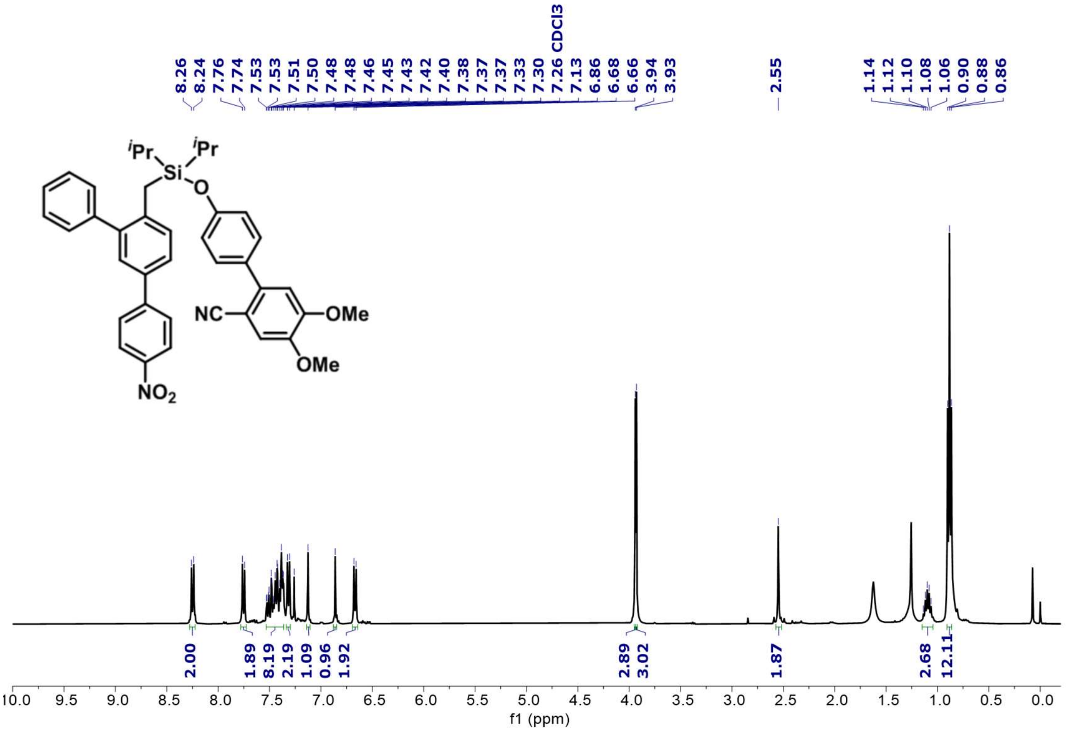

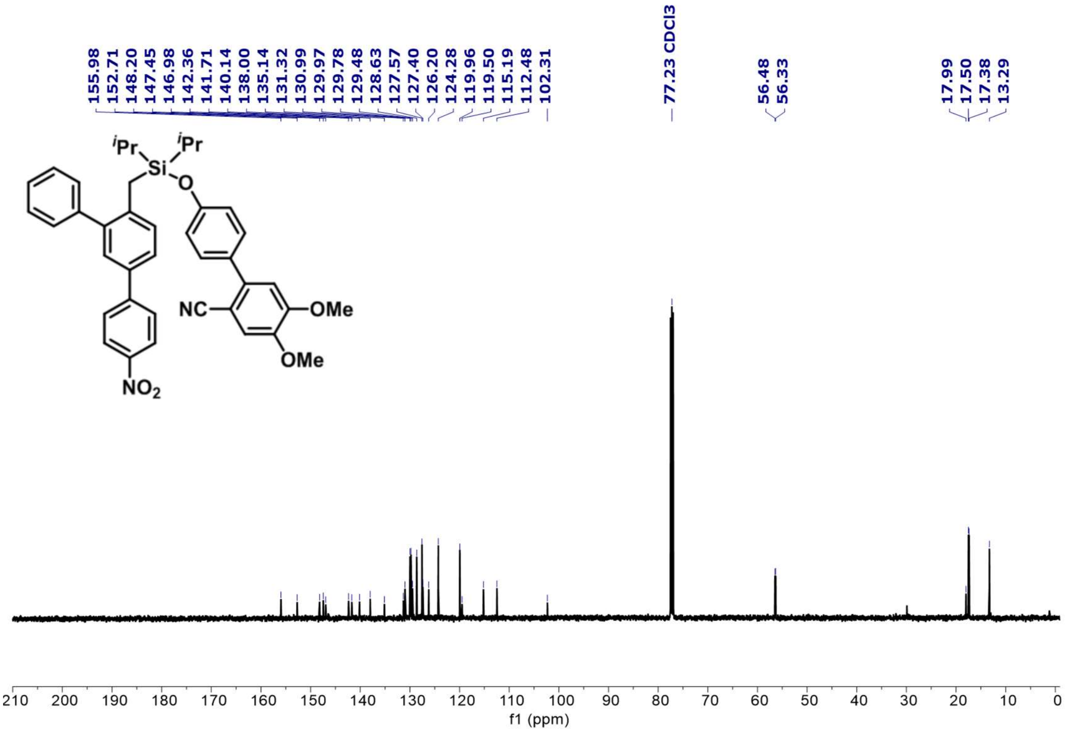

Supplementary Figure 58. ^{1}H (top) and ^{13}C (bottom) NMR of **3km**

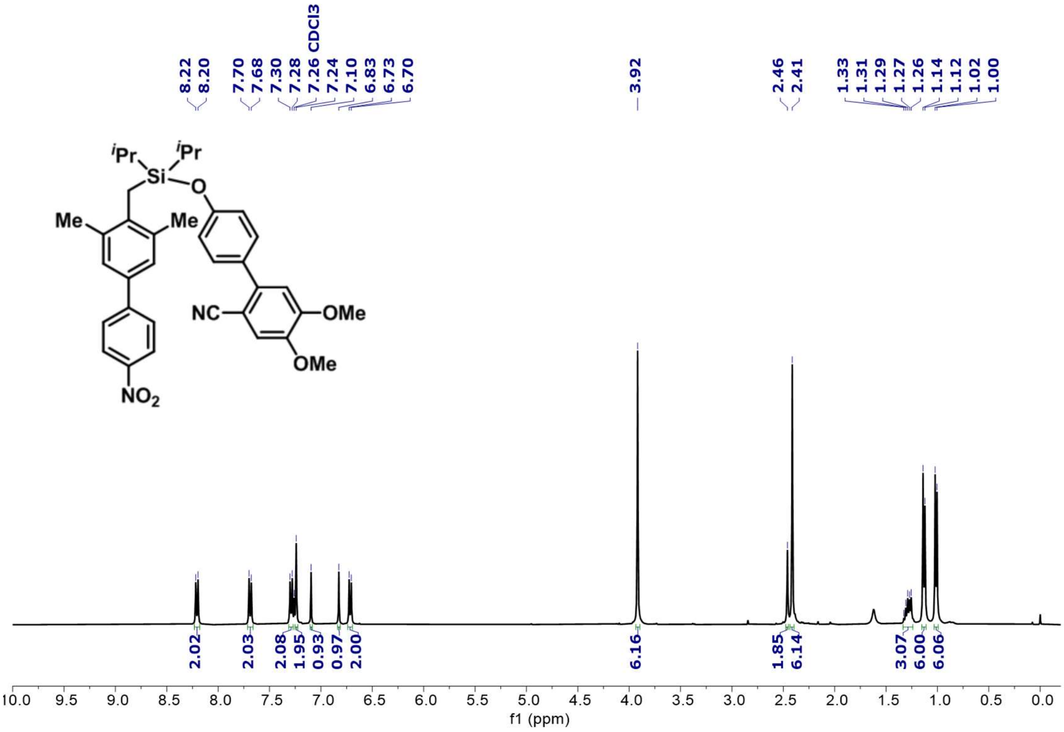

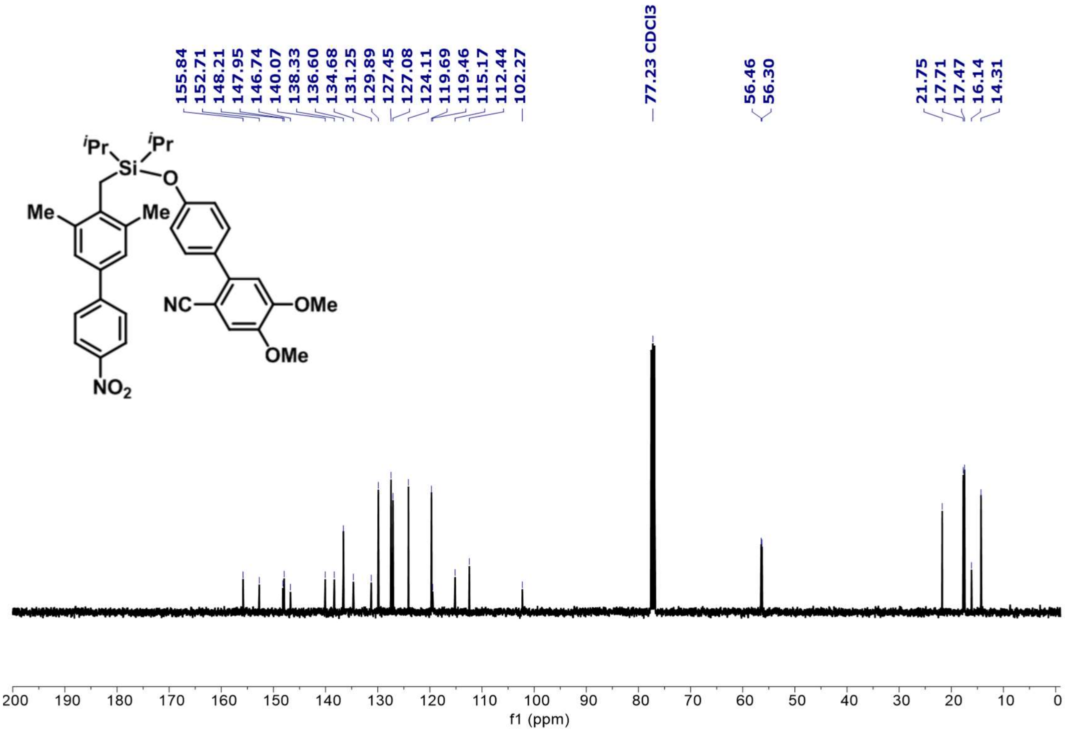

Supplementary Figure 59. ^{1}H (top) and ^{13}C (bottom) NMR of **3lm**

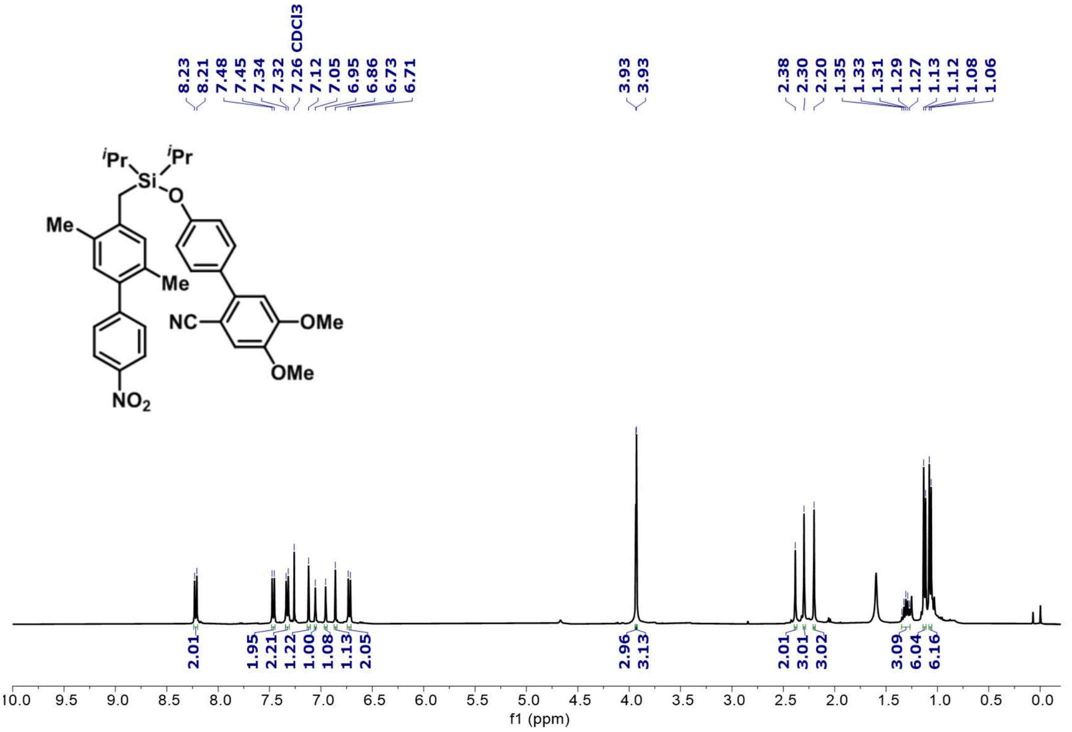

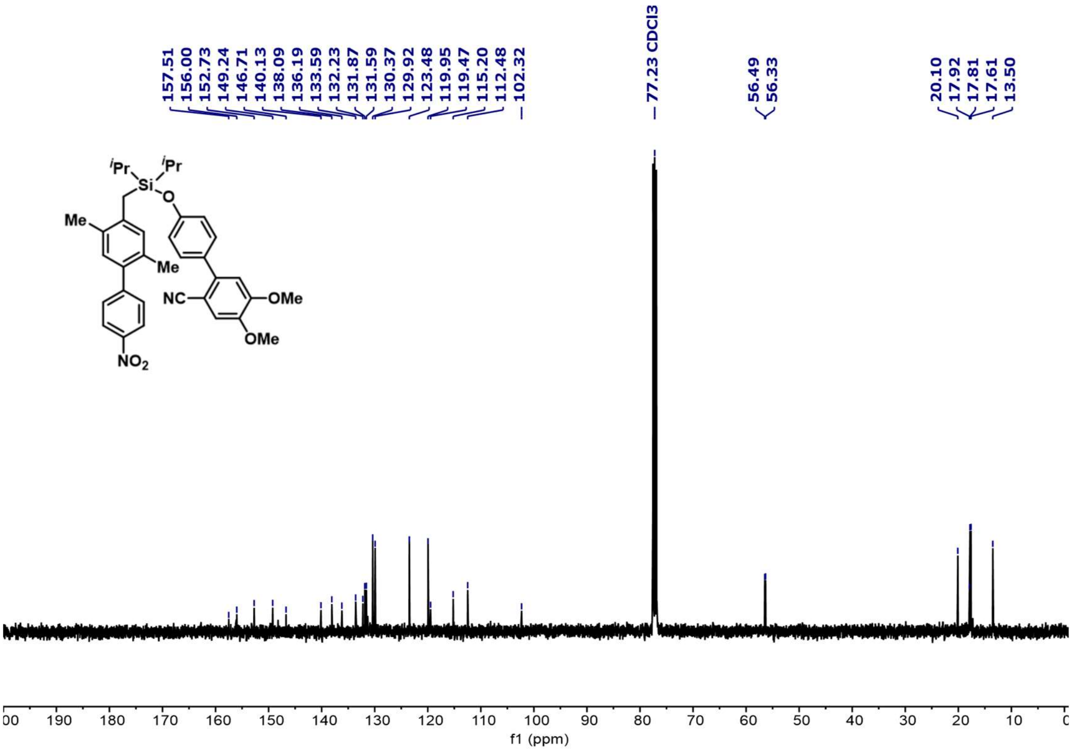

Supplementary Figure 60. ^{1}H (top) and ^{13}C (bottom) NMR of **3mm**

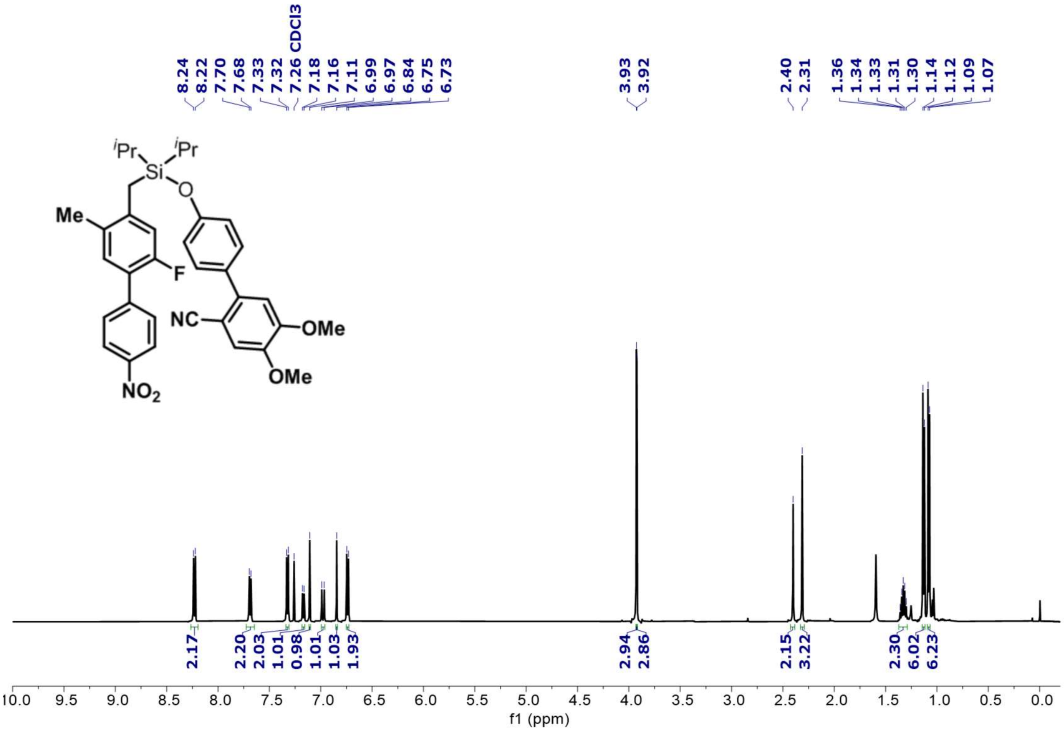

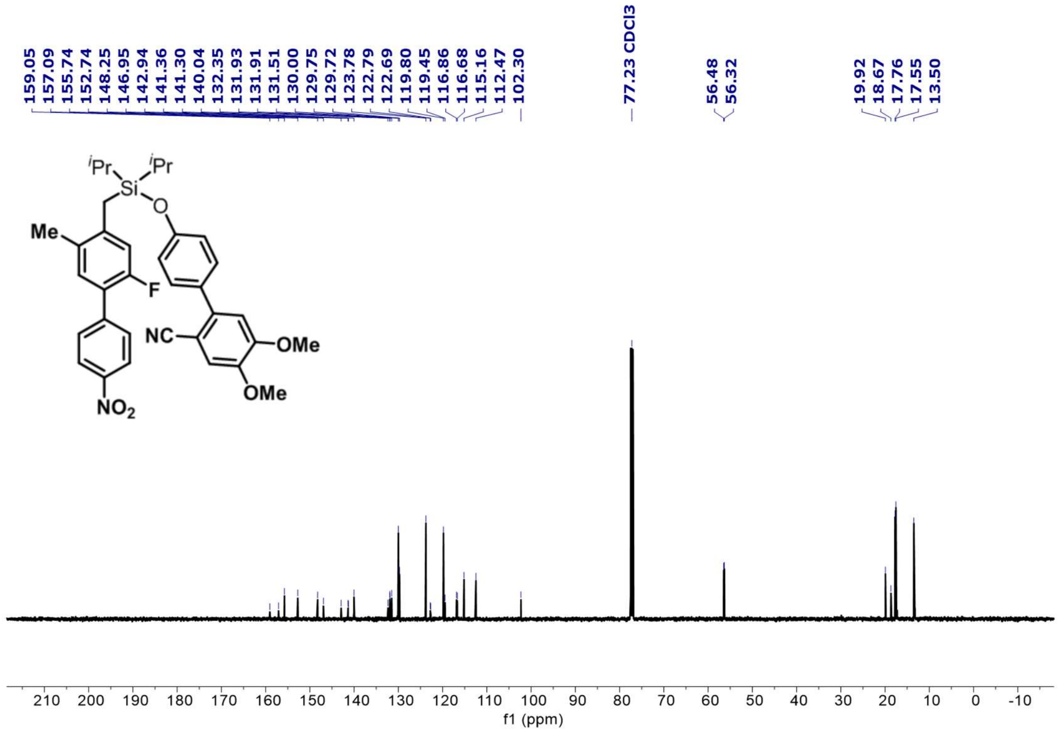

Supplementary Figure 61. ^{1}H (top) and ^{13}C (bottom) NMR of **3nm**

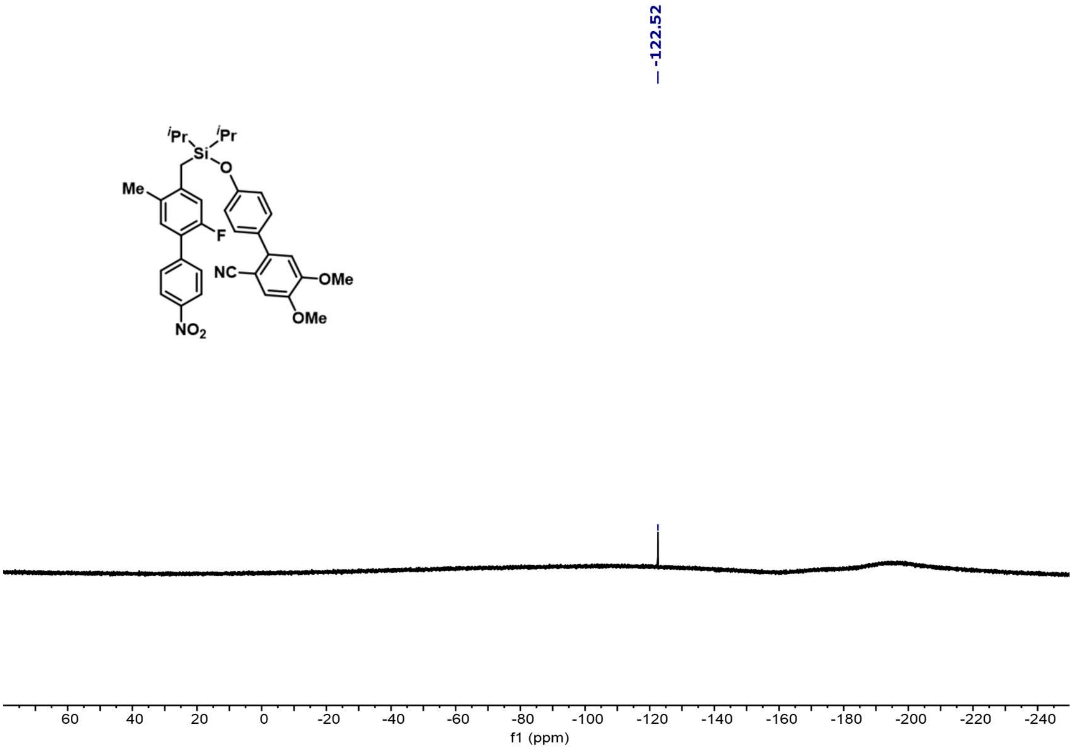

Supplementary Figure 62. ^{19}F NMR of **3nm**

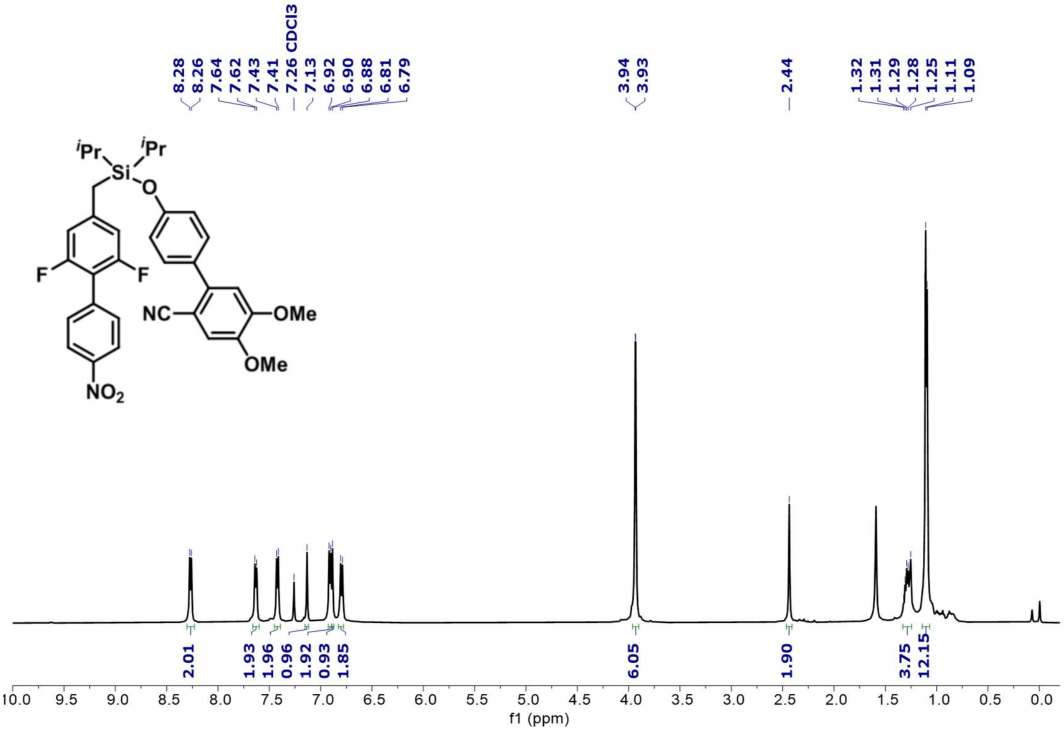

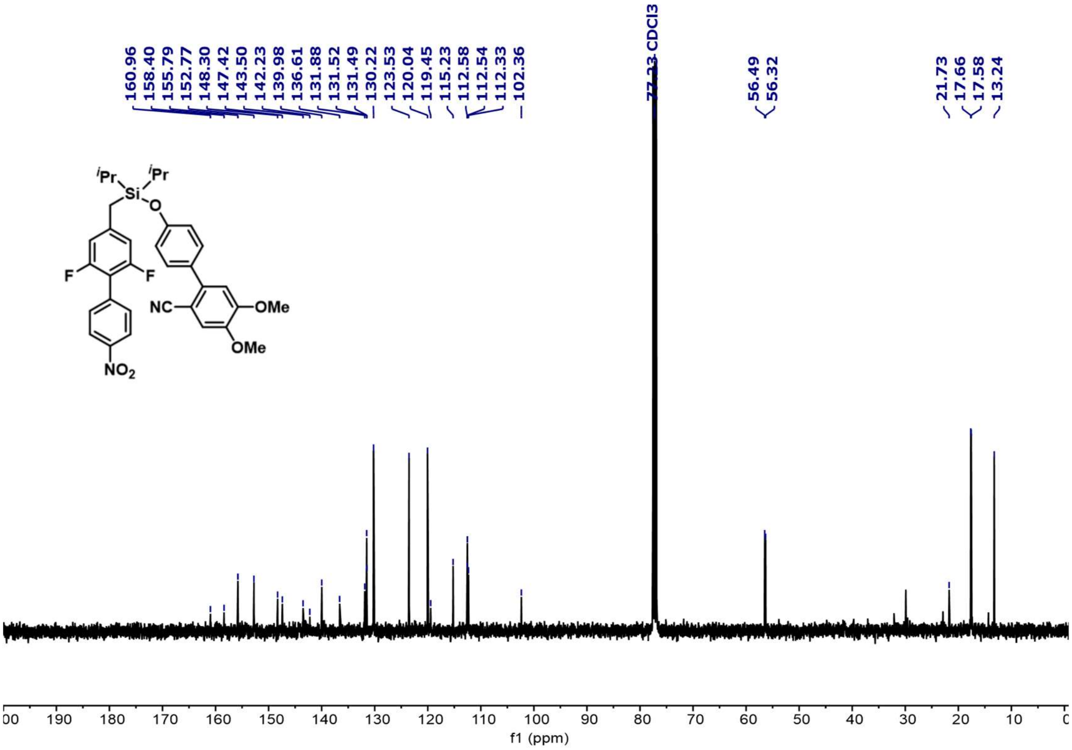

Supplementary Figure 63. ^{1}H (top) and ^{13}C (bottom) NMR of **3om**

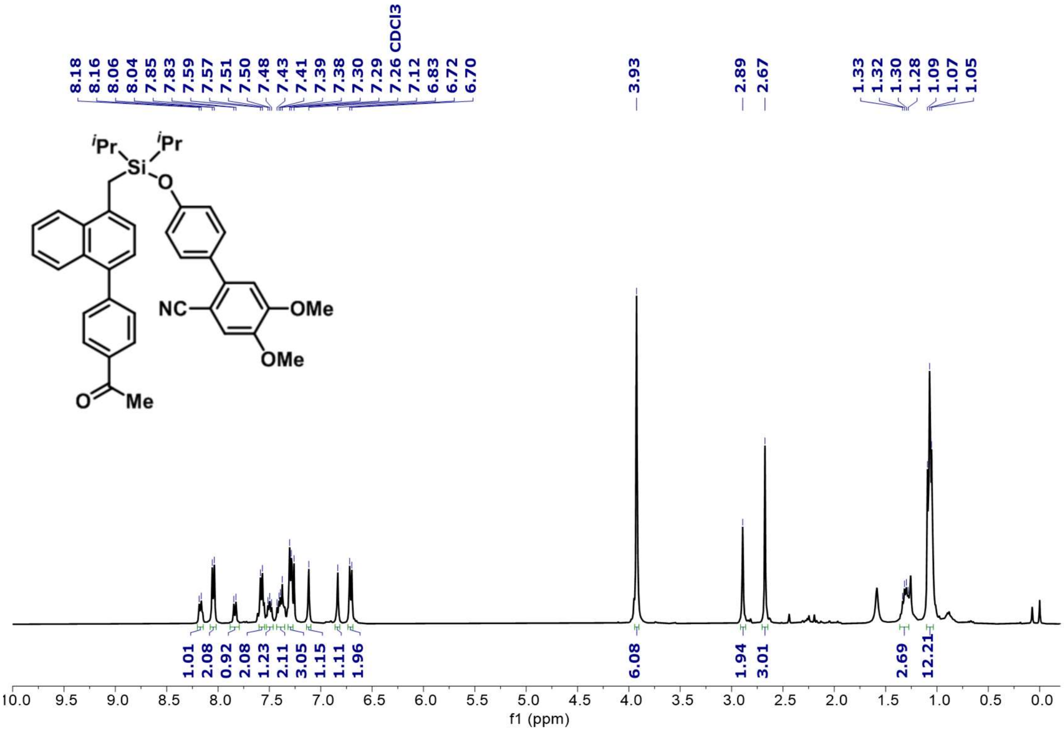

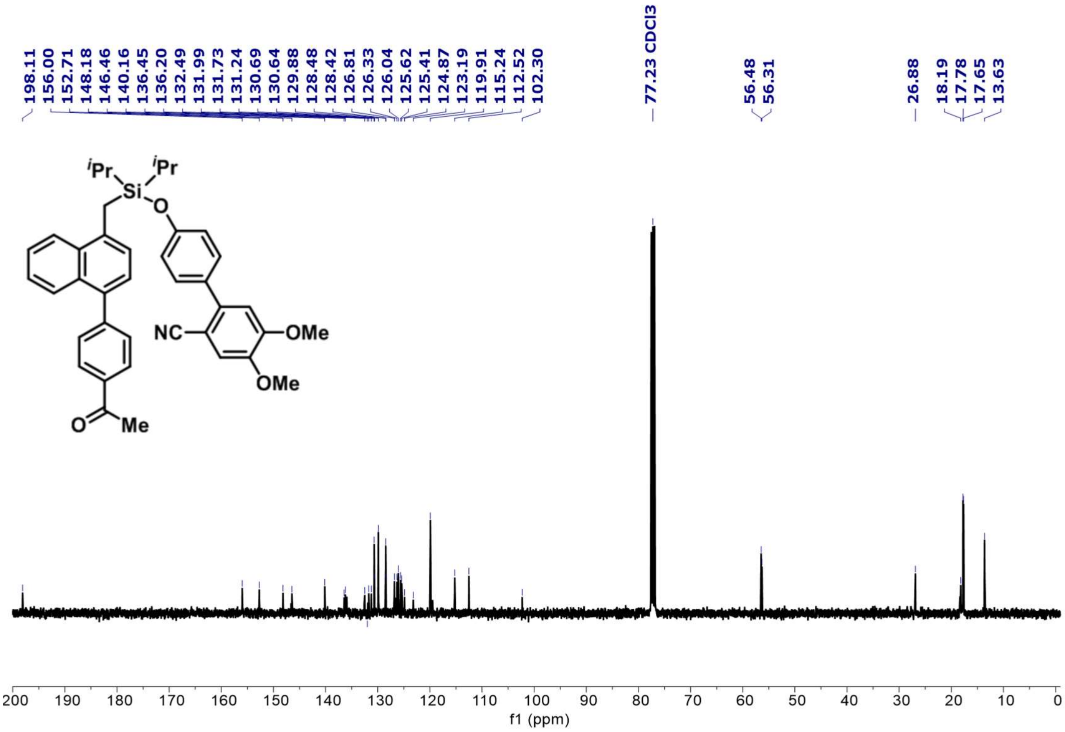

Supplementary Figure 64. ^{1}H (top) and ^{13}C (bottom) NMR of **3pl**

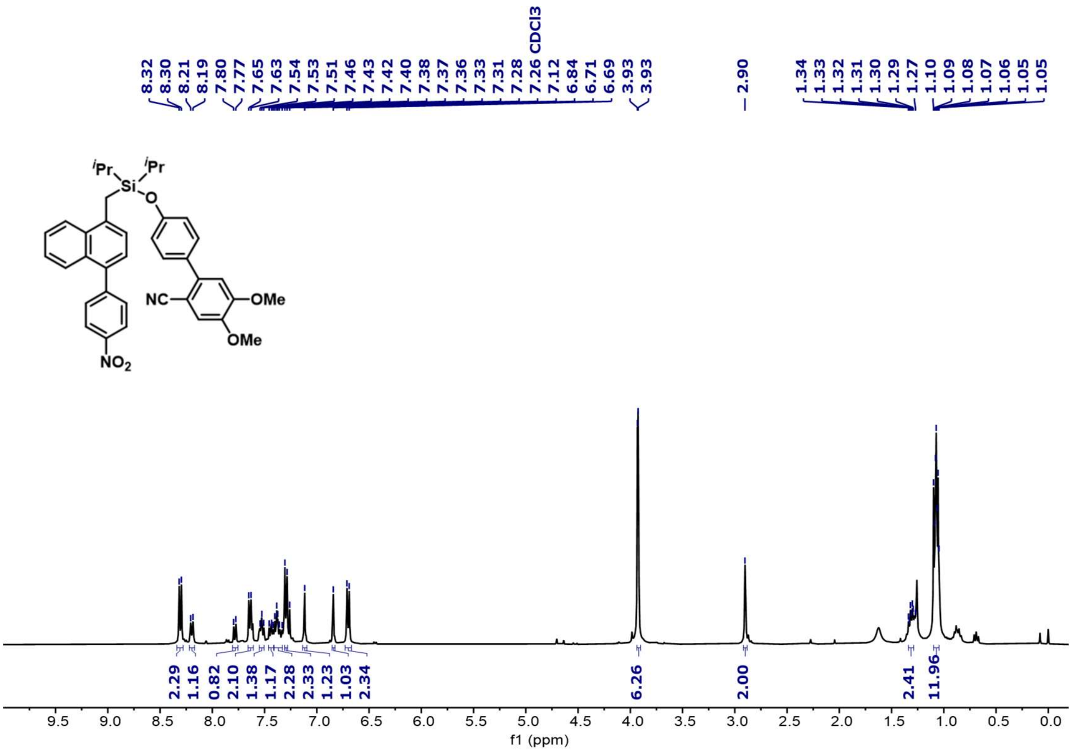

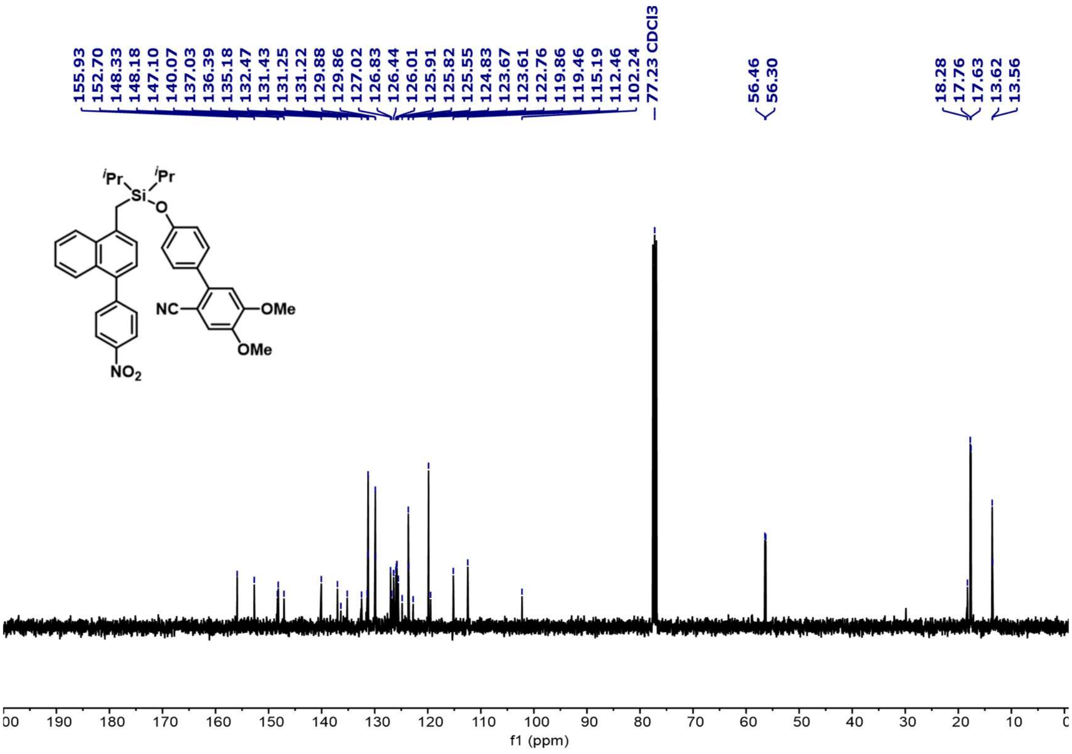

Supplementary Figure 65. ^{1}H (top) and ^{13}C (bottom) NMR of **3pm**

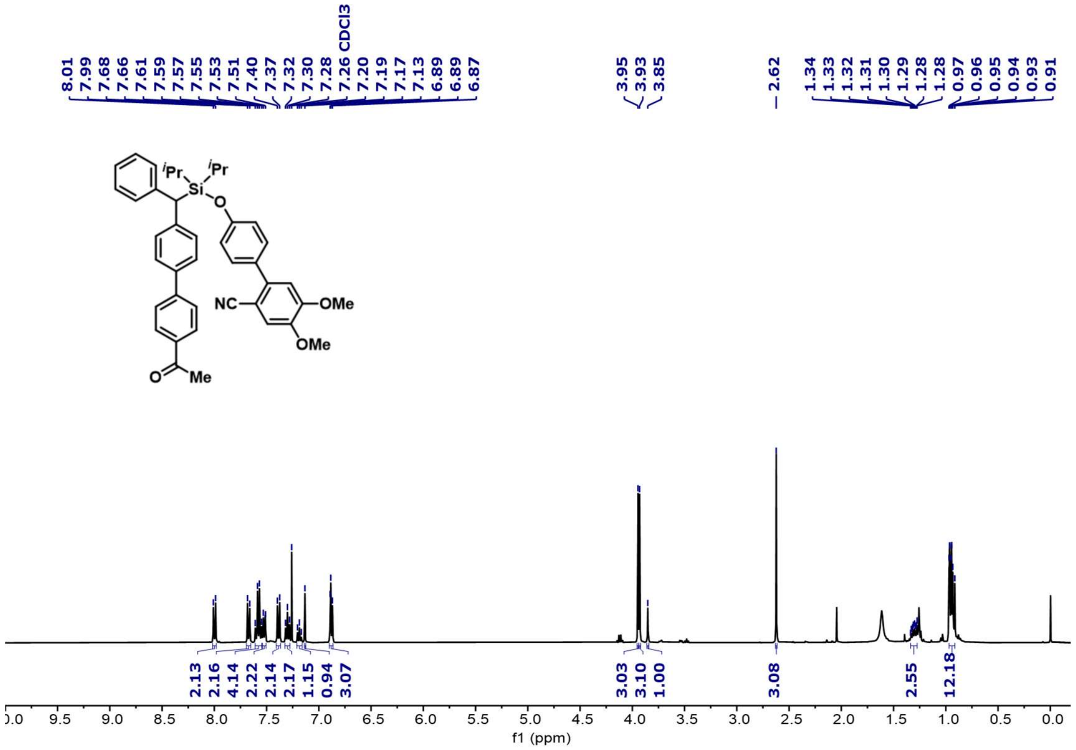

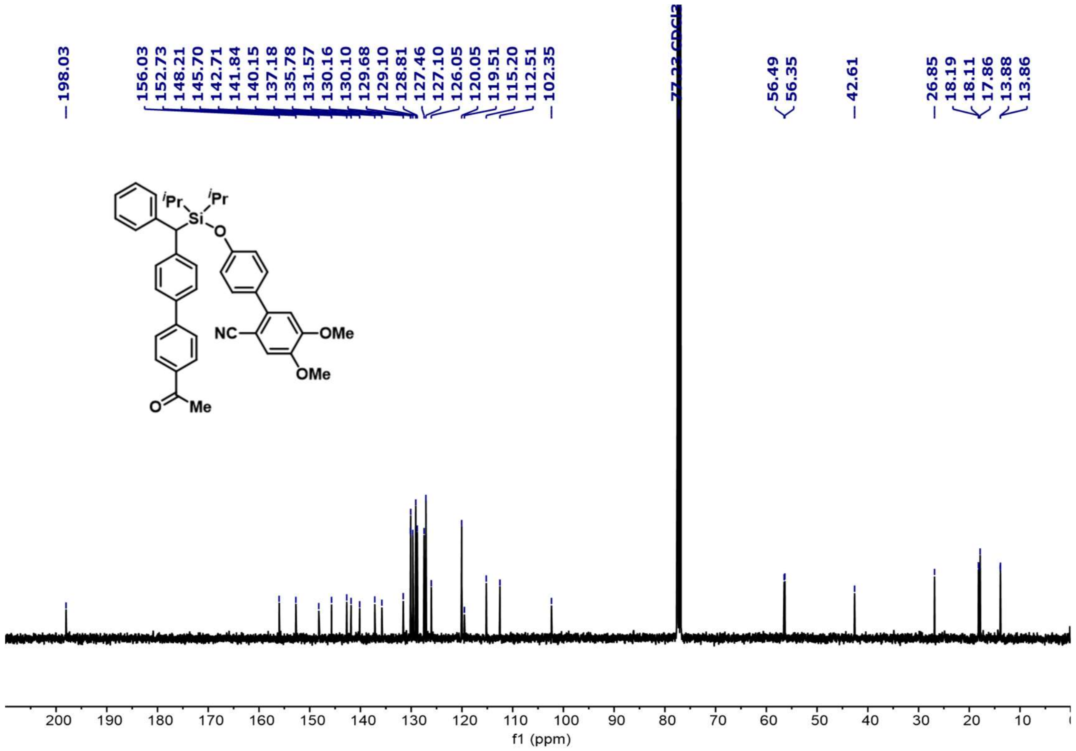

Supplementary Figure 66. ^{1}H (top) and ^{13}C (bottom) NMR of **3ql**

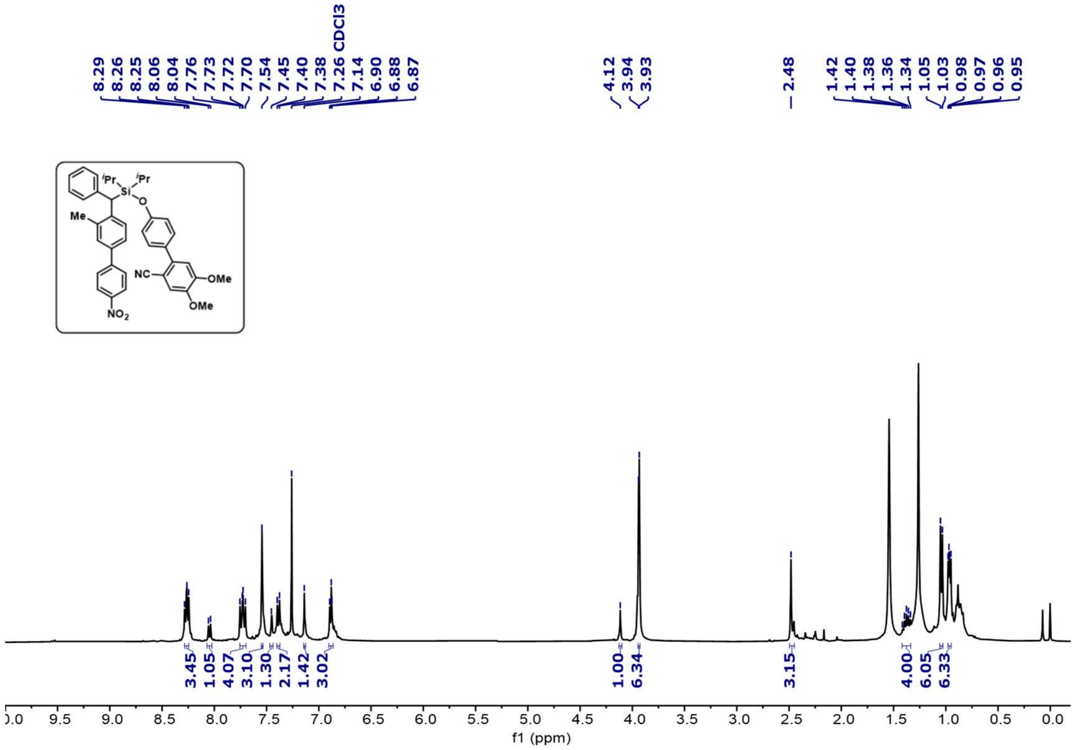

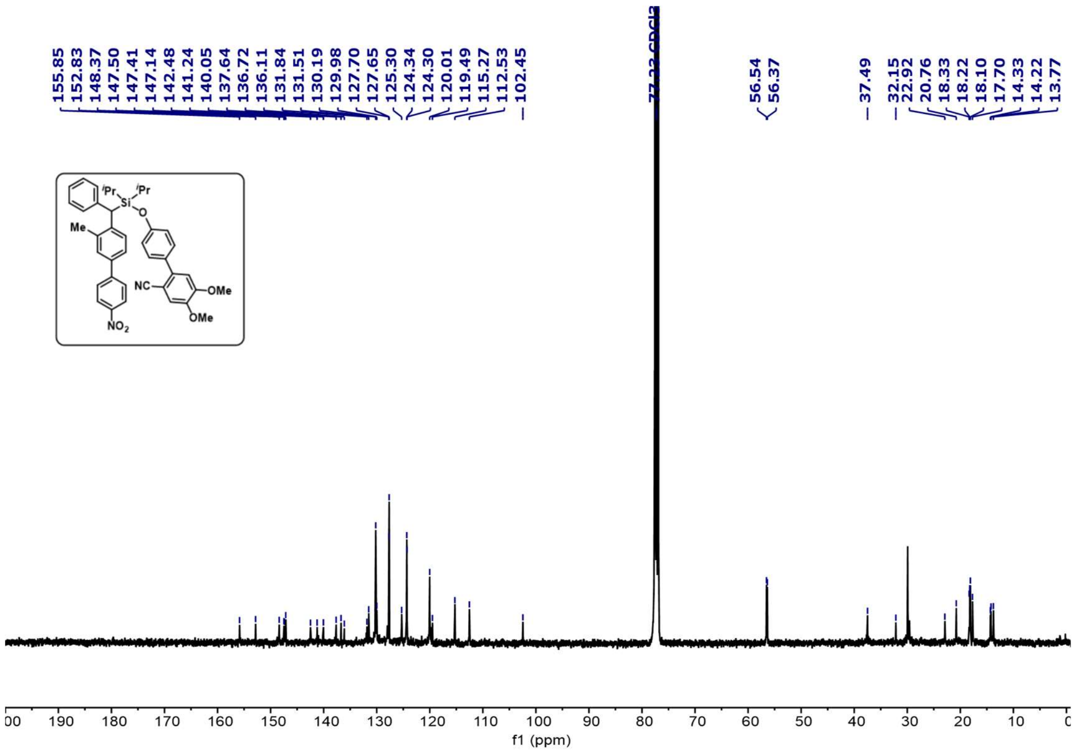

Supplementary Figure 67. ^{1}H (top) and ^{13}C (bottom) NMR of **3rm**

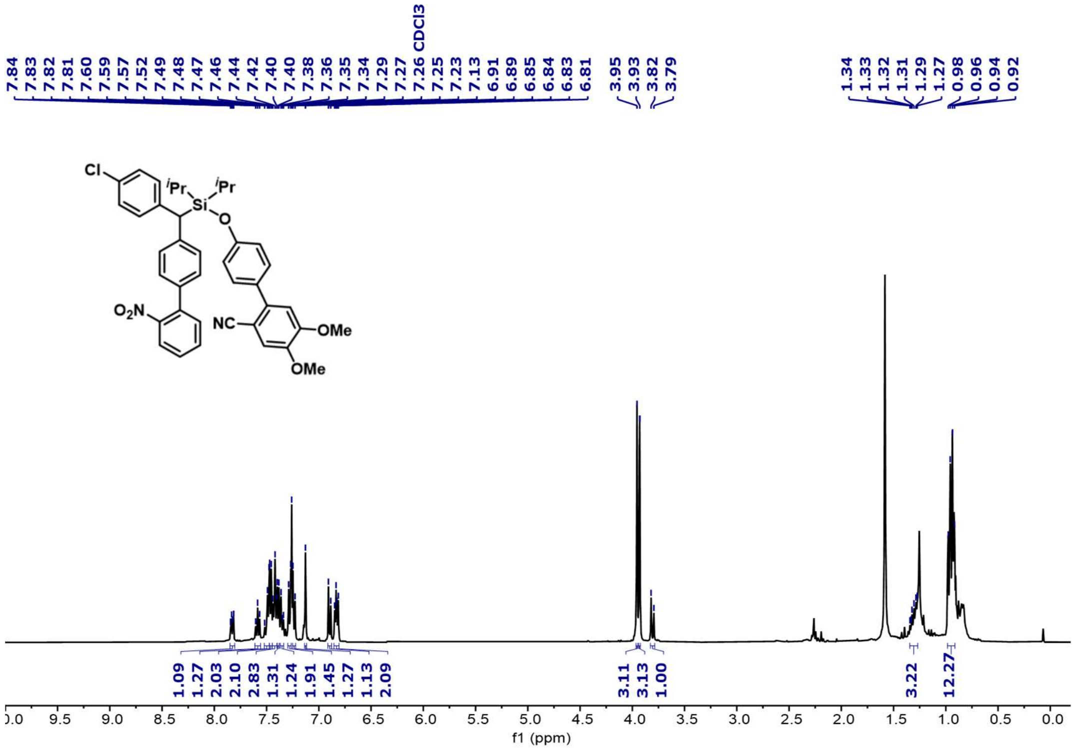

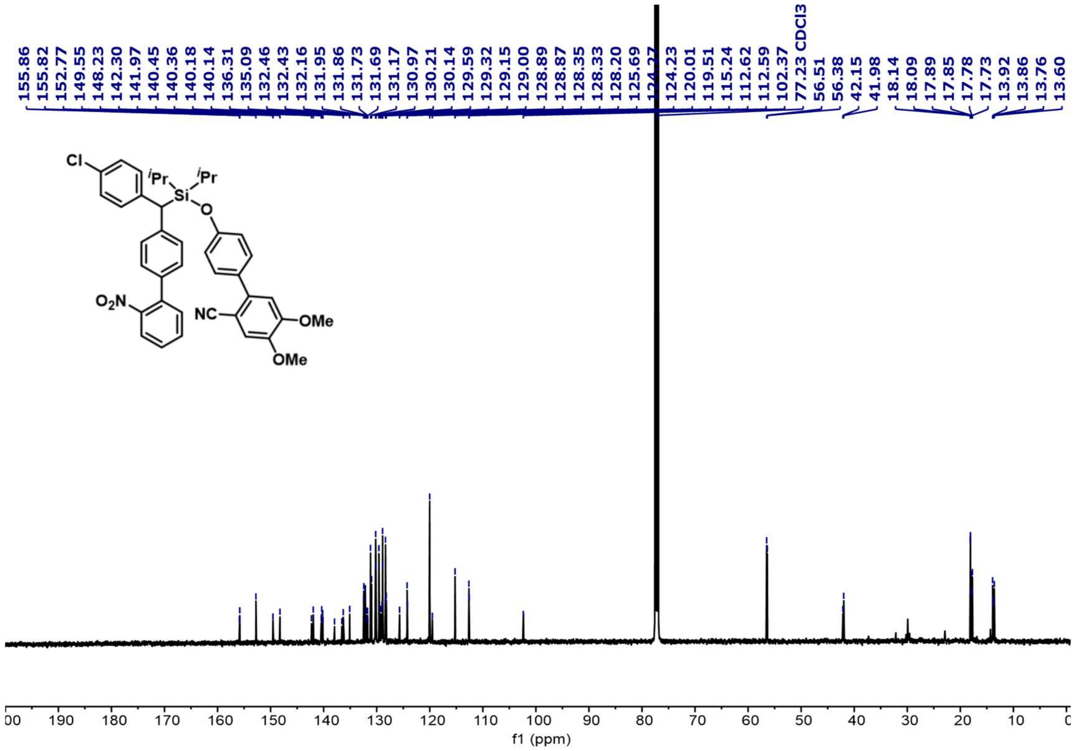

Supplementary Figure 68. ^{1}H (top) and ^{13}C (bottom) NMR of **3sz**

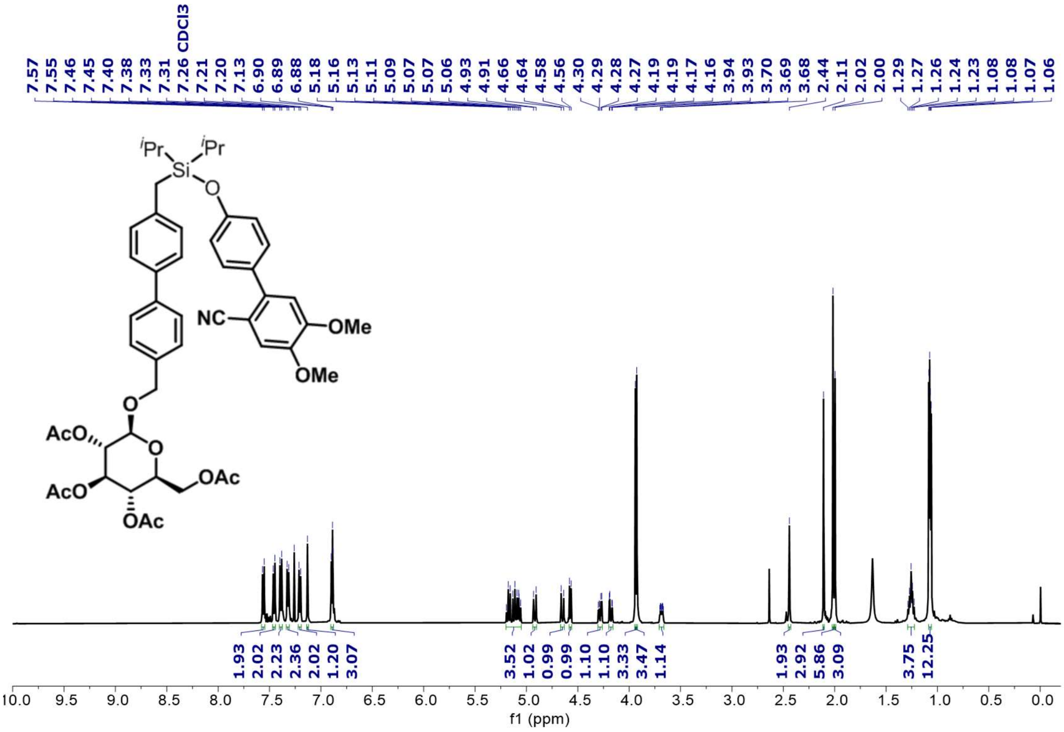

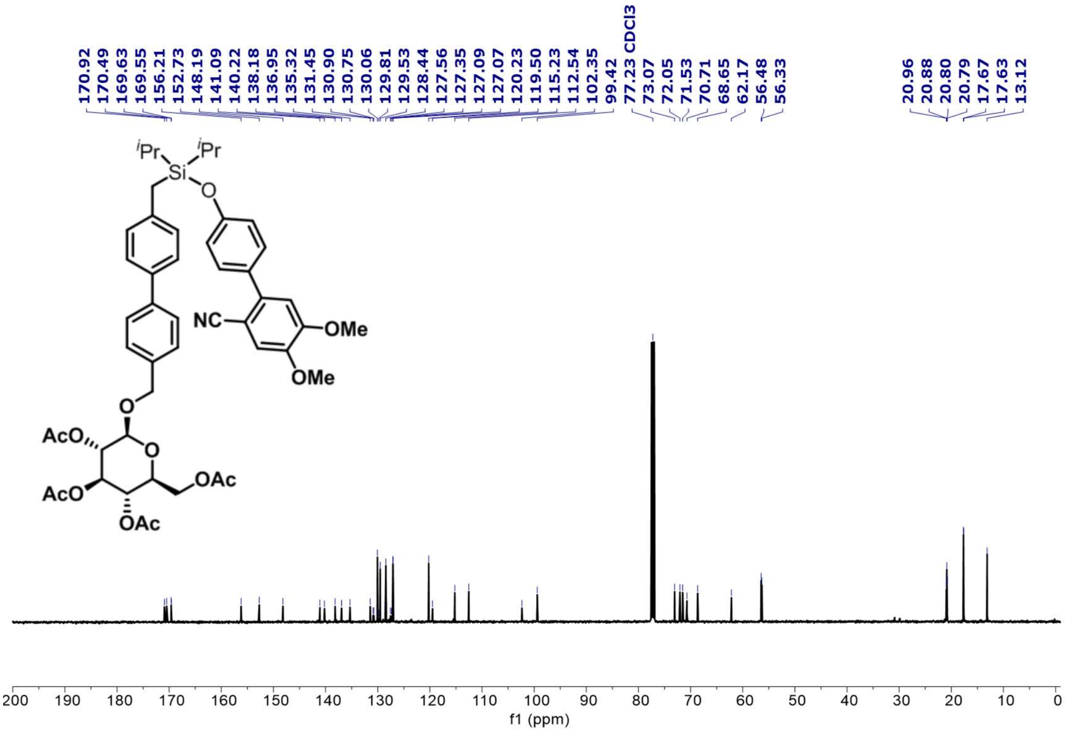

Supplementary Figure 69. ^{1}H (top) and ^{13}C (bottom) NMR of **5aa**

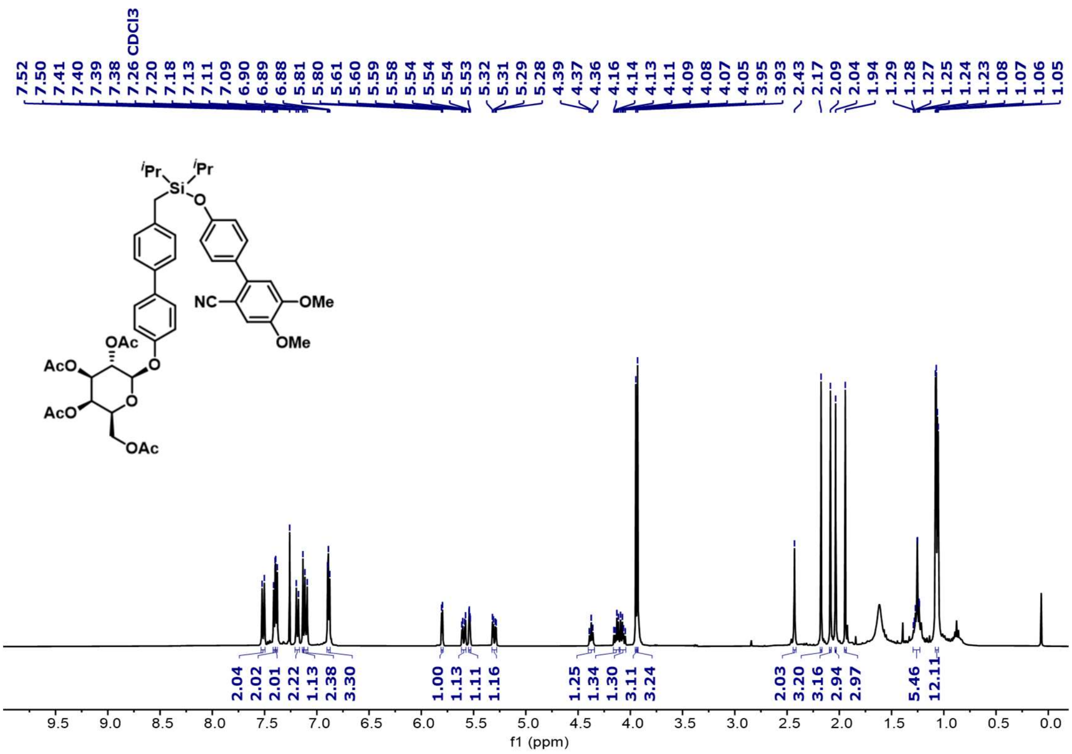

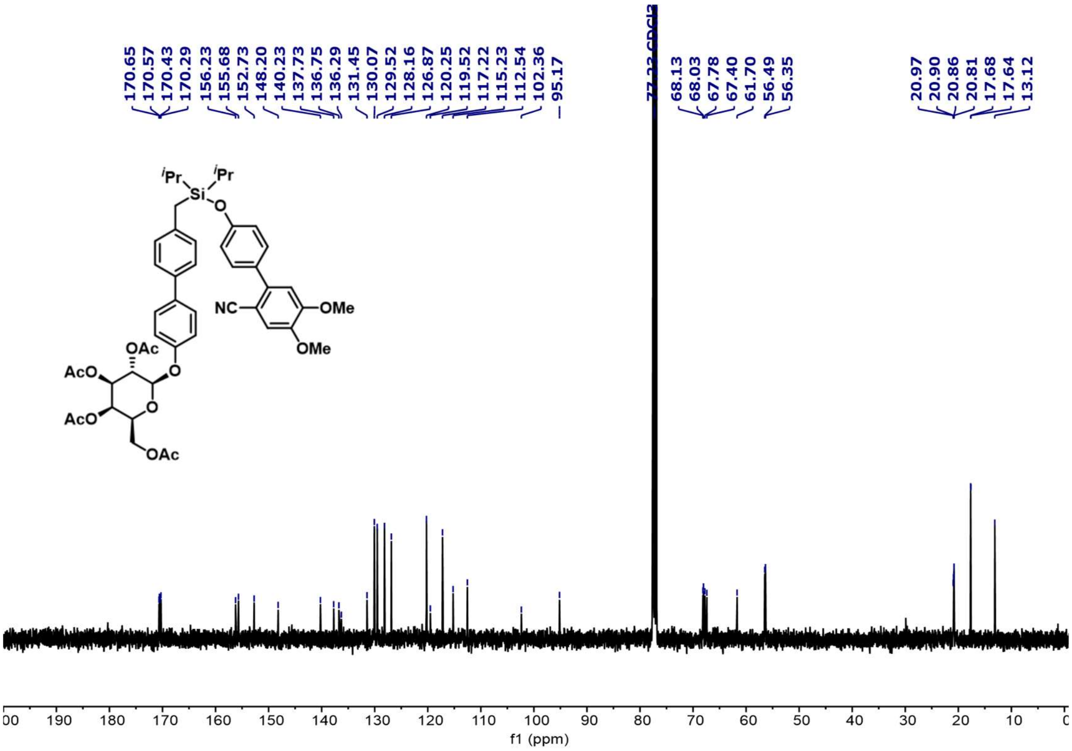

Supplementary Figure 70. ^{1}H (top) and ^{13}C (bottom) NMR of **5ab**

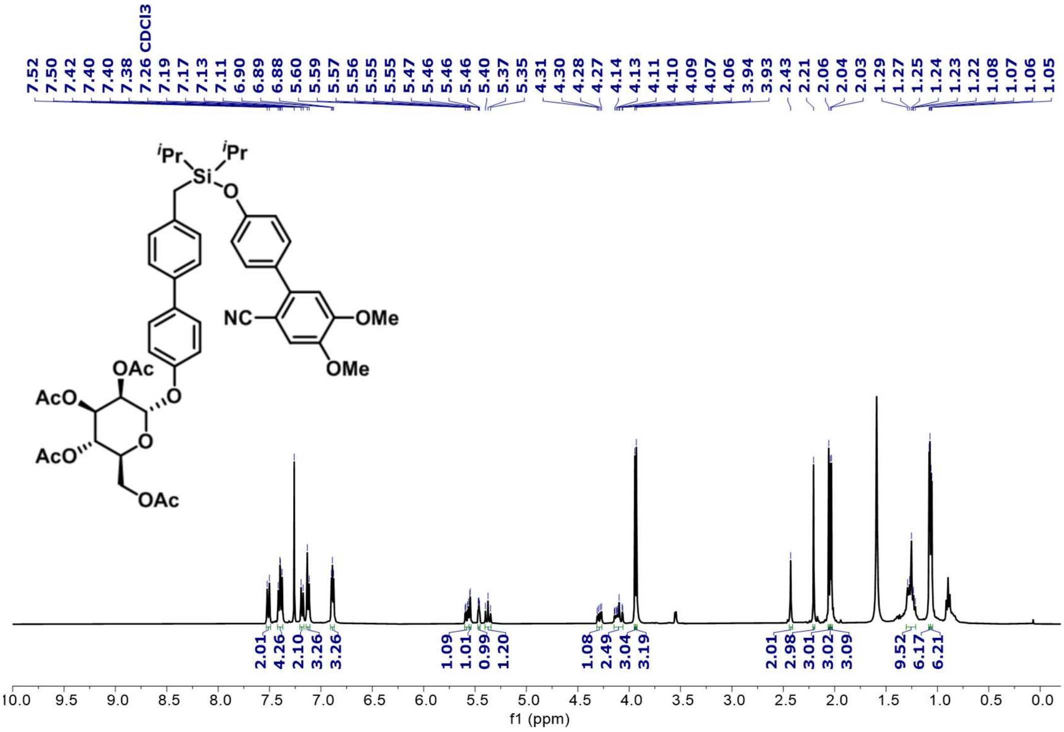

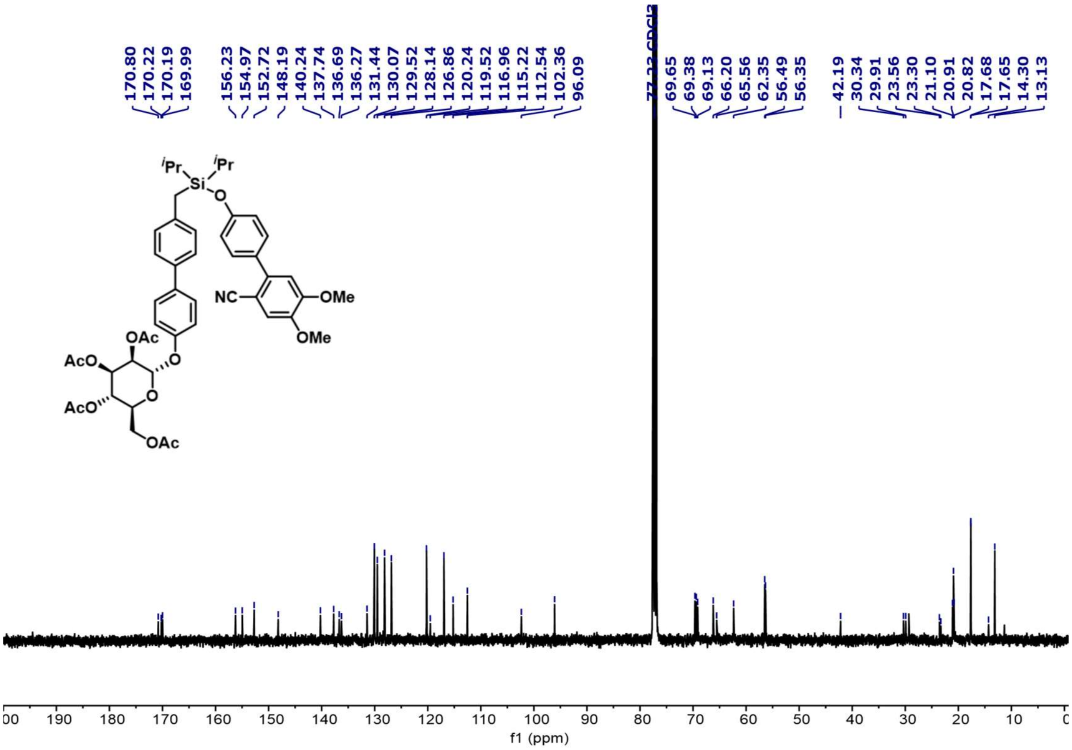

Supplementary Figure 71. ^{1}H (top) and ^{13}C (bottom) NMR of **5ac**

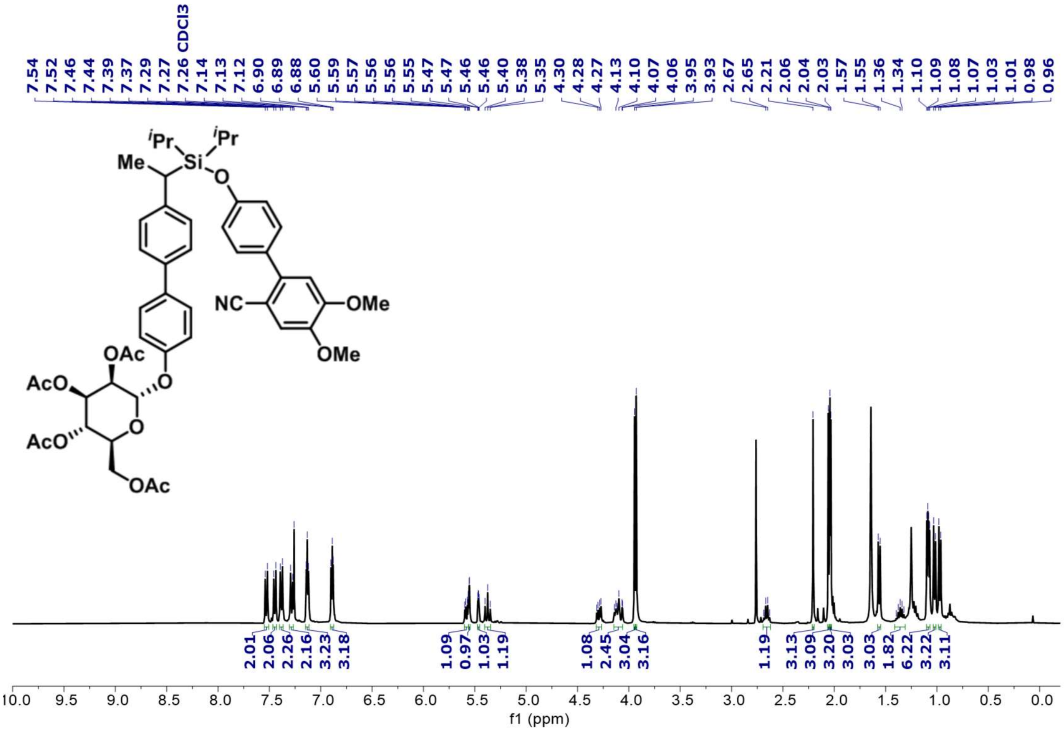

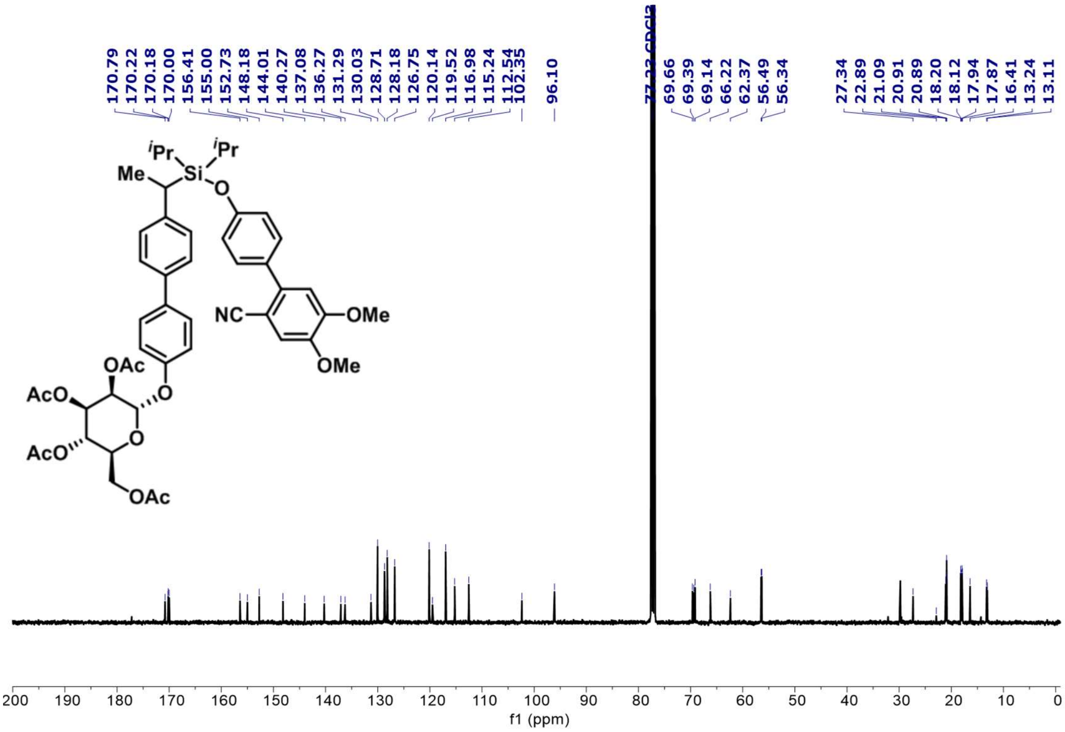

Supplementary Figure 72. ^{1}H (top) and ^{13}C (bottom) NMR of **5tc**

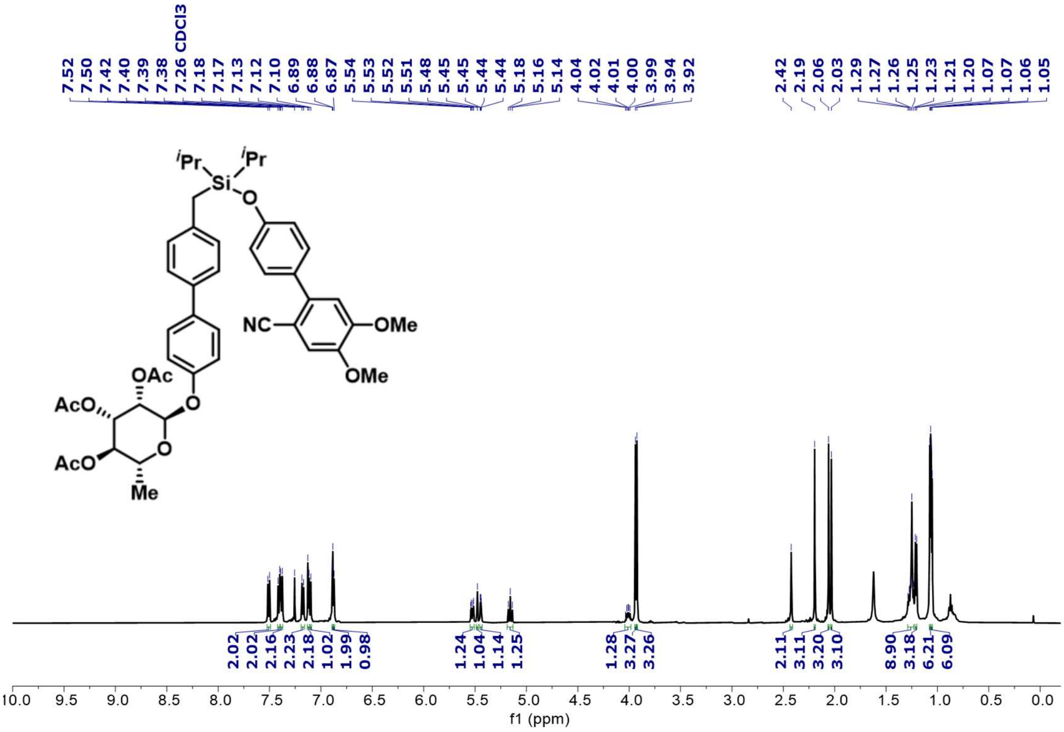

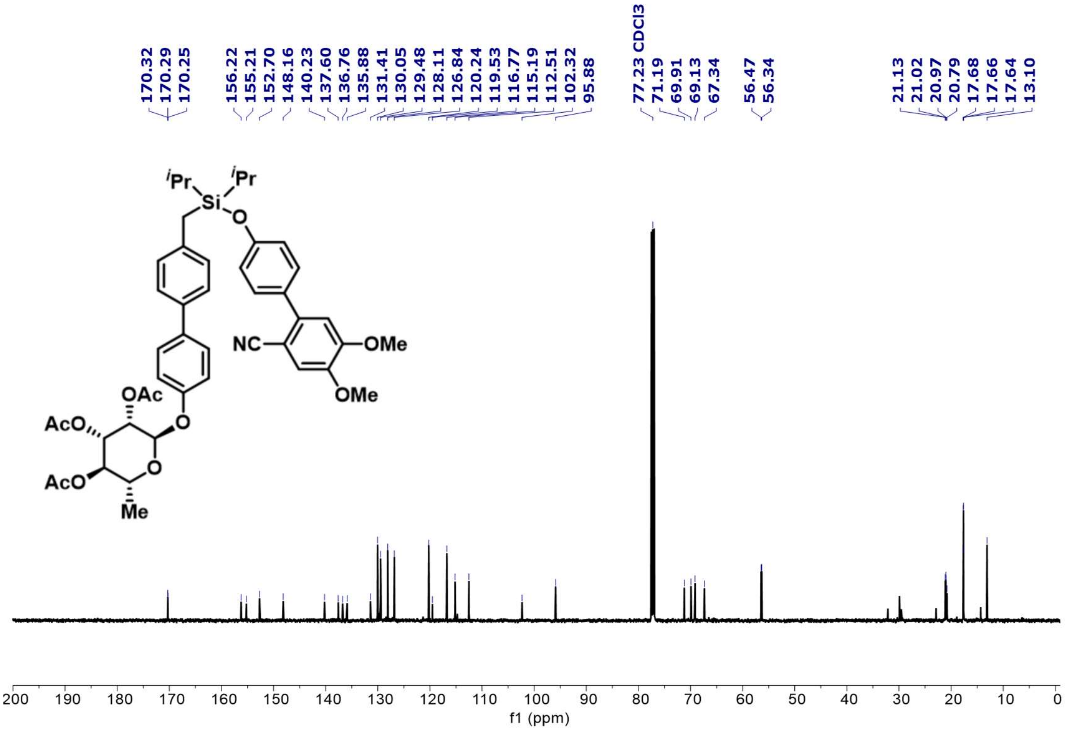

Supplementary Figure 73. ^{1}H (top) and ^{13}C (bottom) NMR of **5ad**

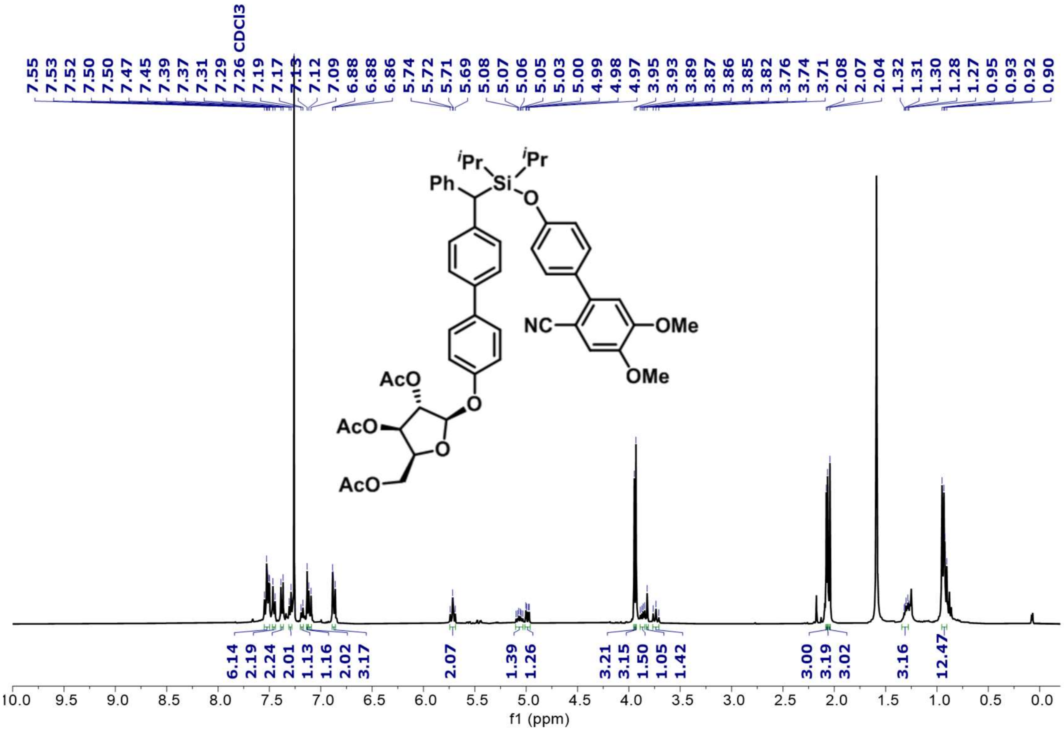

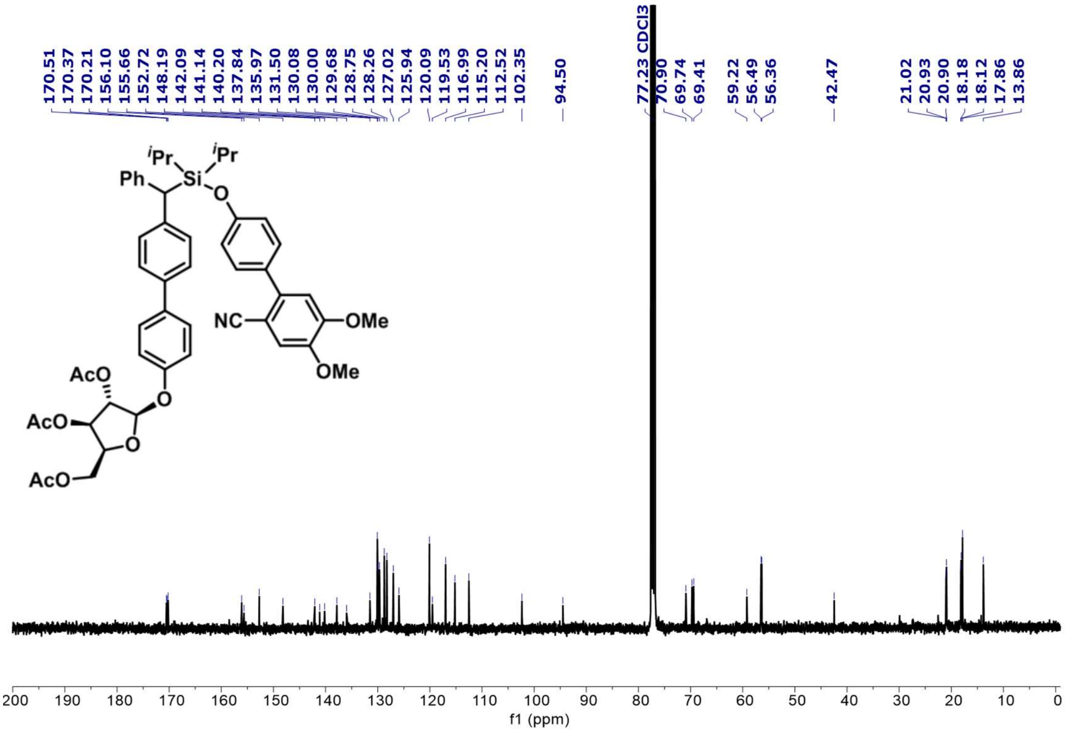

Supplementary Figure 74. ^{1}H (top) and ^{13}C (bottom) NMR of **5qe**

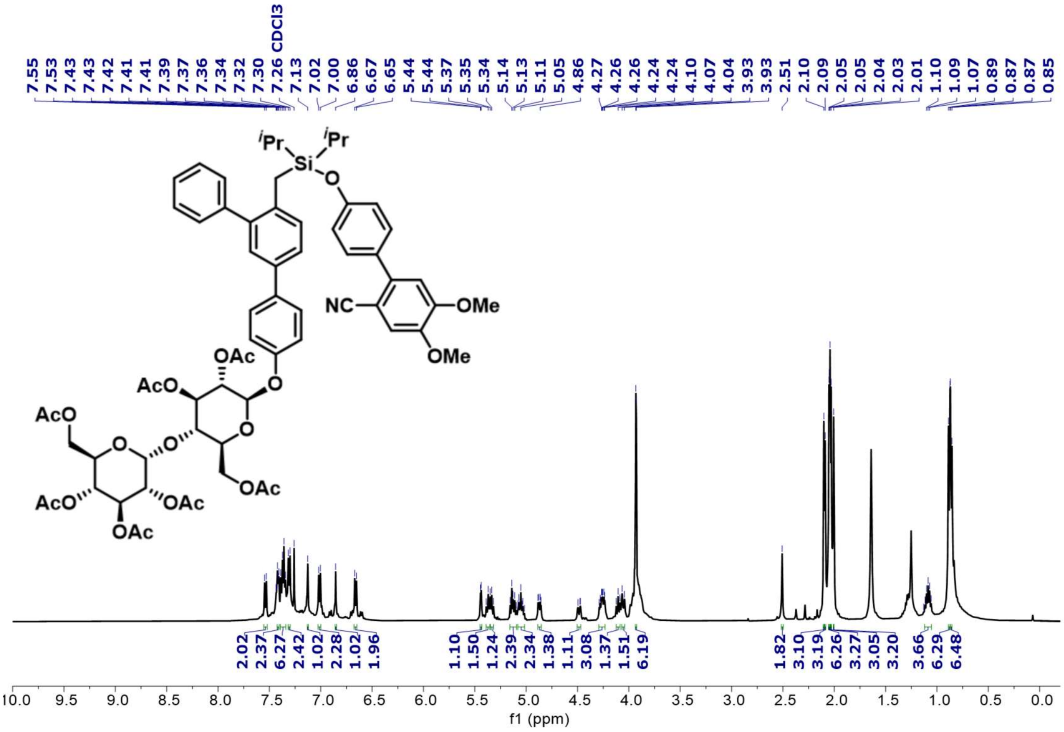

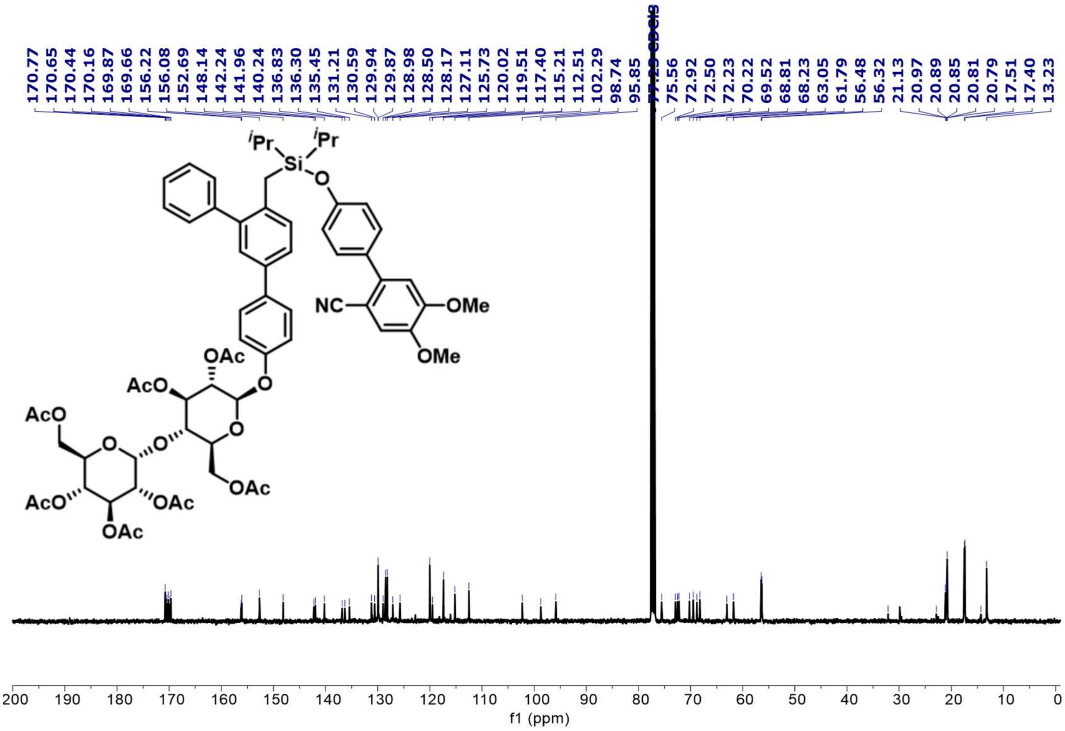

Supplementary Figure 75. ^{1}H (top) and ^{13}C (bottom) NMR of **5kf**

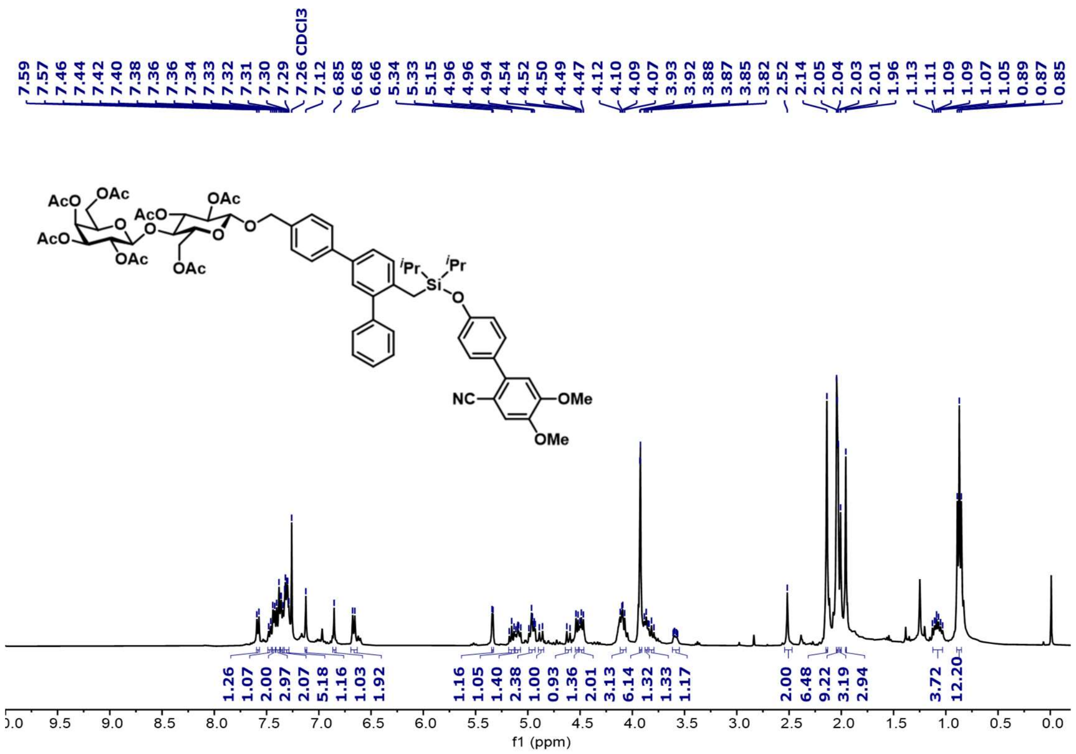

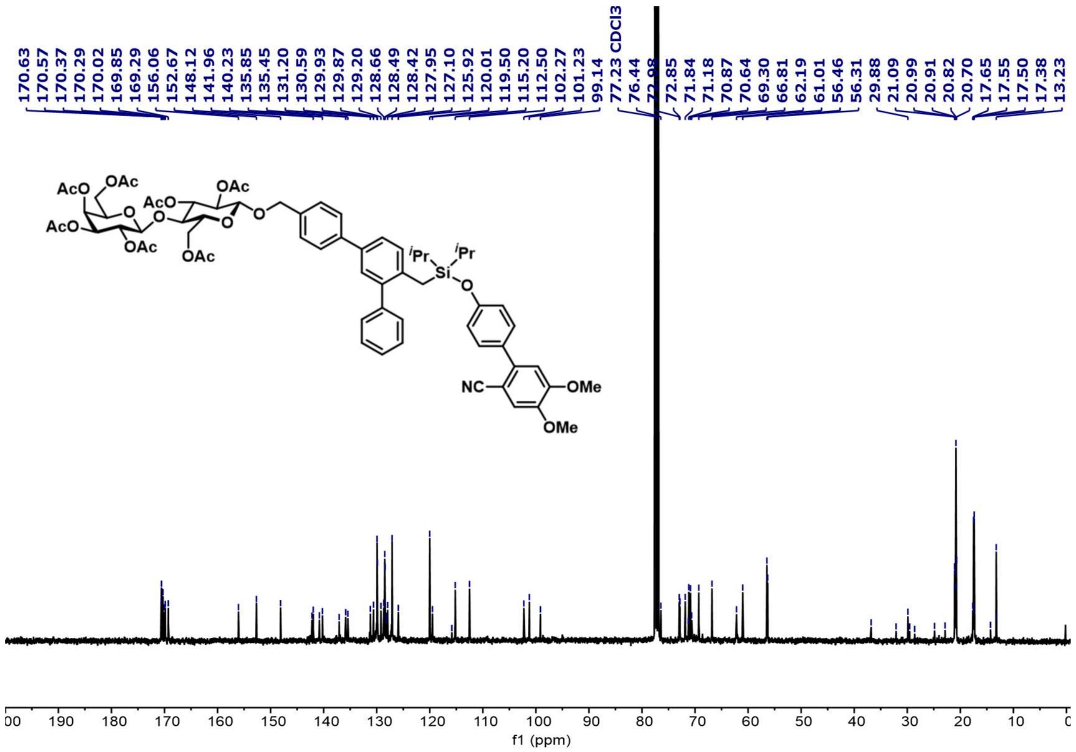

Supplementary Figure 76. ^{1}H (top) and ^{13}C (bottom) NMR of **5kg**

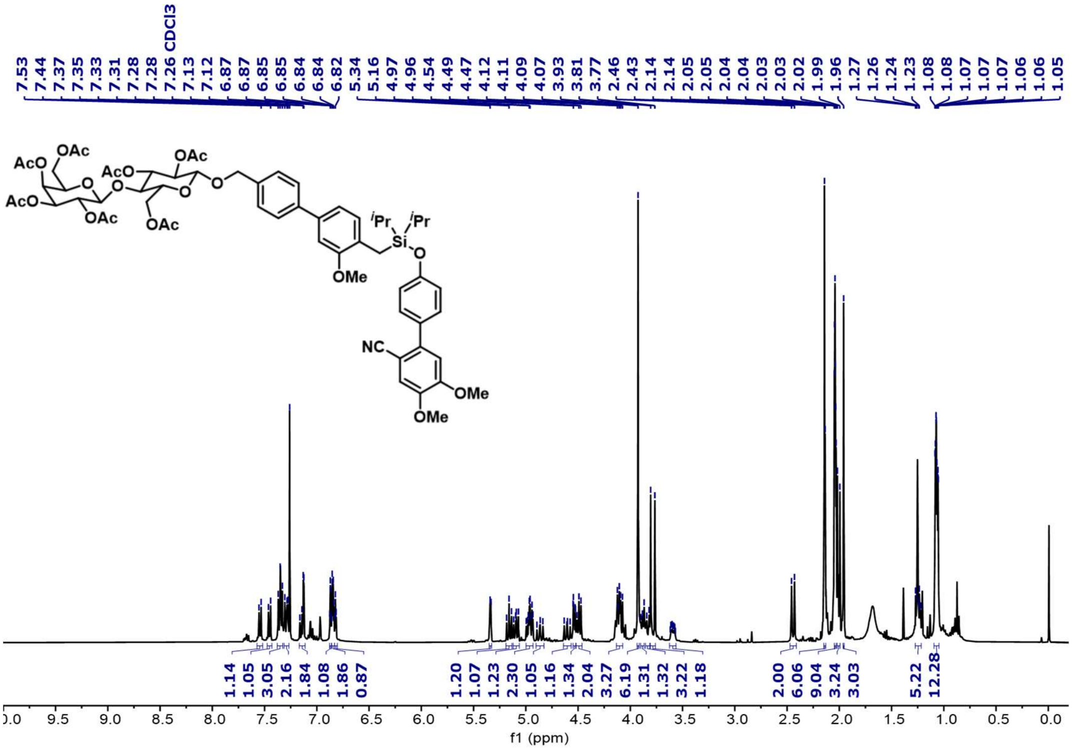

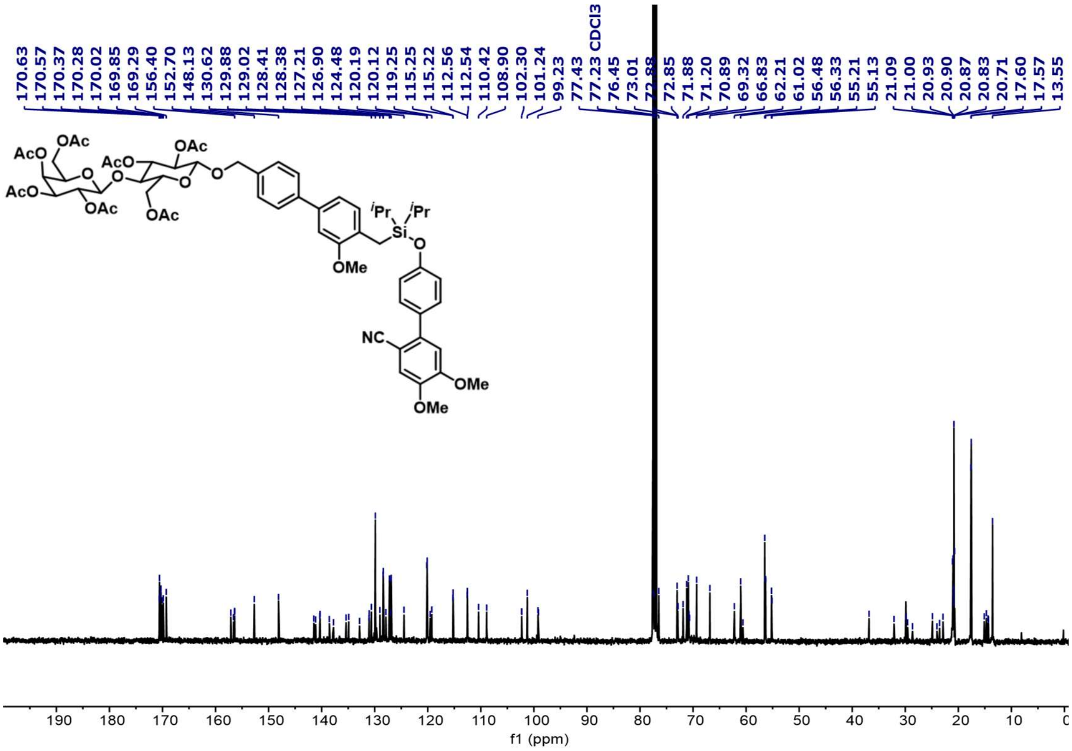

Supplementary Figure 77. ^{1}H (top) and ^{13}C (bottom) NMR of **5cg**

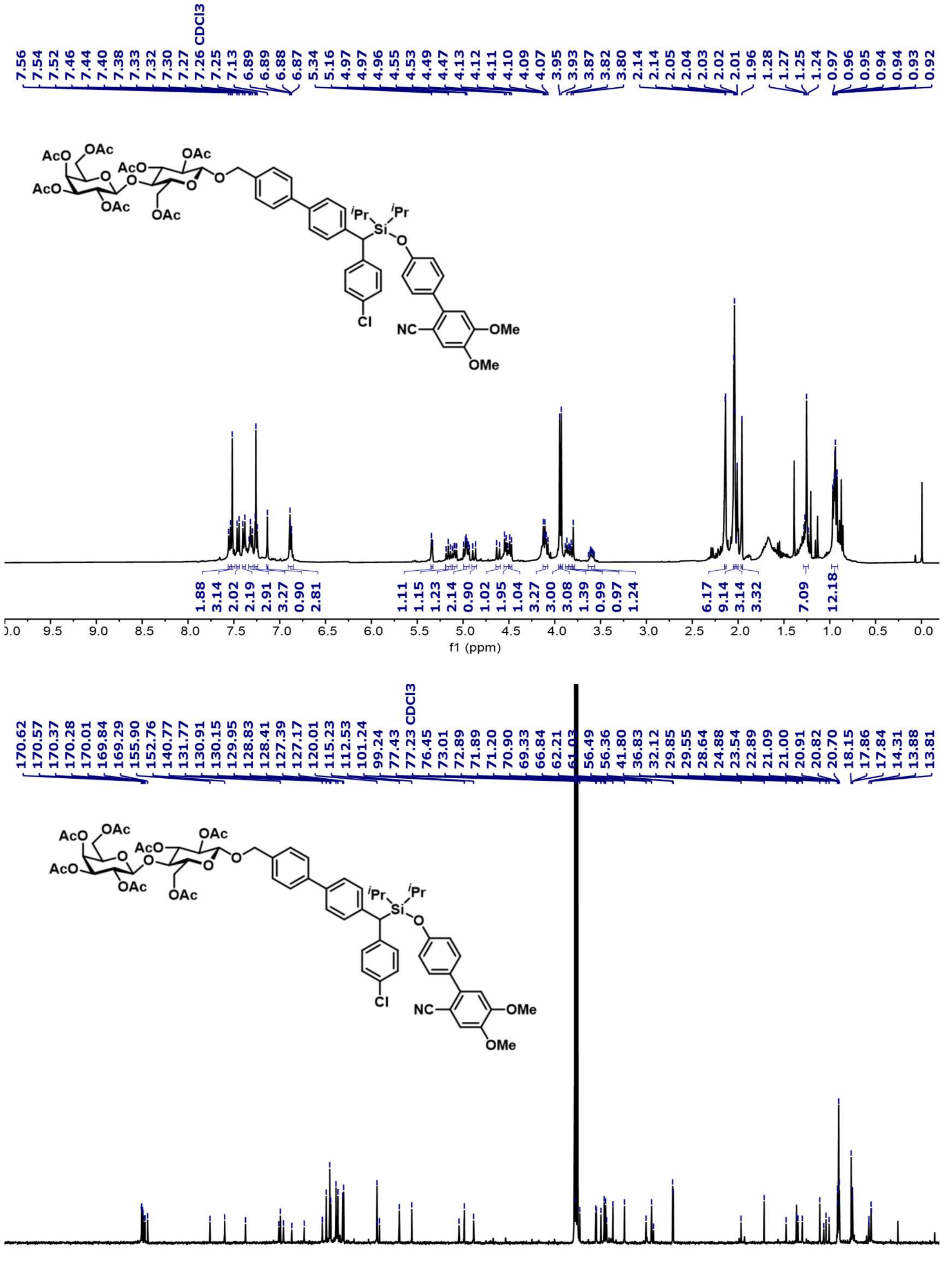

Supplementary Figure 78. ^{1}H (top) and ^{13}C (bottom) NMR of **5sg**

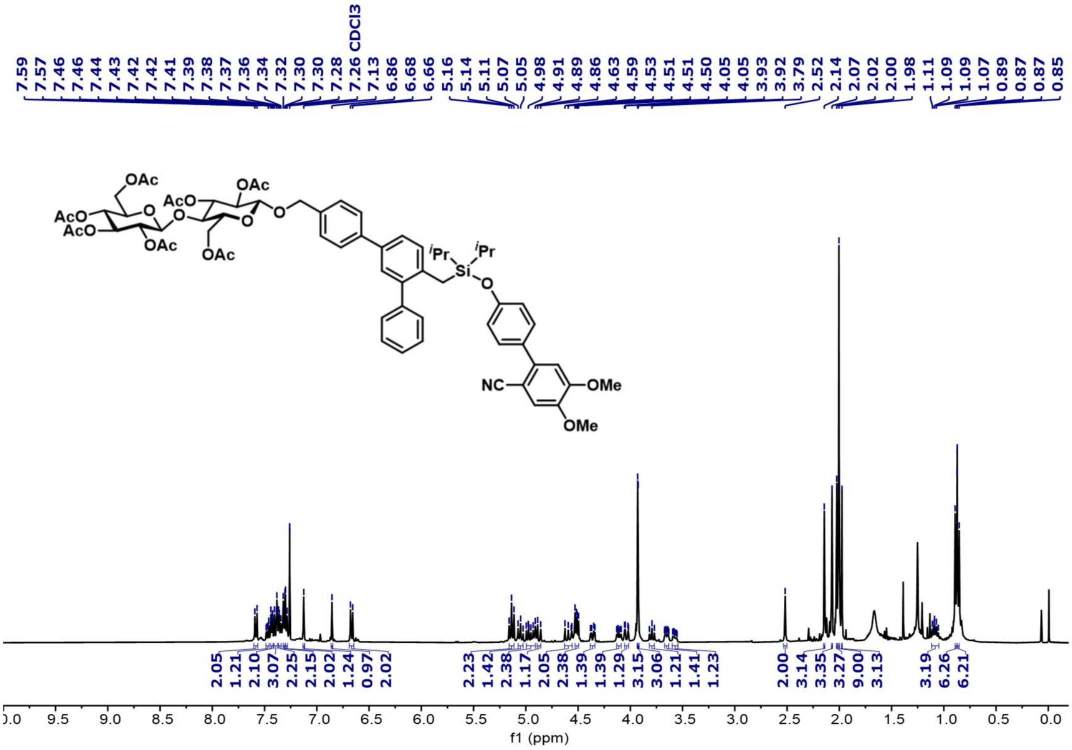

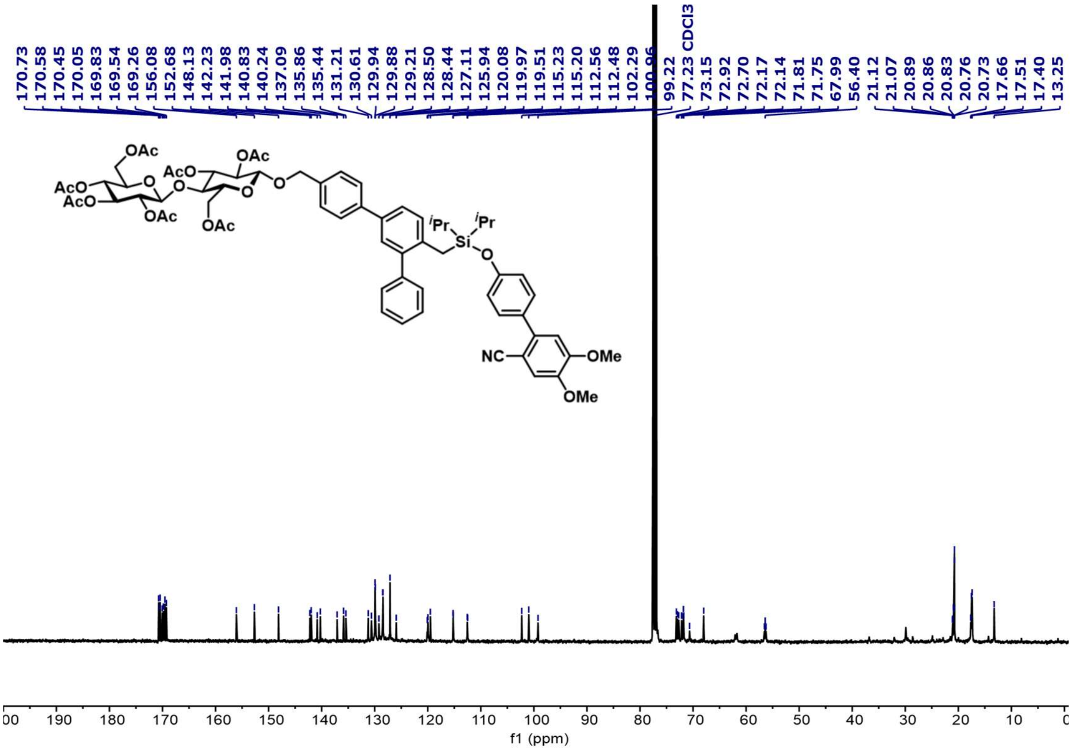

Supplementary Figure 79. ^{1}H (top) and ^{13}C (bottom) NMR of **5kh**

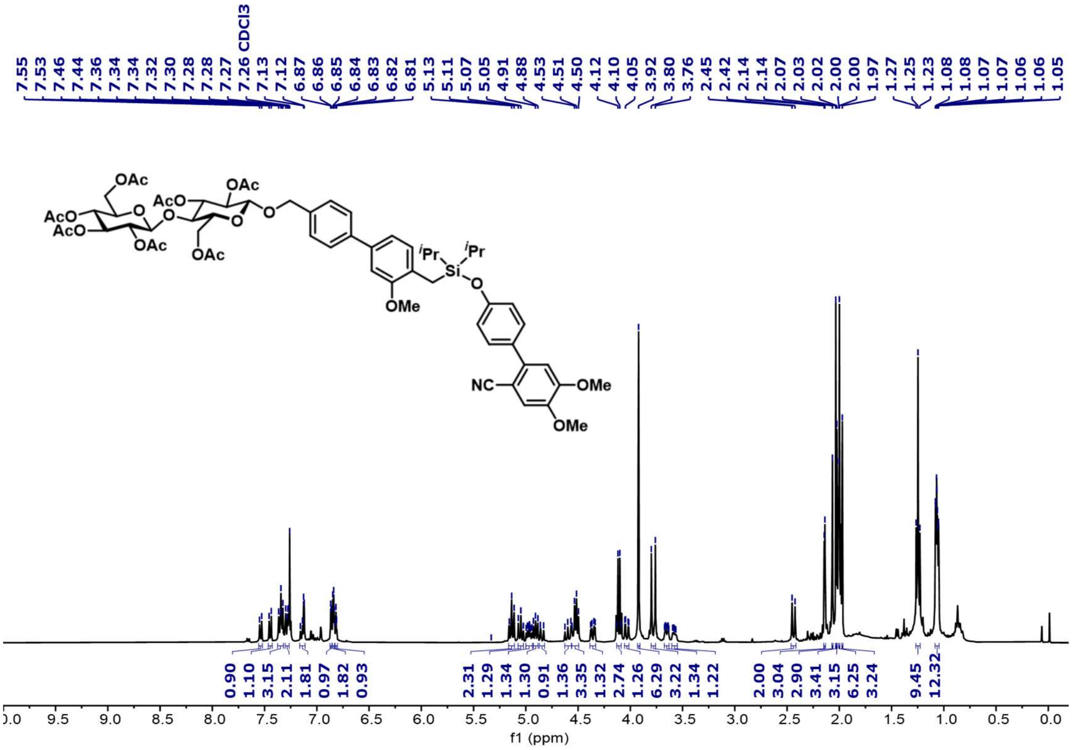

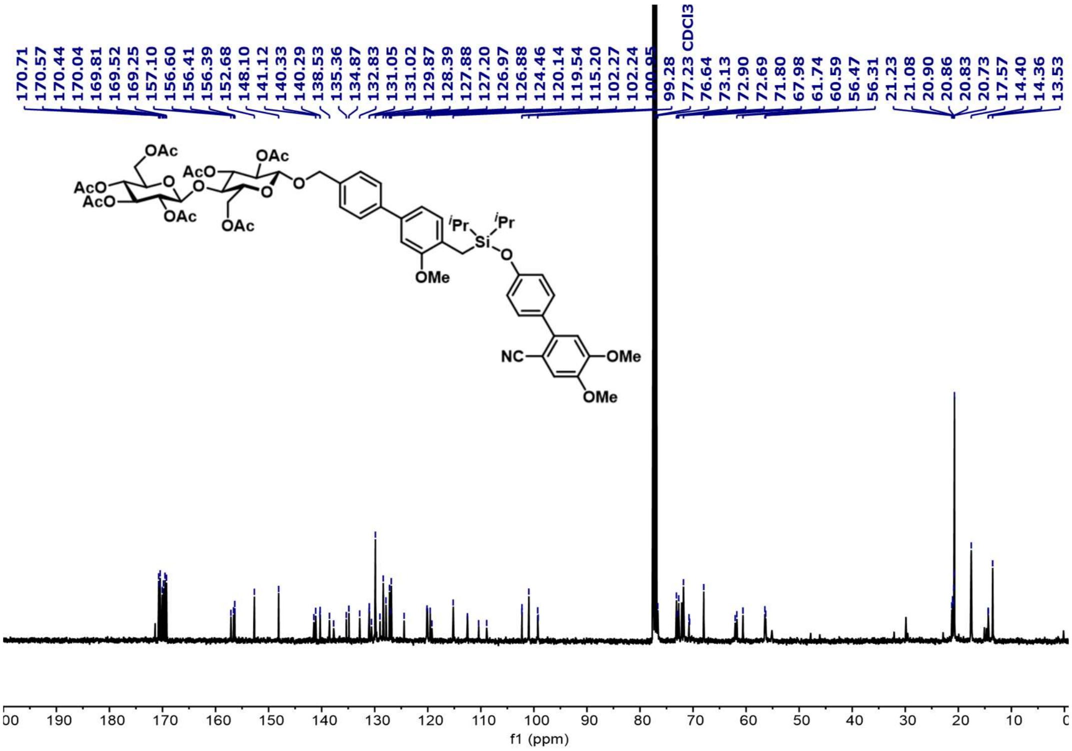

Supplementary Figure 80. ^{1}H (top) and ^{13}C (bottom) NMR of **5ch**

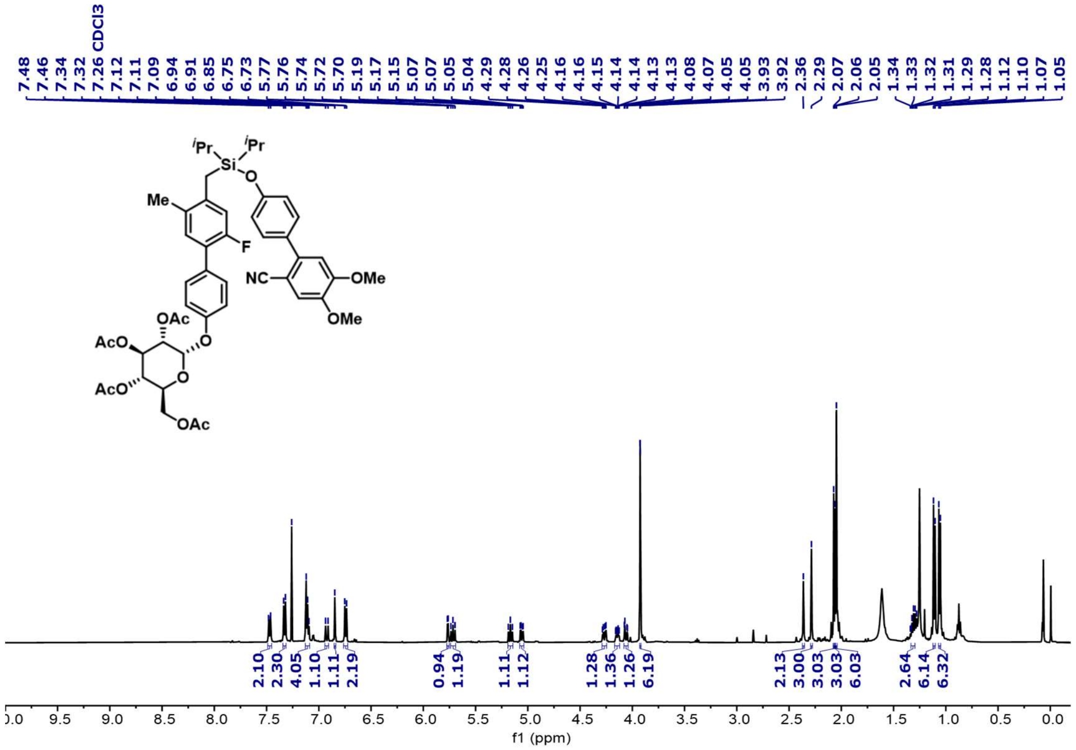

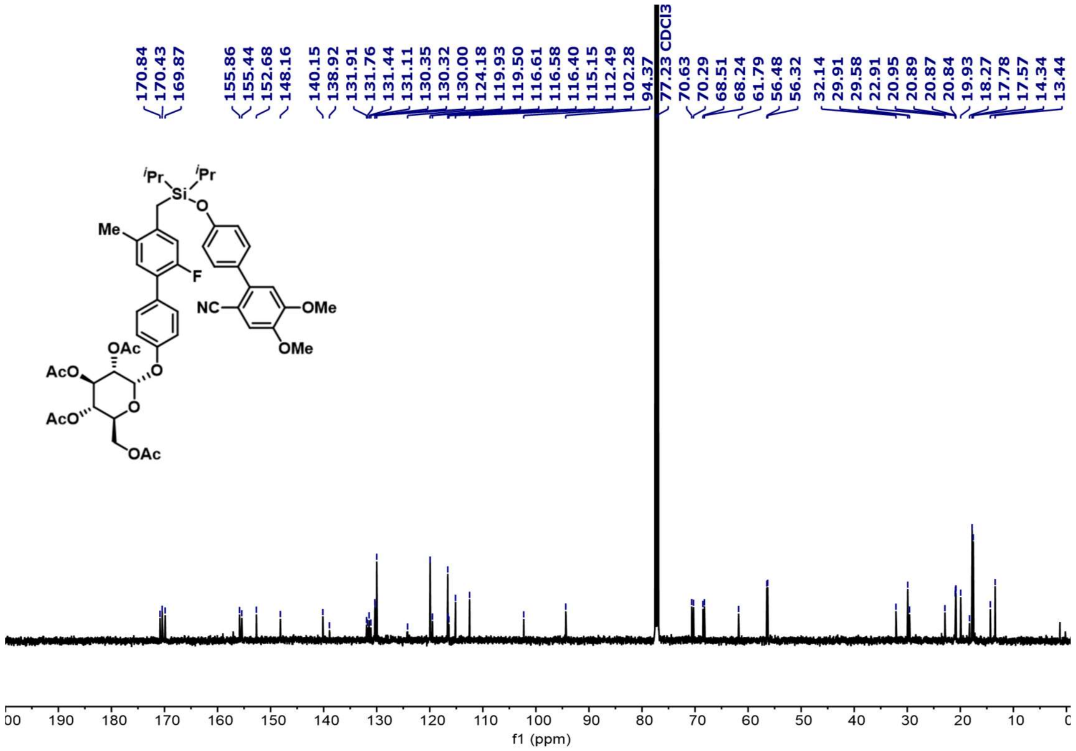

Supplementary Figure 81. ^{1}H (top) and ^{13}C (bottom) NMR of **5ni**

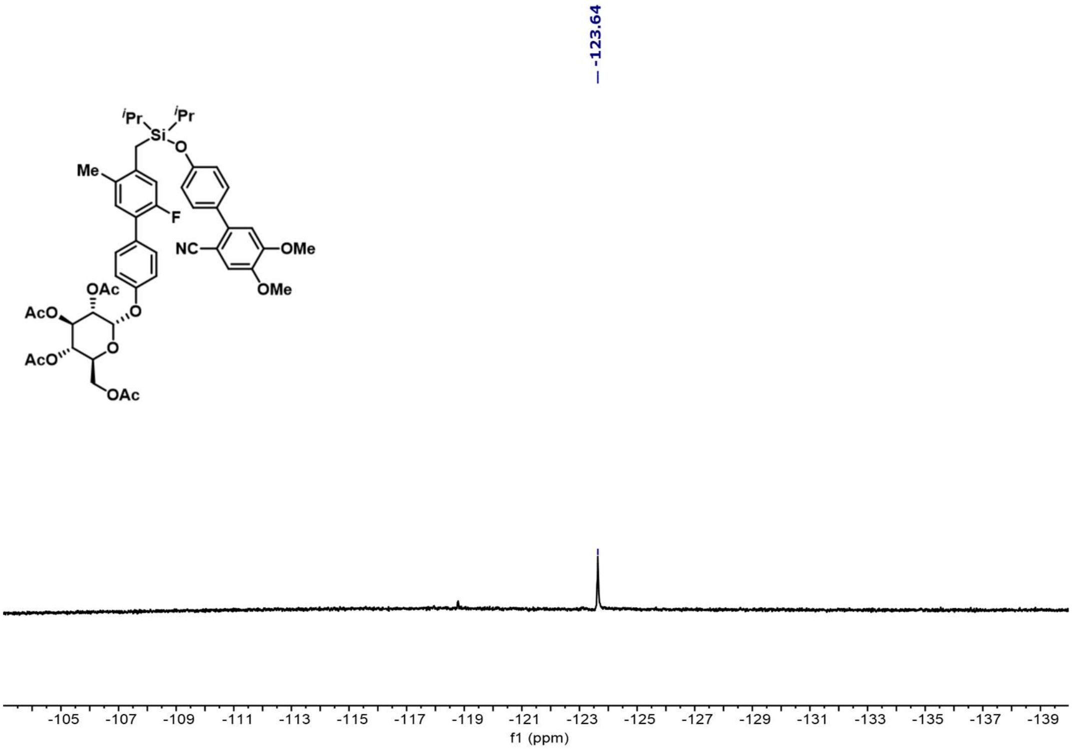

Supplementary Figure 82. ^{19}F NMR of **5ni**

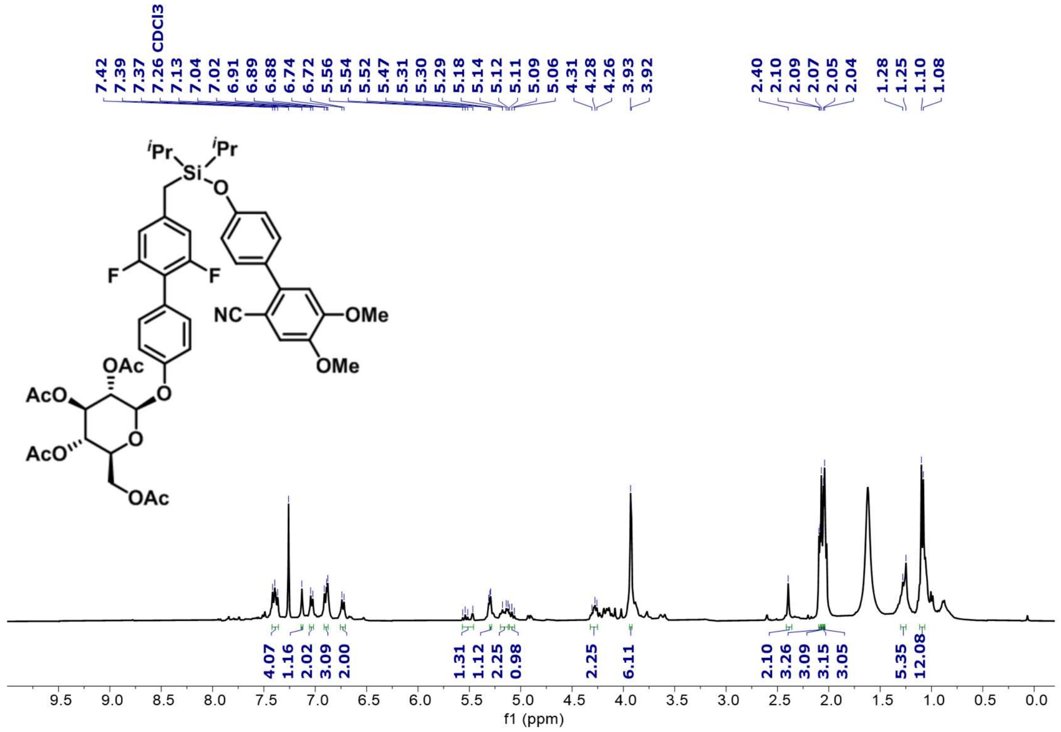

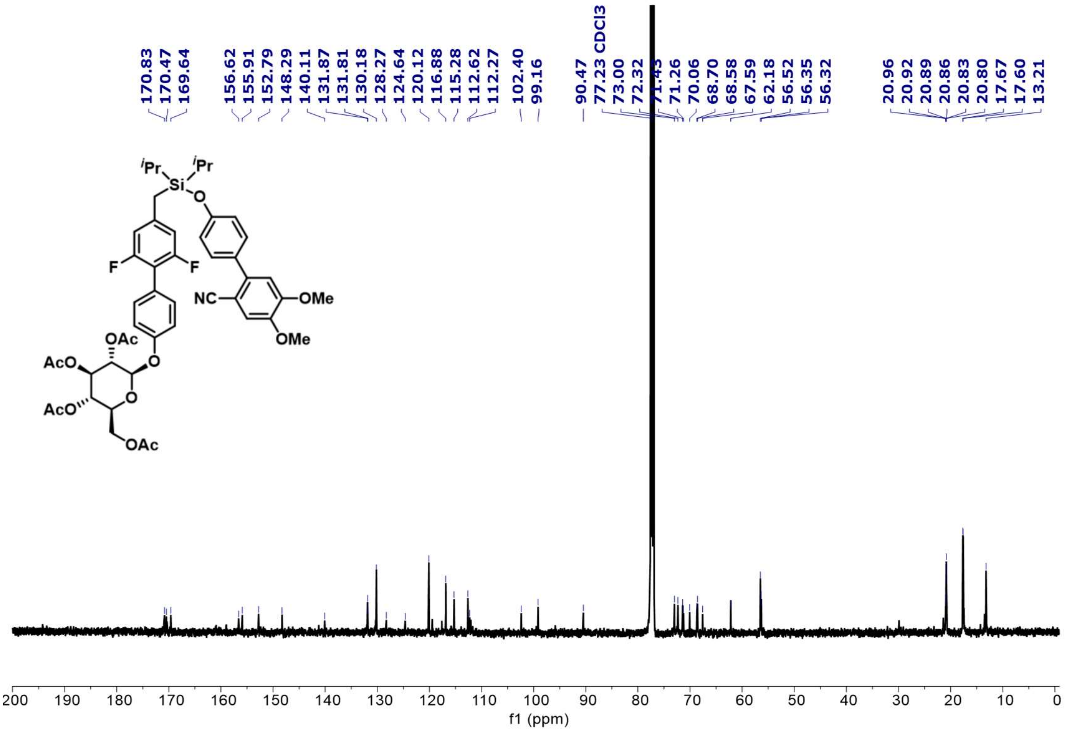

Supplementary Figure 83. ^{1}H (top) and ^{13}C (bottom) NMR of **5oj**

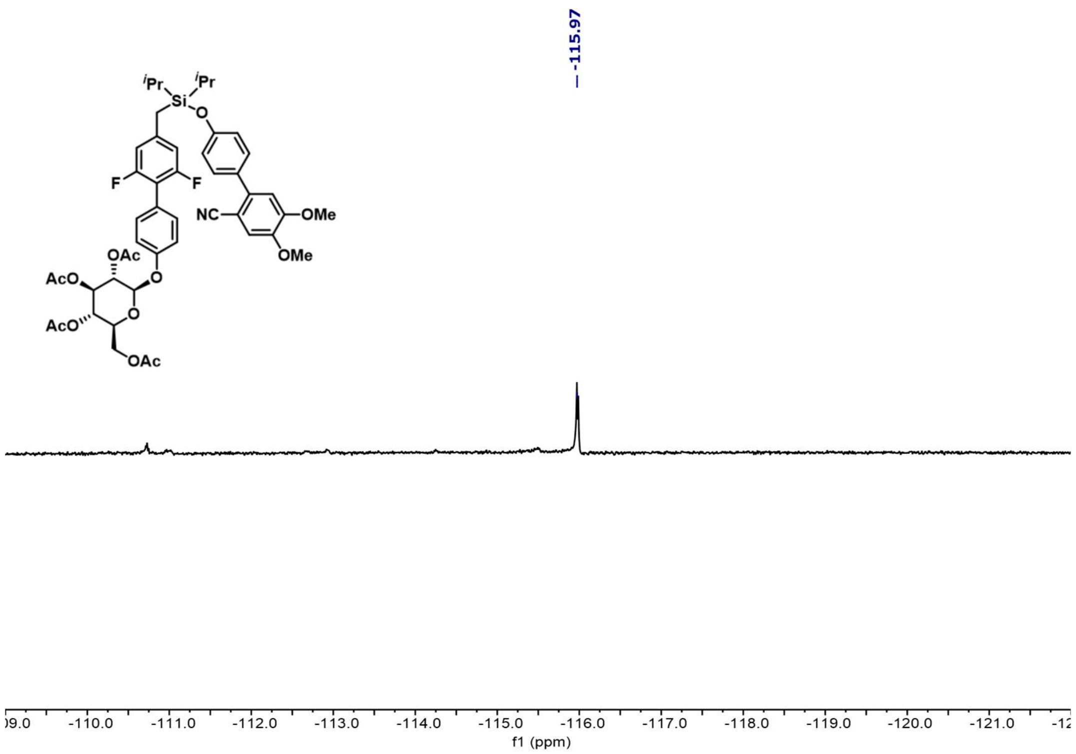

Supplementary Figure 84. ^{19}F NMR of **5oj**

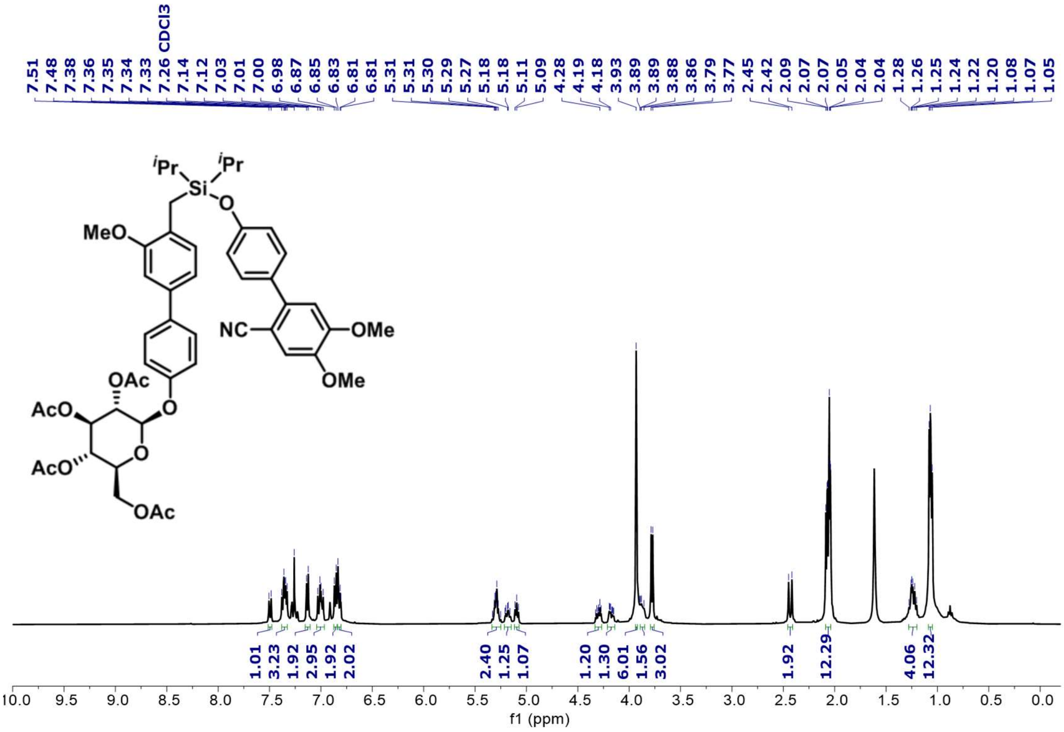

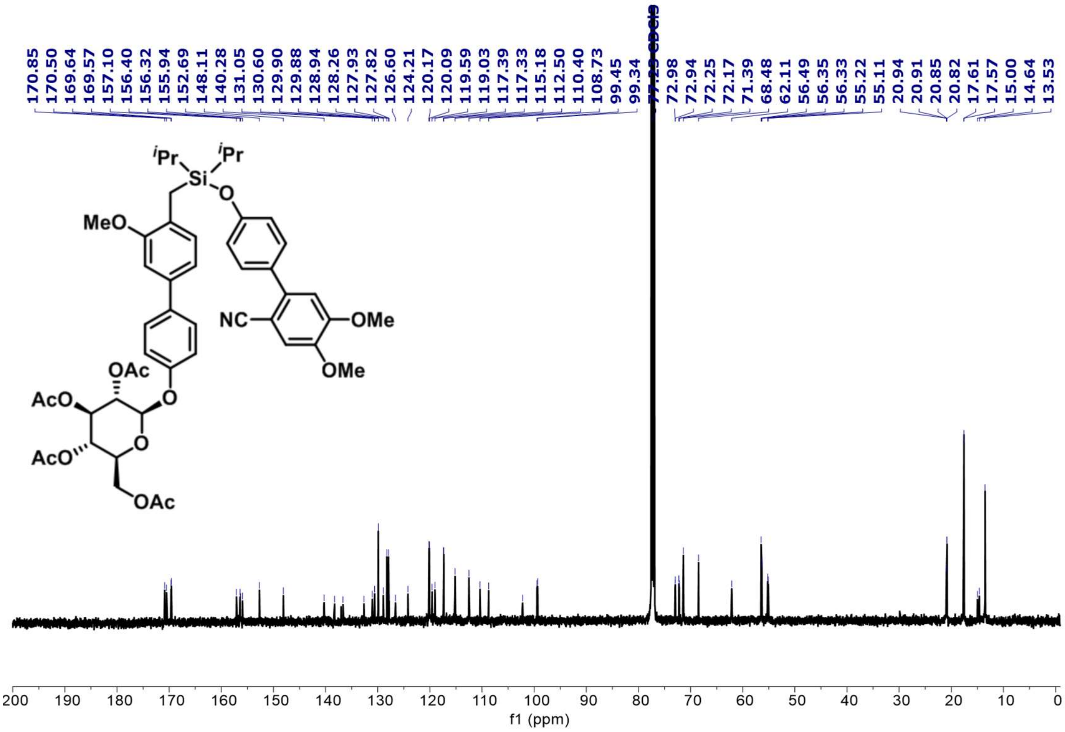

Supplementary Figure 85. ^{1}H (top) and ^{13}C (bottom) NMR of **5cj**

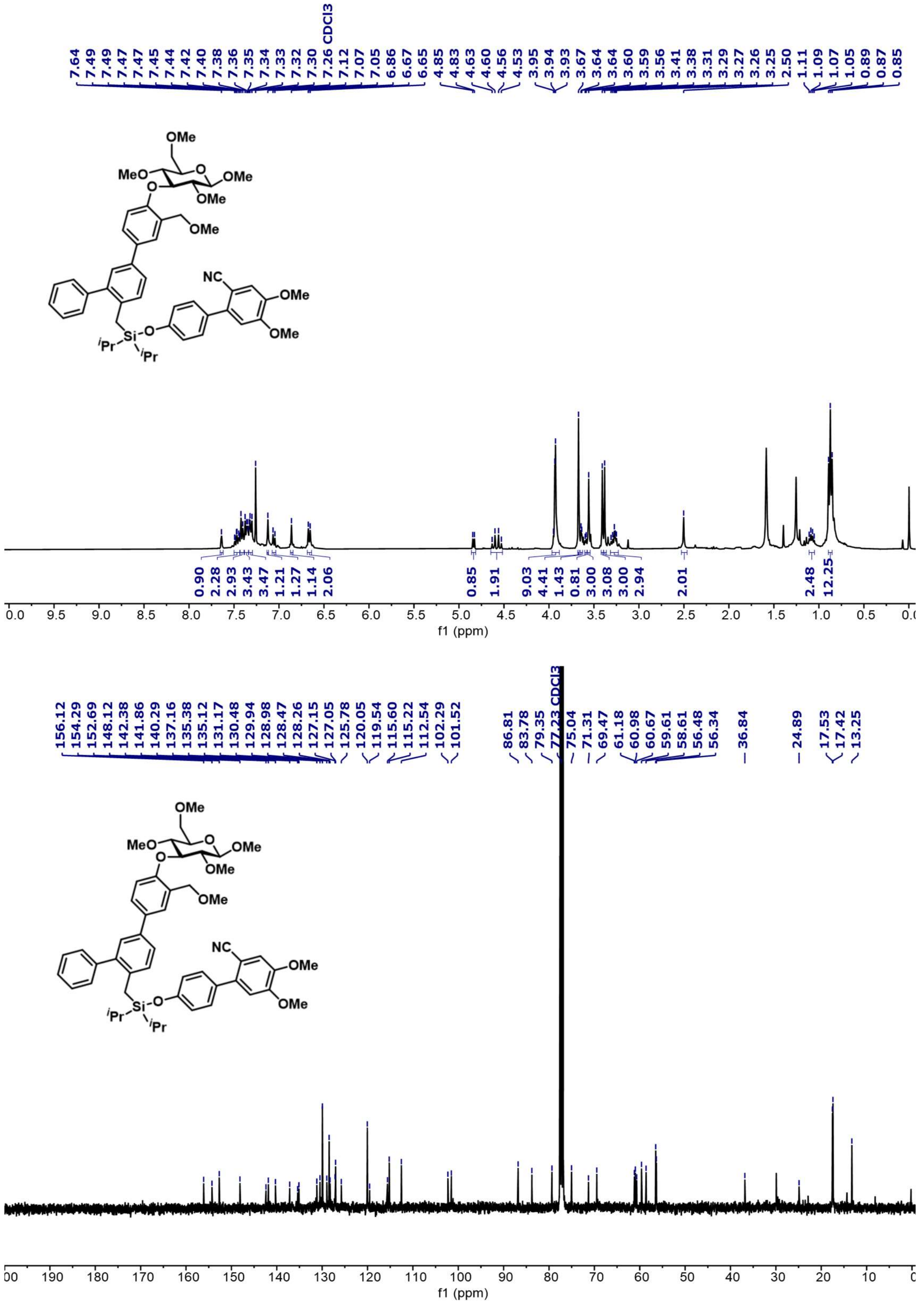

Supplementary Figure 86. ^{1}H (top) and ^{13}C (bottom) NMR of **5kk**

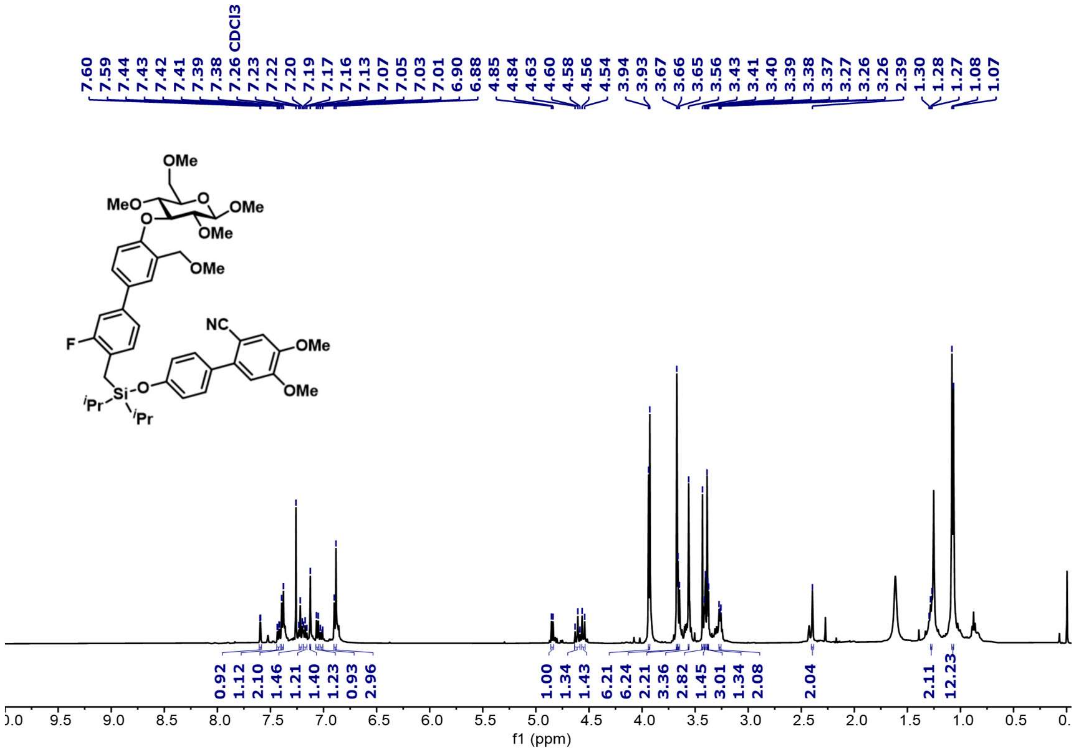

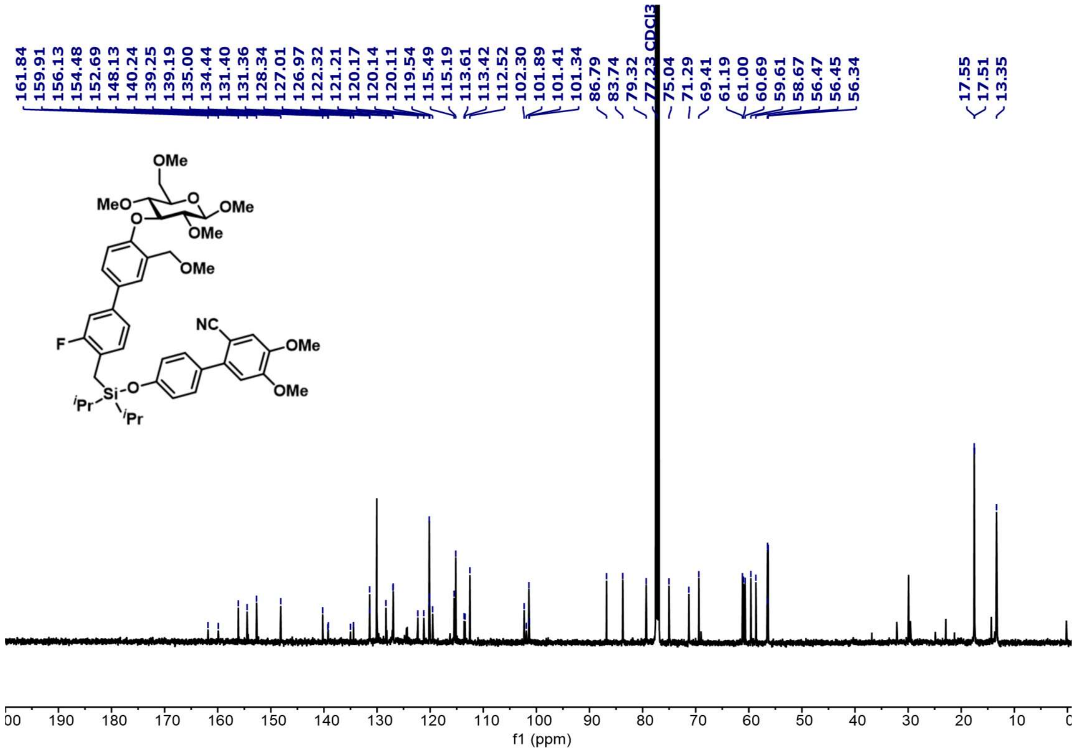

Supplementary Figure 87. ^{1}H (top) and ^{13}C (bottom) NMR of **5uk**

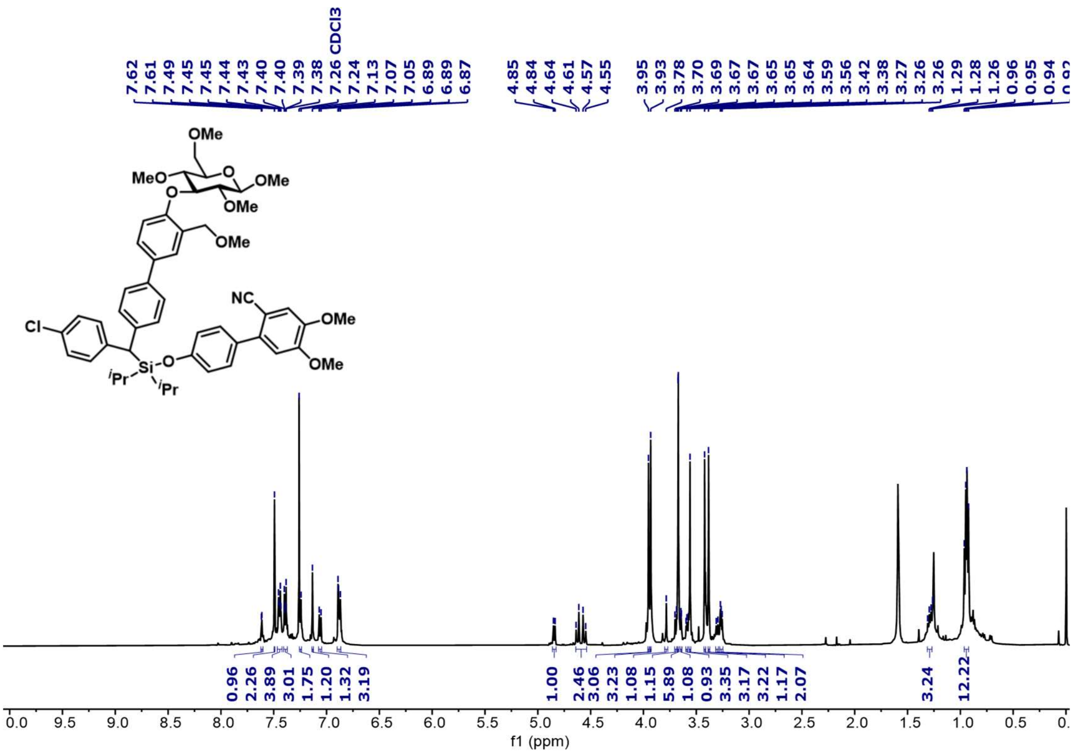

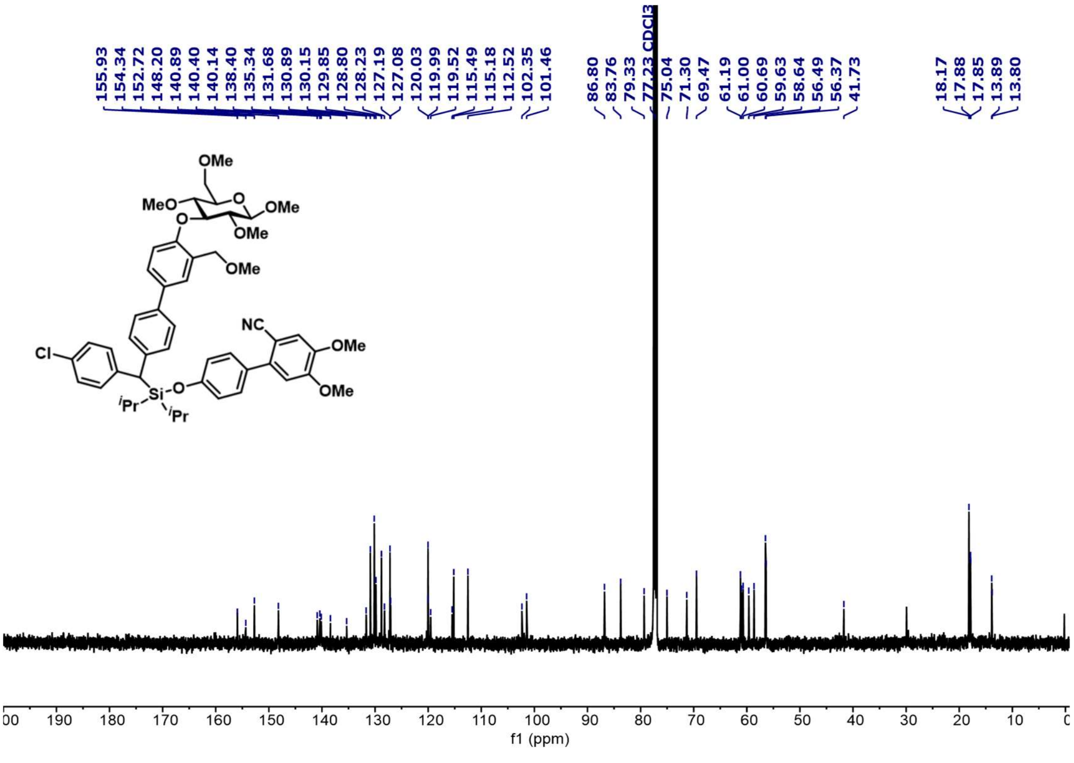

Supplementary Figure 88. ^{1}H (top) and ^{13}C (bottom) NMR of **5sk**

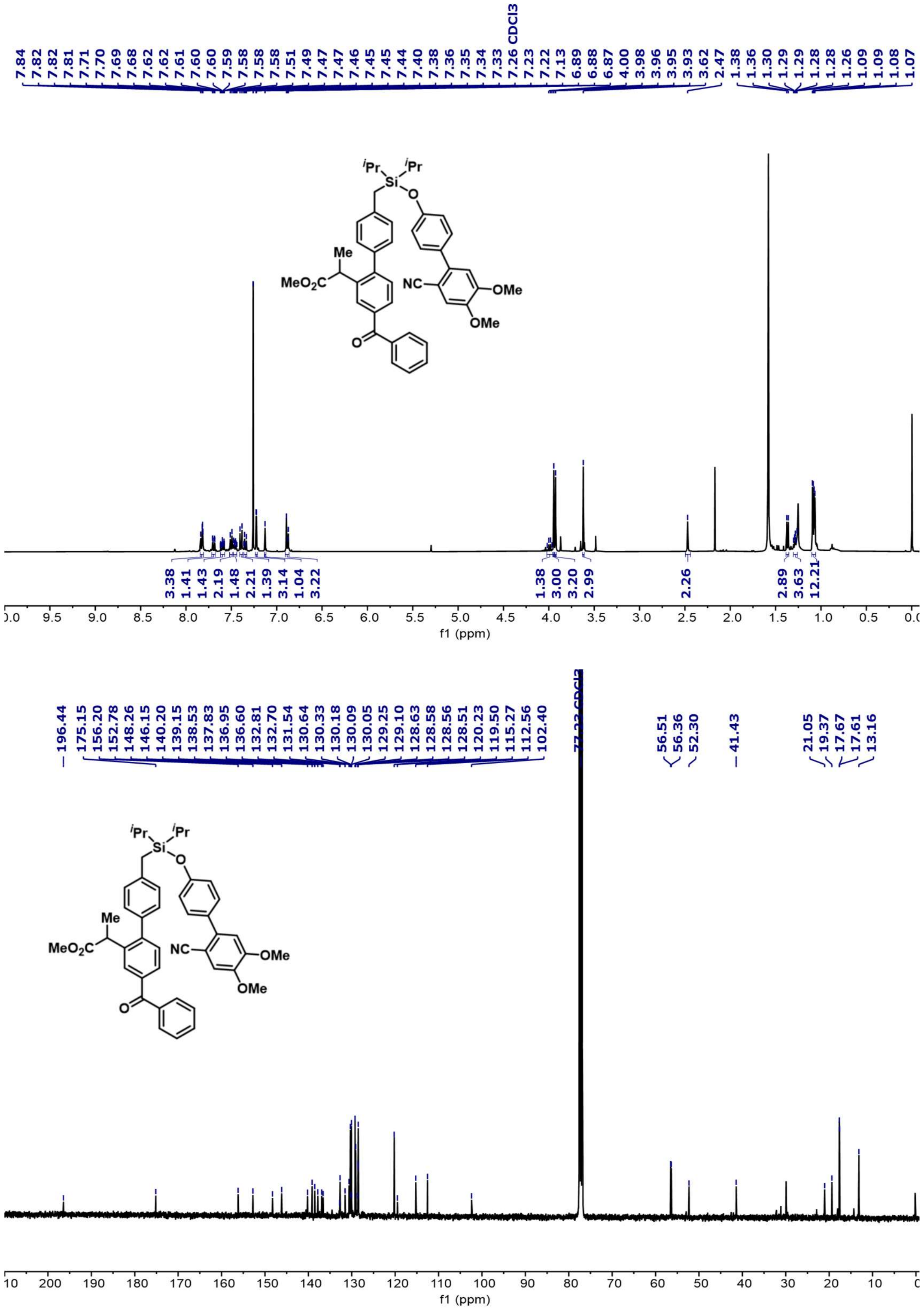

Supplementary Figure 89. ^{1}H (top) and ^{13}C (bottom) NMR of **5al**

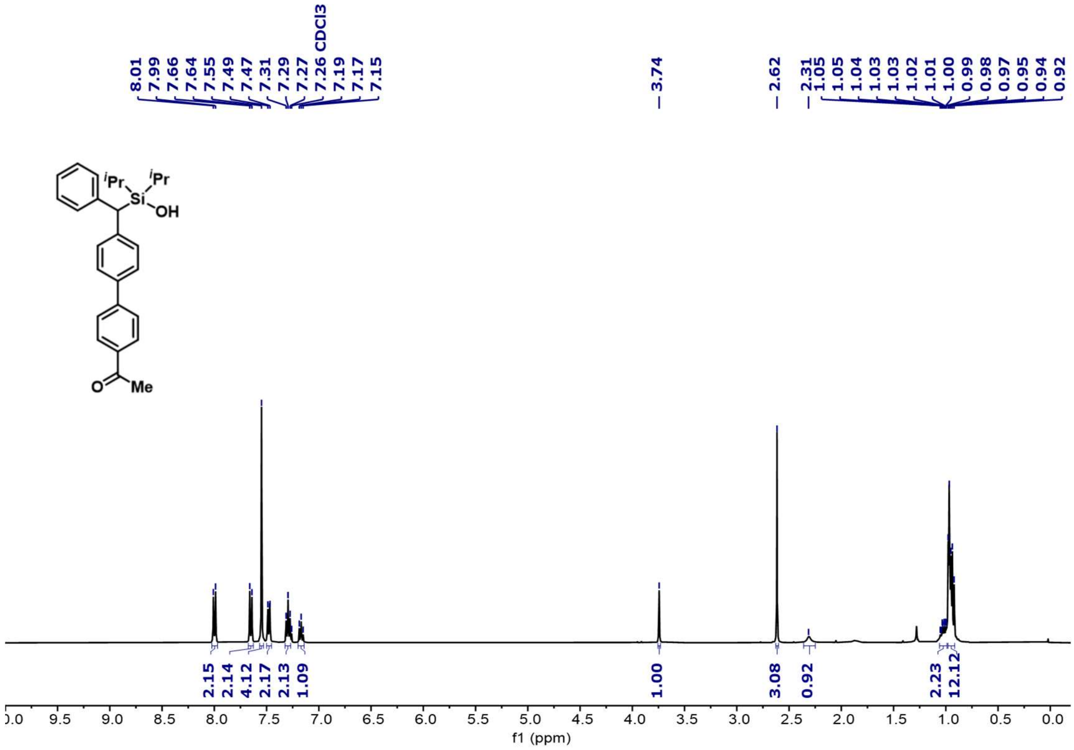

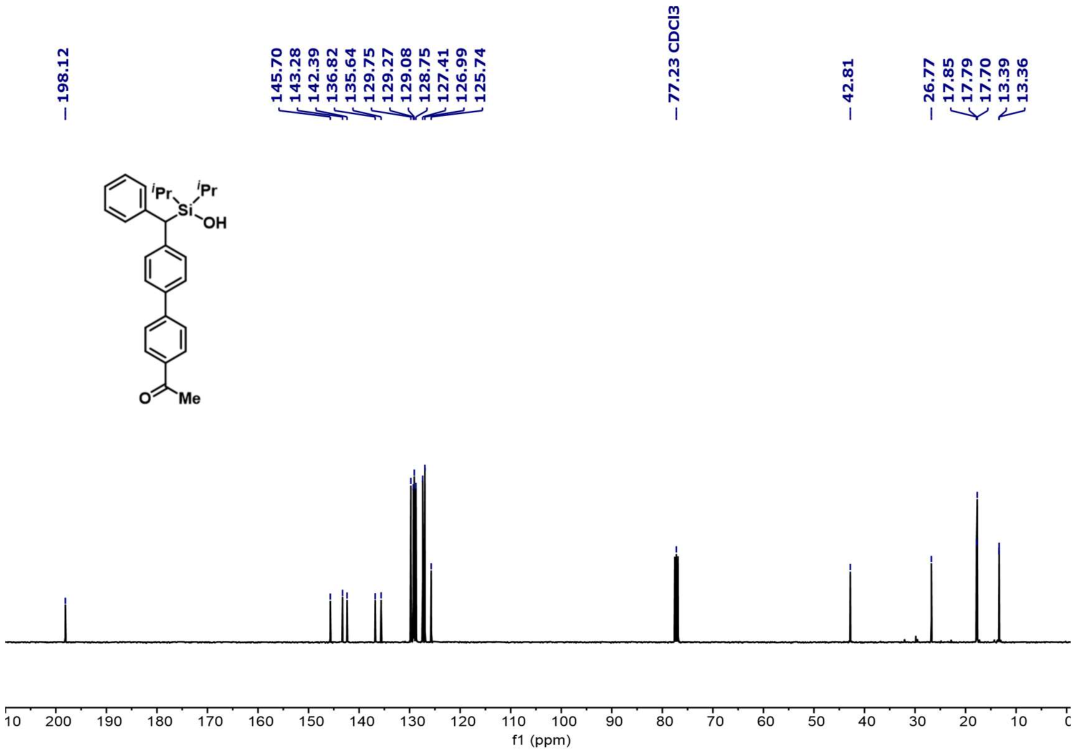

Supplementary Figure 90. ^{1}H (top) and ^{13}C (bottom) NMR of **6**

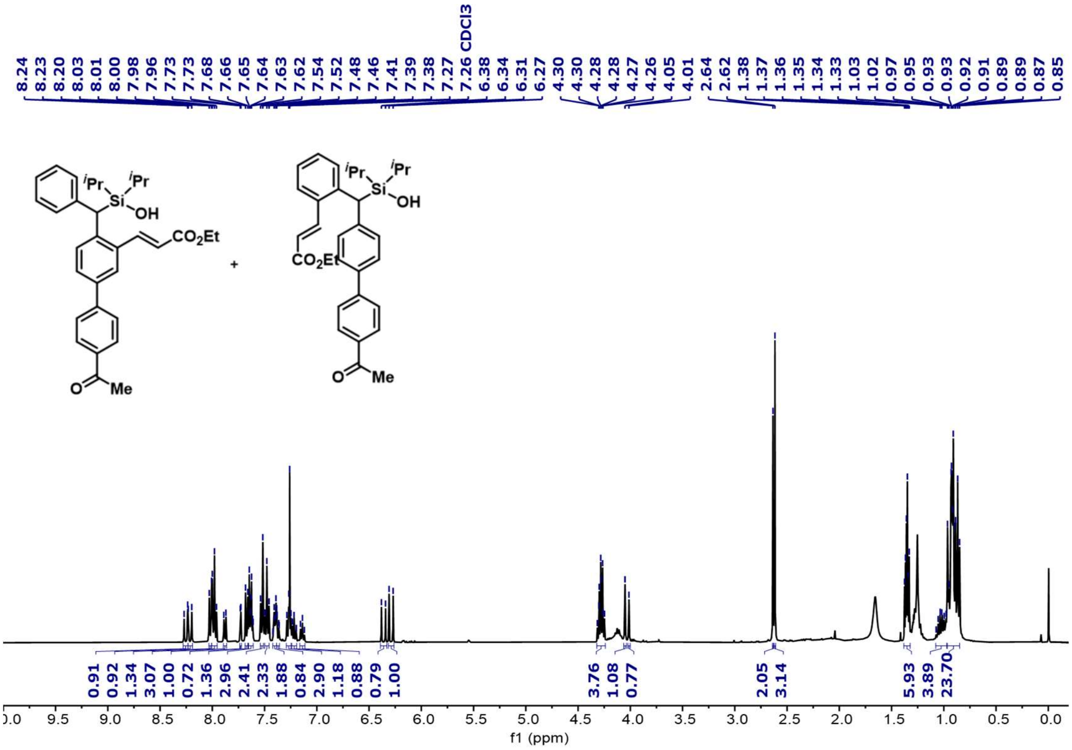

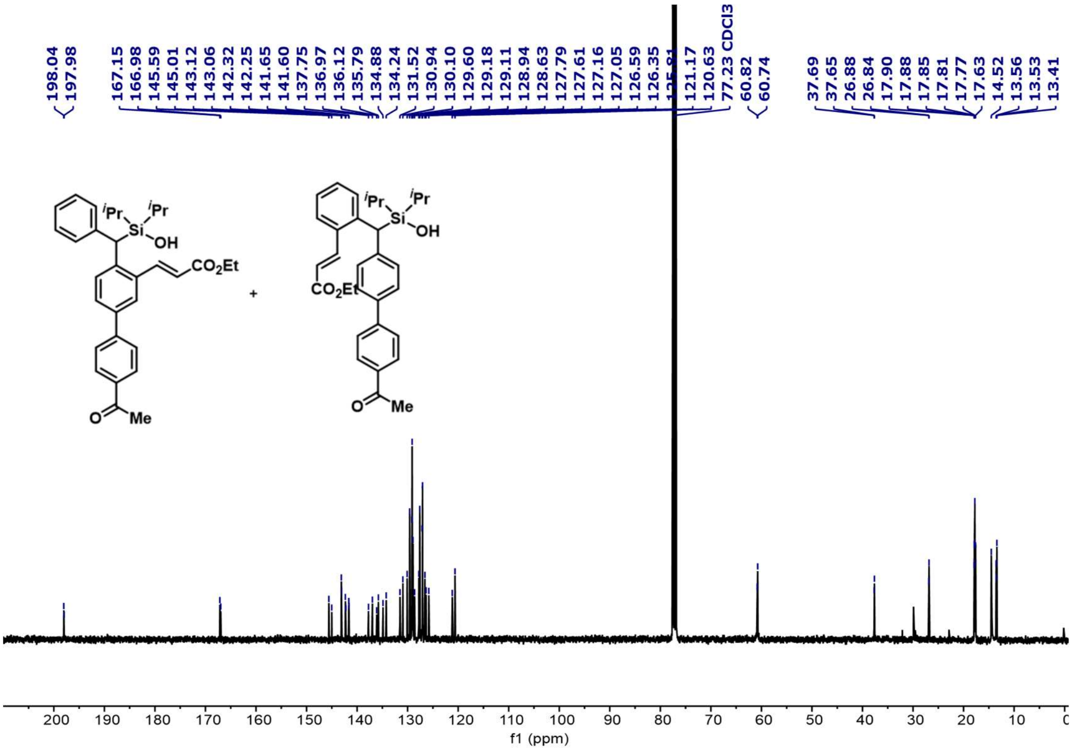

Supplementary Figure 91. ^{1}H (top) and ^{13}C (bottom) NMR of **7** and **8**

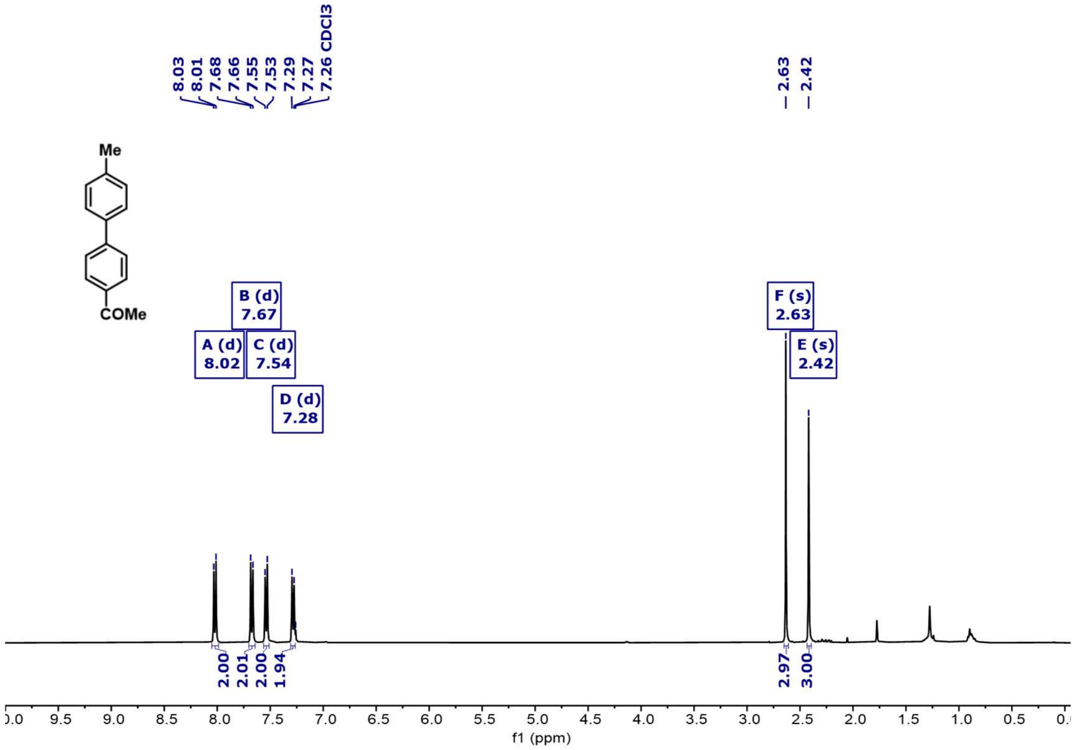

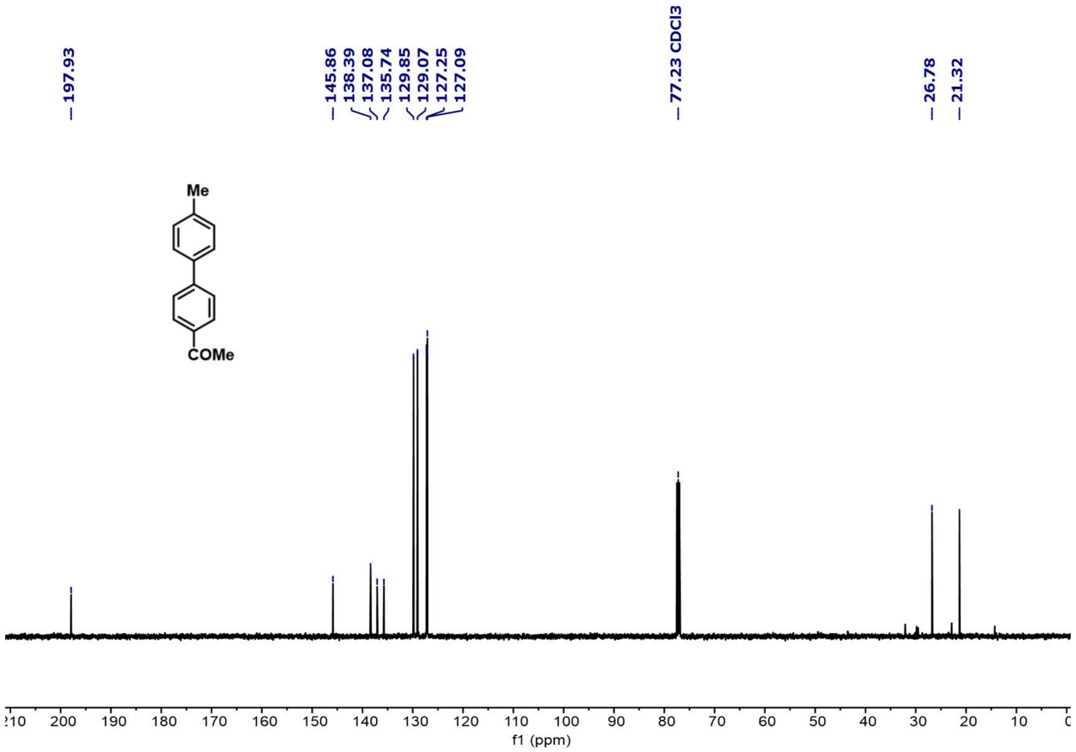

Supplementary Figure 92. ^{1}H (top) and ^{13}C (bottom) NMR of **9**

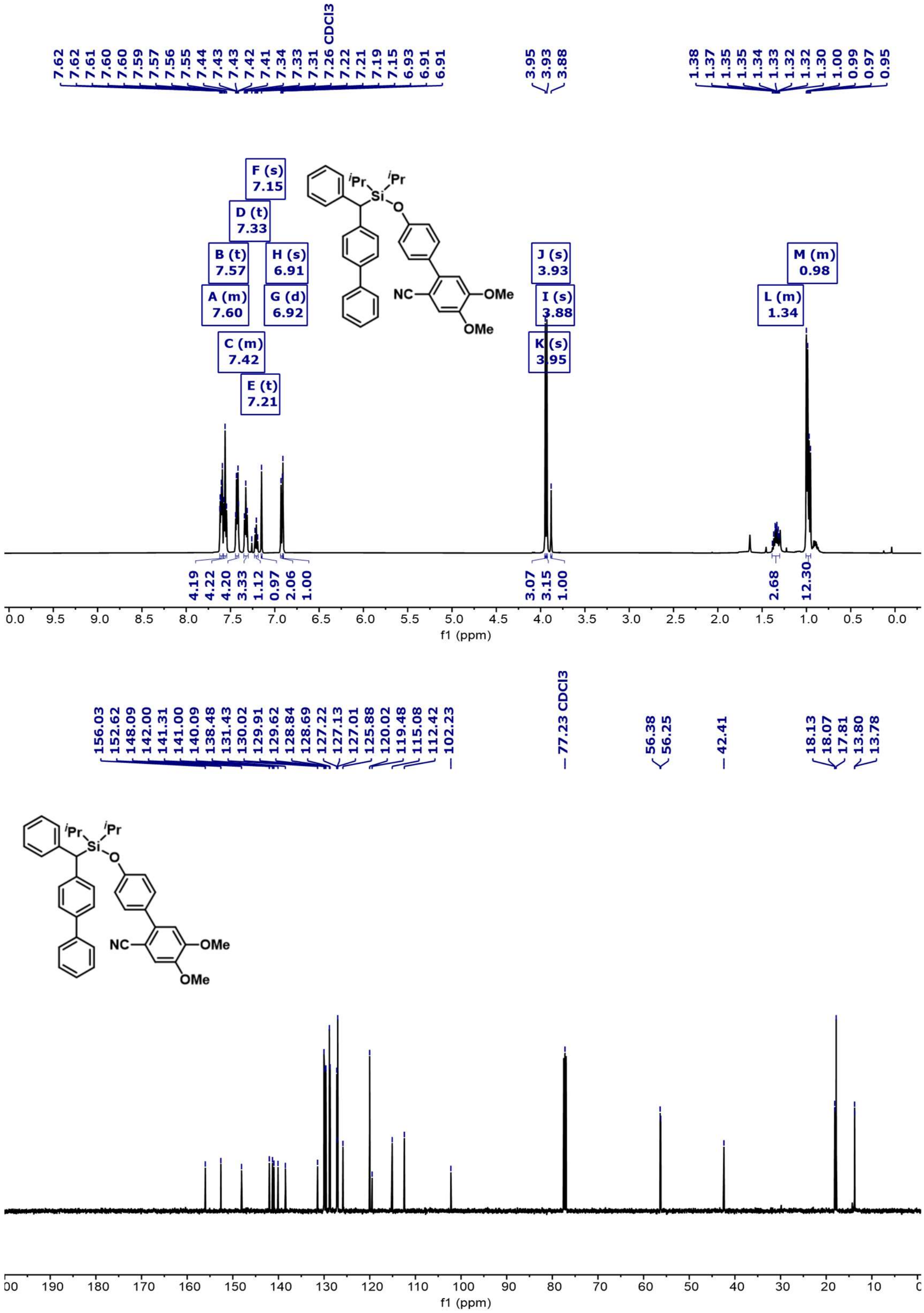

Supplementary Figure 93. ^{1}H (top) and ^{13}C (bottom) NMR of **3qz'**

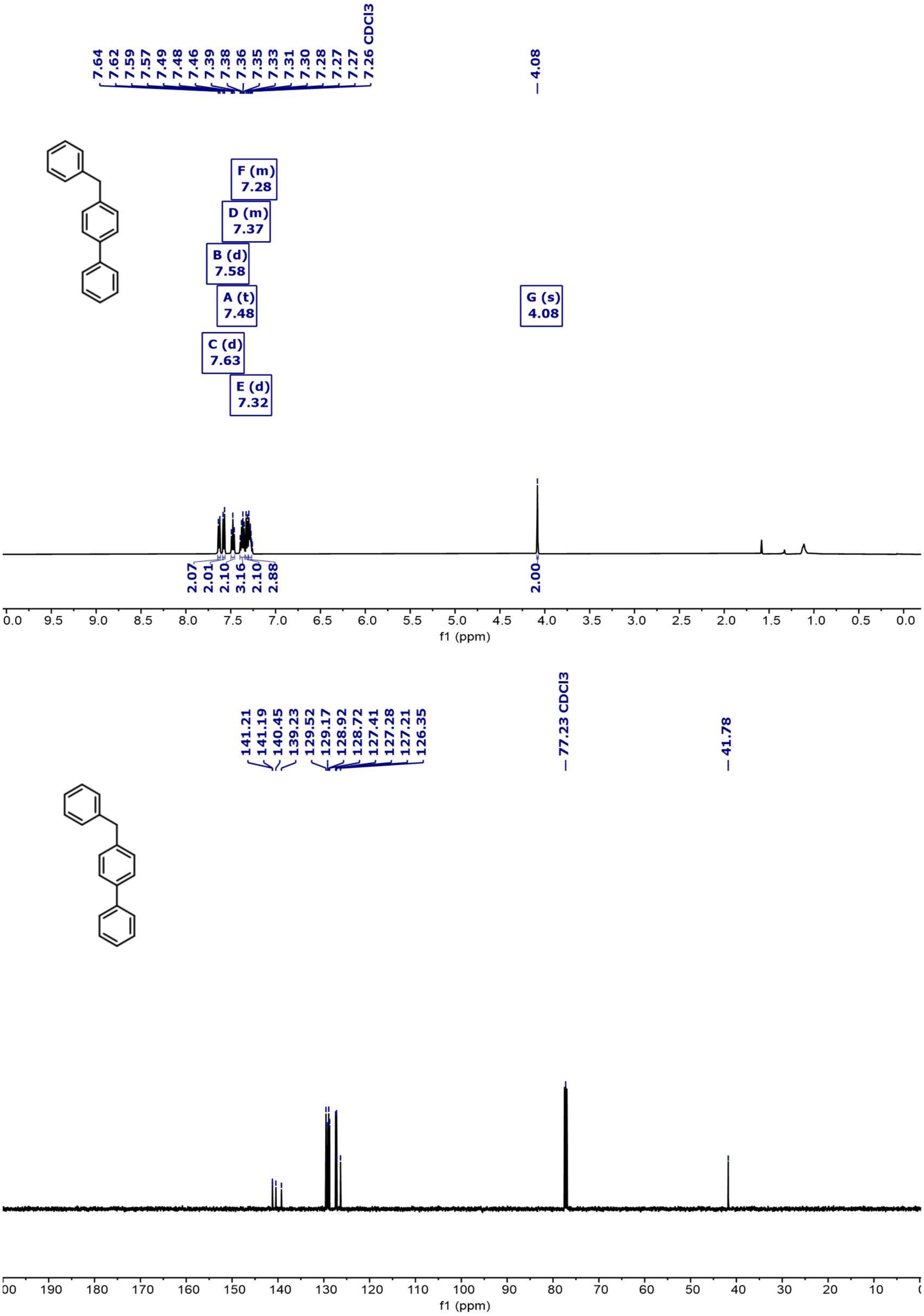

Supplementary Figure 94. ^{1}H (top) and ^{13}C (bottom) NMR of **10**

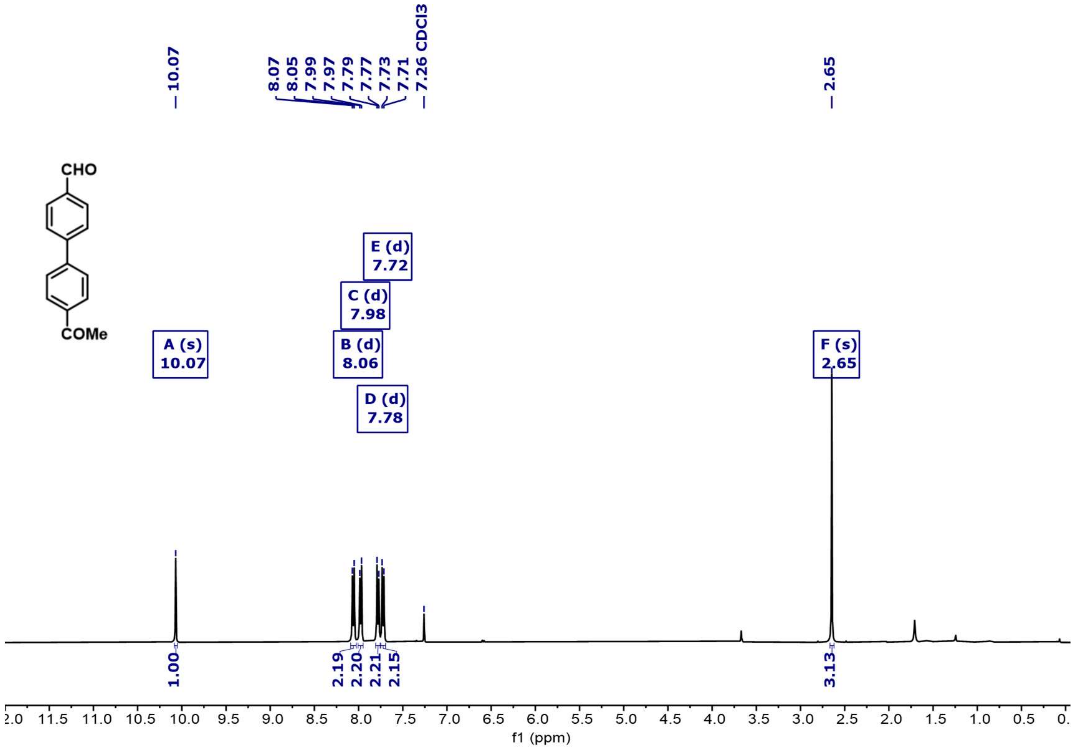

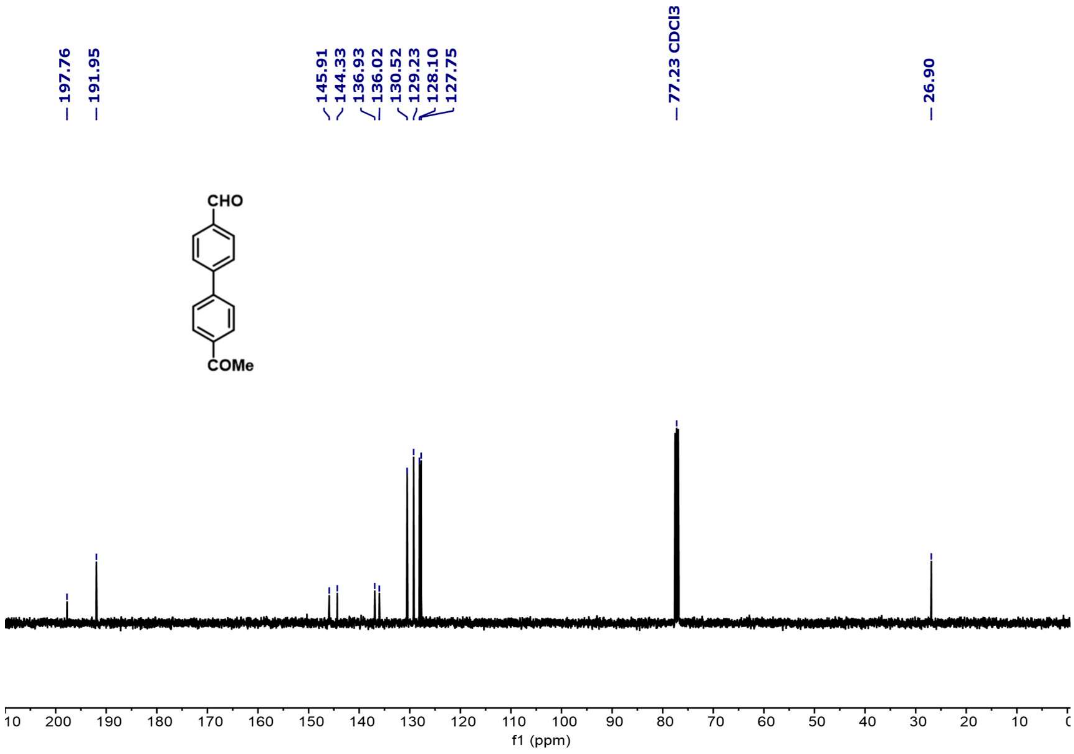

Supplementary Figure 95. ^{1}H (top) and ^{13}C (bottom) NMR of **11**

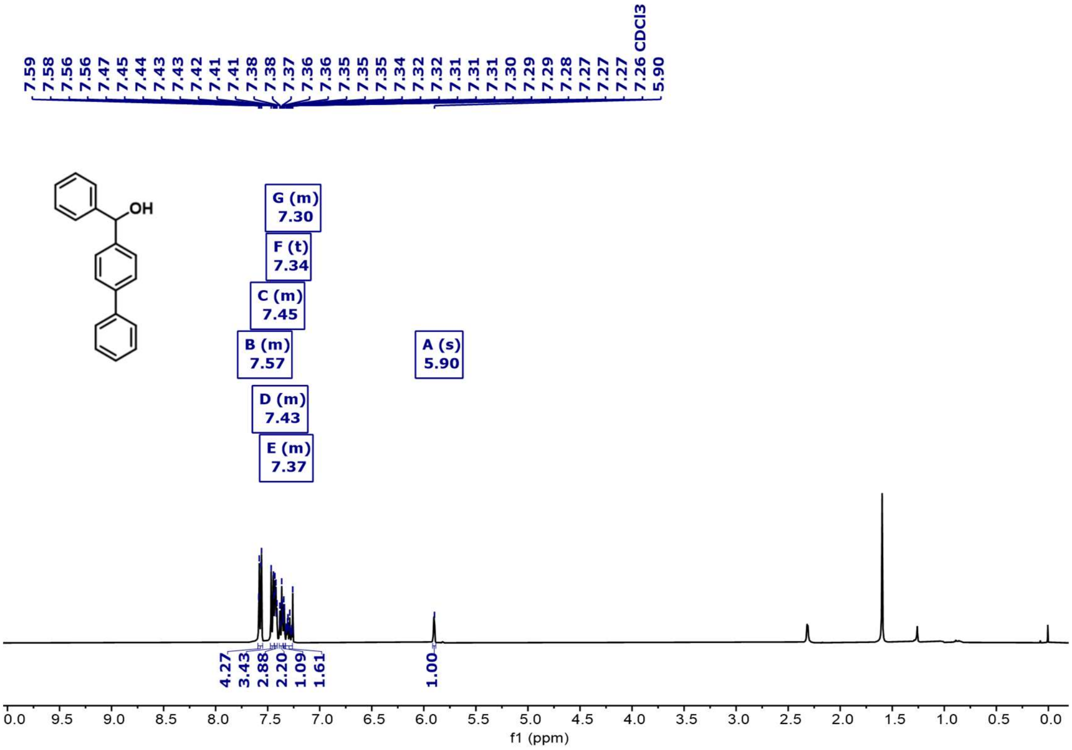

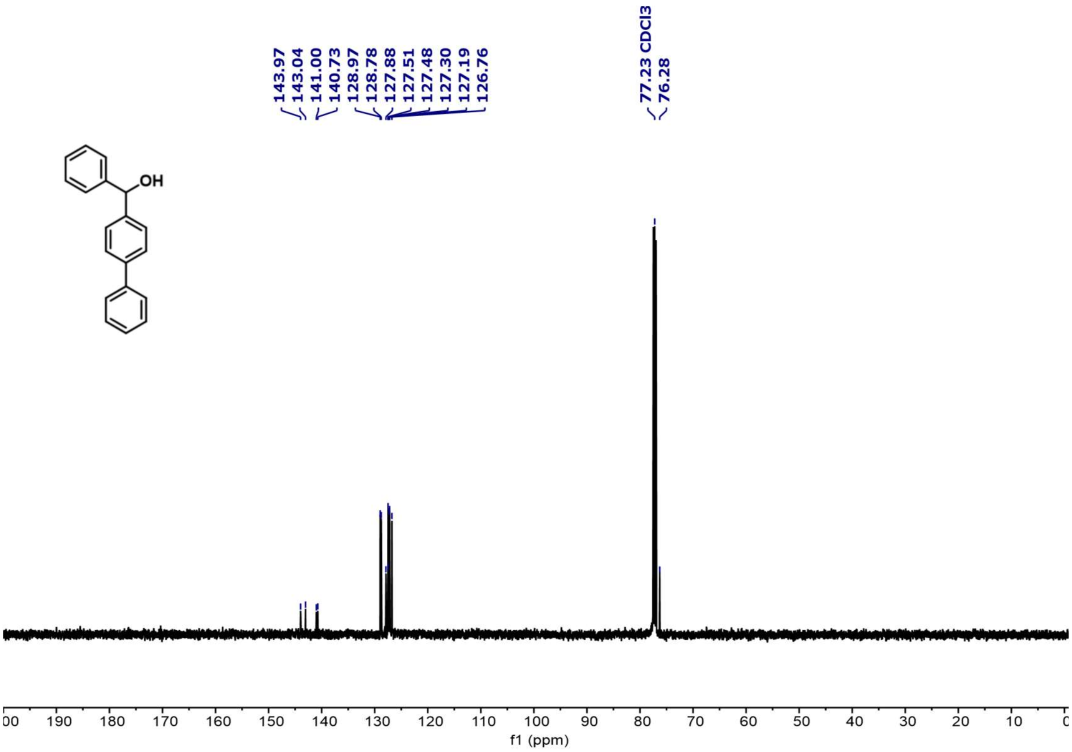

Supplementary Figure 96. ^{1}H (top) and ^{13}C (bottom) NMR of **12**

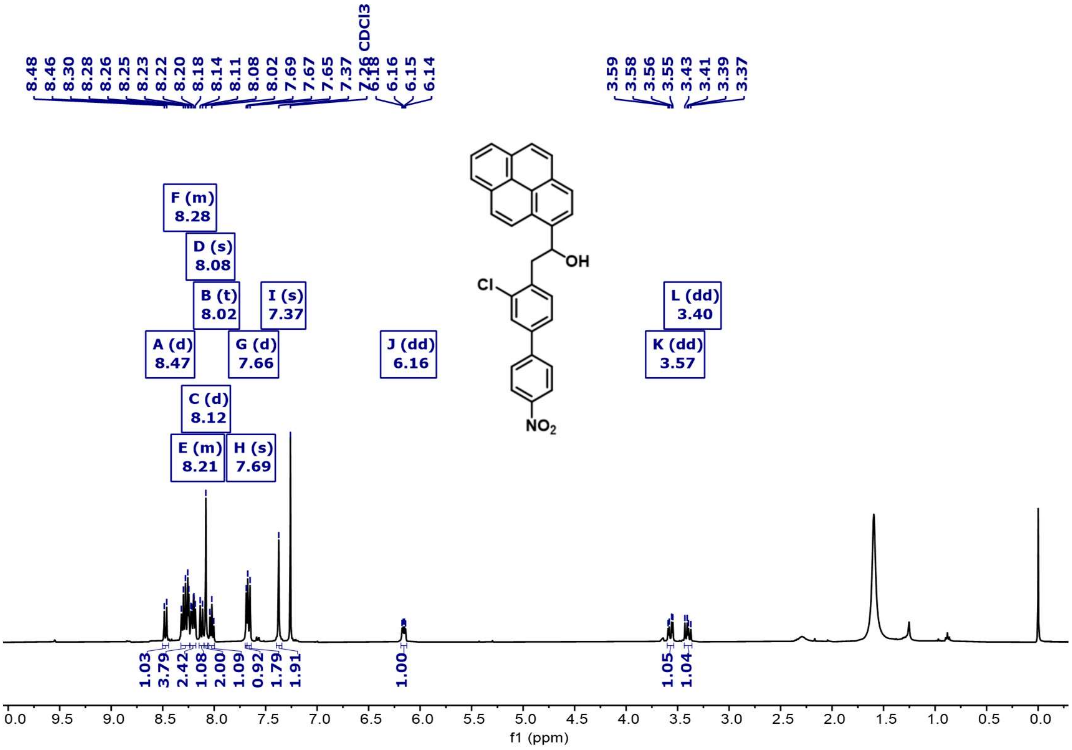

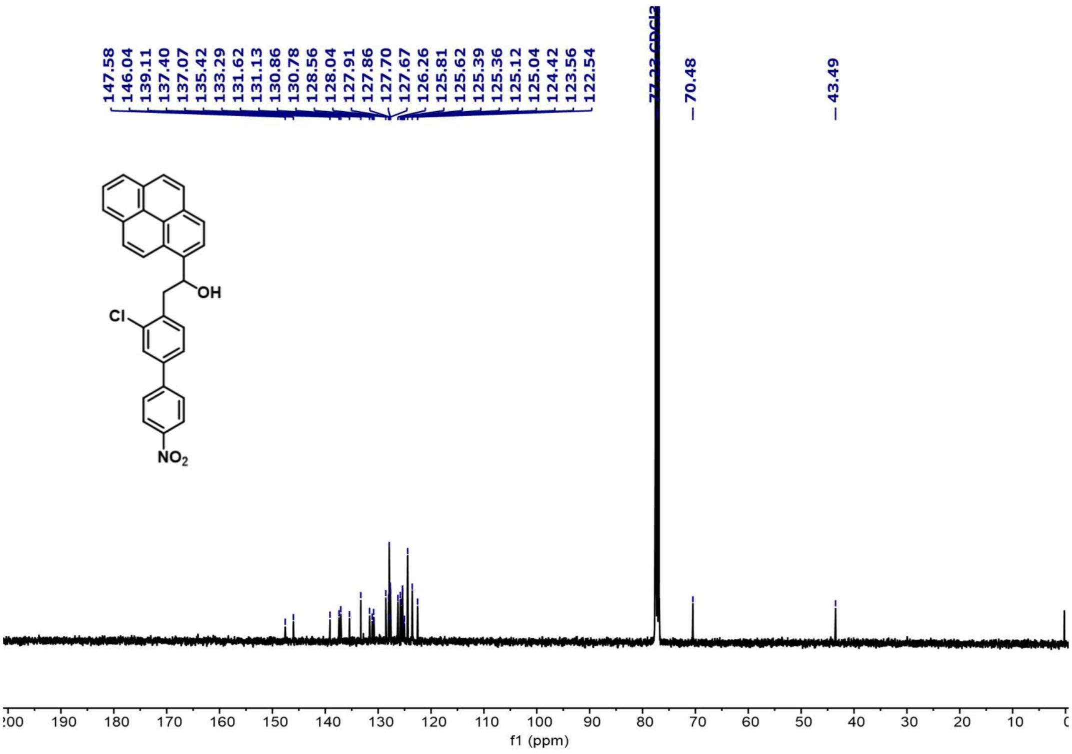

Supplementary Figure 97. ^{1}H (top) and ^{13}C (bottom) NMR of **14**

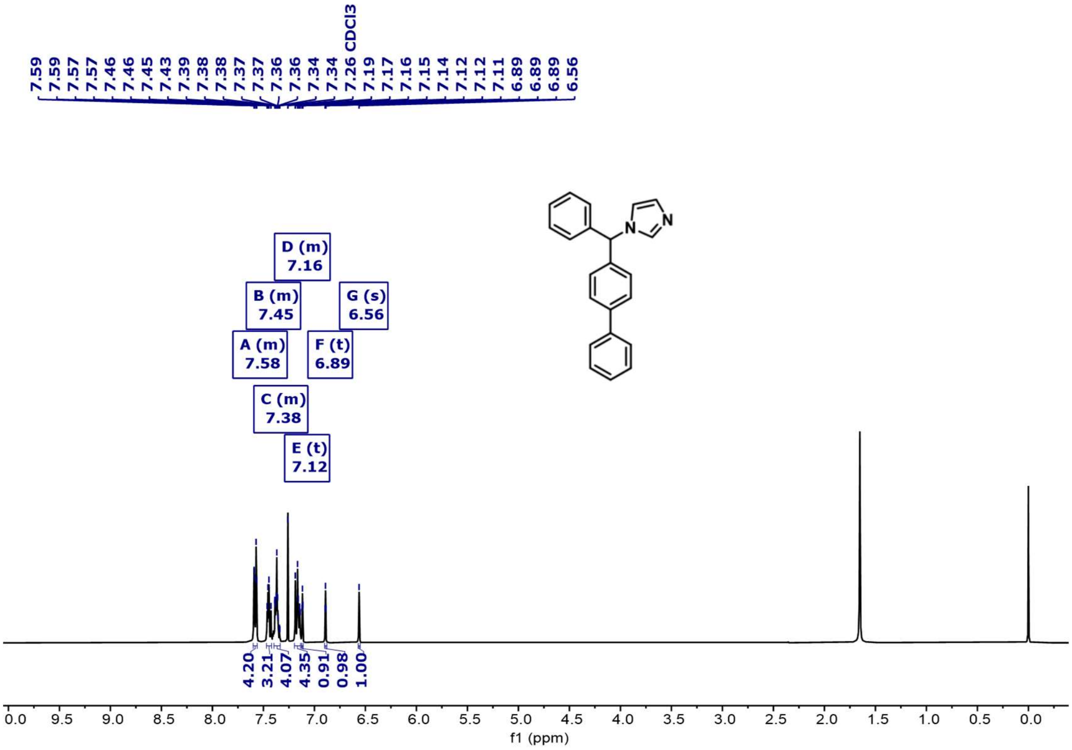

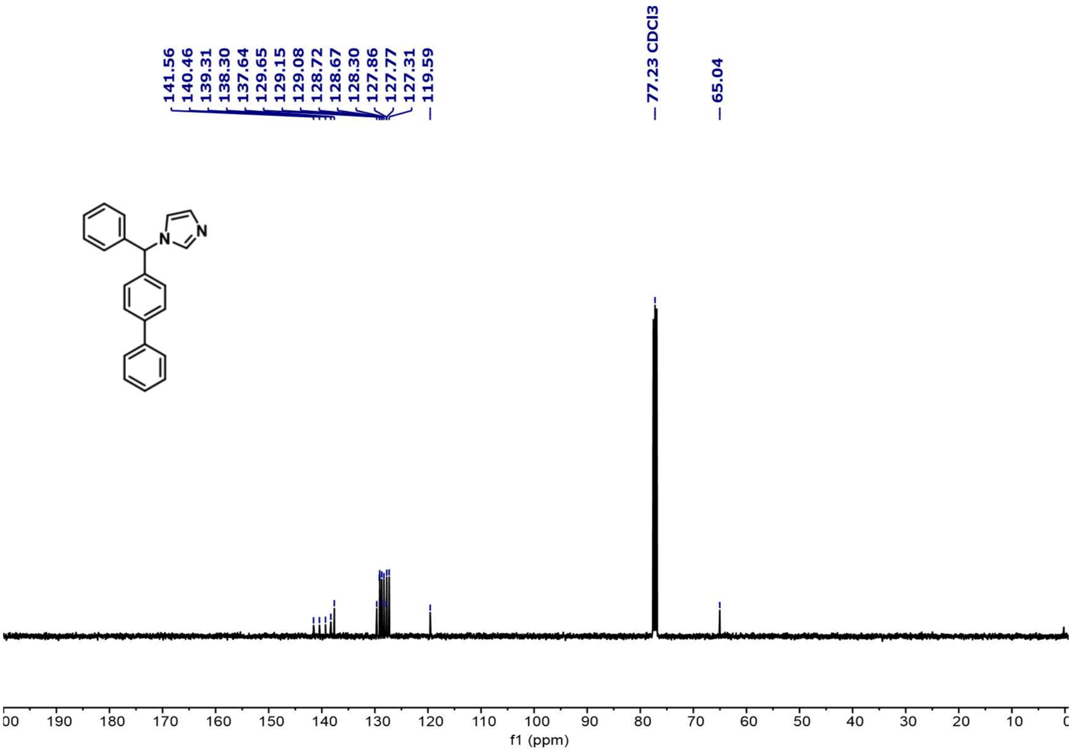

Supplementary Figure 98. ^{1}H (top) and ^{13}C (bottom) NMR of **15**

Supplementary References

1. Rokade, B. V. & Prabhu, K. R. Chemoselective Schmidt Reaction Mediated by Triflic Acid: Selective Synthesis of Nitriles from Aldehydes. *J. Org. Chem.* **77**, 5364–5370 (2012).
2. Bag, S., Patra, T., Modak, A., Deb, A., Maity, S., Dutta, U., Dey, A., Kancherla, R., Maji, A., Hazra, A., Bera, M. & Maiti, D. Remote *para*-C−H Functionalization of Arenes by a D-Shaped Biphenyl Template-Based Assembly. *J. Am. Chem. Soc.* **137**, 11888–11891 (2015).
3. Maji, A., Guin, S., Feng, S., Dahiya, A., Singh, V. K., Liu, P. & Maiti, D. Experimental and Computational Exploration of *para*-Selective Silylation with a Hydrogen-Bonded Template. *Angew. Chem. Int. Ed.* **56**, 14903 –14907 (2017).
4. Dutta, U., Maiti, S., Pimparkar, S., Maiti, S., Gahan, L. R., Krenske, E. H., Lupton, D. W. & Maiti, D. Rhodium Catalyzed Template-Assisted Distal *para*-C−H Olefination. *Chem. Sci.* **10**, 7426-7432 (2019).
5. Jin, S., Dang, H. T., Haug, G. C., He, R., Nguyen, V. D., Nguyen, V. T., Arman, H. D., Schanze, K. S. & Larionov, O. V. Visible Light-Induced Borylation of C−O, C−N, and C−X Bonds. *J. Am. Chem. Soc.* **142**, 1603–1613 (2020).
6. Wang, C. & Ge, H. Silanol as a Removable Directing Group for the Pd(II)-Catalyzed Direct Olefination of Arenes. *Chem. Eur. J.* **17**, 14371-14374 (2011).
7. Gaussian 16, Rev. B.01, Frisch, M. J.; Trucks, G. W.; Schlegel, H. B.; Scuseria, G. E.; Robb, M. A.; Cheeseman, J. R.; Scalmani, G.; Barone, V.; Petersson, G. A.; Nakatsuji, H.; Li, X.; Caricato, M.; Marenich, A. V.; Bloino, J.; Janesko, B. G.; Gomperts, R.; Mennucci, B.; Hratchian, H. P.; Ortiz, J. V.; Izmaylov, A. F.; Sonnenberg, J. L.; Williams-Young, D.; Ding, F.; Lipparini, F.; Egidi, F.; Goings, J.; Peng, B.; Petrone, A.; Henderson, T.; Ranasinghe, D.; Zakrzewski, V. G.; Gao, J.; Rega, N.; Zheng, G.; Liang, W.; Hada, M.; Ehara, M.; oyota, K.; Fukuda, R.; Hasegawa, J.; Ishida, M.; Nakajima, T.; Honda, Y.; Kitao, O.; Nakai, H.; Vreven, T.; Throssell, K.; Montgomery, J. A.; Peralta, Jr., J. E.; Ogliaro, F.; Bearpark, M. J.; Heyd, J. J.; Brothers, E. N.; Kudin, K. N.; Staroverov, V. N. ;Keith, T. A.; Kobayashi, R.; Normand, J.; Raghavachari, K.; Rendell, A. P.; Burant, J. C.; Iyengar, S. S.; Tomasi, J.; Cossi, M.; Millam, J. M.; Klene, M.; Adamo, C.; Cammi, R.; Ochterski, J. W.; Martin, R. L.; Morokuma, K.; Farkas, O.; Foresman, J. B.; Fox, D. J. Gaussian, Inc., Wallingford CT, **2016**.
8. Becke, A. D. Density-functional thermochemistry. III. The role of exact exchange. *J. Chem. Phys.* **98**, 5648−5652 (1993).
9. Lee, C., Yang, W. & Parr, R. G. Development of the Colle-Salvetti correlation-energy formula into a functional of the electron density. *Phys. Rev. B.* **37**, 785−789 (1988).
10. Ditchfield, R., Hehre, W. J. & Pople, J. A. Self-Consistent Molecular-Orbital Methods. IX. An Extended Gaussian-Type Basis for Molecular-Orbital Studies of Organic Molecules. *J. Chem. Phys.* **54**, 724−728 (1971).
11. Hariharan, P. C. & Pople, J. A. The influence of polarization functions on molecular orbital hydrogenation energies. *Theor. Chim. Acta*. **28**, 213−222 (1973).

12. Hehre, W. J., Ditchfield, R. & Pople, J. A. Self—Consistent Molecular Orbital Methods. XII. Further Extensions of Gaussian—Type Basis Sets for Use in Molecular Orbital Studies of Organic Molecules. *J. Chem. Phys.* **56**, 2257−2261 (1972).
13. Roy, L. E., Hay, P. J. & Martin, R. L. Revised Basis Sets for the LANL Effective Core Potentials. *J. Chem. Theory Comput.* **4**, 1029–103 (2008).
14. Pritchard, B. P., Altarawy, D., Didier, B., Gibson, T. D. & Windus. T. L. New Basis Set Exchange: An Open, Up-to-Date Resource for the Molecular Sciences Community. *J. Chem. Inf. Model.* **59**, 4814-4820 (2019).
15. Chai J.-D. & Head-Gordon, M. Long-range corrected hybrid density functionals with damped atom-atom dispersion corrections. *Phys. Chem. Chem. Phys*. **10**, 6615-6620 (2008).
16. Weigend, F. & Ahlrichs, R. Balanced basis sets of split valence, triple zeta valence and quadruple zeta valence quality for H to Rn: Design and assessment of accuracy. *Phys. Chem. Chem. Phys.* **7**, 3297−3305 (2005).
17. Marenich, A. V., Cramer, C. J. & Truhlar, D. G. Universal solvation model based on solute electron density and on a continuum model of the solvent defined by the bulk dielectric constant and atomic surface tensions. *J. Phys. Chem. B.* **113**, 6378-6396 (2009).
18. Stephenson, T. A., Morehouse, S. M., Powell, A. R., Heffer, J. P. & Wilkinson, G. Carboxylates of palladium, platinum, and rhodium, and their adducts. *J. Chem. Soc.*, **1965**, 3632-3640.
19. Bakhmutov, V. I., Berry, J. F., Cotton, F. A., Ibragimovb, S. & Murillo, C. A. Non-trivial behavior of Palladium(II) acetate. *Dalton Trans.*, **2005**, 1989-1992.
