# Supplementary material for: Expanding chemical space by para-C−H arylation of arenes
Source: Nat Commun. 2022 Jul 8;13:3963. doi: 10.1038/s41467-022-31506-x (PMC9270437; doi:10.1038/s41467-022-31506-x)
Supplement: Supplementary file 3 — Description of Additional Supplementary Files [file 41467_2022_31506_MOESM3_ESM.docx]

**Description of Additional Supplementary Files**

File name: Supplementary Data 1

Description: Computed stationary points and Cartesian coordinates
