# Supplementary material for: Expanding chemical space by para-C−H arylation of arenes
Source: Nat Commun. 2022 Jul 8;13:3963. doi: 10.1038/s41467-022-31506-x (PMC9270437; doi:10.1038/s41467-022-31506-x)
Supplement: Supplementary file 4 — Supplementary Data 1 [file 41467_2022_31506_MOESM4_ESM.docx]

**Supplementary Data**

**Expanding chemical space by *para*-C-H arylation of arenes**

Sudip Maiti, Yingzi Li, Sheuli Sasmal, Srimanta Guin, Trisha Bhattacharya, Goutam Kumar Lahiri, Robert S. Paton & Debabrata Maiti

Department of Chemistry, Indian Institute of Technology Bombay, Mumbai, India. dmaiti@iitb.ac.in

Department of Chemistry, Colorado State University, Fort Collins, Colorado 80523, USA. robert.paton@colostate.edu

Department of Chemistry, Indian Institute of Technology Bombay, Mumbai, India. lahiri@chem.iitb.ac.in

**Computed stationary points**

Thermochemical analysis was performed with GoodVibes 3.0.1, applying a quasi-harmonic correction to the vibrational entropy with a cutoff of 100 cm^-1^.^1,2^ Absolute values (in Hartrees) of SCF energy, zero-point vibrational energy (ZPE), enthalpy and quasi-harmonic Gibbs free energy (at 298K) for optimized structures are given below.

| **Structure** | **E_SPC** | **E** | **ZPE** | **H_SPC** | **T.qh-S** | **G(T)_SPC** | **qh-G(T)_SPC** | **im freq** |
| --- | --- | --- | --- | --- | --- | --- | --- | --- |
| **1a** | -1656.91095 | -1656.78452 | 0.54969 | -1656.32572 | 0.09632 | -1656.43011 | -1656.42203 |  |
| **1b** | -642.77092 | -356.34480 | 0.09964 | -642.66246 | 0.04317 | -642.70596 | -642.70562 |  |
| **1c** | -2001.30786 | -2001.15912 | 0.64019 | -2000.62549 | 0.10974 | -2000.74560 | -2000.73523 |  |
| **AcOH** | -229.12253 | -229.07761 | 0.06206 | -229.05498 | 0.03233 | -229.08760 | -229.08731 |  |
| **AgI** | -444.89534 | -157.21122 | 0.00043 | -444.89103 | 0.03013 | -444.92115 | -444.92115 |  |
| **AgOAc** | -375.57499 | -374.26121 | 0.05053 | -375.51731 | 0.03897 | -375.55752 | -375.55628 |  |
| **HFIP** | -789.94908 | -789.77609 | 0.06243 | -789.87653 | 0.04442 | -789.92183 | -789.92094 |  |
| **Ligand** | -512.39288 | -512.31492 | 0.12308 | -512.25889 | 0.04704 | -512.30836 | -512.30593 |  |
| **int3b** | -2012.80689 | -2011.44639 | 0.59172 | -2012.17349 | 0.10832 | -2012.29081 | -2012.28181 |  |
| **int3a** | -2296.06919 | -2294.65303 | 0.65291 | -2295.36978 | 0.11776 | -2295.49779 | -2295.48753 |  |
| **int1** | -584.99971 | -583.74079 | 0.10358 | -584.88620 | 0.04466 | -584.93161 | -584.93086 |  |
| **int2** | -2241.93185 | -2240.54460 | 0.65476 | -2241.22883 | 0.12287 | -2241.36594 | -2241.35169 |  |
| **int2a** | -2296.04914 | -2294.65166 | 0.65166 | -2295.34994 | 0.12148 | -2295.48475 | -2295.47142 |  |
| **int4a** | -2655.58748 | -2367.78071 | 0.69176 | -2654.84382 | 0.13004 | -2654.98786 | -2654.97386 |  |
| **int4b** | -2938.86093 | -2651.01856 | 0.75341 | -2938.05032 | 0.14073 | -2938.20690 | -2938.19105 |  |
| **int5a** | -2655.55638 | -2367.76668 | 0.69199 | -2654.81272 | 0.12852 | -2654.95330 | -2654.94123 |  |
| **int5b** | -2938.83067 | -2650.99704 | 0.75312 | -2938.02035 | 0.14024 | -2938.17554 | -2938.16059 |  |
| **int6a** | -2655.62986 | -2367.83398 | 0.69399 | -2654.88435 | 0.12853 | -2655.02631 | -2655.01288 |  |
| **int6b** | -2938.90379 | -2651.06853 | 0.75568 | -2938.09140 | 0.13913 | -2938.24602 | -2938.23053 |  |
| **ts-1a-m** | -2241.89647 | -2240.50146 | 0.64993 | -2241.19929 | 0.11958 | -2241.33026 | -2241.31887 | -981.41 |
| **ts-1a-o** | -2241.89702 | -2240.49495 | 0.64964 | -2241.20010 | 0.11946 | -2241.33124 | -2241.31956 | -1144.72 |
| **ts-1a-p** | -2241.90561 | -2240.50926 | 0.65000 | -2241.20839 | 0.11950 | -2241.33930 | -2241.32789 | -1131.84 |
| **ts-1b-m** | -2296.03339 | -2294.62884 | 0.64730 | -2295.33984 | 0.11756 | -2295.46767 | -2295.45739 | -1146.72 |
| **ts-1b-o** | -2296.03803 | -2294.62451 | 0.64731 | -2295.34465 | 0.11660 | -2295.47087 | -2295.46124 | -1236.49 |
| **ts-1b-p** | -2296.04079 | -2294.63428 | 0.64754 | -2295.34710 | 0.11727 | -2295.47438 | -2295.46436 | -1191.62 |
| **ts-2b** | -2655.54461 | -2367.75088 | 0.69082 | -2654.80241 | 0.12823 | -2654.94312 | -2654.93064 | -154.41 |
| **ts-2a** | -2938.81865 | -2650.98225 | 0.75198 | -2938.00974 | 0.14031 | -2938.16564 | -2938.15005 | -152.79 |
| **ts-2a-m** | -2938.81513 | -2650.98000 | 0.75209 | -2938.00618 | 0.13982 | -2938.16114 | -2938.14600 | -152.79 |
| **ts-2a-p** | -2938.81382 | -2650.97453 | 0.75204 | -2938.00502 | 0.13910 | -2938.15872 | -2938.14412 | -146.15 |
| **ts-3b** | -2655.54778 | -2367.75388 | 0.69153 | -2654.80516 | 0.12739 | -2654.94479 | -2654.93255 | -145.77 |
| **ts-3a** | -2938.82035 | -2650.98299 | 0.75248 | -2938.01115 | 0.13967 | -2938.16643 | -2938.15082 | -157.33 |
| **ts-1a-m1** | -2796.27039 | -2794.84404 | 0.81653 | -2795.39853 | 0.13617 | -2795.54945 | -2795.53470 | -1154.06 |
| **ts-1a-o1** | -2796.27527 | -2794.83955 | 0.81641 | -2795.40364 | 0.13533 | -2795.55315 | -2795.53896 | -1242.54 |
| **ts-1a-p1** | -2796.27777 | -2794.84925 | 0.81665 | -2795.40583 | 0.13596 | -2795.55634 | -2795.54179 | -1192.53 |
| **ts-1aa-m** | -3086.00080 | -3084.44225 | 0.71170 | -3085.23226 | 0.13837 | -3085.38511 | -3085.37064 | -1087.37 |
| **ts-1aa-o** | -3086.00282 | -3084.43355 | 0.71153 | -3085.23440 | 0.13870 | -3085.38822 | -3085.37310 | -1226.32 |
| **ts-1aa-p** | -3086.00647 | -3084.44639 | 0.71183 | -3085.23776 | 0.13896 | -3085.39173 | -3085.37672 | -1181.14 |
| **int10** | -3031.18549 | -2742.10643 | 0.74389 | -3030.38177 | 0.14581 | -3030.54473 | -3030.52759 |  |
| **int7** | -2617.55508 | -2614.86431 | 0.70719 | -2616.79231 | 0.13664 | -2616.94279 | -2616.92895 |  |
| **int8** | -2900.79488 | -2898.05993 | 0.76689 | -2899.96621 | 0.15086 | -2900.13721 | -2900.11706 |  |
| **int9** | -2388.39688 | -2385.74116 | 0.64225 | -2387.70411 | 0.12789 | -2387.84650 | -2387.83200 |  |
| **ts4-m** | -2617.52770 | -2614.83638 | 0.70192 | -2616.77077 | 0.13554 | -2616.92057 | -2616.90632 | -1147.64 |
| **ts4-o** | -2617.52220 | -2614.82741 | 0.70221 | -2616.76515 | 0.13466 | -2616.91330 | -2616.89981 | -1165.38 |
| **ts4** | -2617.52663 | -2614.83932 | 0.70158 | -2616.76977 | 0.13665 | -2616.92203 | -2616.90642 | -1080.45 |
| **ts4a** | -2900.79049 | -2898.05371 | 0.76274 | -2899.96745 | 0.14610 | -2900.12988 | -2900.11355 | -1167.33 |
| **ts4b** | -2671.64524 | -2668.95261 | 0.69895 | -2670.89203 | 0.13442 | -2671.04088 | -2671.02645 | -1286.87 |
| **ts4c** | -3461.61501 | -3458.75879 | 0.76261 | -3460.78721 | 0.15658 | -3460.96364 | -3460.94379 | -1230.28 |
| **ts5** | -3031.15705 | -2742.07504 | 0.74289 | -3030.35469 | 0.14538 | -3030.51682 | -3030.50007 | -171.76 |

**Cartesian coordinates**

B3LYP-optimized coordinates of stationary points in XYZ format follow:

66

1a Eopt -1656.784517

C 4.993104 -3.444256 -0.113778

C 4.936229 -2.252842 -2.345486

C 5.561482 -0.225514 2.074094

C 3.415923 -1.450981 2.664596

C -7.856568 1.660195 0.028999

C -5.979854 -3.405317 0.565097

C 2.190218 4.425264 0.343344

C 1.471883 3.584780 -0.510450

C 3.502629 4.096042 0.687775

C 2.066844 2.425055 -1.010613

C 4.088690 2.930326 0.190565

C -1.254448 -1.685667 -0.912315

C -0.766881 0.125007 0.593958

C 0.100475 -1.986051 -0.998281

C 0.591953 -0.171790 0.520585

C -5.061721 1.254612 -0.134223

C -4.093886 -1.336207 0.145624

C -2.822429 2.126384 -0.490031

C 4.008137 0.796359 -1.183861

C 5.079941 -2.076531 -0.819692

C 4.071983 -0.424292 1.720332

C 3.379309 2.069562 -0.662251

C -1.717063 -0.615340 -0.125942

C -3.671663 1.007701 -0.207910

C 1.039900 -1.221850 -0.291334

C -3.165290 -0.298552 -0.060093

C -5.961411 0.220644 0.074212

C -5.464882 -1.104572 0.212608

N -2.177458 3.065963 -0.729357

O -7.310276 0.356869 0.159730

O -6.405090 -2.058294 0.417870

O 2.350420 -1.557929 -0.429144

Si 3.841439 -0.800422 -0.137166

H 5.214206 -3.374068 0.957052

H 3.993021 -3.880378 -0.219409

H 5.710700 -4.152169 -0.550551

H 5.089084 -1.314160 -2.890092

H 5.673220 -2.974552 -2.722881

H 3.941257 -2.632291 -2.606761

H 6.130864 -1.156023 1.959269

H 6.042622 0.535936 1.447993

H 5.671688 0.096008 3.118188

H 2.345038 -1.565943 2.467380

H 3.875224 -2.441859 2.571776

H 3.529926 -1.137200 3.711157

H -7.631077 2.094638 -0.953935

H -8.936284 1.541324 0.132759

H -7.486690 2.332330 0.814625

H -6.890789 -3.988612 0.708085

H -5.455529 -3.761184 -0.331168

H -5.327458 -3.527519 1.439286

H 1.731585 5.330088 0.733119

H 0.445456 3.818381 -0.780269

H 4.075383 4.747824 1.343120

H 1.497664 1.780438 -1.675592

H 5.115790 2.690843 0.459183

H -1.965101 -2.269018 -1.491947

H -1.088825 0.944205 1.228799

H 0.455093 -2.799526 -1.623600

H 1.301451 0.426884 1.080792

H -5.411288 2.271906 -0.258155

H -3.711454 -2.340517 0.281228

H 5.088728 0.946471 -1.324342

H 3.608355 0.559310 -2.178441

H 6.080798 -1.658753 -0.624818

H 3.573342 0.543165 1.883154

14

1b Eopt -356.344795

C -0.498451 1.312359 -0.000013

C -1.891371 1.378940 0.000009

C -2.660478 0.209289 -0.000001

C -2.022692 -1.039888 0.000012

C -0.634147 -1.120599 0.000034

C 0.117713 0.059678 0.000002

H 0.093987 2.220595 -0.000035

H -2.384050 2.349031 0.000004

H -2.633878 -1.937394 0.000051

H -0.141998 -2.087030 0.000026

I 2.246168 -0.057868 -0.000003

C -4.138995 0.292023 0.000036

O -4.883073 -0.669574 -0.000059

H -4.545887 1.327591 0.000125

78

1c Eopt -2001.159119

C -6.207049 -2.209593 1.640182

C -5.771442 0.193290 2.292009

C -6.199189 -1.694276 -2.248787

C -4.408219 -3.296486 -1.415573

C 7.240324 -2.609394 -1.160025

C 4.000348 -5.466864 2.127858

C -0.723089 3.315572 -0.145360

C -1.683449 3.180062 0.872279

C -1.002421 2.718212 -1.385649

C -2.867614 2.480932 0.658911

C -2.194043 2.028756 -1.597682

C 0.278565 -1.900164 1.437340

C 0.024285 -1.637525 -0.938781

C -1.070667 -1.620120 1.619260

C -1.328359 -1.353645 -0.767021

C 4.521516 -1.847493 -0.981535

C 2.857015 -3.306893 0.698531

C 2.714282 -0.345986 -1.585228

C -4.443372 1.141856 -0.818878

C -5.973980 -0.779836 1.112591

C -4.776691 -1.820811 -1.661622

C -3.152110 1.891628 -0.582578

C 0.858697 -1.912072 0.155670

C 3.157097 -1.497284 -0.858999

C -1.889173 -1.340960 0.516248

C 2.298095 -2.229704 -0.015597

C 5.053080 -2.912416 -0.271177

C 4.200130 -3.655802 0.590297

N 2.403470 0.598091 -2.192428

O 6.345835 -3.324259 -0.320435

O 4.790608 -4.684499 1.244299

O -3.201793 -1.082598 0.764090

Si -4.557752 -0.662577 -0.155384

H -6.489557 -2.905870 0.843318

H -5.306778 -2.604402 2.125449

H -7.014299 -2.220329 2.384942

H -5.695577 1.236713 1.964498

H -6.615346 0.132747 2.992145

H -4.859921 -0.048403 2.850607

H -6.955598 -2.084670 -1.557116

H -6.467064 -0.655434 -2.479666

H -6.286358 -2.267051 -3.181366

H -3.379039 -3.407538 -1.059089

H -5.066997 -3.762507 -0.674489

H -4.503294 -3.876275 -2.343726

H 7.327689 -1.559046 -0.852670

H 8.208099 -3.100894 -1.048966

H 6.927329 -2.654220 -2.211529

H 4.676456 -6.214884 2.544675

H 3.584433 -4.857423 2.940475

H 3.182401 -5.970239 1.596521

H -1.488940 3.605767 1.852819

H -0.291301 2.813495 -2.200632

H -3.582870 2.389118 1.472742

H -2.389396 1.600441 -2.578660

H 0.902045 -2.086550 2.307633

H 0.429697 -1.655818 -1.945049

H -1.509192 -1.599146 2.611987

H -1.943613 -1.140183 -1.634193

H 5.148658 -1.253781 -1.634663

H 2.200905 -3.892574 1.330561

H -4.652573 1.103836 -1.896064

H -5.277994 1.697041 -0.370074

H -6.882573 -0.465905 0.573223

H -4.082529 -1.433371 -2.424644

C 0.534527 4.068007 0.080095

C 1.741682 3.656658 -0.523717

C 0.551799 5.211705 0.900752

C 2.917970 4.362238 -0.309917

H 1.761896 2.761837 -1.138907

C 1.731360 5.916559 1.115148

H -0.372853 5.562264 1.349887

C 2.926488 5.499920 0.513075

H 3.850021 4.042293 -0.765860

H 1.728667 6.803497 1.746057

C 4.173492 6.253005 0.748754

O 5.261996 5.970212 0.281240

H 4.057126 7.139598 1.413405

8

AcOH Eopt -229.077608

C -0.092358 0.125629 0.000488

O -0.778506 -1.046682 -0.000040

O -0.645714 1.201951 -0.000165

C 1.396912 -0.109479 -0.000076

H -1.723486 -0.802998 -0.000284

H 1.686199 -0.691543 0.881566

H 1.685478 -0.692498 -0.881323

H 1.918252 0.847995 -0.000790

12

HFIP Eopt -789.776092

C -0.004203 0.551029 -0.502998

H -0.001031 0.495274 -1.601508

C 1.267510 -0.166283 -0.026029

C -1.291793 -0.132724 -0.031699

O -0.042367 1.866495 -0.020684

H 0.771189 2.312860 -0.304681

F 1.295950 -0.358212 1.297812

F 1.424612 -1.352131 -0.635002

F 2.331604 0.612636 -0.353808

F -1.294192 -1.425582 -0.413449

F -1.431536 -0.084620 1.298591

F -2.355361 0.468774 -0.590143

16

Ligand Eopt -512.314924

N 0.074857 0.243208 -0.047408

C 1.344113 -0.243050 -0.010083

O 2.241200 0.778498 -0.009541

C 3.612890 0.365747 0.023950

H 3.823360 -0.212695 0.927798

H 4.193664 1.289141 0.019277

H 3.857459 -0.243168 -0.850825

O 1.635136 -1.426556 0.016650

H -0.102086 1.238856 -0.039600

C -1.057805 -0.645399 -0.027182

C -2.330050 0.173928 0.005294

O -2.378992 1.384526 0.017078

O -3.421686 -0.617256 0.018957

H -4.195586 -0.023198 0.039610

H -1.081337 -1.301599 -0.907772

H -1.039617 -1.310850 0.846347

15

int1 Eopt -583.740787

C 2.439174 0.001979 -0.001690

O 1.766280 -1.086657 -0.002211

O 1.768055 1.090312 -0.002282

C -2.439161 0.002024 0.000908

O -1.768010 1.090341 0.000463

O -1.766313 -1.086636 0.000263

C 3.936156 -0.004338 0.004415

C -3.936163 -0.004287 0.002449

Pd -0.000001 0.002008 -0.000901

H 4.319716 1.016078 -0.050371

H 4.294573 -0.486795 0.920071

H 4.303896 -0.590802 -0.843673

H -4.319515 1.017657 -0.001243

H -4.298390 -0.536059 0.888511

H -4.300351 -0.543592 -0.878190

81

int2b Eopt -2240.544602

C -7.284269 2.134422 -0.633589

C -6.938195 0.288003 -2.325150

C -6.523943 0.040597 2.601989

C -4.952983 2.004868 2.241938

C 6.221197 3.529422 0.203319

C 2.045347 6.962465 -0.371182

C -1.321432 -3.428295 1.148920

C -1.464380 -3.040889 -0.186075

C -2.398533 -3.282975 2.025925

C -2.673653 -2.505180 -0.632237

C -3.606825 -2.748826 1.573062

C -1.056525 2.873396 -1.371692

C -0.684828 1.307764 0.416655

C -2.368238 2.430480 -1.491028

C -1.996612 0.852413 0.302875

C 3.636966 2.444032 -0.022470

C 1.479183 4.189769 -0.321937

C 2.213499 0.531661 -0.184683

C -5.058116 -1.737564 -0.253189

C -6.935315 0.644685 -0.823970

C -5.243714 0.531990 1.891790

C -3.763785 -2.343699 0.237198

C -0.183148 2.319969 -0.417670

C 2.316011 1.949252 -0.152190

C -2.852362 1.413613 -0.654383

C 1.209975 2.809817 -0.295360

C 3.874482 3.807769 -0.046911

C 2.772726 4.696778 -0.205151

N 2.296753 -0.623866 -0.201434

O 5.093265 4.388653 0.067392

O 3.089401 6.011216 -0.214485

O -4.141183 1.022556 -0.836341

Si -5.304741 0.139036 0.023985

H -7.426654 2.397297 0.420155

H -6.495256 2.781088 -1.035532

H -8.214584 2.384141 -1.161511

H -6.800681 -0.784742 -2.500807

H -7.892300 0.574676 -2.787440

H -6.140756 0.818531 -2.858410

H -7.407551 0.603892 2.277583

H -6.724782 -1.022248 2.418003

H -6.437717 0.171598 3.688735

H -4.011829 2.352706 1.804539

H -5.748221 2.673083 1.893195

H -4.878454 2.130569 3.330680

H 6.332910 2.875378 -0.669994

H 7.085832 4.190069 0.282084

H 6.146423 2.908627 1.104517

H 2.532449 7.938556 -0.361536

H 1.518978 6.826021 -1.324424

H 1.324363 6.905972 0.454221

H -0.379820 -3.842994 1.499139

H -0.633589 -3.156129 -0.876842

H -2.302659 -3.591510 3.064057

H -2.774778 -2.204197 -1.672677

H -4.443863 -2.657045 2.262402

H -0.692789 3.642889 -2.047326

H -0.051022 0.876630 1.185707

H -3.035023 2.851655 -2.236960

H -2.345637 0.055841 0.950543

H 4.441466 1.723858 0.084301

H 0.641586 4.871027 -0.406495

H -5.915414 -2.234291 0.224660

H -5.165866 -1.918035 -1.329733

H -7.724623 0.049036 -0.337666

H -4.408334 -0.067110 2.284072

Pd 2.880434 -2.531547 -0.214194

C 2.323242 -4.593969 -1.420288

O 1.697028 -3.503824 -1.663689

C 5.063787 -1.242222 1.233081

O 4.164677 -2.195966 1.278548

O 5.201384 -0.403901 0.345071

C 5.976147 -1.283575 2.457479

H 6.693282 -0.461607 2.411439

H 5.383472 -1.212095 3.375171

H 6.511367 -2.238161 2.489378

O 3.245522 -4.559644 -0.536691

C 1.964941 -5.867591 -2.125908

H 2.778370 -6.591036 -2.038641

H 1.062778 -6.290477 -1.667894

H 1.743428 -5.662742 -3.176967

81

int2a Eopt -2294.651655

C -7.601475 0.707292 -0.787852

C -6.872822 -1.111478 -2.384154

C -6.489964 -0.994029 2.578735

C -5.336611 1.209250 2.068240

C 4.883119 5.562553 0.483973

C -0.107849 7.533257 -0.353969

C -0.639245 -3.463830 1.058736

C -0.954971 -3.223948 -0.281091

C -1.657008 -3.459677 2.014778

C -2.275751 -2.976642 -0.656284

C -2.979002 -3.215599 1.634029

C -1.775924 2.734677 -1.411967

C -1.044818 1.329628 0.399543

C -2.914174 1.952895 -1.563857

C -2.179104 0.533696 0.251370

C 2.766916 3.719891 0.133454

C 0.203099 4.719142 -0.287216

C 1.992824 1.450270 -0.051054

C -4.732836 -2.675044 -0.126372

C -6.972958 -0.695861 -0.901485

C -5.327065 -0.313049 1.824056

C -3.310725 -2.966007 0.292349

C -0.816591 2.444340 -0.424262

C 1.672873 2.836603 -0.043890

C -3.132639 0.848320 -0.726832

C 0.365345 3.321704 -0.249995

C 2.582360 5.091366 0.096671

C 1.269778 5.599760 -0.124002

N 2.367470 0.351868 -0.044106

O 3.555548 6.020120 0.254068

O 1.169868 6.946665 -0.140673

O -4.266826 0.129302 -0.930790

Si -5.296135 -0.852918 -0.007459

H -7.818526 0.983411 0.249493

H -6.939287 1.474727 -1.206605

H -8.547939 0.753245 -1.343394

H -6.526880 -2.143739 -2.507895

H -7.853565 -1.035325 -2.872488

H -6.180192 -0.460538 -2.930095

H -7.465387 -0.644424 2.218367

H -6.473408 -2.086328 2.479428

H -6.440637 -0.764669 3.651350

H -4.481878 1.706946 1.599872

H -6.247933 1.679730 1.682817

H -5.293338 1.422033 3.145079

H 5.252656 4.965795 -0.359601

H 5.491448 6.462052 0.586809

H 4.948118 4.969369 1.404927

H 0.057427 8.611311 -0.337957

H -0.524357 7.241183 -1.326173

H -0.811525 7.259265 0.442046

H 0.391359 -3.658602 1.340056

H -0.163048 -3.236946 -1.023326

H -1.426286 -3.655599 3.059128

H -2.512708 -2.790639 -1.701665

H -3.766031 -3.234765 2.385483

H -1.615737 3.573474 -2.083978

H -0.340973 1.086769 1.189833

H -3.648291 2.177274 -2.331388

H -2.317766 -0.330756 0.891544

H 3.752353 3.297661 0.286299

H -0.798093 5.110466 -0.417145

H -5.437795 -3.264153 0.477806

H -4.883819 -2.993839 -1.164900

H -7.647374 -1.408560 -0.399729

H -4.391884 -0.705346 2.251373

Pd 3.332647 -1.457748 0.067168

N 4.291650 -3.152941 0.181478

C 5.737601 -3.040622 0.176209

H 6.193688 -3.702313 0.921128

H 6.175104 -3.288867 -0.800635

C 6.118964 -1.578505 0.522539

O 5.125157 -0.693731 0.551382

O 7.272622 -1.265380 0.737565

C 3.478218 -3.907343 -0.554996

O 2.308557 -3.471935 -0.757703

O 3.923721 -5.076696 -1.025230

C 2.975378 -5.855984 -1.776465

H 2.670976 -5.323009 -2.680791

H 3.500417 -6.776114 -2.034002

H 2.092527 -6.074356 -1.170568

81

Int3a Eopt -2294.673821

C -5.856379 -5.142578 0.178737

C -4.853401 -4.973229 -2.135332

C -2.547454 -5.670626 2.196279

C -3.822359 -3.564050 2.823213

C -0.363553 7.624377 0.260049

C -5.541693 6.000214 0.271694

C 1.149270 -0.886511 -0.578350

C 0.224671 -0.897083 -1.626969

C 1.223388 -1.988445 0.279608

C -0.635830 -1.984714 -1.791867

C 0.346939 -3.066035 0.113804

C -3.908312 1.137224 -0.858453

C -2.274631 0.630934 0.834456

C -4.239873 -0.209952 -0.945493

C -2.601706 -0.719131 0.756991

C -0.882681 4.846445 0.118808

C -3.538151 4.002691 0.137262

C -0.107631 2.561137 -0.091888

C -1.626575 -4.189444 -1.038522

C -4.548969 -5.184900 -0.637861

C -2.803834 -4.188156 1.848659

C -0.612842 -3.074568 -0.909917

C -2.905030 1.585398 0.019804

C -1.196009 3.466198 0.054658

C -3.574076 -1.156835 -0.152390

C -2.534955 3.019222 0.074547

C -1.886949 5.796802 0.188617

C -3.244637 5.363352 0.191011

N 0.816820 1.870382 -0.223653

O -1.698794 7.137495 0.254421

O -4.162910 6.350527 0.263176

O -3.930076 -2.458554 -0.304064

Si -3.221657 -3.973835 -0.003321

H -5.701859 -5.423784 1.225877

H -6.301452 -4.140643 0.163786

H -6.596499 -5.837591 -0.240485

H -3.964593 -5.092831 -2.764905

H -5.600009 -5.699582 -2.483243

H -5.258400 -3.970901 -2.316908

H -3.459450 -6.271957 2.096784

H -1.780597 -6.124413 1.556414

H -2.204804 -5.771459 3.234491

H -3.990332 -2.501064 2.620858

H -4.795154 -4.065324 2.770718

H -3.467330 -3.653383 3.858828

H 0.166223 7.352570 -0.662189

H -0.444902 8.710239 0.324682

H 0.196484 7.247813 1.125827

H -6.084280 6.944973 0.323348

H -5.823423 5.465608 -0.644027

H -5.792242 5.385931 1.145474

H 0.156940 -0.058129 -2.313364

H 1.964859 -2.019294 1.073333

H -1.355379 -1.972586 -2.608295

H 0.419448 -3.915830 0.790584

H -4.407921 1.849852 -1.509531

H -1.521959 0.944072 1.551586

H -4.997090 -0.555080 -1.642531

H -2.076775 -1.432218 1.381558

H 0.158287 5.143966 0.098680

H -4.569275 3.673598 0.174034

H -1.182844 -5.150096 -0.739408

H -1.922282 -4.304704 -2.088434

H -4.113443 -6.190850 -0.524540

H -1.848768 -3.657881 1.986939

Pd 2.388487 0.631301 -0.277150

C 4.903406 0.578229 -0.011547

O 4.398640 1.726388 0.062418

O 4.168983 -0.460583 -0.201977

C 6.405859 0.396773 0.118816

H 6.885404 0.991651 -0.669908

H 6.718805 0.839466 1.072690

N 6.764679 -1.003036 0.034592

C 8.022170 -1.510547 0.131602

O 8.315237 -2.690304 0.045914

O 8.928786 -0.511586 0.343734

C 10.282619 -0.958075 0.462794

H 10.874560 -0.055520 0.624385

H 10.394356 -1.643647 1.307643

H 10.606470 -1.468504 -0.448646

H 6.017845 -1.661799 -0.139189

81

Int3b Eopt -2294.653028

C -7.503994 1.099401 -0.128113

C -6.635749 -0.000759 -2.230529

C -6.011391 -1.336333 2.502158

C -5.123774 1.037405 2.693520

C 5.919804 4.622799 0.221614

C 1.495652 7.718295 -0.342463

C 0.233230 -1.976861 -0.208603

C -0.389699 -1.515265 -1.384682

C -0.615550 -2.432965 0.819950

C -1.780348 -1.486574 -1.515169

C -2.007553 -2.404094 0.689133

C -1.322562 3.320940 -1.134809

C -0.793167 1.935487 0.759331

C -2.577183 2.726934 -1.222219

C -2.045386 1.335216 0.680253

C 3.407210 3.333980 0.093727

C 1.127065 4.915567 -0.182416

C 2.069882 1.315830 0.035841

C -4.121969 -1.825075 -0.610561

C -6.692598 -0.085182 -0.691056

C -4.964583 -0.324168 1.988176

C -2.618887 -1.914107 -0.475567

C -0.395954 2.924704 -0.154053

C 2.124737 2.738648 0.030360

C -2.943480 1.711691 -0.326551

C 0.959015 3.522544 -0.097736

C 3.549895 4.709737 0.017893

C 2.383841 5.515251 -0.130270

N 2.082867 0.156468 0.029796

O 4.725658 5.381087 0.069490

O 2.608718 6.846212 -0.195447

O -4.173496 1.152374 -0.483247

Si -4.956740 -0.244065 0.078296

H -7.682846 1.007498 0.948468

H -6.988227 2.051308 -0.301346

H -8.484946 1.163136 -0.618132

H -6.150271 -0.873432 -2.681792

H -7.648577 0.062136 -2.650302

H -6.087186 0.890746 -2.555497

H -7.034095 -1.005942 2.283488

H -5.884143 -2.332264 2.059942

H -5.934824 -1.452685 3.591221

H -4.357830 1.754909 2.381669

H -6.100601 1.489473 2.489061

H -5.043344 0.917441 3.782368

H 6.070064 3.938334 -0.622938

H 6.730871 5.351750 0.247248

H 5.913413 4.049225 1.157155

H 1.913929 8.724978 -0.381622

H 0.947039 7.513749 -1.270547

H 0.810480 7.640062 0.511042

H 0.218527 -1.158471 -2.212990

H -0.186133 -2.822159 1.741044

H -2.222193 -1.114702 -2.437877

H -2.626829 -2.775611 1.504427

H -1.041433 4.077686 -1.862569

H -0.118551 1.630268 1.553173

H -3.280916 3.018508 -1.995628

H -2.307277 0.555501 1.385258

H 4.272877 2.690376 0.189341

H 0.239656 5.531567 -0.261338

H -4.601856 -2.673311 -0.100783

H -4.401651 -1.909157 -1.667710

H -7.223727 -1.013779 -0.426185

H -3.975386 -0.722168 2.263360

Pd 2.252107 -1.847835 0.054989

C 4.903704 -2.813500 0.685303

O 6.080521 -2.930392 0.998818

O 4.297178 -1.700482 0.392593

C 4.040575 -4.096200 0.648850

H 4.571440 -4.857469 0.071363

H 3.948843 -4.471504 1.675908

N 2.697319 -3.853682 0.088737

C 2.042883 -4.837931 -0.429789

O 0.853430 -4.729700 -1.002339

O 2.550164 -6.071222 -0.418267

C 1.803285 -7.129277 -1.044967

H 2.415606 -8.020753 -0.908863

H 0.831948 -7.256328 -0.561175

H 1.660470 -6.925010 -2.108930

H 0.534718 -3.795953 -0.896833

87

Int4a Eopt -2651.018560

C -6.744414 4.918983 -0.310557

C -6.342002 3.496179 -2.359029

C -6.422943 2.183455 2.423087

C -4.654330 4.001525 2.588765

C 6.973165 2.866346 0.411951

C 4.097274 7.413567 -0.304568

C -0.898846 -0.955652 -0.157542

C -1.276390 -0.345059 -1.358938

C -1.822971 -0.995504 0.892726

C -2.548911 0.214406 -1.502717

C -3.096487 -0.435954 0.741366

C -0.172393 4.430418 -1.151550

C -0.286981 3.025280 0.797228

C -1.556858 4.379626 -1.273554

C -1.672388 2.968108 0.684018

C 4.156738 2.637998 0.229439

C 2.669994 4.977487 -0.125719

C 2.118661 1.309234 0.157018

C -4.828821 0.858707 -0.605687

C -6.466906 3.490609 -0.821332

C -5.044969 2.667624 1.921728

C -3.479264 0.192424 -0.453202

C 0.494702 3.739128 -0.124384

C 2.742852 2.588722 0.140216

C -2.320390 3.630092 -0.366703

C 1.974187 3.760917 -0.030216

C 4.821649 3.852704 0.143009

C 4.059017 5.043410 -0.044370

N 1.631777 0.255445 0.159950

O 6.158442 4.024229 0.217716

O 4.782161 6.182189 -0.117277

O -3.666814 3.602072 -0.556315

Si -4.963807 2.667600 0.012017

H -6.971361 4.941307 0.760579

H -5.884250 5.576424 -0.484063

H -7.603886 5.356889 -0.835921

H -6.233898 2.488403 -2.775783

H -7.234298 3.944104 -2.816352

H -5.474806 4.084208 -2.680718

H -7.219133 2.890947 2.161670

H -6.700295 1.206014 2.009339

H -6.424017 2.084717 3.516527

H -3.656554 4.336534 2.287056

H -5.359326 4.802372 2.339971

H -4.653885 3.898170 3.682295

H 6.866438 2.151162 -0.408529

H 7.998439 3.236672 0.450769

H 6.728364 2.356097 1.349474

H 4.873482 8.179109 -0.341855

H 3.533080 7.418714 -1.245798

H 3.416240 7.623946 0.529740

H -0.576982 -0.287273 -2.189565

H -1.565168 -1.470126 1.836710

H -2.816538 0.691635 -2.443761

H -3.802783 -0.495813 1.567906

H 0.405666 4.985135 -1.886012

H 0.189981 2.509600 1.625060

H -2.066024 4.895955 -2.081388

H -2.241551 2.385344 1.398028

H 4.716150 1.716428 0.346958

H 2.092256 5.886814 -0.237371

H -5.599311 0.288937 -0.066021

H -5.127540 0.848028 -1.661052

H -7.334495 2.863990 -0.558153

H -4.307880 1.913929 2.240298

Pd 0.960513 -1.662643 0.071258

C 3.560654 -3.318223 0.104533

O 3.007673 -2.189697 0.360566

O 3.002453 -4.378296 -0.215529

C 5.098516 -3.315980 0.222988

H 5.519602 -3.649218 -0.730941

H 5.385002 -4.060383 0.976665

I 0.242003 -4.272559 -0.196822

C -1.907779 -4.297128 -0.189925

C -2.575352 -4.797404 0.933742

C -2.616681 -3.865461 -1.311599

C -3.965202 -4.861816 0.934431

H -2.014857 -5.134015 1.800434

C -4.009171 -3.930176 -1.303013

H -2.094911 -3.470853 -2.176374

C -4.689863 -4.426288 -0.184408

H -4.509601 -5.244199 1.792684

H -4.571975 -3.590999 -2.170130

C -6.169108 -4.485616 -0.185273

O -6.846323 -4.886359 0.742063

H -6.646427 -4.124822 -1.123710

N 5.564919 -1.984233 0.569646

C 6.761268 -1.428613 0.272595

O 7.023752 -0.234910 0.385054

O 7.658067 -2.358465 -0.153336

C 8.951658 -1.841824 -0.478277

H 9.539230 -2.705494 -0.793606

H 8.888515 -1.109270 -1.288252

H 9.411456 -1.366379 0.392731

87

Int4b Eopt -2367.780713

C 2.120487 -7.439720 -0.155699

C 2.661181 -6.080511 -2.215775

C 3.420584 -4.983825 2.556948

C 0.922062 -5.428665 2.700128

C -7.739690 2.327139 0.244234

C -8.139419 -3.044875 -0.414892

C 0.807497 0.763188 -0.112474

C 0.760347 0.031933 -1.305906

C 1.567719 0.253515 0.947335

C 1.456447 -1.172935 -1.434837

C 2.265327 -0.951884 0.812728

C -2.913814 -3.217723 -1.142098

C -2.016935 -2.097694 0.789423

C -1.765639 -3.997660 -1.226267

C -0.866059 -2.874722 0.715421

C -5.346631 0.824720 0.126273

C -5.542281 -1.937103 -0.200430

C -2.926763 0.703606 0.091899

C 2.920213 -3.025997 -0.515600

C 2.737643 -6.125508 -0.675545

C 2.024612 -4.583457 2.031939

C 2.214387 -1.694649 -0.376374

C -3.055911 -2.237114 -0.144115

C -4.179339 0.025942 0.064521

C -0.719145 -3.819043 -0.309066

C -4.258887 -1.374212 -0.089038

C -6.601742 0.248443 0.023790

C -6.699276 -1.162512 -0.150178

N -1.936001 1.306916 0.100748

O -7.780217 0.916989 0.070950

O -7.957748 -1.645222 -0.240219

O 0.386386 -4.593934 -0.462810

Si 1.981564 -4.568322 0.120987

H 2.272333 -7.573980 0.920568

H 1.041611 -7.474539 -0.348170

H 2.572401 -8.304669 -0.659830

H 3.173322 -5.207826 -2.636462

H 3.128573 -6.973949 -2.650905

H 1.620025 -6.052431 -2.557300

H 3.658750 -6.027106 2.316558

H 4.220199 -4.357975 2.141826

H 3.465911 -4.885121 3.649485

H -0.080322 -5.125502 2.380358

H 1.031841 -6.494418 2.471145

H 0.968068 -5.325359 3.792713

H -7.215964 2.818288 -0.586071

H -8.780584 2.653158 0.260200

H -7.256408 2.600738 1.191090

H -9.218152 -3.196469 -0.470827

H -7.671791 -3.395665 -1.343423

H -7.734139 -3.609551 0.434051

H 0.170542 0.391657 -2.145960

H 1.636155 0.797697 1.886587

H 1.398307 -1.723700 -2.372191

H 2.866394 -1.312063 1.646309

H -3.696953 -3.343322 -1.885289

H -2.107705 -1.376606 1.595934

H -1.648169 -4.736361 -2.012967

H -0.073142 -2.721092 1.437105

H -5.235854 1.895855 0.240216

H -5.618604 -3.012979 -0.299133

H 3.880809 -3.005995 0.019781

H 3.165933 -3.202803 -1.569883

H 3.802853 -6.118392 -0.393141

H 1.861012 -3.536311 2.330770

Pd -0.261713 2.455215 0.072201

C -1.237673 5.341989 0.309712

O -1.591690 4.106988 0.276835

O -0.075451 5.784704 0.229167

C -2.395465 6.327369 0.461698

H -2.958133 6.099786 1.373591

H -2.024974 7.353281 0.502867

H -3.084964 6.218538 -0.382824

I 1.927667 4.054379 -0.038541

C 3.624385 2.739726 -0.248506

C 4.544294 2.654028 0.803351

C 3.833923 2.035263 -1.435000

C 5.675860 1.856052 0.668558

H 4.377257 3.207675 1.722486

C 4.967192 1.234047 -1.564189

H 3.117358 2.098291 -2.246654

C 5.892248 1.138500 -0.516945

H 6.403587 1.771394 1.470177

H 5.135218 0.676834 -2.483586

C 7.087947 0.280226 -0.664103

O 7.937946 0.120246 0.191657

H 7.167985 -0.236333 -1.647013

95

Int5a Eopt -2650.997038

C 8.676199 0.046016 -1.017079

C 7.962501 -0.942326 1.196809

C 6.384964 -2.048564 -3.373425

C 5.929831 0.449685 -3.445272

C -3.994136 5.449709 0.759283

C 0.804104 7.988741 0.856185

C 0.643807 -1.872878 0.179476

C 1.441781 -1.434377 1.238166

C 1.241504 -2.458354 -0.939977

C 2.828591 -1.579504 1.171234

C 2.635182 -2.588362 -0.995102

C 3.144507 3.186253 1.014504

C 2.165818 2.058028 -0.871961

C 4.286334 2.400920 0.893138

C 3.301780 1.266014 -1.000151

C -1.669928 3.884659 0.472969

C 0.814171 5.179374 0.504127

C -0.582420 1.744411 0.259995

C 4.960913 -2.268364 -0.005626

C 7.767397 -0.995550 -0.332816

C 5.632361 -0.873408 -2.711523

C 3.455581 -2.148897 0.053109

C 2.049600 3.015141 0.149095

C -0.464613 3.156222 0.329085

C 4.366243 1.414150 -0.101337

C 0.797264 3.787340 0.325645

C -1.627839 5.259211 0.634789

C -0.361199 5.915887 0.659687

N -0.751682 0.598243 0.224407

O -2.708126 6.061880 0.780012

O -0.413757 7.254241 0.822909

O 5.495177 0.658960 -0.147753

Si 5.934850 -0.835193 -0.825325

H 8.672277 -0.050450 -2.107948

H 8.365389 1.068034 -0.770106

H 9.715682 -0.071098 -0.682678

H 7.408822 -1.730434 1.719432

H 9.023067 -1.065265 1.453047

H 7.634288 0.021531 1.602505

H 7.471927 -1.913421 -3.318415

H 6.147780 -3.014026 -2.909734

H 6.121753 -2.125965 -4.436326

H 5.374896 1.292785 -3.020901

H 6.994432 0.704668 -3.405689

H 5.654623 0.370361 -4.505469

H -4.107890 4.730878 1.579914

H -4.709251 6.264104 0.882359

H -4.177705 4.938061 -0.193484

H 0.514903 9.029811 1.004810

H 1.443472 7.664143 1.686571

H 1.353960 7.892944 -0.088317

H 1.003076 -0.967010 2.112323

H 0.645348 -2.830785 -1.766288

H 3.433844 -1.227436 2.003792

H 3.080107 -3.064378 -1.866740

H 3.083429 3.915243 1.818341

H 1.359136 1.933051 -1.588047

H 5.120017 2.518139 1.578369

H 3.348415 0.521502 -1.785962

H -2.604471 3.333339 0.463676

H 1.770929 5.686977 0.487520

H 5.246512 -3.179461 -0.550883

H 5.357083 -2.388367 1.009834

H 8.082502 -1.994331 -0.675572

H 4.556441 -1.079487 -2.827034

Pd -1.332615 -1.380374 0.035125

C -3.870941 0.386632 -0.556285

O -3.241510 -0.725076 -0.732721

O -3.674669 1.232868 0.329321

C -5.012435 0.650000 -1.552747

H -5.945801 0.698348 -0.974514

H -4.860969 1.648291 -1.979090

C -1.706287 -1.678166 2.026007

C -1.114084 -2.691877 2.779915

C -2.593327 -0.746599 2.546745

C -1.421592 -2.751695 4.137998

H -0.440915 -3.412297 2.335924

C -2.882186 -0.831900 3.914165

H -3.038325 0.031119 1.928123

C -2.304215 -1.825017 4.712089

H -0.988593 -3.524205 4.766724

H -3.567652 -0.110696 4.355081

I -2.094898 -3.845538 -0.464503

C -2.625155 -1.895180 6.155606

O -2.158681 -2.709373 6.929352

H -3.347508 -1.125135 6.507118

N -5.069686 -0.357321 -2.590419

C -5.923933 -0.372016 -3.645816

O -5.996825 -1.251438 -4.487221

O -6.702544 0.752966 -3.654007

C -7.624357 0.821971 -4.744579

H -8.315782 -0.025338 -4.726518

H -8.168049 1.758904 -4.609421

H -7.097415 0.822658 -5.703256

H -4.485411 -1.172533 -2.459436

87

Int5b Eopt -2367.766684

C 8.203534 0.006476 0.644333

C 6.977977 -0.951510 2.636252

C 6.525110 -2.123070 -2.160277

C 6.122396 0.375796 -2.376139

C -4.500144 5.448692 -0.704717

C 0.134896 7.983335 0.551569

C 0.107935 -1.877556 -0.128210

C 0.624142 -1.423542 1.088183

C 0.965164 -2.483673 -1.051718

C 1.982997 -1.571186 1.371630

C 2.328176 -2.617320 -0.757308

C 2.362762 3.182069 1.266176

C 1.864826 2.045529 -0.795031

C 3.497264 2.393319 1.428185

C 2.994796 1.249640 -0.640799

C -2.176666 3.883462 -0.426371

C 0.227029 5.175252 0.206396

C -1.075302 1.741863 -0.370070

C 4.337990 -2.280789 0.773043

C 7.152507 -1.023986 1.104995

C 5.646892 -0.933276 -1.714755

C 2.865212 -2.160230 0.454136

C 1.508513 3.008597 0.162927

C -0.973573 3.153828 -0.273681

C 3.810944 1.400181 0.487880

C 0.252130 3.782985 0.029146

C -2.173449 5.258024 -0.258089

C -0.950160 5.913064 0.073077

N -1.238667 0.597350 -0.447436

O -3.256677 6.061691 -0.378176

O -1.039005 7.252188 0.219834

O 4.914825 0.641586 0.719078

Si 5.490037 -0.865947 0.188631

H 8.458953 -0.104385 -0.414850

H 7.847897 1.032245 0.797406

H 9.132952 -0.108183 1.218120

H 6.312625 -1.732486 3.021122

H 7.946460 -1.071999 3.139480

H 6.566128 0.017659 2.940305

H 7.568664 -1.993028 -1.848887

H 6.174981 -3.079349 -1.752460

H 6.522992 -2.216701 -3.254093

H 5.489841 1.228575 -2.108730

H 7.149110 0.626196 -2.087722

H 6.107091 0.280500 -3.470182

H -4.806269 4.730306 0.065532

H -5.224381 6.262769 -0.756777

H -4.450777 4.935614 -1.673005

H -0.179653 9.024920 0.628127

H 0.551774 7.655294 1.512013

H 0.899047 7.888580 -0.230029

H -0.014900 -0.941003 1.819254

H 0.590927 -2.869741 -1.994183

H 2.364313 -1.205847 2.322873

H 2.973433 -3.109238 -1.482812

H 2.111713 3.915322 2.028180

H 1.254215 1.917747 -1.683769

H 4.141000 2.512297 2.294097

H 3.226383 0.499420 -1.387493

H -3.080949 3.331899 -0.663050

H 1.159844 5.681683 0.422607

H 4.745276 -3.203916 0.335811

H 4.469787 -2.377904 1.857436

H 7.534896 -2.027840 0.859239

H 4.627719 -1.136022 -2.080013

Pd -1.770638 -1.375094 -0.770079

C -4.046761 0.395242 -1.981390

O -3.390856 -0.722060 -1.996025

O -4.068705 1.227452 -1.058992

C -4.871957 0.624596 -3.246702

H -5.622933 -0.167139 -3.341217

H -5.368387 1.596879 -3.209947

H -4.229397 0.565139 -4.131480

C -2.619814 -1.658351 1.070545

C -2.235428 -2.668140 1.953896

C -3.604759 -0.722371 1.352622

C -2.868156 -2.719560 3.194465

H -1.475836 -3.392197 1.693143

C -4.221791 -0.798159 2.607054

H -3.879459 0.051511 0.636525

C -3.861985 -1.787560 3.528579

H -2.605832 -3.488856 3.914861

H -4.992597 -0.073174 2.861723

I -2.400194 -3.846326 -1.421981

C -4.529453 -1.847516 4.847659

O -4.274246 -2.657960 5.718358

H -5.312368 -1.072206 5.005088

95

Int6a Eopt -2651.068532

C 7.701492 1.740570 0.900080

C 6.161324 0.070285 2.013061

C 6.803379 0.837230 -2.802576

C 6.315116 3.264996 -2.220937

C -5.915488 2.199390 1.160689

C -3.216096 6.759466 2.317533

C 1.054431 -1.939502 1.999908

C 1.717727 -0.761214 2.322167

C 1.359242 -2.538573 0.741778

C 2.645269 -0.166413 1.446388

C 2.290561 -1.935942 -0.129647

C 1.362975 3.684654 1.775736

C 1.047525 3.183619 -0.559032

C 2.729120 3.447739 1.644015

C 2.412865 2.957668 -0.703415

C -3.099197 2.210125 0.763067

C -1.703969 4.567893 1.359239

C -0.998574 1.107217 0.204779

C 3.904055 -0.107440 -0.800290

C 6.634394 0.650965 0.665552

C 5.790043 1.816270 -2.172958

C 2.947532 -0.718667 0.208251

C 0.491490 3.493479 0.691394

C -1.696230 2.285412 0.598479

C 3.248553 3.005493 0.419215

C -0.981517 3.467944 0.878485

C -3.802503 3.311930 1.230302

C -3.086781 4.508680 1.539060

N -0.450611 0.128419 -0.093625

O -5.133322 3.364403 1.439458

O -3.847713 5.528780 1.989654

O 4.548018 2.594652 0.378019

Si 5.208842 1.252036 -0.440695

H 8.159772 2.083600 -0.033554

H 7.271926 2.615362 1.401986

H 8.507832 1.356321 1.538815

H 5.457322 -0.758786 1.887360

H 7.017187 -0.304662 2.589786

H 5.669518 0.837526 2.623553

H 7.748365 0.823907 -2.245953

H 6.425646 -0.192044 -2.839392

H 7.038807 1.132448 -3.833263

H 5.592059 3.976236 -1.808328

H 7.246155 3.379473 -1.654348

H 6.526964 3.562292 -3.256513

H -5.567535 1.343508 1.752872

H -6.931389 2.457372 1.462362

H -5.910066 1.949768 0.093884

H -4.015850 7.418987 2.656836

H -2.482079 6.630400 3.123024

H -2.723804 7.202056 1.442410

H 1.505484 -0.269142 3.267131

H 1.034074 -3.555469 0.547479

H 3.124880 0.752996 1.759574

H 2.620941 -2.488549 -1.003807

H 0.957325 3.950565 2.748436

H 0.398654 3.087817 -1.425093

H 3.396836 3.531206 2.496218

H 2.821008 2.702230 -1.676220

H -3.602619 1.281629 0.521368

H -1.166956 5.484561 1.571921

H 3.298000 0.281766 -1.632052

H 4.485919 -0.921270 -1.262341

H 7.108666 -0.167559 0.100249

H 4.881939 1.783295 -2.797191

Pd 0.005693 -1.652879 -1.037663

C -2.670051 -1.107221 -1.892907

O -1.462878 -1.184821 -2.363422

O -3.039846 -1.273743 -0.730556

C -3.691467 -0.774041 -2.997441

H -3.819123 -1.673230 -3.615809

H -3.305343 0.013331 -3.646504

C 0.092016 -2.576255 2.927524

C -1.076013 -3.196584 2.437549

C 0.324761 -2.571957 4.314931

C -1.980327 -3.789044 3.309238

H -1.288764 -3.178867 1.372454

C -0.581553 -3.167289 5.186049

H 1.234821 -2.125403 4.705031

C -1.740354 -3.780464 4.692041

H -2.887210 -4.257904 2.940126

H -0.387816 -3.167591 6.256805

I 0.307800 -3.911379 -2.409024

C -2.699000 -4.409828 5.627007

O -3.732511 -4.959156 5.296572

H -2.406242 -4.347844 6.699540

N -4.933487 -0.363090 -2.371909

C -5.718311 0.682311 -2.741067

O -6.597139 1.173663 -2.044515

O -5.424117 1.114284 -3.996440

C -6.220309 2.213629 -4.452247

H -5.860967 2.434280 -5.458534

H -7.279070 1.942104 -4.476001

H -6.093741 3.083948 -3.802017

H -5.032619 -0.664598 -1.409237

87

Int6b Eopt -2367.833978

C 7.074683 0.347996 2.295350

C 4.951162 -0.752453 3.119473

C 6.857838 -0.832282 -1.422060

C 6.859734 1.699334 -1.175580

C -5.647489 3.552645 -1.159015

C -2.441517 7.691619 0.270562

C -0.313489 -1.656058 1.977696

C 0.507384 -0.595879 2.346230

C 0.124189 -2.476948 0.897660

C 1.718697 -0.329684 1.682539

C 1.338201 -2.202517 0.236137

C 1.357858 3.720884 1.322744

C 1.526530 3.007052 -0.971474

C 2.614957 3.180549 1.584533

C 2.788408 2.476400 -0.722788

C -2.913332 3.012932 -0.742860

C -1.240795 5.143078 -0.009395

C -1.038637 1.477409 -0.594055

C 3.432290 -0.838809 -0.168176

C 5.848251 -0.497172 1.891946

C 5.998367 0.420869 -1.151986

C 2.160739 -1.104901 0.617559

C 0.766033 3.584022 0.056993

C -1.533591 2.810502 -0.515882

C 3.300861 2.482422 0.580422

C -0.675403 3.870570 -0.162925

C -3.453510 4.282337 -0.601200

C -2.604043 5.362928 -0.220136

N -0.686262 0.373699 -0.645052

O -4.755289 4.600941 -0.793260

O -3.218021 6.560525 -0.102214

O 4.426850 1.793821 0.922431

Si 4.910089 0.238929 0.410233

H 7.805235 0.436603 1.484338

H 6.778801 1.361554 2.590332

H 7.589458 -0.107913 3.151568

H 4.106721 -1.410574 2.890857

H 5.530480 -1.221602 3.925726

H 4.545745 0.186993 3.514801

H 7.616439 -0.975322 -0.642879

H 6.258288 -1.749482 -1.473532

H 7.389338 -0.739220 -2.377993

H 6.258521 2.601069 -1.019308

H 7.632888 1.683429 -0.399052

H 7.372170 1.799218 -2.141602

H -5.690017 2.776163 -0.385385

H -6.626684 4.022338 -1.261088

H -5.357278 3.095111 -2.112920

H -3.139891 8.528684 0.306944

H -1.983403 7.554592 1.258230

H -1.658533 7.900754 -0.469170

H 0.203052 0.060884 3.156276

H -0.374298 -3.422978 0.715058

H 2.310272 0.513029 2.019289

H 1.717843 -2.925861 -0.479387

H 0.797568 4.187335 2.128951

H 1.109774 2.939954 -1.972558

H 3.047244 3.225313 2.579636

H 3.352323 2.015865 -1.527821

H -3.518740 2.158247 -1.024276

H -0.591759 5.968731 0.256971

H 3.134633 -0.426716 -1.144024

H 3.890891 -1.807280 -0.424205

H 6.215201 -1.475008 1.540228

H 5.280404 0.501994 -1.984661

Pd -0.558635 -1.587733 -1.253189

C -2.720260 -0.515044 -2.841456

O -1.519119 -1.029598 -2.941280

O -3.337130 -0.276191 -1.805142

C -3.316729 -0.241780 -4.221995

H -3.574602 -1.196231 -4.693746

H -4.219132 0.364854 -4.118869

H -2.592323 0.259713 -4.870348

C -1.577970 -1.952048 2.688828

C -2.705571 -2.423072 1.984616

C -1.678967 -1.768076 4.080018

C -3.893328 -2.694682 2.650708

H -2.654071 -2.542651 0.906287

C -2.869047 -2.041875 4.745731

H -0.810572 -1.437164 4.642209

C -3.985749 -2.506498 4.038550

H -4.768132 -3.046229 2.112514

H -2.933942 -1.905208 5.823340

I -0.590761 -4.059695 -2.242832

C -5.247484 -2.791882 4.755704

O -6.271181 -3.188471 4.231458

H -5.204273 -2.613861 5.854146

81

ts-1a-p Eopt -2294.634279

C -7.691425 0.354584 0.102216

C -6.785395 -0.408704 -2.129590

C -5.743272 -2.129813 2.391981

C -5.225280 0.317898 2.837854

C 5.494188 4.830284 0.126864

C 0.968771 7.791474 -0.346848

C 0.366447 -2.386733 -0.519667

C -0.250537 -1.766802 -1.632341

C -0.497600 -2.802325 0.523643

C -1.629788 -1.599093 -1.710349

C -1.878582 -2.647675 0.445080

C -1.733951 3.266783 -1.072084

C -1.078940 1.925191 0.815805

C -2.953586 2.599682 -1.126263

C -2.293401 1.250295 0.767820

C 3.021937 3.464933 0.035100

C 0.686724 4.978857 -0.185000

C 1.742198 1.409306 -0.002046

C -3.975221 -1.968587 -0.830663

C -6.751800 -0.643693 -0.605065

C -4.887567 -0.914043 1.973966

C -2.475017 -2.053502 -0.682445

C -0.762747 2.927831 -0.114043

C 1.756610 2.833310 -0.002422

C -3.233921 1.567689 -0.219706

C 0.566221 3.581850 -0.098437

C 3.119707 4.845170 -0.038457

C 1.926586 5.615445 -0.160214

N 1.794380 0.250913 -0.013989

O 4.274633 5.551824 -0.008019

O 2.110225 6.952469 -0.227878

O -4.432913 0.932420 -0.333759

Si -4.977299 -0.617221 0.088030

H -7.805927 0.134633 1.168862

H -7.321304 1.382422 0.010115

H -8.693230 0.324165 -0.346775

H -6.214904 -1.162011 -2.684292

H -7.818224 -0.445048 -2.500605

H -6.377224 0.575708 -2.385950

H -6.812839 -1.942574 2.237849

H -5.482883 -3.038222 1.834611

H -5.603904 -2.352139 3.457942

H -4.602763 1.182797 2.586218

H -6.271093 0.621925 2.720604

H -5.069776 0.093994 3.901673

H 5.653793 4.152106 -0.720935

H 6.282267 5.584227 0.142603

H 5.517856 4.255438 1.061108

H 1.356049 8.810156 -0.393324

H 0.404944 7.572382 -1.262437

H 0.306416 7.691548 0.522270

H 0.365184 -1.441095 -2.467739

H -0.073696 -3.309225 1.387962

H -2.068853 -1.140344 -2.593780

H -2.509307 -3.031189 1.244823

H -1.514320 4.034816 -1.809077

H -0.369630 1.669396 1.596681

H -3.693265 2.846247 -1.881534

H -2.499706 0.470143 1.490791

H 3.907670 2.846574 0.112743

H -0.220744 5.567692 -0.241332

H -4.427559 -2.918907 -0.508729

H -4.224593 -1.864597 -1.893296

H -7.134973 -1.659024 -0.414423

H -3.838325 -1.178658 2.177256

Pd 2.360701 -1.697661 0.057161

C 5.045113 -2.000618 1.005993

O 6.156658 -1.776689 1.455202

O 4.178087 -1.058575 0.699297

C 4.584907 -3.451366 0.774047

H 5.328149 -3.954078 0.145925

H 4.585410 -3.966154 1.744212

N 3.260309 -3.485739 0.158750

C 2.808601 -4.545968 -0.475910

O 1.695984 -4.582594 -1.096899

O 3.584968 -5.647566 -0.445416

C 3.095481 -6.795143 -1.153125

H 3.857306 -7.563109 -1.011671

H 2.138693 -7.126715 -0.741435

H 2.971259 -6.572935 -2.216278

H 1.127240 -3.422466 -0.860185

81

ts-1a-m Eopt -2294.628843

C 7.160301 -1.590666 -1.438680

C 5.826470 0.003406 -2.877822

C 6.497292 0.176751 1.997693

C 5.664147 -2.225321 1.885755

C -6.450837 -3.292635 0.544663

C -2.965241 -7.319157 -0.504027

C 0.541501 2.630950 2.027804

C 0.329030 2.472653 0.638545

C 1.754398 2.274284 2.610869

C 1.387093 1.923143 -0.123039

C 2.792531 1.780624 1.816477

C 0.756839 -3.473607 -1.308205

C 0.713981 -2.363387 0.828469

C 2.064810 -3.044041 -1.507905

C 2.022153 -1.932136 0.639991

C -3.705133 -2.653233 0.378163

C -1.901195 -4.716407 -0.176511

C -1.901296 -1.030773 0.342032

C 3.792320 1.172974 -0.453314

C 6.278462 -0.325423 -1.439403

C 5.337481 -0.750258 1.575519

C 2.632274 1.590735 0.433037

C 0.045393 -3.111337 -0.151758

C -2.320563 -2.389503 0.265380

C 2.694598 -2.236561 -0.550100

C -1.397022 -3.417594 -0.008318

C -4.185118 -3.943337 0.215934

C -3.263801 -4.993766 -0.068186

N -1.614843 0.092486 0.386906

O -5.486332 -4.309985 0.295985

O -3.815750 -6.219303 -0.207710

O 3.947776 -1.784259 -0.825111

Si 4.805916 -0.440504 -0.235576

H 7.614582 -1.780617 -0.460352

H 6.580477 -2.478600 -1.715868

H 7.978289 -1.490194 -2.164686

H 5.296023 0.959828 -2.942253

H 6.694027 0.066049 -3.548133

H 5.162903 -0.775351 -3.271951

H 7.414601 -0.047357 1.440046

H 6.266862 1.237827 1.837708

H 6.727590 0.050665 3.063785

H 4.827040 -2.888350 1.643785

H 6.536139 -2.578961 1.324926

H 5.891538 -2.349810 2.953036

H -6.446528 -2.534184 -0.247924

H -7.416392 -3.799836 0.559251

H -6.278254 -2.806640 1.513339

H -3.621914 -8.186911 -0.579315

H -2.440667 -7.173755 -1.456816

H -2.231366 -7.484284 0.294932

H 1.914343 2.414892 3.677182

H 1.254600 1.819515 -1.198527

H 3.755682 1.578120 2.277551

H 0.260283 -4.051528 -2.083236

H 0.202561 -2.105056 1.750941

H 2.602672 -3.289851 -2.418147

H 2.504557 -1.336748 1.406974

H -4.377876 -1.824934 0.563305

H -1.199255 -5.517308 -0.375406

H 4.557190 1.966678 -0.420124

H 3.450863 1.158526 -1.496314

H 6.896098 0.514973 -1.083847

H 4.468789 -0.482372 2.194260

Pd -1.786656 2.102206 0.119897

C -4.510569 2.793510 -0.432071

O -5.717298 2.730561 -0.595096

O -3.751268 1.739037 -0.213894

C -3.790714 4.151315 -0.474637

H -4.001371 4.621885 -1.442253

H -4.232991 4.796512 0.295546

N -2.354755 3.985576 -0.267281

C -1.502890 4.969615 -0.464061

O -0.242366 4.851244 -0.338661

O -2.029621 6.160844 -0.812484

C -1.099579 7.231132 -1.028959

H -1.713414 8.092210 -1.297084

H -0.527541 7.438522 -0.120825

H -0.408921 6.987972 -1.840733

H -0.056678 3.603380 0.087836

H -0.237842 3.077419 2.640599

81

ts-1a-o Eopt -2294.624514

C -3.039875 1.992687 -3.253663

C -5.101517 1.924123 -1.784283

C -4.444136 3.201992 1.770460

C -4.061818 5.160506 0.191026

C 7.161781 -2.658074 0.694978

C 7.686054 2.595650 -0.544241

C -0.124591 -0.004606 3.789582

C -0.542655 -1.005428 2.915051

C -0.377930 1.325798 3.456764

C -1.206851 -0.715597 1.701483

C -1.059554 1.644511 2.278235

C 1.629063 1.323813 -1.629533

C 2.249419 2.925297 0.061570

C 0.386526 1.932454 -1.759715

C 1.003796 3.537434 -0.059207

C 4.802149 -1.172781 0.222383

C 5.064893 1.529905 -0.444763

C 2.363242 -0.959746 0.108277

C -2.317324 0.972440 0.153699

C -3.751766 2.625831 -2.043002

C -3.444311 3.897521 0.825190

C -1.484917 0.650948 1.389630

C 2.560736 1.775953 -0.682196

C 3.659973 -0.384863 -0.043770

C 0.040875 3.002495 -0.925236

C 3.777291 0.975979 -0.398101

C 6.068827 -0.613294 0.154991

C 6.200936 0.766960 -0.175148

N 1.291800 -1.383316 0.239535

O 7.230843 -1.272859 0.384718

O 7.469549 1.229702 -0.212622

O -1.218295 3.531183 -0.937496

Si -2.661543 2.738384 -0.478950

H -2.132662 2.542932 -3.524135

H -2.760933 0.947982 -3.063505

H -3.698701 1.992006 -4.132392

H -5.675028 2.399010 -0.980216

H -5.726887 1.945240 -2.686696

H -4.962711 0.869537 -1.515082

H -5.325117 2.830126 1.233372

H -3.991228 2.352802 2.293906

H -4.807612 3.904617 2.532405

H -3.347260 5.688463 -0.451235

H -4.942549 4.918971 -0.416553

H -4.389485 5.863285 0.968850

H 6.694285 -3.227956 -0.118287

H 8.194962 -2.984820 0.820525

H 6.608556 -2.833348 1.626935

H 8.766733 2.739110 -0.508047

H 7.320111 2.824735 -1.552873

H 7.203678 3.264131 0.179944

H -0.058597 2.125174 4.121380

H -1.252182 2.689522 2.059056

H 1.867417 0.467947 -2.254446

H 2.962239 3.302192 0.790608

H -0.331242 1.557160 -2.480313

H 0.742404 4.395481 0.552414

H 4.669838 -2.214576 0.486006

H 5.164382 2.571731 -0.724828

H -3.317675 0.539137 0.320566

H -1.920243 0.395201 -0.691913

H -3.968415 3.672076 -2.307673

H -2.595218 4.235968 1.437833

Pd -0.612173 -2.105756 0.115342

C -0.959158 -4.108795 -1.903021

O -0.765059 -4.888461 -2.820034

O -0.021058 -3.353385 -1.372328

C -2.368993 -3.969773 -1.301703

H -3.058225 -3.684044 -2.106168

H -2.689079 -4.955844 -0.944391

N -2.383123 -2.986863 -0.219393

C -3.455340 -2.775717 0.505446

O -3.508137 -1.948440 1.481974

O -4.558237 -3.477504 0.185199

C -5.721661 -3.259545 0.996719

H -6.484765 -3.919523 0.582179

H -5.521223 -3.516234 2.040108

H -6.046723 -2.217506 0.938404

H -2.355885 -1.428117 1.561640

H 0.387259 -0.254254 4.715418

H -0.357224 -2.045995 3.173074

81

ts-1b-p Eopt -2240.509257

C -7.328971 2.122744 0.364101

C -6.700510 1.458745 -1.992303

C -5.986884 -1.261097 2.081003

C -5.018188 0.908991 2.968228

C 6.570990 2.562615 -0.098096

C 3.215430 6.819529 -0.476686

C -0.297033 -2.524367 -0.767794

C -0.594520 -1.501944 -1.698973

C -1.357786 -2.945729 0.069750

C -1.867643 -0.951378 -1.799121

C -2.632621 -2.395916 -0.027761

C -0.805080 3.341785 -0.935339

C -0.542680 1.940405 1.003503

C -2.169228 3.065425 -0.953795

C -1.904252 1.656567 0.992472

C 3.805664 2.075634 0.027131

C 2.060038 4.254796 -0.170442

C 1.951729 0.545207 0.151225

C -4.308009 -0.831503 -1.132381

C -6.696252 1.022296 -0.512401

C -4.920284 -0.165247 1.866110

C -2.915535 -1.389348 -0.969569

C 0.040479 2.764037 0.027384

C 2.404649 1.891491 0.077900

C -2.724736 2.195850 -0.005532

C 1.507286 2.973640 -0.013113

C 4.329023 3.350104 -0.122059

C 3.439288 4.458319 -0.225093

N 1.685631 -0.580429 0.199606

O 5.649752 3.645554 -0.187359

O 4.035227 5.664764 -0.364049

O -4.062621 1.935851 -0.076326

Si -4.960109 0.506027 0.078668

H -7.474396 1.797701 1.399592

H -6.705944 3.024484 0.380990

H -8.313458 2.409136 -0.029592

H -6.366826 0.661738 -2.666056

H -7.713143 1.746230 -2.304875

H -6.049252 2.326454 -2.150241

H -7.002586 -0.852468 2.011721

H -5.907919 -2.075129 1.349892

H -5.885929 -1.709376 3.078022

H -4.276350 1.704624 2.843417

H -6.005135 1.384076 2.982788

H -4.861648 0.458701 3.957519

H 6.424351 1.842903 -0.912370

H 7.561650 3.012819 -0.177057

H 6.478820 2.038257 0.861034

H 3.902864 7.659602 -0.585823

H 2.563678 6.764222 -1.358040

H 2.601307 6.964702 0.421254

H 0.185446 -1.152469 -2.370964

H -1.182616 -3.747220 0.784381

H -2.064212 -0.178921 -2.539161

H -3.430369 -2.772378 0.609292

H -0.380671 3.980735 -1.705289

H 0.074227 1.516773 1.789982

H -2.816177 3.494044 -1.713010

H -2.325266 1.010878 1.754447

H 4.435475 1.193083 0.083388

H 1.386564 5.101654 -0.223769

H -5.041218 -1.649665 -1.070920

H -4.413575 -0.409470 -2.138960

H -7.325302 0.121652 -0.428111

H -3.936464 -0.649976 1.964140

Pd 1.667103 -2.570207 0.152226

C 1.380344 -5.257134 -0.893658

O 0.655945 -4.750575 -1.804496

H 0.296833 -3.573913 -1.339823

C 4.456264 -1.904275 0.976913

O 4.578211 -1.067436 0.077573

O 3.413876 -2.641284 1.231277

C 5.597804 -2.175513 1.961063

H 6.484807 -1.606900 1.672901

H 5.291460 -1.890349 2.973847

H 5.831798 -3.244489 1.983153

O 1.823317 -4.616525 0.111131

C 1.767416 -6.714627 -1.010495

H 2.632895 -6.791730 -1.678965

H 2.046006 -7.116246 -0.034780

H 0.948809 -7.289039 -1.449975

81

ts-1b-m Eopt -2240.501456

C 7.063184 -2.217086 -1.221358

C 5.959984 -0.566658 -2.787296

C 6.462444 -0.224547 2.090480

C 5.362418 -2.519317 2.072206

C -6.676405 -2.157198 0.650939

C -3.738867 -6.649979 -0.182500

C 0.853473 2.915743 1.768517

C 0.636146 2.646453 0.398015

C 2.010887 2.472652 2.402866

C 1.623119 1.903957 -0.291869

C 2.994243 1.798556 1.673587

C 0.464825 -3.354134 -1.293385

C 0.469184 -2.083601 0.752807

C 1.827378 -3.114134 -1.446997

C 1.830770 -1.844990 0.612361

C -3.908719 -1.865103 0.264985

C -2.370220 -4.174312 -0.123212

C -1.941516 -0.478277 -0.001830

C 3.947638 0.906976 -0.522064

C 6.317777 -0.870183 -1.317229

C 5.219595 -1.037409 1.669547

C 2.826008 1.503879 0.309251

C -0.245118 -2.814393 -0.208313

C -2.509469 -1.782528 0.082597

C 2.512710 -2.326767 -0.511231

C -1.716611 -2.933857 -0.088626

C -4.533004 -3.102545 0.250515

C -3.753123 -4.275365 0.035609

N -1.559838 0.611322 -0.101322

O -5.862481 -3.303374 0.424017

O -4.447664 -5.436211 0.024037

O 3.829188 -2.061668 -0.737913

Si 4.794313 -0.779817 -0.177270

H 7.452172 -2.407212 -0.215179

H 6.406976 -3.053213 -1.489122

H 7.918113 -2.233912 -1.910643

H 5.529606 0.432599 -2.916225

H 6.856185 -0.619814 -3.419656

H 5.239454 -1.295104 -3.177599

H 7.367824 -0.584989 1.587591

H 6.363699 0.844323 1.861359

H 6.635928 -0.311239 3.171158

H 4.462852 -3.095538 1.832363

H 6.206109 -3.001585 1.566455

H 5.537627 -2.606633 3.152903

H -6.651252 -1.473493 -0.206761

H -7.689688 -2.538567 0.786163

H -6.361549 -1.612192 1.548992

H -4.492939 -7.438236 -0.166380

H -3.226021 -6.654770 -1.152833

H -3.007536 -6.828349 0.616171

H 2.176021 2.687686 3.455689

H 1.481851 1.712401 -1.353638

H 3.925668 1.534771 2.167764

H -0.066455 -3.924906 -2.050590

H -0.048458 -1.691650 1.623263

H 2.370991 -3.497500 -2.304846

H 2.351714 -1.263838 1.364837

H -4.456918 -0.941080 0.418447

H -1.770520 -5.068532 -0.244033

H 4.786428 1.622475 -0.528740

H 3.619983 0.852837 -1.568205

H 7.003799 -0.080549 -0.970858

H 4.365342 -0.636735 2.234619

Pd -1.504044 2.612099 -0.118954

C -0.782036 5.389797 -0.527545

O 0.367612 4.969034 -0.843520

H 0.403433 3.711696 -0.309138

H 0.127777 3.506205 2.322149

C -4.437572 2.014596 -0.140815

O -3.465495 2.669588 -0.704815

O -4.356277 1.221176 0.801076

C -5.784107 2.314249 -0.806067

H -6.596284 1.885225 -0.214735

H -5.927131 3.393731 -0.910893

H -5.801150 1.884147 -1.814350

O -1.711453 4.650827 -0.065236

C -1.108928 6.854645 -0.721083

H -0.201475 7.458450 -0.660514

H -1.547298 6.983948 -1.717532

H -1.844334 7.181077 0.017119

81

ts-1b-o Eopt -2240.494954

C 3.524970 -1.719368 -3.359347

C 5.522197 -0.865198 -2.060979

C 5.391788 -1.699022 1.652512

C 5.496524 -3.918381 0.410499

C -6.922097 1.467827 0.476327

C -6.804092 -3.934588 -0.014616

C 0.697420 0.460825 3.738337

C 0.857934 1.450319 2.771164

C 1.133969 -0.832520 3.453190

C 1.441288 1.180796 1.512212

C 1.742836 -1.121261 2.228093

C -1.048467 -2.041669 -1.591215

C -1.217715 -3.343976 0.427743

C 0.296127 -2.374038 -1.703963

C 0.132641 -3.676580 0.325289

C -4.414131 0.237479 0.108100

C -4.339482 -2.553830 -0.173997

C -1.988628 0.318268 -0.043426

C 2.627086 -0.423661 -0.071152

C 4.414293 -1.938180 -2.120976

C 4.582798 -2.779668 0.907341

C 1.910994 -0.140266 1.244737

C -1.812457 -2.469387 -0.494282

C -3.192077 -0.442418 -0.084868

C 0.915527 -3.127461 -0.699360

C -3.136461 -1.840256 -0.250913

C -5.598144 -0.482715 0.164022

C -5.557308 -1.902178 0.033764

N -1.033873 0.968770 0.019912

O -6.830116 0.054086 0.340830

O -6.758103 -2.519685 0.109931

O 2.269418 -3.318383 -0.692494

Si 3.452797 -2.098602 -0.476436

H 2.813325 -2.539901 -3.497761

H 2.957780 -0.781495 -3.295474

H 4.138134 -1.660380 -4.268762

H 6.227536 -1.033793 -1.239863

H 6.102923 -0.859044 -2.993024

H 5.104206 0.141565 -1.936831

H 6.117245 -1.203766 0.996188

H 4.745745 -0.923740 2.078124

H 5.961180 -2.146043 2.478522

H 4.928214 -4.714894 -0.084031

H 6.248766 -3.553530 -0.299498

H 6.039505 -4.372105 1.250474

H -6.544894 1.981276 -0.416868

H -7.984302 1.683192 0.600885

H -6.369672 1.822396 1.355512

H -7.856106 -4.205083 0.086236

H -6.435001 -4.262784 -0.994603

H -6.223007 -4.425559 0.776377

H 1.015449 -1.623700 4.189751

H 2.080533 -2.135716 2.045773

H -1.503637 -1.403930 -2.343995

H -1.794476 -3.703976 1.275853

H 0.880545 -2.007307 -2.539841

H 0.608698 -4.304879 1.071865

H -4.391509 1.316979 0.206273

H -4.312897 -3.628990 -0.305378

H 3.425947 0.327175 -0.178861

H 1.938595 -0.196806 -0.897347

H 4.907445 -2.914022 -2.248114

H 3.888899 -3.226635 1.634297

Pd 0.399488 2.352702 -0.006354

C 2.920874 3.837526 0.306171

O 3.376353 2.870082 0.999815

H 2.405399 2.077254 1.186849

H 0.239937 0.691335 4.696764

H 0.523021 2.462565 2.988103

C -1.918406 3.704402 -1.195220

O -2.592038 3.413308 -0.205446

O -0.668011 3.395196 -1.413785

C -2.510014 4.521812 -2.346486

H -2.366278 4.001156 -3.298974

H -3.574027 4.696392 -2.172390

H -1.989955 5.483061 -2.421125

O 1.749158 3.892517 -0.167351

C 3.843266 5.006990 0.046560

H 4.055516 5.512251 0.994981

H 4.795075 4.641520 -0.350210

H 3.386044 5.710002 -0.650425

95

ts-2a Eopt -2650.982252

C 8.810278 -0.344150 -0.812297

C 7.946894 -1.253610 1.381794

C 6.475756 -2.333649 -3.232416

C 6.202078 0.188404 -3.357587

C -3.638849 5.675298 0.672787

C 1.265466 7.981644 1.030460

C 0.621995 -1.750895 0.021206

C 1.388546 -1.360919 1.122250

C 1.251946 -2.366033 -1.064104

C 2.766168 -1.589381 1.137304

C 2.634779 -2.588166 -1.038825

C 3.359884 3.083020 1.048830

C 2.428590 2.071357 -0.925147

C 4.467894 2.246780 0.956861

C 3.530894 1.228727 -1.024471

C -1.381458 4.027296 0.350495

C 1.155985 5.197991 0.529393

C -0.393525 1.853029 0.039387

C 4.917664 -2.403694 0.078171

C 7.810556 -1.316539 -0.153745

C 5.783350 -1.097762 -2.617440

C 3.419867 -2.197452 0.055197

C 2.304345 2.996408 0.123903

C -0.208064 3.250761 0.199347

C 4.551403 1.291469 -0.066896

C 1.080965 3.819154 0.276847

C -1.281295 5.387407 0.590965

C 0.011633 5.981422 0.685882

N -0.623733 0.723235 -0.078130

O -2.329363 6.229414 0.755663

O 0.016285 7.311818 0.918817

O 5.644584 0.481948 -0.084630

Si 6.011876 -1.048217 -0.720796

H 8.844652 -0.456639 -1.901109

H 8.554218 0.698791 -0.591420

H 9.826006 -0.520515 -0.433563

H 7.323670 -1.997972 1.890050

H 8.986383 -1.438512 1.683486

H 7.663295 -0.265469 1.761909

H 7.566596 -2.274966 -3.134552

H 6.151987 -3.272149 -2.765706

H 6.250734 -2.409748 -4.304260

H 5.698058 1.076595 -2.962761

H 7.280786 0.364807 -3.285113

H 5.956948 0.113740 -4.425563

H -3.799560 4.909938 1.441675

H -4.321964 6.510217 0.835090

H -3.823495 5.234089 -0.314526

H 1.022116 9.027118 1.224352

H 1.859486 7.583882 1.862893

H 1.843760 7.907175 0.100951

H 0.925423 -0.866213 1.970689

H 0.683470 -2.684548 -1.932662

H 3.344347 -1.273010 2.003016

H 3.101039 -3.087803 -1.886072

H 3.290846 3.784991 1.875707

H 1.654462 2.009316 -1.683884

H 5.268886 2.298648 1.687708

H 3.583402 0.510379 -1.834012

H -2.338100 3.517830 0.296652

H 2.135413 5.658459 0.574728

H 5.178093 -3.342720 -0.431602

H 5.254398 -2.521665 1.115263

H 8.075293 -2.338015 -0.471407

H 4.700705 -1.229148 -2.772015

Pd -1.325211 -1.202758 -0.146191

C -3.815355 0.648716 -0.526948

O -3.293454 -0.516216 -0.742370

O -3.439828 1.511405 0.272134

C -5.066346 0.954849 -1.374632

H -5.881058 1.212669 -0.686672

H -4.855278 1.861115 -1.957412

C -2.091171 -2.303293 1.645677

C -1.162588 -2.816633 2.557932

C -3.317065 -1.764897 2.039190

C -1.443437 -2.700916 3.915044

H -0.245075 -3.279825 2.220270

C -3.572799 -1.659562 3.406595

H -4.030259 -1.423824 1.302068

C -2.644585 -2.118318 4.349569

H -0.741625 -3.069310 4.657532

H -4.510218 -1.217757 3.737779

I -2.201932 -3.697599 -0.460080

C -2.936482 -2.005194 5.795314

O -2.190422 -2.378518 6.681011

H -3.917779 -1.539696 6.035700

N -5.427074 -0.155241 -2.231883

C -6.461852 -0.196929 -3.108009

O -6.737463 -1.135887 -3.836782

O -7.176230 0.969362 -3.070634

C -8.291451 1.004877 -3.963920

H -9.005936 0.210148 -3.730289

H -8.751937 1.983959 -3.818548

H -7.966079 0.887805 -5.001758

H -4.821180 -0.963769 -2.191807

95

ts-3a Eopt -2650.982990

C -7.840962 2.564118 1.255627

C -7.810289 1.243264 -0.900072

C -5.956683 -0.074412 3.501857

C -4.797721 2.177855 3.315769

C 5.477949 3.586861 -2.530914

C 1.648316 7.432950 -2.592262

C -0.993276 -1.877041 -0.291794

C -1.628870 -1.200054 -1.336587

C -1.730974 -2.303181 0.815198

C -2.982607 -0.889725 -1.235054

C -3.087651 -1.967311 0.902530

C -2.003787 3.590889 -1.836211

C -1.129683 2.448051 0.093967

C -3.295649 3.229978 -1.465939

C -2.417545 2.083871 0.473006

C 2.840915 2.832621 -1.872451

C 0.860939 4.812348 -1.862296

C 1.202758 1.157206 -1.294245

C -5.187005 -0.838560 0.005893

C -7.406942 1.242565 0.589094

C -5.004571 0.799490 2.655987

C -3.737555 -1.242765 -0.106014

C -0.891729 3.186922 -1.077054

C 1.500977 2.521325 -1.535031

C -3.513368 2.450956 -0.319284

C 0.492775 3.508212 -1.497439

C 3.185014 4.129905 -2.211605

C 2.170628 5.134392 -2.221645

N 1.023904 0.018928 -1.144152

O 4.428113 4.549052 -2.543651

O 2.591127 6.367156 -2.570469

O -4.795841 2.106609 -0.026724

Si -5.558570 0.854058 0.830239

H -7.697381 2.549437 2.341164

H -7.277908 3.415190 0.854903

H -8.905921 2.757144 1.069599

H -7.615072 0.282065 -1.388758

H -8.883202 1.450536 -1.007138

H -7.268167 2.017525 -1.455279

H -6.954730 0.374077 3.577761

H -6.078228 -1.083999 3.090142

H -5.572569 -0.185095 4.524065

H -4.103261 2.808965 2.751542

H -5.740156 2.728700 3.407208

H -4.391109 2.059990 4.328826

H 5.293996 2.786066 -3.258010

H 6.380536 4.132542 -2.808655

H 5.605189 3.147276 -1.533897

H 2.208945 8.313725 -2.907422

H 0.841034 7.236569 -3.308699

H 1.221099 7.607459 -1.597120

H -1.083683 -0.911139 -2.225579

H -1.270605 -2.867966 1.615920

H -3.457194 -0.352305 -2.052579

H -3.643010 -2.288479 1.781210

H -1.851601 4.161326 -2.748864

H -0.297395 2.161354 0.730021

H -4.150969 3.522426 -2.067022

H -2.564335 1.500656 1.374591

H 3.574766 2.035194 -1.843674

H 0.105852 5.588028 -1.824068

H -5.748544 -1.589001 0.580219

H -5.641689 -0.815969 -0.991902

H -7.951981 0.421906 1.082614

H -4.028662 0.289066 2.644936

Pd 1.082008 -1.731140 0.030124

C 3.567448 -0.205087 0.605455

O 2.500393 -0.705200 1.158858

O 3.890801 -0.255794 -0.579905

C 4.501474 0.507281 1.598432

H 4.711674 1.504901 1.198059

H 5.454098 -0.040761 1.588544

C 0.481886 -3.132351 -1.408257

C -0.029267 -4.422721 -1.208371

C 1.006966 -2.761122 -2.652981

C 0.017791 -5.347287 -2.244875

H -0.459392 -4.704975 -0.256607

C 1.059618 -3.704395 -3.680813

H 1.392233 -1.762921 -2.823682

C 0.566209 -4.998965 -3.488746

H -0.371065 -6.351921 -2.108064

H 1.488893 -3.425060 -4.641059

I 1.581340 -3.668013 1.798411

C 0.613453 -5.982408 -4.591988

O 0.188500 -7.120393 -4.525044

H 1.079213 -5.603830 -5.529491

N 3.937991 0.595731 2.929905

C 4.489711 1.247723 3.986887

O 4.043204 1.260919 5.121233

O 5.622179 1.913500 3.607848

C 6.258672 2.640359 4.662095

H 6.554276 1.970955 5.474859

H 7.138950 3.100887 4.209827

H 5.592204 3.408171 5.065836

H 3.136205 0.011677 3.124246

87

ts-2b Eopt -2367.750881

C 8.250116 -0.434323 0.651548

C 6.940320 -1.252270 2.651645

C 6.419879 -2.516076 -2.120509

C 6.238975 0.001315 -2.400195

C -4.183101 5.670348 -0.622335

C 0.541985 7.975657 0.743216

C 0.047371 -1.762443 -0.224462

C 0.564801 -1.322896 0.997708

C 0.893181 -2.435680 -1.111425

C 1.902466 -1.553791 1.325226

C 2.234458 -2.660509 -0.775553

C 2.566808 3.079446 1.242967

C 2.073862 2.037515 -0.868255

C 3.661604 2.236465 1.405514

C 3.163840 1.187206 -0.713483

C -1.910999 4.021287 -0.460309

C 0.533931 5.190008 0.244573

C -0.887274 1.845006 -0.546732

C 4.223142 -2.422121 0.801502

C 7.121175 -1.372708 1.124164

C 5.646593 -1.243865 -1.710372

C 2.768333 -2.216048 0.442417

C 1.734094 2.981592 0.114151

C -0.733973 3.243070 -0.356473

C 3.954573 1.261801 0.440313

C 0.510467 3.809295 -0.009142

C -1.860746 5.382632 -0.210640

C -0.615570 5.975723 0.150418

N -1.096316 0.716933 -0.708669

O -2.919267 6.226154 -0.272957

O -0.656694 7.308377 0.371321

O 5.019559 0.446810 0.670441

Si 5.481055 -1.113909 0.189107

H 8.506568 -0.589293 -0.401811

H 7.970283 0.618381 0.776517

H 9.163175 -0.603538 1.238165

H 6.215485 -1.972614 3.047185

H 7.893042 -1.432367 3.167210

H 6.599055 -0.248464 2.929736

H 7.468237 -2.471265 -1.800996

H 5.983264 -3.428098 -1.695169

H 6.419244 -2.634466 -3.212044

H 5.690726 0.915135 -2.148883

H 7.285838 0.160470 -2.119544

H 6.210441 -0.114461 -3.492069

H -4.498523 4.906343 0.098456

H -4.885540 6.504982 -0.606633

H -4.159934 5.226335 -1.625333

H 0.266824 9.022909 0.875653

H 0.943872 7.581338 1.685120

H 1.305279 7.894771 -0.041001

H -0.064400 -0.784894 1.700830

H 0.523299 -2.796611 -2.066850

H 2.282208 -1.196726 2.280476

H 2.867464 -3.203798 -1.475148

H 2.326959 3.795645 2.024505

H 1.480576 1.964952 -1.774557

H 4.286848 2.297691 2.290835

H 3.382942 0.453337 -1.480038

H -2.836189 3.510334 -0.708504

H 1.482419 5.649532 0.494822

H 4.576501 -3.388357 0.412626

H 4.325176 -2.482332 1.891901

H 7.429863 -2.407665 0.904963

H 4.615292 -1.365070 -2.077627

Pd -1.807208 -1.199618 -0.847157

C -4.096914 0.652294 -1.790840

O -3.555188 -0.525632 -1.889178

O -3.911884 1.478070 -0.888604

C -5.047547 0.969001 -2.949549

H -5.697942 0.114838 -3.160904

H -5.648504 1.851970 -2.718881

H -4.463044 1.161400 -3.857420

C -2.973842 -2.205411 0.785433

C -2.275706 -2.675181 1.904230

C -4.236291 -1.617211 0.877668

C -2.832524 -2.467372 3.161110

H -1.322118 -3.174774 1.796764

C -4.771166 -1.419286 2.150468

H -4.762042 -1.305735 -0.013599

C -4.079160 -1.835047 3.295057

H -2.315395 -2.800107 4.056330

H -5.740805 -0.935657 2.248348

I -2.660018 -3.700920 -1.204056

C -4.666685 -1.623163 4.634879

O -4.141235 -1.955151 5.681349

H -5.659665 -1.121231 4.631316

87

ts-3b Eopt -2367.753883

C 7.990806 -0.990503 -0.024990

C 6.986978 -1.726359 2.175882

C 5.559718 -3.015656 -2.386028

C 5.520320 -0.500618 -2.733218

C -3.873291 6.199308 0.220313

C 1.199126 8.047136 0.789523

C -0.278955 -1.777687 0.240472

C 0.408110 -1.223335 1.324559

C 0.365395 -2.691370 -0.597188

C 1.751692 -1.530733 1.523156

C 1.720937 -2.975362 -0.388033

C 2.813424 2.977773 1.365250

C 1.865938 1.919560 -0.577955

C 3.845510 2.044350 1.371431

C 2.894034 0.982674 -0.580594

C -1.781327 4.309368 0.294026

C 0.843050 5.248059 0.553900

C -0.996931 2.027983 0.312701

C 3.916818 -2.662504 0.865122

C 6.900401 -1.854610 0.640807

C 4.966269 -1.674635 -1.901143

C 2.445533 -2.393357 0.660863

C 1.788272 2.920202 0.405053

C -0.687106 3.411633 0.328036

C 3.881822 1.020777 0.412338

C 0.645286 3.863662 0.432731

C -1.561456 5.672384 0.398720

C -0.223934 6.147583 0.546536

N -1.329244 0.915459 0.328478

O -2.523063 6.624958 0.373180

O -0.101132 7.487550 0.648063

O 4.897866 0.120355 0.490663

Si 5.149891 -1.483074 -0.010339

H 8.052371 -1.158250 -1.105453

H 7.805678 0.077747 0.137782

H 8.977386 -1.221985 0.398305

H 6.301172 -2.406491 2.693403

H 8.001655 -1.960211 2.524321

H 6.754284 -0.705445 2.500366

H 6.648685 -3.044375 -2.257739

H 5.140880 -3.878024 -1.852825

H 5.355292 -3.164426 -3.454199

H 5.093883 0.461403 -2.430589

H 6.609085 -0.418731 -2.643574

H 5.292695 -0.644871 -3.797753

H -4.186923 5.559432 1.054538

H -4.470707 7.111876 0.214885

H -4.017873 5.658560 -0.723160

H 1.048295 9.124761 0.863898

H 1.693686 7.682808 1.698582

H 1.826415 7.824748 -0.082616

H -0.090228 -0.555160 2.014989

H -0.157533 -3.167769 -1.417041

H 2.271428 -1.080835 2.365802

H 2.212343 -3.674120 -1.062004

H 2.779686 3.738558 2.140867

H 1.116211 1.874017 -1.362281

H 4.622034 2.075701 2.129357

H 2.913222 0.212540 -1.342864

H -2.777138 3.897578 0.174612

H 1.859343 5.617164 0.619291

H 4.169193 -3.676828 0.524457

H 4.151017 -2.634901 1.936277

H 7.099720 -2.906794 0.381565

H 3.880573 -1.723293 -2.079954

Pd -1.985242 -0.868203 -0.590246

C -3.426283 1.347845 -1.892795

O -2.594516 0.366161 -2.133941

O -3.896421 1.656112 -0.797496

C -3.793552 2.105417 -3.168027

H -2.895903 2.374203 -3.733302

H -4.398723 1.454807 -3.808584

H -4.366406 3.001081 -2.917944

C -2.328243 -2.033664 1.116218

C -2.416772 -3.429261 1.220613

C -2.834218 -1.213108 2.132879

C -3.040087 -3.997404 2.325080

H -2.005547 -4.066894 0.449142

C -3.469222 -1.796937 3.230535

H -2.762930 -0.133731 2.068686

C -3.575697 -3.187479 3.338544

H -3.119902 -5.076119 2.421526

H -3.884246 -1.159929 4.009322

I -3.009821 -2.769222 -2.165109

C -4.235671 -3.791065 4.514965

O -4.355351 -4.986663 4.707796

H -4.633544 -3.055052 5.249581

81

ts-1a-m Eopt -2294.628843

C 7.160301 -1.590666 -1.438680

C 5.826470 0.003406 -2.877822

C 6.497292 0.176751 1.997693

C 5.664147 -2.225321 1.885755

C -6.450837 -3.292635 0.544663

C -2.965241 -7.319157 -0.504027

C 0.541501 2.630950 2.027804

C 0.329030 2.472653 0.638545

C 1.754398 2.274284 2.610869

C 1.387093 1.923143 -0.123039

C 2.792531 1.780624 1.816477

C 0.756839 -3.473607 -1.308205

C 0.713981 -2.363387 0.828469

C 2.064810 -3.044041 -1.507905

C 2.022153 -1.932136 0.639991

C -3.705133 -2.653233 0.378163

C -1.901195 -4.716407 -0.176511

C -1.901296 -1.030773 0.342032

C 3.792320 1.172974 -0.453314

C 6.278462 -0.325423 -1.439403

C 5.337481 -0.750258 1.575519

C 2.632274 1.590735 0.433037

C 0.045393 -3.111337 -0.151758

C -2.320563 -2.389503 0.265380

C 2.694598 -2.236561 -0.550100

C -1.397022 -3.417594 -0.008318

C -4.185118 -3.943337 0.215934

C -3.263801 -4.993766 -0.068186

N -1.614843 0.092486 0.386906

O -5.486332 -4.309985 0.295985

O -3.815750 -6.219303 -0.207710

O 3.947776 -1.784259 -0.825111

Si 4.805916 -0.440504 -0.235576

H 7.614582 -1.780617 -0.460352

H 6.580477 -2.478600 -1.715868

H 7.978289 -1.490194 -2.164686

H 5.296023 0.959828 -2.942253

H 6.694027 0.066049 -3.548133

H 5.162903 -0.775351 -3.271951

H 7.414601 -0.047357 1.440046

H 6.266862 1.237827 1.837708

H 6.727590 0.050665 3.063785

H 4.827040 -2.888350 1.643785

H 6.536139 -2.578961 1.324926

H 5.891538 -2.349810 2.953036

H -6.446528 -2.534184 -0.247924

H -7.416392 -3.799836 0.559251

H -6.278254 -2.806640 1.513339

H -3.621914 -8.186911 -0.579315

H -2.440667 -7.173755 -1.456816

H -2.231366 -7.484284 0.294932

H 1.914343 2.414892 3.677182

H 1.254600 1.819515 -1.198527

H 3.755682 1.578120 2.277551

H 0.260283 -4.051528 -2.083236

H 0.202561 -2.105056 1.750941

H 2.602672 -3.289851 -2.418147

H 2.504557 -1.336748 1.406974

H -4.377876 -1.824934 0.563305

H -1.199255 -5.517308 -0.375406

H 4.557190 1.966678 -0.420124

H 3.450863 1.158526 -1.496314

H 6.896098 0.514973 -1.083847

H 4.468789 -0.482372 2.194260

Pd -1.786656 2.102206 0.119897

C -4.510569 2.793510 -0.432071

O -5.717298 2.730561 -0.595096

O -3.751268 1.739037 -0.213894

C -3.790714 4.151315 -0.474637

H -4.001371 4.621885 -1.442253

H -4.232991 4.796512 0.295546

N -2.354755 3.985576 -0.267281

C -1.502890 4.969615 -0.464061

O -0.242366 4.851244 -0.338661

O -2.029621 6.160844 -0.812484

C -1.099579 7.231132 -1.028959

H -1.713414 8.092210 -1.297084

H -0.527541 7.438522 -0.120825

H -0.408921 6.987972 -1.840733

H -0.056678 3.603380 0.087836

H -0.237842 3.077419 2.640599

102

ts-1a-m1 Eopt -2794.844041

C -4.806957 6.801504 1.170344

C -2.937284 5.842382 2.575916

C -3.114787 6.282308 -2.328837

C -5.317660 5.039202 -2.047119

C -4.088013 -6.984203 -0.120810

C -8.632753 -4.231442 0.987929

C 0.385539 0.875217 -2.235949

C 0.327223 0.707553 -0.832765

C -0.211613 1.972706 -2.850038

C -0.373670 1.685724 -0.088743

C -0.852251 2.943270 -2.075356

C -5.503156 0.161821 1.418817

C -4.495759 0.204832 -0.768237

C -5.312141 1.534720 1.536426

C -4.303332 1.577568 -0.661807

C -3.953837 -4.163261 -0.107400

C -6.285300 -2.734161 0.476203

C -2.688466 -2.097404 -0.238601

C -1.531739 3.931468 0.178969

C -3.406300 6.156419 1.139563

C -3.794943 4.999130 -1.805735

C -0.950065 2.821513 -0.678462

C -5.066280 -0.533950 0.278700

C -3.942944 -2.749777 -0.070101

C -4.674825 2.247282 0.510637

C -5.109378 -2.013533 0.216680

C -5.125939 -4.858793 0.144405

C -6.313397 -4.128260 0.443321

N -1.639283 -1.617027 -0.357683

O -5.251894 -6.207134 0.141175

O -7.409862 -4.884019 0.672225

O -4.447769 3.574362 0.704797

Si -3.310389 4.627466 0.006625

H -5.118827 7.167814 0.186306

H -5.564387 6.089142 1.517245

H -4.820737 7.658685 1.856893

H -1.900190 5.490999 2.610951

H -2.998629 6.740474 3.204820

H -3.567572 5.073922 3.038847

H -3.476013 7.172960 -1.800722

H -2.023273 6.254881 -2.217460

H -3.329671 6.429113 -3.395483

H -5.805163 4.110554 -1.732769

H -5.797005 5.861662 -1.505229

H -5.531596 5.184506 -3.114534

H -3.307784 -6.802260 0.628604

H -4.410593 -8.024756 -0.066571

H -3.686304 -6.777362 -1.120883

H -9.361603 -5.029031 1.137916

H -8.544269 -3.639218 1.907468

H -8.963991 -3.583125 0.166886

H -0.147824 2.101778 -3.927792

H -0.405758 1.590660 0.995172

H -1.246611 3.829734 -2.565210

H -5.947032 -0.388284 2.244405

H -4.188099 -0.300523 -1.678832

H -5.613392 2.067774 2.432729

H -3.839088 2.117442 -1.479421

H -3.025673 -4.684391 -0.307130

H -7.191465 -2.178676 0.685994

H -0.892499 4.823477 0.070675

H -1.436530 3.647197 1.234777

H -2.706101 6.893135 0.714033

H -3.402924 4.160998 -2.400068

Pd 0.378632 -1.409912 -0.211783

C 1.589684 -3.935135 0.387938

O 1.762673 -5.124281 0.594719

O 0.403028 -3.390627 0.216884

C 2.792431 -2.979096 0.312571

H 3.355679 -3.061168 1.249599

H 3.453533 -3.331899 -0.490290

N 2.354681 -1.607104 0.074270

C 3.169009 -0.580178 0.199168

O 2.808245 0.630412 0.050314

O 4.454015 -0.859930 0.501396

C 5.346641 0.265680 0.644756

H 5.375250 0.833062 -0.289542

H 4.966061 0.920654 1.433932

H 1.531357 0.565001 -0.322407

H 0.941240 0.160125 -2.837608

C 6.730992 -0.281655 1.005636

C 7.704065 0.846376 1.321106

H 6.607319 -0.943572 1.875206

C 7.437862 -1.010369 -0.129110

C 7.609932 1.844814 2.284656

C 8.820559 0.782888 0.462316

C 7.045167 -2.146277 -0.830310

C 8.656232 -0.368093 -0.435473

C 8.639846 2.787822 2.388942

H 6.754853 1.896721 2.954745

C 9.847466 1.723261 0.568822

C 7.874961 -2.635624 -1.846412

H 6.110832 -2.645783 -0.595087

C 9.482458 -0.858975 -1.448552

C 9.747687 2.725761 1.536807

H 8.578711 3.571826 3.138787

H 10.712592 1.679718 -0.087810

C 9.081652 -1.996869 -2.152425

H 7.578889 -3.522176 -2.400641

H 10.423050 -0.368946 -1.687121

H 10.539981 3.463807 1.630218

H 9.714647 -2.391521 -2.942863

102

ts-1a-o1 Eopt -2794.839551

C 0.241714 3.459299 -3.409129

C -1.790972 4.230824 -2.110048

C -1.008071 5.020491 1.559048

C 0.311970 6.672905 0.143571

C 7.146197 -5.149752 0.994879

C 9.931938 -0.573745 0.151567

C 1.354274 0.228412 3.688941

C 0.648909 -0.478764 2.717621

C 1.710420 1.551550 3.429175

C 0.290342 0.098599 1.478250

C 1.344467 2.161322 2.225261

C 4.035857 0.854492 -1.482986

C 5.092012 1.996507 0.358211

C 3.177281 1.931563 -1.667453

C 4.231141 3.077759 0.183130

C 5.679470 -2.796454 0.439868

C 7.109137 -0.438240 -0.023237

C 3.575712 -1.572286 0.151282

C 0.140809 2.141799 -0.032359

C -0.253293 4.303037 -2.218955

C 0.277954 5.251233 0.740565

C 0.637069 1.464343 1.239714

C 4.971338 0.845642 -0.436737

C 5.002057 -1.593608 0.139076

C 3.224981 3.022149 -0.790471

C 5.710166 -0.399484 -0.113463

C 7.063946 -2.820858 0.508294

C 7.791151 -1.616011 0.281990

N 2.418073 -1.508143 0.170399

O 7.816489 -3.914522 0.783126

O 9.134014 -1.729950 0.373667

O 2.306753 4.030321 -0.864836

Si 0.627569 3.905202 -0.571571

H 1.316972 3.583272 -3.574709

H 0.040436 2.389807 -3.263968

H -0.270120 3.757723 -4.333884

H -2.187858 4.883177 -1.323976

H -2.259707 4.536779 -3.054809

H -2.132824 3.209859 -1.898347

H -1.906977 5.071595 0.932777

H -1.007009 4.046021 2.059722

H -1.115162 5.790657 2.334732

H 1.240579 6.864829 -0.406565

H -0.525452 6.843160 -0.544106

H 0.235755 7.427832 0.937623

H 6.566168 -5.447282 0.111842

H 7.931320 -5.884494 1.178479

H 6.481622 -5.101081 1.867392

H 10.964663 -0.900282 0.280392

H 9.795648 -0.183478 -0.864719

H 9.704052 0.215084 0.879439

H 2.263882 2.123433 4.170317

H 1.625314 3.196828 2.063830

H 3.958843 -0.003385 -2.144795

H 5.820216 2.017589 1.165008

H 2.444397 1.913663 -2.466115

H 4.289351 3.947499 0.830287

H 5.099726 -3.692500 0.622394

H 7.660848 0.472170 -0.224491

H -0.960146 2.166816 0.027683

H 0.341395 1.475217 -0.881712

H 0.013988 5.349230 -2.432485

H 1.126693 5.182314 1.437521

Pd 0.408268 -1.364658 -0.146832

C -0.535431 -2.980810 -2.315661

O -0.594993 -3.741935 -3.266353

O 0.574571 -2.697815 -1.669291

C -1.815442 -2.292787 -1.807386

H -2.257907 -1.731608 -2.639859

H -2.532553 -3.076068 -1.532466

N -1.528834 -1.419502 -0.671247

C -2.482202 -0.793033 -0.023620

O -2.280201 -0.042231 0.993844

O -3.740826 -0.953503 -0.475134

C -4.790777 -0.279123 0.253544

H -4.802990 -0.631794 1.288528

H -4.584097 0.794889 0.257420

H -1.029723 -0.059301 1.200572

H 1.620326 -0.239751 4.633110

H 0.358576 -1.508017 2.917618

C -6.116869 -0.579639 -0.451530

C -7.257647 0.213927 0.171824

H -5.993683 -0.318763 -1.512765

C -6.601844 -2.015273 -0.301403

C -7.396013 1.590376 0.314284

C -8.260138 -0.659205 0.641505

C -5.995866 -3.200229 -0.707178

C -7.854480 -2.040045 0.347451

C -8.545521 2.097488 0.932625

H -6.630035 2.270626 -0.051131

C -9.406436 -0.151635 1.256705

C -6.644904 -4.414654 -0.453541

H -5.036290 -3.186162 -1.213932

C -8.500136 -3.252478 0.598364

C -9.540302 1.231719 1.399568

H -8.666230 3.171105 1.048341

H -10.184941 -0.817805 1.619436

C -7.884712 -4.439487 0.194456

H -6.180959 -5.346193 -0.765964

H -9.466142 -3.276895 1.096291

H -10.427553 1.639602 1.876552

H -8.375395 -5.390932 0.381835

102

ts-1a-p1 Eopt -2794.849250

C -6.226202 6.348392 0.330641

C -4.796684 5.967380 2.379011

C -3.468470 5.920432 -2.389639

C -5.353819 4.223724 -2.549611

C -3.391021 -7.278996 -0.021211

C -8.130905 -4.890971 1.120310

C 0.260155 0.806702 0.054013

C -0.410167 1.039766 1.278593

C 0.018051 1.744540 -0.980346

C -1.238136 2.142440 1.465947

C -0.798668 2.855839 -0.793635

C -5.537300 -0.254682 1.455118

C -4.335456 -0.148259 -0.627491

C -5.575159 1.135027 1.507145

C -4.365052 1.240905 -0.583024

C -3.464511 -4.455603 0.063709

C -5.901892 -3.216137 0.627442

C -2.355770 -2.306779 -0.069549

C -2.238944 4.336924 0.695313

C -4.800305 6.050776 0.838380

C -4.009168 4.567466 -1.877463

C -1.433974 3.085108 0.440936

C -4.896899 -0.924487 0.398166

C -3.560757 -3.044818 0.106878

C -4.967059 1.895494 0.498475

C -4.785642 -2.401345 0.375372

C -4.579849 -5.242445 0.302310

C -5.821076 -4.607503 0.599084

N -1.341218 -1.761690 -0.204828

O -4.602492 -6.596374 0.283041

O -6.856962 -5.446292 0.820624

O -5.012641 3.251534 0.612894

Si -4.029936 4.505768 0.032715

H -6.251224 6.550511 -0.745320

H -6.902260 5.508528 0.529446

H -6.639103 7.230342 0.838362

H -3.787525 5.861726 2.792575

H -5.232268 6.876905 2.813419

H -5.392345 5.116481 2.729047

H -4.145514 6.746274 -2.139899

H -2.482846 6.163502 -1.973528

H -3.364676 5.906213 -3.482417

H -5.738197 3.249169 -2.231176

H -6.123647 4.969386 -2.323180

H -5.240122 4.196180 -3.641554

H -2.613554 -7.065351 0.723122

H -3.637042 -8.341322 0.004734

H -3.021277 -7.012414 -1.019232

H -8.795881 -5.743000 1.267592

H -8.099129 -4.288942 2.037222

H -8.503746 -4.275160 0.292164

H -0.248506 0.358065 2.110564

H 0.532157 1.623896 -1.931594

H -1.715736 2.301268 2.430461

H -0.911764 3.581318 -1.596959

H -5.975204 -0.829771 2.266761

H -3.872419 -0.633940 -1.480749

H -6.049187 1.651266 2.336081

H -3.907738 1.810449 -1.383256

H -2.501535 -4.903066 -0.149393

H -6.853545 -2.734958 0.817769

H -1.704848 5.204106 0.278011

H -2.294723 4.513125 1.776046

H -4.156395 6.896155 0.547206

H -3.283676 3.798443 -2.184850

Pd 0.599083 -1.269509 -0.541771

C 2.075603 -3.455391 -1.655089

O 2.383238 -4.535921 -2.129998

O 0.875192 -3.172048 -1.197312

C 3.121467 -2.328655 -1.567604

H 3.991238 -2.716520 -1.025777

H 3.456182 -2.094686 -2.587020

N 2.568100 -1.150235 -0.906375

C 3.330268 -0.197497 -0.413531

O 2.883808 0.801780 0.238994

O 4.657075 -0.301463 -0.635902

C 5.491066 0.737946 -0.080096

H 5.139378 1.706725 -0.445296

H 5.408116 0.730556 1.010576

H 1.574807 0.690783 0.199158

C 6.931984 0.473945 -0.528458

C 7.588771 -0.735469 0.123080

H 6.922383 0.368713 -1.623169

C 7.853126 1.609775 -0.103104

C 7.217202 -2.075487 0.076554

C 8.731501 -0.336964 0.848473

C 7.775198 2.964943 -0.405731

C 8.894629 1.116178 0.709098

C 7.989346 -3.017575 0.766846

H 6.344049 -2.388459 -0.486718

C 9.500627 -1.278546 1.535341

C 8.745990 3.833178 0.108665

H 6.977882 3.352380 -1.035884

C 9.862649 1.983313 1.220184

C 9.119560 -2.621548 1.490358

H 7.707684 -4.066599 0.737746

H 10.382844 -0.976705 2.094233

C 9.779434 3.344157 0.914951

H 8.696926 4.893881 -0.121980

H 10.669891 1.610286 1.845251

H 9.708576 -3.366191 2.019286

H 10.526414 4.029694 1.306417

93

ts-1aa-m Eopt -3084.442245

C -8.652439 1.439698 -0.824313

C -7.460370 -0.156542 -2.380240

C -7.580472 -0.265129 2.545669

C -6.812276 2.148888 2.304766

C 5.053504 3.461516 -0.196584

C 1.397703 7.367021 -1.124806

C -1.582504 -2.585521 1.911145

C -1.559020 -2.459676 0.501735

C -2.722296 -2.250576 2.636213

C -2.723803 -1.960642 -0.130878

C -3.867663 -1.805596 1.970474

C -2.298351 3.411321 -1.430716

C -2.032824 2.408415 0.741897

C -3.606868 2.941989 -1.490130

C -3.339832 1.935941 0.693099

C 2.321021 2.753541 -0.132426

C 0.423796 4.764415 -0.596158

C 0.539538 1.106935 0.027583

C -5.169373 -1.274993 -0.162076

C -7.756517 0.186841 -0.905140

C -6.493621 0.674827 1.981453

C -3.895051 -1.645932 0.573567

C -1.475383 3.122945 -0.328758

C 0.936094 2.465925 -0.109256

C -4.124819 2.167123 -0.442959

C -0.033301 3.465249 -0.332927

C 2.754231 4.046835 -0.389611

C 1.785247 5.067131 -0.627741

N 0.241970 -0.012644 0.103383

O 4.043134 4.441580 -0.448109

O 2.294688 6.295628 -0.866969

O -5.387032 1.674760 -0.580775

Si -6.162641 0.341704 0.127563

H -9.000969 1.637348 0.195017

H -8.120048 2.331559 -1.174428

H -9.542703 1.317258 -1.455676

H -6.925343 -1.106225 -2.490597

H -8.395402 -0.241489 -2.949677

H -6.856710 0.625810 -2.855098

H -8.558791 -0.067099 2.091503

H -7.348950 -1.324727 2.377366

H -7.692119 -0.124238 3.628759

H -6.022627 2.823423 1.957919

H -7.750194 2.475870 1.843048

H -6.917999 2.287766 3.389059

H 5.007436 2.636945 -0.915476

H 6.002417 3.988917 -0.298129

H 4.964695 3.054316 0.815268

H 2.025972 8.242267 -1.295795

H 0.789195 7.174003 -2.017496

H 0.738118 7.552764 -0.267533

H -2.740229 -2.366581 3.716989

H -2.732511 -1.882207 -1.216375

H -4.768353 -1.614227 2.547983

H -1.889841 3.962858 -2.273365

H -1.432754 2.208120 1.624614

H -4.231413 3.131718 -2.357463

H -3.735597 1.368117 1.527725

H 3.029968 1.948482 0.022556

H -0.309908 5.545070 -0.757593

H -5.913396 -2.070587 0.008832

H -4.972747 -1.302555 -1.241276

H -8.318085 -0.656753 -0.472636

H -5.555890 0.432772 2.502755

Pd 0.409220 -1.993588 -0.320928

C 3.011048 -2.539189 -1.348806

O 4.207107 -2.417567 -1.647652

O 2.266580 -1.531862 -1.019776

C 2.354950 -3.920358 -1.372141

H 2.400979 -4.305886 -2.398702

H 2.954269 -4.599885 -0.753126

N 0.976694 -3.832461 -0.893333

C 0.168766 -4.874329 -0.916127

O -1.038354 -4.840662 -0.523146

O 0.690909 -6.024237 -1.385798

C -0.184457 -7.161969 -1.407499

H 0.417858 -7.976811 -1.811170

H -0.528960 -7.404738 -0.399076

H -1.049590 -6.972956 -2.048186

H -1.225005 -3.591273 -0.058580

H -0.714539 -2.987671 2.427573

H 4.671407 -0.807434 -1.196581

O 5.084230 -0.046354 -0.704442

C 5.815605 -0.601412 0.341731

H 6.026985 -1.666246 0.181969

C 5.013092 -0.510758 1.650211

C 7.174182 0.104559 0.427210

F 4.607690 0.750663 1.912827

F 3.908923 -1.277552 1.559705

F 5.721828 -0.940658 2.714139

F 7.051355 1.416424 0.729246

F 7.813567 0.013814 -0.749304

F 7.962002 -0.453817 1.368956

93

ts-1aa-o Eopt -3084.433547

C -4.399177 -0.113583 -3.649207

C -6.041203 -1.551061 -2.366759

C -6.933430 -0.135466 1.070723

C -7.756938 1.532216 -0.665922

C 5.762487 3.084842 1.192417

C 2.946997 7.414038 -0.482265

C -2.066783 0.486548 3.895874

C -1.572656 -0.566293 3.130530

C -3.045202 1.313404 3.343928

C -2.029241 -0.819219 1.814935

C -3.538853 1.071649 2.058251

C -0.780541 2.533294 -1.622524

C -1.555134 4.165728 -0.028688

C -2.091775 2.157536 -1.887522

C -2.873062 3.788910 -0.283346

C 3.025744 2.730402 0.600090

C 1.574838 4.943503 -0.317057

C 1.004426 1.359083 0.456436

C -3.649782 -0.317568 -0.087061

C -5.501713 -0.119677 -2.573613

C -6.546916 1.044986 0.157198

C -3.055719 0.017482 1.275739

C -0.486282 3.505450 -0.653899

C 1.662271 2.607011 0.253749

C -3.143920 2.736273 -1.167716

C 0.924155 3.705279 -0.235300

C 3.662667 3.956649 0.486542

C 2.917329 5.086938 0.038545

N 0.434604 0.366328 0.642327

O 4.965252 4.188488 0.770592

O 3.612462 6.243182 -0.026419

O -4.428571 2.289090 -1.306464

Si -5.008070 0.725713 -0.932561

H -4.083508 0.903890 -3.901910

H -3.512465 -0.676914 -3.329742

H -4.759829 -0.584326 -4.573587

H -6.884598 -1.586657 -1.668169

H -6.389343 -1.972869 -3.318934

H -5.264003 -2.224066 -1.983199

H -7.242878 -1.015697 0.494502

H -6.106544 -0.438730 1.722240

H -7.779499 0.137569 1.715523

H -7.507775 2.399314 -1.288702

H -8.141167 0.745173 -1.326159

H -8.581013 1.827529 -0.002782

H 5.776467 2.286467 0.441999

H 6.769121 3.484033 1.323202

H 5.404298 2.679645 2.147779

H 3.692477 8.209414 -0.444431

H 2.590849 7.295581 -1.513286

H 2.102197 7.673135 0.168420

H -3.442249 2.146607 3.918876

H -4.309637 1.728083 1.669321

H 0.030356 2.044750 -2.155483

H -1.351953 4.934284 0.712687

H -2.299740 1.390494 -2.624393

H -3.698826 4.263878 0.237466

H 3.560013 1.851465 0.934490

H 1.019928 5.792363 -0.698333

H -4.074255 -1.332096 -0.012199

H -2.828640 -0.428056 -0.807995

H -6.330881 0.492157 -2.960282

H -6.253819 1.881122 0.809278

Pd -0.381594 -1.495030 0.575922

C 1.233546 -3.375549 -0.842250

O 2.174945 -3.913368 -1.438942

O 1.220075 -2.118947 -0.527866

C 0.014261 -4.215025 -0.450928

H -0.448150 -4.597318 -1.370197

H 0.359467 -5.088472 0.114944

N -0.929450 -3.411997 0.328030

C -2.026794 -3.937564 0.823625

O -2.873179 -3.287954 1.528908

O -2.251413 -5.236675 0.560453

C -3.439149 -5.816486 1.123529

H -3.425470 -6.858559 0.802382

H -3.421356 -5.752263 2.214317

H -4.332308 -5.312342 0.746034

H -2.403960 -2.108902 1.654808

H -1.698014 0.662521 4.902777

H -0.813651 -1.219783 3.555044

H 3.418663 -2.744547 -1.762821

O 4.203900 -2.136542 -1.780048

C 4.892852 -2.316792 -0.589441

H 4.391616 -3.014529 0.093920

C 6.280289 -2.912428 -0.880959

C 4.985243 -0.973481 0.149063

F 7.046908 -2.081134 -1.609582

F 6.144134 -4.061029 -1.564263

F 6.950188 -3.193543 0.260597

F 5.732326 -1.056951 1.272062

F 5.516778 -0.001704 -0.619207

F 3.753613 -0.559706 0.523892

93

ts-1aa-p Eopt -3084.446394

C 8.941413 1.541111 0.427290

C 7.957685 0.581444 2.546628

C 7.655505 -1.101656 -2.108691

C 6.764219 1.242721 -2.521582

C -4.776422 3.705163 -0.570251

C -0.890491 7.471740 -0.118824

C 1.441301 -2.546340 0.310989

C 1.887094 -1.942817 1.512767

C 2.412613 -2.705169 -0.710132

C 3.208746 -1.548037 1.689311

C 3.739260 -2.327499 -0.530351

C 2.522040 3.468386 1.029884

C 2.254434 2.058163 -0.901064

C 3.817909 2.990647 1.201691

C 3.546408 1.570805 -0.735560

C -2.101005 2.826214 -0.370361

C -0.089054 4.758598 -0.119095

C -0.449806 1.059488 -0.205442

C 5.617556 -1.413522 0.926242

C 8.111621 0.385437 1.023889

C 6.567701 -0.070344 -1.736945

C 4.167360 -1.746546 0.678569

C 1.704701 2.991744 -0.009320

C -0.742322 2.450072 -0.249917

C 4.330677 2.015209 0.335363

C 0.286367 3.406690 -0.131853

C -2.450050 4.169265 -0.374551

C -1.422431 5.150143 -0.239600

N -0.256125 -0.083630 -0.174551

O -3.705142 4.649325 -0.495229

O -1.849899 6.432044 -0.250959

O 5.592020 1.556815 0.573656

Si 6.431902 0.146907 0.157353

H 9.193172 1.373642 -0.625205

H 8.402971 2.493681 0.495554

H 9.886434 1.657893 0.974358

H 7.462337 -0.266220 3.033284

H 8.941855 0.697213 3.019449

H 7.375222 1.482298 2.770790

H 8.657749 -0.745174 -1.842347

H 7.505709 -2.068339 -1.611919

H 7.655787 -1.289442 -3.190277

H 5.977666 1.973576 -2.307249

H 7.724193 1.715737 -2.288114

H 6.752510 1.048244 -3.602329

H -4.821115 3.084535 0.328060

H -5.685622 4.301278 -0.651781

H -4.681157 3.055364 -1.447464

H -1.459189 8.402246 -0.145909

H -0.351392 7.400628 0.834502

H -0.169999 7.459775 -0.946571

H 1.184090 -1.806242 2.331165

H 2.123823 -3.186241 -1.642210

H 3.518543 -1.106294 2.633797

H 4.464347 -2.518184 -1.318901

H 2.121695 4.189532 1.737619

H 1.664589 1.703206 -1.740557

H 4.438810 3.340023 2.020713

H 3.932592 0.834908 -1.430882

H -2.856997 2.052299 -0.445925

H 0.690236 5.507286 -0.042437

H 6.245798 -2.246675 0.575969

H 5.788681 -1.344741 2.006824

H 8.671468 -0.549570 0.861121

H 5.603385 -0.497800 -2.052757

Pd -0.575210 -2.077658 -0.311881

C -3.232985 -2.605282 -1.206326

O -4.412109 -2.458449 -1.555376

O -2.440797 -1.604928 -0.988077

C -2.654210 -4.012684 -1.034268

H -3.321253 -4.588951 -0.383606

H -2.664066 -4.506423 -2.015663

N -1.300092 -3.942191 -0.484216

C -0.705911 -4.998859 0.031379

O 0.437462 -4.967688 0.591047

O -1.369939 -6.166990 -0.057009

C -0.730922 -7.314493 0.523305

H -1.422414 -8.140778 0.353777

H 0.227088 -7.511870 0.035763

H -0.567266 -7.166852 1.593797

H 0.858410 -3.726281 0.497766

H -4.734413 -0.738434 -1.321950

O -5.120668 0.093209 -0.938573

C -6.003264 -0.316812 0.058133

H -6.292330 -1.369387 -0.052370

C -7.291458 0.505190 -0.058068

C -5.336936 -0.197575 1.438155

F -7.070435 1.829197 0.093722

F -8.198551 0.138366 0.870792

F -7.844874 0.320205 -1.267616

F -4.926743 1.061299 1.705255

F -4.252147 -0.993971 1.487748

F -6.165792 -0.574363 2.433748

95

int10 Eopt -2742.106433

C -3.941758 7.056782 0.122310

C -2.660025 6.924428 2.297433

C -0.933811 6.123281 -2.282040

C -2.928251 4.557731 -2.398290

C -6.153781 -6.090130 -0.809494

C -9.002338 -1.474646 -1.036481

C 1.903443 1.155100 0.541104

C 1.056428 1.369669 1.631009

C 2.102199 2.181501 -0.386374

C 0.429507 2.608671 1.795058

C 1.473690 3.416633 -0.205855

C -5.008797 1.066273 1.009644

C -3.048391 0.497386 -0.266356

C -4.504311 2.341669 1.225825

C -2.529547 1.773011 -0.049550

C -4.810540 -3.667326 -0.227339

C -6.259939 -1.297399 -0.349001

C -2.809516 -2.498714 0.427856

C -0.079572 4.979035 1.065345

C -2.565178 6.790681 0.763241

C -1.626528 4.897880 -1.645610

C 0.625443 3.656118 0.885580

C -4.297240 0.116141 0.252834

C -4.179935 -2.428040 0.049711

C -3.263048 2.713817 0.687388

C -4.895161 -1.213971 -0.012809

C -6.153230 -3.723401 -0.557132

C -6.894148 -2.508021 -0.614692

N -1.698465 -2.659489 0.729783

O -6.849544 -4.851451 -0.839620

O -8.194610 -2.642716 -0.951018

O -2.837769 3.978348 0.933601

Si -1.796829 5.125577 0.241483

H -3.888332 7.109275 -0.970269

H -4.660960 6.271367 0.384381

H -4.355317 8.010940 0.475970

H -1.681806 6.850017 2.785334

H -3.089287 7.896871 2.573606

H -3.304947 6.146497 2.722638

H -1.560895 7.021173 -2.216683

H 0.027618 6.356385 -1.808170

H -0.735658 5.943614 -3.346785

H -3.407359 3.655025 -2.006820

H -3.657992 5.373163 -2.342228

H -2.717608 4.384599 -3.462277

H -5.753056 -6.300702 0.190674

H -6.891000 -6.850164 -1.071710

H -5.335239 -6.107689 -1.540802

H -9.998421 -1.826000 -1.308487

H -9.049033 -0.950372 -0.073852

H -8.632365 -0.789074 -1.808893

H 0.860717 0.580553 2.346909

H 2.737600 2.036455 -1.252060

H -0.225991 2.756265 2.650812

H 1.655727 4.207412 -0.931299

H -5.963214 0.793367 1.451692

H -2.473781 -0.195243 -0.873822

H -5.055086 3.068763 1.814304

H -1.556443 2.031457 -0.452640

H -4.220739 -4.573254 -0.164836

H -6.818262 -0.373590 -0.431887

H 0.538989 5.798867 0.673687

H -0.216330 5.184448 2.134186

H -1.870517 7.566593 0.403106

H -0.944393 4.043132 -1.768373

C 3.082029 -1.504622 4.418240

H 3.598228 -2.471316 4.454973

H 2.168139 -1.581004 5.012148

C 2.740179 -1.195817 2.980858

O 3.605879 -0.529149 2.296508

O 1.663687 -1.583089 2.462023

Pd 2.768144 -0.686533 0.392082

C 3.390639 -3.846015 0.359159

O 3.768955 -2.638856 0.267756

O 2.220440 -4.277369 0.560985

C 4.481725 -4.894670 0.171318

H 4.192876 -5.839785 0.635967

H 5.430563 -4.538616 0.579814

H 4.621302 -5.065810 -0.903021

Ag 0.428702 -3.065502 0.690732

H 3.748514 -0.744728 4.832770

C 4.432952 0.060317 -0.467291

C 4.936503 -0.518157 -1.630210

C 5.120133 1.057205 0.229088

C 6.160214 -0.055176 -2.120072

H 4.408156 -1.314694 -2.135620

C 6.339324 1.504035 -0.275466

H 4.723604 1.467539 1.149334

C 6.864046 0.956289 -1.455468

H 6.570664 -0.492353 -3.028484

H 6.901163 2.278947 0.237898

I 1.334833 -1.080359 -1.855624

C 8.155133 1.433450 -1.993603

O 8.838144 2.307860 -1.493235

H 8.482584 0.923572 -2.927762

89

int7 Eopt -2614.864308

C -0.702105 7.270465 0.442481

C -1.139326 6.724105 -1.987121

C -3.062955 4.597150 2.005553

C -0.632323 4.241991 2.639253

C 7.452552 -3.796963 -0.201664

C 8.388061 1.492238 0.586004

C -4.208616 -0.280815 -1.321167

C -2.884269 -0.203349 -1.808617

C -4.802450 0.881087 -0.773367

C -2.198325 1.030492 -1.787806

C -4.120971 2.081817 -0.785384

C 3.581285 2.559171 -0.917881

C 2.057971 1.088535 0.226533

C 2.605019 3.546508 -0.966422

C 1.067854 2.067043 0.166055

C 5.260000 -2.017142 -0.378467

C 5.730171 0.693083 0.024522

C 2.906955 -1.619787 -0.765748

C -2.199779 3.555375 -1.542784

C -1.482116 6.360657 -0.527277

C -1.718063 3.969353 1.578482

C -2.816456 2.192700 -1.333529

C 3.334202 1.309388 -0.316779

C 4.192979 -1.089833 -0.453855

C 1.335889 3.310203 -0.417988

C 4.409092 0.289722 -0.249684

C 6.551634 -1.596071 -0.111080

C 6.789292 -0.207642 0.093489

N 1.878393 -2.105206 -1.013494

O 7.639904 -2.400715 -0.019337

O 8.072237 0.124503 0.362063

O 0.433374 4.325278 -0.472209

Si -1.227495 4.504967 -0.182992

H -1.010609 7.129837 1.484113

H 0.375443 7.077614 0.384472

H -0.862073 8.328258 0.193603

H -1.734706 6.157565 -2.712392

H -1.325549 7.790237 -2.173303

H -0.081803 6.531661 -2.201680

H -2.984221 5.685814 2.112762

H -3.869832 4.391495 1.292409

H -3.381777 4.196347 2.976397

H 0.319440 3.762328 2.389486

H -0.440347 5.314557 2.756579

H -0.950156 3.859965 3.618290

H 7.069955 -4.022969 -1.205623

H 8.439137 -4.246587 -0.080415

H 6.767667 -4.211369 0.549623

H 9.462716 1.519505 0.771440

H 8.152938 2.106412 -0.292518

H 7.856141 1.888049 1.460330

H -2.437489 -1.043932 -2.330575

H -5.819397 0.834688 -0.394222

H -1.200312 1.081808 -2.215275

H -4.616587 2.978538 -0.420941

H 4.547343 2.752301 -1.376406

H 1.810564 0.161667 0.731717

H 2.802544 4.506824 -1.432491

H 0.092225 1.851679 0.586687

H 5.045122 -3.065371 -0.544352

H 5.910880 1.742799 0.218300

H -2.999669 4.247392 -1.842688

H -1.513804 3.514977 -2.398905

H -2.557447 6.550365 -0.378044

H -1.874404 2.881550 1.526141

Pd -3.038732 -1.657070 0.237314

C -2.106904 -3.979609 1.843300

O -2.918521 -2.977031 1.800238

Ag -0.252646 -2.565354 -0.321720

C -2.892662 -3.684446 -1.846482

O -3.690828 -3.176269 -0.961949

O -1.740225 -3.288666 -2.105655

C -3.469804 -4.878669 -2.590416

H -3.006874 -4.963808 -3.576030

H -3.235222 -5.786074 -2.021056

H -4.556372 -4.805776 -2.679048

C -1.294679 -0.227191 1.983155

O -2.519255 -0.217320 1.566908

O -0.333976 -0.801915 1.437830

C -1.072572 0.527386 3.286975

H -1.106967 -0.201118 4.106076

H -0.085245 0.995527 3.294969

H -1.853155 1.270589 3.462338

H -4.821117 -1.151942 -1.528606

C -2.426592 -4.961953 2.964984

H -2.739074 -4.430794 3.867737

H -3.260423 -5.599496 2.648792

H -1.558380 -5.590627 3.171894

O -1.151315 -4.212449 1.081802

97

int8 Eopt -2898.059931

C -4.441657 6.367684 -1.189716

C -4.240482 6.525466 1.324903

C -0.625358 5.670160 -1.928652

C -2.209491 3.862042 -2.744977

C -4.322933 -7.203380 -0.008910

C -7.982945 -3.557113 -1.672824

C 1.191435 1.348059 2.500804

C -0.061848 1.501125 3.102248

C 1.634509 2.295181 1.570228

C -0.862251 2.599764 2.787153

C 0.827712 3.393528 1.265241

C -5.147585 0.210585 0.449215

C -2.873366 -0.065129 -0.290460

C -4.962088 1.585123 0.525936

C -2.673770 1.310803 -0.200940

C -3.686687 -4.459821 0.267097

C -5.554205 -2.584774 -0.589164

C -2.169195 -2.714369 0.978007

C -1.283674 4.778301 1.565022

C -3.471456 6.299721 0.006626

C -1.415845 4.401790 -1.538596

C -0.427518 3.570236 1.870486

C -4.109187 -0.646779 0.038944

C -3.411469 -3.075329 0.380069

C -3.720787 2.152162 0.199391

C -4.342694 -2.107616 -0.053528

C -4.885470 -4.905037 -0.263194

C -5.840827 -3.942783 -0.697727

N -1.140753 -2.471735 1.468226

O -5.246787 -6.204018 -0.414653

O -6.981229 -4.454762 -1.211846

O -3.619029 3.502562 0.284757

Si -2.438249 4.697401 0.046583

H -3.917658 6.351331 -2.151288

H -5.145163 5.526714 -1.178249

H -5.033702 7.292384 -1.155758

H -3.573872 6.595302 2.191650

H -4.816401 7.459652 1.280019

H -4.949099 5.710379 1.513201

H -1.293705 6.483483 -2.237608

H -0.003426 6.045927 -1.107027

H 0.045002 5.461968 -2.772646

H -2.746560 2.937993 -2.507279

H -2.946634 4.588071 -3.105559

H -1.531750 3.646928 -3.582293

H -4.107230 -7.138492 1.065715

H -4.805211 -8.157980 -0.224260

H -3.383335 -7.133861 -0.572612

H -8.800093 -4.186061 -2.028666

H -8.344454 -2.911068 -0.862886

H -7.613936 -2.935378 -2.498145

H -0.409891 0.771617 3.829478

H 2.602827 2.187217 1.087742

H -1.830071 2.717387 3.269721

H 1.193319 4.139117 0.562014

H -6.107776 -0.208960 0.737547

H -2.047853 -0.673507 -0.645069

H -5.764888 2.240943 0.848159

H -1.697807 1.719668 -0.437878

H -2.942442 -5.164956 0.615601

H -6.268234 -1.857573 -0.954737

H -0.645541 5.662091 1.418366

H -1.918726 5.006287 2.430050

H -2.745875 7.121924 -0.101139

H -0.673921 3.637331 -1.262283

Pd 3.351680 -0.792390 -0.206867

C 5.880600 -2.295964 -1.817998

O 7.003961 -3.047720 -1.959541

O 4.814521 -2.631277 -2.281106

C 6.185740 -1.061006 -0.990512

H 6.470523 -1.411920 0.014336

H 7.069769 -0.574563 -1.414073

N 5.086974 -0.125649 -0.954463

C 5.306232 1.200601 -0.737600

O 4.474050 2.014475 -0.354195

O 6.590665 1.575107 -1.048872

C 6.841077 2.978960 -0.952621

H 7.893272 3.105015 -1.215841

H 6.207381 3.538116 -1.647535

H 6.655974 3.342064 0.062206

Ag 1.000656 -1.566696 0.885453

C 3.689808 -2.055312 2.366439

O 4.362240 -1.543524 1.412585

O 2.429590 -2.170294 2.410839

C 4.475591 -2.544057 3.566025

H 4.356781 -1.823680 4.383678

H 4.075019 -3.502075 3.907995

H 5.536535 -2.636723 3.327378

C 1.119548 -0.254453 -1.935056

O 2.367753 -0.058500 -1.852557

O 0.387783 -0.822018 -1.060316

C 0.440843 0.202334 -3.210645

H 0.278952 -0.669682 -3.855180

H -0.536818 0.637102 -2.985739

H 1.066973 0.920484 -3.742927

H 6.734128 -3.834605 -2.469485

H 1.829455 0.512842 2.777358

81

int9 Eopt -2385.741161

C -6.903396 -3.194656 -0.005675

C -5.705432 -4.106467 -2.036847

C -4.215536 -4.274385 2.676104

C -4.655505 -1.775895 2.574052

C 0.729648 7.934588 0.208002

C -4.575355 6.787495 0.299412

C 1.450148 -2.728448 0.095859

C 0.788302 -2.448624 -1.105947

C 0.815691 -3.526552 1.057273

C -0.511575 -2.917582 -1.309776

C -0.486399 -3.991738 0.841751

C -3.508681 1.929524 -1.156358

C -1.810487 1.103714 0.334198

C -4.039807 0.650855 -1.266641

C -2.329375 -0.184760 0.224287

C -0.051780 5.232392 -0.101865

C -2.768032 4.634362 -0.046038

C 0.518566 2.915025 -0.478559

C -2.574740 -4.204041 -0.593665

C -5.650701 -3.942929 -0.503506

C -3.866473 -2.950139 1.961735

C -1.172933 -3.695790 -0.346012

C -2.383612 2.187510 -0.351206

C -0.491597 3.894199 -0.249521

C -3.459553 -0.420708 -0.570974

C -1.863449 3.569270 -0.216272

C -0.959785 6.263938 0.066482

C -2.349960 5.955533 0.089705

N 1.393531 2.172855 -0.667735

O -0.644828 7.574114 0.215936

O -3.171788 7.013630 0.264042

O -4.035549 -1.639771 -0.721660

Si -4.017717 -3.142223 0.067847

H -6.971596 -3.178497 1.087376

H -6.910554 -2.155684 -0.356280

H -7.816310 -3.674906 -0.383140

H -4.889572 -4.728991 -2.420641

H -6.648394 -4.580757 -2.340267

H -5.648623 -3.134168 -2.540127

H -5.275615 -4.530218 2.557029

H -3.627433 -5.121573 2.302131

H -4.021029 -4.194426 3.753725

H -4.376847 -0.815509 2.129043

H -5.736682 -1.899704 2.446561

H -4.462651 -1.708705 3.653403

H 1.205372 7.680464 -0.748187

H 0.752775 9.016194 0.348009

H 1.276294 7.448626 1.026610

H -5.028055 7.770587 0.434999

H -4.932811 6.346246 -0.639457

H -4.854676 6.137443 1.138017

H 1.274531 -1.857458 -1.875650

H 1.330208 -3.803680 1.977755

H -1.020054 -2.680952 -2.242414

H -0.957333 -4.620736 1.595162

H -3.959898 2.740477 -1.721701

H -0.956921 1.264647 0.985250

H -4.903945 0.456247 -1.894072

H -1.852512 -1.003610 0.751959

H 1.011554 5.434046 -0.135647

H -3.823537 4.399434 0.009777

H -2.697934 -5.205288 -0.155494

H -2.736940 -4.325701 -1.671703

H -5.662254 -4.951517 -0.059853

H -2.798368 -2.758663 2.145096

C 4.851384 -2.485140 -3.286474

H 5.826195 -1.992135 -3.374761

H 4.297306 -2.293165 -4.209332

C 4.102436 -1.872306 -2.109354

O 4.060758 -2.618565 -1.064915

O 3.615980 -0.728124 -2.242686

Pd 3.163644 -1.967545 0.669839

C 5.185134 0.073282 1.769469

O 4.684216 1.015436 1.100144

O 4.814377 -1.146742 1.763222

C 6.368590 0.396188 2.673101

H 6.232967 -0.073315 3.652019

H 6.493711 1.474861 2.783960

H 7.279085 -0.026965 2.232923

Ag 3.108755 0.710336 -0.500298

H 5.004981 -3.556612 -3.149481

89

ts4-m Eopt -2614.836382

C 0.380507 7.256573 0.874895

C -0.124102 6.969650 -1.583407

C -2.308226 4.839470 2.340417

C 0.089093 4.171604 2.844295

C 6.783213 -4.833726 -0.314648

C 8.503354 0.288004 0.207630

C -1.780960 -0.199778 -2.269557

C -0.890541 0.706295 -2.842071

C -2.713776 0.221383 -1.293755

C -0.933065 2.049778 -2.469906

C -2.716989 1.585188 -0.925657

C 3.782853 1.981017 -0.989042

C 2.176834 0.782607 0.344061

C 2.947050 3.090781 -1.010584

C 1.327470 1.885488 0.315186

C 4.852157 -2.766488 -0.401341

C 5.724277 -0.140039 -0.128465

C 2.556640 -2.049769 -0.634338

C -1.911463 3.988709 -1.172576

C -0.534558 6.561414 -0.153314

C -1.059168 4.095491 1.818387

C -1.853962 2.516919 -1.514795

C 3.419046 0.800917 -0.312121

C 3.921722 -1.698492 -0.431704

C 1.706138 3.054265 -0.357140

C 4.342648 -0.358278 -0.289897

C 6.205911 -2.526083 -0.242582

C 6.649632 -1.179850 -0.105732

N 1.457316 -2.399177 -0.796969

O 7.175803 -3.473347 -0.200630

O 7.982349 -1.026453 0.056019

O 0.943628 4.177866 -0.413789

Si -0.610724 4.667771 0.054432

H 0.038384 7.107219 1.904337

H 1.409738 6.884246 0.806857

H 0.411145 8.339658 0.694546

H -0.804268 6.573379 -2.345806

H -0.121775 8.063057 -1.685853

H 0.884945 6.612905 -1.819714

H -2.109248 5.907597 2.491748

H -3.163416 4.753650 1.659259

H -2.623989 4.428478 3.308168

H 0.972745 3.612897 2.519309

H 0.401475 5.205546 3.027588

H -0.230926 3.754198 3.808243

H 6.297564 -5.031976 -1.279170

H 7.704156 -5.414594 -0.246891

H 6.106813 -5.125465 0.499388

H 9.582259 0.166176 0.312416

H 8.288755 0.907093 -0.672540

H 8.101590 0.776465 1.104154

H -0.174266 0.374611 -3.589268

H -0.251546 2.756806 -2.937632

H -3.438951 1.925356 -0.186694

H 4.724234 2.020732 -1.530551

H 1.850755 -0.084457 0.908293

H 3.231445 3.994733 -1.540244

H 0.367758 1.819195 0.812876

H 4.481739 -3.777605 -0.515287

H 6.063408 0.878096 0.014585

H -2.896888 4.236020 -0.754373

H -1.820442 4.577380 -2.094323

H -1.562971 6.920341 0.014512

H -1.342584 3.036913 1.712198

Pd -3.191396 -1.304060 0.141258

C -3.002710 -3.907045 1.569227

O -3.620548 -2.788862 1.539790

Ag -0.545827 -2.632280 0.182629

C -4.879536 -1.978920 -2.087013

O -4.996619 -0.724535 -2.236024

C -1.398997 -0.218319 2.214408

O -2.556433 -0.110691 1.665453

O -0.424183 -0.878795 1.797865

C -1.260749 0.537636 3.530993

H -1.460040 -0.164425 4.349537

H -0.240246 0.909998 3.649604

H -1.980826 1.355578 3.602211

H -3.935382 -0.215096 -1.604125

C -3.673900 -4.992135 2.400251

H -4.256365 -4.554530 3.214136

H -4.359596 -5.553065 1.753909

H -2.927145 -5.686784 2.791401

O -1.934108 -4.198886 0.975474

H -1.781550 -1.235881 -2.599151

C -5.767403 -2.892134 -2.902669

H -5.995770 -2.436674 -3.868503

H -5.294515 -3.867295 -3.033889

H -6.709213 -3.036422 -2.360214

O -4.067468 -2.528488 -1.274701

89

ts4-o Eopt -2614.827406

C -2.222230 6.570221 0.322100

C -2.609670 5.438927 -1.905253

C -3.610020 3.752696 2.607437

C -1.142499 4.220413 3.025900

C 7.811579 -3.073721 -0.410065

C 8.115407 2.299268 0.297451

C -0.503576 -0.222006 -3.423226

C -0.391720 1.170522 -3.409560

C -1.367245 -0.832415 -2.521444

C -1.119481 1.926792 -2.493076

C -2.106560 -0.091329 -1.561523

C 3.060579 2.697995 -0.740702

C 1.897067 1.055530 0.579329

C 1.936284 3.515930 -0.728851

C 0.764423 1.864039 0.587243

C 5.396546 -1.598727 -0.371371

C 5.542987 1.154055 -0.002672

C 2.988035 -1.519246 -0.536613

C -2.766426 2.179548 -0.587648

C -2.748935 5.300694 -0.376006

C -2.162143 3.456844 2.160261

C -1.955128 1.324631 -1.538853

C 3.063784 1.443907 -0.099457

C 4.213497 -0.819508 -0.338613

C 0.767587 3.095145 -0.077497

C 4.265985 0.579112 -0.145657

C 6.640780 -1.009736 -0.227964

C 6.712836 0.400432 -0.042660

N 2.023467 -2.150978 -0.701843

O 7.829487 -1.663109 -0.245169

O 7.960316 0.899566 0.099297

O -0.320204 3.907515 -0.134610

Si -1.959385 3.695514 0.279599

H -2.418836 6.563419 1.399611

H -1.140331 6.678550 0.181372

H -2.700223 7.468206 -0.092566

H -3.026239 4.580073 -2.444400

H -3.133609 6.335353 -2.263150

H -1.556764 5.537648 -2.195953

H -3.862256 4.814411 2.494731

H -4.347719 3.172787 2.038624

H -3.746536 3.497317 3.666590

H -0.109930 3.976947 2.752584

H -1.258329 5.306591 2.934040

H -1.274288 3.969527 4.087364

H 7.368569 -3.359840 -1.373097

H 8.855822 -3.388435 -0.384312

H 7.261915 -3.565672 0.403075

H 9.190281 2.467606 0.375844

H 7.716477 2.869646 -0.550776

H 7.625057 2.628851 1.221922

H 0.258042 1.673312 -4.121698

H -1.509680 -1.909166 -2.572259

H -1.023462 3.007646 -2.512761

H -3.408172 -0.380432 -1.660925

H 3.940721 3.021485 -1.290213

H 1.857307 0.117376 1.122739

H 1.934402 4.471362 -1.244426

H -0.135837 1.516109 1.077979

H 5.307435 -2.667346 -0.521932

H 5.601097 2.220193 0.176820

H -3.180826 1.541539 0.200341

H -3.631232 2.582036 -1.139036

H -3.823174 5.209500 -0.146676

H -1.998776 2.381440 2.315971

Pd -2.557340 -1.414439 0.090716

C -2.392519 -3.989563 1.522233

O -2.943309 -2.836505 1.567511

Ag 0.083953 -2.838033 0.115199

C -4.537176 -2.064742 -1.893882

O -4.590583 -0.811549 -2.089323

C -0.840515 -0.625840 2.358695

O -1.771772 -0.214395 1.559461

O 0.011088 -1.501032 2.122226

C -0.783685 0.109307 3.694105

H -1.781692 0.412123 4.019126

H -0.318703 -0.533016 4.445152

H -0.167135 1.010185 3.589373

C -3.026163 -5.048262 2.413149

H -3.585990 -4.589505 3.230746

H -3.720510 -5.644042 1.808633

H -2.257368 -5.719844 2.803177

O -1.415100 -4.333504 0.808966

H 0.056717 -0.815880 -4.140258

O -3.654762 -2.634179 -1.174134

C -5.586995 -2.945856 -2.532201

H -5.186286 -3.945435 -2.712303

H -6.432600 -3.036103 -1.840139

H -5.947836 -2.497906 -3.460175

89

ts4 Eopt -2614.839321

C 2.045507 7.222578 0.449307

C 1.129041 7.016647 -1.899865

C -1.081901 5.580150 2.242475

C 1.105441 4.367660 2.680254

C 5.813988 -5.857877 -0.396625

C 8.447256 -1.176558 0.386305

C -3.202727 0.729477 -0.796375

C -2.051826 0.811690 -1.617210

C -3.591112 1.914397 -0.126171

C -1.340505 1.998412 -1.765960

C -2.891408 3.104319 -0.284281

C 4.205604 1.406253 -1.052851

C 2.344088 0.531390 0.198877

C 3.598898 2.654889 -1.094526

C 1.722298 1.777634 0.151407

C 4.322748 -3.456299 -0.524128

C 5.663434 -1.052612 -0.118706

C 2.225970 -2.298326 -0.861194

C -1.013823 4.469484 -1.299585

C 0.858637 6.737607 -0.406799

C -0.080306 4.532647 1.708754

C -1.750987 3.170733 -1.107047

C 3.593748 0.315775 -0.405640

C 3.618269 -2.228477 -0.571770

C 2.348753 2.853740 -0.489893

C 4.278283 -0.998695 -0.363536

C 5.685342 -3.484276 -0.281559

C 6.369381 -2.252044 -0.076950

N 1.089599 -2.411891 -1.093747

O 6.451664 -4.601338 -0.215731

O 7.694460 -2.362232 0.163836

O 1.823140 4.104699 -0.562749

Si 0.411116 4.912622 -0.094621

H 1.836997 7.158808 1.522814

H 2.946107 6.630637 0.248610

H 2.282265 8.271090 0.223458

H 0.265182 6.782242 -2.532120

H 1.370574 8.076468 -2.056506

H 1.979521 6.428252 -2.263926

H -0.617492 6.569259 2.338302

H -1.961694 5.688811 1.596513

H -1.444498 5.293893 3.238299

H 1.803105 3.590716 2.351873

H 1.674002 5.298020 2.790882

H 0.743771 4.088269 3.678928

H 5.360032 -5.937857 -1.393069

H 6.601000 -6.606630 -0.296128

H 5.045311 -6.031186 0.367777

H 9.474246 -1.506568 0.548334

H 8.410224 -0.510058 -0.484587

H 8.091881 -0.637776 1.273563

H -1.731837 -0.065568 -2.175164

H -4.472559 1.899246 0.509930

H -0.467475 2.028019 -2.413750

H -3.240567 4.005213 0.216257

H 5.159539 1.266214 -1.554206

H 1.835446 -0.259737 0.739063

H 4.071787 3.490676 -1.600707

H 0.748183 1.892722 0.613519

H 3.773318 -4.374225 -0.691698

H 6.181450 -0.121436 0.073242

H -1.721660 5.308731 -1.242289

H -0.576774 4.507007 -2.305392

H -0.029344 7.320315 -0.113757

H -0.612771 3.570392 1.669265

Pd -3.513892 -1.111189 0.267223

C -3.268795 -3.908010 1.322662

O -3.892592 -2.799927 1.433031

Ag -0.893599 -2.429780 0.015965

C -5.072320 -1.545624 -2.168223

O -4.414114 -2.152766 -1.266667

O -5.110911 -0.285441 -2.304586

C -5.882218 -2.395746 -3.123243

H -6.910693 -2.457691 -2.748573

H -5.909005 -1.932138 -4.111854

H -5.472126 -3.405693 -3.178495

C -1.495926 -0.121777 2.176231

O -2.749279 -0.166876 1.905505

O -0.562457 -0.638603 1.521814

C -1.171348 0.637579 3.457482

H -1.454573 0.020139 4.317738

H -0.102509 0.853163 3.514116

H -1.752249 1.562512 3.513028

H -4.214879 0.196393 -1.462044

C -3.933098 -5.090639 2.015681

H -4.479266 -4.762511 2.903119

H -4.653110 -5.542254 1.322769

H -3.188550 -5.845025 2.279166

O -2.199314 -4.119586 0.696572

97

ts4a Eopt -2898.053714

C 3.916396 6.747295 -0.177200

C 2.478166 6.712658 -2.256876

C 1.043868 5.860129 2.363145

C 2.953403 4.188000 2.367395

C 4.994282 -6.740123 -0.238379

C 8.444166 -2.576458 0.228974

C -2.790601 1.553446 -0.012460

C -1.981115 1.486295 -1.173029

C -2.604620 2.673711 0.832742

C -1.059121 2.485347 -1.476927

C -1.702335 3.682308 0.516489

C 4.672427 0.644648 -1.335138

C 2.753226 0.179217 0.043732

C 4.284728 1.975191 -1.434183

C 2.349410 1.508719 -0.060578

C 3.959153 -4.120877 -0.506506

C 5.719215 -1.980264 -0.255165

C 2.103075 -2.614536 -0.913849

C 0.019390 4.735000 -1.018727

C 2.493269 6.523081 -0.725800

C 1.636437 4.606229 1.683164

C -0.913213 3.611086 -0.647698

C 3.916127 -0.282596 -0.594440

C 3.487055 -2.791604 -0.622661

C 3.118890 2.420174 -0.793748

C 4.361572 -1.692962 -0.489722

C 5.299036 -4.380134 -0.271574

C 6.198214 -3.283314 -0.148422

N 0.967350 -2.510958 -1.152851

O 5.852406 -5.612772 -0.143106

O 7.487455 -3.617974 0.086141

O 2.802149 3.735733 -0.933737

Si 1.752222 4.845350 -0.206904

H 3.944318 6.748559 0.917844

H 4.602863 5.967550 -0.527492

H 4.314165 7.713485 -0.516073

H 1.465439 6.671517 -2.673364

H 2.903478 7.688112 -2.528806

H 3.076836 5.941887 -2.756381

H 1.724414 6.717246 2.290068

H 0.084082 6.164097 1.927251

H 0.870892 5.673918 3.431189

H 3.355601 3.259211 1.951430

H 3.726597 4.958724 2.271275

H 2.789148 4.026095 3.441314

H 4.514386 -6.797844 -1.224309

H 5.633807 -7.612497 -0.095776

H 4.220987 -6.723073 0.540798

H 9.399038 -3.075401 0.400341

H 8.509031 -1.964923 -0.679920

H 8.209609 -1.931961 1.085518

H -2.104427 0.649066 -1.855859

H -3.207655 2.763850 1.732468

H -0.460764 2.410365 -2.382348

H -1.623885 4.555146 1.161503

H 5.563777 0.312412 -1.860912

H 2.145099 -0.479757 0.653756

H 4.866388 2.686839 -2.011867

H 1.430979 1.814529 0.429371

H 3.250814 -4.932587 -0.615555

H 6.398920 -1.147417 -0.124652

H -0.463206 5.698689 -0.797875

H 0.197126 4.727009 -2.101205

H 1.837674 7.292536 -0.287534

H 0.916024 3.787605 1.829906

Pd -3.334718 -0.318822 0.871633

C -3.191768 -3.190085 1.789588

O -3.834123 -2.087137 1.886753

Ag -0.963853 -1.820602 -0.057103

C -5.408155 0.159921 -1.150966

O -5.278642 1.387992 -0.838563

C -0.871952 0.138979 2.466613

O -2.152496 0.193242 2.474127

O -0.141753 -0.320469 1.558315

C -0.217846 0.734968 3.707836

H 0.827874 0.428730 3.775491

H -0.266457 1.828800 3.646555

H -0.761277 0.432518 4.607241

H -4.086981 1.446220 -0.306594

C -3.640727 -4.283103 2.753336

H -4.668176 -4.121170 3.086185

H -3.537992 -5.265160 2.284753

H -2.986608 -4.260128 3.633313

O -2.249482 -3.447063 1.002549

N -4.648632 -0.797503 -0.652468

C -4.806501 -2.160312 -1.105904

H -4.911441 -2.828157 -0.245278

H -5.705588 -2.284157 -1.717081

C -3.619533 -2.650885 -1.918598

O -2.607691 -2.027860 -2.180356

O -3.817869 -3.916070 -2.339839

H -3.004613 -4.179257 -2.811583

O -6.361710 -0.185578 -2.042626

C -7.154494 0.883536 -2.579189

H -6.525347 1.611754 -3.096857

H -7.839765 0.404639 -3.280217

H -7.712266 1.388318 -1.786323

89

ts4b Eopt -2668.952605

C 2.399623 6.396040 -0.513016

C 0.329047 7.062160 -1.798296

C -0.669148 5.313999 2.598685

C 1.577839 4.185105 2.352079

C 7.247976 -4.965429 0.031952

C 8.613789 0.232106 -0.741034

C -3.637097 0.828122 -0.205685

C -2.837463 1.138160 -1.334603

C -3.826724 1.860656 0.740775

C -2.240427 2.382899 -1.493276

C -3.249021 3.115931 0.574402

C 3.716181 1.411349 -1.621134

C 2.321668 0.440457 0.083676

C 2.841319 2.485369 -1.736098

C 1.429684 1.504337 -0.038980

C 5.166410 -3.064584 -0.223883

C 5.856026 -0.402266 -0.618253

C 2.814693 -2.545178 -0.296122

C -1.738602 4.723138 -0.693999

C 0.857958 6.446663 -0.486890

C 0.158359 4.312233 1.763106

C -2.427235 3.393712 -0.533605

C 3.487608 0.371401 -0.698887

C 4.157805 -2.077785 -0.355868

C 1.696689 2.552375 -0.928815

C 4.484558 -0.718431 -0.554859

C 6.506669 -2.727753 -0.295137

C 6.856394 -1.362670 -0.498509

N 1.745371 -3.000629 -0.231099

O 7.544852 -3.592771 -0.183204

O 8.182584 -1.108589 -0.543862

O 0.862965 3.617313 -1.081606

Si 0.077220 4.746175 -0.093611

H 2.825944 6.027976 0.426015

H 2.757060 5.740757 -1.315623

H 2.815545 7.396251 -0.694551

H -0.753830 7.226376 -1.775198

H 0.803984 8.034177 -1.987930

H 0.551004 6.419775 -2.659918

H -0.245520 6.325450 2.552716

H -1.712859 5.375102 2.271577

H -0.676093 5.016196 3.655339

H 2.200533 3.476032 1.797902

H 2.094219 5.152546 2.362567

H 1.528709 3.837404 3.392750

H 6.671084 -5.386603 -0.801750

H 8.212729 -5.470521 0.096587

H 6.694758 -5.113889 0.968511

H 9.703742 0.191239 -0.753700

H 8.251419 0.632086 -1.696377

H 8.281744 0.883496 0.077035

H -2.694766 0.383175 -2.104483

H -4.432945 1.669338 1.622386

H -1.621162 2.584071 -2.363828

H -3.430857 3.895003 1.312105

H 4.583977 1.369022 -2.273632

H 2.089771 -0.319789 0.821532

H 3.025057 3.278658 -2.454084

H 0.527155 1.509573 0.561223

H 4.864491 -4.093879 -0.075928

H 6.130963 0.638314 -0.733266

H -2.286513 5.515412 -0.167324

H -1.716995 5.007704 -1.753234

H 0.557556 7.113379 0.337376

H -0.332660 3.331914 1.853413

Pd -3.240269 -1.149621 0.473859

C -1.052521 -0.357625 2.450441

O -2.276799 -0.289362 2.090328

Ag -0.290499 -2.937088 0.596271

C -5.522958 -1.549969 -1.321714

O -5.781857 -0.301735 -1.222421

H -4.787631 0.220973 -0.627967

C -0.743780 0.368643 3.757743

H -0.980703 -0.298103 4.595741

H 0.317572 0.622086 3.811472

H -1.359841 1.265537 3.861661

O -0.117719 -0.955413 1.866842

N -4.445075 -2.118511 -0.828782

C -4.264829 -3.565901 -0.892730

H -5.112804 -4.094326 -0.434243

H -4.190499 -3.920096 -1.926450

C -2.987797 -3.989525 -0.157203

O -2.530412 -3.117426 0.732799

O -2.451383 -5.059216 -0.389524

O -6.390163 -2.348601 -1.973129

C -7.558265 -1.720540 -2.522445

H -7.280684 -0.961506 -3.258258

H -8.116716 -2.527096 -2.999473

H -8.156390 -1.256748 -1.733954

101

ts4c Eopt -3458.758794

C -8.163154 1.068474 1.065169

C -8.366489 -0.431951 -0.958636

C -5.855018 -1.844190 3.026972

C -5.046792 0.557945 3.028194

C 3.334513 7.085292 -1.271027

C -1.872405 8.495100 -0.663796

C -1.373102 -3.453462 -0.695522

C -1.871993 -2.410168 -1.517204

C -2.299490 -4.075820 0.174619

C -3.201634 -2.010683 -1.469426

C -3.633081 -3.684560 0.218789

C -3.262952 3.574133 -1.398432

C -1.927953 2.204699 0.062294

C -4.318836 2.679071 -1.281869

C -2.976263 1.292355 0.172920

C 1.407503 5.019202 -1.108902

C -1.262787 5.731776 -0.786795

C 0.841165 2.670516 -1.117535

C -5.541218 -2.188502 -0.533118

C -7.767689 -0.292093 0.456480

C -5.129152 -0.748912 2.214577

C -4.107097 -2.637333 -0.593242

C -2.044139 3.361861 -0.726355

C 0.406925 4.020897 -1.012226

C -4.189133 1.530072 -0.487073

C -0.952673 4.359910 -0.841224

C 1.077689 6.362172 -1.051092

C -0.290778 6.723769 -0.890075

N 1.253582 1.583655 -1.203689

O 1.953588 7.393064 -1.132311

O -0.531812 8.051337 -0.833552

O -5.265404 0.704434 -0.420086

Si -5.884694 -0.592880 0.470769

H -7.865365 1.159076 2.114918

H -7.700747 1.896709 0.515520

H -9.251395 1.209502 1.019956

H -8.210424 -1.430155 -1.382396

H -9.449602 -0.251944 -0.937508

H -7.927175 0.297860 -1.649507

H -6.892327 -1.562233 3.246592

H -5.874675 -2.809426 2.507433

H -5.352371 -2.004204 3.989598

H -4.527724 1.355695 2.487629

H -6.042081 0.929820 3.296569

H -4.503498 0.387407 3.967261

H 3.530996 6.534395 -2.199987

H 3.849782 8.046180 -1.303774

H 3.702489 6.501944 -0.417190

H -1.822081 9.584646 -0.654859

H -2.510613 8.164942 -1.493013

H -2.293514 8.140435 0.285170

H -1.201121 -1.925587 -2.222878

H -1.962410 -4.881460 0.822026

H -3.553165 -1.211381 -2.117329

H -4.324902 -4.196817 0.884351

H -3.377009 4.443478 -2.040373

H -1.024896 2.002078 0.628364

H -5.254269 2.846957 -1.806470

H -2.834400 0.402158 0.774514

H 2.436775 4.708741 -1.238160

H -2.295980 6.012402 -0.625628

H -6.176408 -2.982384 -0.117905

H -5.913103 -1.989172 -1.546100

H -8.207085 -1.084086 1.083810

H -4.100642 -1.103476 2.047609

Pd 0.577817 -3.062874 0.062840

C -0.465818 -1.327166 2.302839

O -0.331378 -2.507637 1.832754

Ag 1.460647 -0.234159 0.088578

C 1.145999 -4.707446 -2.314387

O -0.088748 -5.024686 -2.366009

H -0.687259 -4.310431 -1.471710

C -1.100688 -1.260527 3.685769

H -0.307387 -1.357816 4.436570

H -1.591253 -0.295517 3.833122

H -1.807193 -2.080712 3.833535

O -0.113308 -0.250439 1.760162

N 1.649599 -3.839562 -1.462174

C 3.088509 -3.601122 -1.399190

H 3.651368 -4.541435 -1.361645

H 3.447828 -3.058592 -2.283840

C 3.464129 -2.775988 -0.165229

O 2.491498 -2.378559 0.616426

O 4.651503 -2.515253 0.034931

O 2.008502 -5.278803 -3.176645

C 1.461125 -6.233928 -4.098653

H 0.705643 -5.767791 -4.736068

H 2.309537 -6.570036 -4.696083

H 1.013257 -7.075608 -3.564279

H 5.059062 -1.569517 1.408032

O 5.301566 -1.031763 2.212200

C 5.720395 0.237035 1.854557

H 5.938364 0.794828 2.772518

C 4.617405 1.033480 1.133077

C 7.032587 0.208938 1.048330

F 3.477535 1.010749 1.859465

F 4.952212 2.327012 0.953354

F 4.322592 0.517501 -0.083886

F 6.900947 -0.466755 -0.105535

F 7.994629 -0.387082 1.772620

F 7.458389 1.458492 0.751052

95

ts5 Eopt -2742.075037

C -4.438889 6.991338 0.219325

C -3.118972 6.690152 2.353454

C -1.591804 6.073672 -2.324138

C -3.623073 4.550489 -2.423180

C -5.653479 -6.544545 -0.667748

C -9.048837 -2.346448 -1.216467

C 1.646981 1.059232 0.349962

C 0.957576 1.301553 1.549140

C 1.514359 2.012085 -0.670018

C 0.197621 2.463570 1.722852

C 0.772000 3.181923 -0.481256

C -5.417882 0.769119 0.881150

C -3.398883 0.360301 -0.363209

C -5.024973 2.084936 1.092551

C -2.990602 1.674213 -0.149944

C -4.647262 -3.948844 -0.160846

C -6.379871 -1.787587 -0.451601

C -2.848782 -2.478074 0.527566

C -0.648231 4.731579 0.960323

C -3.061218 6.633807 0.812340

C -2.281913 4.832497 -1.717158

C 0.106330 3.442231 0.725311

C -4.612648 -0.124042 0.150983

C -4.192010 -2.629557 0.077265

C -3.806003 2.551815 0.577729

C -5.051142 -1.521463 -0.073868

C -5.958448 -4.187602 -0.536660

C -6.844269 -3.080824 -0.678976

N -1.750949 -2.405240 0.902413

O -6.495791 -5.406454 -0.788657

O -8.103531 -3.396347 -1.052020

O -3.481677 3.845998 0.826321

Si -2.378847 4.967532 0.185825

H -4.407894 7.100193 -0.870065

H -5.185098 6.224065 0.457693

H -4.802052 7.942088 0.632423

H -2.135101 6.552240 2.815328

H -3.502724 7.663081 2.688489

H -3.786406 5.917296 2.752121

H -2.195045 6.979326 -2.185768

H -0.605529 6.264010 -1.882875

H -1.445035 5.944695 -3.404576

H -4.096896 3.635427 -2.054075

H -4.336536 5.371077 -2.289811

H -3.466602 4.429518 -3.503691

H -5.274655 -6.656027 0.356574

H -6.278131 -7.402980 -0.918483

H -4.807100 -6.492668 -1.364911

H -9.981674 -2.833387 -1.503584

H -9.197661 -1.792795 -0.280960

H -8.740524 -1.651294 -2.007338

H 1.001386 0.596404 2.369109

H 1.980664 1.865897 -1.637635

H -0.310641 2.617704 2.672751

H 0.734926 3.911186 -1.288587

H -6.352549 0.419074 1.311182

H -2.764097 -0.289442 -0.957416

H -5.643810 2.768847 1.664868

H -2.035961 2.009422 -0.540904

H -3.952174 -4.768883 -0.030791

H -7.045548 -0.945657 -0.595604

H -0.047893 5.582636 0.603997

H -0.768143 4.887228 2.039403

H -2.342108 7.398507 0.477140

H -1.625931 3.971471 -1.913207

C 3.490059 -1.040170 4.482496

H 4.197943 -1.877217 4.504042

H 2.796926 -1.152403 5.318858

C 2.732468 -1.083448 3.163073

O 3.306326 -0.466882 2.197967

O 1.642355 -1.699630 3.118662

Pd 2.713740 -0.673820 0.217721

C 2.970014 -3.757142 0.132838

O 1.722479 -3.877854 0.216131

O 3.637699 -2.672422 0.225303

C 3.795712 -5.011673 -0.131324

H 4.669272 -5.035423 0.527207

H 4.162330 -4.989219 -1.164546

H 3.192135 -5.909818 0.012279

Ag 0.441531 -2.327943 1.281473

H 4.059712 -0.112858 4.574497

C 4.581367 0.029053 -1.017861

C 5.616347 -0.885137 -0.825786

C 4.791870 1.410268 -1.047159

C 6.896826 -0.382330 -0.597707

H 5.414182 -1.946935 -0.801316

C 6.079129 1.884910 -0.816547

H 3.982285 2.104815 -1.217204

C 7.141476 0.996770 -0.591897

H 7.715672 -1.078259 -0.425499

H 6.277913 2.952617 -0.810181

I 2.761361 -0.827377 -2.472960

C 8.507746 1.508835 -0.361330

O 8.815393 2.686644 -0.353412

H 9.274434 0.720557 -0.189305

95

ts6 Eopt -2742.088966

C -3.457709 7.255786 0.509578

C -1.802108 6.825107 2.367748

C -1.356248 6.084128 -2.586024

C -3.448540 4.730108 -2.108780

C -6.749363 -5.681134 -0.648349

C -9.259833 -0.876331 -0.382939

C 2.256445 1.001076 0.102283

C 1.975137 1.597087 1.344957

C 1.857481 1.688225 -1.058704

C 1.314770 2.821320 1.417388

C 1.219357 2.923478 -0.976237

C -4.845542 1.293303 1.258978

C -3.108916 0.616153 -0.263931

C -4.199185 2.506292 1.463969

C -2.445369 1.822993 -0.054761

C -5.171912 -3.383377 -0.155983

C -6.450019 -0.914403 -0.026500

C -3.027604 -2.361069 0.303118

C 0.328662 4.899243 0.362282

C -2.022237 6.800726 0.840257

C -1.956379 4.919466 -1.767602

C 0.942774 3.526779 0.262675

C -4.320256 0.323801 0.384890

C -4.426356 -2.203866 0.084246

C -2.998029 2.787615 0.797622

C -5.053697 -0.942559 0.147049

C -6.545155 -3.331718 -0.325489

C -7.197172 -2.067242 -0.254479

N -1.893899 -2.547539 0.475560

O -7.350529 -4.396388 -0.559507

O -8.535043 -2.098851 -0.437052

O -2.416515 3.995291 1.030781

Si -1.555861 5.110801 0.090439

H -3.614055 7.393487 -0.565424

H -4.198961 6.530025 0.865888

H -3.679944 8.214347 0.997425

H -0.768135 6.595952 2.649009

H -2.037331 7.817948 2.773456

H -2.450608 6.098516 2.870047

H -1.831573 7.040717 -2.336227

H -0.277315 6.200011 -2.427256

H -1.509911 5.918216 -3.660266

H -3.885634 3.872620 -1.588516

H -4.041276 5.614501 -1.851465

H -3.571747 4.560231 -3.186862

H -6.252834 -5.957165 0.290993

H -7.566149 -6.377015 -0.844599

H -6.024034 -5.727980 -1.470978

H -10.304109 -1.146598 -0.544643

H -9.154336 -0.392412 0.596136

H -8.935128 -0.184027 -1.169746

H 2.292312 1.117153 2.260911

H 2.073110 1.280874 -2.038173

H 1.115743 3.254559 2.394993

H 0.970299 3.445094 -1.898047

H -5.764429 1.085157 1.800307

H -2.679708 -0.094692 -0.962663

H -4.607452 3.250215 2.140878

H -1.500691 2.011733 -0.554190

H -4.646937 -4.329689 -0.192881

H -6.944874 0.048526 -0.009810

H 0.806892 5.572200 -0.364576

H 0.551276 5.320621 1.349639

H -1.327051 7.528145 0.390366

H -1.435547 4.001786 -2.080697

C 2.806295 -1.600217 4.416321

H 3.302975 -2.569212 4.545199

H 1.922814 -1.578208 5.058357

C 2.388548 -1.462988 2.961008

O 3.293007 -0.927258 2.197553

O 1.271762 -1.853923 2.588791

Pd 2.822619 -1.022670 0.173696

C 3.060110 -4.151612 0.352417

O 3.586394 -2.988293 0.289025

O 1.845504 -4.432263 0.468432

C 4.056630 -5.299759 0.244239

H 3.631975 -6.211420 0.669146

H 4.996417 -5.044521 0.739560

H 4.271289 -5.472706 -0.816892

Ag 0.294202 -2.826269 0.578792

H 3.512251 -0.818057 4.705619

C 4.288357 0.432180 -0.226918

C 4.739304 0.506856 -1.549981

C 5.101904 0.837207 0.838594

C 6.032251 0.967049 -1.795308

H 4.111587 0.195491 -2.374338

C 6.386033 1.299298 0.573223

H 4.745011 0.766221 1.857046

C 6.864441 1.370519 -0.744008

H 6.395655 1.010096 -2.820329

H 7.037972 1.613038 1.383281

I 1.706723 -1.663093 -2.225664

C 8.226369 1.866890 -1.023430

O 9.013191 2.254526 -0.178899

H 8.505658 1.868013 -2.101281

**References**

1. Luchini, G.; Alegre-Requena, J. V.; Funes-Ardoiz, I.; Paton, R. S. GoodVibes: Automated Thermochemistry for Heterogeneous Computational Chemistry Data. F1000Research, **9**, 291 (2020).
2. Grimme, S. Supramolecular Binding Thermodynamics by Dispersion-Corrected Density Functional Theory, *Chem. Eur. J*. **18**, 9955-9964 (2012).
